# Photocatalytic, modular difunctionalization of alkenes enabled by ligand-to-metal charge transfer and radical ligand transfer†

**DOI:** 10.1039/d3sc05231a

**Published:** 2023-11-24

**Authors:** Kang-Jie Bian, David Nemoto, Xiao-Wei Chen, Shih-Chieh Kao, James Hooson, Julian G. West

**Affiliations:** a Department of Chemistry, Rice University 6100 Main St MS 602 Houston TX 77005 USA jgwest@rice.edu

## Abstract

Ligand-to-metal charge transfer (LMCT) is a mechanistic strategy that provides a powerful tool to access diverse open-shell species using earth abundant elements and has seen tremendous growth in recent years. However, among many reaction manifolds driven by LMCT reactivity, a general and catalytic protocol for modular difunctionalization of alkenes remains unknown. Leveraging the synergistic cooperation of iron-catalyzed ligand-to-metal charge transfer and radical ligand transfer (RLT), here we report a photocatalytic, modular difunctionalization of alkenes using inexpensive iron salts catalytically to function as both radical initiator and terminator. Additionally, strategic use of a fluorine atom transfer reagent allows for general fluorochlorination of alkenes, providing the first example of interhalogen compound formation using earth abundant element photocatalysis. Broad scope, mild conditions and versatility in converting orthogonal nucleophiles (TMSN_3_ and NaCl) directly into corresponding open-shell radical species are demonstrated in this study, providing a robust means towards accessing vicinal diazides and homo-/hetero-dihalides motifs catalytically. These functionalities are important precursors/intermediates in medicinal and material chemistry. Preliminary mechanistic studies support the radical nature of these transformations, disclosing the tandem LMCT/RLT as a powerful reaction manifold in catalytic olefin difunctionalization.

## Abstract 2

Simple iron salts are able to photocatalyze the diazidation, dichlorination, and fluorochlorination of alkenes *via* the merger of ligand-to-metal charge transfer (LMCT) and either radical ligand transfer (RLT) or fluorine atom transfer (FAT).

## Graphical abstract

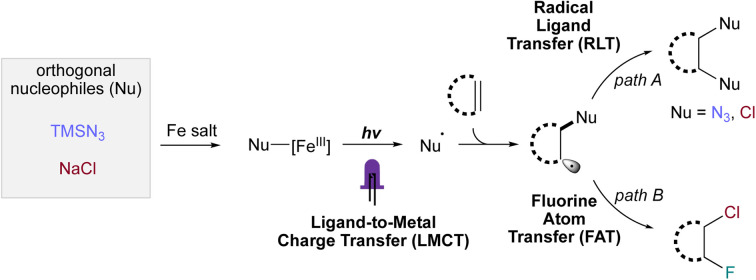

## Introduction

Radical difunctionalization of alkenes has allowed direct construction of complex molecules from simple, organic feedstock chemicals, establishing itself as a powerful strategy in enhancing molecular complexity.^1–7^ However, despite significant progress over the past decades, previous radical difunctionalization methods have encountered significant limitations, including the common requirement of the alkene component to be activated (the alkene adjacent to aromatic (aryl) or heteroatom (C=O, N, O) functionalities).^8^ The use of more common unactivated alkenes is less developed using a radical difunctionalization approach, with the selective functionalization of the transient alkyl radical intermediate formed after initial radical addition, presenting a particular challenge. Recently, our group has made progress in addressing this barrier through development of bio-inspired radical ligand transfer (RLT) catalysis, where a coordinated ligand on a metal center rebounds to the transient alkyl radical intermediate to selectively deliver the coordinated functional group.^9–11^ This strategy allows for efficient delivery of diverse nucleophiles (halogen, azide, thiocyanate), to transient alkyl radical intermediates, permitting modular functionalization of feedstock chemicals including diverse unactivated alkenes and carboxylic acids. However, in our scheme and many conventional photoredox transformations, the initial radical generation is promoted by the expensive, noble-metal based photocatalysis *via* outer-sphere single electron transfer (OSET), limiting the sustainability of the process.^12–16^ Further, the redox potential of both the photocatalyst and reactants must be carefully matched, limiting the possible functionalization reagents that can be utilized in this approach. To address unsustainability and rigid reaction manifold issues of previous methods and achieve a general, modular difunctionalization strategy, a new mechanistic pathway is required.

**Fig. 1:**
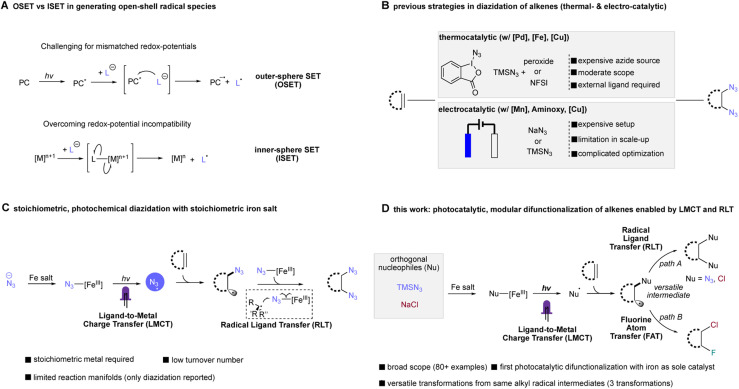
Previous methods of radical diazidation of alkenes and synopsis of the photocatalytic difunctionalization of alkenes (this work). (A) Divergent pathway of OSET and ISET in generating radical species. (B) Thermal-, electrochemical diazidation of alkenes. (C) Our previous photochemical diazidation of alkenes enabled by LMCT and RLT. (D) This work: the general photocatalytic difunctionalization of alkenes enabled by LMCT and RLT.

In comparison to conventional OSET photocatalysis, ligand-to-metal charge transfer (LMCT) is one reaction manifold under inner-sphere single electron transfer (ISET), where an electron is transferred to - or from- a metal to a directly coordinated substrate.^17,18^ This reaction pathway represents a direct, modular platform for nucleophiles to be converted to open-shell radical species, overriding redox-matching requirement in OSET photocatalysis, where challenging reactants such as anionic chloride can be net oxidized into corresponding radical form (Cl^−^/Cl˙, *E*^ox^_1/2_ = 2.03 V *vs.* SCE) with otherwise weakly oxidizing metals (Fig. 1A).^19,20^ Followed by Kochi's original finding of C–H chlorination and olefin dichlorination using a simple copper chloride complex,^21^ recent studies exploiting copper^20,22–25^ and other 3d metals such as nickel^26–28^ and cobalt^29^ have provided an alternative pathway in accessing important organic motifs. In contrast, the application of earth-abundant, cheap iron salts in LMCT transformations has been more limited, largely focusing on decarboxylation,^30–32^ C–H functionalization,^19,33,34^ generation of oxo-radical/utilization of alkyl alcohols^35,36^ and other specialized transformations.^37^ Toward further expanding this reactivity, we and the group of Shi recently leveraged the synergistic cooperation of iron-mediated LMCT and our RLT strategy to achieve the first photochemical alkene diazidation, representing important example leveraging iron LMCT reactivities in difunctionalization of alkenes (Fig. 1C).^38,39^ Nevertheless, a stoichiometric amount of iron salt was needed in most cases, with modest (2–3 turnover number) catalysis possible only through use of an elaborate continuous-flow strategy. With regard to increasing the practicality and safety of this reaction, it would be extremely desirable to develop a simple, photocatalytic version of this transformation using a common batch reaction setup. In addition to improved mass intensity, this catalytic approach would also improve the safety of azidation processes by limiting the concentration of metal azide species formed *in situ*. Furthermore, if a photocatalytic alkene diazidation could be achieved, we were excited that this same approach might be extensible to other nucleophiles such as chloride, providing a mild and direct synthesis of vicinal dichlorides, a product class that has found many applications in medicinal chemistry and can be used as a reactive handle for subsequent functionalization. Finally, this stepwise radical functionalization approach provides the opportunity to intercept the transient radical intermediate generated after LMCT in an orthogonal functionalization step such as fluorine atom transfer, delivering the first example of iron-photocatalyzed heterodifunctionalization of alkenes and opening the door to myriad other useful heterodifunctionalization reactions.

Achieving these aspirational transformations with a single catalyst system would represent a unified strategy for modular difunctionalization of alkenes, providing direct access to these useful products and offering insights into the generation of transient alkyl radicals and design principles for their subsequent transformations in a single catalytic manifold.

Both alkene diazidation and dichlorination have witnessed recent progress in catalytic reaction development. Recent reports in catalytic alkene diazidation by Greaney,^40^ Loh,^41^ Xu,^42^ Bao,^43^ Liu,^44^ significantly expanded the alkene scope tolerance, allowing facile diazidation at moderate temperature without strongly acidic conditions. In a groundbreaking study, Xu leveraged earth-abundant iron as a redox catalyst, achieving highly diastereoselective diazidation of alkenes with low catalyst loadings.^42^ However, these approaches require use of reactive and premade (or *in situ* generated) electrophilic azide sources to proceed, presenting some barriers to the use of these protocols (Fig. 1B). For alkene dichlorination, modern contributions by Borhan,^45^ Snyder,^46^ Hennecke,^47^ Nicolaou,^48^ Burns^49^ and Denmark^50^ have made progress addressing the harsh conditions required for classical dichlorination, including use of toxic molecular chlorine as the chlorination source. However, pursuing a radical-based approach to dichlorination has proven challenging due to the high potential of chloride oxidation to chlorine radical (*E*^ox^_1/2_ = 2.03 V *vs.* SCE) using an OSET process, a potential incompatible with most organic solvents and functional groups. Towards addressing these limitations, elegant studies in electrocatalysis achieved by the Lin group^51^ and the Xu group^52^ have offered an alternative strategy in accessing these important motifs where electricity serves as terminal oxidant and nucleophilic reactants can be used as radical precursors. While these advances are exciting, the high cost of electrochemical apparatuses and the required multivariate optimization of factors including electrode composition, morphology and mass transport provide significant challenges to the development of this strategy as a general approach to the modular difunctionalization of alkenes. Very recently, an elegant study by the Wan group^22^ disclosed a vicinal dichlorination of alkenes using copper photocatalysis, building on Kochi's initial stoichiometric CuCl_2_ finding.^21^ However, the transformation requires the use of corrosive and volatile HCl solution as the chloride source, limiting this approach to terminal unactivated alkenes with low ionization potential; activated alkenes were able to be engaged using superstoichiometric CuCl_2_. However, a general, photocatalytic strategy compatible with both activated and unactivated alkenes remains elusive.

Taken together, while important advances have been made in both alkene diazidation and dichlorination, a general, unified catalyst system able to achieve diverse alkene functionalization (including both diazidation and dichlorination) for activated and unactivated alkenes remains purely aspirational. Considering other desirable traits of such a hypothetical system, it would be ideal to use only cheap, earth abundant catalysts, be able to achieve different difunctionalization results based only on the identity of simple and safe nucleophilic reagents, and use a simple and readily-available reaction apparatus. Finally, if such a system could be modified to allow heterodifunctionalization in addition to homodifunctionalization, the synthetic value and applicability of this unified scheme would be markedly enhanced. As a research group concerned with the development of appealing reactions, we have challenged ourselves to incorporate these characteristics as design goals for catalysis.

Herein, we report our effort to realize this aspiration with a general and modular photocatalytic difunctionalization system, enabling diazidation and dichlorination of a broad range of alkenes using a simple earth-abundant element photocatalyst. Leveraging the merger of ligand-to-metal charge transfer and radical ligand transfer, we were able to utilize orthogonal nucleophiles such as TMSN_3_ or sodium chloride in alkene diazidation and dichlorination with cheap, low-toxicity, earth-abundant iron as sole catalyst, addressing challenges in state-of-art unsustainable protocols, such as use of superstoichiometric metals, corrosive chloride sources (such as HCl) and other substrate scope limitations for both diazidation and dichlorination. Importantly, this system is also able to achieve heterodifunctionalization of alkenes with simple adjustment of reactant loading, enabling direct access to vicinal fluorochlorides in good yield and regioselectivity, offering a pathway to accessing interhalogen compounds in excellent regioselectivity, a significant challenge using previous strategies (Fig. 1D).

## Results and discussion

### Reaction design and optimization

We first set out to explore the possibility of photocatalytic diazidation by using *N*-butenyl phthalimide as our test substrate, nucleophilic trimethylsilyl azide (TMSN_3_) as the azide source, different commercially available iron salts as the catalyst, and different organic terminal oxidants under 390 nm LED irradiation at room temperature. To our delight, we found that the commercially-available reagent Selectfluor is able to function as a terminal oxidant for the photocatalytic diazidation of alkenes in batch configuration, permitting formation of 86% of our desired diazide product 1 using only 10 mol% of cheap and abundant Fe(OAc)_2_ (Table 1, entry 1). Screening of different iron salts indicated Fe(OAc)_2_ was the optimal catalyst, with other common iron salts giving moderately to severely reduced product formation (Table 1, entry 2 and Table S1†). Importantly, the loading of oxidant is crucial to this photocatalytic diazidation, with more than 1 equivalent of Selectfluor leading to significantly decreased yield (Table 1, entries 3–5). Importantly, with more Selectfluor addition to the system, lower mass balance was observed while no fluoroazidation product was detected (for details, see ESI†). Further screening indicated the catalyst loading (entries 6 and 7) can be reduced to as low as 2.5 mol%, giving diazide 1 in 72% yield. Considering the low price and sustainability of the iron salt, we proceeded with 10 mol% of Fe(OAc)_2_ in our standard conditions due to its slightly increased yield (Table 1, entry 1). Single-electron oxidants other than Selectfluor were also screened; however, these afforded significantly less efficient transformations (Table 1, entries 8 and 9). We also observed a strong solvent effect for the diazidation, with DCM and THF unsuccessful for the reaction (Table 1, entries 10 and 11) and ethyl acetate and acetone able to mediate formation of diazide 1, albeit with lower (16–44%) yield (Table 1, entries 12 and 13). We next endeavored to determine the necessary elements of our photocatalytic diazidation through control experiments. Almost no conversion was observed when the reaction was carried out with either oxidant in the absence of iron catalyst (Table 1, entry 14) or using iron with oxidant in the dark (Table 1, entry 15), indicating a divergent mechanistic pathway from previously-disclosed thermochemical diazidation methods.^40–44^ When running the reaction only with 10 mol% iron salt without oxidant under light irradiation, we observed 20% yield of diazidation product, a result consistent with our previous stoichiometric photochemical diazidation (Table 1, entry 16).^38^ Notably, this photocatalytic diazidation does not require supporting ligands or expensive electrochemical apparatuses, providing an exceptionally simple and robust strategy for accessing vicinal diazides.

**Table 1:** Optimization of the photocatalytic diazidation of alkenes^a^

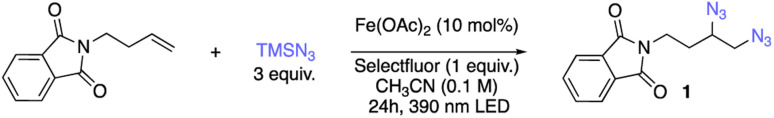		
Entry	Deviation from standard conditions	Yield^b^ (%)
1	None	88 (86)
2^c^	FeCl_3_·6H_2_O, Fe(acac)_3_, Fe(NO_3_)_3_·9H_2_O, Fe(OTf)_2_, FeCl_2_	24–50
3	2.0 equiv. of Selectfluor with 20 mol% Fe(OAc)_2_	62
4	1.5 equiv. of Selectfluor with 20 mol% Fe(OAc)_2_	76
5	1.0 equiv. of Selectfluor with 20 mol% Fe(OAc)_2_	80
6	5 mol% of Fe(OAc)_2_	76
7	2.5 mol% of Fe(OAc)_2_	72
8	[Py–F]	16
9	NFSI	44
10	DCM	8
11	THF	ND
12	EA	16
13	Acetone	44
14	No iron salt	12
15	In the dark	ND
16	No Selectfluor	20

a Reaction conditions: alkene (0.1 mmol, 1.0 equiv.), TMSN_3_ (3.0 equiv.), Fe catalyst (10 mol%), oxidant (1.0 equiv.) and solvent (0.1 M), 24 h, RT, 390 nm Kessil LED (25% intensity).

b ^1^H NMR yield was determined by using CH_2_Br_2_ as an internal standard. Isolated yield in the parentheses.

c 20 mol% of iron salt, 2 equiv. of Selectfluor.

Arriving at an optimized set of conditions for the photocatalytic diazidation, we were encouraged to expand this synergistic LMCT/RLT system to another valuable transformation: dichlorination. Organochlorides are prevalent in many bioactive compounds and have been widely used as important reaction intermediates.^45–50^ As the dominant form of chlorine in nature is chloride (Cl^−^), many recent dichlorination efforts have been devoted to using nucleophilic chloride as the chlorine source. However, the most common form of nucleophilic chloride, NaCl, has been severely underexplored in this transformation. Bolstered by our successful development of a photocatalytic diazidation enabled by a tandem catalytic LMCT/RLT process, where the azido radical is generated upon the irradiation of an *in situ* formed Fe^III^–N_3_ species, we envision the combination of common chloride nucleophiles and iron salts could also lead to the formation of an analogous Fe^III^–Cl, which is capable of generating chlorine radical *via* ligand-to-metal charge transfer, a process studied by the Shul'pin group^53^ and more recently the Rovis group^33^ for C–H functionalization. The *in situ* generated transient alkyl radical formed by Cl˙ addition to alkenes could then be sequestered by Fe^III^–Cl species, delivering second equivalent of chloride *via* radical ligand transfer, analogous to our diazidation approach. We were delighted to see the unified method could also function well in photocatalytic dichlorination using NaCl as the chloride source (for details, see ESI†), suggesting this catalyst system to be capable of general difunctionalization.

### Scope of photocatalytic diazidation and dichlorination of alkenes

**Scheme 1:**
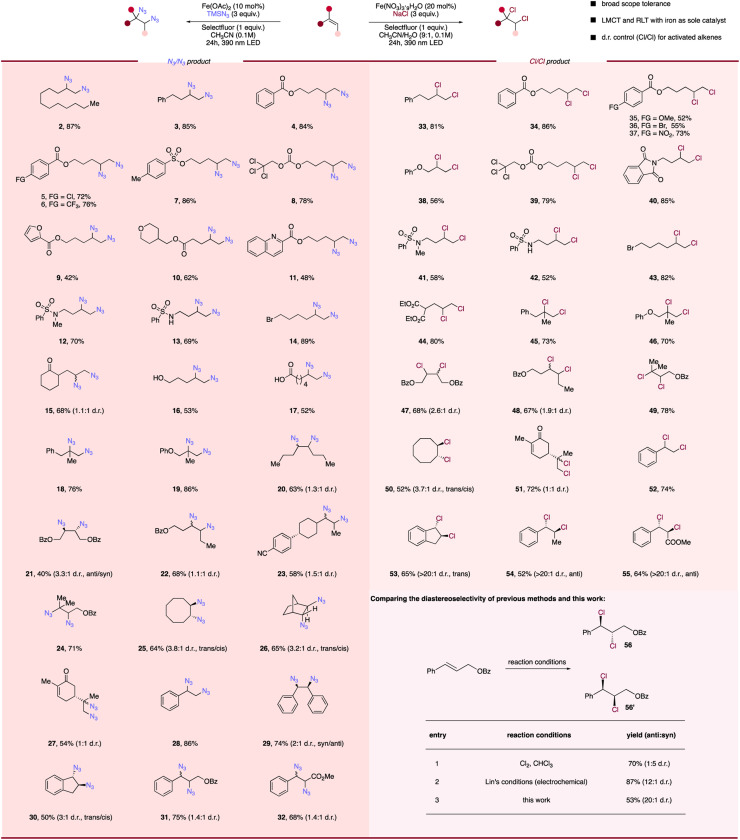
Scope of photocatalytic diazidation and dichlorination of alkenes. Reaction conditions of diazidation of alkenes: alkene (0.1 mmol, 1.0 equiv.), TMSN_3_ (3.0 equiv.), Fe(OAc)_2_ (10 mol%), Selectfluor (1 equiv.) and CH_3_CN (0.1 M), 24 h, RT, 390 nm Kessil blue LED. Reaction conditions of dichlorination of alkenes: alkene (0.1 mmol, 1.0 equiv.), NaCl (3.0 equiv.), Fe(NO_3_)_3_·9H_2_O (20 mol%), Selectfluor (1 equiv.) and CH_3_CN/H_2_O (9 : 1, 0.1 M), 24 h, RT, 390 nm Kessil blue LED. Diastereoselectivity (d.r.) is determined by ^1^H NMR.

With two complementary difunctionalization conditions in hand, we next sought to evaluate the substrate tolerance of this photocatalytic system for diverse alkenes (Scheme 1). In both transformations, moderate to excellent yields of diazidated and dichlorinated products can be generated from unactivated alkenes (2–27; 33–51) and activated alkenes (28–32; 52–56). First, simple aliphatic alkenes (2, 3, 33) and aryl moieties bearing functional groups with various electronic properties such as methoxy (35), chloro (5), bromo (36), trifluoromethyl (6) and nitro (37) could afford corresponding diazidation and dichlorination products in good efficiency. Useful protecting groups including benzoate (4, 34), tosylate (7), and reductively labile 2,2,2-trichloroethoxycarbonyl (Troc) (8, 39) were also tolerated, indicating that this method is amenable to diversely protected molecules. Unactivated alkenes containing heterocycles that are prevalent in medicinal chemistry settings including furan (9), tetrahydropyran (10), *N*-phthalimide (40) and strongly coordinating quinoline (11) were all compatible with our diazidation or dichlorination protocols. Next, we sought to test the alkenes featuring diverse labile functionalities that are susceptible to deprotonation, oxidation, reduction, and nucleophilic substitution to further probe the generality of these methods. When acidic hydrogen was present in sulfonamide-containing substrates (13, 42), the reaction behaved well, giving the diazidated product in 69% yield and dichlorinated product in 52% yield. Furthermore, substrates bearing an unhindered primary alkyl bromide handle (14, 43), a common target for nucleophilic substitution, were converted to corresponding products smoothly (89% and 82%), with no competitive nucleophile displacement. The substrates containing enolizable ketone (15), oxidation-labile primary alcohol (16), carboxylic acid (17) and ester (44) were also preserved in our system, affording difunctionalized products in moderate to good yields. Following our investigation of the functional group tolerance, substitution patterns of alkenes were next evaluated. 1,1- (18, 19, 45, 46) and 1,2-disubstituted (20–23, 47, 48) alkenes were found to be tolerated under the standard conditions, offering corresponding products smoothly. Importantly, sterically hindered tri-substituted (24, 49) alkenes also behave well, giving organic diazides or dichlorides in high yields. Notably, the high efficiency of our protocol towards sterically-hindered alkenes provides a simple solution to accessing tertiary azides and chlorides which would be difficult to obtain *via* direct nucleophilic substitution. Furthermore, the system is also compatible with cyclic or bridged alkenes, where the diazidated products (25, 26) and dichlorinated products (50) could be easily accessed. With two olefinic functionalities presented in the substrates, such as natural product *R*-carvone (27, 51), our method was able to provide diazidation or dichlorination product at the electron-rich site while the electron-deficient conjugated olefin remains intact.

Reminiscent of the high efficiency of both protocols for unactivated alkenes, styrene-type activated alkenes (28–32, 52–56) functioned well in both difunctionalization systems. Notably, recent study by the Wan group has shown that activated alkenes can be dichlorinated in high yields with CuCl_2_; however, superstoichiometric, reagent quantities of copper is required for successful transformation in this case.^22^ Thus, a highly efficient, diastereoselective dichlorination of activated alkenes *via* a photocatalytic process remains elusive. To our delight, reaction of styrene (28, 52), stilbene (29), indene (30, 53), cinnamyl benzoate (32, 56) and methyl cinnamate (33, 55) all led to corresponding diazides or dichlorides being formed in moderate to high yields with earth-abundant iron salt in catalytic quantities, addressing the limitations of Wan's approach. Interestingly, we also found almost exclusive *trans*- (*anti*-) isomer generation with 1,2-disubstituted alkenes in our photocatalytic dichlorination protocol, showing a divergent stereoselectivity to Cl_2_ addition and greatly enhanced diastereoselectivity compared with previous electrochemical dichlorination.^54^ The simplicity and generality of both protocols towards diverse functional groups while using cheap, earth-abundant iron as sole catalyst demonstrates that tandem LMCT/RLT can serve as a powerful solution to radical olefin difunctionalization.

### Scope of photocatalytic diazidation and dichlorination of alkenes derived from pharmaceuticals and natural products

**Scheme 2:**
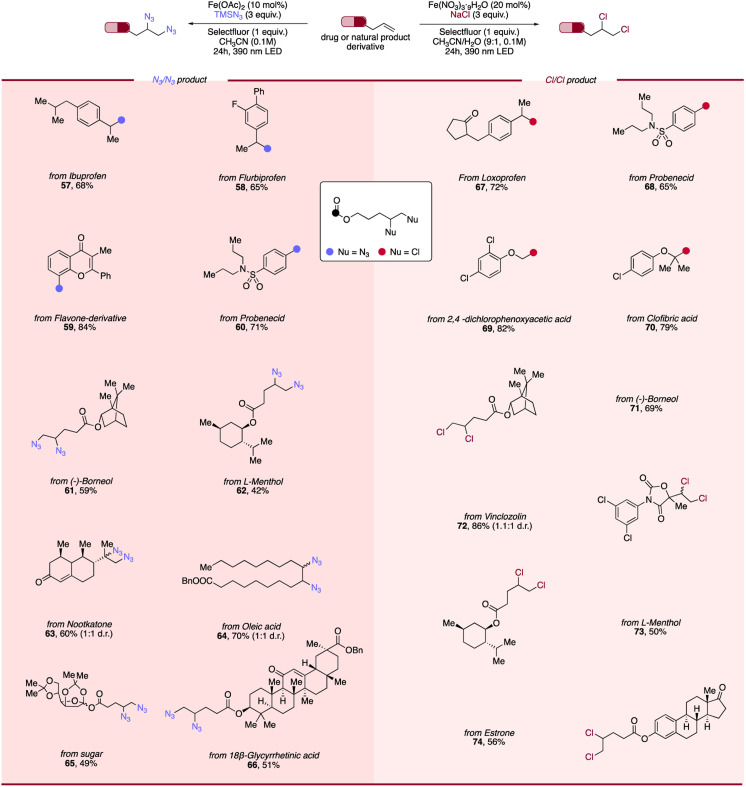
Scope of photocatalytic diazidation and dichlorination of alkenes derived from pharmaceuticals and natural products. Reaction conditions of diazidation of alkenes: alkene (0.1 mmol, 1.0 equiv.), TMSN_3_ (3.0 equiv.), Fe(OAc)_2_ (10 mol%), Selectfluor (1 equiv.) and CH_3_CN (0.1 M), 24 h, RT, 390 nm Kessil blue LED. Reaction conditions of dichlorination of alkenes: alkene (0.1 mmol, 1.0 equiv.), NaCl (3.0 equiv.), Fe(NO_3_)_3_·9H_2_O (20 mol%), Selectfluor (1 equiv.) and CH_3_CN/H_2_O (9 : 1, 0.1 M), 24 h, RT, 390 nm Kessil blue LED. Diastereoselectivity (d.r.) is determined by ^1^H NMR.

Recognizing the desirable properties engendered by the incorporation of versatile handles such as azide or chloride in bioactive molecules, we next endeavored to investigate an array of alkenes derived from commercially available active pharmaceutical ingredients (APIs) and natural products to evaluate both protocols' viability in late-stage modification (Scheme 2). To our delight, NSAID-derived alkenes including ibuprofen (57), flurbiprofen (58), probenecid (60, 68), and lipid-lowering clofibric acid (70) all functioned smoothly, giving difunctionalized products in good efficiency. Additionally, natural products-derived alkenes such as flavone (59), (−)-borneol (61, 71), l-menthol (62), oleic acid (64) and a protected sugar (65) underwent successful conversion to their corresponding products in moderate to good yields. Moreover, alkenes containing herbicides fragments including nootkatone (63), 2,4-dichlorophenoxy acetic acid (69) and vinclozolin (72) also showed broad compatibility, giving diazidation and dichlorination products in good yields. Steroid-derived alkenes such as 18-glycyrrhetinic acid (66) and estrone (74) were also explored, with moderate yields of corresponding diazides/dichlorides obtained using our approach. The excellent tolerance of our LMCT/RLT system towards diverse types of APIs, herbicides and natural products combined with the low toxicity of iron recommends its use for medicinal chemistry campaigns and provides a powerful strategy for late-stage modification of bioactive molecules.

### Scope of photocatalytic fluorochlorination of alkenes

The incorporation of fluorine atom into organic molecules has emerged as a powerful method of enhancing the metabolic stability, lipophilicity, and bioavailability of parent compounds, allowing for the design and development of potent pharmaceuticals, agrochemicals, and advanced functional materials.^55,56^ Although important advances have been made in developing efficient protocols to access fluorine-containing chemicals, the regioselective construction of C(sp^3^)–F bond remains challenging. One approach to accessing diverse aliphatic monofluorides is through the derivatization of fluorine-containing vicinal heterodihalide compounds, where one reactive halide (*e.g.* C–Cl) can participate in conventional nucleophilic substitution or cross-coupling reactions while the strong C–F bond remains intact. This building-block strategy has the benefit of the heterodihalide synthon serving as a starting point for a wide array of monofluorinated compounds *via* downstream chemistry. However, traditional pathways to prepare these synthons largely utilize *N*-chloro-succinimide to form *in situ* a chloronium intermediate, followed by fluoride substitution^57^ or other modification of halide-containing precursors.^58^ While previously demonstrated, this approach often proceeds with limited fluoride addition regioselectivity and the need for multiple steps. To overcome these challenges, it would be extremely useful to develop a facile, general method to achieve regioselective preparation of diverse fluorine-containing heterodihalides. Encouraged by the high efficiency of our photocatalytic difunctionalization system, we became intrigued whether we could design a photocatalytic fluorochlorination, extending our catalyst system to heterodifunctionalization reactions. First, we envisioned leveraging the disclosed LMCT reactivity of Fe^III^–Cl, formed *in situ* using a simple iron salt and cheap, safe NaCl, to facilitate chloro radical addition to an alkene, giving a chorine-containing alkyl radical intermediate. In diverting this intermediate to C–F bond formation, we envisioned that Selectfluor, already serving as a single-electron oxidizing agent, might also sequester this transient alkyl radical and outcompete the RLT *via* a fluorine atom transfer (FAT) process.^59^ Excitingly, as the chloro radical is known to have a strong preference to add to the less substituted position of the alkene and generate the more substituted radical, this tandem LMCT/FAT pathway presents the chance to access diverse chloro/fluoro interhalogen compounds regioselectively from wide range of olefins.

**Scheme 3:**
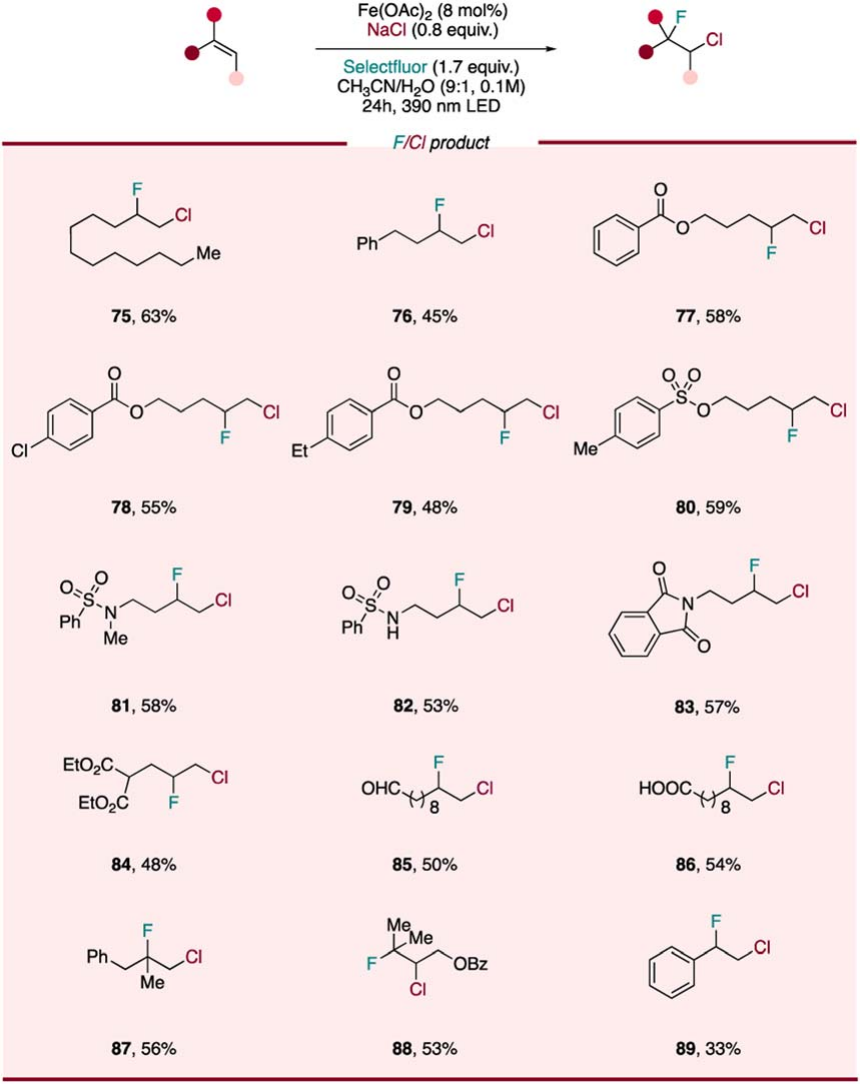
Scope of photocatalytic fluorochlorination of alkenes. Reaction conditions of fluorochlorination of alkenes: alkene (0.1 mmol, 1 equiv.), NaCl (0.8 equiv.), Fe(OAc)_2_ (8 mol%), Selectfluor (1.7 equiv.) and CH_3_CN/H_2_O (9 : 1, 0.1 M), 24 h, RT, 390 nm Kessil blue LED.

To our delight, slight tuning of the dichlorination reaction conditions (mainly increasing Selectfluor loading and decreasing NaCl loading) allows for fluorochlorination to be performed in high yield and selectivity (for details, see ESI†). We next sought to evaluate the scope of this heterodifunctionalization reaction (Scheme 3) and found aliphatic terminal alkenes (75, 76), along with substrates with substituted aryl and various protecting groups (77–80) were all tolerated, providing corresponding heterodihalides in 45–63% yield. Interestingly, we did not observe any C–H functionalization products for a substrate bearing a weaker benzylic C–H bonds (79), highlighting the chemoselectivity of this photocatalytic protocol and showing a divergence from the reactivity found by Rovis.^33^ As observed with the diazidation and dichlorination procedures, functional groups including sulfonamide (82), nitrogen-containing heterocycle (83), oxidatively labile ester (84) and aldehyde (85) are tolerated, with chloro-fluorinated products being isolated in moderate to good yields. Even a free carboxylic acid (86), which is liable to undergo decarboxylation under LMCT conditions, was found to be intact with only heterodihalide product observed. More substituted alkenes, such as 1,1-disubstituted (87) and tri-substituted alkene (88) were also interrogated, displaying similar reactivity as the other less-substituted substrates. Styrene (89) was also tolerated and provided synthetically useful yields of heterodihalide product, showing that activated alkenes can be engaged in this fluorochlorination reaction. Compared with previous strategies, our fluorochlorination protocol provides an efficient and regioselective alternative to accessing these useful fluorine-containing interhalogen synthons with applications in late-stage fluorine incorporation into pharmaceuticals and natural products.

### Mechanistic studies and proposed mechanism

**Scheme 4:**
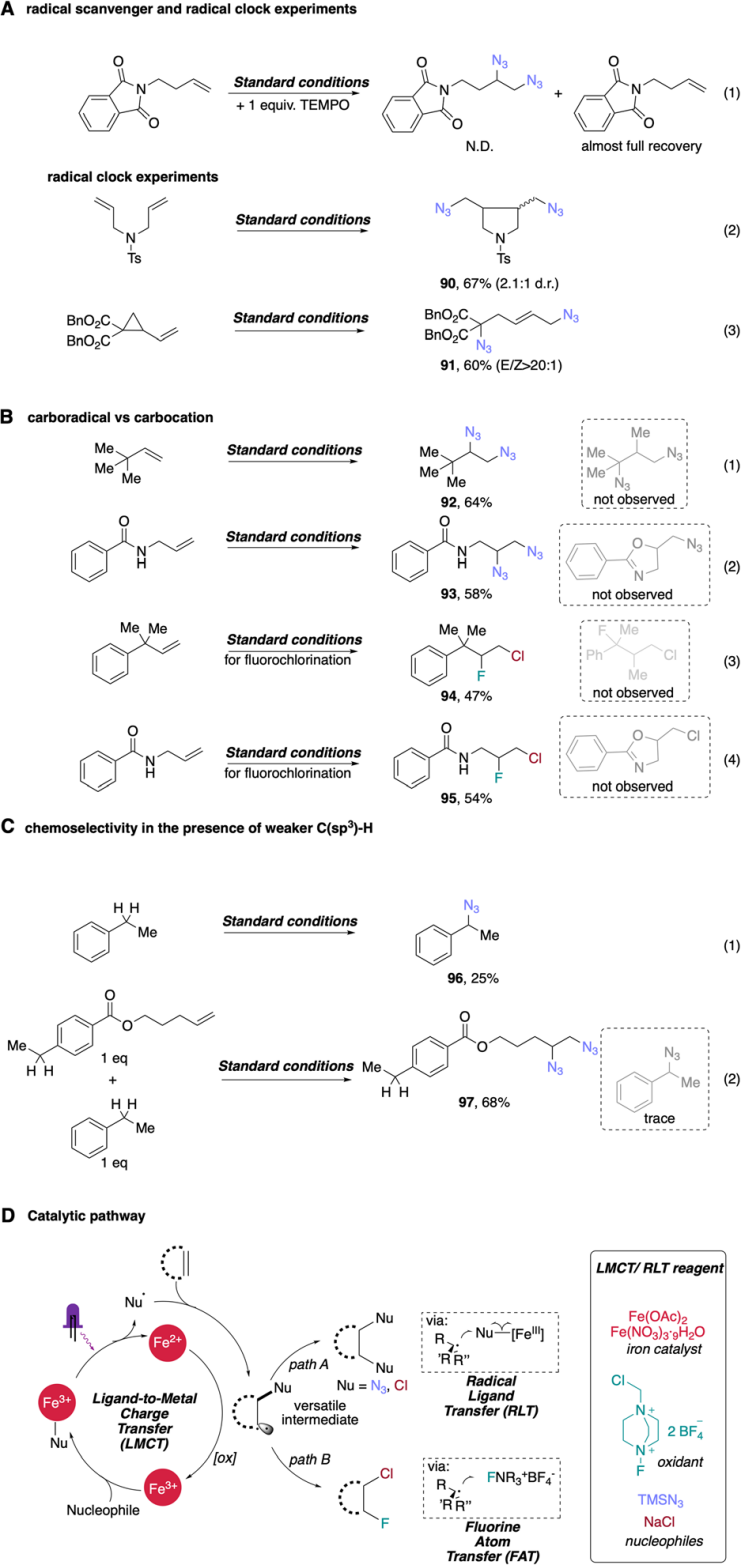
Mechanistic studies and proposed reaction pathway. (A) Radical scavenger and radical clocks experiments. (B) Validating radical ligand transfer instead of radical polar-crossover in subsequent functionalization. (C) Radical diazidation of alkenes shows significant chemoselectivity over radical azidation of benzylic C–H bond. (D) Proposed reaction mechanism.

The efficient conversion of starting material as well as characteristic product profiles led us to explore the mechanistic details of our difunctionalization system (Scheme 4). First, adding 1 equivalent of the radical scavenger 2,2,6,6-tetramethyl-1-piperidinyloxy (TEMPO) to our standard conditions for diazidation completely suppressed the reaction. Alkene starting material was fully recovered, supporting the presence of radical intermediates in the reaction (eqn (1), Scheme 4A). We sought further evidence to support a radical pathway by exploring the reaction of two different radical clock substrates. An *N*-tosylated-tethered diene furnished 5-*exo*-trig cyclization product 90 in 67% yield (eqn (2), Scheme 4A), consistent with radical cyclization. In another experiment, a cyclopropyl-substituted alkene underwent facile ring opening upon initial azide radical addition, giving the expected product 91 with excellent *E*/*Z* selectivity in 60% yield (eqn (3), Scheme 4A). These results are also consistent with our previous work and that of others,^38,39^ strongly supporting the intermediacy of radicals in this photocatalytic difunctionalization process. Importantly, in these entries, rearrangement products were the only products found in both entries, leading us to postulate that an iron-mediated, azido ligand transfer takes place after radical rearrangement in both cases, indicating the rate of radical ligand transfer must be slower than the slower rearrangement rate constant, 2 × 10^5^ s^−1^ (approx. for 5-*exo*-trig), in our system. After obtaining this compelling evidence supporting the radical nature of the first azide addition, we next considered whether the second azide might be delivered *via* a radical-polar crossover (RPC) process, where the carbon-centered radical intermediate can be oxidized to a carbocation, facilitating S_N_1-type addition of anionic azide. To test this possibility, we first subjected 3,3-dimethylbut-1-ene to our standard diazidation conditions, as the quaternary alkyl *tert*-butyl group can undergo a ‘1,2-methyl shift’ upon the generation of an adjacent carbocation. However, no migration was observed in this case, with 1,2-diazide 92 formed in 64% yield (eqn (1), Scheme 4B). In addition to this substrate, we also carried out the diazidation on unprotected amide-tethered alkenes, where a cyclized byproduct could be obtained if the *in situ* generated carbon radical is further oxidized into carbocation; however, only 1,2-diazidation product 93 was observed in this condition (eqn (2), Scheme 4B). Considering these two entries, the delivery of second azide is less likely to occur *via* the RPC pathway, a result consistent with our radical ligand transfer proposal. To analogously interrogate the possible mechanism of our fluorochlorination reaction, we also conducted standard conditions with two alkenes that are labile to rearrangement or ring closing upon the generation of cation intermediates. In these entries, direct fluorochlorination products 94 and 95 were obtained in moderate yields with no rearrangement/ring-closing byproducts obtained, supporting facile fluorine atom transfer following chlorine radical addition to the alkene and disfavoring a tandem LMCT and radical polar crossover (RPC) pathway.

Interestingly, when we subjected ethyl benzene to our standard conditions, direct benzylic C–H azidation product 96 was afforded in low, though appreciable (25%) yield, presumably *via* hydrogen-atom-transfer (HAT) followed by iron-catalyzed azido-RLT (eqn (1), Scheme 4C). This result supports generation of free azide radical (*i.e.*
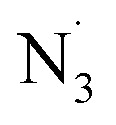
), a competent HAT reagent, *via* an LMCT process. To interrogate the chemoselectivity for unactivated alkenes in the presence of weaker benzylic C–H bonds, we next subjected a substrate with both olefin and benzylic C–H bonds and ethyl benzene in one-pot where high yield of corresponding vicinal diazide 97 was observed with the benzylic site remaining essentially unreacted in this competition, with only trace amounts of benzylic C–H azidated product. This result indicates a high chemoselectivity for photocatalytic diazidation of alkenes over C–H azidation (eqn (2), Scheme 4C).

Based on the collective mechanistic evidence and literature studies,^17,18,42,60,61^ we propose a possible pathway for photocatalytic difunctionalization with iron as sole catalyst (Scheme 4D). First, coordination of nucleophilic reagent to the dissolved iron salt produces corresponding ‘iron–ligand’ complex, which is capable of photoinduced LMCT, converting nucleophiles (TMSN_3_, NaCl) to their radical forms (
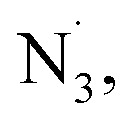
 Cl˙) accordingly. These radicals can then perform radical addition to diverse alkenes, forming a new alkyl radical intermediate. The *in situ* formed, transient alkyl radical intermediate can then participate in two divergent pathways, leading to homodifunctionalization or heterodifunctionalization. In pathway A, the transient intermediate can be sequestered by a high-valent iron–Nu species *via* radical ligand transfer to provide diverse homodifunctionalized products. Alternatively, in pathway B, increasing the amount of atom-transfer reagent Selectfluor could outcompete the iron-catalyzed RLT process, to generate heterodifunctionalized product *via* fluorine atom transfer. The utilization of a common, transient alkyl radical intermediate indicates the potential of this photocatalytic protocol to be extended to many difunctionalizations and its prospective application in preparing a library of molecules from orthogonal, cheap/safe nucleophiles promoted by one general catalytic manifold.

## Conclusion

In summary, we have developed a general strategy for the photocatalytic difunctionalization of a broad range of alkenes. Driven by iron-catalyzed ligand-to-metal charge transfer and radical ligand transfer, orthogonal, cheap and safe nucleophiles can be utilized in preparing vicinal diazides and dichlorides, addressing issues of substrate scope limitations, harsh conditions, and complicated setup in thermal- and electrochemical difunctionalization. Importantly, heterofunctionalization is also successful using this catalyst system, with our fluorochlorination synthesis representing the first photocatalytic solution to accessing interhalogen compounds with striking regioselectivity. Key to these transformations is iron playing dual and synergistic roles as radical initiator and terminator, showcasing ligand-to-metal charge transfer between iron and common nucleophiles as an important strategy in radical generation and iron-catalyzed radical ligand transfer offers a powerful platform for functionalizing transient alkyl radical intermediates. Future studies driven by iron-catalyzed ligand-to-metal charge transfer and radical ligand transfer are ongoing in our lab.

## Data availability

All supporting data has been uploaded as part of the ESI.†

## Author contributions

K.-J. B. designed the project. K.-J. B., D. N. Jr, X.-W. C., S.-C. K. and J. H. performed the experiments. K.-J. B., D. N. Jr and J. G. W. wrote the manuscript. J. G. W. directed the project. All authors interpreted the results in the manuscript.

## Conflicts of interest

There are no conflicts to declare.

## Supplementary Material

SC-015-D3SC05231A-s001
