# Supplementary material for: Photocatalytic, modular difunctionalization of alkenes enabled by ligand-to-metal charge transfer and radical ligand transfer
Source: Chem Sci. 2023 Nov 24;15(1):124–33. doi: 10.1039/d3sc05231a (PMC10732012; doi:10.1039/d3sc05231a)
Supplement: SC-015-D3SC05231A-s001 [file SC-015-D3SC05231A-s001.pdf]

Electronic Supplementary Material (ESI) for Chemical Science.
This journal is © The Royal Society of Chemistry 2023

Photocatalytic, Modular Difunctionalization of Alkenes Enabled by Ligand-to-Metal Charge Transfer and Radical Ligand Transfer

Kang-Jie Bian, David Nemoto Jr., Xiao-Wei Chen, Shih-Chieh Kao, James Hooson and Julian G. West*

Department of Chemistry, Rice University, 6500 Main St, Houston, TX-77005, USA.

Contents

I. General Procedures for Substrate Synthesis 3
General Procedure 1 for the Synthesis of Unactivated Alkenes 3
General Procedure 2 for the Synthesis of Unactivated Alkenes 3
Procedure for the Synthesis of N-(but-3-en-1-yl)benzenesulfonamide 4
Procedure for the Synthesis of N-(but-3-en-1-yl)-N-methylbenzenesulfonamide 4
Procedure for the Synthesis of 2-(but-3-en-1-yl)isoindoline-1,3-dione 5
II. Optimization of Conditions (Photocatalytic Diazidation) 6
2.1 optimization of iron salts 6
2.2 optimization of the amount of oxidant 7
2.3 optimization of iron catalyst loading 7
2.4 optimization of wavelength 8
2.5 control experiments 8
III. Optimization of Conditions (Photocatalytic Dichlorination) 9
3.1 optimization of iron salts 9
3.2 control experiments 10
IV. Optimization of Conditions (Photocatalytic Fluorochlorination) 11
4.1 optimization of iron salts 11
4.2 optimization of catalyst loading 12
4.3 optimization of reactants loading (alkene) 12
4.4 optimization of reactants loading (Selectfluor) 13
V. Experimental Methods of Difunctionalization of Alkenes 13
VI. Preliminary Testing on Photocatalytic Fluoroazidation 15
VII. Characterization of Corresponding Products 18

VIII. NMR Spectrum for New Compounds 48
IX. References 266

General Information

All reagents were purchased from commercially available sources and used without further purification. All reactions were monitored by either ^{1}H NMR or thin layer chromatography (TLC) carried out on 0.25 mm pre-coated silia plates (F-254) purchased from Silicycle, Quebec, Canada, using shortwave UV light as visualizing agent and KMnO4 or phosphomolybdic acid (PMA) as developing agents. Flash column chromatography was performed using SiliaFlash-P60 silica gel (40 – 63 μm) purchased from Silicycle, Quebec, Canada and preparatory thin-layer chromatography was purchased from Miles Scientific (GF 1000 μm, 20 x 20 cm). ^{1}H, ^{13}C and ^{19}F NMR spectra were recorded on a Bruker DRX-600 spectrometers operating at 600 MHz for proton nuclei, 151 MHz for carbon nuclei and 565 MHz for fluorine nuclei were calibrated using residual undeuterated solvent as an internal reference (CDCl3: 7.26 ppm ^{1}H NMR and 77.00 ppm ^{13}C NMR). 25 W PR160L 427 nm LEDs from Kessil Lights were used as light source. For reporting NMR peak multiplicities, the following abbreviations were used: s = singlet, d = doublet, t = triplet, q = quartet, quin = quintet, hept = heptet, m = multiplet. High-resolution mass spectra (HRMS) were recorded on an Agilent UHPLC TOF mass spectrometer using electrospray ionization time-of-flight (ESI-TOF), chemical ionization time-of-flight (CI-TOF) or atmospheric pressure chemical ionization (APCI).

I. General Procedures for Substrate Synthesis

General Procedure 1 for the Synthesis of Unactivated Alkenes

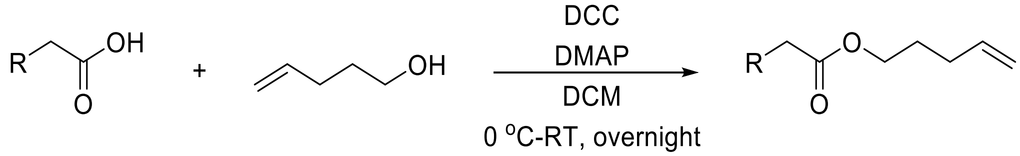

To an RB flask were added 5-pentenol (4.9 mmol, 1.96 equiv), carboxylic acid (2.5 mmol, 1.0 equiv), 4-dimethylamino pyridine (0.24 mmol, 0.097 equiv), and a stir bar. The RB flask was then evacuated and backfilled with nitrogen gas three times. Dry dichloromethane (0.225 M) was added via syringe to the RB flask, dissolving the solid components. The RB flask was then placed in an ice bath positioned on top of a stirring plate. Dicyclohexyl carbodiimide (4.85 mmol, 1.94 mmol) was added to the mixture via syringe dropwise over a period of 5 minutes. The ice bath was then removed, allowing the reaction to return to room temperature. The reaction was left to stir overnight. Following reaction, the mixture was concentrated through rotary evaporation. Subsequent flash column chromatography (hexanes/EtOAc) allowed for isolation of the ester.[1-2]

General Procedure 2 for the Synthesis of Unactivated Alkenes

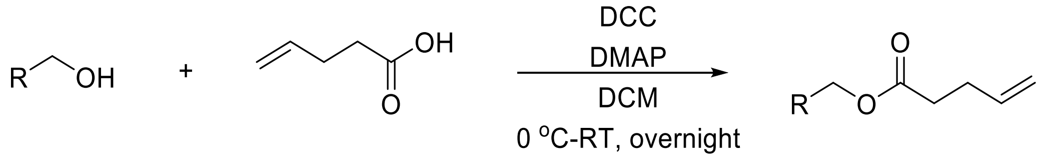

To an RB flask were added 5-pentenoic acid (2.5 mmol, 1.0 equiv), alcohol (4.9 mmol, 1.96 equiv), 4-dimethylamino pyridine (0.24 mmol, 0.097 equiv), and a stir bar. The RB flask was then evacuated and backfilled with nitrogen gas three times. Dry dichloromethane (0.225 M) was added via syringe to the RB flask, dissolving the solid components. The RB flask was then placed in an ice bath positioned on top of a stirring plate. Dicyclohexyl carbodiimide (4.85 mmol, 1.94 mmol) was added to the mixture via syringe dropwise over a period of 5 minutes. The ice bath was then removed, allowing the reaction to return to room temperature. The reaction was left to stir overnight. Following reaction, the mixture was concentrated through rotary

evaporation. Subsequent flash column chromatography (hexanes/EtOAc) allowed for isolation of the ester.[1-2]

Procedure for the Synthesis of N-(but-3-en-1-yl)benzenesulfonamide

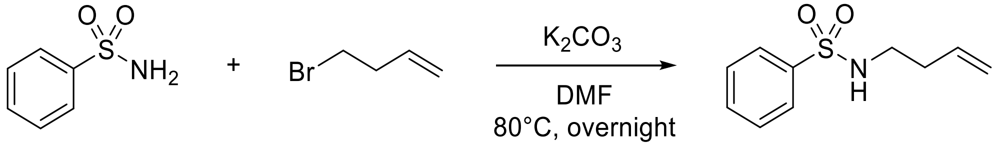

To an RB flask were added benzenesulfonamide (944 mg, 6.0 mmol), 4-bromobut-1-ene (0.8 mL, 6.0 mmol), dimethylformamide (30 mL), and a stir bar. Potassium carbonate (830 mg, 6.0 mmol) was added to the reaction mixture. After stirring overnight at 80 °C, the mixture was cooled to room temperature and quenched with water. The reaction mixture was then washed with brine and extracted with diethyl ether. The organic layer was concentrated through rotary evaporation. Subsequent flash column chromatography (hexanes/EtOAc) (3:1) allowed for isolation of *N*-(but-3-en-1-yl)benzenesulfonamide.[3]

Procedure for the Synthesis of N-(but-3-en-1-yl)-N-methylbenzenesulfonamide

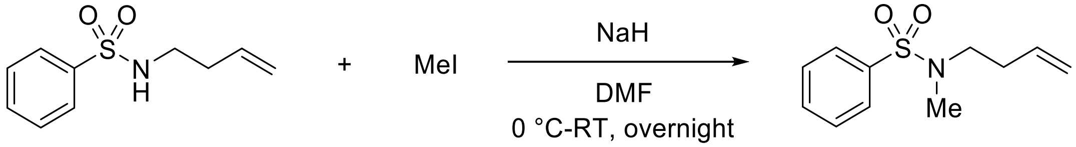

To an RB flask were added sodium hydride (60% in mineral oil, 240 mg, 6 mmol), dimethylformamide (25 mL), a solution of *N*-(but-3-en-1-yl)benzenesulfonamide (1.20 g, 5 mmol) in DMF (5 mL), and a stir bar in an ice bath at 0 °C. The reaction mixture was brought to room temperature and stirred for 30 minutes. The reaction mixture was cooled to 0 °C in an ice bath again, and a solution of methyl iodide (1.06 g, 7.5 mmol) in DMF (5 mL) was added dropwise over a period of 5 minutes by syringe. The reaction mixture was brought to room temperature and left to run overnight. The reaction mixture was quenched with a saturated aqueous solution of sodium bicarbonate. The mixture was then washed with brine and extracted with diethyl ether. The organic phase was dried over sodium sulfate and concentrated through rotary evaporation. Subsequent flash column chromatography (hexanes/EtOAc) (10:1) produced

N-(but-3-en-1-yl)-*N*-methylbenzenesulfonamide.[4]

Procedure for the Synthesis of 2-(but-3-en-1-yl)isoindoline-1,3-dione

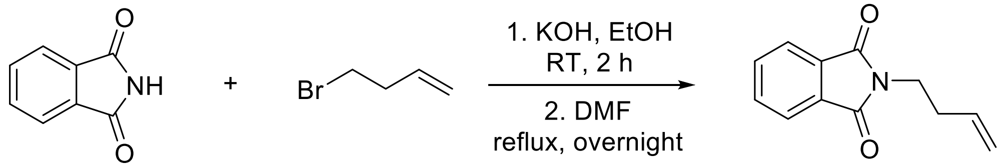

To an RB flask were added phthalimide (1.71 g, 11.6 mmol), potassium hydroxide (0.650 g, 11.6 mmol), ethyl alcohol (20 mL), and a stir bar. The reaction mixture was stirred at room temperature for 2 h and evaporated to remove EtOH. The resulting residue was then dissolved in dimethylformamide (15 mL) and 4-bromobut-1-ene (1.10 mL, 12.8 mmol) was added. The reaction mixture was stirred at reflux overnight. The reaction mixture was cooled, diluted with ethyl acetate, and quenched with saturated sodium bicarbonate. The mixture was then washed with brine. The extracted organic layer was dried over sodium sulfate and concentrated through rotary evaporation. Subsequent flash column chromatography (hexanes/EtOAc) (10:1) produced 2-(but-3-en-1-yl)isoindoline-1,3-dione.[5]

II. Optimization of Conditions (Photocatalytic Diazidation)

2.1 optimization of iron salts

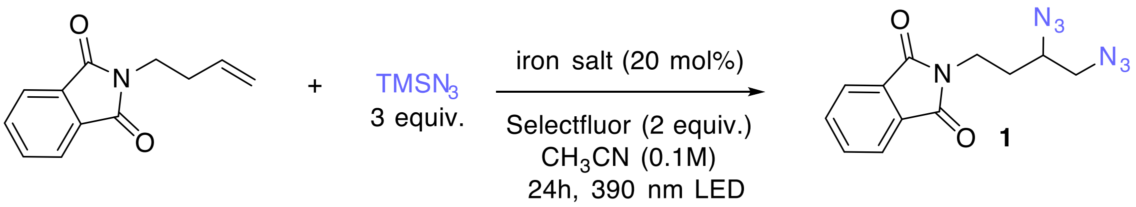

Entry	Cat.	yield (%)
1	$Fe(NO_3)_3{\cdot}9H_2O$	50
2	$Fe(OTf)_3$	36
3	$Fe(acac)_3$	48
4	$FeCl_3{\cdot}6H_2O$	24
5	$Fe(OAc)_2$	62
6	$FeCl_2$	48

2.2 optimization of the amount of oxidant

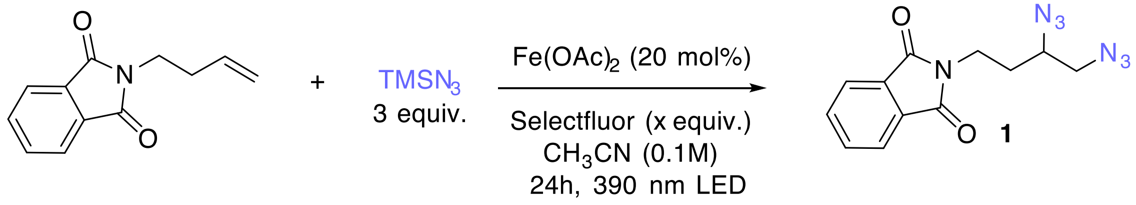

1

Entry	Selectfluor	yield (%)
1	1.0 equiv.	80
2	1.5 equiv.	76
3	2.0 equiv.	62
4	2.5 equiv.	56

2.3 optimization of iron catalyst loading

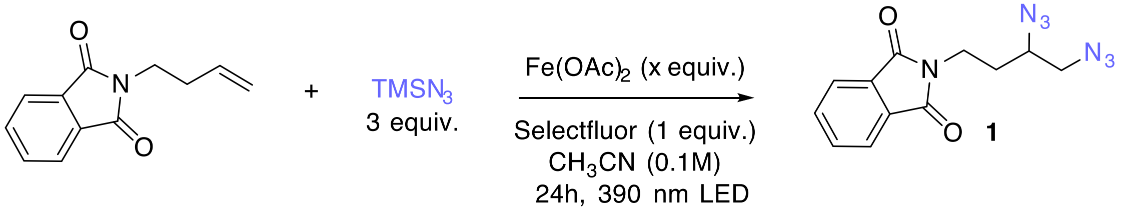

1

Entry	$Fe(OAc)_2$	yield (%)
1	2.5 mol%	72
2	5 mol%	76
3	10 mol%	88
4	20 mol%	80

2.4 optimization of wavelength

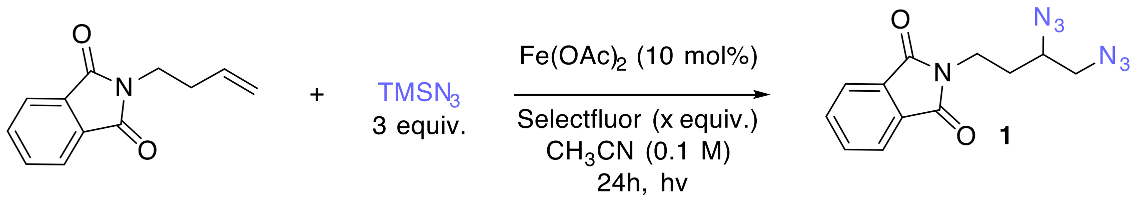

Entry	wavelength	yield (%)
1	390 nm	88
2	427 nm	72
3	456 nm	76

2.5 control experiments

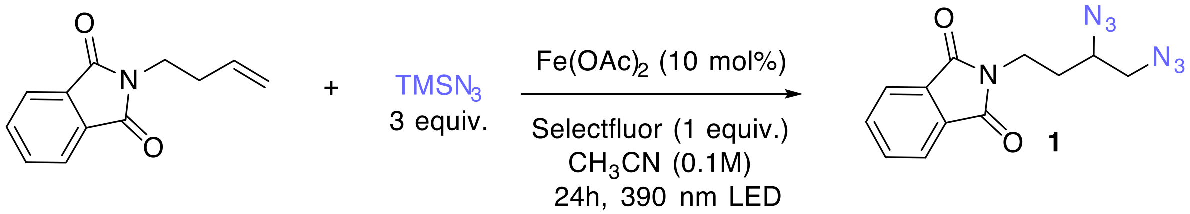

Entry	Deviation from the standard conditions	yield (%)
1	no iron salt	12
2	no light	ND
3	no Selectfluor	20

III. Optimization of Conditions (Photocatalytic Dichlorination)

3.1 optimization of iron salts

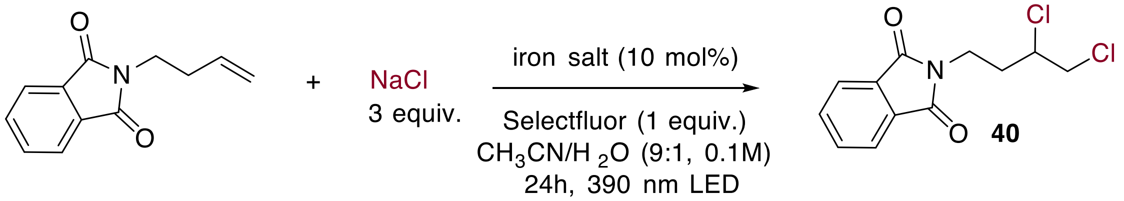

Entry	Cat. (20 mol%)	yield (%)
1	$Fe(NO_3)_3 \cdot 9H_2O$	86
2	$Fe(OTf)_3$	82
3	$Fe(acac)_3$	12
4	$FeCl_3$ $6H_2O$	82
5	$Fe(SO_4)_3$ $5H_2O$	80
6	$Fe(OAc)_2$	32
7	$FeCl_2$	72

3.2 control experiments

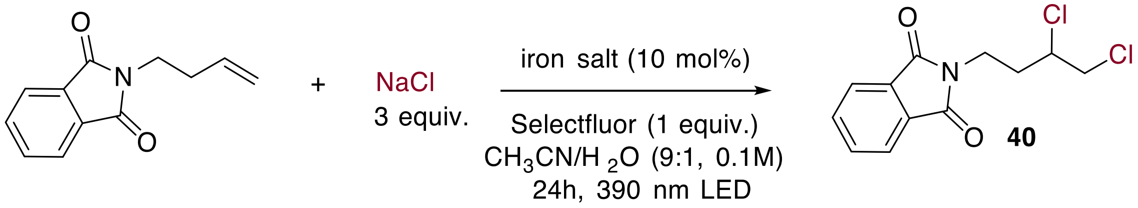

Entry	Deviation from the standard conditions	yield (%)
1	no iron salt	ND
2	no light	ND
3	no Selectfluor	trace
4	no water	trace

IV. Optimization of Conditions (Photocatalytic Fluorochlorination)

4.1 optimization of iron salts

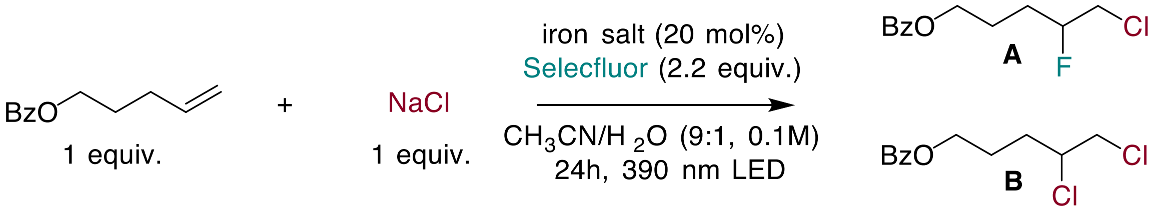

Entry	[Fe]	yield (A/B)
1	$FeCl_2$	8/4
2	$Fe(OAc)_2$	56/8
3	$Fe(NO_3)_3 \cdot 9H_2O$	trace/44
4	$Fe(OTf)_3$	8/36
5	$FeCl_3$ $6H_2O$ (5%)	trace/32
6	$FeCl_3$ $6H_2O$ (10%)	trace/52

4.2 optimization of catalyst loading

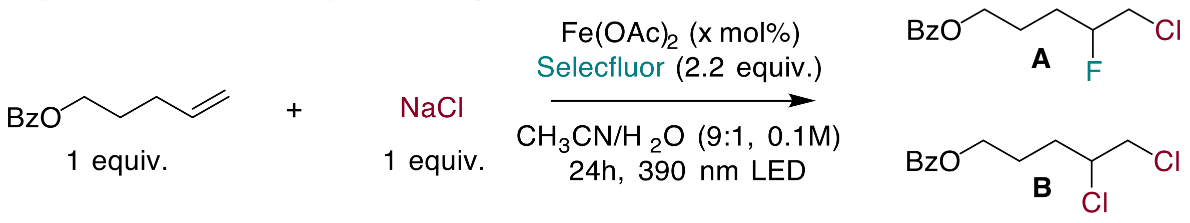

Entry	$Fe(OAc)_2$	yield (A/B)
1	10%	64/8
2	20%	56/8
3	30%	52/20

4.3 optimization of reactants loading (alkene)

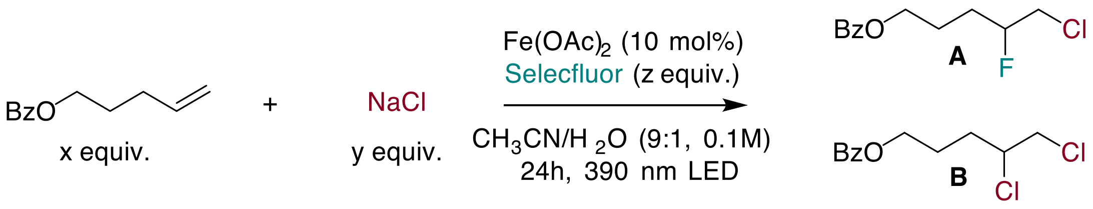

Entry	x	y	z	yield (A/B)
1	1	1	2.2	64/8
2	1.2	1	2.2	72/8
3	1.4	1	2.2	68/12
4	1.5	1	2.2	66/8
5	1.5	1	2.5	70/16

4.4 optimization of reactants loading (Selectfluor)

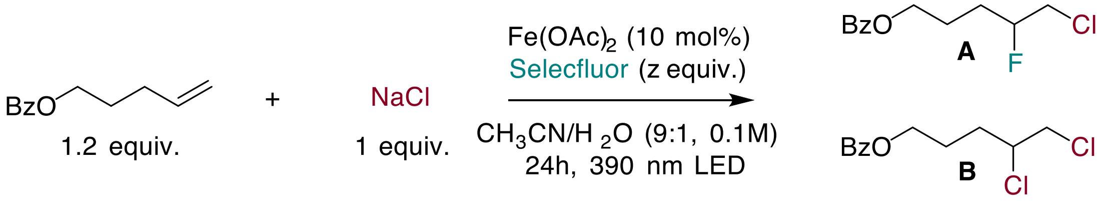

Entry	z	yield (A/B)
1	2	72/4
2	2.4	68/16
3	1.8	64/12

V. Experimental Methods of Difunctionalization of Alkenes

General Procedure A for diazidation of alkenes: $Fe(OAc)_2$ (0.01 mmol, 10 mol%) and Selectfluor (0.1 mmol, 1.0 equiv.) were added in an oven-dried 8-mL test vial containing a Teflon®-coated magnetic stir bar. The vial was evacuated and backfilled with N2 (repeated for 4 times), followed by addition of alkenes (0.1 mmol, 1.0 equiv.) and TMSN3 (0.3 mmol, 3.0 equiv) in MeCN (1.0 mL, 0.1 M in regard to alkenes) via syringe under N2. The reaction mixture was placed under 390nm Kessil® light (25% intensity) light after sealing the punctured holes of the vial cap with vacuum grease and electric tape/parafilm for better air-tight protection and allowed to react at room temperature for 24 h. Following this, the reaction mixture was filtered through a pad of celite which was subsequently rinsed with DCM. The filtrate was concentrated, and the residue was then purified by flash column chromatography to give the corresponding diazidated products.

General Procedure B for dichlorination of alkenes: Fe(NO3)3·9H2O (0.02 mmol, 20 mol%), sodium chloride (0.3 mmol, 3.0 equiv.) and Selectfluor (0.1 mmol, 1.0 equiv.) were added in an oven-dried 8-mL test vial containing a Teflon®-coated magnetic stir bar. The vial was evacuated and backfilled with N2 (repeated for 4 times), followed by addition of alkenes (0.1 mmol, 1.0

equiv.) in MeCN/H_2O (9:1, 1.0 mL, 0.1 M in regard to alkenes) via syringe under N2. The reaction mixture was placed under 390nm Kessil® light (25% intensity) light after sealing the punctured holes of the vial cap with vacuum grease and electric tape/parafilm for better air-tight protection and allowed to react at room temperature for 24 h. Following this, the reaction mixture was filtered through a pad of celite which was subsequently rinsed with DCM. The filtrate was concentrated, and the residue was then purified by flash column chromatography to give the corresponding dichlorinated products.

General Procedure C for fluorochlorination of alkenes: $Fe(OAc)_2$ (0.01 mmol, 8 mol%), sodium chloride (0.1 mmol, 0.8 equiv.) and Selectfluor (0.2 mmol, 1.7 equiv.) were added in an oven-dried 8-mL test vial containing a Teflon®-coated magnetic stir bar. The vial was evacuated and backfilled with N2 (repeated for 4 times), followed by addition of alkenes (0.12 mmol, 1.0 equiv.) in MeCN/H_2O (9:1, 1.0 mL, 0.1 M in regard to alkenes) via syringe under N2. The reaction mixture was placed under 390nm Kessil® light (25% intensity) light after sealing the punctured holes of the vial cap with vacuum grease and electric tape/parafilm for better air-tight protection and allowed to react at room temperature for 24 h. Following this, the reaction mixture was filtered through a pad of celite which was subsequently rinsed with DCM. The filtrate was concentrated, and the residue was then purified by flash column chromatography to give the corresponding fluorochlorinated products. Note that optimization of this reaction described in the tables above was done using NaCl as the limiting reagent.

VI. Preliminary Testing on Photocatalytic Fluoroazidation

Procedure for fluoroazidation of alkenes: $Fe(OAc)_2$ (0.01 mmol, 10 mol%) and Selectfluor (loading according to Table below) were added in an oven-dried 8-mL test vial containing a Teflon®-coated magnetic stir bar. The vial was evacuated and backfilled with N_2 (repeated for 4 times), followed by addition of alkenes (0.1 mmol, 1.0 equiv.) and $TMSN_3$ (0.1 mmol, 1.0 equiv) in MeCN (1.0 mL, 0.1 M in regard to alkenes) via syringe under N_2. The reaction mixture was placed under 390nm Kessil® light (25% intensity) after sealing the punctured holes of the vial cap with vacuum grease and electric tape/parafilm for better air-tight protection and allowed to react at room temperature for 24 h. Following this, the reaction mixture was filtered through a pad of celite which was subsequently rinsed with DCM. The filtrate was concentrated, and the residue was then purified by preparatory thin-layer chromatography (with eluent of Hex:EA = 5:1) to give the corresponding fluoroazidation products.

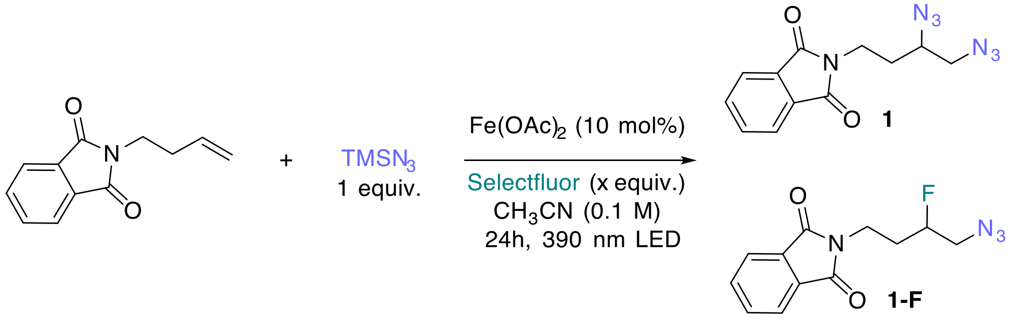

Entry	x equiv.	yield (1) (%)	yield (1-F) (%)
1[a]	2 equiv.	16	8
2[b]	1.5 equiv.	20	9
3[c]	2 equiv.	68	trace

[a] with 1 equiv. of alkene. [b] with 1.2equiv. of alkene. [c] with 3 equiv. of $TMSN_3$. ^1H NMR yield was determined by using CH_2Br_2 as an internal standard.

2-(4-azido-3-fluorobutyl)isoindoline-1,3-dione

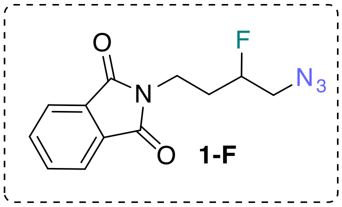

^{1}H NMR (600 MHz, $CDCl_3$) δ 7.79 (dd, J = 5.5, 3.0 Hz, 2H), 7.66 (dd, J = 5.5, 3.0 Hz, 2H), 4.74 – 4.58 (m, 1H), 3.83 – 3.77(m, 2H), 3.41 – 3.32 (m, 2H), 2.14 – 2.04 (m, 1H), 1.99 – 1.86 (m, 1H).
^{13}C NMR (151 MHz, $CDCl_3$) δ 168.22, 134.13, 132.00, 123.40, 90.61 (d, J = 174.3 Hz), 54.03 (d, J = 21.9 Hz), 34.13 (d, J = 4.7 Hz), 31.12 (d, J = 20.6 Hz).
^{19}F NMR (565 MHz, $CDCl_3$) δ -186.19 – -186.66 (m).
HRMS ESI: $[M-N_2+H]^+$ calcd. for $C_{12}H_{12}FN_4O_2$: 263.0939; Found 263.0936

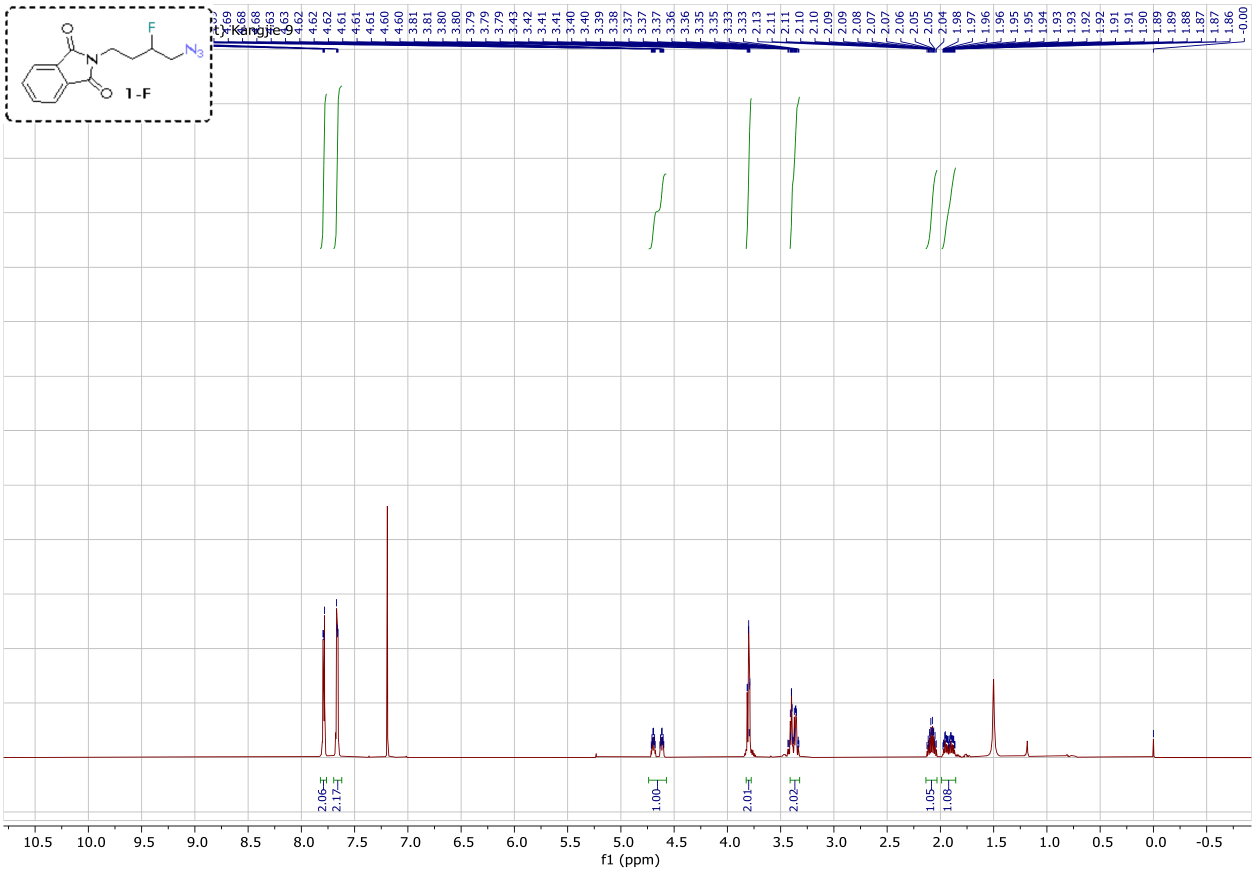

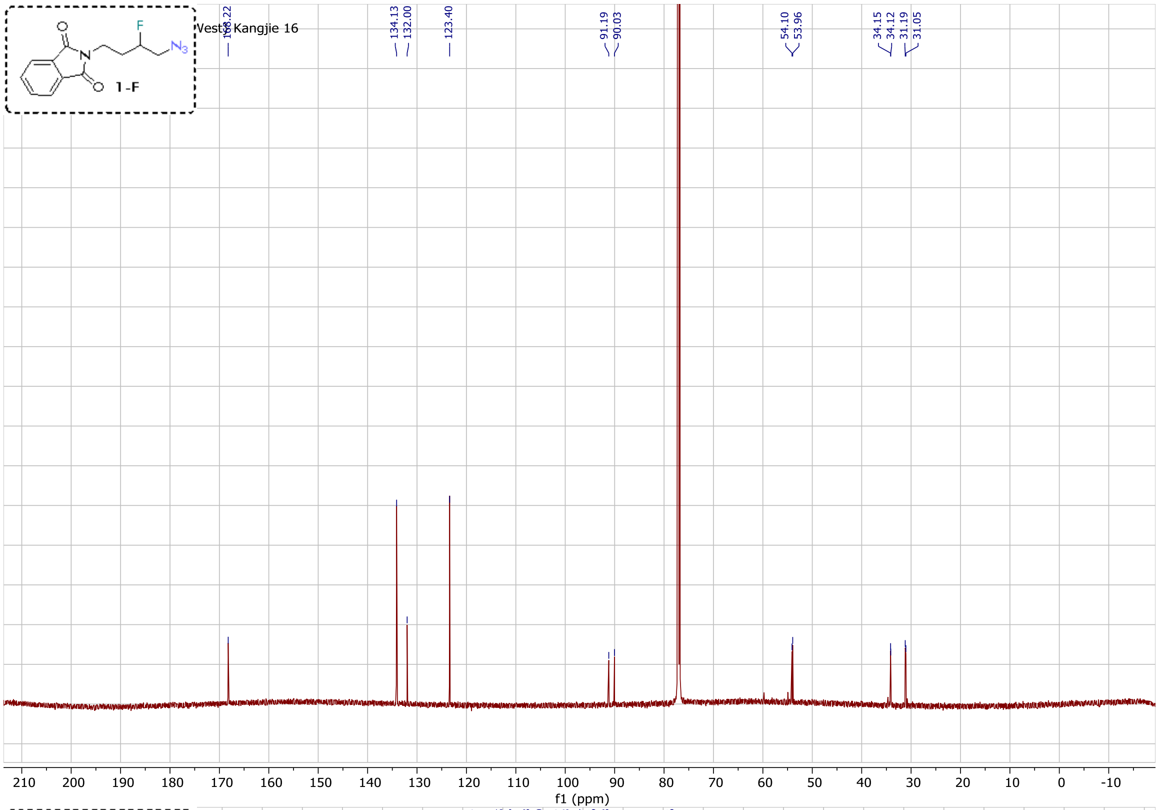
1-F
Kangjie 16
134.13
132.00
123.40
91.19
90.03
54.10
53.96
34.15
34.12
31.19
31.05
210 200 190 180 170 160 150 140 130 120 110 100 90 80 70 60 50 40 30 20 10 0 -10
f1 (ppm)

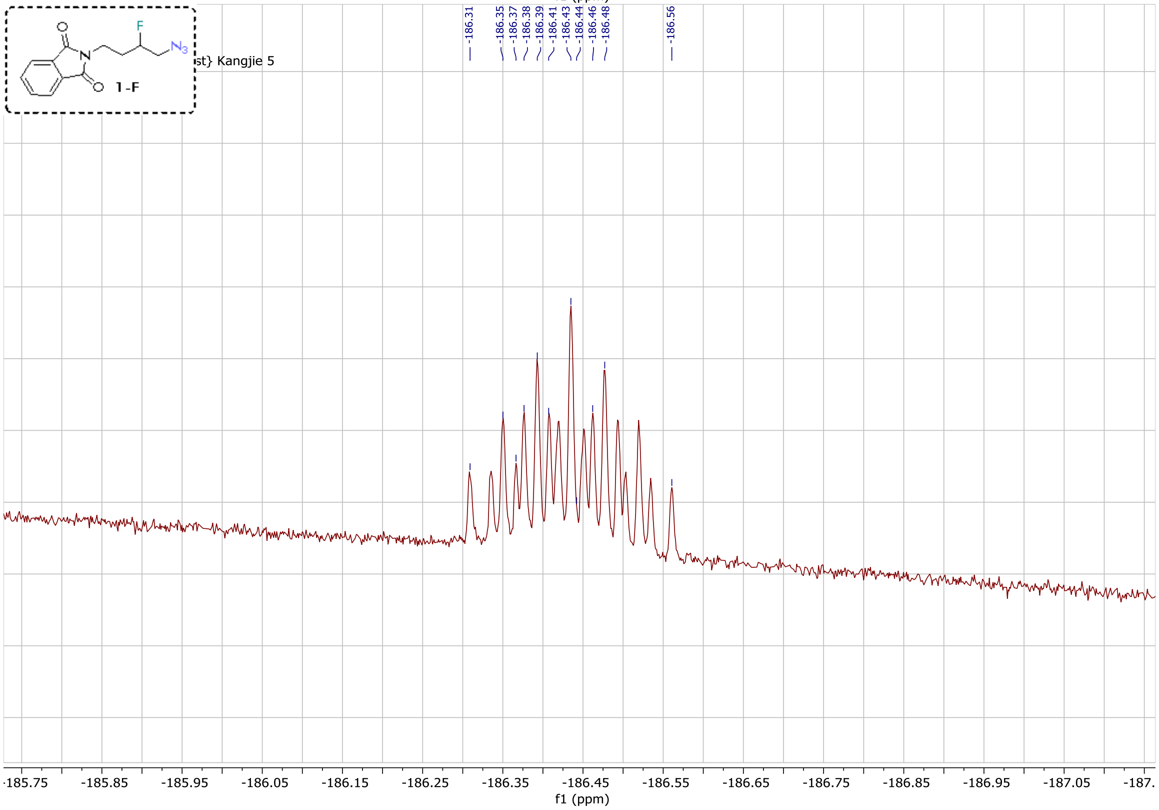
1-F
Kangjie 5
-186.31
-186.35
-186.37
-186.38
-186.39
-186.41
-186.43
-186.44
-186.46
-186.48
-186.56
-185.75 -185.85 -185.95 -186.05 -186.15 -186.25 -186.35 -186.45 -186.55 -186.65 -186.75 -186.85 -186.95 -187.05
f1 (ppm)

VII. Characterization of Corresponding Products

2-(3,4-diazidobutyl)isoindoline-1,3-dione

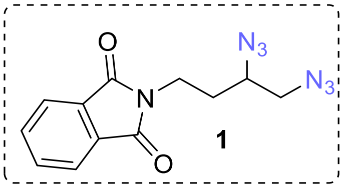

Prepared according to General Procedure A and obtained as white solid. **Yield** 86%, 24.5 mg. Purification is through preparatory thin-layer chromatography (with eluent of Hex: EA = 3:1) to give the corresponding diazidation products.
^{1}H NMR (600 MHz, CDCl$_3$) δ 7.87 (dd, J = 5.4, 3.1 Hz, 2H), 7.74 (dd, J = 5.4, 3.0 Hz, 2H), 3.84 (qt, J = 14.0, 6.7 Hz, 2H), 3.58 – 3.52 (m, 1H), 3.49 (dd, J = 12.7, 4.1 Hz, 1H), 3.42 (dd, J = 12.7, 7.3 Hz, 1H), 1.91 (dtd, J = 14.1, 7.0, 4.3 Hz, 1H), 1.82 (ddt, J = 14.3, 9.3, 6.4 Hz, 1H).
^{13}C NMR (151 MHz, CDCl$_3$) δ 168.27, 134.19, 131.95, 123.43, 59.75, 54.94, 34.68, 30.77.
The compound characterization was reported in literature.[6]

1,2-diazidododecane

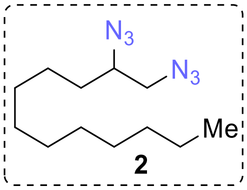

Prepared according to General Procedure A and obtained as colorless oil. **Yield** 87%, 21.9 mg. Purification is through preparatory column chromatography (with eluent of Hex: EA = 15:1) to give the corresponding diazidation products.
^{1}H NMR (600 MHz, CDCl$_3$) δ 3.49-3.43 (m, 1H), 3.38 (dd, J = 12.7, 4.0 Hz, 1H), 3.31 (dd, J = 12.7, 7.4 Hz, 1H), 1.58 – 1.53 (m, 2H), 1.48 – 1.40 (m, 1H), 1.37 – 1.26 (m, 15H), 0.88 (t, J = 6.9 Hz, 3H).
^{13}C NMR (151 MHz, CDCl$_3$) δ 62.09, 54.85, 31.90, 31.78, 29.56, 29.51, 29.41, 29.31, 29.30, 25.88, 22.69, 14.12.
HRMS APCI: $[M-N_2+H]^+$ calcd. for $C_{12}H_{25}N_4$: 225.2074; Found 225.2067

(3,4-diazidobutyl)benzene

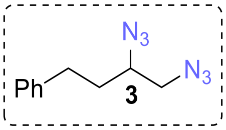

Prepared according to General Procedure A and obtained as colorless oil. **Yield** 85%, 18.4 mg. Purification is through preparatory thin-layer chromatography (with eluent of Hex: EA = 15:1) to give the corresponding diazidation products.
^{1}H NMR (600 MHz, CDCl$_3$) δ 7.35-7.29 (m, 2H), 7.24-7.15 (m, 3H), 3.49 – 3.38 (m, 2H), 3.35 (dd, J = 12.6, 7.3 Hz, 1H), 2.86-2.77 (m, 1H), 2.73-2.66 (m, 1H), 1.91 – 1.78 (m, 2H).
^{13}C NMR (151 MHz, CDCl$_3$) δ 140.35, 128.66, 128.39, 126.36, 61.11, 54.94, 33.39, 32.00.
The compound characterization was reported in literature.[6]

4,5-diazidopentyl benzoate

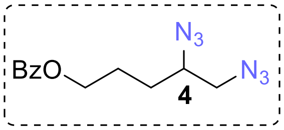

Prepared according to General Procedure A and obtained as colorless oil. **Yield** 84%, 23.0 mg. Purification is through preparatory thin-layer chromatography (with eluent of Hex: EA = 10:1) to give the corresponding diazidation products. **^{1}H NMR (600 MHz, CDCl$_3$)** δ 8.08 – 7.98 (m, 2H), 7.60 – 7.54 (m, 1H), 7.49 – 7.41 (m, 2H), 4.41 – 4.31 (m, 2H), 3.58-3.52 (m, 1H), 3.45 (dd, J = 12.7, 4.2 Hz, 1H), 3.38 (dd, J = 12.7, 7.3 Hz, 1H), 2.02 – 1.92 (m, 1H), 1.92-1.82 (m, 1H), 1.77 – 1.63 (m, 2H).

^{13}C NMR (151 MHz, CDCl$_3$) δ 166.54, 133.09, 130.08, 129.57, 128.44, 64.13, 61.61, 54.86, 28.55, 25.32.
The compound characterization was reported in literature.[6]

4,5-diazidopentyl 4-chlorobenzoate

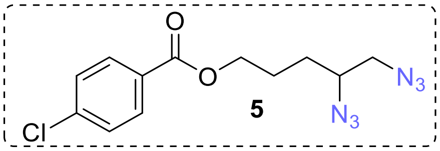

Prepared according to General Procedure A and obtained as colorless oil. **Yield** 72%, 22.2 mg. Purification is through preparatory thin-layer chromatography (with eluent of Hex: EA = 10:1) to give the corresponding diazidation products.
^{1}H NMR (600 MHz, CDCl$_3$) δ 7.97 (d, J = 8.6 Hz, 2H), 7.42 (d, J = 8.6 Hz, 2H), 4.42 – 4.30 (m, 2H), 3.58-3.52 (m, 1H), 3.45 (dd, J = 12.7, 4.3 Hz, 1H), 3.38 (dd, J = 12.7, 7.2 Hz, 1H), 2.02 – 1.92 (m, 1H), 1.91-1.81 (m, 1H), 1.76 – 1.62 (m, 2H).
^{13}C NMR (151 MHz, CDCl$_3$) δ 165.67, 139.55, 130.96, 128.79, 128.49, 64.38, 61.54, 54.82, 28.48, 25.27.
The compound characterization was reported in literature.[6]

4,5-diazidopentyl 4-(trifluoromethyl)benzoate

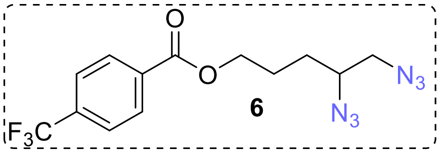

Prepared according to General Procedure A and obtained as colorless oil. **Yield** 76%, 26.0 mg. Purification is through preparatory thin-layer chromatography (with eluent of Hex: EA = 10:1) to give the corresponding diazidation products.
^{1}H NMR (600 MHz, CDCl$_3$) δ 8.15 (d, J = 8.1 Hz, 2H), 7.72 (d, J = 8.2 Hz, 2H), 4.51-4.33 (m, 2H), 3.61-3.52 (m, 1H), 3.46 (dd, J = 12.6, 4.3 Hz, 1H), 3.40 (dd, J = 12.7, 7.2 Hz, 1H), 2.03-1.94 (m, 1H), 1.95-1.85 (m, 1H), 1.78 – 1.63 (m, 2H).
^{13}C NMR (151 MHz, CDCl$_3$) δ 165.32, 134.55 (q, J = 32.7 Hz), 133.24, 129.99, 125.49 (q, J = 3.8 Hz), 123.60 (q, J = 272.5 Hz), 64.72, 61.53, 54.82, 28.45, 25.25.
^{19}F NMR (565 MHz, CDCl$_3$) δ -63.10.
The compound characterization was reported in literature.[6]

4,5-diazidopentyl 4-methylbenzenesulfonate

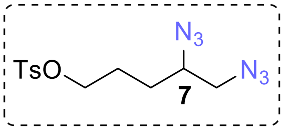

Prepared according to General Procedure A and obtained as colorless oil. **Yield** 86%, 27.9 mg. Purification is through preparatory thin-layer chromatography (with eluent of Hex: EA = 5:1) to give the corresponding diazidation products.
^{1}H NMR (600 MHz, CDCl$_3$) δ 7.79 (d, J = 8.2 Hz, 2H), 7.36 (d, J = 8.0 Hz, 2H), 3.45 – 3.36 (m, 2H), 3.31 (dd, J = 12.5, 7.1 Hz, 1H), 2.46 (s, 3H), 1.88-1.78 (m, 1H), 1.78-1.69 (m, 1H), 1.65 – 1.59 (m, 1H), 1.55-1.46 (m, 1H).
^{13}C NMR (151 MHz, CDCl$_3$) δ 145.04, 132.86, 129.95, 127.90, 69.54, 61.26, 54.81, 27.93, 25.40, 21.66.
The compound characterization was reported in literature.[6]

4,5-diazidopentyl (2,2,2-trichloroethyl) carbonate

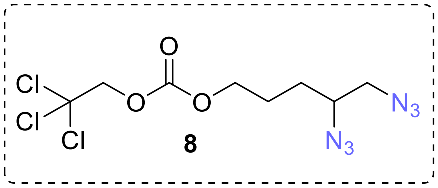

Prepared according to General Procedure A and obtained as colorless oil. **Yield** 78%, 27.0 mg. Purification is through preparatory thin-layer chromatography (with eluent of Hex: EA = 7:1) to give the corresponding diazidation products.
^{1}H NMR (600 MHz, $CDCl_3$) δ 4.78 (s, 2H), 4.33-4.22 (m, 2H), 3.56-3.48 (m, 1H), 3.44 (dd, J = 12.7, 4.2 Hz, 1H), 3.37 (dd, J = 12.7, 7.2 Hz, 1H), 1.97-1.88 (m, 1H), 1.88 – 1.78 (m, 1H), 1.73 – 1.65 (m, 1H), 1.65 – 1.58 (m, 1H).
^{13}C NMR (151 MHz, $CDCl_3$) δ 153.94, 94.38, 76.80, 68.33, 61.51, 54.85, 28.20, 25.14. The compound characterization was reported in literature.[6]

4,5-diazidopentyl furan-2-carboxylate

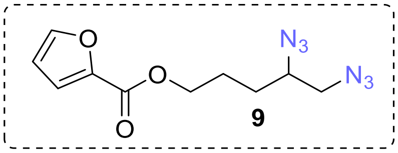

Prepared according to General Procedure A and obtained as colorless oil. **Yield** 42%, 11.1 mg. Purification is through preparatory thin-layer chromatography (with eluent of Hex: EA = 10:1) to give the corresponding diazidation products.
^{1}H NMR (600 MHz, $CDCl_3$) δ 7.59 (dd, J = 1.8, 0.9 Hz, 1H), 7.20 (dd, J = 3.5, 0.8 Hz, 1H), 6.53 (dd, J = 3.5, 1.7 Hz, 1H), 4.39-4.30 (m, 2H), 3.61 – 3.50 (m, 1H), 3.45 (dd, J = 12.7, 4.1 Hz, 1H), 3.37 (dd, J = 12.7, 7.3 Hz, 1H), 1.99 – 1.89 (m, 1H), 1.89-1.81 (m, 1H), 1.73 – 1.62 (m, 2H). [1]
^{3}C NMR (151 MHz, $CDCl_3$) δ 158.65, 146.46, 144.46, 118.14, 111.93, 64.11, 61.56, 54.86, 28.47, 25.24.
HRMS ESI: $[M-N_2+H]^+$ calcd. for $C_{10}H_{13}N_4O_3$: 237.0982; Found 237.0986

(tetrahydro-2*H*-pyran-4-yl)methyl 4,5-diazidopentanoate

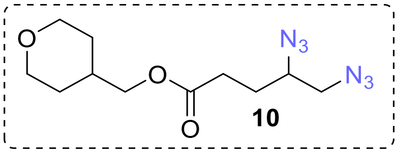

Prepared according to General Procedure A and obtained as colorless oil. **Yield** 62%, 17.5 mg. Purification is through preparatory column chromatography (with eluent of Hex: EA = 5:1) to give the corresponding diazidation products.
^{1}H NMR (600 MHz, $CDCl_3$) δ 4.03 – 3.94 (m, 4H), 3.62 – 3.54 (m, 1H), 3.46 (dd, J = 12.7, 4.2 Hz, 1H), 3.42 – 3.35 (m, 3H), 2.53 – 2.43 (m, 2H), 1.94 – 1.87 (m, 2H), 1.80-1.73 (m, 1H), 1.63-1.58 (m, 2H), 1.42-1.33 (m, 2H).
^{13}C NMR (151 MHz, $CDCl_3$) δ 172.48, 69.00, 67.44, 61.09, 54.86, 34.46, 30.25, 29.47, 26.98.
The compound characterization was reported in literature.[6]

4,5-diazidopentyl quinoline-2-carboxylate

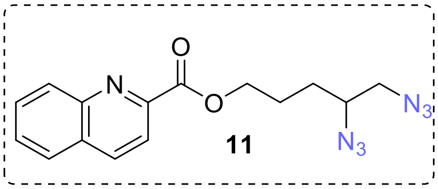

Prepared according to General Procedure A and obtained as colorless oil. **Yield** 48%, 15.6 mg. Purification is through preparatory thin-layer chromatography (with eluent of Hex: EA = 5:1) to give the corresponding diazidation products.
^{1}H NMR (600 MHz, $CDCl_3$) δ 9.45 (d, J = 2.1 Hz, 1H), 8.86 (d, J = 2.2 Hz, 1H), 8.18 (d, J = 8.5 Hz, 1H), 7.96 (dd, J = 8.2, 1.4 Hz, 1H), 7.88—7.82 (m, 1H), 7.70 – 7.57 (m, 1H), 4.52 – 4.39 (m, 2H), 3.61-3.55 (m, 1H), 3.48 (dd, J = 12.7, 4.3 Hz, 1H), 3.41 (dd, J = 12.7, 7.2 Hz, 1H), 2.11-2.00 (m, 1H), 1.99-1.88 (m, 1H), 1.82-1.74 (m, 1H), 1.74 – 1.69 (m, 1H).

^{13}C NMR (151 MHz, CDCl$_3$) δ 165.34, 149.94, 149.88, 138.89, 132.05, 129.50, 129.18, 127.59, 126.84, 122.89, 64.69, 61.56, 54.86, 28.49, 25.33.
HRMS APCI: $[M + H]^+$ calcd. for $C_{15}H_{16}N_7O_2$: 326.1360; Found 326.1348

***N*-(3,4-diazidobutyl)-*N*-methylbenzenesulfonamide**

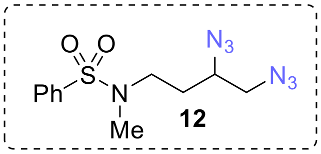

12

Prepared according to General Procedure A and obtained as colorless oil. **Yield** 70%, 21.7 mg. Purification is through preparatory thin-layer chromatography (with eluent of Hex: EA = 5:1) to give the corresponding diazidation products.
^{1}H NMR (600 MHz, CDCl$_3$) δ 7.85 – 7.76 (m, 2H), 7.66 – 7.60 (m, 1H), 7.60-7.52 (m, 2H), 3.75-3.67 (m, 1H), 3.53 (dd, *J* = 12.8, 4.0 Hz, 1H), 3.45 (dd, *J* = 12.8, 6.7 Hz, 1H), 3.26-3.18 (m, 1H), 3.05-2.96 (m, 1H), 2.76 (s, 3H), 1.86-1.77 (m, 1H), 1.71 – 1.64 (m, 1H).
^{13}C NMR (151 MHz, CDCl$_3$) δ 136.84, 132.88, 129.22, 129.20, 127.38, 58.93, 54.66, 46.99, 35.36, 29.85.
The compound characterization was reported in literature.[6]

***N*-(3,4-diazidobutyl)benzenesulfonamide**

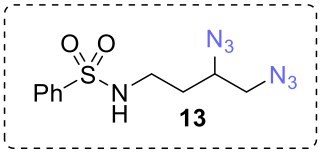

13

Prepared according to General Procedure A and obtained as colorless oil. **Yield** 69%, 20.4 mg. Purification is through preparatory thin-layer chromatography (with eluent of Hex: EA = 3:1) to give the corresponding diazidation products.
^{1}H NMR (600 MHz, CDCl$_3$) δ 7.94 – 7.82 (m, 2H), 7.65 – 7.59 (m, 1H), 7.58-7.52 (m, 2H), 4.95 (t, *J* = 6.3 Hz, 1H), 3.67-3.61 (m, 1H), 3.44 (dd, *J* = 12.7, 4.1 Hz, 1H), 3.35 (dd, *J* = 12.7, 7.1 Hz, 1H), 3.14 – 3.03 (m, 2H), 1.79-1.69 (m, 1H), 1.64-1.56 (m, 1H). [1]
^{3}C NMR (151 MHz, CDCl$_3$) δ 139.42, 132.98, 129.31, 127.02, 59.05, 54.75, 39.88, 31.68.
The compound characterization was reported in literature.[6]

1,2-diazido-6-bromohexane

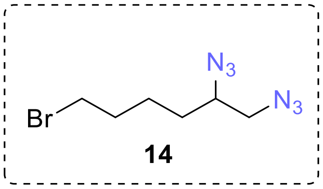

14

Prepared according to General Procedure A and obtained as colorless oil. **Yield** 89%, 22.0 mg. Purification is through preparatory column chromatography (with eluent of Hex: EA = 10:1) to give the corresponding diazidation products.
^{1}H NMR (600 MHz, CDCl$_3$) δ 3.51 – 3.45 (m, 1H), 3.45 – 3.40 (m, 3H), 3.35 (dd, *J* = 12.7, 7.3 Hz, 1H), 1.96 – 1.81 (m, 2H), 1.66 – 1.60 (m, 1H), 1.58 – 1.51 (m, 3H).
^{13}C NMR (151 MHz, CDCl$_3$) δ 61.79, 54.76, 33.16, 32.20, 30.91, 24.52.
The compound characterization was reported in literature.[6]

2-(2,3-diazidopropyl)cyclohexan-1-one

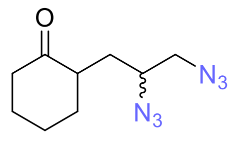

15 1.1:1 d.r.

Prepared according to General Procedure A and obtained as colorless oil. **Yield** 68%, 15.1 mg. Purification is through preparatory column chromatography (with eluent of Hex: EA = 5:1) to give the corresponding 1.1:1 mixture of diastereomeric diazidation products.

^{1}H NMR (600 MHz, CDCl$_3$) δ 3.74-3.64 (m, 0.50H), 3.63-3.55 (m, 0.45H), 3.50 (dd, J = 12.7, 3.8 Hz, 1H), 3.44 (dd, J = 12.7, 3.9 Hz, 0.48H), 3.39-3.30 (m, 1H), 2.61-2.54 (m, 0.54H), 2.52-2.46 (m, 0.48H), 2.45 – 2.31 (m, 2H), 2.24-2.16 (m, 0.47H), 2.16 – 2.01 (m, 2H), 1.98 – 1.85 (m, 1.55H), 1.78 – 1.64 (m, 2H), 1.47 – 1.33 (m, 1.54H), 1.21 – 1.16 (m, 0.52H).

^{13}C NMR (151 MHz, CDCl$_3$) δ 212.58, 211.93, 60.56, 59.18, 55.55, 55.15, 47.17, 46.92, 42.39, 42.10, 35.50, 33.67, 32.44, 31.26, 28.22, 27.85, 25.38, 25.09.

HRMS APCI: $[M-N_2+H]^+$ calcd. for $C_9H_{15}N_3O$: 195.1240; Found 195.1250

4,5-diazidopentan-1-ol

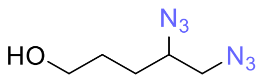

16

Prepared according to General Procedure A and obtained as colorless oil. **Yield** 53%, 9.0 mg. Purification is through preparatory column chromatography (with eluent of Hex: EA = 5:1) to give the corresponding diazidation products.

^{1}H NMR (600 MHz, CDCl$_3$) δ 3.75-3.66 (m, 2H), 3.57 – 3.51 (m, 1H), 3.43 (dd, J = 12.7, 4.0 Hz, 1H), 3.36 (dd, J = 12.7, 7.4 Hz, 1H), 1.76 – 1.62 (m, 4H).

^{13}C NMR (151 MHz, CDCl$_3$) δ 62.16, 61.86, 54.89, 28.80, 28.31.

The compound characterization was reported in literature.[6]

6,7-diazidoheptanoic acid

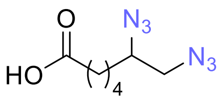

17

Prepared according to General Procedure A and obtained as colorless oil. **Yield** 52%, 11.0 mg. Purification is through preparatory column chromatography (with eluent of Hex: EA: AcOH = 2:1:0.1) to give the corresponding diazidation products.

^{1}H NMR (600 MHz, CDCl$_3$) δ 3.52-3.44 (m, 1H), 3.40 (dd, J = 12.7, 4.1 Hz, 1H), 3.33 (dd, J = 12.7, 7.3 Hz, 1H), 2.40 (t, J = 7.3 Hz, 2H), 1.72 – 1.63 (m, 2H), 1.60-1.49 (m, 3H), 1.45 (qt, J = 9.6, 2.6 Hz, 1H).

^{13}C NMR (151 MHz, CDCl$_3$) δ 179.04, 61.78, 54.80, 33.60, 31.45, 25.33, 24.24.

The compound characterization was reported in literature.[6]

(2,3-diazido-2-methylpropyl)benzene

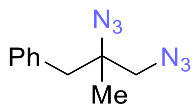

18

Prepared according to General Procedure A and obtained as colorless oil. **Yield** 76%, 16.4 mg. Purification is through preparatory thin-layer chromatography (with eluent of Hex: EA = 15:1) to give the corresponding diazidation products.

^{1}H NMR (600 MHz, CDCl$_3$) δ 7.35 – 7.26 (m, 3H), 7.24 – 7.19 (m, 2H), 3.23 (s, 2H), 2.87 (d, *J* = 13.6 Hz, 1H), 2.82 (d, *J* = 13.6 Hz, 1H), 1.29 (s, 3H).
^{13}C NMR (151 MHz, CDCl$_3$) δ 135.32, 130.47, 128.40, 127.15, 63.94, 58.35, 43.20, 21.09.
The compound characterization was reported in literature.[6]

(2,3-diazido-2-methylpropoxy)benzene

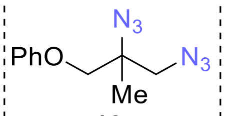

19

Prepared according to General Procedure A and obtained as colorless oil. **Yield** 86%, 19.9 mg. Purification is through preparatory thin-layer chromatography (with eluent of Hex: EA = 10:1) to give the corresponding diazidation products.
^{1}H NMR (600 MHz, CDCl$_3$) δ 7.33 – 7.27 (m, 2H), 7.02-6.96 (m, 1H), 6.95-6.88 (m, 2H), 4.00 – 3.93 (m, 2H), 3.51 (d, *J* = 12.5 Hz, 1H), 3.47 (d, *J* = 12.6 Hz, 1H), 1.45 (s, 3H).
^{13}C NMR (151 MHz, CDCl$_3$) δ 158.07, 129.60, 121.62, 114.60, 71.29, 62.86, 56.24, 19.26.
The compound characterization was reported in literature.[6]

4,5-diazidooctane

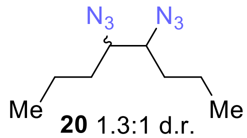

20 1.3:1 d.r.

Prepared according to General Procedure A and obtained as colorless oil in the mixture of two isomers. **Yield** 63%, 12.4 mg. d.r. 1.3:1. Purification is through preparatory column chromatography (with eluent of Hex: EA = 15:1) to give the corresponding diazidation products.
^{1}H NMR (600 MHz, CDCl$_3$) δ 3.37 – 3.33 (m, 0.85H), 3.30 – 3.25 (m, 1.15H), 1.71-1.63 (m, 1H), 1.62 – 1.57 (m, 2H), 1.56 – 1.48 (m, 3H), 1.46 – 1.38 (m, 2H), 1.00-0.95 (m, 6H).
^{13}C NMR (151 MHz, CDCl$_3$) δ 65.62, 65.02, 33.36, 32.40, 19.60, 19.48, 13.82.
The compound characterization was reported in literature.[6]

2,3-diazidobutane-1,4-diyl dibenzoate

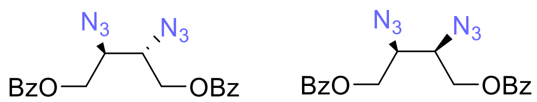

21 **21'**

anti (21): syn (21') = 3.3:1

Prepared according to General Procedure A and obtained as white solid in the mixture of two isomers. **Yield** 40%, 15.2 mg. d.r. = 3.3:1 (anti:syn).[7] Purification is through preparatory thin-layer chromatography (with eluent of Hex: EA = 8:1) to give the corresponding diazidation products.
^{1}H NMR (600 MHz, CDCl$_3$) δ 8.11-8.03 (m, 4H), 7.65-7.56 (m, 2H), 7.52-7.43 (m, 4H), 4.77 (dd, *J* = 11.7, 2.7 Hz, 0.47H), 4.67 (dd, *J* = 11.6, 4.0 Hz, 1.53H), 4.57 (dd, *J* = 11.6, 7.2 Hz, 1.55H), 4.54 – 4.49 (m, 0.45H), 4.04 – 3.96 (m, 1.51H), 3.95 – 3.90 (m, 0.44H).
^{13}C NMR (151 MHz, CDCl$_3$) δ 165.98, 165.96, 133.61, 133.57, 129.81, 129.09, 129.00, 128.61, 64.30, 64.28, 60.46, 60.32.
HRMS APCI: $[M-N2+H]^+$ calcd. for $C_{18}H_{17}N_4O_4$: 353.1244; Found 353.1236

3,4-diazidohexyl benzoate

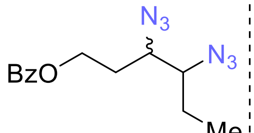

22 1.1:1 d.r. Prepared according to General Procedure A and obtained as colorless oil in a mixture of two isomers. **Yield** 68%, 19.6 mg. d.r. 1.1:1. Purification is through preparatory thin-layer chromatography (with eluent of Hex: EA = 10:1) to give the corresponding diazidation products.

^{1}H NMR (600 MHz, CDCl$_3$) δ 8.00-7.99 (m, 2H), 7.63 – 7.55 (m, 1H), 7.50 – 7.41 (m, 2H), 4.56 – 4.51 (m, 1H), 4.48 – 4.41 (m, 1H), 3.64-3.58 (m, 0.58H), 3.58-3.53 (m, 0.42H), 3.44-3.38 (m, 0.53H), 3.33-3.28 (m, 0.47H), 2.16 – 2.02 (m, 1.56H), 1.96-1.89 (m, 0.54H), 1.79-1.72 (m, 1H), 1.71 – 1.62 (m, 1H), 1.07 (q, J = 7.2 Hz, 3H).

^{13}C NMR (151 MHz, CDCl$_3$) δ 166.36, 133.22, 133.19, 129.83, 129.79, 129.58, 128.49, 128.47, 67.29, 66.85, 62.42, 62.01, 61.51, 61.44, 30.65, 29.45, 24.39, 23.90, 10.79, 10.63.

The compound characterization was reported in literature.[6]

4-(4-(1,2-diazidopropyl)cyclohexyl)benzonitrile

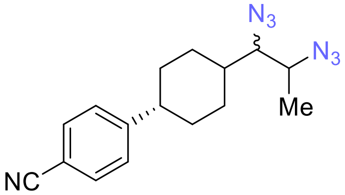

23 1.5:1 d.r. Prepared according to General Procedure A and obtained as colorless oil in a mixture of two isomers. **Yield** 58%, 17.9 mg. d.r. 1.5:1. Purification is through preparatory thin-layer chromatography (with eluent of Hex: EA = 5:1) to give the corresponding diazidation products.

^{1}H NMR (600 MHz, CDCl$_3$) δ 7.61 – 7.56 (m, 2H), 7.33 – 7.28 (m, 2H), 3.70 (qd, J = 6.6, 5.0 Hz, 0.40H), 3.64 (p, J = 6.5 Hz, 0.60H), 3.17 (t, J = 6.2 Hz, 0.59H), 2.95 (dd, J = 6.8, 5.0 Hz, 0.39H), 2.59-2.52 (m, 1H), 2.03 – 1.94 (m, 3H), 1.86 – 1.61 (m, 2H), 1.51 – 1.30 (m, 7H).

^{13}C NMR (151 MHz, CDCl$_3$) δ 152.25, 132.32, 127.62, 119.05, 109.99, 72.13, 72.02, 58.06, 57.63, 44.17, 38.89, 38.65, 33.27, 33.17, 33.06, 33.00, 30.10, 30.08, 28.40, 28.23, 16.83, 14.64.

The compound characterization was reported in literature.[6]

2,3-diazido-3-methylbutyl benzoate

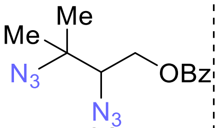

24 Prepared according to General Procedure A and obtained as colorless oil. **Yield** 71%, 19.5 mg. Purification is through preparatory thin-layer chromatography (with eluent of Hex: EA = 10:1) to give the corresponding diazidation products.

^{1}H NMR (600 MHz, CDCl$_3$) δ 8.10 – 8.05 (m, 2H), 7.62 – 7.58 (m, 1H), 7.50-7.43 (m, 2H), 4.75 (dd, J = 11.5, 2.8 Hz, 1H), 4.30 (dd, J = 11.5, 9.5 Hz, 1H), 3.69 (dd, J = 9.4, 2.8 Hz, 1H), 1.42 (s, 3H), 1.39 (s, 3H).

^{13}C NMR (151 MHz, CDCl$_3$) δ 166.27, 133.46, 129.81, 129.33, 128.58, 68.32, 64.65, 62.11, 23.84, 22.96.

The compound characterization was reported in literature.[6]

1,2-diazidocyclooctane

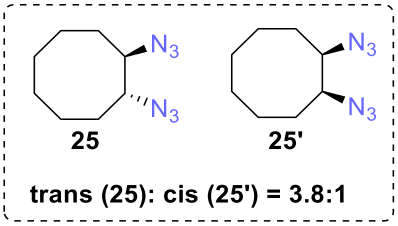

Prepared according to General Procedure A and obtained as colorless oil in the mixture of two isomers. **Yield** 64%, 12.4 mg. d.r. = 3.8:1 (trans:cis).[8] Purification is through preparatory column chromatography (with eluent of Hex: EA = 15:1) to give the corresponding diazidation products.

^{1}H NMR (600 MHz, CDCl$_3$) δ 3.81 – 3.70 (m, 0.41H), 3.51 (dd, J = 4.9, 2.2 Hz, 1.60H), 1.97-1.89 (m, 2H), 1.84 – 1.71 (m, 4H), 1.70 – 1.62 (m, 2H), 1.60 – 1.54 (m, 2H), 1.48-1.31 (m, 2H).

^{13}C NMR (151 MHz, CDCl$_3$) δ 66.57, 63.37, 29.24, 28.10, 26.43, 25.55, 24.74, 23.46.

The compound characterization was reported in literature.[6]

2,3-diazidobicyclo[2.2.1]heptane, (1*R*,2*R*,3*R*,4*S*)-2,3-diazidobicyclo[2.2.1]heptane

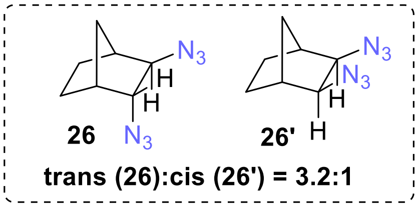

Prepared according to General Procedure A and obtained as colorless oil in the mixture of two isomers. **Yield** 65%, 11.5 mg. d.r. = 3.2:1 (trans:cis).[9] Purification is through preparatory column chromatography (with eluent of Hex: EA = 15:1) to give the corresponding diazidation products.

^{1}H NMR (600 MHz, CDCl$_3$) δ 3.66 (dt, J = 4.5, 2.2 Hz, 0.26H), 3.60 (d, J = 1.7 Hz, 1.74H), 3.19 (t, J = 2.6 Hz, 0.25H), 2.52 – 2.41 (m, 0.31H), 2.36 (dq, J = 3.3, 1.6 Hz, 1.74H), 2.33 (d, J = 4.3 Hz, 0.28H), 1.79-1.73 (m, 1H), 1.69 – 1.62 (m, 1H), 1.62-1.59 (m, 1H), 1.43 – 1.36 (m, 0.57H), 1.24-1.21 (m, 1H), 1.18-16 (m, 1H).

^{13}C NMR (151 MHz, CDCl$_3$) δ 70.44, 69.99, 67.47, 41.96, 41.61, 41.54, 40.51, 35.01, 33.37, 26.27, 25.89, 25.78, 20.86.

The compound characterization was reported in literature.[6]

(5*R*)-5-(1,2-diazidopropan-2-yl)-2-methylcyclohex-2-en-1-one

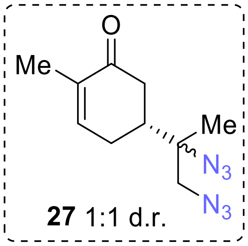

Prepared according to General Procedure A and obtained as colorless oil in the mixture of two isomers. **Yield** 54%, 12.7 mg. d.r. 1:1. Purification is through preparatory thin-layer chromatography (with eluent of Hex: EA = 3:1) to give the corresponding diazidation products.

^{1}H NMR (600 MHz, CDCl$_3$) δ 6.82-6.65 (m, 1H), 3.47 – 3.33 (m, 2H), 2.55 – 2.46 (m, 1H), 2.42 – 2.22 (m, 4H), 1.78 (s, 3H), 1.37 (d, J = 5.4 Hz, 3H).

^{13}C NMR (151 MHz, CDCl$_3$) δ 198.52, 198.39, 144.06, 143.64, 135.64, 135.50, 64.73, 64.67, 57.60, 57.48, 41.24, 41.21, 39.04, 38.81, 26.97, 26.67, 18.64, 18.48, 15.60, 15.59.

The compound characterization was reported in literature.[6]

(1,2-diazidoethyl)benzene

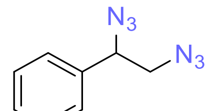

28

Prepared according to General Procedure A and obtained as colorless oil. **Yield** 86%, 16.2 mg. Purification is through preparatory thin-layer chromatography (with eluent of Hex: EA = 15:1) to give the corresponding diazidation products.

^{1}H NMR (600 MHz, $CDCl_3$) δ 7.45 – 7.36 (m, 3H), 7.36 – 7.31 (m, 2H), 4.67 (dd, J = 8.5, 4.8 Hz, 1H), 3.50 (dd, J = 12.8, 8.4 Hz, 1H), 3.44 (dd, J = 12.8, 4.8 Hz, 1H).

^{13}C NMR (151 MHz, $CDCl_3$) δ 136.30, 129.10, 129.07, 126.95, 65.52, 55.94.

The compound characterization was reported in literature.[6]

1,2-diazido-1,2-diphenylethane

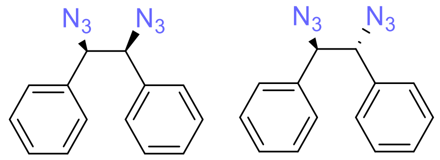

29 29'

syn (29):anti (29') = 2:1

Prepared according to General Procedure A and obtained as colorless oil in the mixture of two isomers. **Yield** 74%, 19.6 mg. d.r. = 2:1 (syn:anti).[10] Purification is through preparatory thin-layer chromatography (with eluent of Hex: EA = 15:1) to give the corresponding diazidation products.

^{1}H NMR (600 MHz, $CDCl_3$) δ 7.41-7.35 (m, 2H), 7.30-7.25 (m, 2H), 7.24-7.21 (s, 3H), 7.12 – 7.01 (m, 3H), 4.69 (s, 0.65H), 4.64 (s, 1.35H).

^{13}C NMR (151 MHz, $CDCl_3$) δ 135.85, 135.76, 129.01, 128.74, 128.70, 128.60, 127.97, 127.68, 70.74, 69.66.

The compound characterization was reported in literature.[6]

1,2-diazido-2,3-dihydro-1*H*-indene

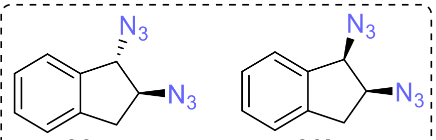

30 30'

trans (30):cis (30') = 3:1

Prepared according to General Procedure A and obtained as colorless oil in a mixture of two isomers. **Yield** 50%, 10.0 mg. d.r. = 3:1 (trans:cis).[10] Purification is through preparatory thin-layer chromatography (with eluent of Hex: EA = 15:1) to give the corresponding diazidation products.

^{1}H NMR (trans) (600 MHz, $CDCl_3$) δ 7.40 – 7.26 (m, 4H), 4.77 (d, J = 5.6 Hz, 1H), 4.17 (td, J = 7.0, 5.6 Hz, 1H), 3.35 (dd, J = 16.0, 7.3 Hz, 1H), 2.94 (dd, J = 15.9, 6.6 Hz, 1H).

^{13}C NMR (trans) (151 MHz, $CDCl_3$) δ 139.09, 137.73, 129.48, 127.77, 125.15, 124.57, 70.25, 67.66, 36.12.

^{1}H NMR (cis) (600 MHz, $CDCl_3$) δ 7.42 – 7.29 (m, 4H), 4.84 (d, J = 5.6 Hz, 1H), 4.30 (td, J = 6.6, 5.6 Hz, 1H), 3.23 – 3.12 (m, 2H).

^{13}C NMR (cis) (151 MHz, $CDCl_3$) δ 139.72, 137.49, 129.75, 127.73, 125.41, 124.94, 66.94, 64.09, 35.57.

The compound characterization was reported in literature.[6]

2,3-diazido-3-phenylpropyl benzoate

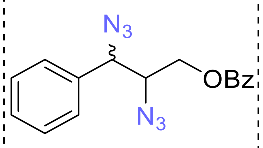

31 1.4:1 d.r.

Prepared according to General Procedure A and obtained as colorless oil in the mixture of two isomers. **Yield** 75%, 24.2 mg. d.r. 1.4:1. Purification is through preparatory thin-layer chromatography (with eluent of Hex: EA = 10:1) to give the corresponding diazidation products.
^{1}H NMR (600 MHz, CDCl$_3$) δ 8.08-8.00 (m, 2H), 7.62 – 7.56 (m, 1H), 7.50 – 7.39 (m, 6H), 7.39 – 7.34 (m, 1H), 4.73-4.66 (m, 1H), 4.61-4.56 (m, 0.57H), 4.41 – 4.33 (m, 1H), 4.13-4.09 (m, 0.46H), 4.04-3.97 (m, 0.59H)., 3.96 – 3.90 (m, 0.41H).
^{13}C NMR (151 MHz, CDCl$_3$) δ 166.02, 165.90, 135.30, 135.12, 133.47, 133.42, 129.77, 129.74, 129.45, 129.33, 129.22, 129.14, 128.56, 128.54, 127.75, 127.42, 66.75, 65.75, 64.62, 64.30, 64.12, 64.02.
The compound characterization was reported in literature.[6]

methyl 2,3-diazido-3-phenylpropanoate

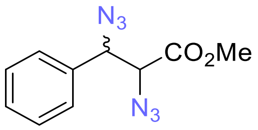

32 1.4:1 d.r.

Prepared according to General Procedure A and obtained as colorless oil in a mixture of two isomers. **Yield** (trans+cis) 68%, 16.7 mg. d.r. 1.4:1. Purification is through preparatory thin-layer chromatography (with eluent of Hex: EA =10:1) to give the corresponding diazidation products.
^{1}H NMR (trans) (600 MHz, CDCl$_3$) δ 7.48 – 7.36 (m, 5H), 4.90 (d, J = 8.0 Hz, 1H), 4.10 (d, J = 8.1 Hz, 1H), 3.83 (s, 3H).
^{13}C NMR (trans) (151 MHz, CDCl$_3$) δ 168.17, 134.35, 129.45, 129.00, 127.75, 65.45, 65.37, 52.96.
^{1}H NMR (cis) (600 MHz, CDCl$_3$) δ 7.48 – 7.34 (m, 5H), 5.06 (d, J = 5.8 Hz, 1H), 4.02 (d, J = 5.7 Hz, 1H), 3.75 (s, 3H).
^{13}C NMR (cis) (151 MHz, CDCl$_3$) δ 168.05, 134.89, 129.32, 129.04, 127.43, 66.31, 66.26, 53.04.
The compound characterization was reported in literature.[6]

(3,4-dichlorobutyl)benzene

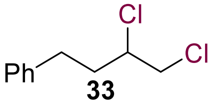

33

Prepared according to General Procedure B and obtained as colorless oil. **Yield** 81%, 16.3 mg. Purification is through preparatory thin-layer chromatography (with eluent of Hexane) to give the corresponding dichlorination products.
^{1}H NMR (600 MHz, CDCl$_3$) δ 7.34 – 7.28 (m, 2H), 7.24 – 7.18 (m, 3H), 4.03-3.96 (m, 1H), 3.78 (dd, J = 11.3, 5.1 Hz, 1H), 3.66 (dd, J = 11.4, 7.4 Hz, 1H), 2.96-2.87 (m, 1H), 2.81-2.72 (m, 1H), 2.35-2.27 (m, 1H), 2.08-1.97 (m, 1H).
^{13}C NMR (151 MHz, CDCl$_3$) δ 140.35, 128.57, 128.51, 126.28, 60.20, 48.22, 36.67, 31.99.
HRMS APCI: $[M+H]^+$ calcd. for $C_{18}H_{28}N_7O_4S$: 438.1918; Found 438.1908

4,5-dichloropentyl benzoate

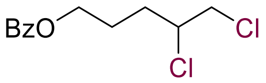

34

Prepared according to General Procedure B and obtained as colorless oil. **Yield** 86%, 22.5 mg. Purification is through preparatory thin-layer chromatography (with eluent of Hex: EA = 10:1) to give the corresponding dichlorination products.

^{1}H NMR (600 MHz, $CDCl_3$) δ 8.10 – 8.02 (m, 2H), 7.61 – 7.54 (m, 1H), 7.49-7.42 (m, 2H), 4.43 – 4.34 (m, 2H), 4.16 – 4.09 (m, 1H), 3.81 (dd, *J* = 11.4, 5.0 Hz, 1H), 3.68 (dd, *J* = 11.3, 7.8 Hz, 1H), 2.25 – 2.16 (m, 1H), 2.15 – 2.03 (m, 1H), 2.01 – 1.81 (m, 2H).
^{13}C NMR (151 MHz, $CDCl_3$) δ 166.54, 133.03, 130.09, 129.56, 128.41, 64.03, 60.44, 47.96, 31.73, 25.27.
The compound characterization was reported in literature.[11]

4,5-dichloropentyl 4-methoxybenzoate

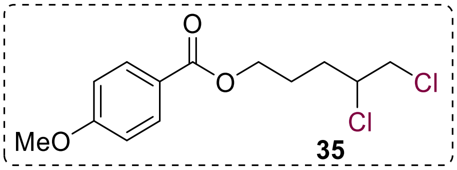

Prepared according to General Procedure B and obtained as colorless oil. **Yield** 52%, 15.1 mg. Purification is through preparatory thin-layer chromatography (with eluent of Hex: EA = 10:1) to give the corresponding dichlorination products.
^{1}H NMR (600 MHz, $CDCl_3$) δ 8.05 – 7.96 (m, 2H), 6.98-6.87 (m, 2H), 4.39 – 4.30 (m, 2H), 4.15-4.08 (m, 1H), 3.87 (s, 3H), 3.81 (dd, *J* = 11.3, 5.0 Hz, 1H), 3.68 (dd, *J* = 11.3, 7.8 Hz, 1H), 2.25 – 2.16 (m, 1H), 2.12-2.01 (m, 1H), 1.97 – 1.83 (m, 2H).
^{13}C NMR (151 MHz, $CDCl_3$) δ 166.29, 163.38, 131.59, 122.50, 113.63, 63.72, 60.48, 55.44, 47.99, 31.76, 25.31.
HRMS APCI: $[M+H]^+$ calcd. for $C_{13}H_{17}C_{12}O_3$: 291.0549; Found 291.0541

4,5-dichloropentyl 4-bromobenzoate

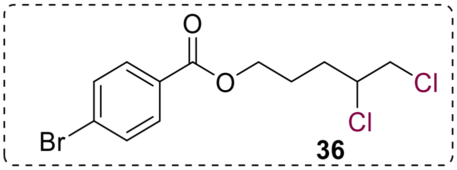

Prepared according to General Procedure B and obtained as colorless oil. **Yield** 55%, 18.7 mg. Purification is through preparatory thin-layer chromatography (with eluent of Hex: EA = 10:1) to give the corresponding dichlorination products.
^{1}H NMR (600 MHz, $CDCl_3$) δ 8.02 – 7.85 (m, 2H), 7.65 – 7.38 (m, 2H), 4.44 – 4.33 (m, 2H), 4.17 – 4.07 (m, 1H), 3.81 (dd, *J* = 11.3, 4.9 Hz, 1H), 3.67 (dd, *J* = 11.3, 7.9 Hz, 1H), 2.24-2.16 (m, 1H), 2.11 – 2.05 (m, 1H), 1.97 – 1.82 (m, 2H).
^{13}C NMR (151 MHz, $CDCl_3$) δ 165.80, 131.76, 131.09, 130.96, 128.98, 128.77, 128.16, 64.29, 60.33, 47.88, 31.64, 25.19.
HRMS APCI: $[M+Cl]^-$ calcd. for $C_{12}H_{13}BrCl_3O_2$: 372.9170; Found 372.9167

4,5-dichloropentyl 4-nitrobenzoate

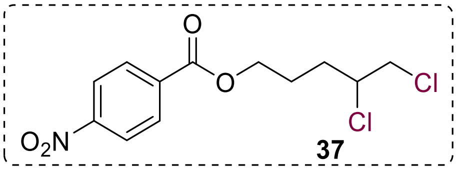

Prepared according to General Procedure B and obtained as colorless oil. **Yield** 73%, 22.2 mg. Purification is through preparatory thin-layer chromatography (with eluent of Hex: EA = 5:1) to give the corresponding dichlorination products.
^{1}H NMR (600 MHz, $CDCl_3$) δ 8.32 – 8.28 (m, 2H), 8.24 – 8.19 (m, 2H), 4.49 – 4.39 (m, 2H), 4.16-4.07 (m, 1H), 3.83 (dd, *J* = 11.3, 4.8 Hz, 1H), 3.67 (dd, *J* = 11.3, 8.1 Hz, 1H), 2.27-2.18 (m, 1H), 2.18 – 2.07 (m, 1H), 2.02-1.92 (m, 1H), 1.92 – 1.84 (m, 1H).
^{13}C NMR (151 MHz, $CDCl_3$) δ 164.62, 150.59, 135.48, 130.71, 123.60, 65.00, 60.19, 47.80, 31.58, 25.14.
HRMS APCI: $[M]^-$ calcd. for $C_{12}H_{13}Cl_2NO_4$: 305.0222; Found 305.0225

(2,3-dichloropropoxy)benzene

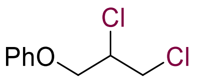

38

Prepared according to General Procedure B and obtained as colorless oil. **Yield** 56%, 11.4 mg. Purification is through preparatory thin-layer chromatography (with eluent of Hex: EA = 15:1) to give the corresponding dichlorination products.

^{1}H NMR (600 MHz, CDCl$_3$) δ 7.34 – 7.28 (m, 2H), 7.03-6.98 (m, 1H), 6.97 – 6.89 (m, 2H), 4.38 (tt, *J* = 6.3, 5.0 Hz, 1H), 4.32 – 4.24 (m, 2H), 3.97 (dd, *J* = 11.6, 6.5 Hz, 1H), 3.91 (dd, *J* = 11.6, 5.0 Hz, 1H).
^{13}C NMR (151 MHz, CDCl$_3$) δ 157.92, 129.63, 121.66, 114.70, 68.05, 57.29, 45.02.
HRMS APCI: $[M+H]^+$ calcd. for $C_9H_{11}Cl_2O$: 205.0181; Found 205.0177

4,5-dichloropentyl (2,2,2-trichloroethyl) carbonate

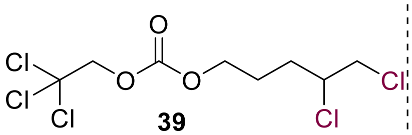

39

Prepared according to General Procedure B and obtained as colorless oil. **Yield** 79%, 26.2 mg. Purification is through preparatory column chromatography (with eluent of Hex: EA = 15:1) to give the corresponding dichlorination products.

^{1}H NMR (600 MHz, CDCl$_3$) δ 4.78 (s, 2H), 4.30 (td, *J* = 6.2, 3.7 Hz, 2H), 4.12-4.03 (m, 1H), 3.80 (dd, *J* = 11.4, 4.9 Hz, 1H), 3.66 (dd, *J* = 11.4, 7.8 Hz, 1H), 2.22-2.12 (m, 1H), 2.09 – 1.97 (m, 1H), 1.96 – 1.72 (m, 2H).
^{13}C NMR (151 MHz, CDCl$_3$) δ 153.94, 94.36, 76.75, 68.26, 60.27, 47.90, 31.32, 25.20.
HRMS APCI: $[M+Cl]^-$ calcd. for $C_8H_{11}Cl_6O_3$: 364.8845; Found 364.8842

2-(3,4-dichlorobutyl)isoindoline-1,3-dione

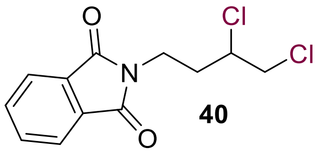

40

Prepared according to General Procedure B and obtained as white solid. **Yield** 85%, 23.1 mg. Purification is through preparatory thin-layer chromatography (with eluent of Hex: EA = 5:1) to give the corresponding dichlorination products.

^{1}H NMR (600 MHz, CDCl$_3$) δ 7.91 – 7.84 (m, 2H), 7.77-7.71 (m, 2H), 4.14-4.05 (m, 1H), 3.98 – 3.86 (m, 2H), 3.84 (dd, *J* = 11.5, 4.9 Hz, 1H), 3.70 (dd, *J* = 11.5, 7.4 Hz, 1H), 2.52-2.41 (m, 1H), 2.16 – 2.08 (m, 1H).
^{13}C NMR (151 MHz, CDCl$_3$) δ 168.21, 134.12, 131.94, 123.38, 58.11, 47.89, 35.07, 33.88.
HRMS APCI: $[M+H]^+$ calcd. for $C_{12}H_{12}Cl_2NO_2$: 272.0240; Found 272.0232

***N*-(3,4-dichlorobutyl)-*N*-methylbenzenesulfonamide**

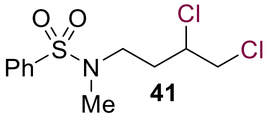

41

Prepared according to General Procedure B and obtained as colorless oil. **Yield** 58%, 17.1 mg. Purification is through preparatory thin-layer chromatography (with eluent of Hex: EA = 5:1) to give the corresponding dichlorination products.

^{1}H NMR (600 MHz, CDCl$_3$) δ 7.84 – 7.79 (m, 2H), 7.64 – 7.60 (m, 1H), 7.57 – 7.53 (m, 2H), 4.24 – 4.18 (m, 1H), 3.87 (dd, *J* = 11.5, 4.6 Hz, 1H), 3.71 (dd, *J* = 11.5, 7.2 Hz, 1H), 3.32-3.23 (m, 1H), 3.17-3.08 (m, 1H), 2.78 (s, 3H), 2.39-2.29 (m, 1H), 1.94-1.86 (m, 1H).
^{13}C NMR (151 MHz, CDCl$_3$) δ 136.93, 132.83, 129.20, 127.42, 57.65, 48.08, 47.25, 35.52, 33.50.
HRMS APCI: $[M+H]^+$ calcd. for $C_{11}H_{16}Cl_2NO_2S$: 296.0273; Found 296.0265

***N*-(3,4-dichlorobutyl)benzenesulfonamide**

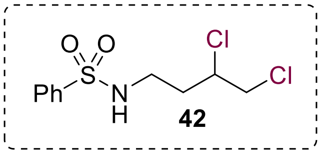

42

Prepared according to General Procedure B and obtained as colorless oil. **Yield** 52%, 14.7 mg. Purification is through preparatory thin-layer chromatography (with eluent of Hex: EA = 3:1) to give the corresponding dichlorination products.

^{1}H NMR (600 MHz, CDCl$_3$) δ 7.91 – 7.85 (m, 2H), 7.64 – 7.58 (m, 1H), 7.55 (dd, *J* = 8.3, 6.9 Hz, 2H), 4.76 (t, *J* = 6.5 Hz, 1H), 4.17-4.10 (m, 1H), 3.76 (dd, *J* = 11.5, 4.8 Hz, 1H), 3.63 (dd, *J* = 11.5, 7.3 Hz, 1H), 3.25 – 3.13 (m, 2H), 2.29-2.21 (m, 1H), 1.88-1.78 (m, 1H).

^{13}C NMR (151 MHz, CDCl$_3$) δ 139.53, 132.94, 129.29, 127.05, 57.82, 47.99, 40.12, 35.10.

HRMS APCI: $[M+H]^+$ calcd. for $C_{10}H_{14}Cl_2NO_2S$: 282.0117; Found 282.0109

6-bromo-1,2-dichlorohexane

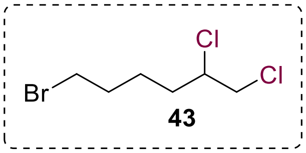

43

Prepared according to General Procedure B and obtained as colorless oil. **Yield** 82%, 19.1 mg. Purification is through preparatory column chromatography (with eluent of Hexane) to give the corresponding dichlorination products.

^{1}H NMR (600 MHz, CDCl$_3$) δ 4.07-4.01 (m, 1H), 3.78 (dd, *J* = 11.3, 5.0 Hz, 1H), 3.65 (dd, *J* = 11.3, 7.7 Hz, 1H), 3.43 (t, *J* = 6.7 Hz, 2H), 2.10 – 2.00 (m, 1H), 1.99 – 1.84 (m, 2H), 1.82 – 1.70 (m, 2H), 1.63 – 1.56 (m, 1H).

^{13}C NMR (151 MHz, CDCl$_3$) δ 60.63, 47.99, 34.12, 33.14, 32.02, 24.53.

GC-MS: $[M]^+$ calcd. for $C_6H_{11}BrCl_2$: 231.9421; Found 232

diethyl 2-(2,3-dichloropropyl)malonate

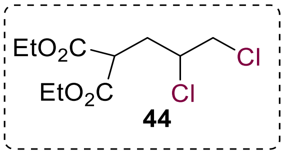

44

Prepared according to General Procedure B and obtained as colorless oil. **Yield** 80%, 21.7 mg. Purification is through preparatory column chromatography (with eluent of Hex: EA = 7:1) to give the corresponding dichlorination products.

^{1}H NMR (600 MHz, CDCl$_3$) δ 4.31 – 4.17 (m, 4H), 4.17-4.10 (m, 1H), 3.80 (ddd, *J* = 11.5, 5.0, 1.5 Hz, 1H), 3.76 – 3.65 (m, 2H), 2.72-2.64 (m, 1H), 2.23-2.12 (m, 1H), 1.33-1.23 (m, 6H).

^{13}C NMR (151 MHz, CDCl$_3$) δ 168.69, 168.47, 61.88, 61.82, 58.48, 49.06, 48.23, 34.42, 14.05, 14.02.

HRMS APCI: $[M+H]^+$ calcd. for $C_{10}H_{17}Cl_2O_4$ 271.0498; Found 271.0491

(2,3-dichloro-2-methylpropyl)benzene

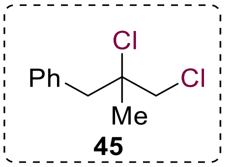

45

Prepared according to General Procedure B and obtained as colorless oil. **Yield** 73%, 14.8 mg. Purification is through preparatory thin-layer chromatography (with eluent of Hexane) to give the corresponding dichlorination products.

^{1}H NMR (600 MHz, CDCl$_3$) δ 7.41 – 7.26 (m, 5H), 3.63 (s, 2H), 3.14 (d, *J* = 14.1 Hz, 2H), 1.67 (s, 3H).

^{13}C NMR (151 MHz, CDCl$_3$) δ 135.51, 130.84, 128.12, 127.21, 70.62, 51.93, 45.91, 28.25.

GC-MS: $[M]^+$ calcd. for $C_{10}H_{12}Cl_2$: 202.0316; Found 202

(2,3-dichloro-2-methylpropoxy)benzene

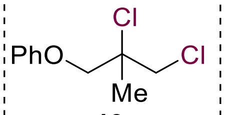

46

Prepared according to General Procedure B and obtained as colorless oil. **Yield** 70%, 15.3 mg. Purification is through preparatory thin-layer chromatography (with eluent of Hex: EA = 15:1) to give the corresponding dichlorination products.

^{1}H NMR (600 MHz, CDCl$_3$) δ 7.35 – 7.28 (m, 2H), 7.02-6.97 (m, 1H), 6.97 – 6.91 (m, 2H), 4.21 – 4.10 (m, 2H), 4.01 (d, J = 11.4 Hz, 1H), 3.83 (d, J = 11.4 Hz, 1H), 1.75 (s, 3H).

^{13}C NMR (151 MHz, CDCl$_3$) δ 158.15, 129.56, 121.56, 114.79, 72.00, 67.96, 50.04, 25.28.

HRMS APCI: $[M+H]^+$ calcd. for $C_{10}H_{13}Cl_2O$: 219.0338; Found 219.0332

2,3-dichlorobutane-1,4-diyl dibenzoate

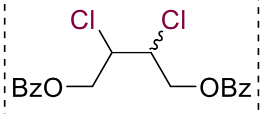

47 2.6:1 d.r.

Prepared according to General Procedure B and obtained as white solid in the mixture of two isomers. **Yield** 68%, 24.9 mg. d.r. 2.6:1. Purification is through preparatory thin-layer chromatography (with eluent of Hex: EA = 5:1) to give the corresponding dichlorination products.

^{1}H NMR (600 MHz, CDCl$_3$) δ 8.13 – 7.99 (m, 4H), 7.64-7.56 (m, 2H), 7.51-7.43 (m, 4H), 4.87 – 4.77 (m, 3H), 4.73 – 4.69 (m, 0.56H), 4.67 – 4.62 (m, 1H), 4.58 – 4.52 (m, 1.44H).

^{13}C NMR (151 MHz, CDCl$_3$) δ 165.84, 133.54, 133.48, 129.77, 129.76, 129.26, 128.56, 128.54, 65.27, 64.95, 58.06, 57.43.

HRMS APCI: $[M+H]^+$ calcd. for $C_{18}H_{17}Cl_2O_4$: 367.0498; Found 367.0489

3,4-dichlorohexyl benzoate

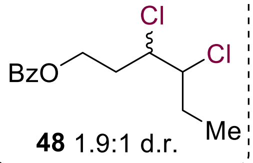

48 1.9:1 d.r.

Prepared according to General Procedure B and obtained as colorless oil in a mixture of two isomers. **Yield** 67%, 18.4 mg. d.r. 1.9:1. Purification is through preparatory thin-layer chromatography (with eluent of Hex: EA = 10:1) to give the corresponding dichlorination products.

^{1}H NMR (600 MHz, CDCl$_3$) δ 8.13-8.01 (m, 2H), 7.63-7.54 (m, 1H), 7.49-7.41 (m, 2H), 4.64 – 4.54 (m, 1H), 4.54 – 4.45 (m, 1H), 4.34-4.29 (m, 0.34H), 4.22-4.16 (m, 0.66H), 4.06 – 3.97 (m, 1H), 2.62 – 2.55 (m, 0.67H), 2.48-2.39 (m, 0.34)2.28 – 2.14 (m, 1H), 2.12 – 1.99 (m, 1H), 1.90-1.80 (m, 1H), 1.13-1.05 (m, 3H).

^{13}C NMR (151 MHz, CDCl$_3$) δ 166.35, 133.13, 133.10, 129.97, 129.94, 129.58, 128.44, 128.43, 67.43, 67.00, 61.70, 61.67, 61.58, 61.36, 34.01, 33.88, 28.25, 27.80, 11.42, 10.54.

HRMS APCI: $[M+H]^+$ calcd. for $C_{13}H_{17}Cl_2O_2$: 275.0600; Found 275.0592

2,3-dichloro-3-methylbutyl benzoate

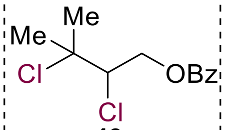

49

Prepared according to General Procedure B and obtained as colorless oil. **Yield** 78%, 20.4 mg. Purification is through preparatory thin-layer chromatography (with eluent of Hex: EA = 10:1) to give the corresponding dichlorination products.

^{1}H NMR (600 MHz, CDCl$_3$) δ 8.13 – 8.04 (m, 2H), 7.62 – 7.55 (m, 1H), 7.49-7.43 (m, 2H), 5.04-4.97 (m, 1H), 4.63-4.52 (m, 1H), 4.39-4.31 (m, 1H), 1.80 (d, J = 2.2 Hz, 3H), 1.72 (d, J = 2.1 Hz, 3H).
^{13}C NMR (151 MHz, CDCl$_3$) δ 166.15, 133.31, 129.76, 129.59, 128.47, 69.20, 67.13, 66.04, 31.75, 27.81.
HRMS APCI: [M+H]$^+$ calcd. for $C_{12}H_{15}Cl_2O_2$: 261.0444; Found 261.0437

1,2-dichlorocyclooctane

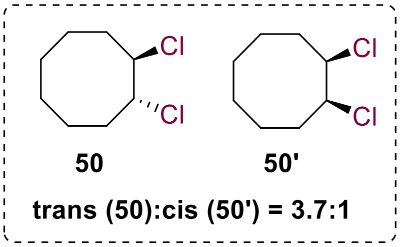

Prepared according to General Procedure B and obtained as colorless oil. **Yield** 52%, 9.4 mg. d.r. = 3.7:1 (trans:cis).[12] Purification is through preparatory column chromatography (with eluent of Hexane) to give the corresponding dichlorination products.
^{1}H NMR (600 MHz, CDCl$_3$) δ 4.51-4.46 (m, 0.42H), 4.33 – 4.24 (m, 1.58H), 2.36 – 2.18 (m, 2H), 2.15 – 2.00 (m, 2H), 1.98 – 1.81 (m, 2H), 1.79 – 1.65 (m, 2H), 1.64 – 1.56 (m, 2H), 1.55-1.52 (m, 0.42H) 1.47-1.37 (m, 1.58H).
^{13}C NMR (151 MHz, CDCl$_3$) δ 68.13, 33.28, 25.42, 25.04.
The compound characterization was reported in literature.[12]

(5*R*)-5-(1,2-dichloropropan-2-yl)-2-methylcyclohex-2-en-1-one

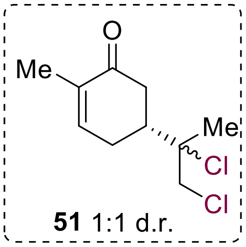

Prepared according to General Procedure B and obtained as colorless oil in a mixture of two isomers. **Yield** 52%, 15.9 mg. d.r. 1:1. Purification is through preparatory thin-layer chromatography (with eluent of Hex: EA = 5:1) to give the corresponding dichlorination products.
^{1}H NMR (600 MHz, CDCl$_3$) δ 6.81 – 6.67 (m, 1H), 3.85 (dd, J = 14.0, 11.4 Hz, 1H), 3.70 (dd, J = 15.8, 11.4 Hz, 1H), 2.70 – 2.55 (m, 2H), 2.55 – 2.39 (m, 3H), 1.80 (q, J = 1.8 Hz, 3H), 1.68 (d, J = 9.6 Hz, 3H).
^{13}C NMR (151 MHz, CDCl$_3$) δ 198.75, 198.44, 143.86, 143.76, 135.38, 135.28, 73.22, 73.17, 50.81, 50.77, 41.14, 40.97, 39.02, 38.78, 26.87, 26.67, 26.31, 26.29, 15.62, 15.60.
HRMS APCI: [M+H]$^+$ calcd. for $C_{10}H_{15}Cl_2O$: 221.0494; Found 221.0488

(1,2-dichloroethyl)benzene

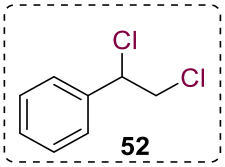

Prepared according to General Procedure B and obtained as colorless oil. **Yield** 74%, 13.0 mg. Purification is through preparatory thin-layer chromatography (with eluent of Hexane) to give the corresponding dichlorination products.
^{1}H NMR (600 MHz, CDCl$_3$) δ 7.50 – 7.32 (m, 5H), 5.00 (dd, J = 8.0, 6.6 Hz, 1H), 4.00 (dd, J = 11.3, 6.6 Hz, 1H), 3.93 (dd, J = 11.4, 8.0 Hz, 1H).
^{13}C NMR (151 MHz, CDCl$_3$) δ 137.97, 129.17, 128.82, 127.39, 61.73, 48.33.
The compound characterization was reported in literature.[13]

1,2-dichloro-2,3-dihydro-1*H*-indene

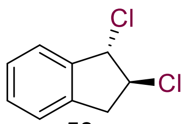

53
trans:cis > 20:1

Prepared according to General Procedure B and obtained as colorless oil in single isomer. **Yield** 65%, 12.2 mg. d.r. > 20:1 (trans:cis).[13] Purification is through preparatory thin-layer chromatography (with eluent of Hexane) to give the corresponding dichlorination products.
^{1}H NMR (600 MHz, $CDCl_3$) δ 7.49 – 7.42 (m, 1H), 7.37 – 7.27 (m, 3H), 5.35 (d, *J* = 2.9 Hz, 1H), 4.66 (dt, *J* = 6.2, 3.2 Hz, 1H), 3.71 (dd, *J* = 16.7, 6.0 Hz, 1H), 3.18 (dd, *J* = 16.7, 3.4 Hz, 1H).
^{13}C NMR (151 MHz, $CDCl_3$) δ 139.83, 139.79, 129.69, 127.92, 125.48, 125.10, 67.57, 64.46, 40.71.
The compound characterization was reported in literature.[13]

1,2-dichloropropyl)benzene

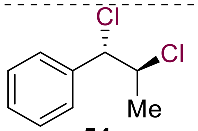

54
trans:cis > 20:1

Prepared according to General Procedure B and obtained as colorless oil in single isomer. **Yield** 52%, 9.8 mg. d.r. > 20:1 (trans:cis).[13] Purification is through preparatory thin-layer chromatography (with eluent of Hexane) to give the corresponding dichlorination products.
^{1}H NMR (600 MHz, $CDCl_3$) δ 7.42 – 7.33 (m, 5H), 4.91 (d, *J* = 7.8 Hz, 1H), 4.42 – 4.35 (m, 1H), 1.71 (d, *J* = 6.5 Hz, 3H).
^{13}C NMR (151 MHz, $CDCl_3$) δ 138.64, 128.79, 128.53, 127.74, 67.40, 60.20, 22.17.
The compound characterization was reported in literature.[13]

methyl 2,3-dichloro-3-phenylpropanoate

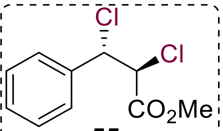

55
trans:cis > 20:1

Prepared according to General Procedure B and obtained as colorless oil in single isomer. **Yield** 64%, 14.9 mg. d.r. > 20:1 (trans:cis).[14] Purification is through preparatory thin-layer chromatography (with eluent of Hexane) to give the corresponding dichlorination products.
^{1}H NMR (600 MHz, $CDCl_3$) δ 7.46 – 7.36 (m, 5H), 5.18 (d, *J* = 10.7 Hz, 1H), 4.62 (d, *J* = 10.7 Hz, 1H), 3.90 (s, 3H).
^{13}C NMR (151 MHz, $CDCl_3$) δ 168.01, 136.30, 129.48, 128.81, 128.07, 61.00, 58.76, 53.42.
HRMS APCI: $[M+NH4]^+$ calcd. for $C_{10}H_{14}Cl_2NO_2$: 250.0396; Found 250.0392

2,3-dichloro-3-phenylpropyl benzoate

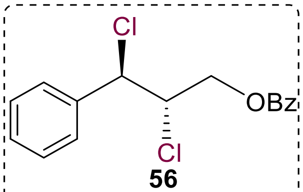

56
trans:cis > 20:1

Prepared according to General Procedure B and obtained as colorless oil in single isomer. **Yield** 53%, 16.4 mg. d.r. > 20:1 (trans:cis).[13] Purification is through preparatory thin-layer chromatography (with eluent of Hex: EA = 10:1) to give the corresponding dichlorination products.
^{1}H NMR (600 MHz, $CDCl_3$) δ 8.12 – 8.05 (m, 2H), 7.64 – 7.57 (m, 1H), 7.51 – 7.44 (m, 4H), 7.43 – 7.36 (m, 3H), 5.16 (d, *J* = 8.5 Hz, 1H), 4.80 (dd, *J* = 4.7, 2.3 Hz, 1H), 4.65 (dt, *J* = 9.0, 4.7 Hz, 1H).

^{13}C NMR (151 MHz, CDCl$_3$) δ 165.90, 137.51, 133.42, 129.77, 129.43, 129.15, 128.72, 128.70, 128.53, 127.92, 65.51, 62.04, 61.62.
The compound characterization was reported in literature.[13]

4,5-diazidopentyl 2-(4-isobutylphenyl)propanoate

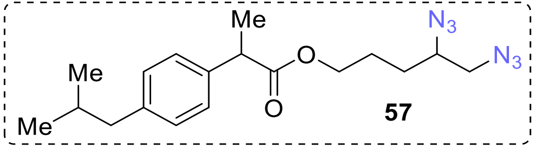

57

Prepared according to General Procedure A and obtained as colorless oil. **Yield** 68%, 24.3 mg. Purification is through preparatory thin-layer chromatography (with eluent of Hex: EA = 10:1) to give the corresponding diazidation products.
^{1}H NMR (600 MHz, CDCl$_3$) δ 7.19 (d, J = 8.1 Hz, 2H), 7.10 (d, J = 8.1 Hz, 2H), 4.18-4.02 (m, 2H), 3.69 (q, J = 7.1 Hz, 1H), 3.41-3.33 (m, 1H), 3.28 (dd, J = 12.7, 4.1 Hz, 1H), 3.23 (dd, J = 12.7, 7.3 Hz, 1H), 2.45 (d, J = 7.2 Hz, 2H), 1.89-1.81 (m, 1H), 1.79 – 1.71 (m, 1H), 1.68 – 1.62 (m, 1H), 1.49 (d, J = 7.2 Hz, 3H), 1.46 – 1.40 (m, 2H), 0.90 (d, J = 6.6 Hz, 6H).
^{13}C NMR (151 MHz, CDCl$_3$) δ 174.67, 140.66, 137.72, 137.71, 129.37, 127.15, 63.81, 63.79, 63.66, 61.44, 61.41, 54.77, 54.76, 45.16, 45.14, 45.13, 45.02, 30.21, 28.30, 28.18, 25.05, 25.01, 22.39, 18.33, 18.29.
HRMS APCI: $[M+H]^+$ calcd. for $C_{18}H_{27}N_6O_2$: 359.2190; Found 359.2184

4,5-diazidopentyl 2-(2-fluoro-[1,1'-biphenyl]-4-yl)propanoate

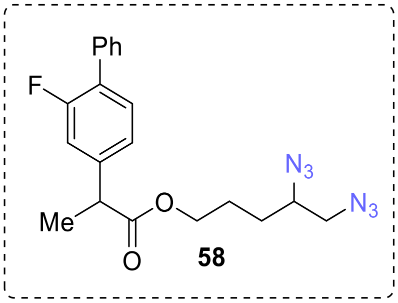

58

Prepared according to General Procedure A and obtained as colorless oil. **Yield** 65%, 25.7 mg. Purification is through preparatory thin-layer chromatography (with eluent of Hex: EA = 10:1) to give the corresponding diazidation products.
^{1}H NMR (600 MHz, CDCl$_3$) δ 7.57-7.51 (m, 2H), 7.47-7.42 (m, 2H), 7.42 – 7.34 (m, 2H), 7.17 – 7.09 (m, 2H), 4.21 – 4.07 (m, 2H), 3.76 (q, J = 7.2 Hz, 1H), 3.45 – 3.38 (m, 1H), 3.34 (ddd, J = 12.7, 4.2, 1.6 Hz, 1H), 3.28 (dd, J = 12.7, 7.2 Hz, 1H), 1.84-1.74 (m, 1H), 1.74 – 1.65 (m, 1H), 1.55 – 1.44 (m, 5H).
^{13}C NMR (151 MHz, CDCl$_3$) δ 173.91, 159.66 (d, J = 248.4 Hz), 141.70 (d, J = 7.6 Hz), 135.36, 130.84 (d, J = 3.9 Hz), 128.91 (d, J = 3.0 Hz), 128.49, 127.89 (d, J = 13.6 Hz), 127.73, 123.53 (d, J = 3.3 Hz), 115.21 (d, J = 23.6 Hz), 64.11 (d, J = 14.3 Hz), 61.41 (d, J = 3.6 Hz), 54.75, 45.00, 28.26 (d, J = 13.6 Hz), 25.04 (d, J = 5.4 Hz), 18.23 (d, J = 4.5 Hz).
^{19}F NMR (565 MHz, CDCl$_3$) δ -117.52 (t, J = 10.5 Hz).
The compound characterization was reported in literature.[6]

4,5-diazidopentyl 3-methyl-4-oxo-2-phenyl-4*H*-chromene-8-carboxylate

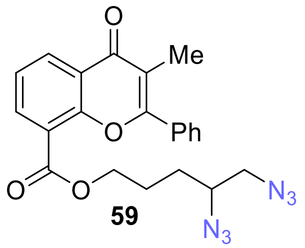

59

Prepared according to General Procedure A and obtained as colorless oil. **Yield** 84%, 36.3 mg. Purification is through preparatory thin-layer chromatography (with eluent of Hex:EA= 2:1) to give the corresponding diazidation products.

^{1}H NMR (600 MHz, CDCl$_3$) δ 8.48 (dd, *J* = 7.9, 1.8 Hz, 1H), 8.27 (dd, *J* = 7.5, 1.8 Hz, 1H), 7.81 – 7.75 (m, 2H), 7.60-7.53 (m, 3H), 7.46 (t, *J* = 7.7 Hz, 1H), 4.48 – 4.31 (m, 2H), 3.39-3.31 (m, 1H), 3.27 (dd, *J* = 12.7, 4.1 Hz, 1H), 3.22 (dd, *J* = 12.7, 7.4 Hz, 1H), 2.23 (s, 3H), 1.95 – 1.87 (m, 1H), 1.84 – 1.76 (m, 1H), 1.59 – 1.46 (m, 2H).

^{13}C NMR (151 MHz, CDCl$_3$) δ 178.25, 164.53, 161.00, 154.45, 136.25, 133.10, 131.01, 130.59, 129.34, 128.51, 124.11, 123.33, 120.44, 117.82, 64.76, 61.51, 54.79, 28.43, 25.19, 11.80.

The compound characterization was reported in literature.[6]

4,5-diazidopentyl 4-(*N*,*N*-dipropylsulfamoyl)benzoate

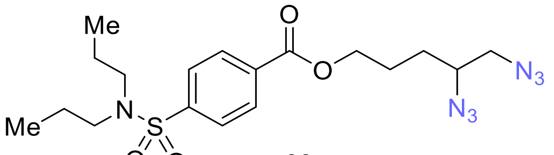

60

Prepared according to General Procedure A and obtained as colorless oil. **Yield** 71%, 31.0mg. Purification is through preparatory thin-layer chromatography (with eluent of Hex: EA = 3:1) to give the corresponding diazidation products.

^{1}H NMR (600 MHz, CDCl$_3$) δ 8.15 (d, *J* = 8.4 Hz, 2H), 7.89 (d, *J* = 8.4 Hz, 2H), 4.45 – 4.35 (m, 2H), 3.59-3.52 (m, 1H), 3.47 (dd, *J* = 12.7, 4.2 Hz, 1H), 3.40 (dd, *J* = 12.7, 7.1 Hz, 1H), 3.14 – 3.06 (m, 4H), 2.03-1.95 (m, 1H), 1.94-1.85 (m, 1H), 1.76 – 1.63 (m, 2H), 1.60-1.50 (m, 4H), 0.87 (t, *J* = 7.4 Hz, 6H).

^{13}C NMR (151 MHz, CDCl$_3$) δ 165.18, 144.35, 133.30, 130.22, 127.05, 64.80, 61.51, 54.80, 49.90, 28.44, 25.24, 21.92, 11.16.

HRMS APCI: $[M+H]^+$ calcd. for $C_{18}H_{28}N_7O_4S$: 438.1918; Found 438.1908

(2*S*,4*R*)-1,7,7-trimethylbicyclo[2.2.1]heptan-2-yl 4,5-diazidopentanoate

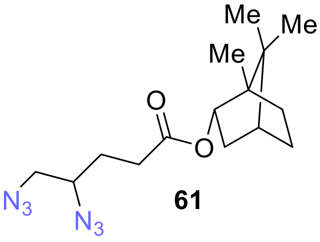

61

Prepared according to General Procedure A and obtained as colorless oil. **Yield** 59%, 18.9 mg. Purification is through preparatory column chromatography (with eluent of Hex: EA = 10:1) to give the corresponding diazidation products.

^{1}H NMR (600 MHz, CDCl$_3$) δ 4.93-4.87 (m, 1H), 3.61-3.52 (m, 1H), 3.45 (dd, *J* = 12.7, 4.1 Hz, 1H), 3.36 (dd, *J* = 12.7, 7.3 Hz, 1H), 2.53-2.44 (m, 2H), 2.40-2.31 (m, 1H), 1.95-1.86 (m, 2H), 1.83 – 1.72 (m, 2H), 1.72-1.66 (m, 1H), 1.35 – 1.26 (m, 1H), 1.26-1.19 (m, 1H), 0.98-0.93 (m, 1H), 0.90 (s, 3H), 0.87 (s, 3H), 0.83 (d, *J* = 2.4 Hz, 3H).

^{13}C NMR (151 MHz, CDCl$_3$) δ 172.75, 80.42, 61.23, 54.90, 48.78, 47.85, 44.86, 36.80, 30.68, 28.05, 28.03, 27.16, 27.15, 27.12, 19.70, 18.83, 13.54.

HRMS APCI: $[M+H]^+$ calcd. for $C_{15}H_{25}N_6O_2$: 321.2034; Found 321.2030

(1*R*,2*S*,5*R*)-2-isopropyl-5-methylcyclohexyl 4,5-diazidopentanoate

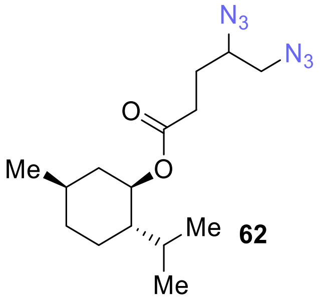

62

Prepared according to General Procedure A and obtained as colorless oil. **Yield** 42%, 13.5 mg. Purification is through preparatory column chromatography (with eluent of Hex: EA = 10:1) to give the corresponding diazidation products.

^{1}H NMR (600 MHz, CDCl$_3$) δ 4.77-4.64 (m, 1H), 3.60-3.53 (m, 1H), 3.45 (dd, *J* = 12.8, 4.1 Hz, 1H), 3.36 (dd, *J* = 12.7, 7.4 Hz, 1H), 2.51-2.38 (m, 2H), 2.00-1.93 (m, 1H), 1.93-1.80 (m, 2H), 1.79-1.72 (m, 1H), 1.71-1.65 (m, 2H), 1.52-1.45 (m, 1H), 1.41-1.33 (m, 1H), 1.09 – 0.96 (m, 2H), 0.91-0.86 (m, 7H), 0.76 (dd, *J* = 7.0, 2.2 Hz, 3H).

^{13}C NMR (151 MHz, CDCl$_3$) δ 172.02, 74.69 (d, *J* = 2.8 Hz), 61.24 (d, *J* = 3.1 Hz), 54.91, 46.99 (d, *J* = 2.1 Hz), 40.91 (d, *J* = 5.0 Hz), 34.20, 31.40, 30.69, 27.14 (d, *J* = 4.9 Hz), 26.36 (d, *J* = 5.4 Hz), 23.41 (d, *J* = 1.9 Hz)., 22.01, 20.75 (d, *J* = 2.4 Hz), 16.31 (d, *J* = 2.7 Hz).

HRMS APCI: $[M-N2+H]^+$ calcd. for $C_{19}H_{27}N_4O_2$: 295.2129; Found 295.2124

(4*R*,5*R*,6*R*)-6-(1,2-diazidopropan-2-yl)-4,5-dimethyl-4,4a,5,6,7,8-hexahydronaphthalen-2(3*H*)-on

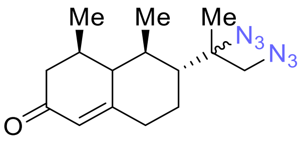

63 1:1 d.r.

Prepared according to General Procedure A and obtained as colorless oil in a mixture of isomers. **Yield** 60%, 18.1 mg. d.r. 1:1. Purification is through preparatory thin-layer chromatography (with eluent of Hex: EA = 2:1) to give the corresponding diazidation products.

^{1}H NMR (600 MHz, CDCl$_3$) δ 5.80-5.70 (m, 1H), 3.45-3.31 (m, 2H), 2.53 – 2.43 (m, 1H), 2.43-2.35 (m, 1H), 2.31 – 2.20 (m, 2H), 2.04 – 1.91 (m, 3H), 1.91 – 1.81 (m, 1H), 1.28 – 1.21 (m, 3H), 1.24-1.07 (m, 3H), 1.01-0.96 (m, 3H).

^{13}C NMR (151 MHz, CDCl$_3$) δ 199.32, 199.25, 169.06, 169.04, 124.84, 65.90, 65.75, 58.08, 57.73, 41.99, 40.49, 39.23, 39.20, 39.00, 38.77, 32.54, 32.48, 27.33, 27.13, 18.27, 17.97, 16.86, 16.81, 15.00, 14.98.

HRMS APCI: $[M+H]^+$ calcd. for $C_{15}H_{23}N_6O$: 303.1928; Found 303.1922

benzyl 9,10-diazidooctadecanoate

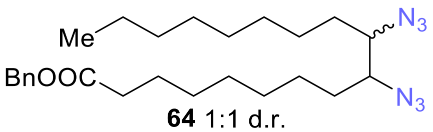

64 1:1 d.r.

Prepared according to General Procedure A and obtained as colorless oil in the mixture of two isomers. **Yield** 70%, 31.9 mg. d.r. = 1:1. Purification is through preparatory thin-layer chromatography (with eluent of Hex: EA = 15:1) to give the corresponding diazidation products.

^{1}H NMR (600 MHz, CDCl$_3$) δ 7.39 – 7.30 (m, 5H), 5.12 (s, 2H), 3.32 (h, *J* = 4.9 Hz, 1H), 3.25 (dq, *J* = 6.7, 4.3 Hz, 1H)., 2.36 (t, *J* = 7.5 Hz, 2H), 1.69-1.28 (m, 24H), 0.89 (t, *J* = 7.0 Hz, 3H).

^{13}C NMR (151 MHz, CDCl$_3$) δ 173.64, 136.06, 128.55, 128.19, 66.11, 65.86, 65.82, 65.24, 65.20, 34.25, 31.82, 31.26, 31.24, 30.36, 30.28, 29.39, 29.35, 29.33, 29.19, 29.14, 29.04, 28.96, 26.31, 26.25, 26.19, 26.12, 24.84, 22.65, 14.12.

The compound characterization was reported in literature.[6]

(1*R*,5*R*,7*S*)-7-(2,2-dimethyl-1,3-dioxolan-4-yl)-3,3-dimethyl-2,4,6-trioxabicyclo[3.2.1]octan-5-yl 4,5-diazidopentanoate

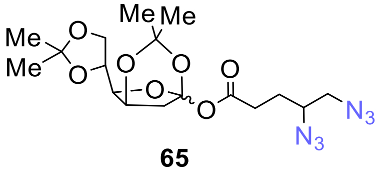

65

Prepared according to General Procedure A and obtained as colorless oil. **Yield** 49%, 20.9 mg. Purification is through preparatory column chromatography (with eluent of Hex: EA = 2:1) to give the corresponding diazidation products.

^{1}H NMR (600 MHz, CDCl$_3$) δ 6.16 (d, *J* = 2.1 Hz, 1H), 4.87 (dd, *J* = 5.9, 3.6 Hz, 1H), 4.71 (dd, *J* = 5.9, 2.1 Hz, 1H), 4.45-4.36 (m, 1H), 4.13 – 4.07 (m, 1H), 4.07-4.01 (m, 2H), 3.62-3.54 (m, 1H), 3.47 (dt, *J* = 12.7, 4.0 Hz, 1H), 3.38 (dd, *J* = 12.7, 7.3 Hz, 1H), 2.54 – 2.43 (m, 2H), 1.96 – 1.85 (m, 1H), 1.80 – 1.71 (m, 1H), 1.49 (s, 3H), 1.47 (s, 3H), 1.38 (s, 3H), 1.35 (s, 3H).

^{13}C NMR (151 MHz, CDCl$_3$) δ 171.02 (d, *J* = 3.3 Hz), 113.36, 109.41, 101.01 (d, *J* = 4.7 Hz), 85.01, 82.33 (d, *J* = 5.5 Hz), 79.25 (d, *J* = 2.7 Hz), 72.83 (d, *J* = 2.3 Hz), 66.78 (d, *J* = 4.7 Hz), 60.95 (d, *J* = 12.0 Hz), 54.84 (d, *J* = 3.2 Hz), 30.31 (d, *J* = 17.4 Hz), 27.00 (d, *J* = 1.9 Hz), 26.72 (d, *J* = 9.8 Hz), 25.93, 25.12 (d, *J* = 2.3 Hz), 24.63 (d, *J* = 2.1 Hz).

HRMS APCI: [M-N2+H]$^+$ calcd. for $C_{17}H_{27}N_4O_7$: 399.1874; Found 399.1864

benzyl (2*S*,4a*S*,6a*S*,6b*R*,8a*R*,10*S*,12a*S*,12b*R*,14b*R*)-10-((4,5-diazidopentanoyl)oxy)-2,4a,6a,6b,9,9,12a-heptamethyl-13-oxo-1,2,3,4,4a,5,6,6a,6b,7,8,8a,9,10,11,12,12a,12b,13,14b-icosahydropicene-2-carboxylate

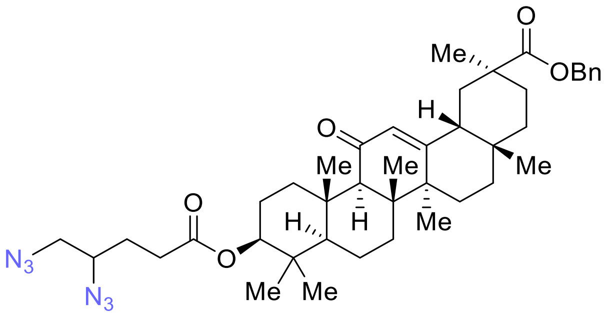

66

Prepared according to General Procedure A and obtained as white solid. **Yield** 51%, 37.0 mg. Purification is through preparatory thin-layer chromatography (with eluent of Hex: EA = 2:1) to give the corresponding diazidation products.

^{1}H NMR (600 MHz, CDCl$_3$) δ 7.43 – 7.31 (m, 5H), 5.55 (s, 1H), 5.20 (d, *J* = 12.2 Hz, 1H), 5.09 (d, *J* = 12.2 Hz, 1H), 4.54 (dd, *J* = 11.8, 4.6 Hz, 1H), 3.62-3.52 (m, 1H), 3.46 (dd, *J* = 12.7, 4.1 Hz, 1H), 3.37 (dd, *J* = 12.9, 7.2 Hz, 1H), 2.84-2.76 (m, 1H), 2.54 – 2.41 (m, 2H), 2.34 (s, 1H), 2.06-1.97 (m, 3H), 1.97 – 1.87 (m, 2H), 1.84 – 1.74 (m, 2H), 1.74 – 1.63 (m, 3H), 1.57 (s, 1H), 1.48 – 1.28 (m, 9H), 1.19-1.14 (m, 7H), 1.11 (s, 3H), 1.07 – 0.98 (m, 2H), 0.91 – 0.86 (m, 6H), 0.82-0.78 (m, 1H), 0.73 (s, 3H).

^{13}C NMR (151 MHz, CDCl$_3$) δ 200.03, 176.23, 172.24, 172.22, 169.21, 169.20, 136.09, 128.61, 128.42, 128.30, 128.24, 81.14, 66.22, 61.63, 61.19, 54.96, 54.87, 48.20, 45.34, 43.98, 43.14, 41.00, 38.72, 38.06, 37.61, 36.87, 32.63, 31.76, 31.13, 30.77, 30.68, 29.70 28.41, 28.28, 28.10, 27.13, 27.07, 26.42, 26.35, 23.59, 23.55, 23.29, 18.63, 17.34, 16.76, 16.41.

The compound characterization was reported in literature.[6]

4,5-dichloropentyl 2-(4-((2-oxocyclopentyl)methyl)phenyl)propanoate

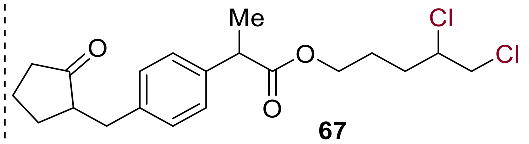
67

Prepared according to General Procedure B and obtained as colorless oil. **Yield** 72%, 27.7 mg. Purification is through preparatory thin-layer chromatography (with eluent of Hex: EA = 5:1) to give the corresponding dichlorination products.

^{1}H NMR (600 MHz, CDCl$_3$) δ 7.24 – 7.19 (m, 2H), 7.15 – 7.09 (m, 2H), 4.18 – 4.05 (m, 2H), 4.00 – 3.92 (m, 1H), 3.75 – 3.66 (m, 2H), 3.61 – 3.51 (m, 1H), 3.13 (dd, *J* = 14.0, 4.1 Hz, 1H), 2.50 (dd, *J* = 13.9, 9.6 Hz, 1H), 2.39 – 2.29 (m, 2H), 2.16 – 2.06 (m, 2H), 2.00-1.92 (m, 2H), 1.90-1.83 (m, 1H), 1.78-1.69 (m, 2H), 1.59-1.51 (m, 1H), 1.49 (d, *J* = 7.1 Hz, 3H).

^{13}C NMR (151 MHz, CDCl$_3$) δ 220.31, 174.58, 138.97, 138.96, 138.29, 129.17, 127.54, 63.76, 63.67, 60.38, 60.35, 51.04, 47.97, 45.14, 38.21, 35.20, 31.56, 31.48, 29.29, 25.08, 20.56, 18.35, 18.33.

HRMS APCI: $[M+H]^+$ calcd. for $C_{20}H_{27}Cl_2O_3$: 385.1332; Found 385.1330

4,5-dichloropentyl 4-(*N*,*N*-dipropylsulfamoyl)benzoate

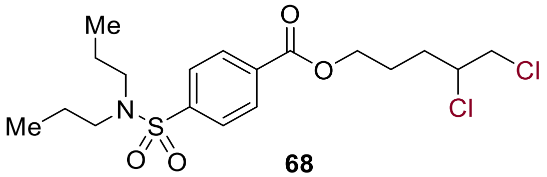
68

Prepared according to General Procedure B and obtained as colorless oil. **Yield** 65%, 27.5 mg. Purification is through preparatory thin-layer chromatography (with eluent of Hex: EA = 3:1) to give the corresponding dichlorination products.

^{1}H NMR (600 MHz, CDCl$_3$) δ 8.19 – 8.13 (m, 2H), 7.92 – 7.85 (m, 2H), 4.45 – 4.37 (m, 2H), 4.16 – 4.09 (m, 1H), 3.82 (dd, *J* = 11.3, 4.9 Hz, 1H), 3.68 (dd, *J* = 11.3, 8.0 Hz, 1H), 3.15 – 3.06 (m, 4H), 2.26-2.18 (m, 1H), 2.16 – 2.06 (m, 1H), 1.99 – 1.84 (m, 2H), 1.60 – 1.49 (m, 4H), 0.87 (t, *J* = 7.4 Hz, 6H).

^{13}C NMR (151 MHz, CDCl$_3$) δ 165.19, 144.30, 133.36, 130.21, 127.04, 64.69, 60.26, 49.91, 47.85, 31.62, 25.17, 21.92, 11.16.

HRMS APCI: $[M+H]^+$ calcd. for $C_{18}H_{28}Cl_2NO_4S$: 424.1111; Found 424.1106

4,5-dichloropentyl 2-(2,4-dichlorophenoxy)acetate

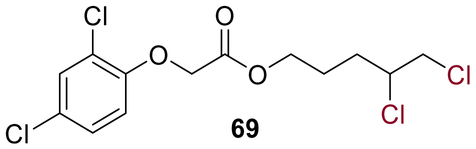
69

Prepared according to General Procedure B and obtained as colorless oil. **Yield** 82%, 29.5 mg. Purification is through preparatory thin-layer chromatography (with eluent of Hex: EA = 10:1) to give the corresponding dichlorination products.

^{1}H NMR (600 MHz, CDCl$_3$) δ 7.39 (t, *J* = 2.1 Hz, 1H), 7.17 (dt, *J* = 8.9, 2.1 Hz, 1H), 6.78 (dd, *J* = 8.8, 1.6 Hz, 1H), 4.70 (s, 2H), 4.28 – 4.19 (m, 2H), 4.02 (ddt, *J* = 12.4, 7.9, 3.8 Hz, 1H), 3.76 (ddd, *J* = 11.5, 4.9, 1.6 Hz, 1H), 3.65 – 3.52 (m, 1H), 2.09-2.00 (m, 1H), 1.99-1.88 (m, 1H), 1.84 – 1.76 (m, 1H), 1.76 – 1.68 (m, 1H).

^{13}C NMR (151 MHz, CDCl$_3$) δ 168.10, 152.36, 130.43, 127.60, 127.18, 124.27, 114.62, 66.34, 64.68, 60.23, 47.88, 31.51, 25.09.

HRMS APCI: $[M+H]^+$ calcd. for $C_{13}H_{15}Cl_4O_3$: 358.9770; Found 358.9768

4,5-dichloropentyl 2-(4-chlorophenoxy)-2-methylpropanoate

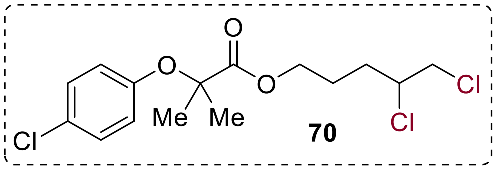
70

Prepared according to General Procedure B and obtained as colorless oil. **Yield** 79%, 27.9 mg. Purification is through preparatory thin-layer chromatography (with eluent of Hex: EA = 10:1) to give the corresponding dichlorination products.

^{1}H NMR (600 MHz, CDCl$_3$) δ 7.20 (d, *J* = 9.0 Hz, 2H), 6.77 (d, *J* = 8.9 Hz, 2H), 4.24 – 4.16 (m, 2H), 4.01-3.92 (m, 1H), 3.71 (dd, *J* = 11.3, 5.0 Hz, 1H), 3.55 (dd, *J* = 11.3, 7.8 Hz, 1H), 1.97 – 1.88 (m, 2H), 1.79-1.69 (m, 1H), 1.65-1.58 (m, 7H).

^{13}C NMR (151 MHz, CDCl$_3$) δ 173.96, 154.10, 129.20, 127.16, 120.14, 79.45, 64.60, 60.27, 47.87, 31.48, 25.38, 25.35, 25.08.

HRMS APCI: [M+H]$^+$ calcd. for $C_{13}H_{20}Cl_3O_3$: 353.0473; Found 353.0468

(2*S*,4*R*)-1,7,7-trimethylbicyclo[2.2.1]heptan-2-yl 4,5-dichloropentanoate

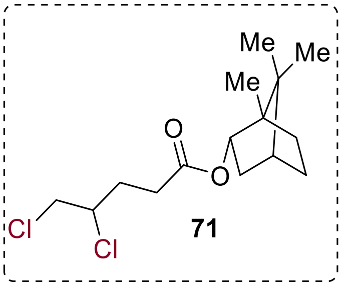
71

Prepared according to General Procedure B and obtained as colorless oil. **Yield** 69%, 21.2 mg. Purification is through preparatory column chromatography (with eluent of Hex: EA = 10:1) to give the corresponding dichlorination products.

^{1}H NMR (600 MHz, CDCl$_3$) δ 4.91 (dq, *J* = 8.9, 3.1 Hz, 1H), 4.18 – 4.08 (m, 1H), 3.81 (dd, *J* = 11.4, 5.0 Hz, 1H), 3.67 (dd, *J* = 11.4, 7.6 Hz, 1H), 2.65 – 2.51 (m, 2H), 2.48 – 2.33 (m, 2H), 2.05 – 1.90 (m, 2H), 1.80-1.71 (m, 1H), 1.71-1.66 (m, 1H), 1.35-1.28 (m, 1H), 1.24-1.19 (m, 1H), 0.97 (dd, *J* = 13.8, 3.5 Hz, 1H), 0.91 (s, 3H), 0.88 (s, 3H), 0.84 (s, 3H).

^{13}C NMR (151 MHz, CDCl$_3$) δ 172.76, 80.34, 80.31, 60.08, 60.06, 48.75, 48.02, 47.81, 44.82, 36.79, 36.74, 30.91, 30.40, 30.37, 28.01, 27.09, 19.69, 18.82, 13.55, 13.54.

HRMS ESI: [M+H]$^+$ calcd. for $C_{15}H_{25}Cl_2O_2$: 307.1226; Found 307.1667

5-(1,2-dichloroethyl)-3-(3,5-dichlorophenyl)-5-methyloxazolidine-2,4-dione

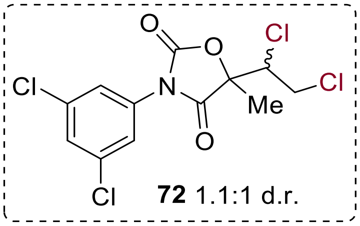
72 1.1:1 d.r.

Prepared according to General Procedure B and obtained as colorless oil in the mixture of two isomers. **Yield** 86%, 30.7 mg. d.r. = 1.1:1. Purification is through preparatory thin-layer chromatography (with eluent of Hex: EA = 3:1) to give the corresponding dichlorination products.

^{1}H NMR (600 MHz, CDCl$_3$) δ 7.63 – 7.28 (m, 3H), 4.50 – 4.43 (m, 1H), 4.08 (dd, *J* = 12.4, 4.7 Hz, 0.50H), 4.02 (dd, *J* = 12.2, 6.8 Hz, 0.55H), 3.93 (dd, *J* = 12.2, 7.0 Hz, 0.56H), 3.83 (dd, *J* = 12.4, 7.6 Hz, 0.50H), 1.84 (d, *J* = 16.0 Hz, 3H).

^{13}C NMR (151 MHz, CDCl$_3$) δ 170.71, 170.69, 151.79, 151.63, 135.73, 132.20, 132.13, 129.46, 129.44, 123.89, 123.85, 85.53, 84.79, 62.96, 62.45, 43.18, 42.77, 22.04, 20.21.

HRMS APCI: [M+H]$^+$ calcd. for $C_{12}H_{10}Cl_4NO_3$: 355.9409; Found 355.9405

(1*R*,2*S*,5*R*)-2-isopropyl-5-methylcyclohexyl 4,5-dichloropentanoate

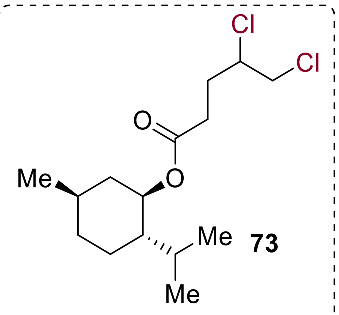

73

Prepared according to General Procedure B and obtained as colorless oil. **Yield** 50%, 15.5 mg. Purification is through preparatory column chromatography (with eluent of Hex: EA = 10:1) to give the corresponding dichlorination products.

^{1}H NMR (600 MHz, $CDCl_3$) δ 4.76-4.66 (m, 1H), 4.17-4.08 (m, 1H), 3.79 (ddd, *J* = 11.4, 5.0, 1.5 Hz, 1H), 3.66 (dd, *J* = 11.4, 7.5 Hz, 1H), 2.61 – 2.46 (m, 2H), 2.43 – 2.36 (m, 1H), 2.02 – 1.92 (m, 2H), 1.89-1.80 (m, 1H), 1.74-1.63 (m, 2H), 1.52-1.44 (m, 1H), 1.41-1.34 (m, 1H), 1.11 – 0.97 (m, 2H), 0.95 – 0.82 (m, 7H), 0.76 (dd, *J* = 7.0, 2.0 Hz, 3H).

^{13}C NMR (151 MHz, $CDCl_3$) δ 172.01 (d, *J* = 3.3 Hz), 74.61, 60.10 (d, *J* = 7.4 Hz), 48.06, 46.99 (d, *J* = 2.5 Hz), 40.91 (d, *J* = 2.8 Hz), 34.22, 31.40, 30.95, 30.43 (d, *J* = 6.0 Hz), 26.32 (d, *J* = 6.2 Hz), 23.43 (d, *J* = 6.8 Hz), 22.02, 20.76 (d, *J* = 3.2 Hz), 16.33 (d, *J* = 4.6 Hz).

HRMS APCI: $[M+Na]^+$ calcd. for $C_{15}H_{26}Cl_2NaO_2$: 331.1202; Found 331.1198

(8*R*,9*S*,13*S*,14*S*)-13-methyl-17-oxo-7,8,9,11,12,13,14,15,16,17-decahydro-6*H*-cyclopenta[*a*]phenanthren-3-yl 4,5-dichloropentanoate

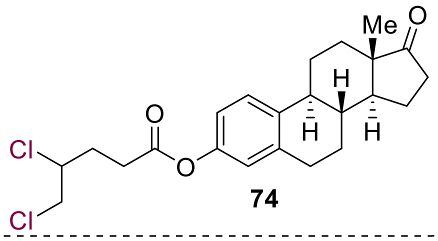

74

Prepared according to General Procedure B and obtained as white solid. **Yield** 56%, 23.7 mg. Purification is through preparatory thin-layer chromatography (with eluent of Hex: EA = 5:1) to give the corresponding dichlorination products.

^{1}H NMR (600 MHz, $CDCl_3$) δ 7.32-7.27 (m, 1H), 6.89 – 6.80 (m, 2H), 4.25-4.17 (m, 1H), 3.83 (dd, *J* = 11.4, 4.9 Hz, 1H), 3.70 (dd, *J* = 11.4, 7.7 Hz, 1H), 2.95 – 2.89 (m, 2H), 2.89-2.81 (m, 1H), 2.81-2.74 (m, 1H), 2.55 – 2.47 (m, 2H), 2.46 – 2.38 (m, 1H), 2.33-2.26 (td, *J* = 10.9, 4.3 Hz, 1H), 2.19 – 1.94 (m, 5H), 1.68 – 1.62 (m, 2H), 1.58 – 1.41 (m, 4H), 0.91 (s, 3H).

^{13}C NMR (151 MHz, $CDCl_3$) δ 171.38, 148.37, 138.12, 137.57, 126.50, 121.50, 118.66, 59.86, 50.41, 47.97, 44.15, 37.98, 35.89, 31.54, 30.74, 30.26, 29.43, 26.33, 25.76, 21.61, 13.85.

HRMS ESI: $[M+H]^+$ calcd. for $C_{23}H_{29}Cl_2O_3$: 423.1488; Found 423.1429

1-chloro-2-fluorododecane

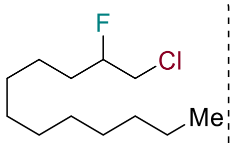

75

Prepared according to General Procedure C and obtained as colorless oil. **Yield** 63%, 16.7 mg. Yield is referred to equivalent of olefin. Purification is through flash column chromatography (with eluent of Hexane) to give the corresponding fluorochlorination products.

^{1}H NMR (600 MHz, $CDCl_3$) δ 4.72-4.57 (m, 1H), 3.69 – 3.55 (m, 2H), 1.79 – 1.61 (m, 2H), 1.50 – 1.42 (m, 1H), 1.42 – 1.25 (m, 15H), 0.88 (t, *J* = 7.0 Hz, 3H).

^{13}C NMR (151 MHz, CDCl$_3$) δ 92.50 (d, *J* = 174.5 Hz), 45.87 (d, *J* = 25.4 Hz), 32.40 (d, *J* = 20.5 Hz), 31.90, 29.58, 29.52, 29.42, 29.32, 29.30, 24.69 (d, *J* = 4.1 Hz), 22.69, 14.14.
^{19}F NMR (565 MHz, CDCl$_3$) δ -172.53 – -189.11 (m).
HRMS ESI: [M+Na]$^+$ calcd. for $C_{12}H_{24}ClFNa$: 245.1443; Found 245.2320

(4-chloro-3-fluorobutyl)benzene

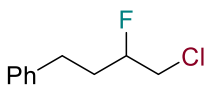

76

Prepared according to General Procedure C and obtained as colorless oil. **Yield** 45%, 10.2 mg. Yield is referred to equivalent of olefin. Purification is through preparatory thin-layer chromatography (with eluent of Hexane) to give the corresponding fluorochlorination products.
^{1}H NMR (600 MHz, CDCl$_3$) δ 7.31 (t, *J* = 7.6 Hz, 2H), 7.24 – 7.16 (m, 3H), 4.71 – 4.58 (m, 1H), 3.66-3.59 (m, 2H), 2.90-2.79 (m, 1H), 2.79-2.68 (m, 1H), 2.15 – 2.06 (m, 1H), 2.03 – 1.89 (m, 1H).
^{13}C NMR (151 MHz, CDCl$_3$) δ 140.59, 128.57, 128.45, 126.25, 91.35 (d, *J* = 175.4 Hz), 45.76 (d, *J* = 25.4 Hz), 34.11 (d, *J* = 20.7 Hz), 30.85 (d, *J* = 4.3 Hz).
^{19}F NMR (565 MHz, CDCl$_3$) δ -174.72 – -189.52 (m).
GC-MS: [M]$^+$ calcd. for $C_{10}H_{12}ClF$: 186.0612; Found 186

5-chloro-4-fluoropentyl benzoate

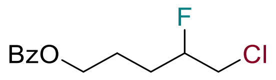

77

Prepared according to General Procedure C and obtained as colorless oil. **Yield** 58%, 17.1 mg. Yield is referred to equivalent of olefin. Purification is through preparatory thin-layer chromatography (with eluent of Hex: EA = 10:1) to give the corresponding fluorochlorination products.
^{1}H NMR (600 MHz, CDCl$_3$) δ 8.08 – 7.99 (m, 2H), 7.61 – 7.53 (m, 1H), 7.45 (t, *J* = 7.8 Hz, 2H), 4.81 – 4.67 (m, 1H), 4.44 – 4.32 (m, 2H), 3.71 – 3.60 (m, 2H), 2.06-1.96 (m, 1H), 1.96 – 1.83 (m, 3H).
^{13}C NMR (151 MHz, CDCl$_3$) δ 166.54, 133.04, 130.09, 129.55, 128.41, 91.88 (d, *J* = 175.5 Hz), 64.20, 45.52 (d, *J* = 25.6 Hz), 29.16 (d, *J* = 20.8 Hz), 24.24 (d, *J* = 4.1 Hz).
^{19}F NMR (565 MHz, CDCl$_3$) δ -182.39 – -182.93 (m).
HRMS APCI: [M+H]$^+$ calcd. for $C_{12}H_{15}ClFO_2$: 245.0739; Found 245.0738

5-chloro-4-fluoropentyl 4-chlorobenzoate

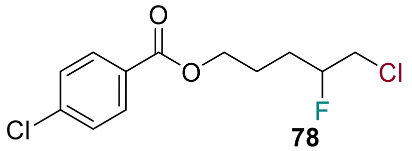

78

Prepared according to General Procedure C and obtained as colorless oil. **Yield** 55%, 18.4 mg. Yield is referred to equivalent of olefin. Purification is through preparatory thin-layer chromatography (with eluent of Hex: EA = 10:1) to give the corresponding fluorochlorination products.
^{1}H NMR (600 MHz, CDCl$_3$) δ 8.00 – 7.93 (m, 2H), 7.46 – 7.39 (m, 2H), 4.81 – 4.65 (m, 1H), 4.43 – 4.32 (m, 2H), 3.66 (dd, *J* = 19.1, 5.0 Hz, 2H), 2.05-1.96 (m, 1H), 1.96 – 1.81 (m, 3H).
^{13}C NMR (151 MHz, CDCl$_3$) δ 165.67, 139.48, 130.95, 128.77, 128.53, 91.83 (d, *J* = 175.7 Hz), 64.46, 45.46 (d, *J* = 25.9 Hz), 29.10 (d, *J* = 21.1 Hz), 24.20 (d, *J* = 4.1 Hz).
^{19}F NMR (565 MHz, CDCl$_3$) δ -179.52 – -186.92 (m).
HRMS APCI: [M+H]$^+$ calcd. for $C_{12}H_{14}Cl_2FO_2$: 279.0349; Found 279.0349

5-chloro-4-fluoropentyl 4-ethylbenzoate

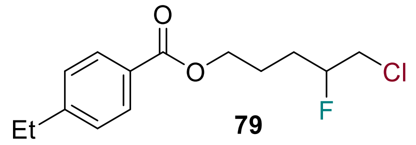

79

Prepared according to General Procedure C and obtained as colorless oil. **Yield** 48%, 15.8 mg. Yield is referred to equivalent of olefin. Purification is through preparatory thin-layer chromatography (with eluent of Hex: EA = 10:1) to give the corresponding fluorochlorination products.

^{1}H NMR (600 MHz, CDCl$_3$) δ 7.99 – 7.90 (m, 2H), 7.30-7.24 (m, 2H), 4.80 – 4.66 (m, 1H), 4.42 – 4.29 (m, 2H), 3.70 – 3.59 (m, 2H), 2.71 (q, *J* = 7.6 Hz, 2H), 2.03-1.95 (m, 1H), 1.94 – 1.81 (m, 3H), 1.26 (t, *J* = 7.6 Hz, 4H).

^{13}C NMR (151 MHz, CDCl$_3$) δ 166.62, 149.92, 129.68, 127.94, 127.56, 91.90 (d, *J* = 175.5 Hz), 64.00, 45.54 (d, *J* = 25.6 Hz), 29.17 (d, *J* = 21.1 Hz), 28.96, 24.26 (d, *J* = 4.2 Hz), 15.27.

^{19}F NMR (565 MHz, CDCl$_3$) δ -177.12 – -190.41 (m).

HRMS APCI: $[M+H]^+$ calcd. for $C_{14}H_{19}ClFO_2$: 273.1052; Found 273.1050

5-chloro-4-fluoropentyl 4-methylbenzenesulfonate

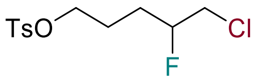

80

Prepared according to General Procedure C and obtained as colorless oil. **Yield** 59%, 20.9 mg. Yield is referred to equivalent of olefin. Purification is through preparatory thin-layer chromatography (with eluent of Hex: EA = 6:1) to give the corresponding fluorochlorination products.

^{1}H NMR (600 MHz, CDCl$_3$) δ 7.79 (d, *J* = 8.3 Hz, 2H), 7.36 (d, *J* = 8.1 Hz, 2H), 4.68-4.50 (m, 1H), 4.14-4.02 (m, 2H), 3.58 (dd, *J* = 19.3, 5.0 Hz, 2H), 2.46 (s, 3H), 1.92 – 1.83 (m, 1H), 1.82 – 1.71 (m, 3H).

^{13}C NMR (151 MHz, CDCl$_3$) δ 144.97, 132.81, 129.93, 127.89, 91.52 (d, *J* = 175.6 Hz), 69.64, 45.41 (d, *J* = 25.4 Hz), 28.50 (d, *J* = 20.9 Hz), 24.42 (d, *J* = 3.8 Hz), 21.67.

^{19}F NMR (565 MHz, CDCl$_3$) δ -176.71 – -191.51 (m).

HRMS APCI: $[M+H]^+$ calcd. for $C_{12}H_{17}ClFO_3S$: 295.0565; Found 295.0565

***N*-(4-chloro-3-fluorobutyl)-*N*-methylbenzenesulfonamide**

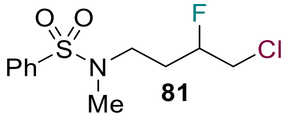

81

Prepared according to General Procedure C and obtained as colorless oil. **Yield** 58%, 19.2 mg. Yield is referred to equivalent of olefin. Purification is through preparatory thin-layer chromatography (with eluent of Hex: EA = 5:1) to give the corresponding fluorochlorination products.

^{1}H NMR (600 MHz, CDCl$_3$) δ 7.84 – 7.77 (m, 2H), 7.65 – 7.58 (m, 1H), 7.58-7.51 (m, 2H), 4.94 – 4.77 (m, 1H), 3.77 – 3.66 (m, 2H), 3.20 (dt, *J* = 14.2, 7.2 Hz, 1H), 3.12 (dt, *J* = 13.4, 6.4 Hz, 1H), 2.78 (s, 3H), 2.10 – 1.96 (m, 2H).

^{13}C NMR (151 MHz, CDCl$_3$) δ 136.92, 132.84, 129.20, 127.38, 89.52 (d, *J* = 175.1 Hz), 46.28 (d, *J* = 5.0 Hz), 45.54 (d, *J* = 24.6 Hz), 35.48, 30.83 (d, *J* = 21.0 Hz).

^{19}F NMR (565 MHz, CDCl$_3$) δ -184.27 (dddt, *J* = 46.4, 28.0, 22.1, 18.1 Hz).

HRMS APCI: $[M+H]^+$ calcd. for $C_{11}H_{16}ClFNO_2S$: 280.0569; Found 280.0568

***N*-(4-chloro-3-fluorobutyl)benzenesulfonamide**

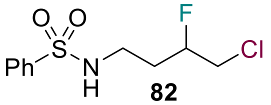

82

Prepared according to General Procedure C and obtained as colorless oil. **Yield** 53%, 17.0 mg. Yield is referred to equivalent of olefin. Purification is through preparatory thin-layer chromatography (with eluent of Hex: EA = 3:1) to give the corresponding fluorochlorination products.

^{1}H NMR (600 MHz, CDCl$_3$) δ 7.91 – 7.83 (m, 2H), 7.64 – 7.58 (m, 1H), 7.57 – 7.52 (m, 2H), 4.85 (t, J = 6.3 Hz, 1H), 4.83 – 4.69 (m, 1H), 3.71 – 3.52 (m, 2H), 3.25 – 3.08 (m, 2H), 1.99 – 1.85 (m, 2H).
^{13}C NMR (151 MHz, CDCl$_3$) δ 139.49, 132.92, 129.28, 127.02, 90.13 (d, J = 174.9 Hz), 45.32 (d, J = 25.0 Hz), 39.31 (d, J = 3.9 Hz), 32.41 (d, J = 20.4 Hz).
^{19}F NMR (565 MHz, CDCl$_3$) δ -174.72 – -189.79 (m).
HRMS APCI: $[M+H]^+$ calcd. for $C_{10}H_{14}ClFNO_2S$: 266.0412; Found 266.0410

2-(4-chloro-3-fluorobutyl)isoindoline-1,3-dione

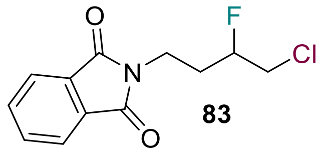

83

Prepared according to General Procedure C and obtained as white solid. **Yield** 57%, 17.3 mg. Yield is referred to equivalent of olefin. Purification is through preparatory thin-layer chromatography (with eluent of Hex: EA = 5:1) to give the corresponding fluorochlorination products.
^{1}H NMR (600 MHz, CDCl$_3$) δ 7.86 (dd, J = 5.4, 3.1 Hz, 2H), 7.74 (dd, J = 5.4, 3.1 Hz, 2H), 4.76 (ddq, J = 47.6, 8.8, 4.5 Hz, 1H), 3.89 (t, J = 6.9 Hz, 2H), 3.68 (dd, J = 19.3, 5.0 Hz, 2H), 2.22 – 2.01 (m, 2H).
^{13}C NMR (151 MHz, CDCl$_3$) δ 168.25, 134.14, 131.98, 123.40, 90.30 (d, J = 176.0 Hz), 45.31 (d, J = 25.4 Hz), 34.11 (d, J = 4.4 Hz), 31.34 (d, J = 20.5 Hz).
^{19}F NMR (565 MHz, CDCl$_3$*d*) δ -176.23 – -190.00 (m).
HRMS APCI: $[M]^-$ calcd. for $C_{12}H_{11}ClFNO_2$: 255.0457; Found 255.0465

diethyl 2-(3-chloro-2-fluoropropyl)malonate

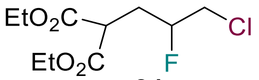

84

Prepared according to General Procedure C and obtained as colorless oil. **Yield** 48%, 14.7 mg. Yield is referred to equivalent of olefin. Purification is through flash column chromatography (with eluent of Hex: EA = 7:1) to give the corresponding fluorochlorination products.
^{1}H NMR (600 MHz, CDCl$_3$) δ 4.84 – 4.68 (m, 1H), 4.29 – 4.18 (m, 4H), 3.72 – 3.57 (m, 3H), 2.37 – 2.26 (m, 2H), 1.34-1.26 (m, 6H).
^{13}C NMR (151 MHz, CDCl$_3$) δ 168.79, 168.63, 90.01 (d, J = 176.4 Hz), 61.85, 47.80, 47.78, 45.43 (d, J = 24.8 Hz), 31.67 (d, J = 20.3 Hz), 14.02 (d, J = 4.3 Hz).
^{19}F NMR (565 MHz, CDCl$_3$) δ -177.32 – -190.00 (m).
HRMS APCI: $[M+H]^+$ calcd. for $C_{10}H_{17}ClFO_4$: 255.0794; Found 255.0792

11-chloro-10-fluoroundecanal

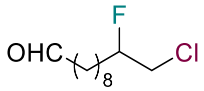

85

Prepared according to General Procedure C and obtained as colorless oil. **Yield** 50%, 13.3 mg. Yield is referred to equivalent of olefin. Purification is through flash column chromatography (with eluent of Hex: EA = 10:1) to give the corresponding fluorochlorination products.
^{1}H NMR (600 MHz, CDCl$_3$) δ 9.88 – 9.64 (m, 1H), 4.72 – 4.56 (m, 1H), 3.70 – 3.54 (m, 2H), 2.49-2.38 (m, 2H), 1.78 – 1.61 (m, 4H), 1.49 – 1.31 (m, 10H).
^{13}C NMR (151 MHz, CDCl$_3$) δ 202.85, 92.44 (d, J = 174.8 Hz), 45.80 (d, J = 25.5 Hz), 43.89, 32.38 (d, J = 20.4 Hz), 29.20, 29.17, 29.09, 24.65 (d, J = 4.4 Hz), 22.03.
^{19}F NMR (565 MHz, CDCl$_3$) δ -170.34 – -186.44 (m).
GC-MS: $[M-H2O]^+$ calcd. for C10H18ClF: 204.1081; Found 204

11-chloro-10-fluoroundecanoic acid

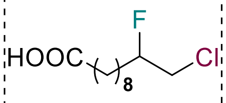
86

Prepared according to General Procedure C and obtained as colorless oil. **Yield** 54%, 15.5 mg. Yield is referred to equivalent of olefin. Purification is through flash column chromatography (with eluent of Hex: EA: AcOH = 3:1:0.1) to give the corresponding fluorochlorination products.

^{1}H NMR (600 MHz, $CDCl_3$) δ 4.71 – 4.55 (m, 1H), 3.68 – 3.54 (m, 2H), 2.35 (t, *J* = 7.5 Hz, 2H), 1.78 – 1.61 (m, 4H), 1.51-1.42 (m, 1H), 1.42 – 1.30 (m, 9H).

^{13}C NMR (151 MHz, $CDCl_3$) δ 179.70, 92.48 (d, *J* = 174.5 Hz), 45.86 (d, *J* = 25.5 Hz), 33.96, 32.39 (d, *J* = 20.5 Hz), 29.22, 29.19, 29.10, 28.98, 24.67 (d, *J* = 4.3 Hz), 24.63.

^{19}F NMR (565 MHz, $CDCl_3$) δ -172.73 – -192.40 (m).

HRMS APCI: $[M-H]^-$ calcd. for $C_{11}H_{19}ClFO_2$: 237.1052; Found 237.1060

(3-chloro-2-fluoro-2-methylpropyl)benzene

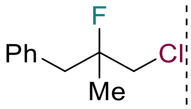
87

Prepared according to General Procedure C and obtained as colorless oil. **Yield** 56%, 12.5 mg. Yield is referred to equivalent of olefin. Purification is through preparatory thin-layer chromatography (with eluent of Hexane) to give the corresponding fluorochlorination products.

^{1}H NMR (600 MHz, $CDCl_3$) δ 7.34 – 7.25 (m, 5H), 3.49 (dd, *J* = 14.4, 1.1 Hz, 2H), 3.06 (d, *J* = 20.5 Hz, 2H), 1.42 (d, *J* = 21.5 Hz, 3H).

^{13}C NMR (151 MHz, $CDCl_3$) δ 135.36, 135.33, 130.36, 128.36, 127.00, 95.33 (d, *J* = 176.0 Hz), 48.80 (d, *J* = 30.3 Hz), 43.11 (d, *J* = 21.9 Hz), 22.88 (d, *J* = 23.5 Hz).

^{19}F NMR (565 MHz, $CDCl_3$) δ -130.53 – -164.24 (m).

HRMS ESI: $[M+K]^+$ calcd. for $C_{10}H_{12}ClFK$: 225.0243; Found 225.0693

2-chloro-3-fluoro-3-methylbutyl benzoate

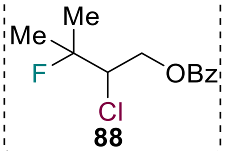
88

Prepared according to General Procedure C and obtained as colorless oil. **Yield** 53%, 15.4 mg. Yield is referred to equivalent of olefin. Purification is through preparatory thin-layer chromatography (with eluent of Hex: EA = 10:1) to give the corresponding fluorochlorination products.

^{1}H NMR (600 MHz, $CDCl_3$) δ 8.13 – 8.02 (m, 2H), 7.62 – 7.56 (m, 1H), 7.47 (t, *J* = 7.8 Hz, 2H), 4.78 (ddd, *J* = 12.0, 3.6, 0.8 Hz, 1H), 4.50 (dd, *J* = 11.9, 8.3 Hz, 1H), 4.24 (ddd, *J* = 9.5, 8.3, 3.6 Hz, 1H), 1.65 – 1.48 (m, 1H).

^{13}C NMR (151 MHz, $CDCl_3$) δ 166.21, 133.36, 129.77, 129.56, 128.51, 95.46 (d, *J* = 172.8 Hz), 64.93 (d, *J* = 4.7 Hz), 63.49 (d, *J* = 27.8 Hz), 25.49 (d, *J* = 23.9 Hz), 23.08 (d, *J* = 23.9 Hz).

^{19}F NMR (565 MHz, $CDCl_3$) δ -140.69 (pd, *J* = 22.3, 10.1 Hz).

HRMS ESI: $[M+K]^+$ calcd. for $C_{12}H_{14}ClFKO_2$: 283.0298; Found 283.2843

(2-chloro-1-fluoroethyl)benzene

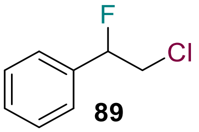

89 Prepared according to General Procedure C and obtained as colorless oil. **Yield** 33%, 6.3 mg. Yield is referred to equivalent of olefin. Purification is through preparatory thin-layer chromatography (with eluent of Hexane) to give the corresponding fluorochlorination products.
^{1}H NMR (600 MHz, $CDCl_3$) δ 7.47 – 7.34 (m, 5H), 5.60 (ddd, *J* = 47.1, 7.9, 3.8 Hz, 1H), 3.88 – 3.68 (m, 2H).
^{13}C NMR (151 MHz, $CDCl_3$) δ 136.57 (d, *J* = 20.1 Hz), 129.26 (d, *J* = 1.9 Hz), 128.72, 125.75 (d, *J* = 6.8 Hz), 93.07 (d, *J* = 178.2 Hz), 46.89 (d, *J* = 28.2 Hz).
^{19}F NMR (565 MHz, $CDCl_3$) δ -178.70 (ddd, *J* = 48.0, 26.4, 15.5 Hz).
The compound characterization was reported in literature.[15]

***N,N*-diallyl-4-methylbenzenesulfonamide--3,4-bis(azidomethyl)-1-tosylpyrrolidine**

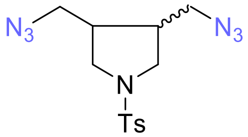

90 2.1:1 d.r. Prepared according to General Procedure A and obtained as colorless oil in the mixture of two isomers. **Yield** 67%, 22.4 mg. d.r. = 2.1:1.
^{1}H NMR (600 MHz, $CDCl_3$) δ 7.72 (dd, *J* = 13.2, 8.0 Hz, 2H), 7.36 (d, *J* = 7.9 Hz, 2H), 3.47-3.37 (m, 2H), 3.34-3.27 (m, 2H), 3.27-3.21 (m, 0.70H), 3.18-3.13 (m, 1.36H), 3.13-3.07 (m, 1.35H), 3.04-2.99 (m, 0.65H), 2.47-2.43 (m, 3H), 2.42-2.37 (m, 1.28H), 2.14-2.08 (m, 0.63H).
^{13}C NMR (151 MHz, $CDCl_3$) δ 143.94, 133.16, 132.46, 129.82, 127.71, 127.48, 52.77, 50.58, 50.25, 49.54, 41.13, 39.75, 21.59, 21.56.
The compound characterization was reported in literature.[6]

dibenzyl (*E*)-2-azido-2-(4-azidobut-2-en-1-yl)malonate

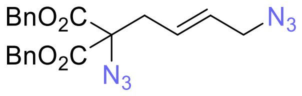

91 E/Z > 20:1 Prepared according to General Procedure A and obtained as colorless oil in single isomer. **Yield** 60%, 25.2 mg. E/Z >20:1.
^{1}H NMR (600 MHz, $CDCl_3$) δ 7.48 – 7.27 (m, 10H), 5.70 – 5.43 (m, 2H), 5.35 – 5.14 (m, 4H), 3.58 (d, *J* = 6.5 Hz, 2H), 2.70 (t, *J* = 6.0 Hz, 2H).
^{13}C NMR (151 MHz, $CDCl_3$) δ 166.57, 134.46, 128.81, 128.71, 128.59, 128.56, 127.53, 71.43, 68.48, 52.25, 36.89.
The compound characterization was reported in literature.[6]

1,2-diazido-3,3-dimethylbutane

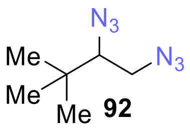

92 Prepared according to General Procedure A and obtained as colorless oil. **Yield** 64%, 6.7 mg.
^{1}H NMR (600 MHz, $CDCl_3$) δ 3.51 (dd, *J* = 12.2, 2.2 Hz, 1H), 3.26 (dd, *J* = 12.2, 10.2 Hz, 1H), 3.21 (dd, *J* = 10.2, 2.2 Hz, 1H), 0.96 (s, 9H).
^{13}C NMR (151 MHz, $CDCl_3$) δ 72.75, 52.45, 34.99, 26.43.
The compound characterization was reported in literature.[6]

N-(2,3-diazidopropyl)benzamide

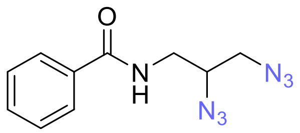

93

Prepared according to General Procedure A and obtained as colorless oil. **Yield** 58%, 14.2 mg.

^{1}H NMR (600 MHz, CDCl$_3$) δ 7.84 – 7.75 (m, 2H), 7.59 – 7.52 (m, 1H), 7.49 – 7.42 (m, 2H), 6.51 (t, J = 6.1 Hz, 1H), 3.92 (tt, J = 7.2, 4.2 Hz, 1H), 3.74 (ddd, J = 14.1, 6.4, 4.5 Hz, 1H), 3.59 (dd, J = 12.9, 4.0 Hz, 1H), 3.51 – 3.39 (m, 2H).

^{13}C NMR (151 MHz, CDCl$_3$) δ 167.89, 133.65, 132.00, 128.76, 126.96, 61.08, 52.90, 41.38.

HRMS APCI: $[M+H]^+$ calcd. for $C_{10}H_{12}N_7O$: 246.1098; Found 246.1096

(4-chloro-3-fluoro-2-methylbutan-2-yl)benzene

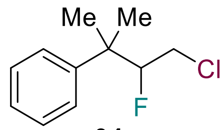

94

Prepared according to General Procedure C and obtained as colorless oil. **Yield** 47%, 9.4 mg.

^{1}H NMR (600 MHz, CDCl$_3$) δ 7.38 – 7.32 (m, 4H), 7.28 – 7.25 (m, 1H), 4.68 (ddd, J = 48.2, 7.5, 3.5 Hz, 1H), 3.40 – 3.30 (m, 2H), 1.44 (d, J = 1.7 Hz, 3H), 1.42 (d, J = 1.3 Hz, 3H).

^{13}C NMR (151 MHz, CDCl$_3$) δ 143.91 (d, J = 3.4 Hz), 128.61, 126.92, 126.27, 100.45, 99.83 (d, J = 185.1 Hz), 43.94 (d, J = 23.7 Hz), 41.85 (d, J = 18.7 Hz), 26.20 (d, J = 4.3 Hz), 22.49 (d, J = 4.7 Hz).

^{19}F NMR (565 MHz, CDCl$_3$) δ -189.10 – -189.62 (m).

GC-MS: $[M]^+$ calcd. for $C_{11}H_{14}ClF$: 200.0768; Found 200

N-(3-chloro-2-fluoropropyl)benzamide

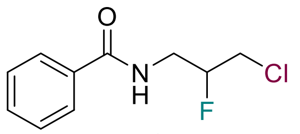

95

Prepared according to General Procedure C and obtained as colorless oil. **Yield** 54%, 11.6 mg.

^{1}H NMR (600 MHz, CDCl$_3$) δ 7.84 – 7.74 (m, 2H), 7.58 – 7.50 (m, 1H), 7.49-7.41 (m, 2H), 6.47 (s, 1H), 4.98 – 4.85 (m, 1H), 4.03-3.87 (m, 1H), 3.82 – 3.65 (m, 3H).

^{13}C NMR (151 MHz, CDCl$_3$) δ 167.82, 133.72, 131.97, 128.74, 126.99, 90.84 (d, J = 176.6 Hz), 43.53 (d, J = 24.2 Hz), 41.55 (d, J = 21.4 Hz).

^{19}F NMR (565 MHz, CDCl$_3$) δ -187.92 – -188.26 (m).

HRMS ESI: $[M+H]^+$ calcd. for $C_{21}H_{26}Cl_2FNO$: 216.0586; Found 216.0589

(1-azidoethyl)benzene

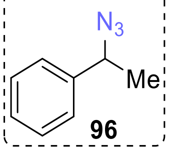

96

Prepared according to General Procedure A and obtained as colorless oil. **Yield** 25%, 3.6 mg. **^{1}H NMR (600 MHz, CDCl$_3$)** δ 7.45 – 7.29 (m, 5H), 4.62 (q, J = 6.8 Hz, 1H), 1.54 (d, J = 6.8 Hz, 3H).

^{13}C NMR (151 MHz, CDCl$_3$) δ 140.85, 128.78, 128.14, 126.38, 61.10, 21.59.

HRMS APCI: [M-N2+H]$^+$ calcd. for $C_8H_{10}N$: 120.0808; Found 120.0810

4,5-diazidopentyl 4-ethylbenzoate

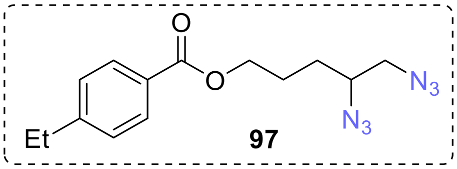

Prepared according to General Procedure A and obtained as colorless oil.

Yield 68%, 20.5 mg.

^{1}H NMR (600 MHz, CDCl$_3$) δ 7.99 – 7.90 (m, 2H), 7.30 – 7.24 (m, 2H), 4.41 – 4.29 (m, 2H), 3.55 (ddt, *J* = 8.8, 7.3, 4.4 Hz, 1H), 3.45 (dd, *J* = 12.7, 4.2 Hz, 1H), 3.38 (dd, *J* = 12.7, 7.3 Hz, 1H), 2.71 (q, *J* = 7.6 Hz, 2H), 2.01 – 1.92 (m, 1H), 1.92 – 1.83 (m, 1H), 1.78 – 1.62 (m, 2H), 1.26 (t, *J* = 7.6 Hz, 4H).

^{13}C NMR (151 MHz, CDCl$_3$) δ 166.60, 149.98, 129.69, 127.96, 127.49, 63.91, 61.61, 54.85, 28.96, 28.53, 25.32, 15.26.

The compound characterization was reported in literature.[6]

VIII. NMR Spectrum for New Compounds

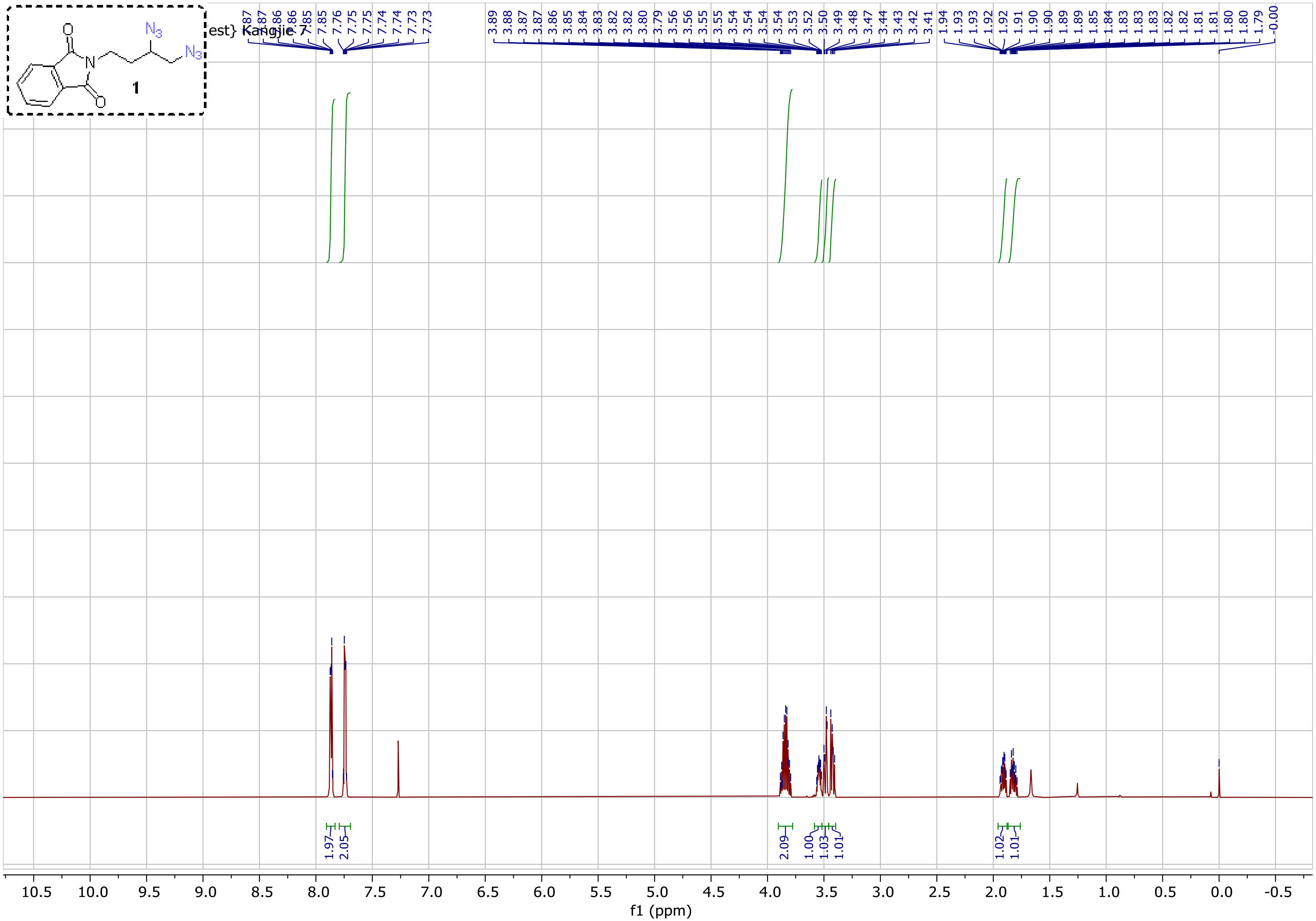
est} Kangjie 7
1
f1 (ppm)

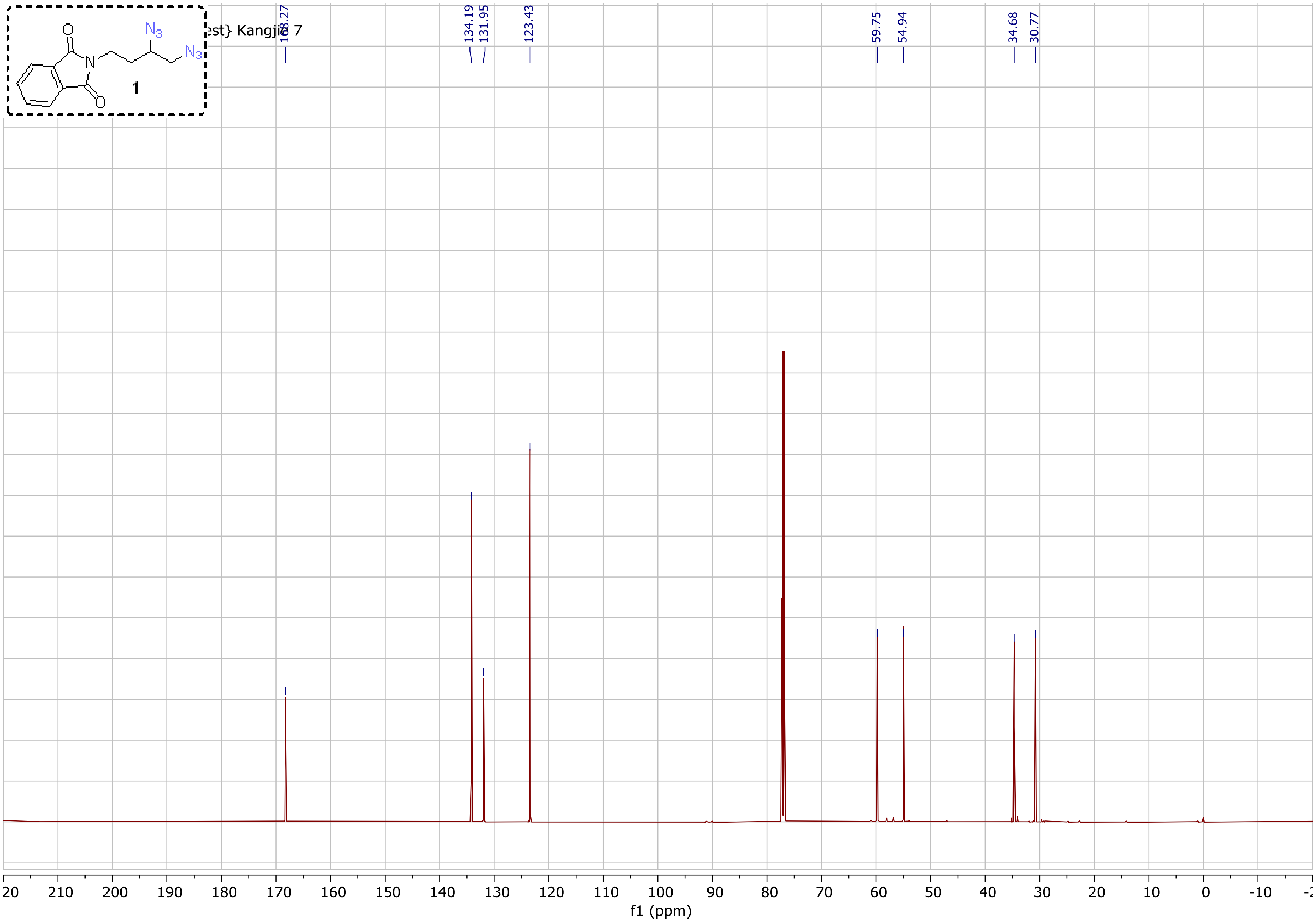

O
N_3
N
N_3
O
1
est} Kangjie 7
168.27
134.19
131.95
123.43
59.75
54.94
34.68
30.77
f1 (ppm)

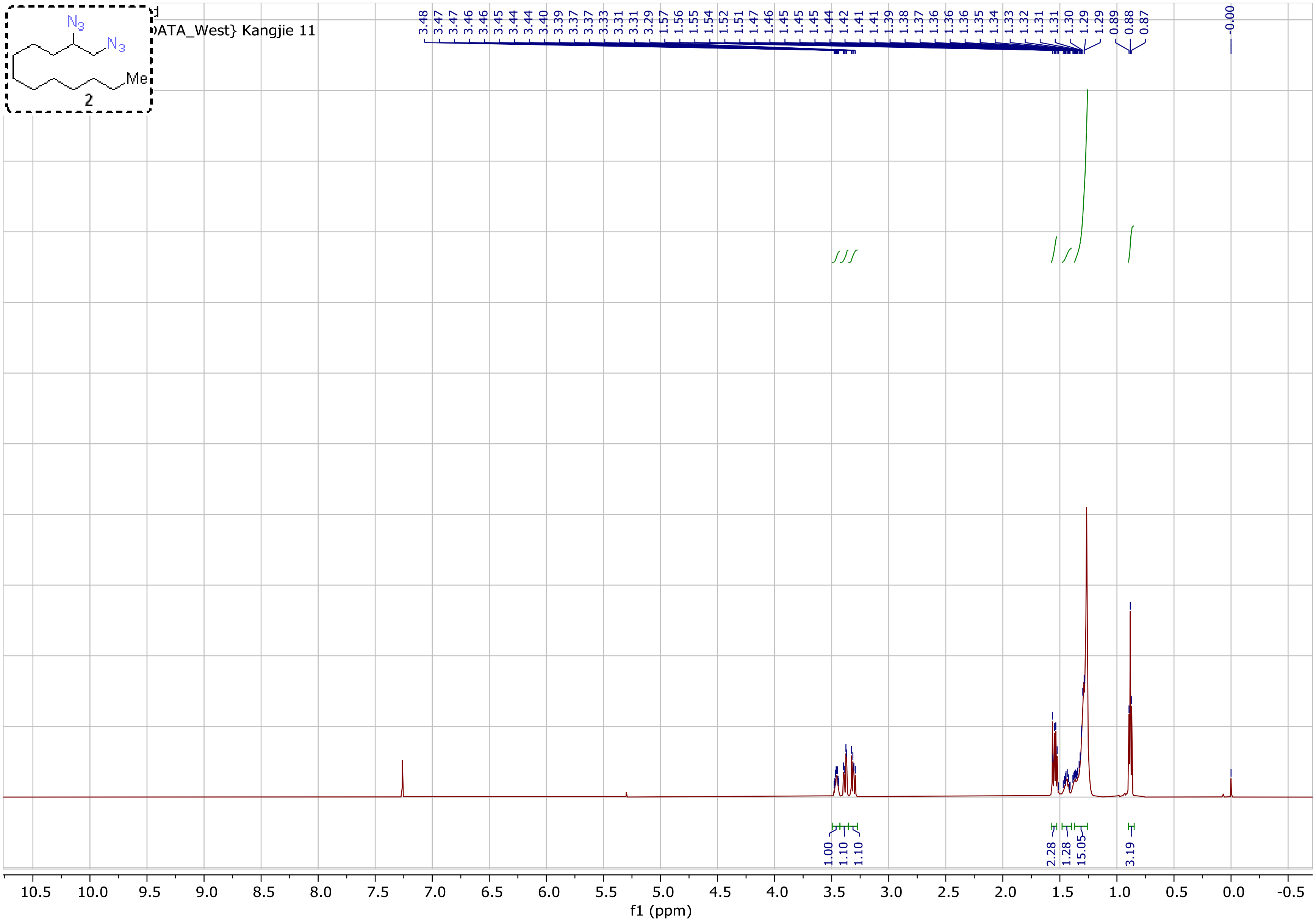
DATA_West} Kangjie 11
N3
N3
Me
2
3.48 3.47 3.47 3.46 3.45 3.44 3.44 3.40 3.39 3.37 3.37 3.33 3.31 3.31 3.29 1.57 1.56 1.55 1.54 1.52 1.51 1.47 1.46 1.45 1.45 1.45 1.44 1.42 1.41 1.41 1.39 1.38 1.37 1.36 1.36 1.36 1.35 1.34 1.33 1.32 1.31 1.31 1.30 1.29 1.29 0.89 0.88 0.87
-0.00
1.00 1.10 1.10
2.28 1.28 15.05
3.19
10.5 10.0 9.5 9.0 8.5 8.0 7.5 7.0 6.5 6.0 5.5 5.0 4.5 4.0 3.5 3.0 2.5 2.0 1.5 1.0 0.5 0.0 -0.5
f1 (ppm)

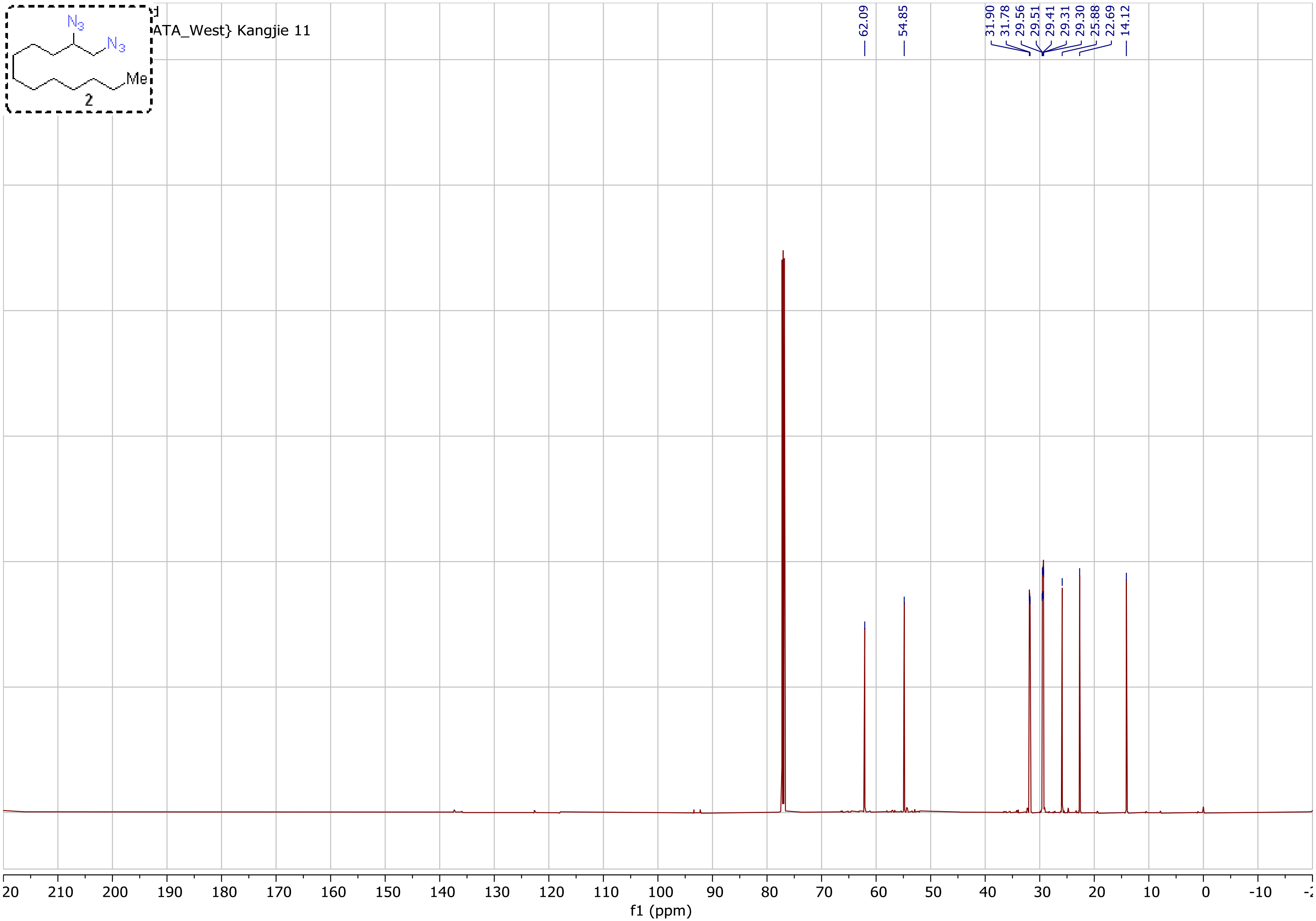

d
ATA_West} Kangjie 11
N3
N3
Me
2
62.09
54.85
31.90
31.78
29.56
29.51
29.41
29.31
29.30
25.88
22.69
14.12
f1 (ppm)

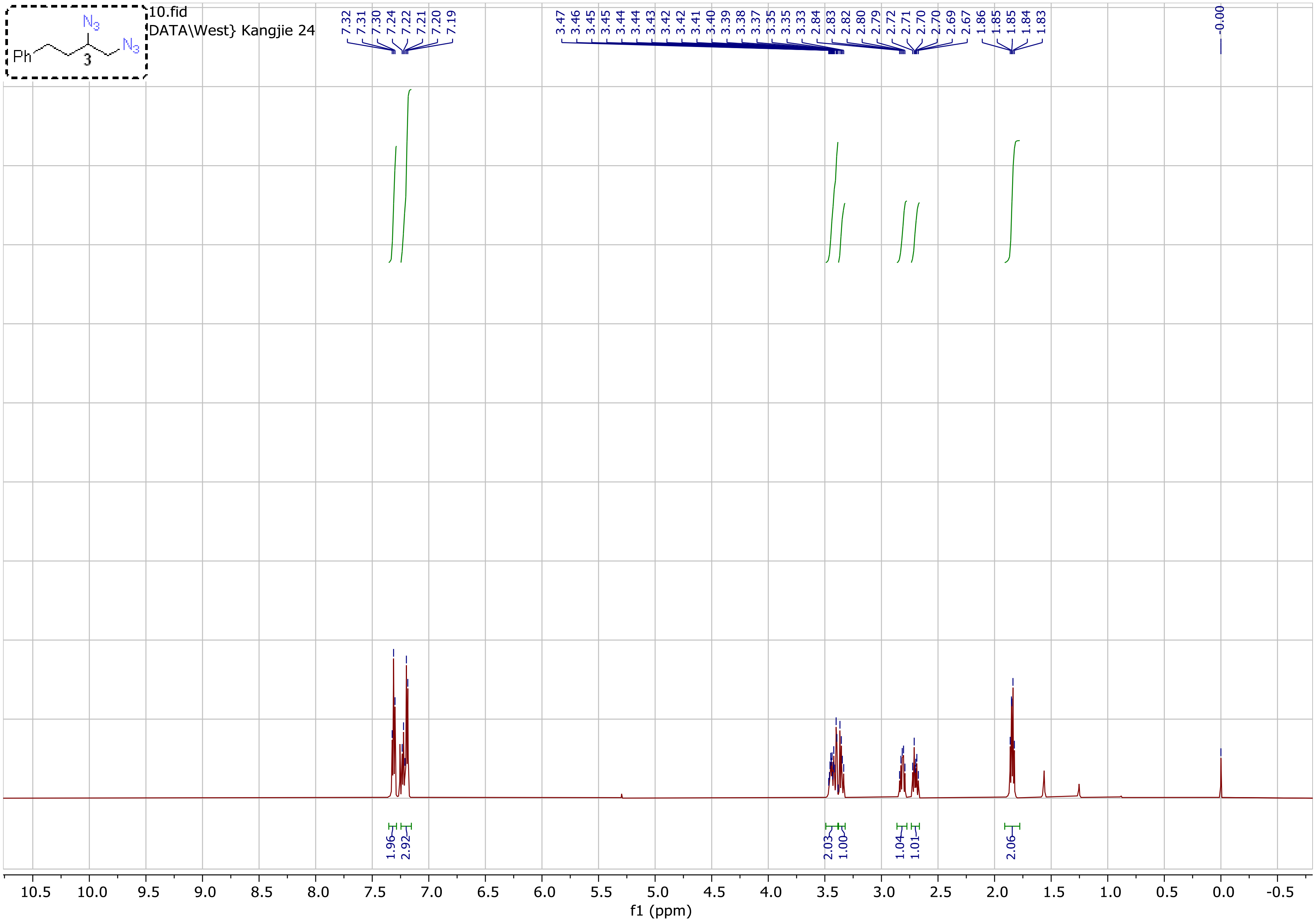

10.fid
DATA\West} Kangjie 24
Ph
N3
N3
3
7.32
7.31
7.30
7.24
7.22
7.21
7.20
7.19
3.47
3.46
3.45
3.45
3.44
3.44
3.43
3.42
3.42
3.41
3.40
3.39
3.38
3.37
3.35
3.35
3.33
2.84
2.83
2.82
2.80
2.79
2.72
2.71
2.70
2.70
2.69
2.67
1.86
1.85
1.85
1.84
1.83
-0.00
1.96
2.92
2.03
1.00
1.04
1.01
2.06
10.5
10.0
9.5
9.0
8.5
8.0
7.5
7.0
6.5
6.0
5.5
5.0
4.5
4.0
3.5
3.0
2.5
2.0
1.5
1.0
0.5
0.0
-0.5
f1 (ppm)

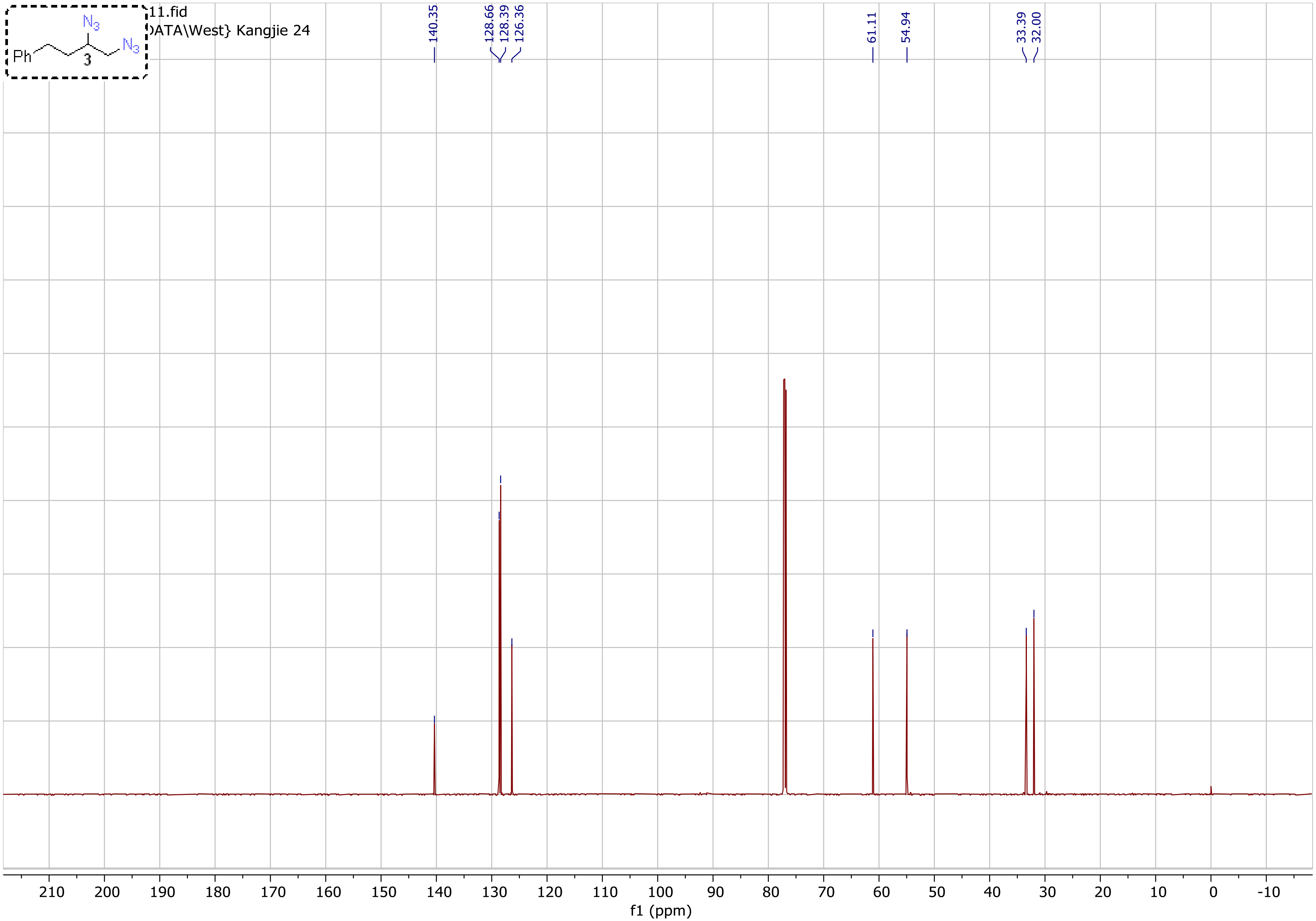

Ph
N3
N3
3
11.fid
DATA\West} Kangjie 24
140.35
128.66
128.39
126.36
61.11
54.94
33.39
32.00
210 200 190 180 170 160 150 140 130 120 110 100 90 80 70 60 50 40 30 20 10 0 -10
f1 (ppm)

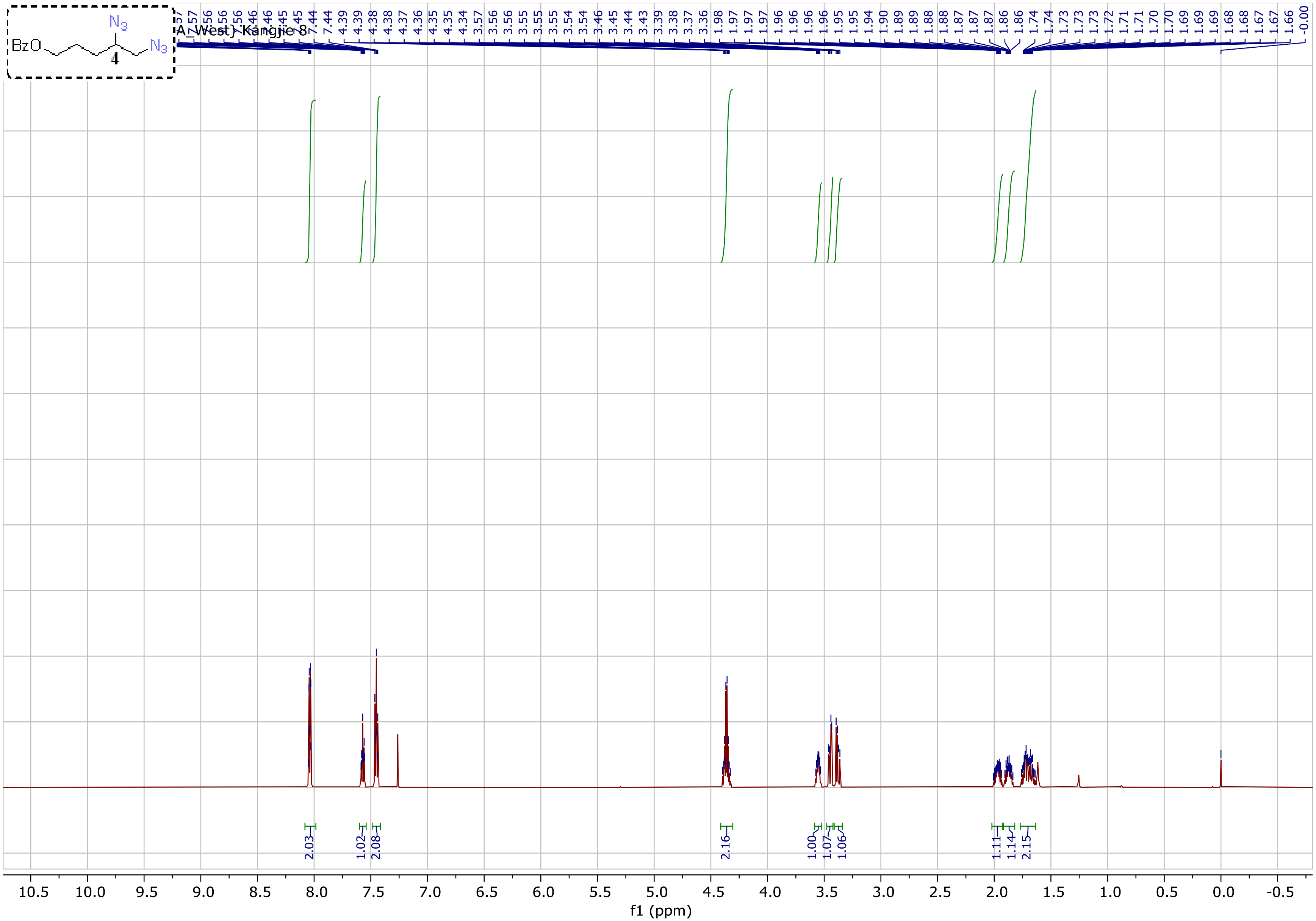

BzO
N3
N3
4
A_West} Kangjie 8
7.57 7.56 7.56 7.56 7.46 7.46 7.45 7.45 7.44 7.44 4.39 4.39 4.38 4.38 4.37 4.36 4.35 4.35 4.34 3.57 3.56 3.56 3.55 3.55 3.55 3.54 3.54 3.46 3.45 3.44 3.43 3.39 3.38 3.37 3.36 1.98 1.97 1.97 1.97 1.96 1.96 1.96 1.95 1.95 1.94 1.90 1.89 1.89 1.88 1.88 1.87 1.87 1.87 1.86 1.86 1.74 1.74 1.73 1.73 1.72 1.71 1.71 1.70 1.70 1.69 1.69 1.69 1.68 1.68 1.67 1.67 1.66 -0.00
2.03
1.02
2.08
2.16
1.00
1.07
1.06
1.11
1.14
2.15
10.5 10.0 9.5 9.0 8.5 8.0 7.5 7.0 6.5 6.0 5.5 5.0 4.5 4.0 3.5 3.0 2.5 2.0 1.5 1.0 0.5 0.0 -0.5
f1 (ppm)

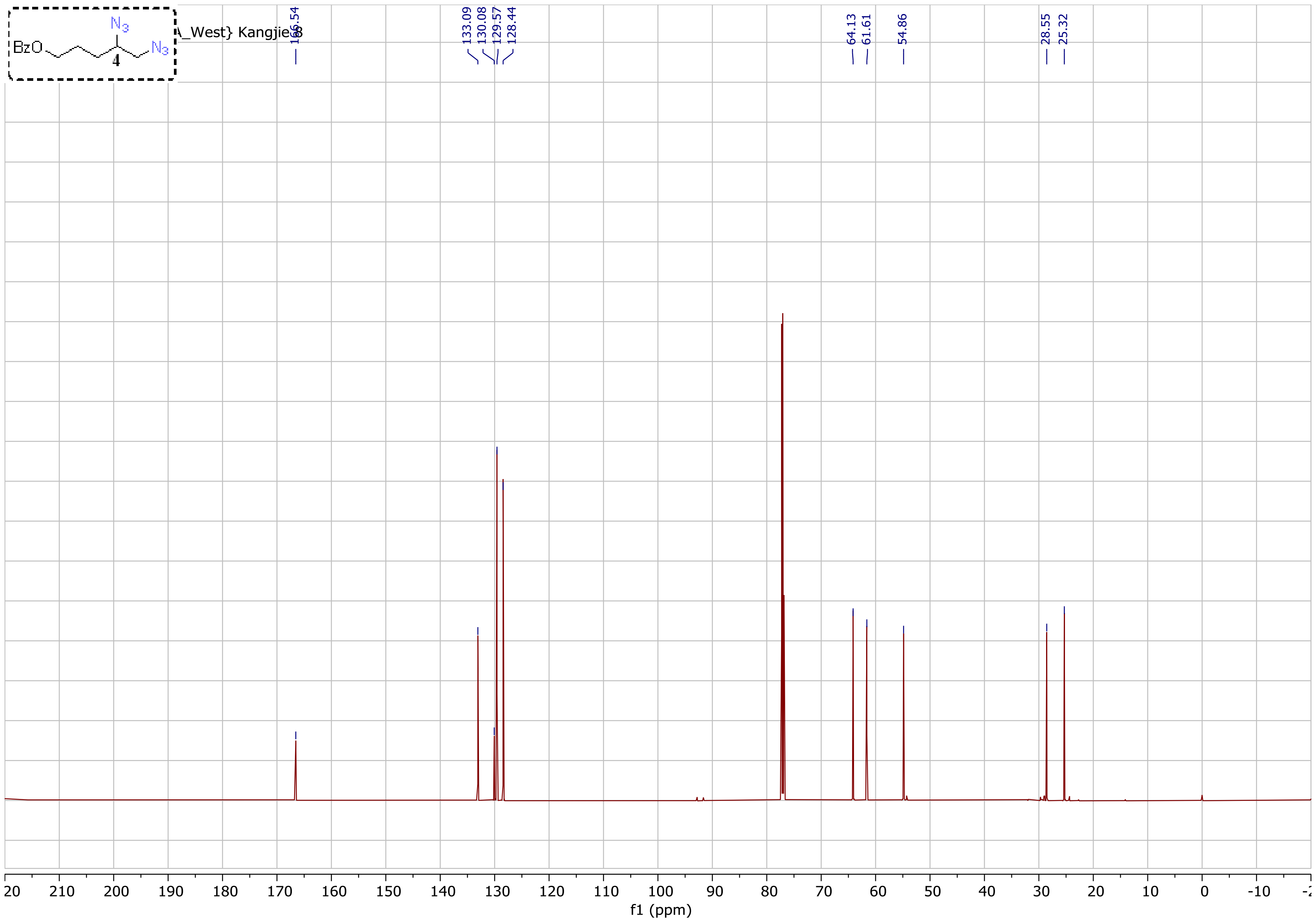
BzO
N3
N3
4
_West} Kangjie8
166.54
133.09
130.08
129.57
128.44
64.13
61.61
54.86
28.55
25.32
f1 (ppm)

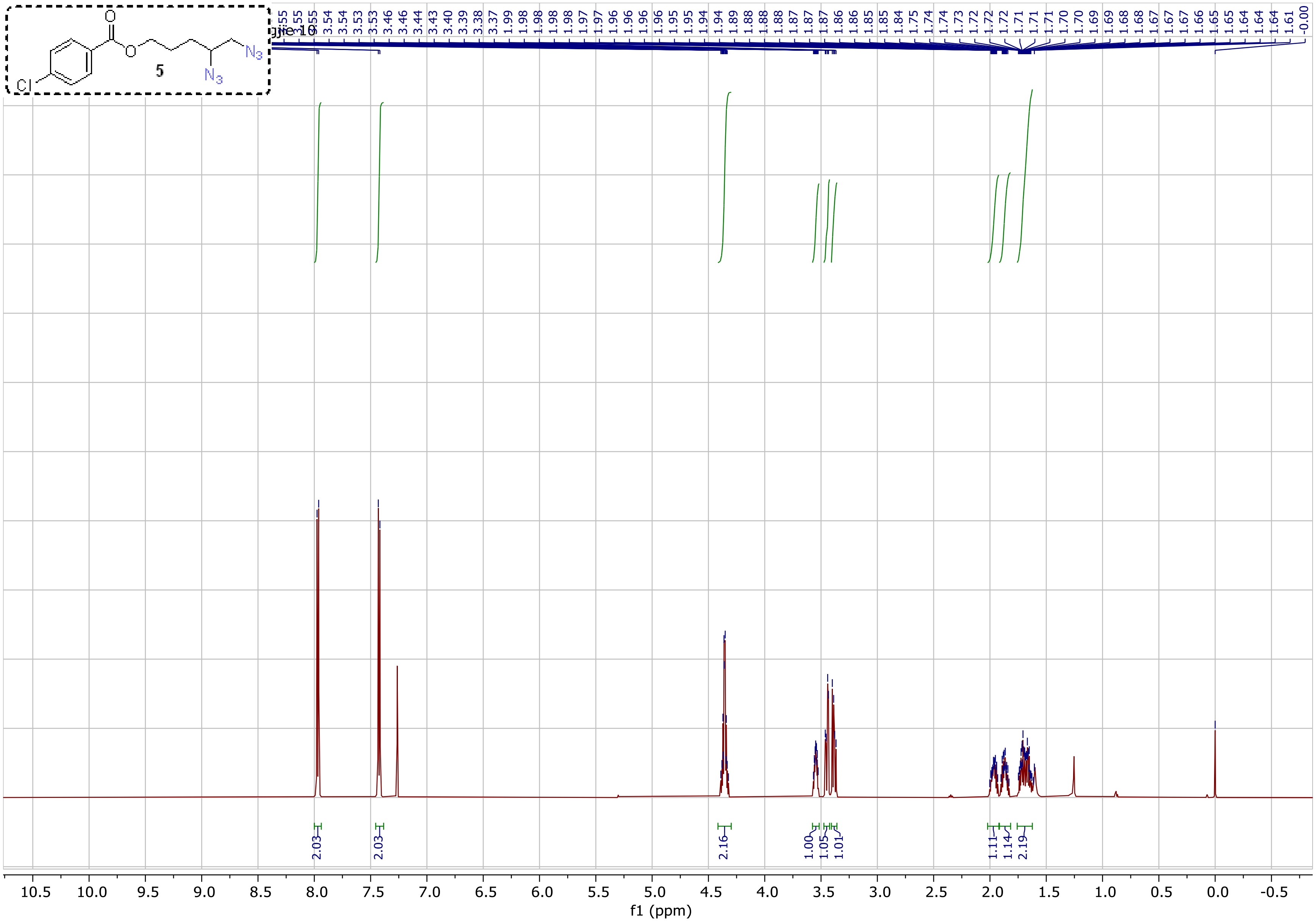

Cl
O
O
N3
N3
5
3.55 3.55 3.55 3.54 3.54 3.53 3.53 3.46 3.44 3.43 3.40 3.39 3.38 3.37 1.99 1.98 1.98 1.98 1.98 1.97 1.97 1.96 1.96 1.96 1.95 1.95 1.94 1.94 1.89 1.88 1.88 1.88 1.87 1.87 1.87 1.86 1.86 1.85 1.85 1.84 1.75 1.74 1.74 1.73 1.72 1.72 1.72 1.71 1.71 1.71 1.70 1.69 1.69 1.68 1.68 1.67 1.67 1.67 1.66 1.65 1.65 1.64 1.64 1.61 -0.00
2.03
2.03
2.16
1.00
1.05
1.01
1.11
1.14
2.19
10.5 10.0 9.5 9.0 8.5 8.0 7.5 7.0 6.5 6.0 5.5 5.0 4.5 4.0 3.5 3.0 2.5 2.0 1.5 1.0 0.5 0.0 -0.5
f1 (ppm)

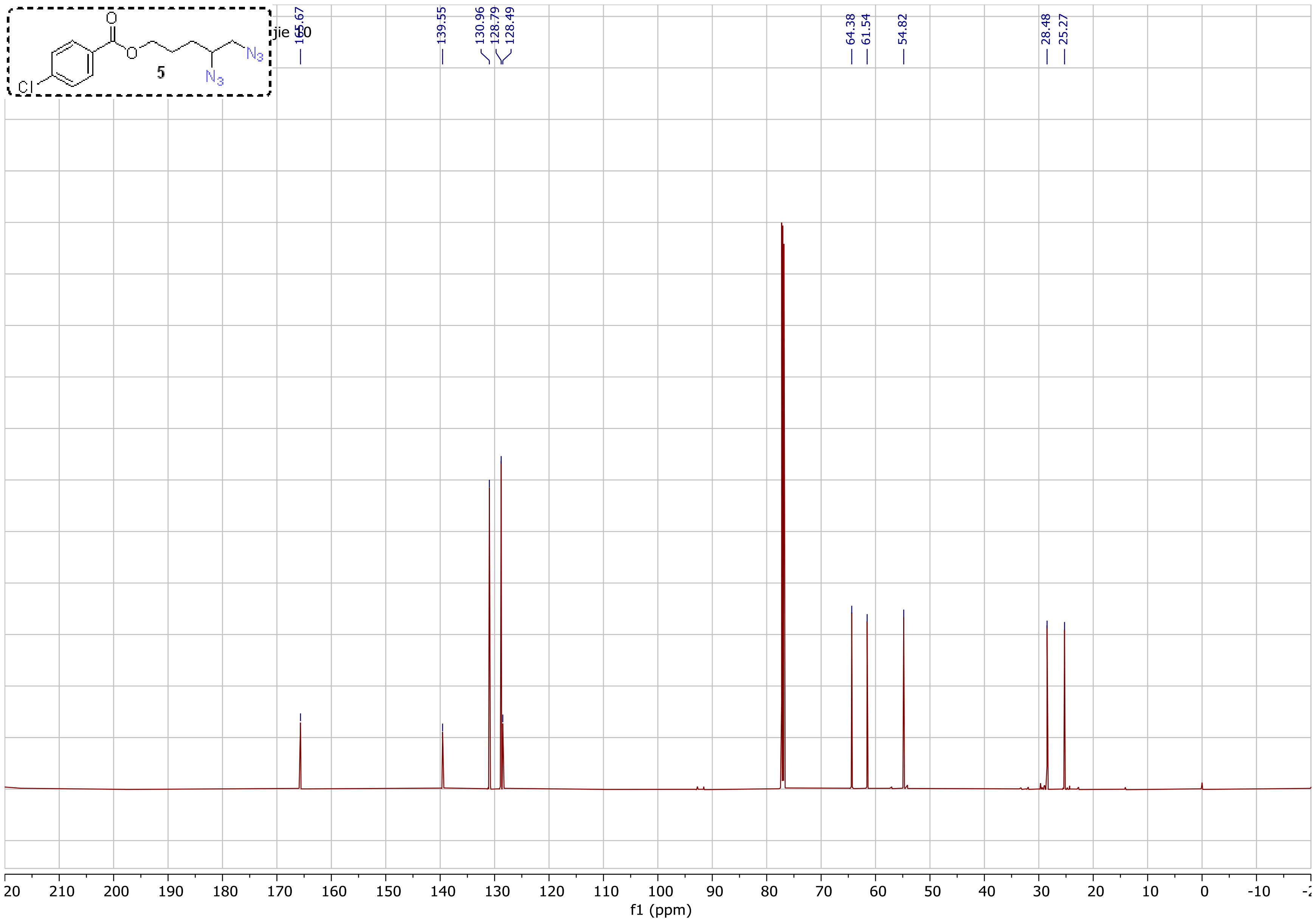

O
O
Cl
5
N3
N3
jie 60
165.67
139.55
130.96
128.79
128.49
64.38
61.54
54.82
28.48
25.27
f1 (ppm)

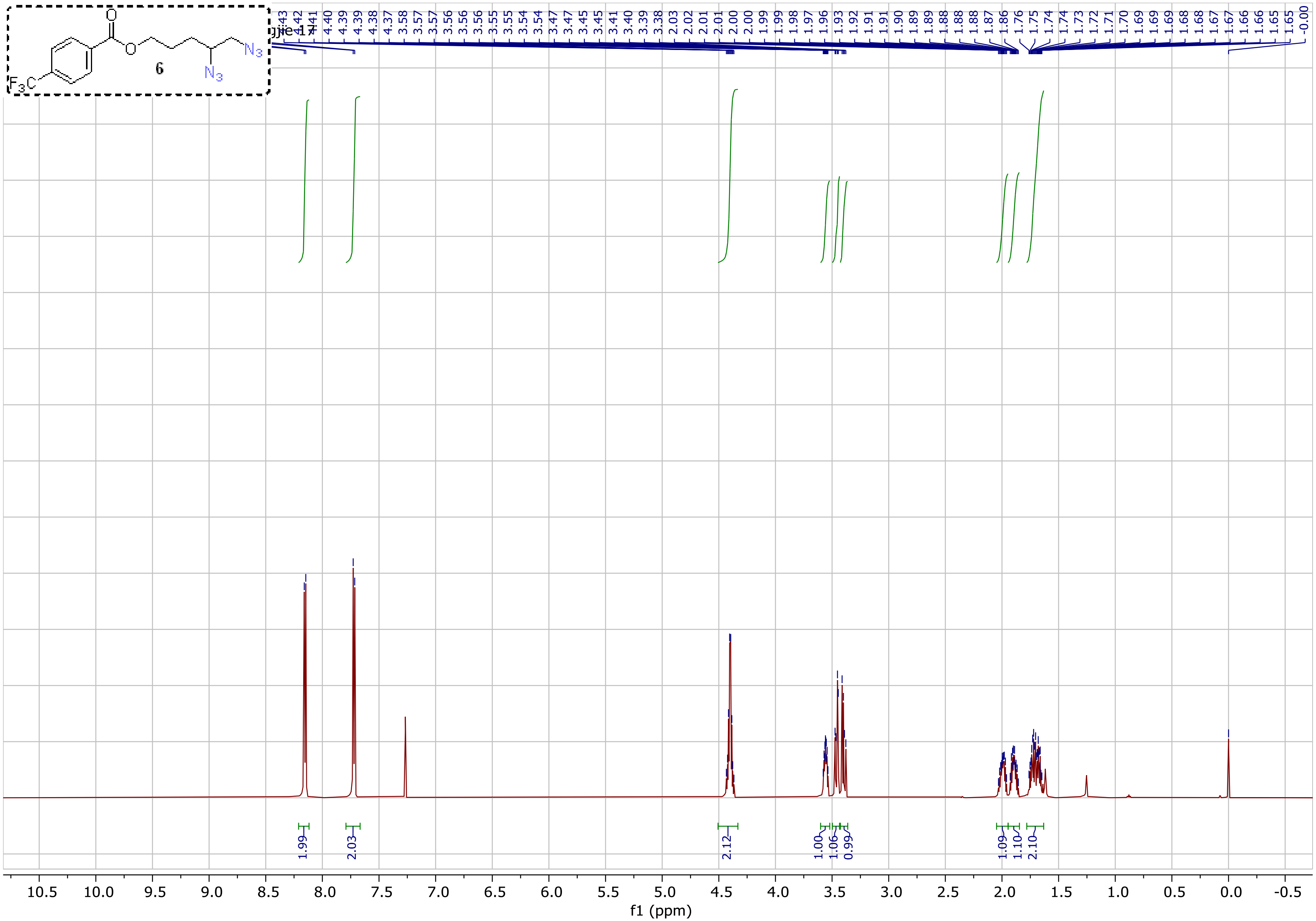
6
F3C
N3
N3
1.99
2.03
2.12
1.00
1.06
0.99
1.09
1.10
2.10
f1 (ppm)

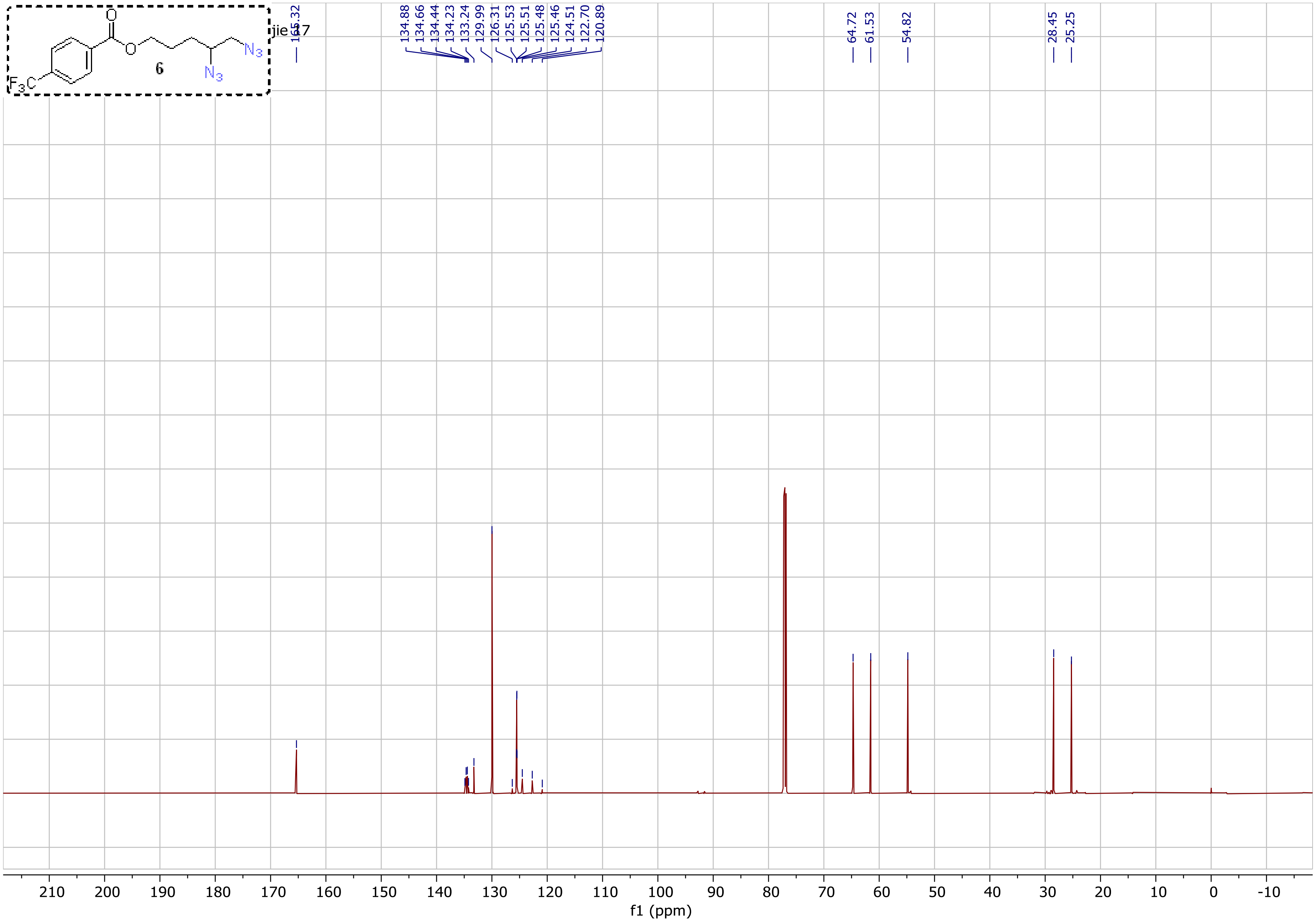
6
jie17
165.32
134.88
134.66
134.44
134.23
133.24
129.99
126.31
125.53
125.51
125.48
125.46
124.51
122.70
120.89
64.72
61.53
54.82
28.45
25.25
210 200 190 180 170 160 150 140 130 120 110 100 90 80 70 60 50 40 30 20 10 0 -10
f1 (ppm)

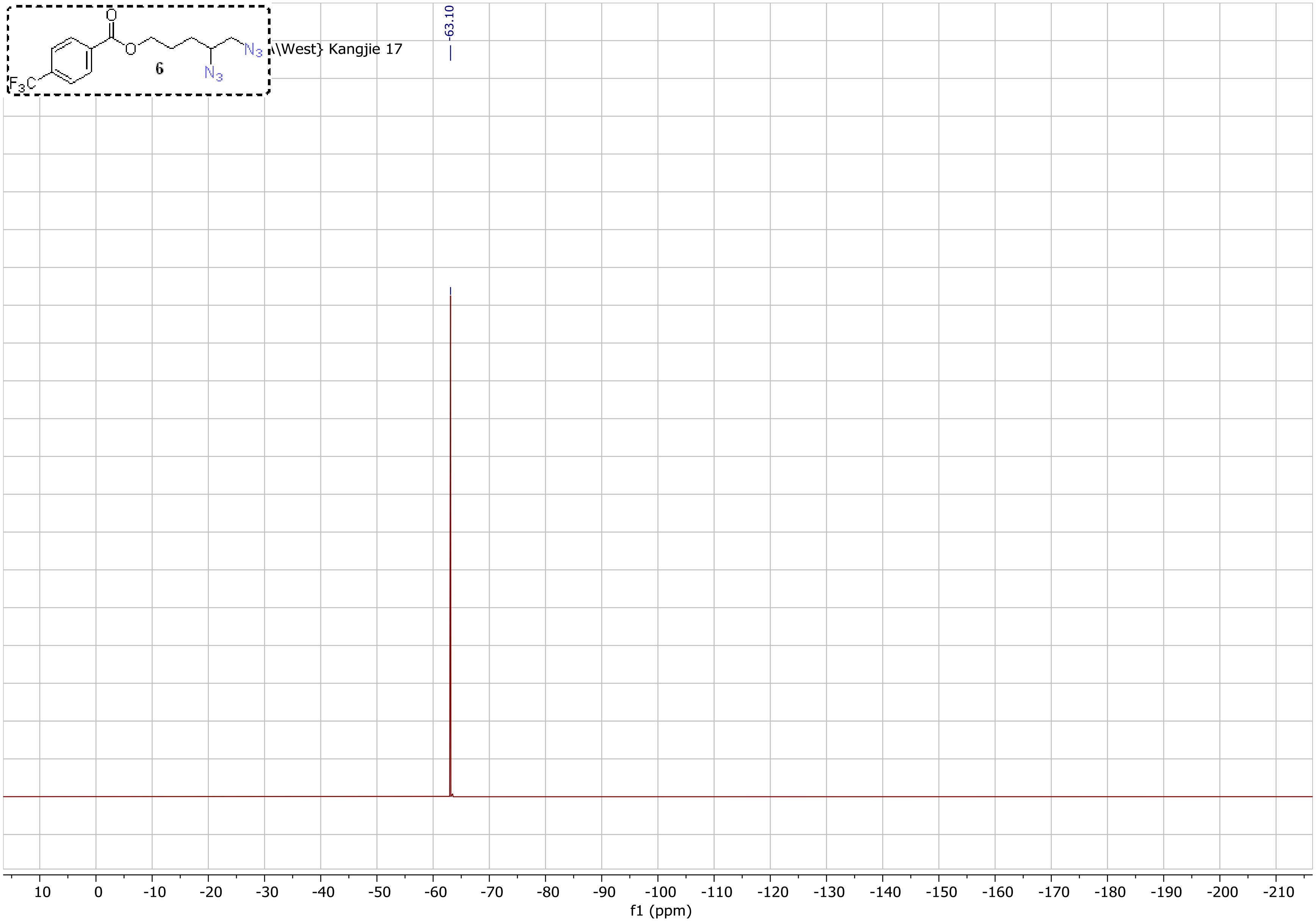
O
O
F3C
6
N3
N3
\\West} Kangjie 17
-63.10
10 0 -10 -20 -30 -40 -50 -60 -70 -80 -90 -100 -110 -120 -130 -140 -150 -160 -170 -180 -190 -200 -210
f1 (ppm)

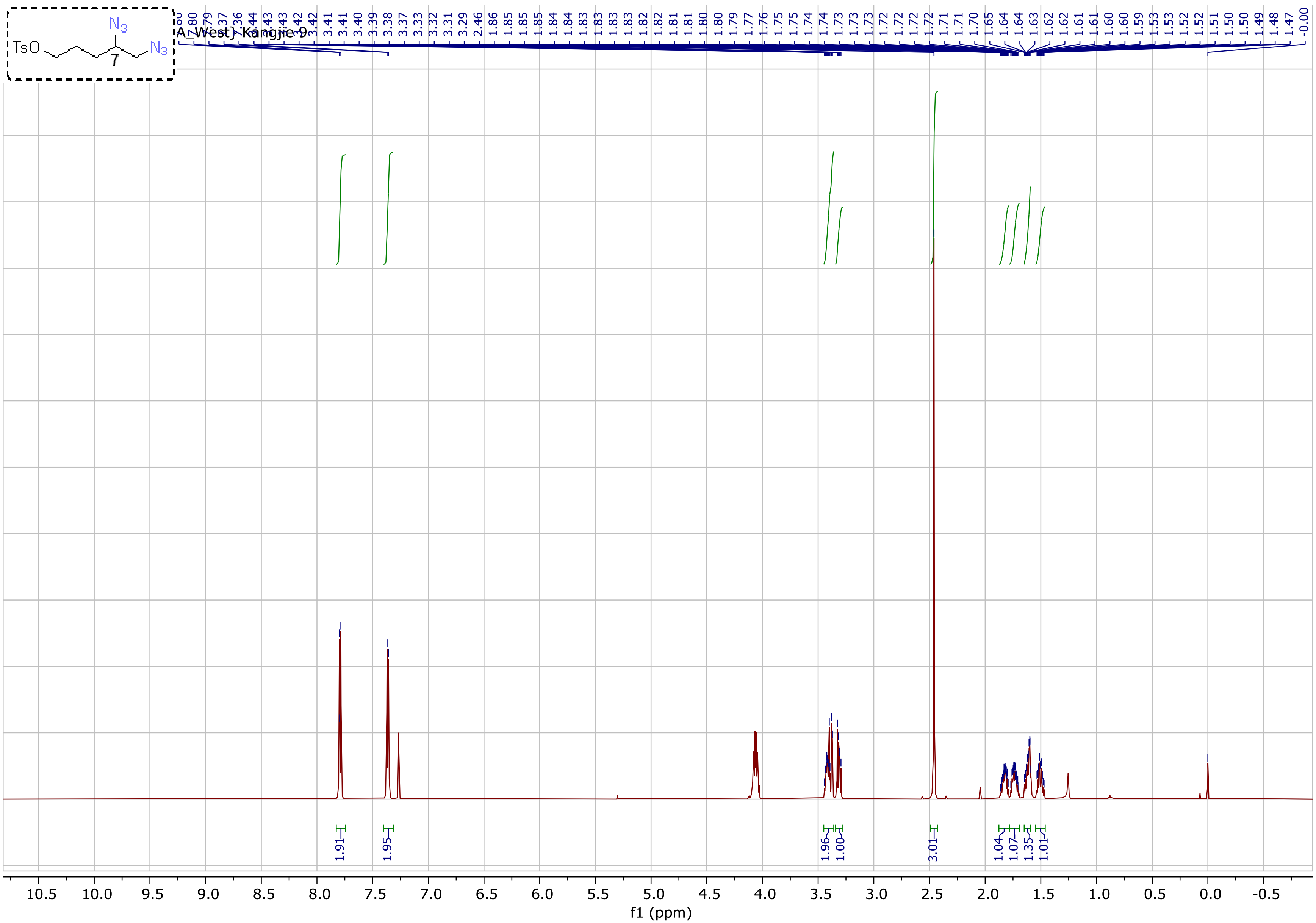
A_West} Kangjie 9
TsO
N3
N3
7
f1 (ppm)

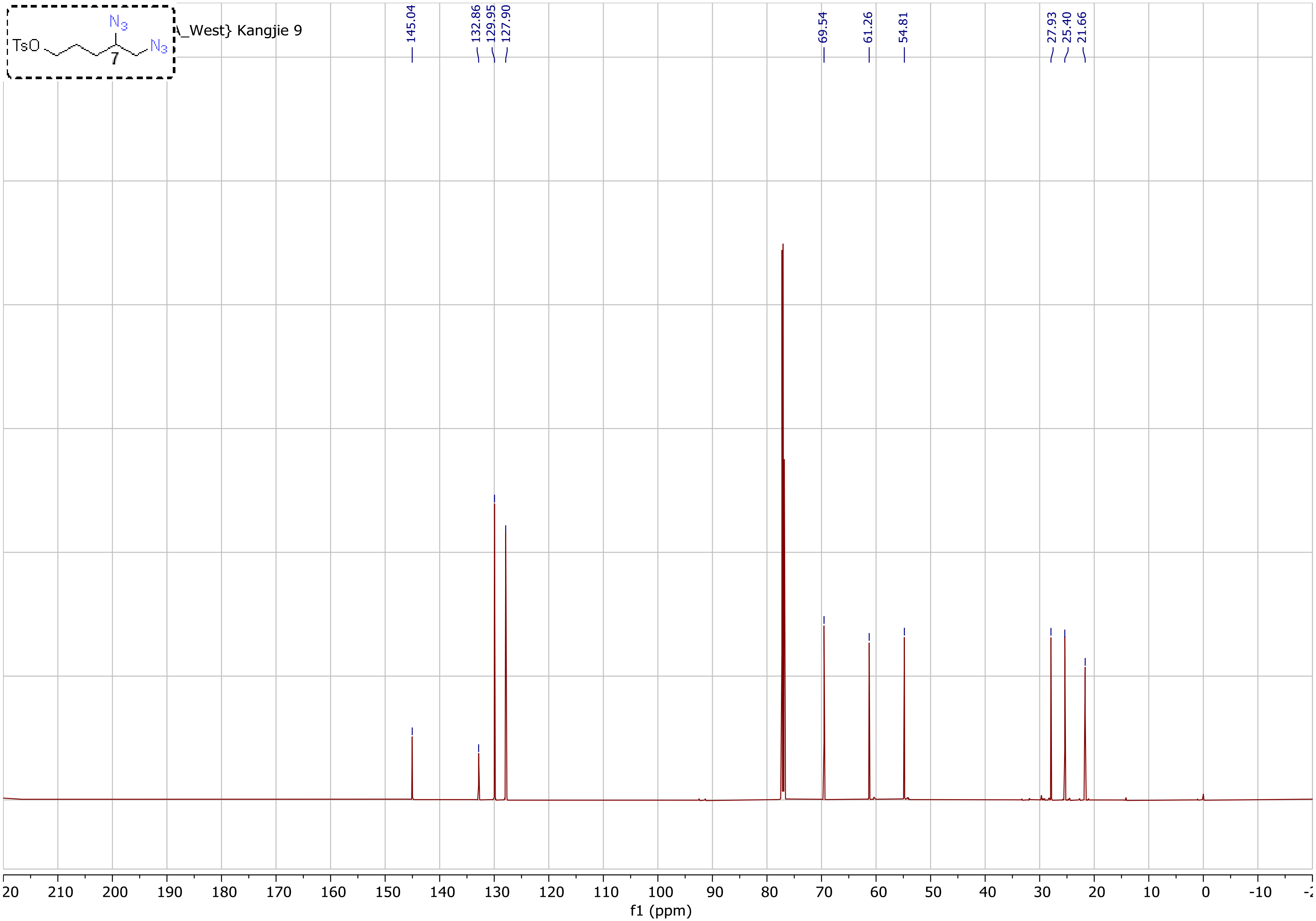
TsO
N3
N3
7
_West} Kangjie 9
145.04
132.86
129.95
127.90
69.54
61.26
54.81
27.93
25.40
21.66
20
210
200
190
180
170
160
150
140
130
120
110
100
90
80
70
60
50
40
30
20
10
0
-10
f1 (ppm)

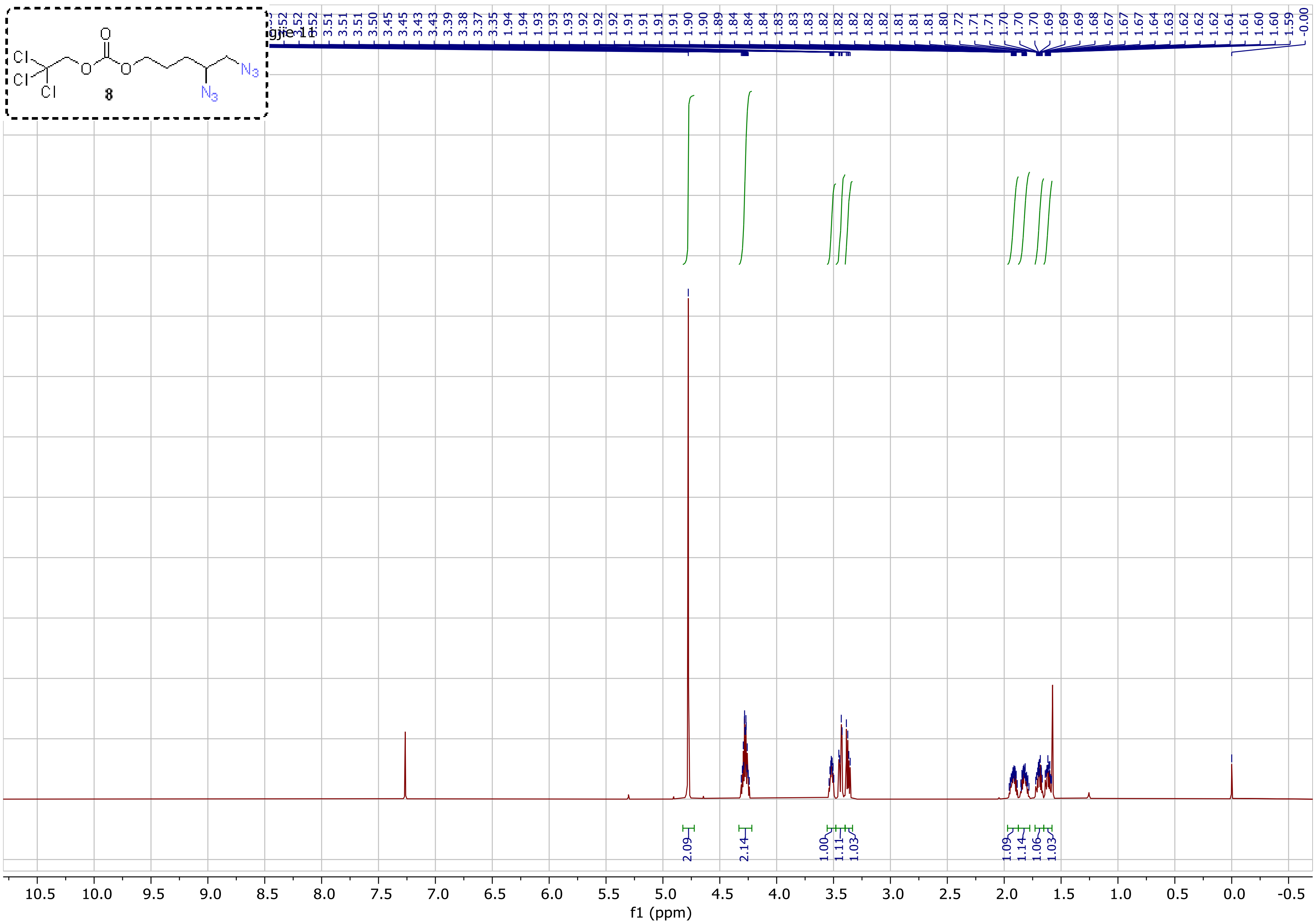

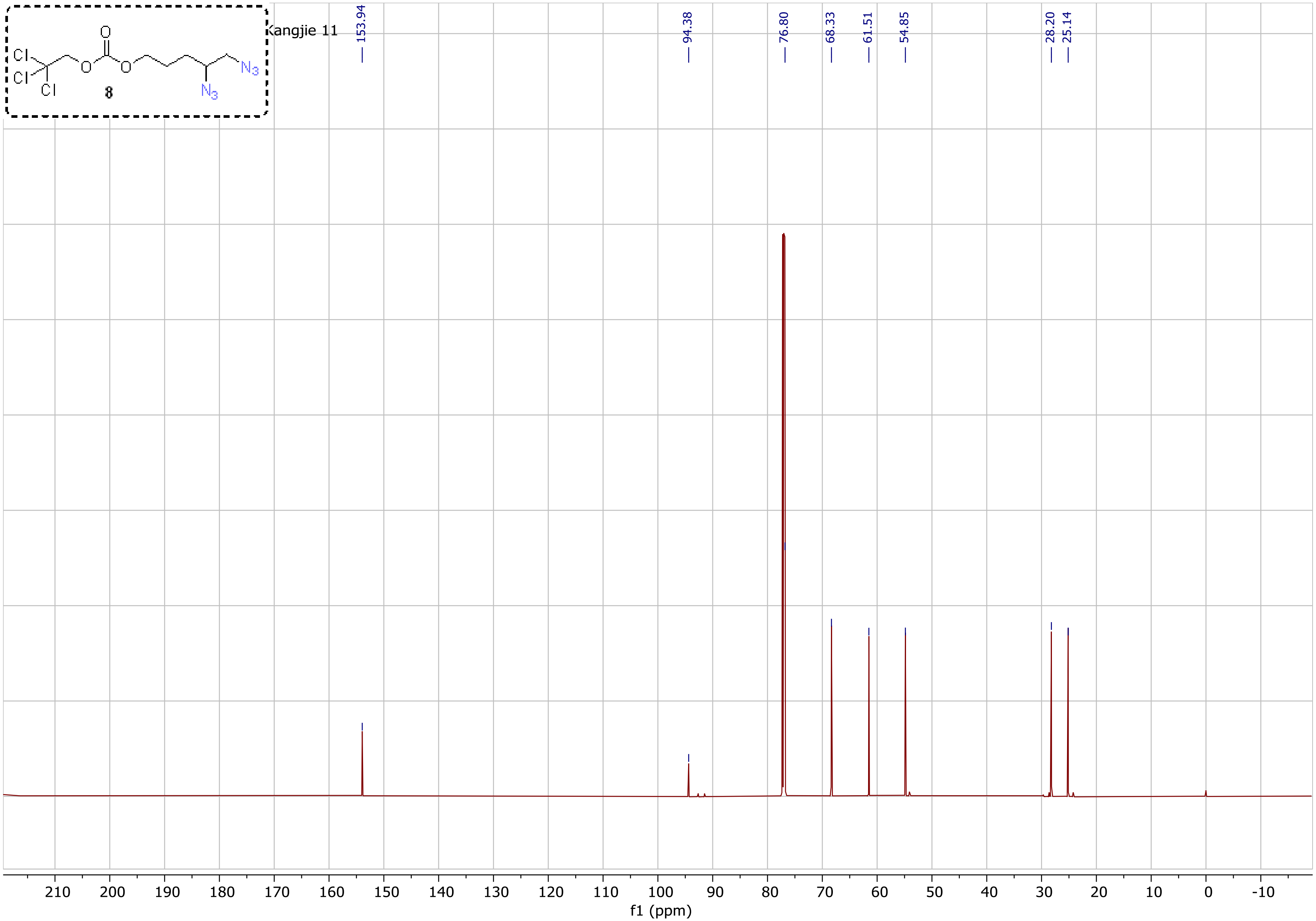
Kangjie 11
153.94
94.38
76.80
68.33
61.51
54.85
28.20
25.14
Cl
Cl
Cl
O
O
O
N3
N3
8
210 200 190 180 170 160 150 140 130 120 110 100 90 80 70 60 50 40 30 20 10 0 -10
f1 (ppm)

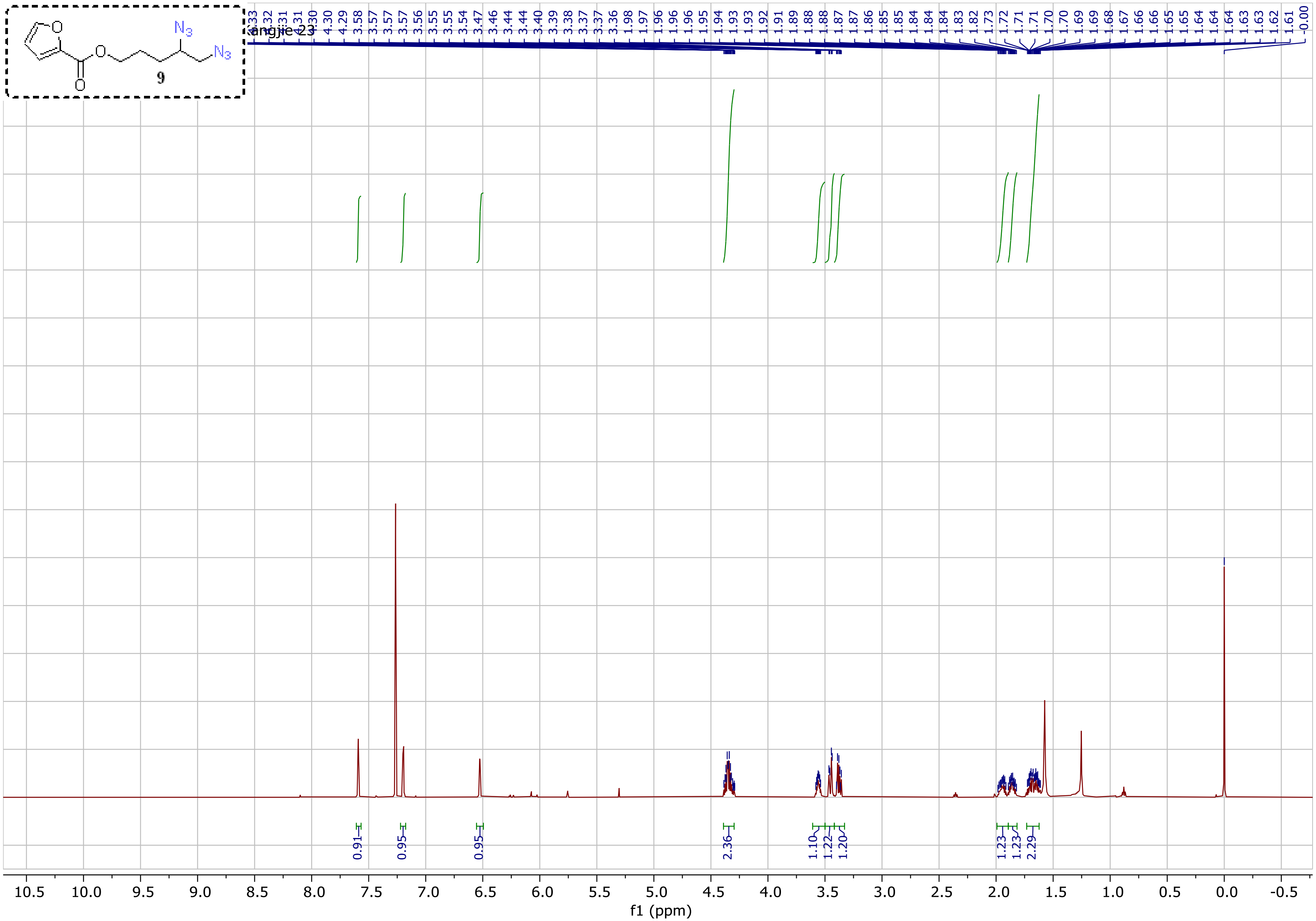
9
N3
N3
angjie 23
0.91
0.95
0.95
2.36
1.10
1.22
1.20
1.23
1.23
2.29
f1 (ppm)

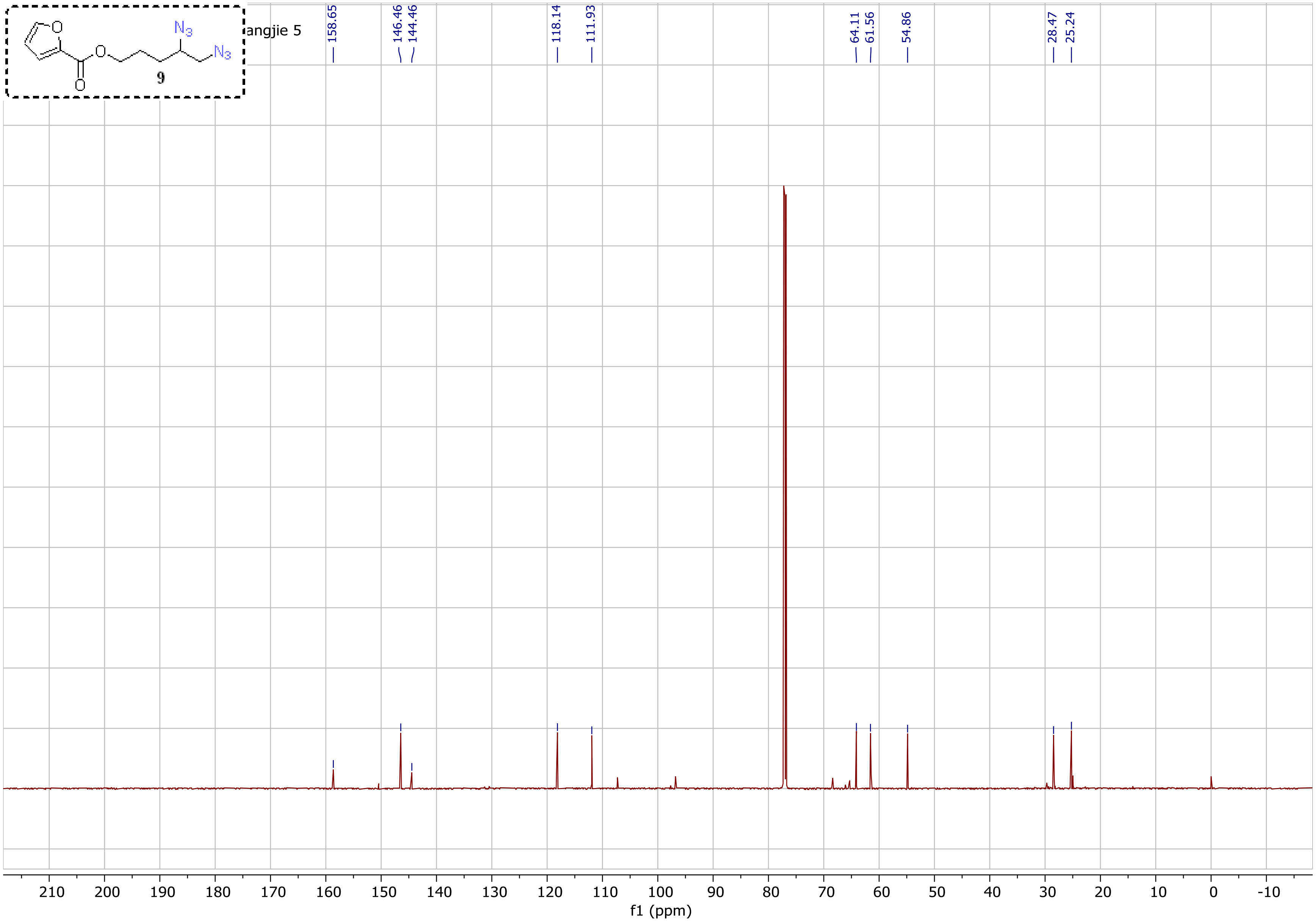

O
N3
O
N3
O
9
angjie 5
158.65
146.46
144.46
118.14
111.93
64.11
61.56
54.86
28.47
25.24
210 200 190 180 170 160 150 140 130 120 110 100 90 80 70 60 50 40 30 20 10 0 -10
f1 (ppm)

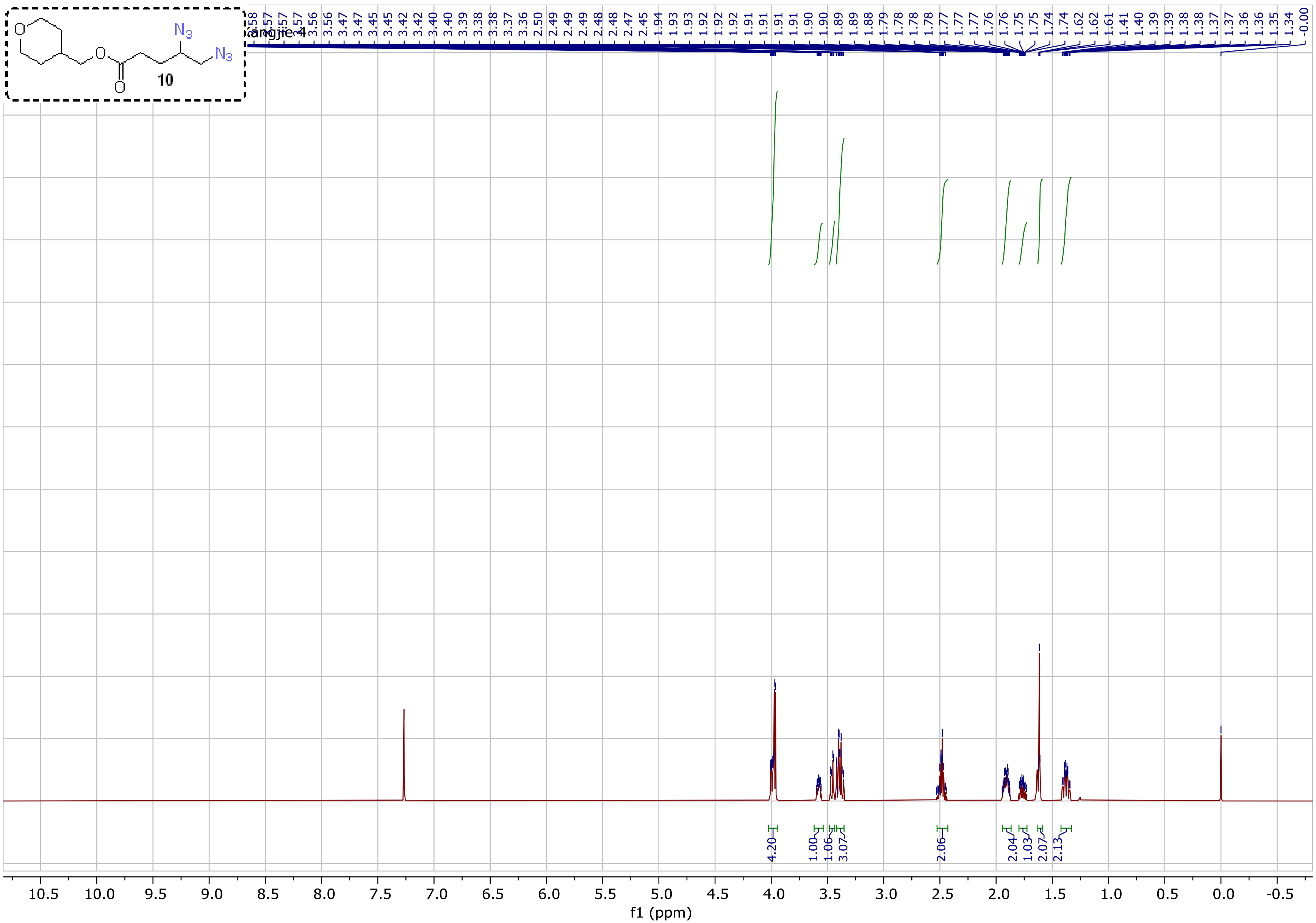

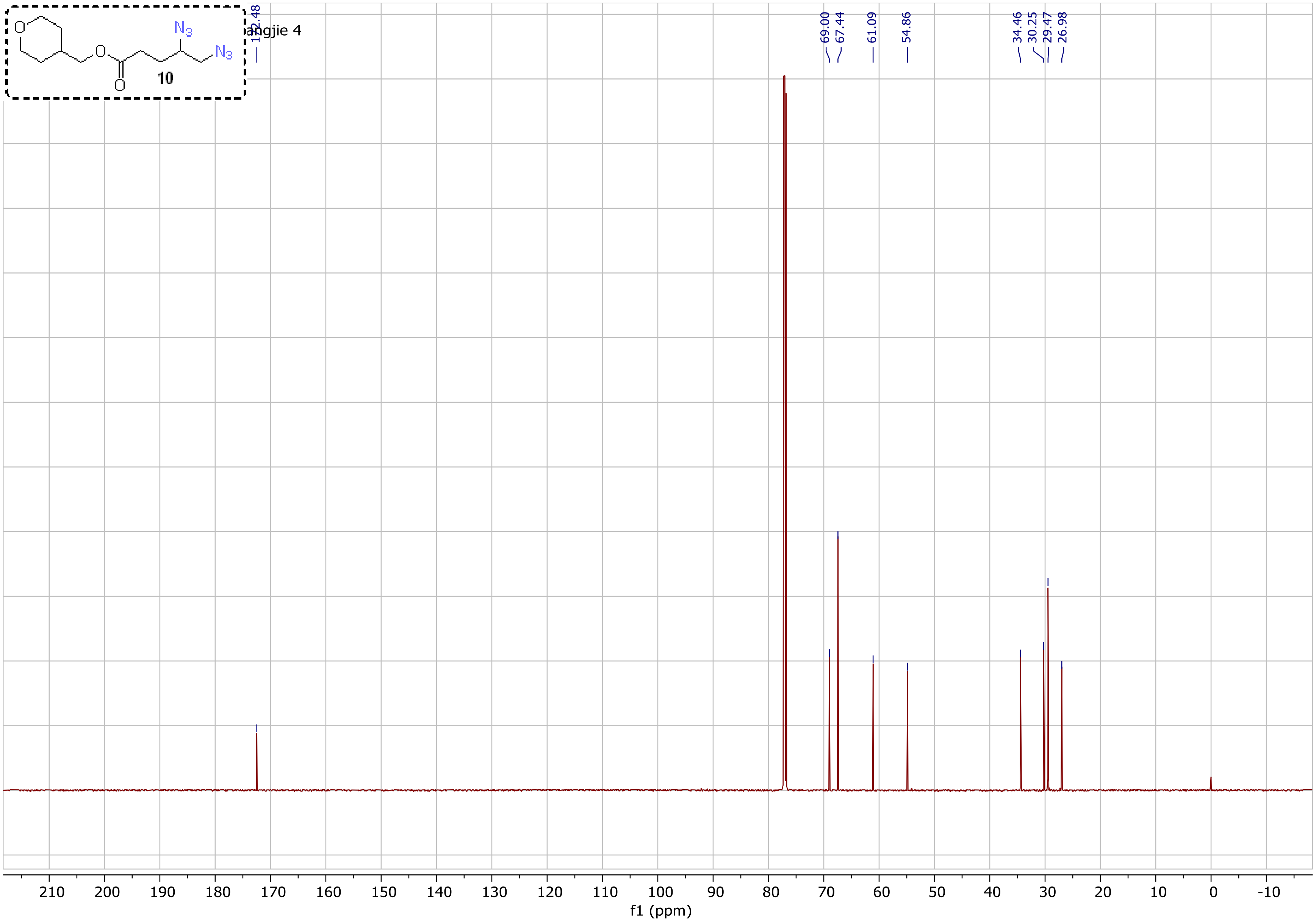
10
angjie 4
172.48
69.00
67.44
61.09
54.86
34.46
30.25
29.47
26.98
210
200
190
180
170
160
150
140
130
120
110
100
90
80
70
60
50
40
30
20
10
0
-10
f1 (ppm)

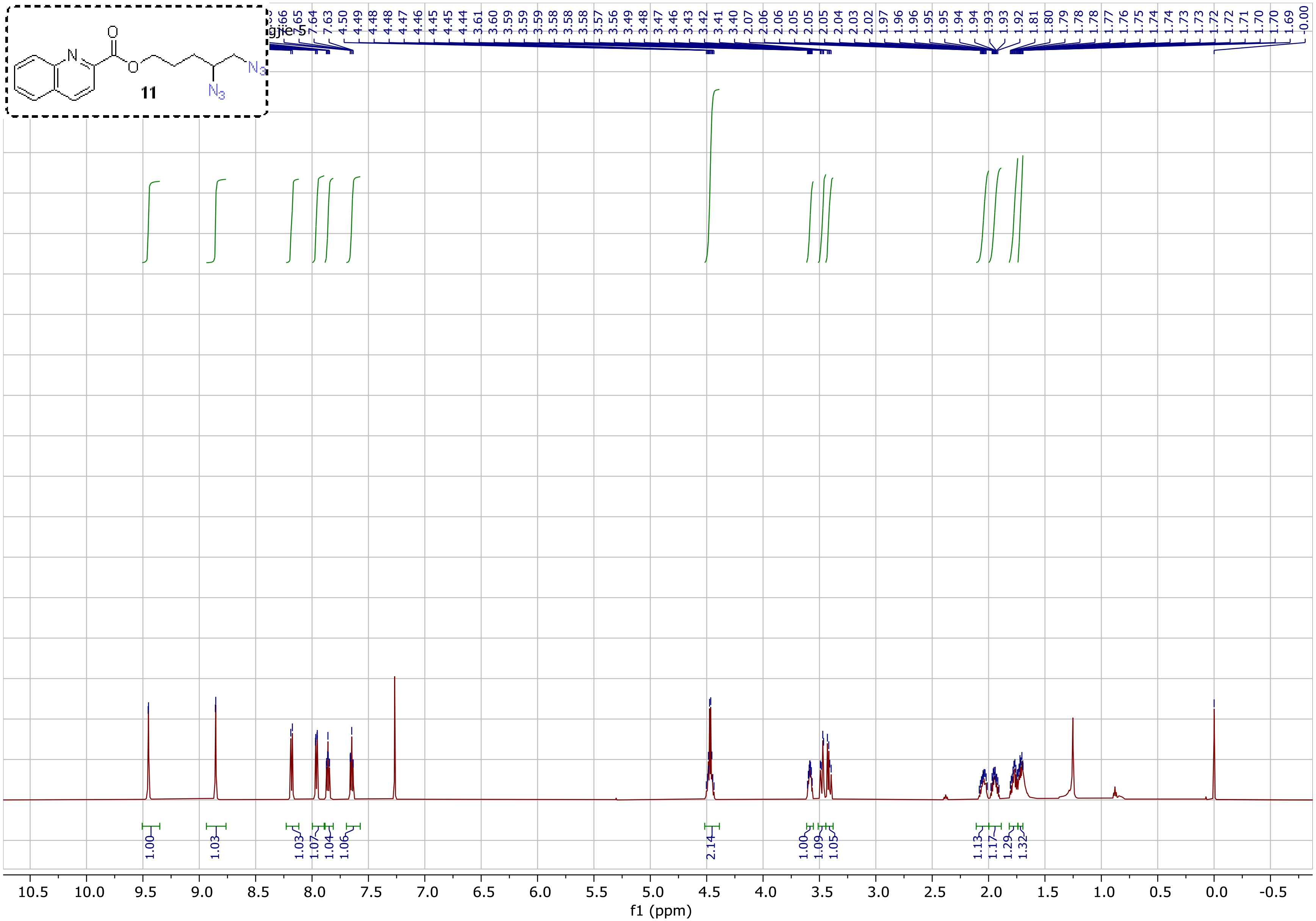

11
N
O
O
N_3
N_3
7.65
7.64
7.63
4.50
4.49
4.48
4.48
4.47
4.46
4.45
4.45
4.44
3.61
3.60
3.59
3.59
3.58
3.58
3.57
3.56
3.49
3.48
3.47
3.46
3.43
3.42
3.41
3.40
2.07
2.06
2.06
2.05
2.05
2.05
2.04
2.03
2.02
1.97
1.96
1.96
1.95
1.95
1.94
1.94
1.93
1.93
1.92
1.81
1.80
1.79
1.78
1.78
1.77
1.76
1.75
1.74
1.74
1.73
1.73
1.72
1.72
1.71
1.70
1.70
1.69
-0.00
1.00
1.03
1.03
1.07
1.04
1.06
2.14
1.00
1.09
1.05
1.13
1.17
1.29
1.32
10.5 10.0 9.5 9.0 8.5 8.0 7.5 7.0 6.5 6.0 5.5 5.0 4.5 4.0 3.5 3.0 2.5 2.0 1.5 1.0 0.5 0.0 -0.5
f1 (ppm)

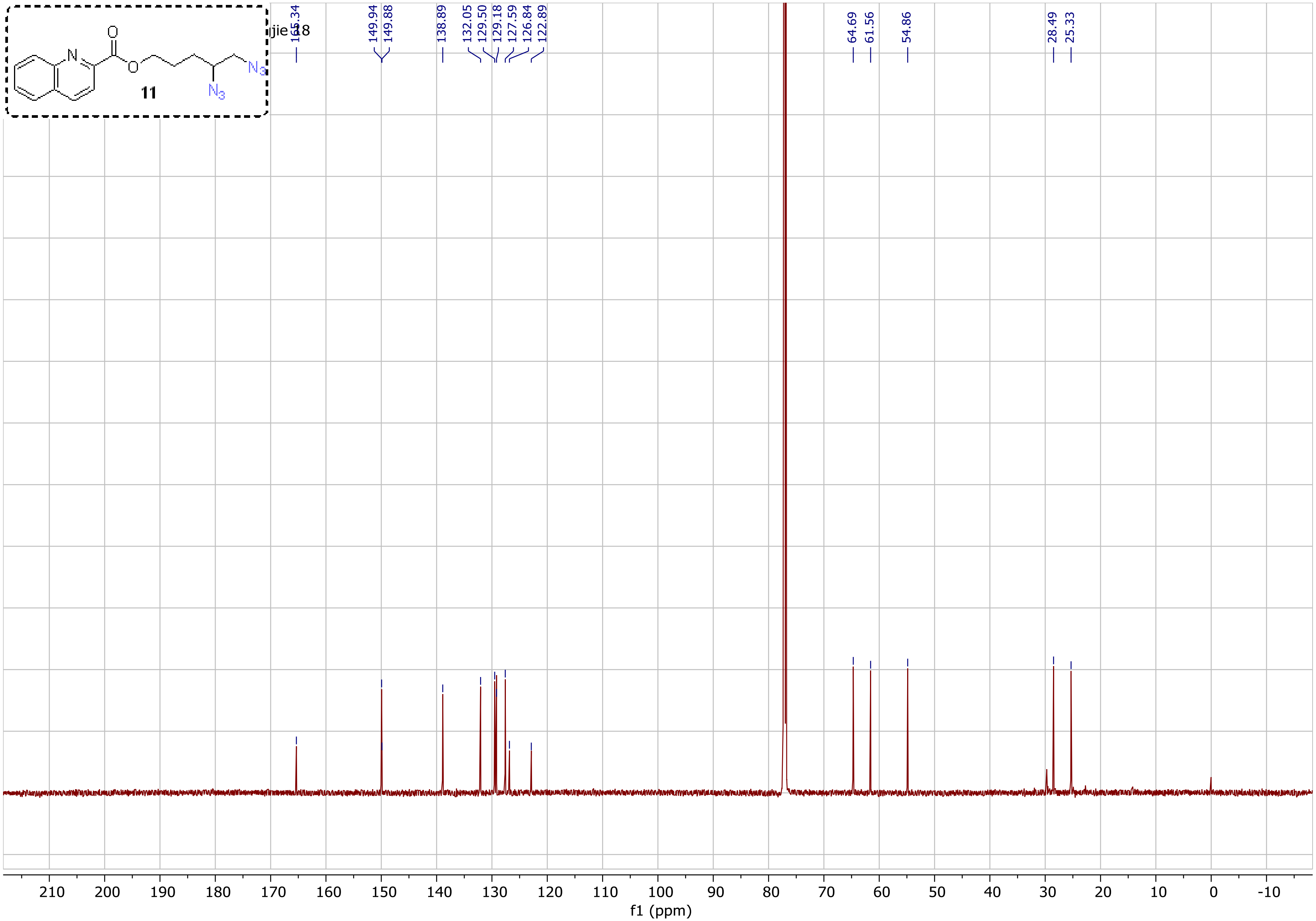

jie18
11
N
O
O
N3
N3
165.34
149.94
149.88
138.89
132.05
129.50
129.18
127.59
126.84
122.89
64.69
61.56
54.86
28.49
25.33
210 200 190 180 170 160 150 140 130 120 110 100 90 80 70 60 50 40 30 20 10 0 -10
f1 (ppm)

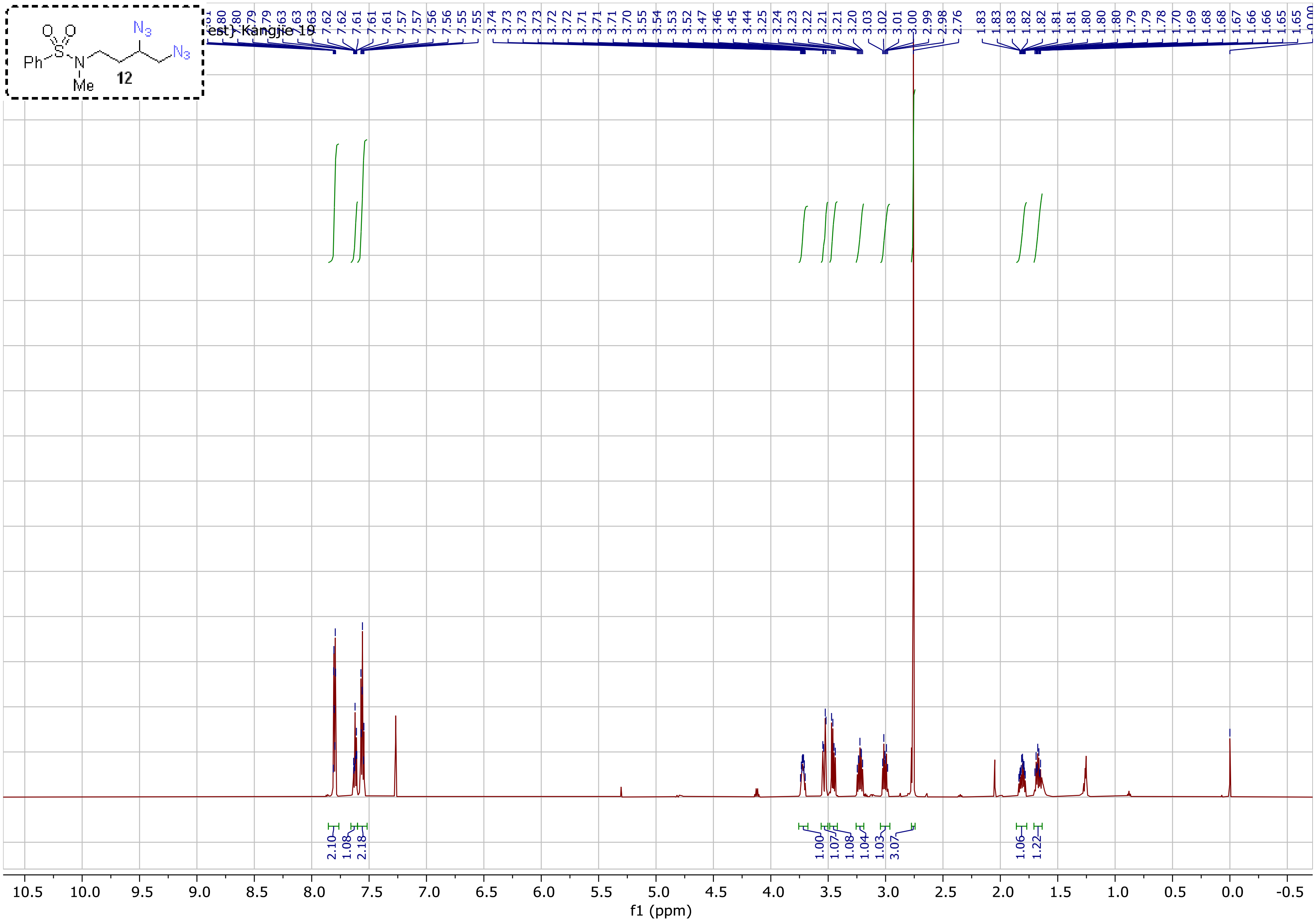

Ph
N
Me
12
N3
N3
f1 (ppm)

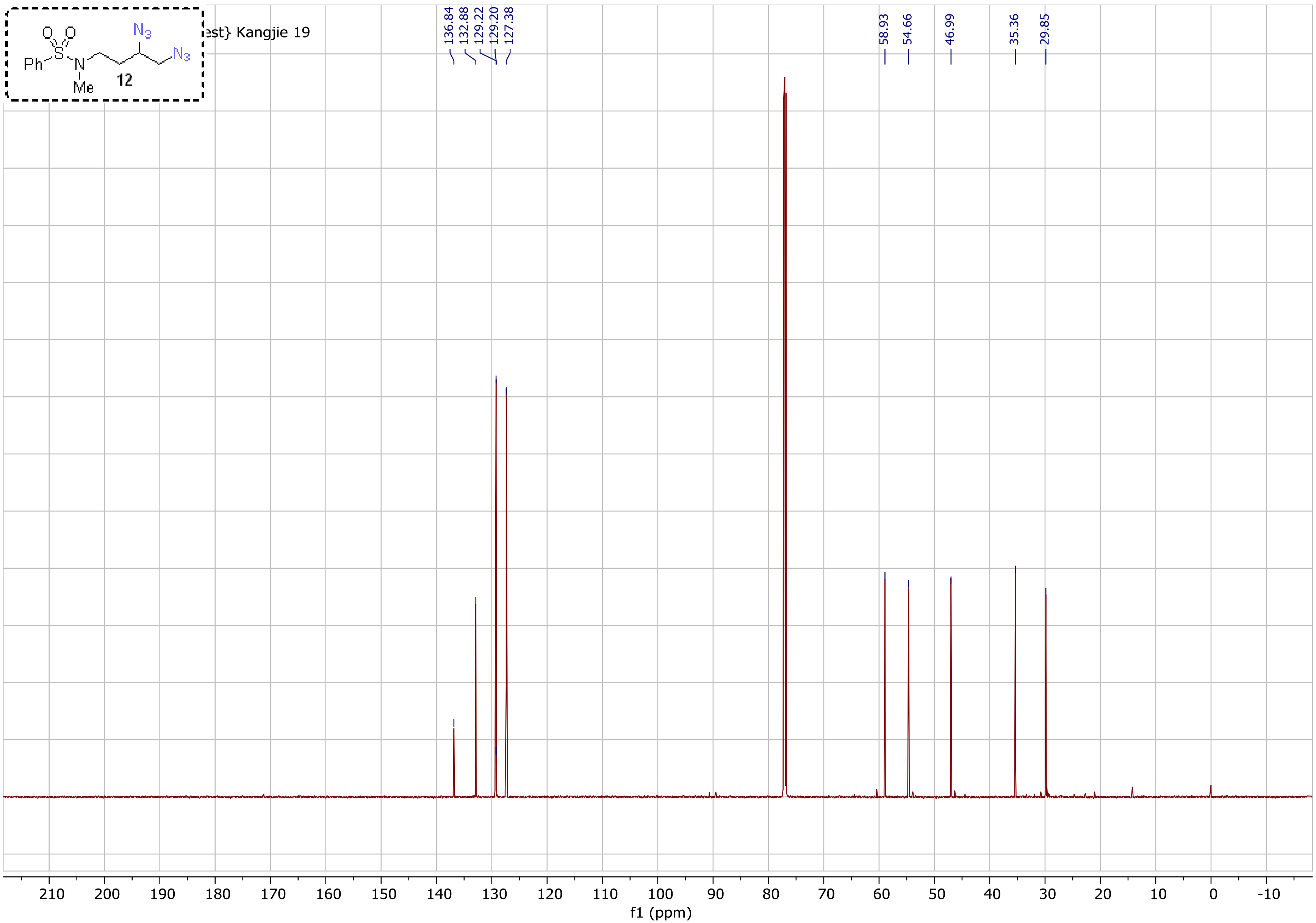

Ph S N Me N3 N3
12
est} Kangjie 19
136.84
132.88
129.22
129.20
127.38
58.93
54.66
46.99
35.36
29.85
210 200 190 180 170 160 150 140 130 120 110 100 90 80 70 60 50 40 30 20 10 0 -10
f1 (ppm)

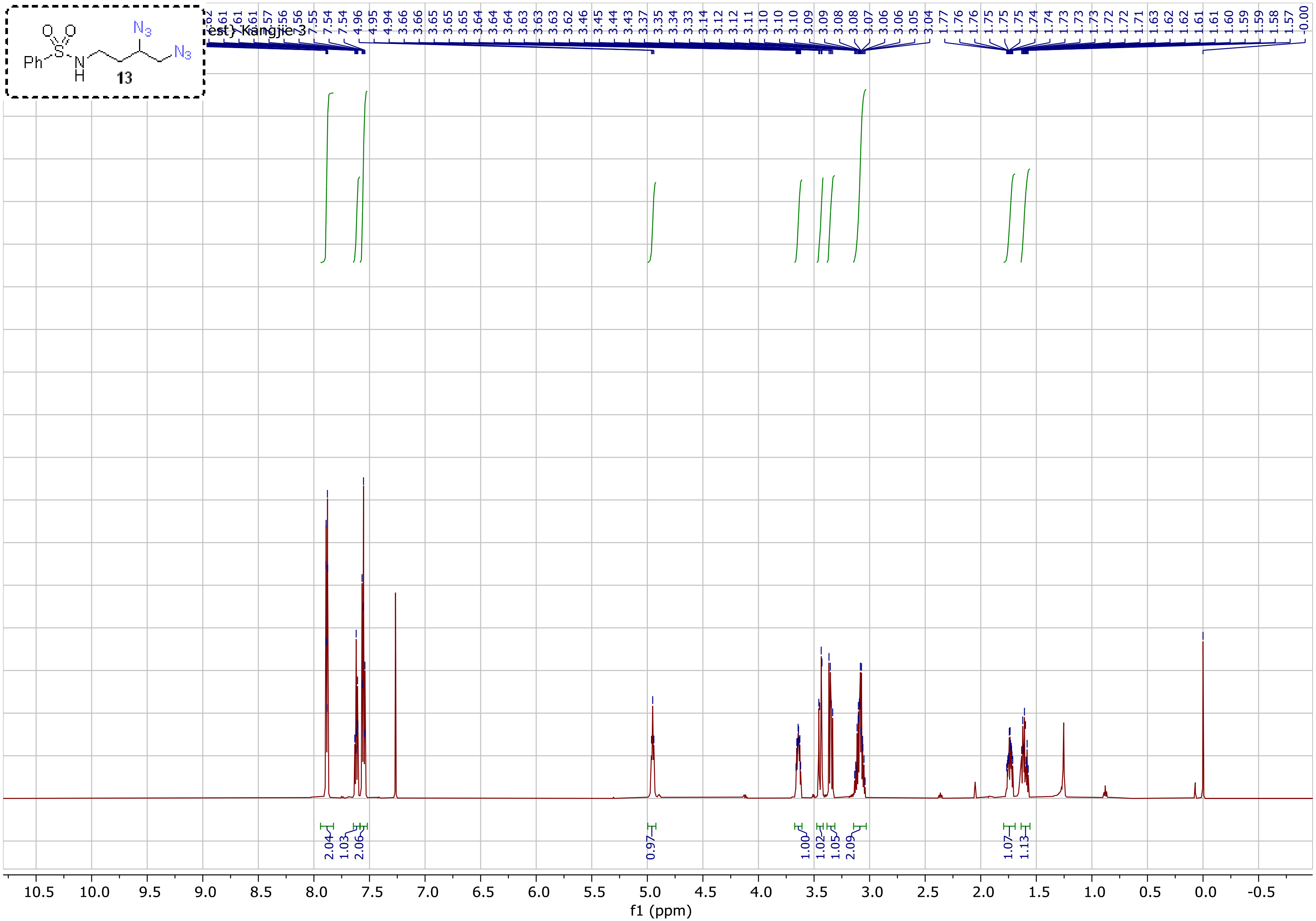

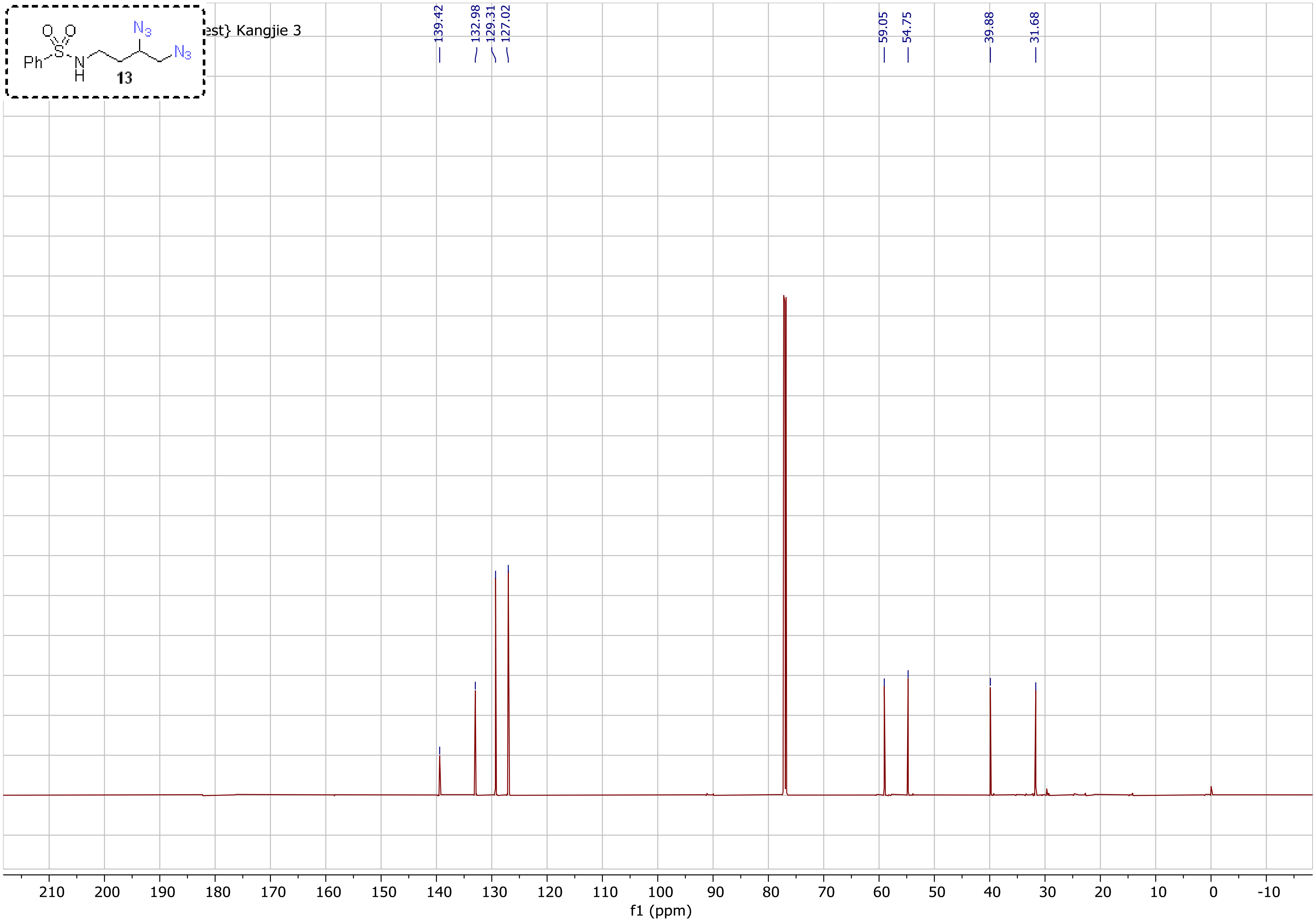

Ph
S
O O
N
H
N3
N3
13
est} Kangjie 3
139.42
132.98
129.31
127.02
59.05
54.75
39.88
31.68
210 200 190 180 170 160 150 140 130 120 110 100 90 80 70 60 50 40 30 20 10 0 -10
f1 (ppm)

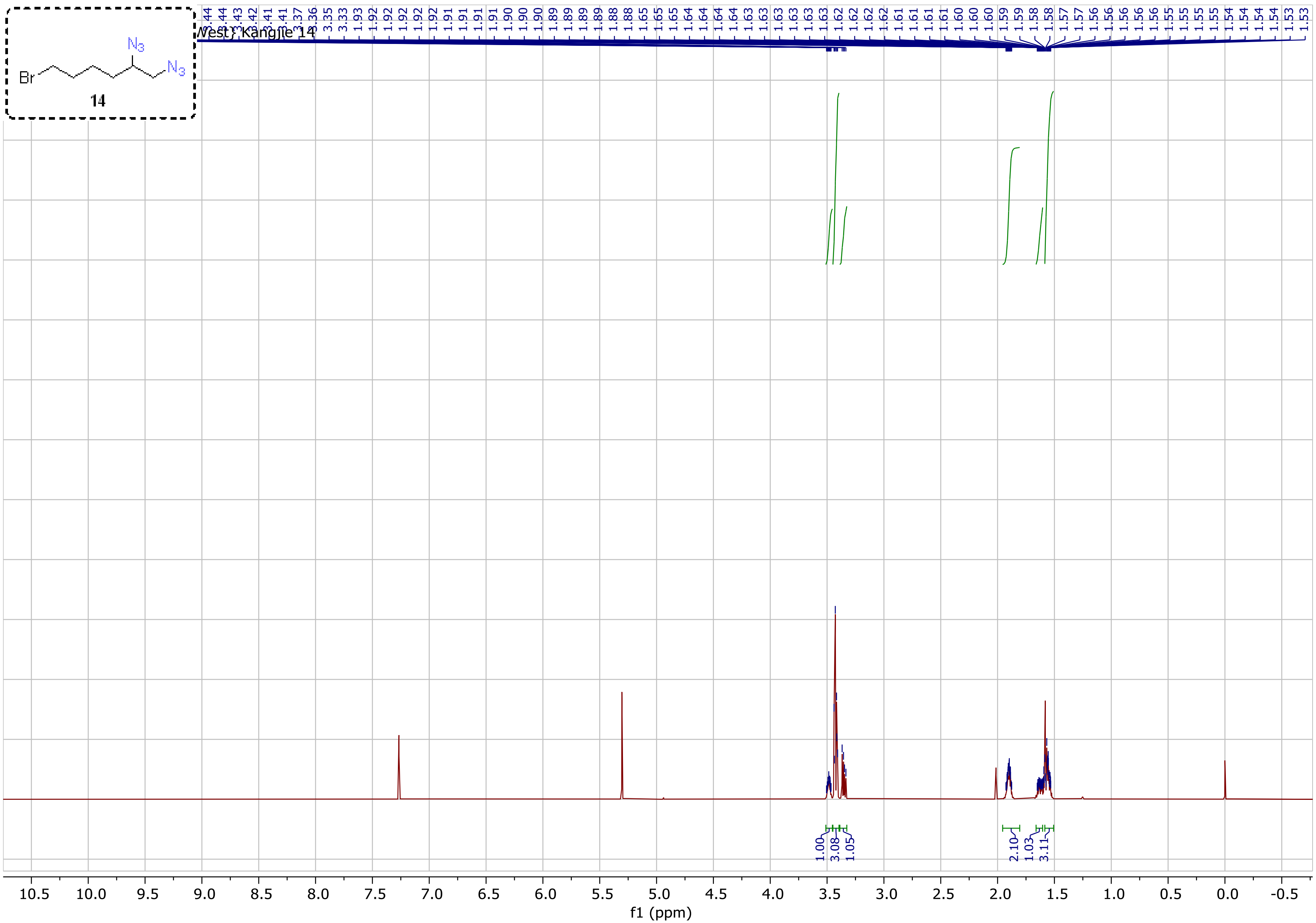

West} Kangjie 14
14
f1 (ppm)

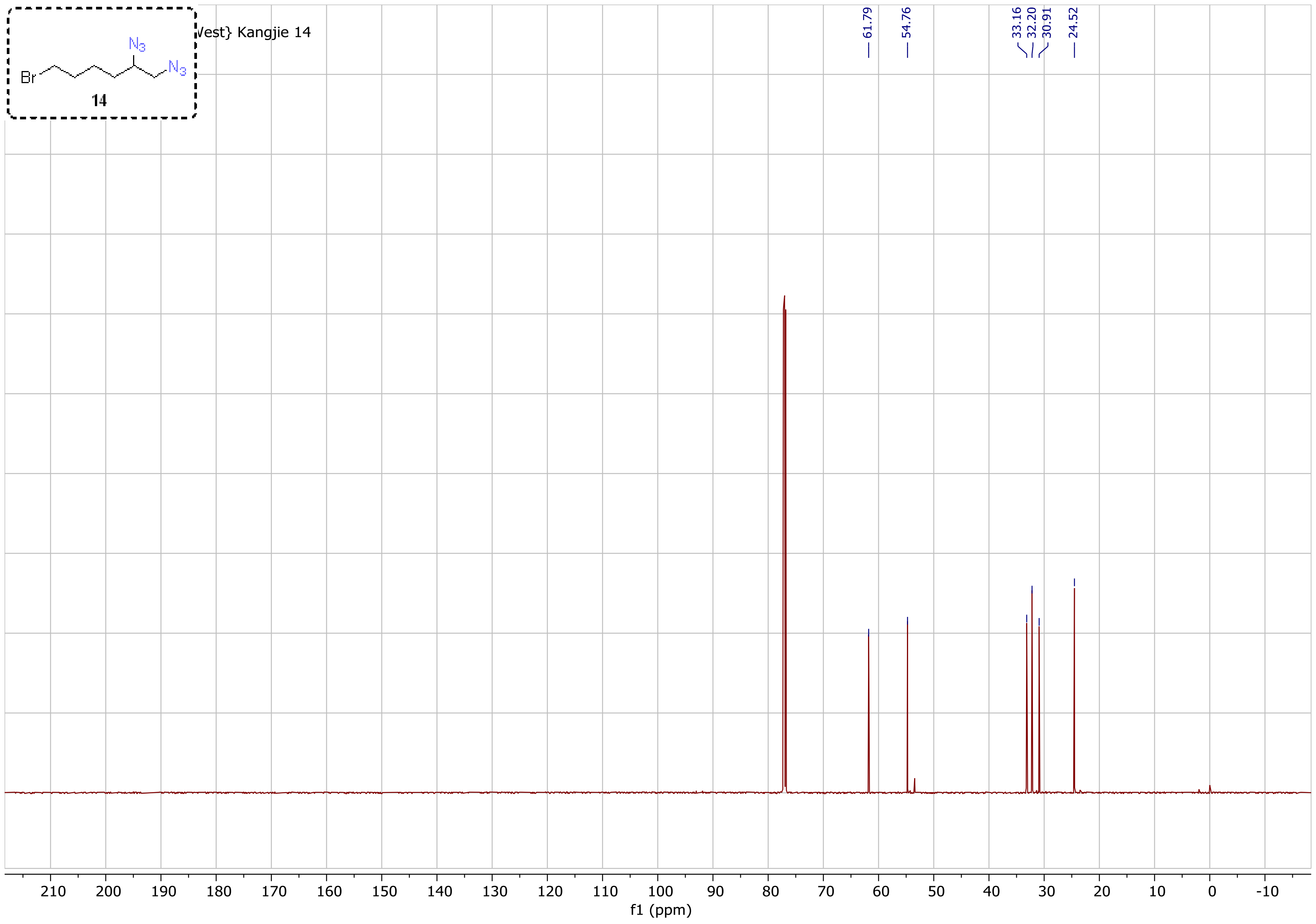

Vest} Kangjie 14
61.79
54.76
33.16
32.20
30.91
24.52
Br
N3
N3
14
f1 (ppm)

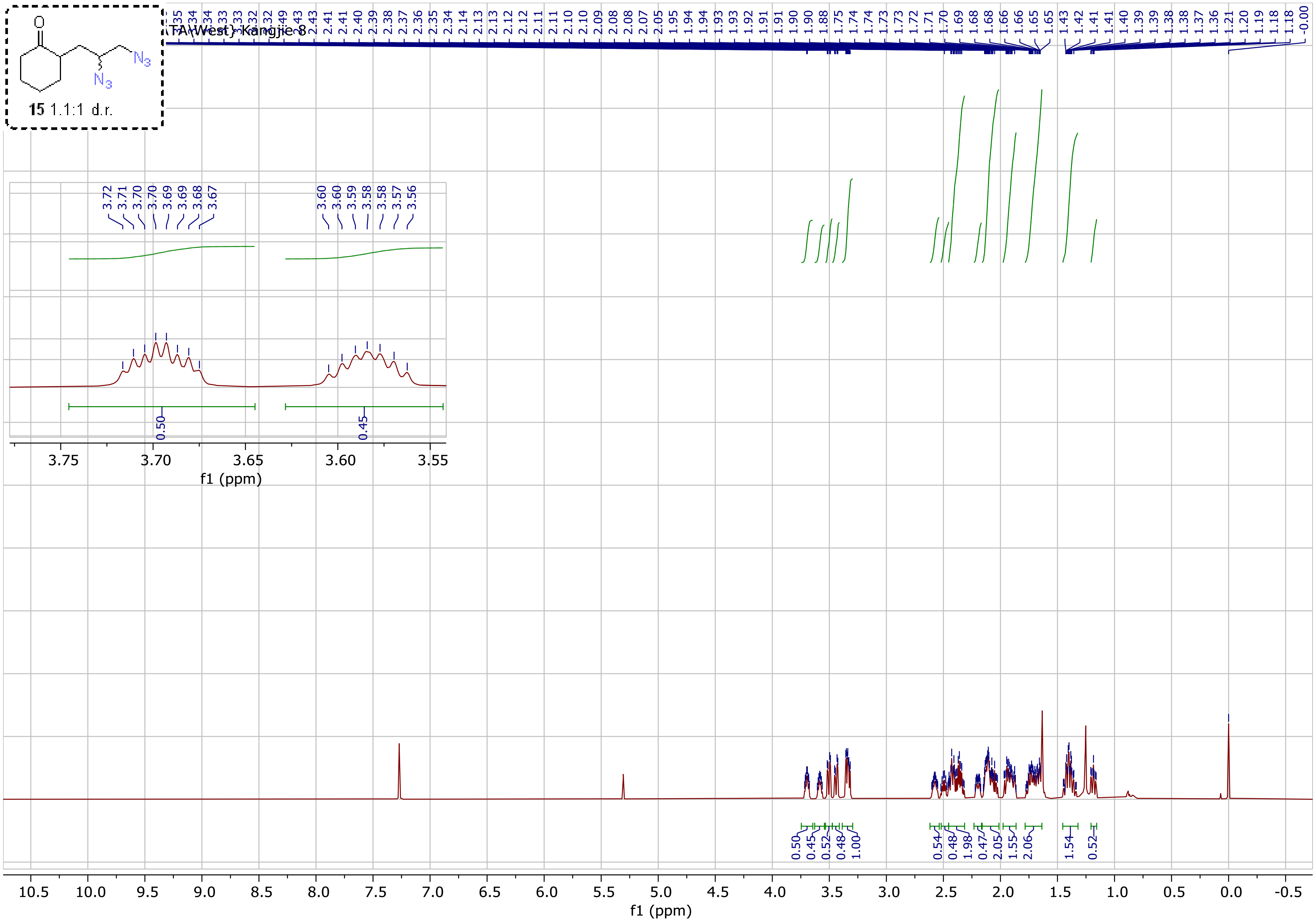

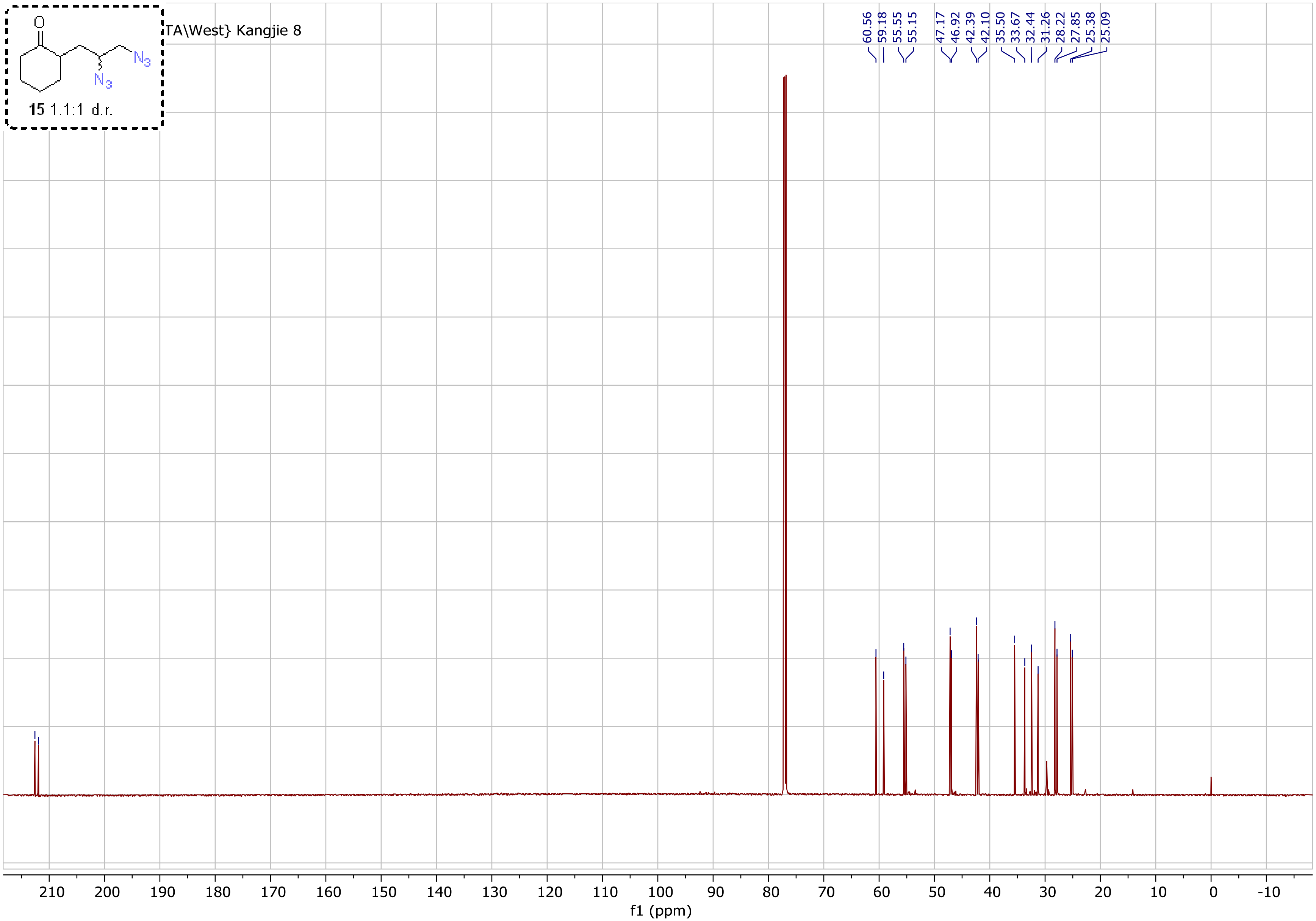
TA\West} Kangjie 8
O
N3
N3
15 1.1:1 d.r.
60.56
59.18
55.55
55.15
47.17
46.92
42.39
42.10
35.50
33.67
32.44
31.26
28.22
27.85
25.38
25.09
210 200 190 180 170 160 150 140 130 120 110 100 90 80 70 60 50 40 30 20 10 0 -10
f1 (ppm)

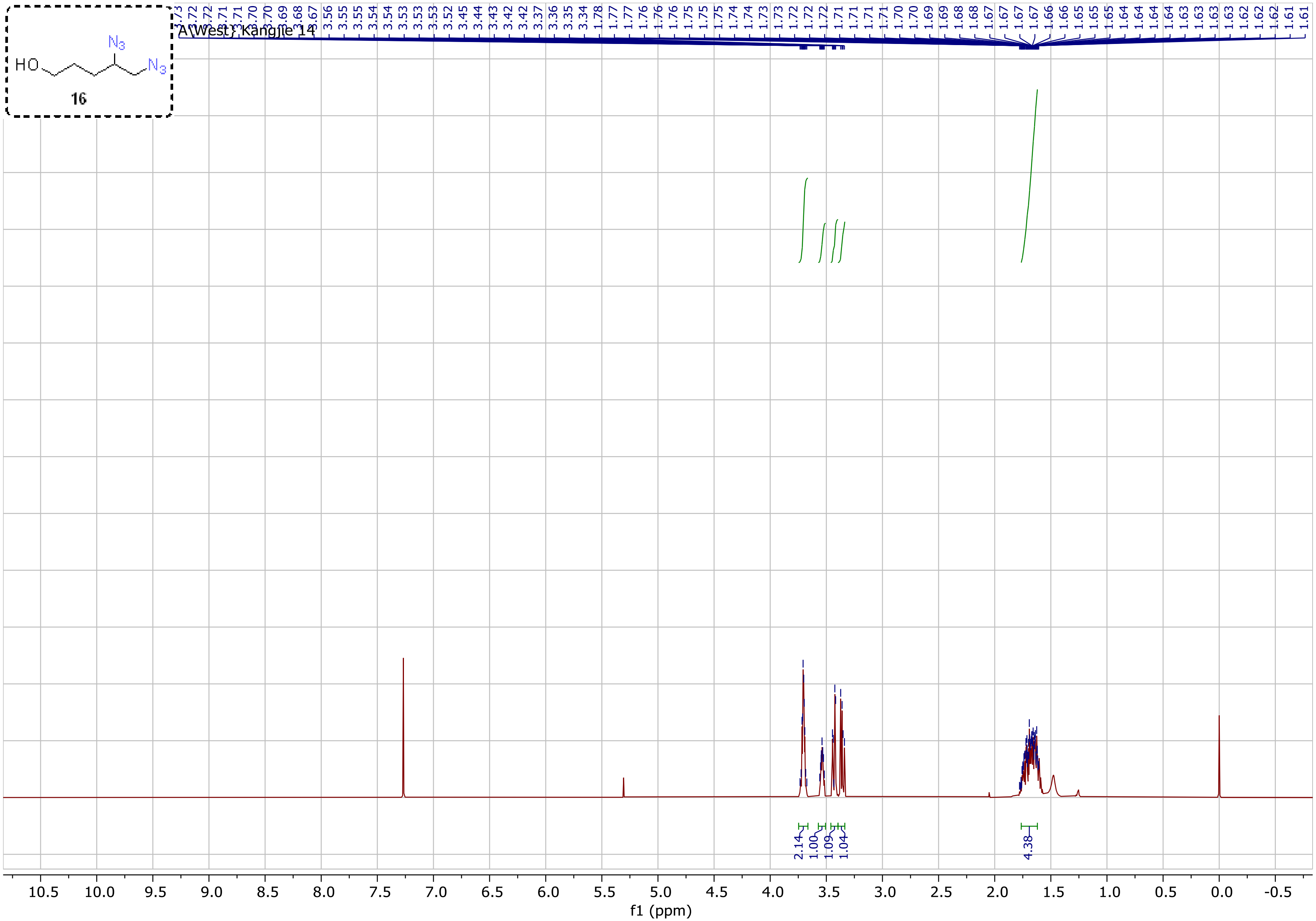
HO
N3
N3
16
A\West} Kangjie 14
2.14
1.00
1.09
1.04
4.38
f1 (ppm)

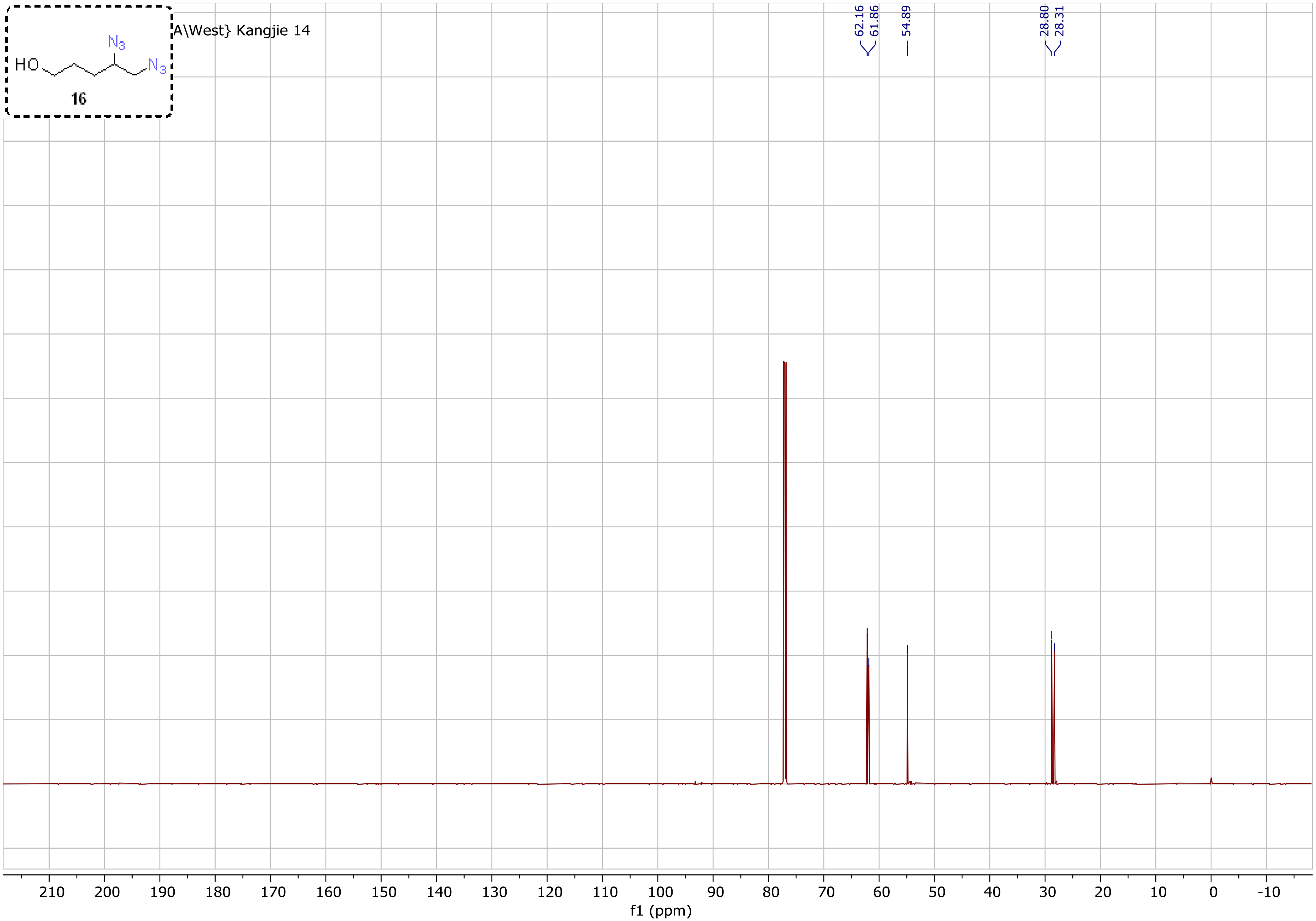
HO
N3
N3
16
A\West} Kangjie 14
62.16
61.86
54.89
28.80
28.31
210 200 190 180 170 160 150 140 130 120 110 100 90 80 70 60 50 40 30 20 10 0 -10
f1 (ppm)

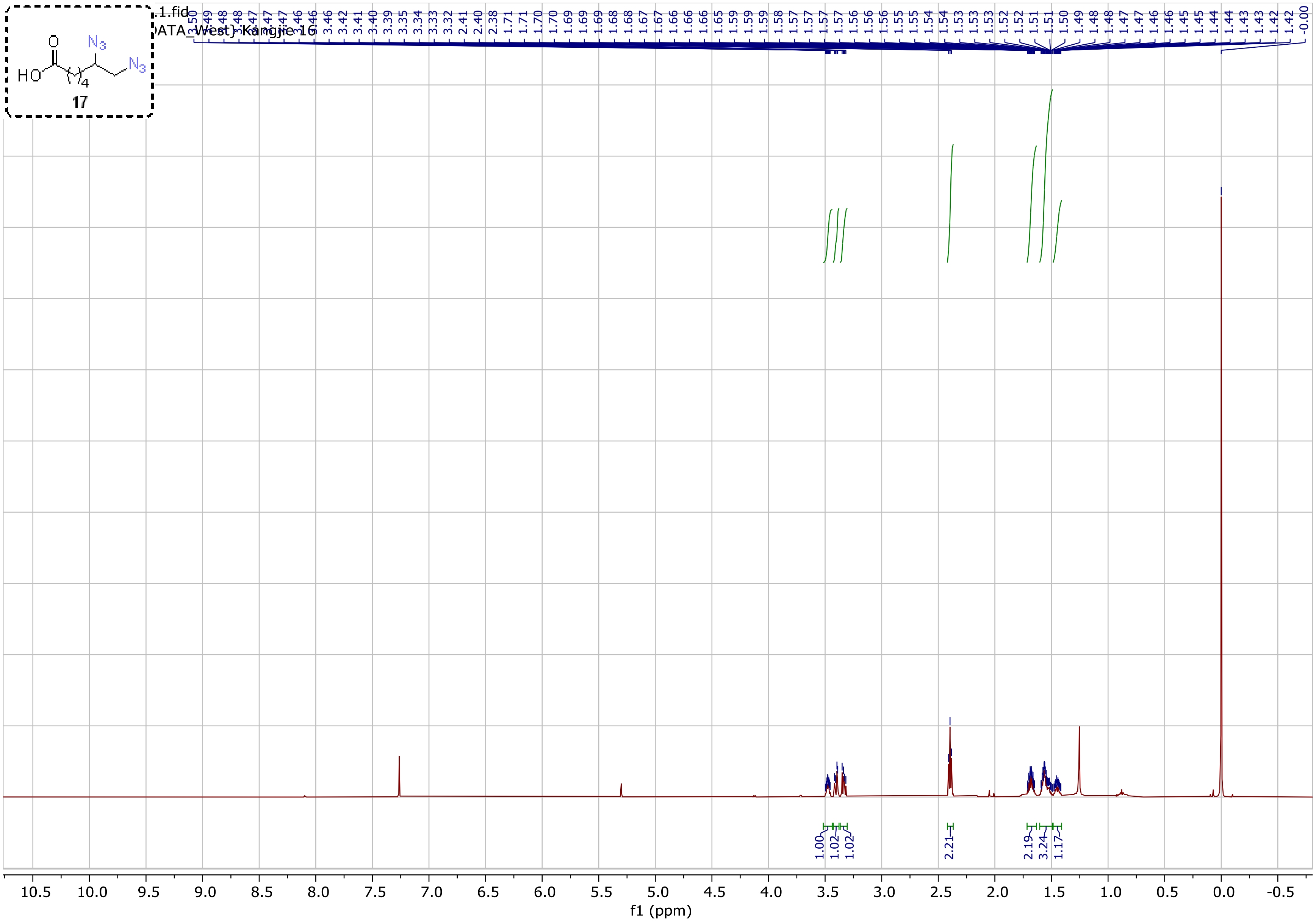
17
1.00
1.02
1.02
2.21
2.19
3.24
1.17
f1 (ppm)

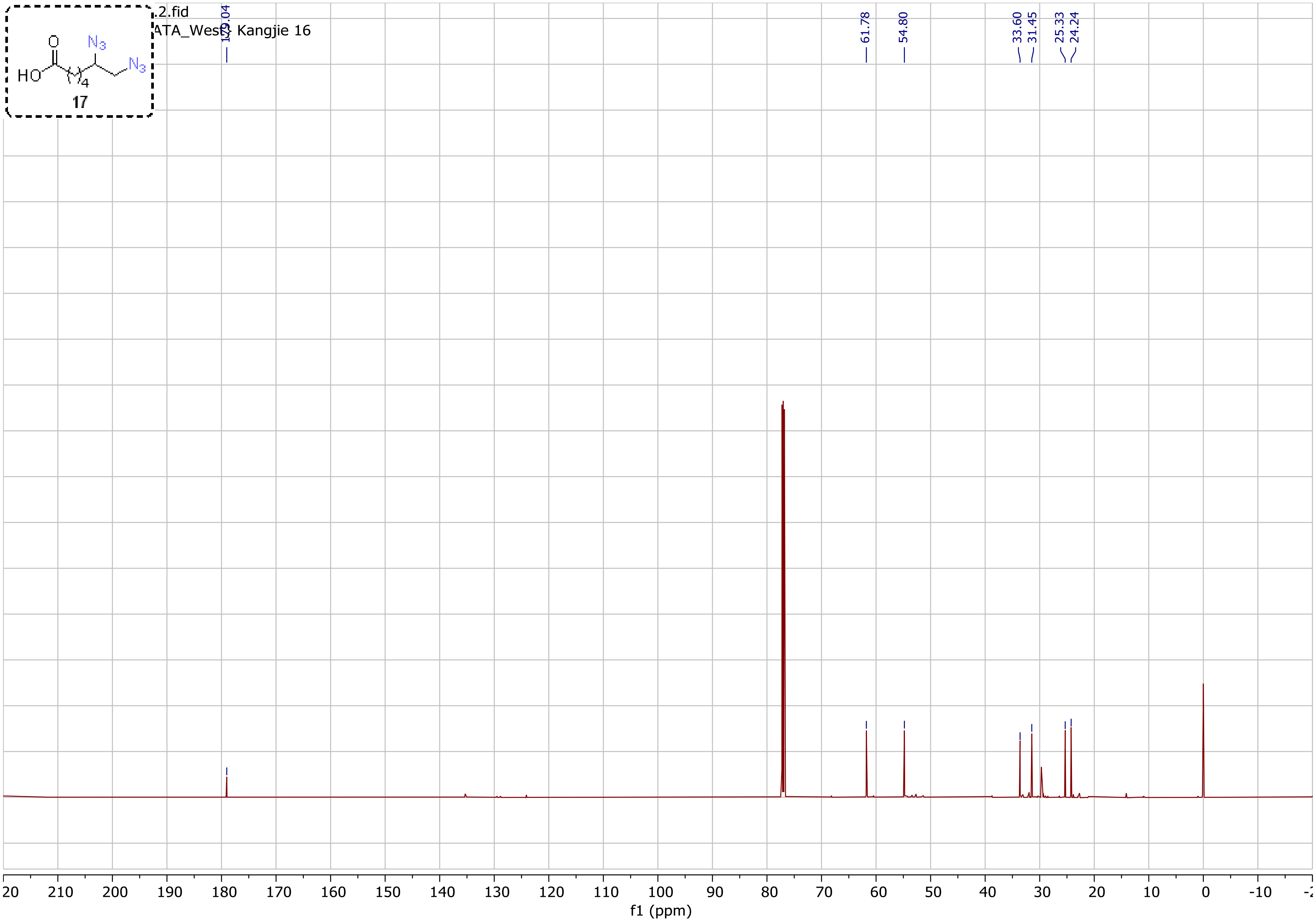
.2.fid
ATA_West) Kangjie 16
17
61.78
54.80
33.60
31.45
25.33
24.24
f1 (ppm)

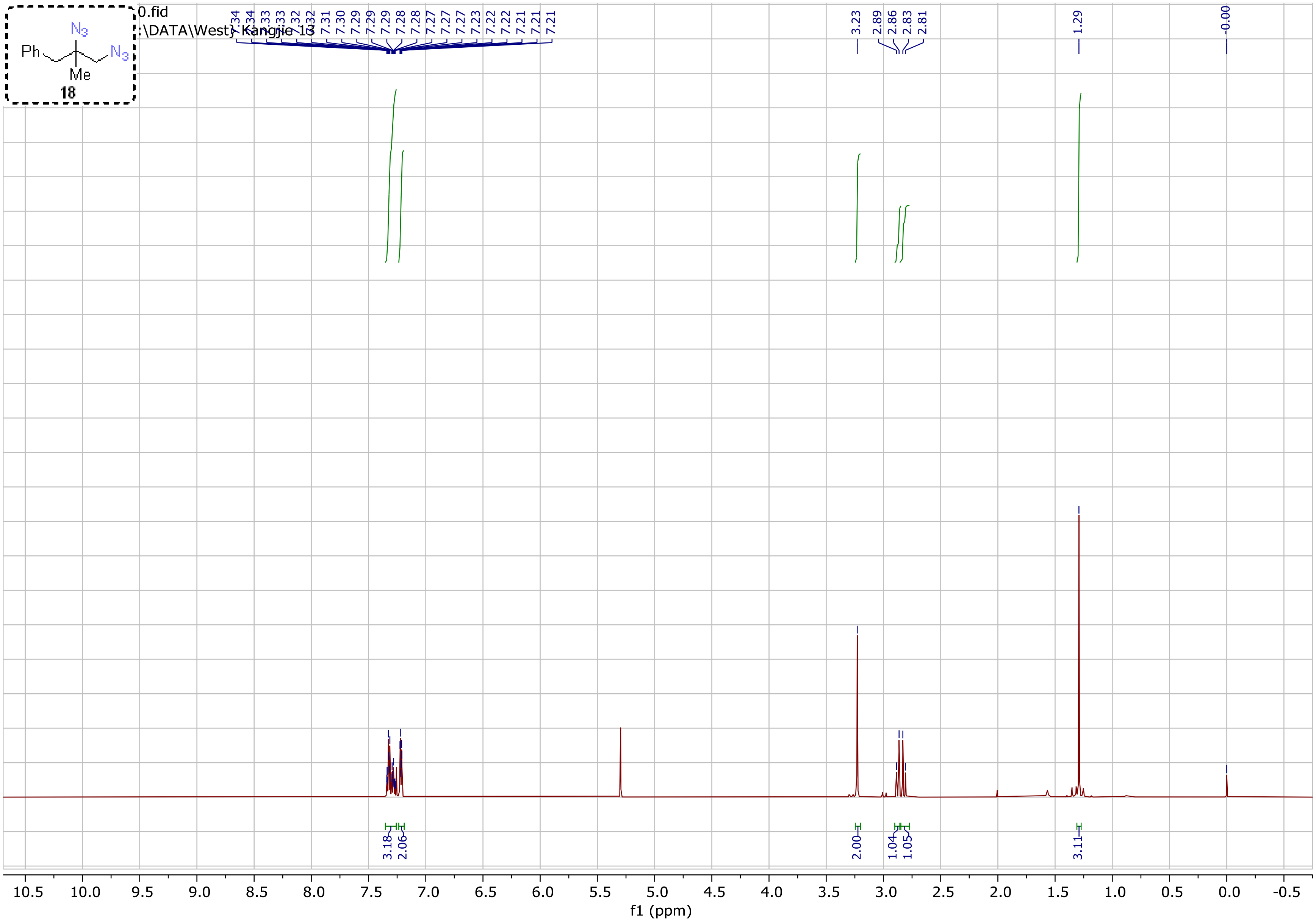
N3
Ph
N3
Me
18
0.fid
:\DATA\West}-Kangjie 13
7.34
7.34
7.33
7.33
7.32
7.32
7.31
7.30
7.29
7.29
7.29
7.28
7.28
7.27
7.27
7.27
7.23
7.22
7.22
7.21
7.21
7.21
3.23
2.89
2.86
2.83
2.81
1.29
-0.00
3.18
2.06
2.00
1.04
1.05
3.11
10.5
10.0
9.5
9.0
8.5
8.0
7.5
7.0
6.5
6.0
5.5
5.0
4.5
4.0
3.5
3.0
2.5
2.0
1.5
1.0
0.5
0.0
-0.5
f1 (ppm)

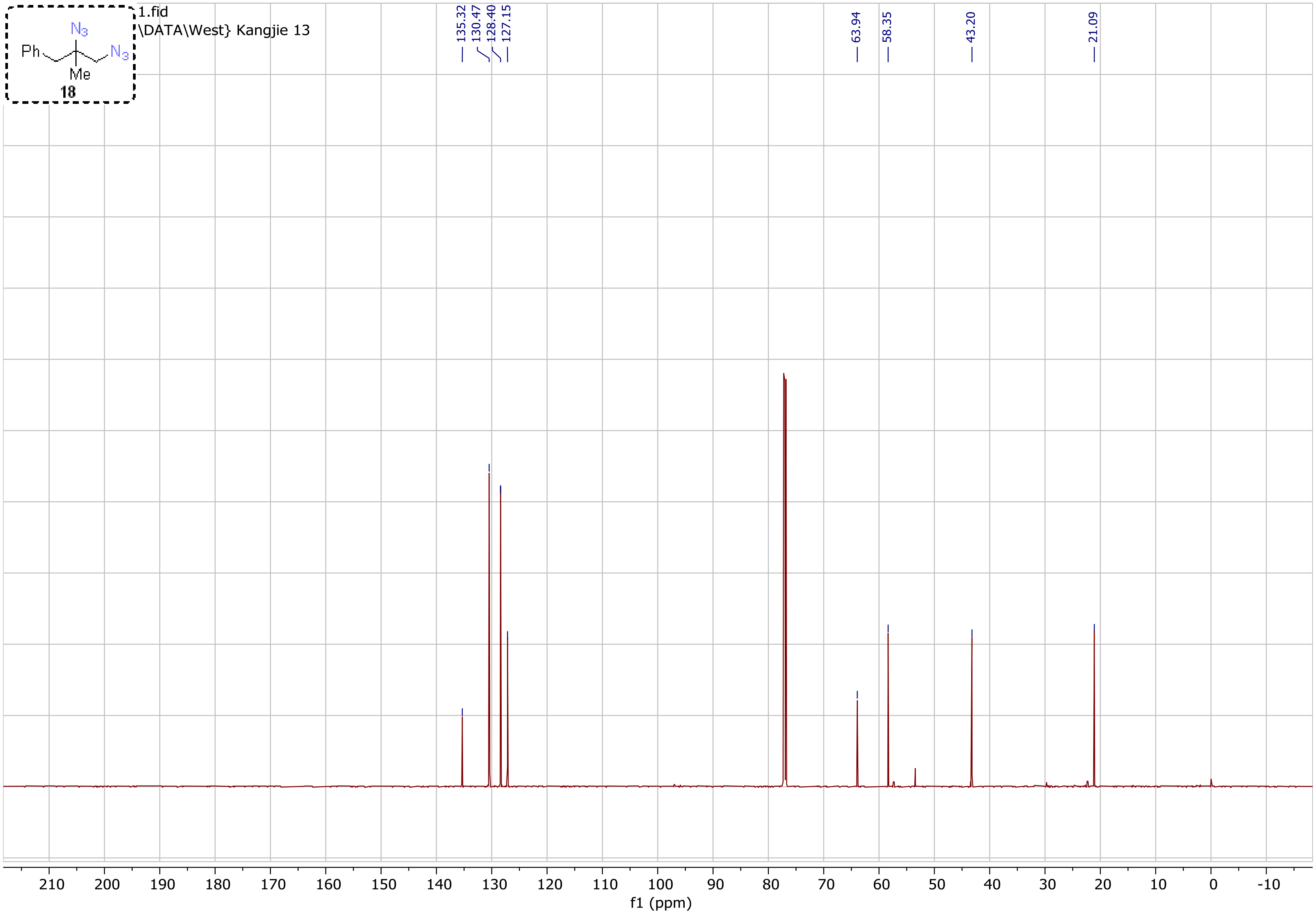

N3
Ph
N3
Me
18
1.fid
\DATA\West} Kangjie 13
135.32
130.47
128.40
127.15
63.94
58.35
43.20
21.09
210
200
190
180
170
160
150
140
130
120
110
100
90
80
70
60
50
40
30
20
10
0
-10
f1 (ppm)

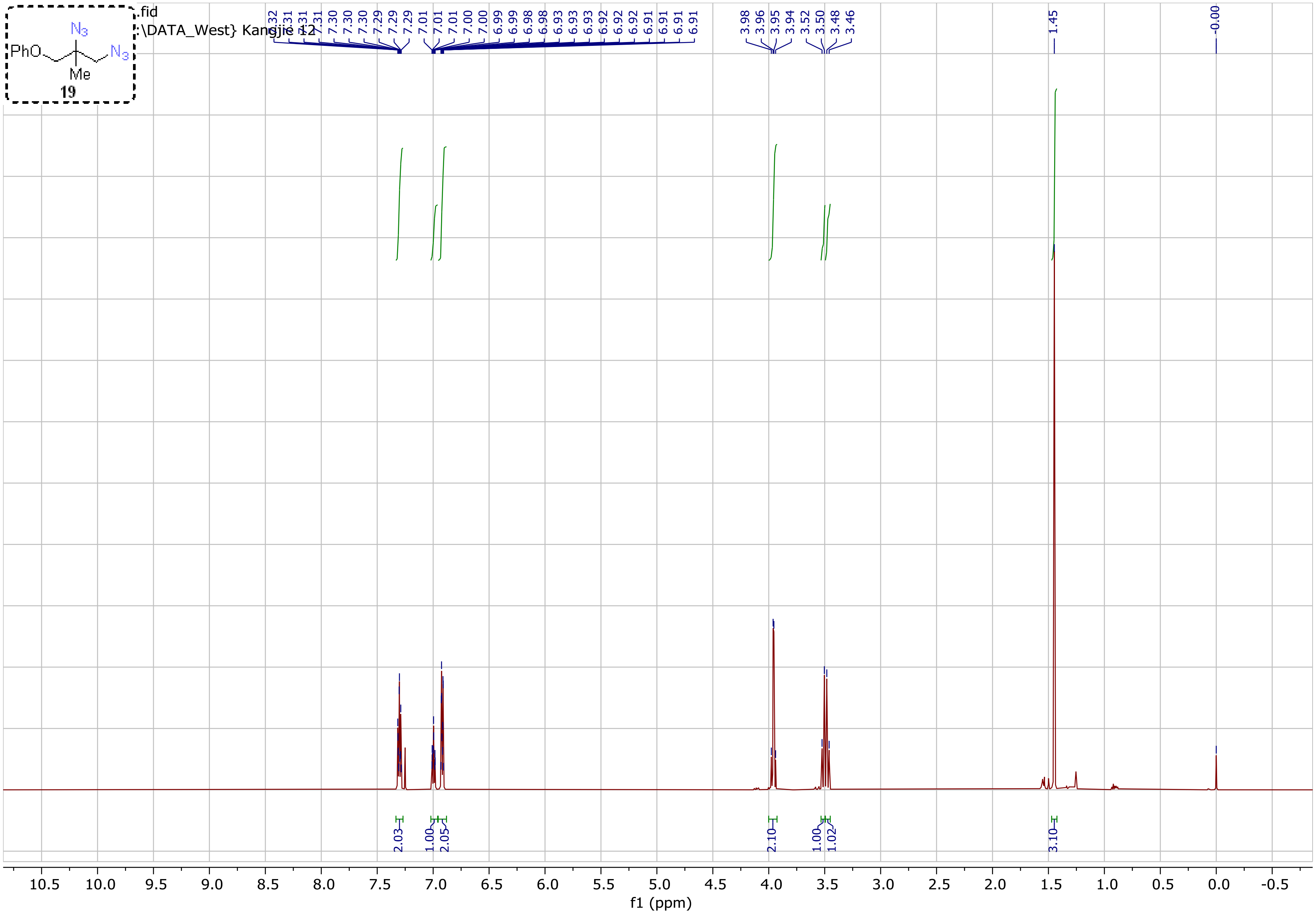

.fid
:\DATA_West} Kangjie 12
PhO
N3
Me
19
7.32 7.31 7.31 7.31 7.30 7.30 7.30 7.29 7.29 7.29 7.01 7.01 7.01 7.00 6.99 6.99 6.98 6.98 6.93 6.93 6.93 6.92 6.92 6.92 6.91 6.91 6.91 6.91
3.98 3.96 3.95 3.94 3.52 3.50 3.48 3.46
1.45
-0.00
2.03 1.00 2.05 2.10 1.00 1.02 3.10
10.5 10.0 9.5 9.0 8.5 8.0 7.5 7.0 6.5 6.0 5.5 5.0 4.5 4.0 3.5 3.0 2.5 2.0 1.5 1.0 0.5 0.0 -0.5
f1 (ppm)

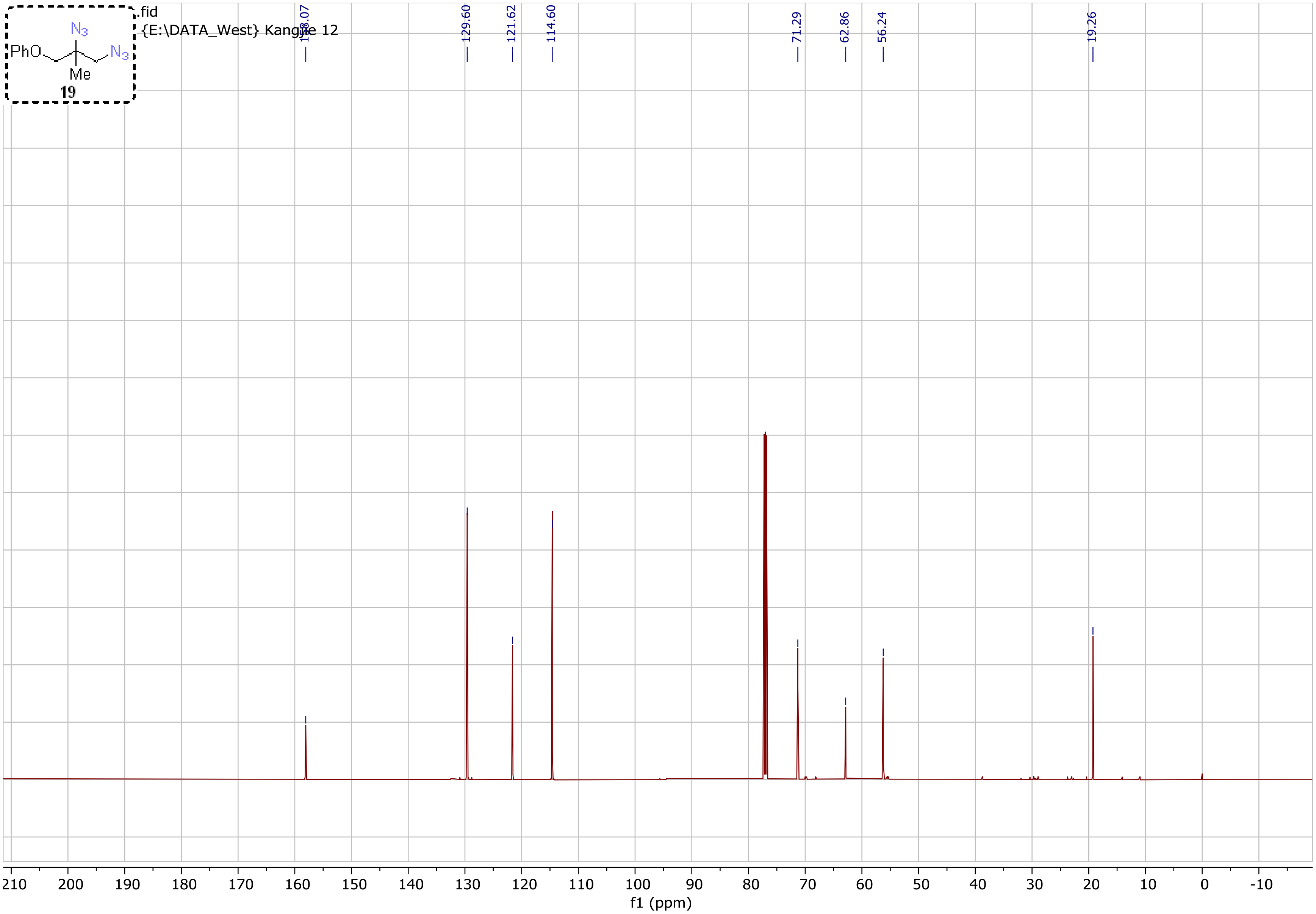

PhO
N3
N3
Me
19
.fid
{E:\DATA_West} Kangjie 12
158.07
129.60
121.62
114.60
71.29
62.86
56.24
19.26
210
200
190
180
170
160
150
140
130
120
110
100
90
80
70
60
50
40
30
20
10
0
-10
f1 (ppm)

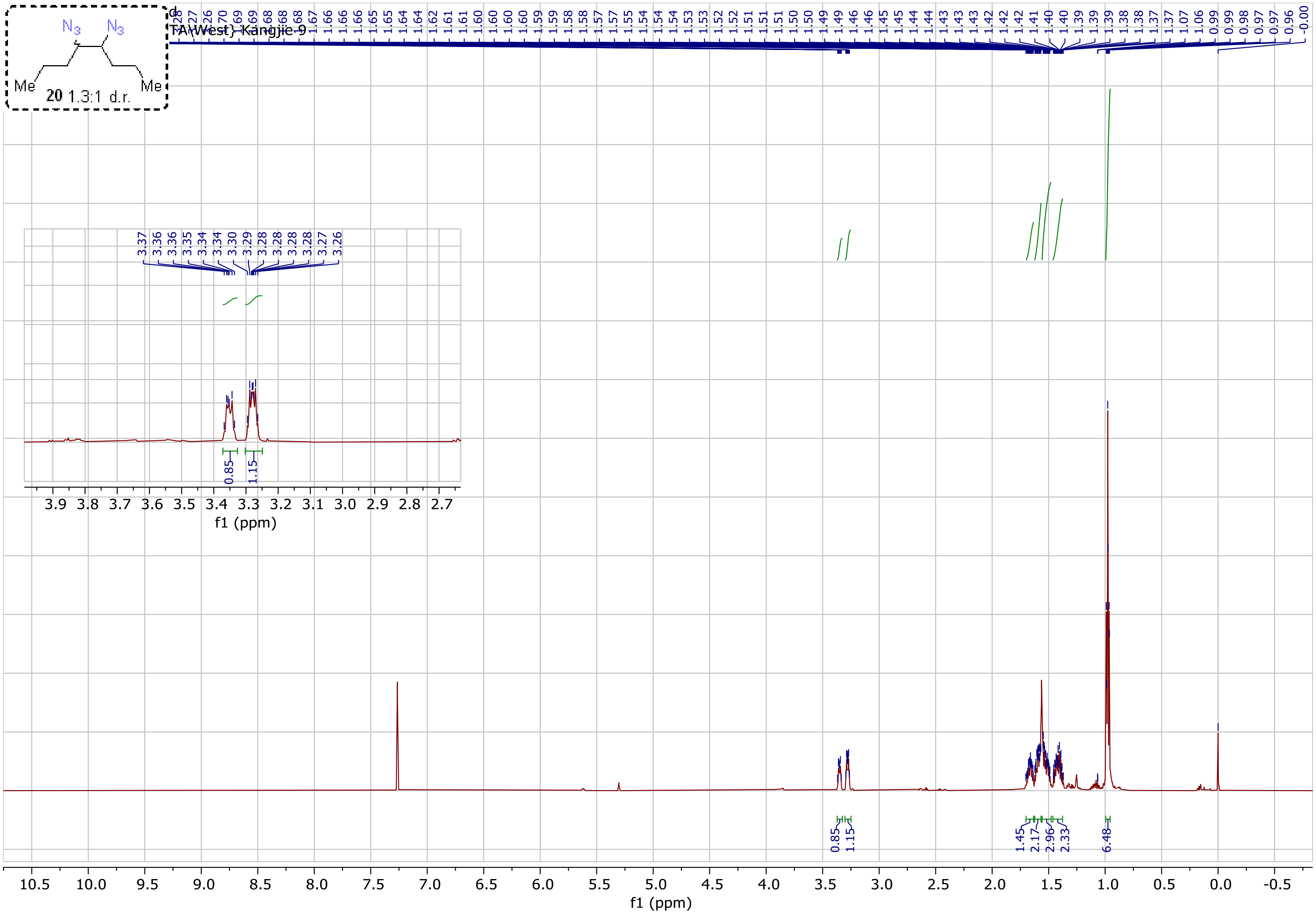

N3
N3
Me
Me
20 1.3:1 d.r.
TA(West) Kangjie-9
f1 (ppm)

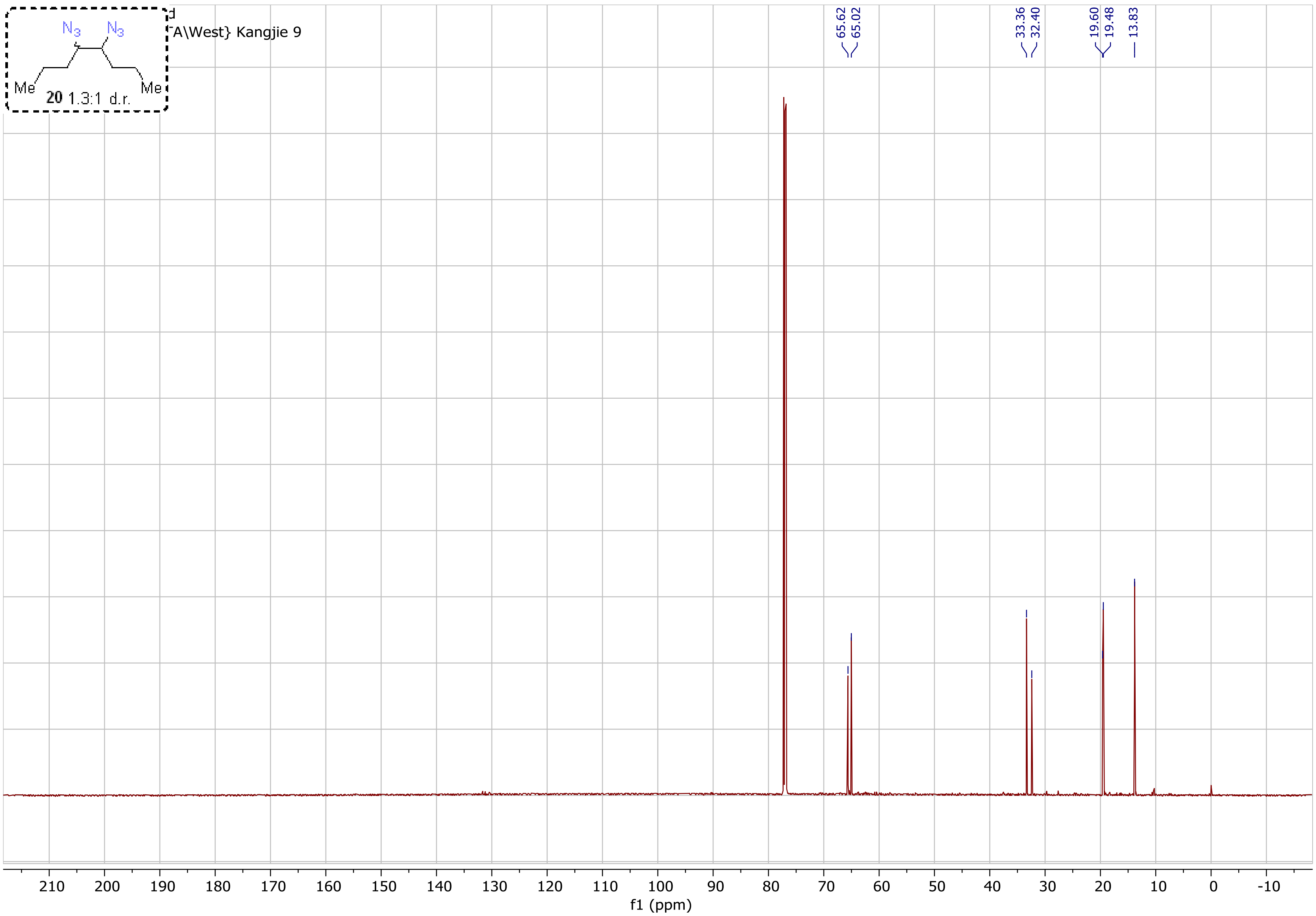
A\West} Kangjie 9
N3
N3
Me
Me
20 1.3:1 d.r.
65.62
65.02
33.36
32.40
19.60
19.48
13.83
210
200
190
180
170
160
150
140
130
120
110
100
90
80
70
60
50
40
30
20
10
0
-10
f1 (ppm)

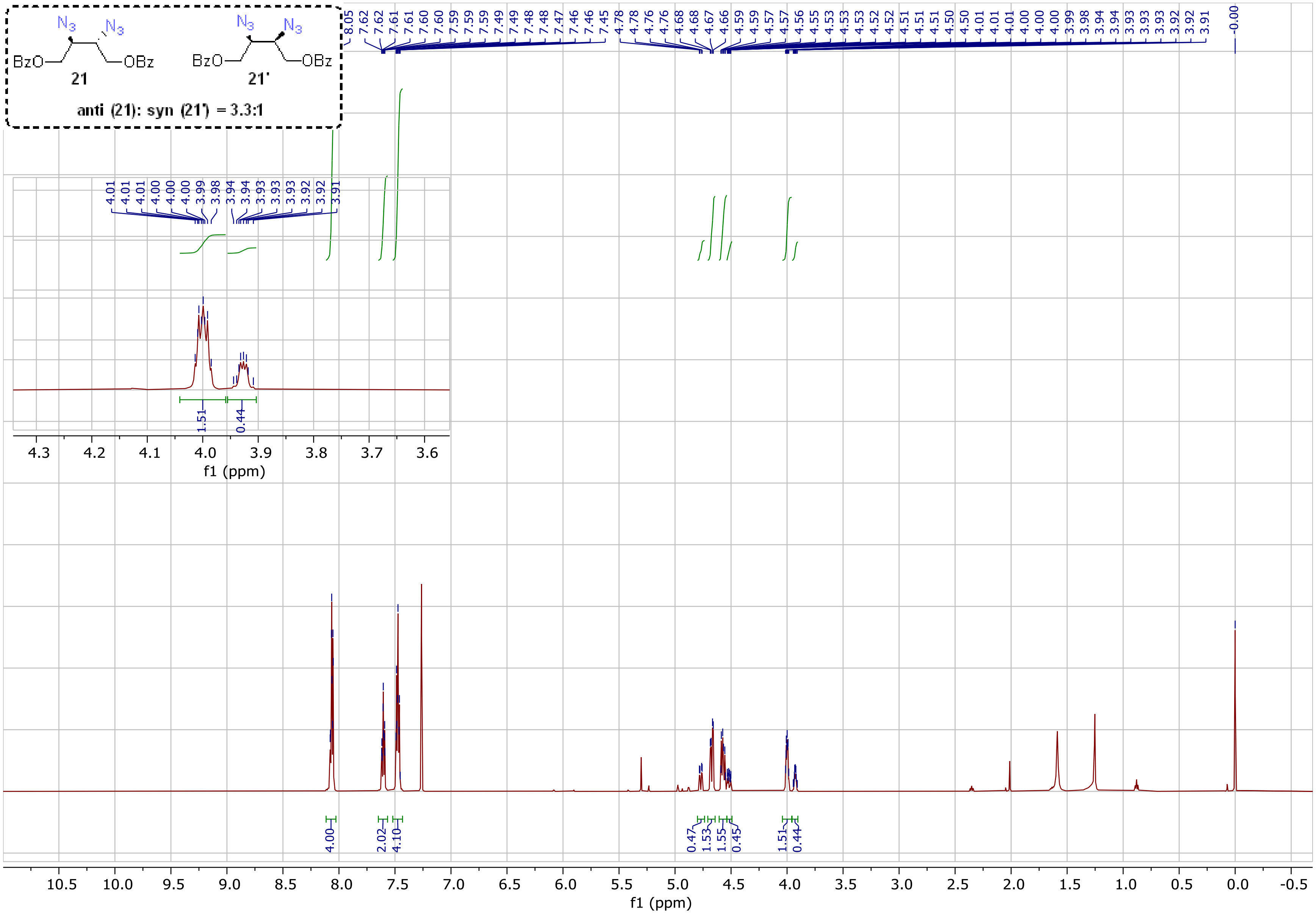

BzO
N3
OBz
21
21'
anti (21): syn (21') = 3.3:1
f1 (ppm)

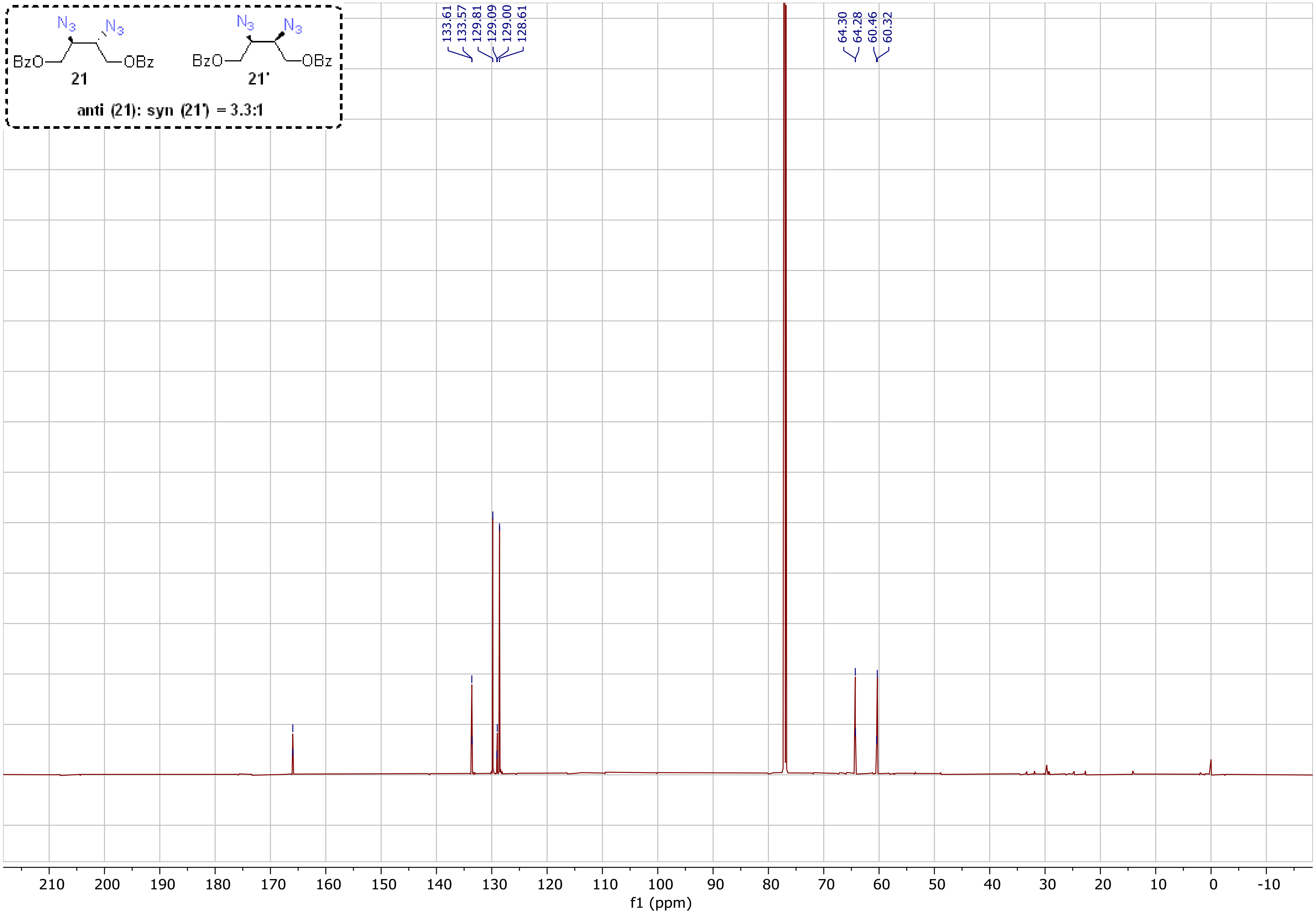

N3
N3
BzO
OBz
21
21'
anti (21): syn (21') = 3.3:1
133.61
133.57
129.81
129.09
129.00
128.61
64.30
64.28
60.46
60.32
210 200 190 180 170 160 150 140 130 120 110 100 90 80 70 60 50 40 30 20 10 0 -10
f1 (ppm)

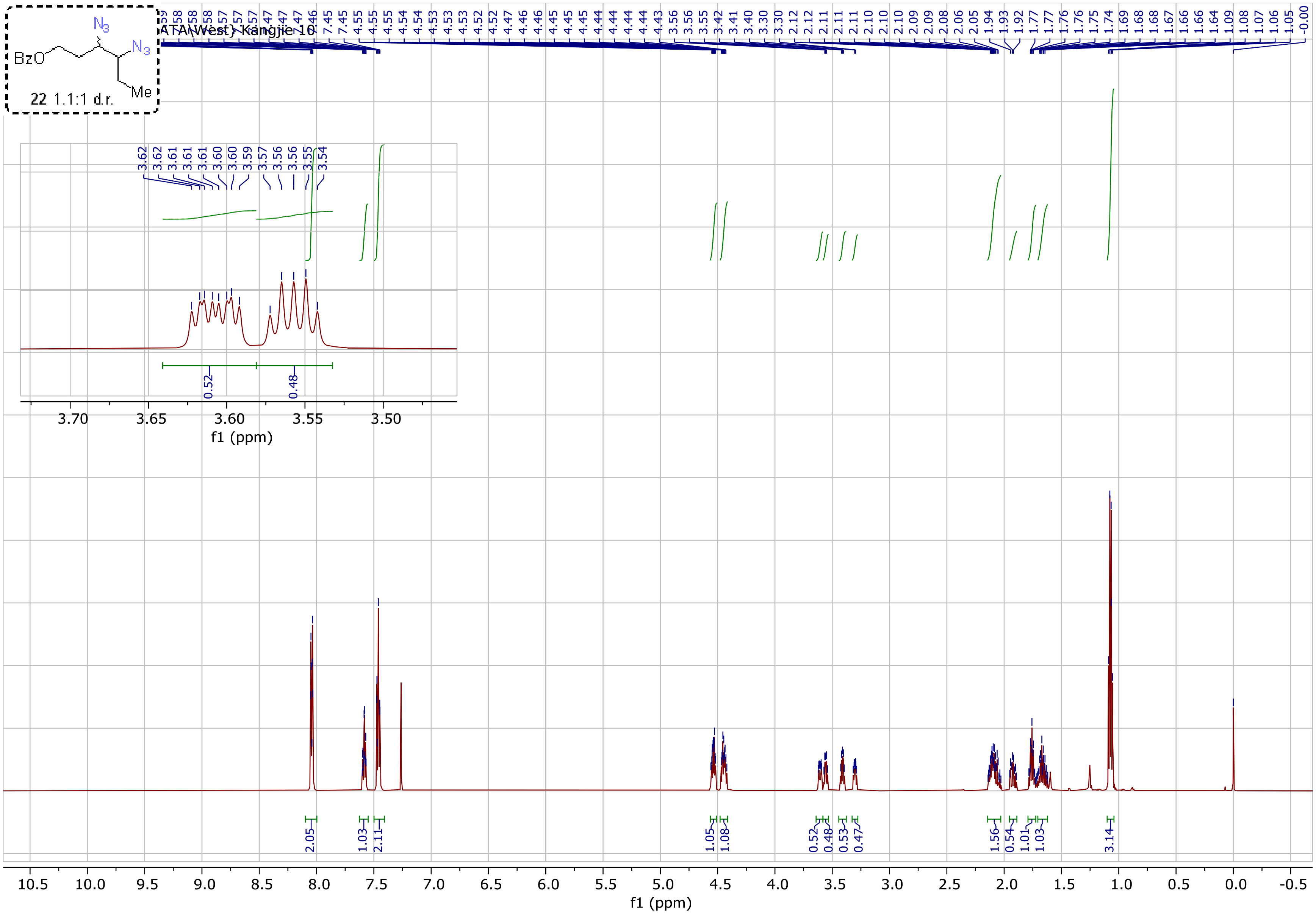

BzO
N3
N3
Me
22 1.1:1 d.r.
ATA\West} Kangjie 16
f1 (ppm)

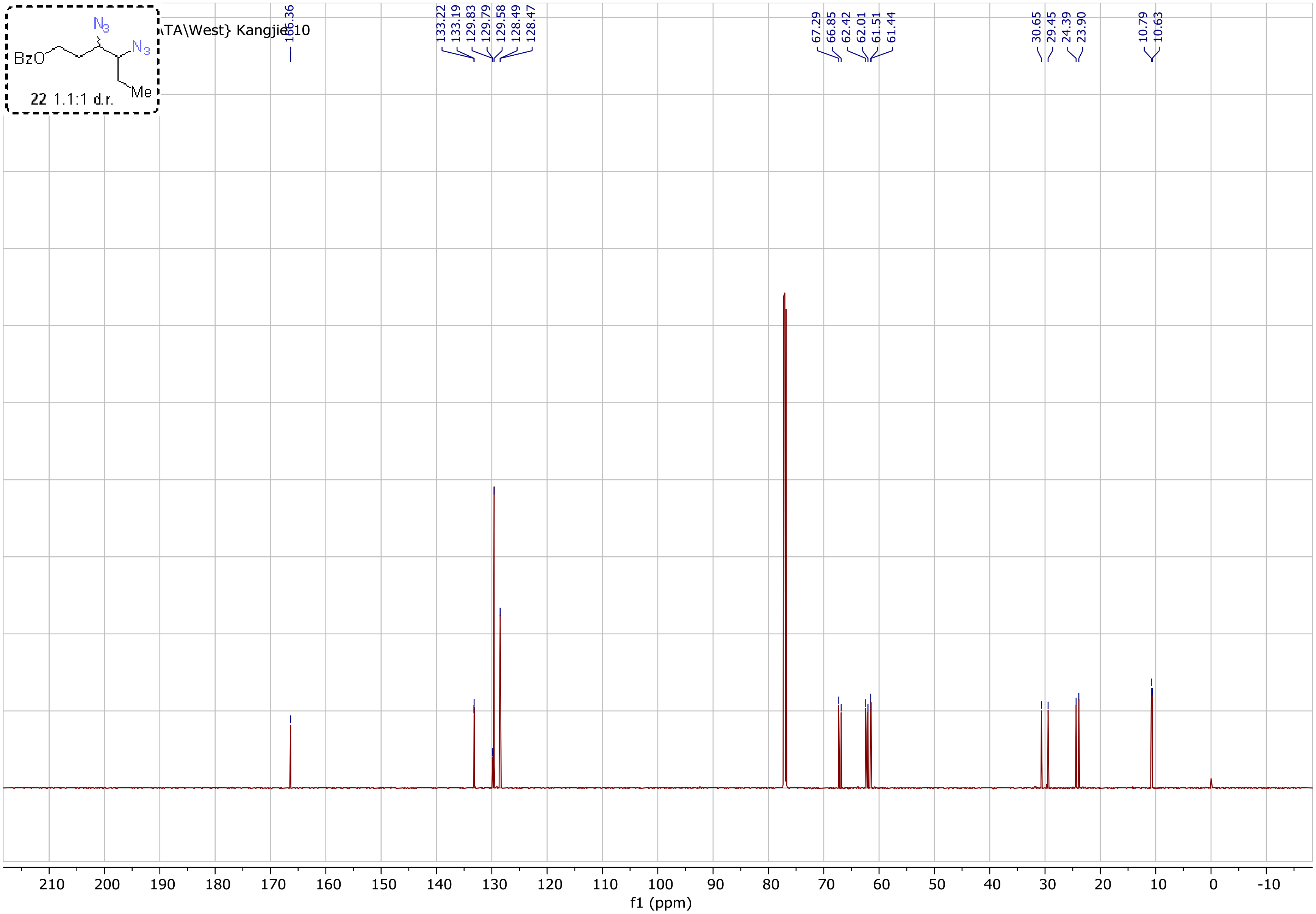
N3
N3
BzO
Me
22 1.1:1 d.r.
TA\West} Kangjie610
166.36
133.22
133.19
129.83
129.79
129.58
128.49
128.47
67.29
66.85
62.42
62.01
61.51
61.44
30.65
29.45
24.39
23.90
10.79
10.63
210
200
190
180
170
160
150
140
130
120
110
100
90
80
70
60
50
40
30
20
10
0
-10
f1 (ppm)

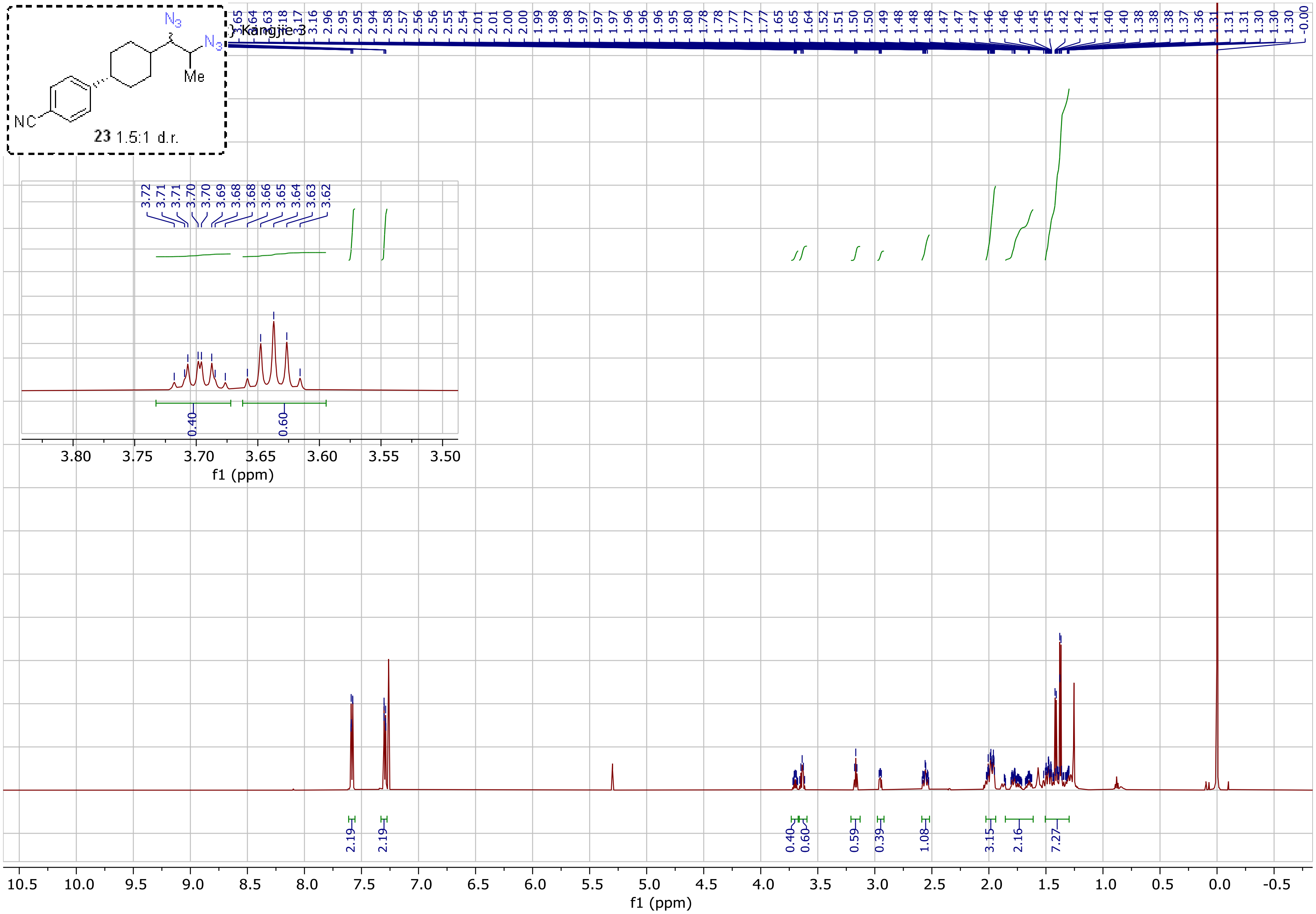
N3
N3
Me
NC
23 1.5:1 d.r.
Kangjie 3
0.40
0.60
f1 (ppm)
2.19
2.19
0.40
0.60
0.59
0.39
1.08
3.15
2.16
7.27
f1 (ppm)

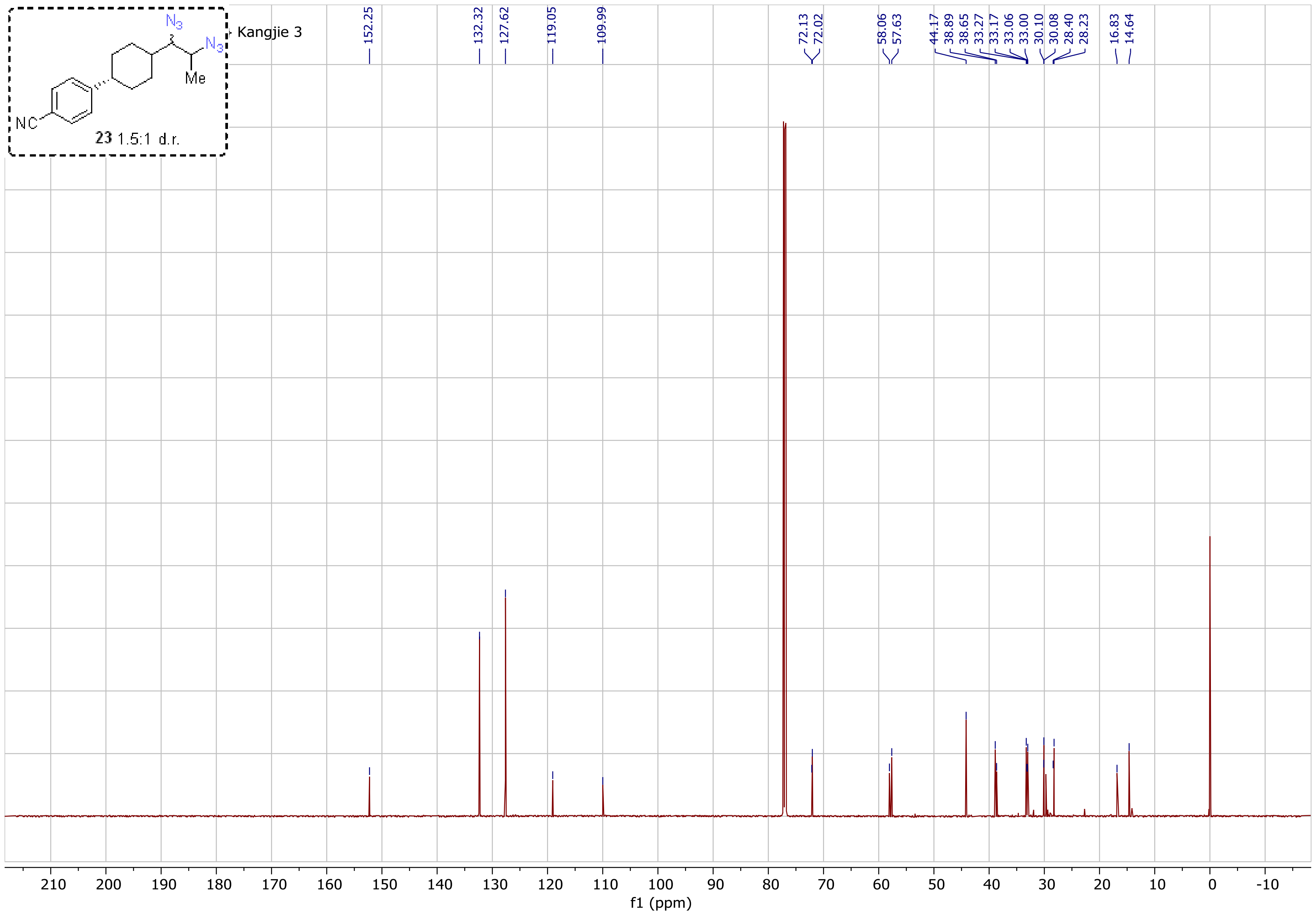
N3
N3
Me
NC
23 1.5:1 d.r.
Kangjie 3
152.25
132.32
127.62
119.05
109.99
72.13
72.02
58.06
57.63
44.17
38.89
38.65
33.27
33.17
33.06
33.00
30.10
30.08
28.40
28.23
16.83
14.64
210 200 190 180 170 160 150 140 130 120 110 100 90 80 70 60 50 40 30 20 10 0 -10
f1 (ppm)

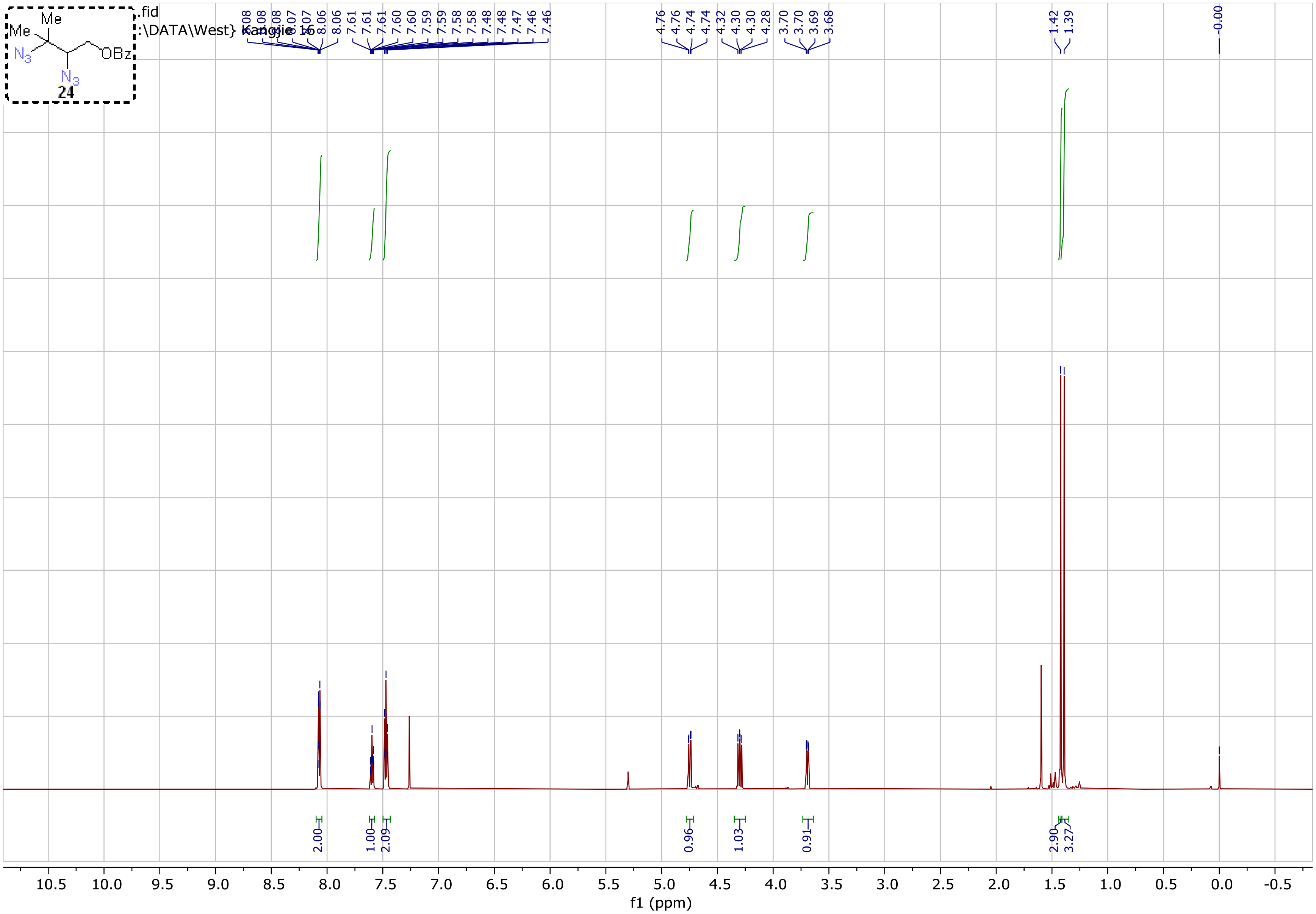
Me
Me
N3
OBz
N3
24
.fid
:\DATA\West} Kangjie 16
8.08 8.08 8.08 8.07 8.07 8.06 7.61 7.61 7.61 7.60 7.60 7.59 7.59 7.58 7.58 7.48 7.48 7.47 7.46 7.46
4.76 4.76 4.74 4.74 4.32 4.30 4.30 4.28 3.70 3.70 3.69 3.68
1.42 1.39
-0.00
2.00
1.00
2.09
0.96
1.03
0.91
2.90
3.27
10.5 10.0 9.5 9.0 8.5 8.0 7.5 7.0 6.5 6.0 5.5 5.0 4.5 4.0 3.5 3.0 2.5 2.0 1.5 1.0 0.5 0.0 -0.5
f1 (ppm)

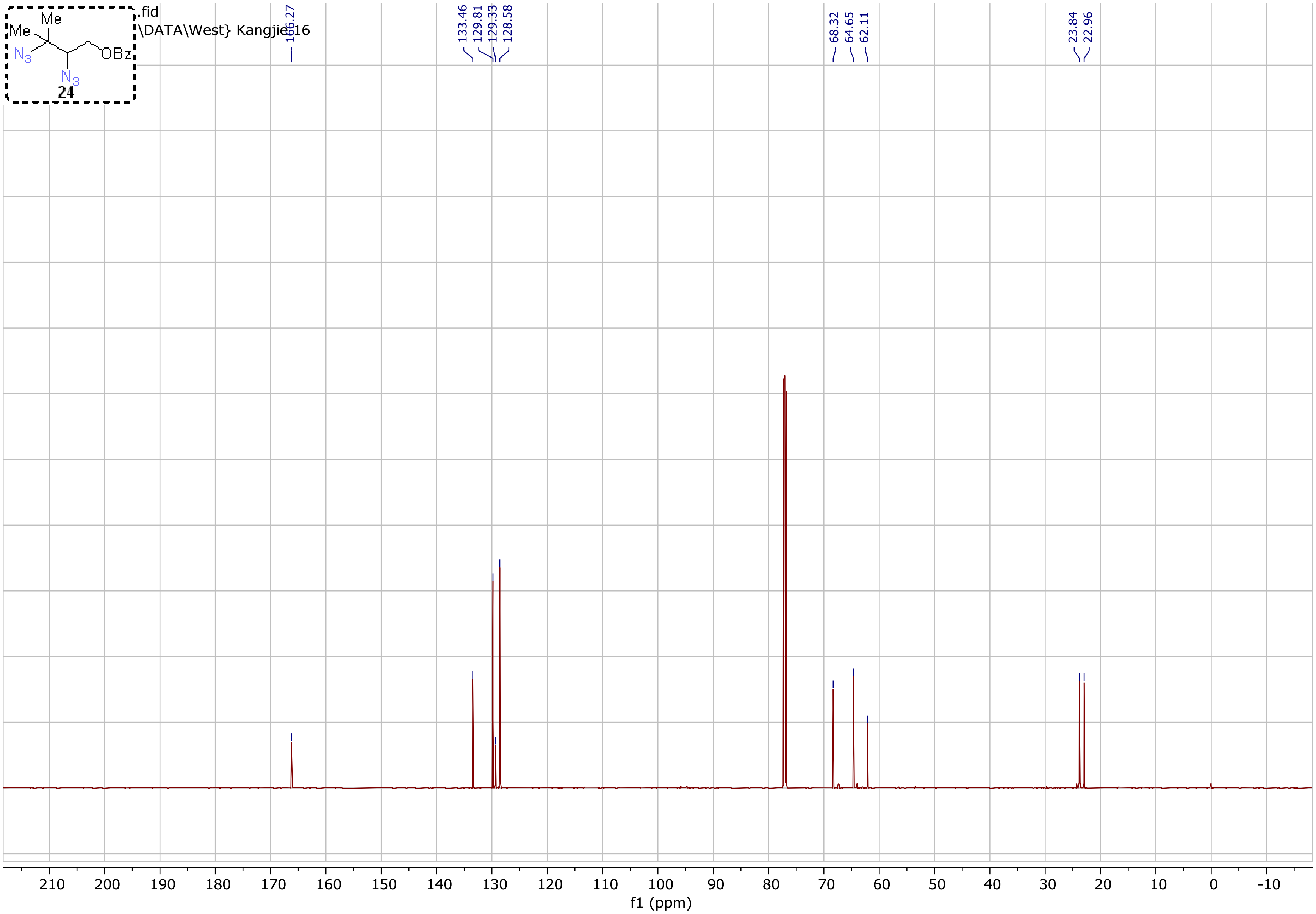

Me
Me
N3
N3
OBz
24
.fid
\DATA\West} Kangjie-16
166.27
133.46
129.81
129.33
128.58
68.32
64.65
62.11
23.84
22.96
210
200
190
180
170
160
150
140
130
120
110
100
90
80
70
60
50
40
30
20
10
0
-10
f1 (ppm)

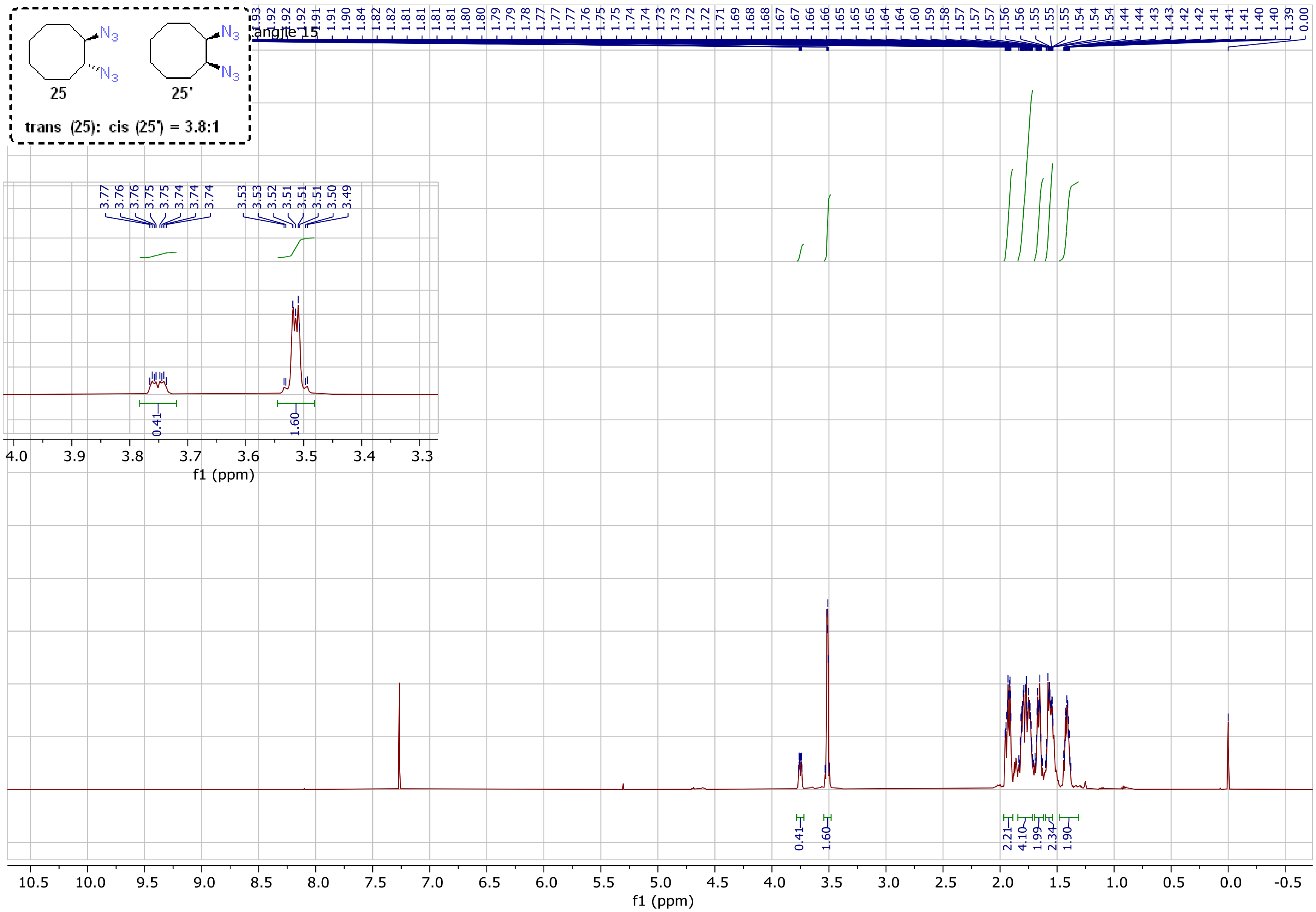
25
25'
trans (25): cis (25') = 3.8:1
angjie 15
0.41
1.60
2.21
4.10
1.99
2.34
1.90
f1 (ppm)

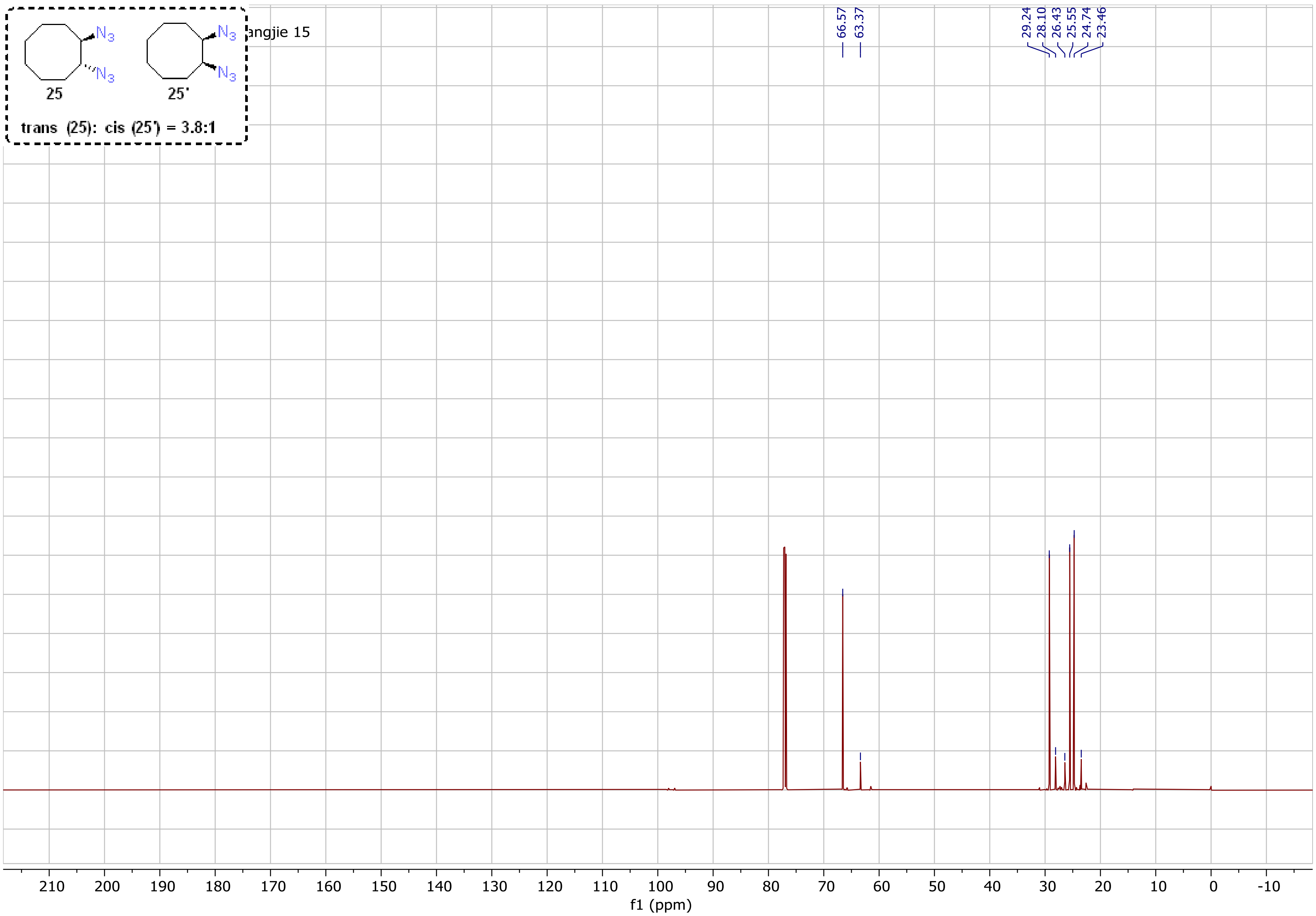
angjie 15
N_3
N_3
N_3
N_3
25
25'
trans (25): cis (25') = 3.8:1
66.57
63.37
29.24
28.10
26.43
25.55
24.74
23.46
210
200
190
180
170
160
150
140
130
120
110
100
90
80
70
60
50
40
30
20
10
0
-10
f1 (ppm)

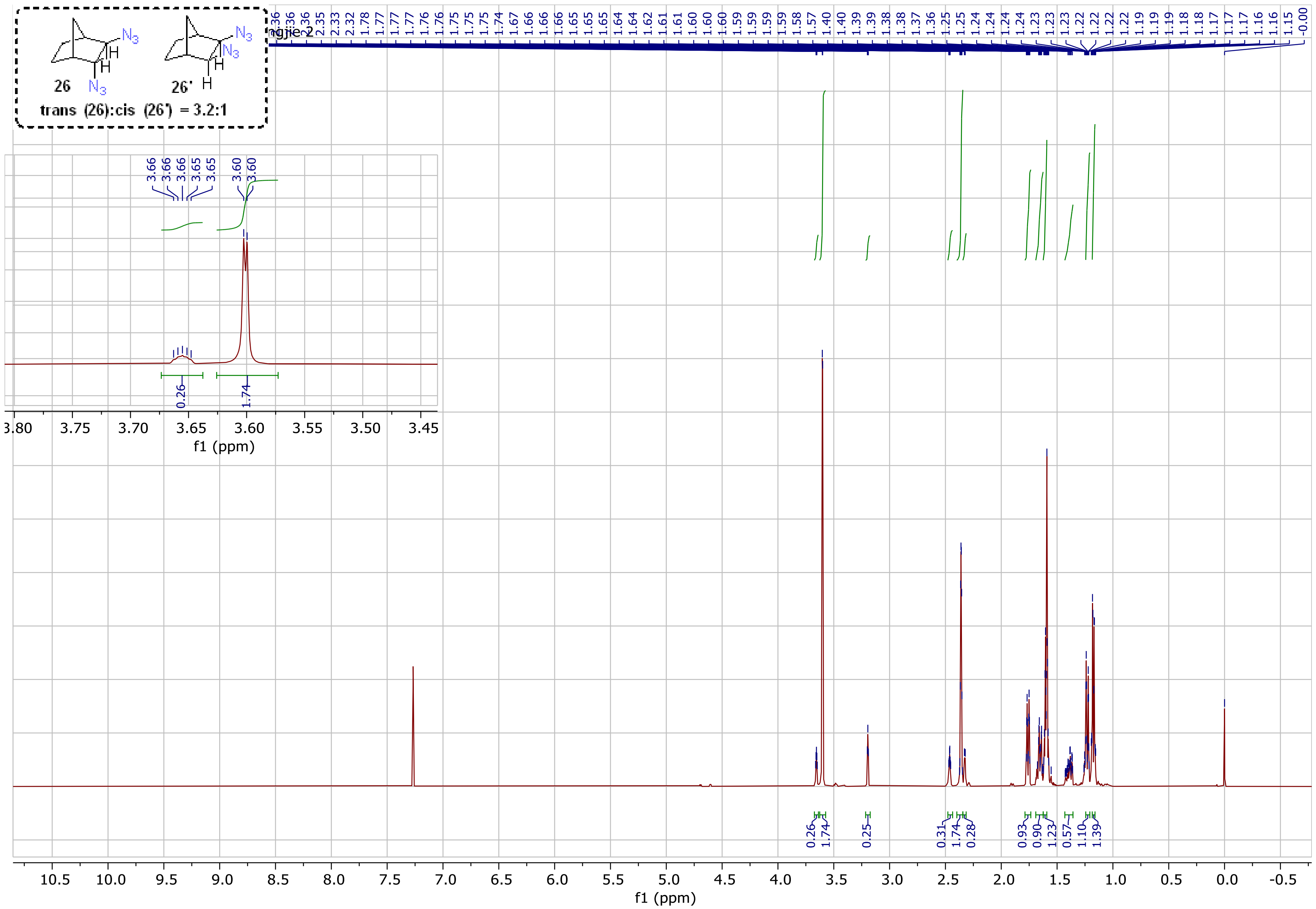
26
26'
trans (26):cis (26') = 3.2:1
f1 (ppm)

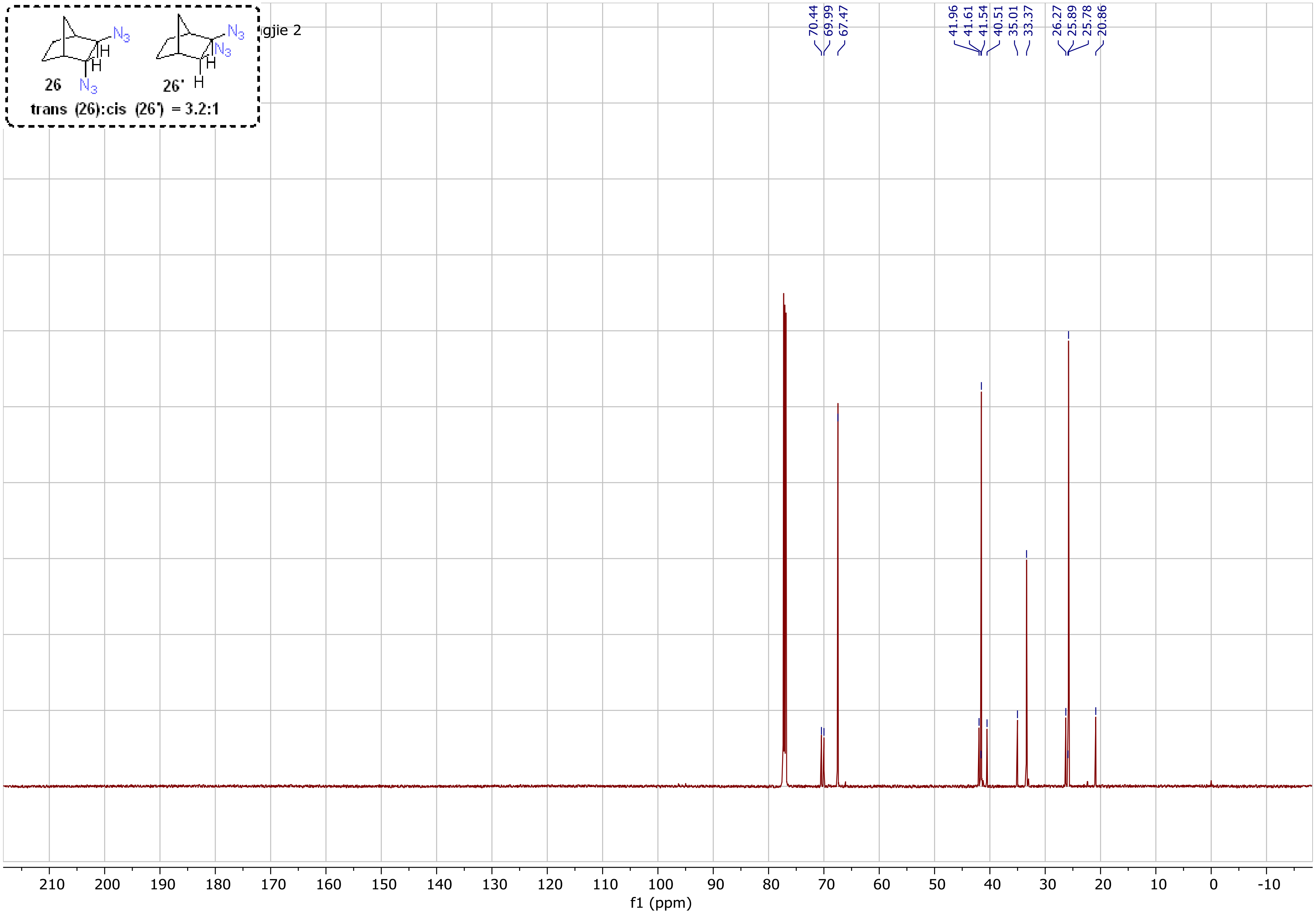
N3
H
H
26
N3
N3
N3
H
26'
H
trans (26):cis (26') = 3.2:1
gjie 2
70.44
69.99
67.47
41.96
41.61
41.54
40.51
35.01
33.37
26.27
25.89
25.78
20.86
210
200
190
180
170
160
150
140
130
120
110
100
90
80
70
60
50
40
30
20
10
0
-10
f1 (ppm)

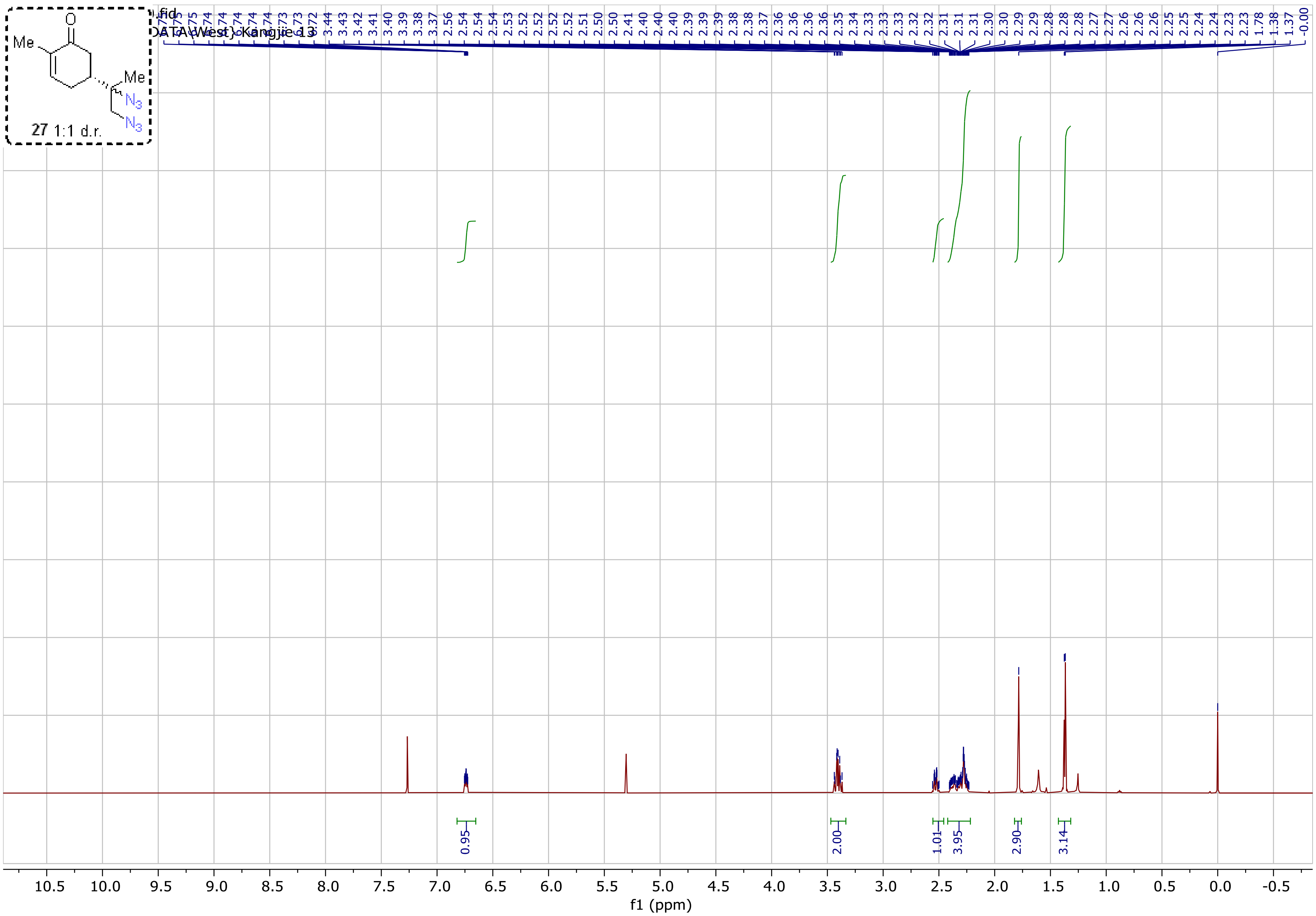

Me
O
Me
N3
N3
27 1:1 d.r.
f1 (ppm)

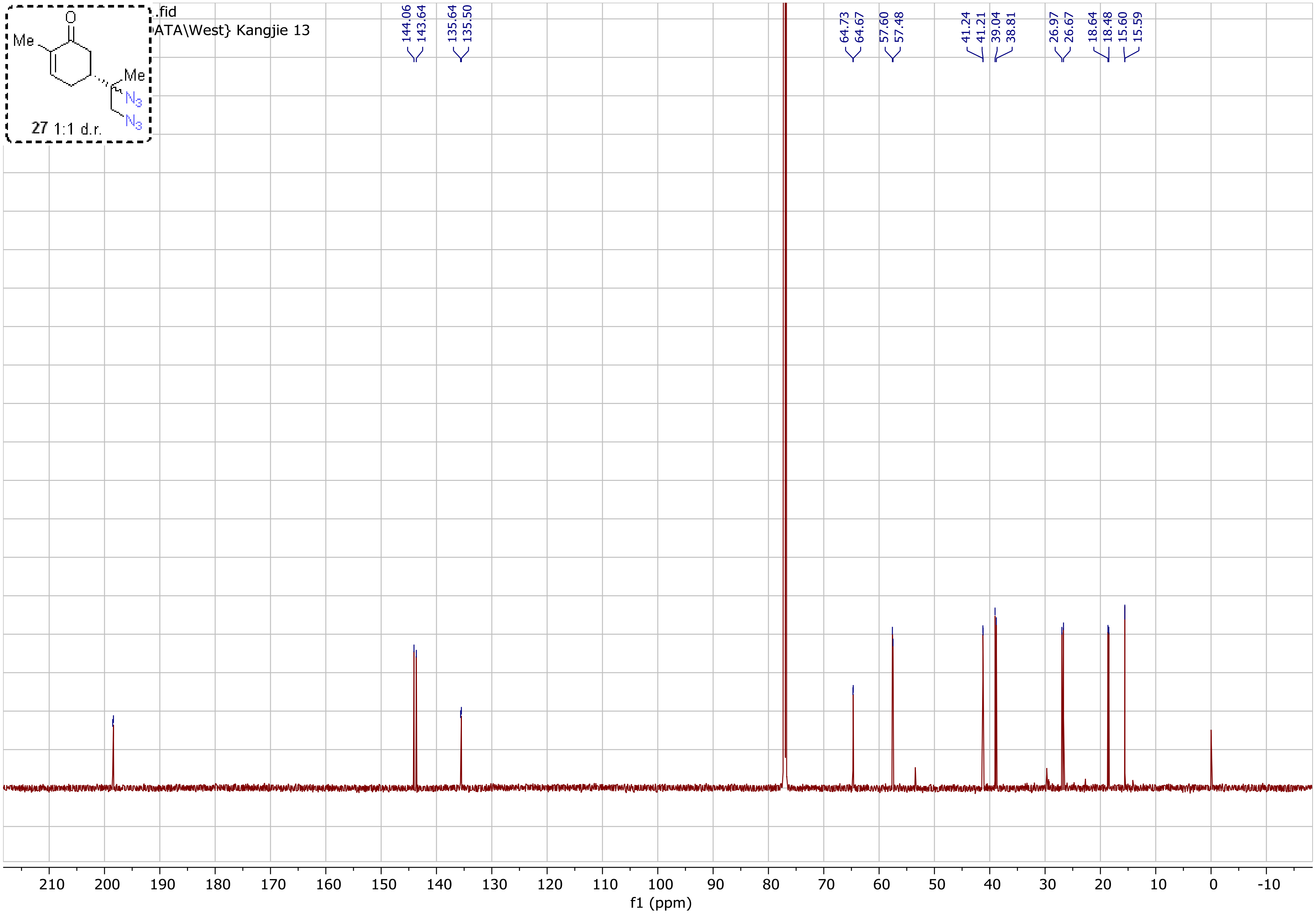

.fid
ATA\West} Kangjie 13
27 1:1 d.r.
Me
O
Me
N3
N3
144.06
143.64
135.64
135.50
64.73
64.67
57.60
57.48
41.24
41.21
39.04
38.81
26.97
26.67
18.64
18.48
15.60
15.59
210
200
190
180
170
160
150
140
130
120
110
100
90
80
70
60
50
40
30
20
10
0
-10
f1 (ppm)

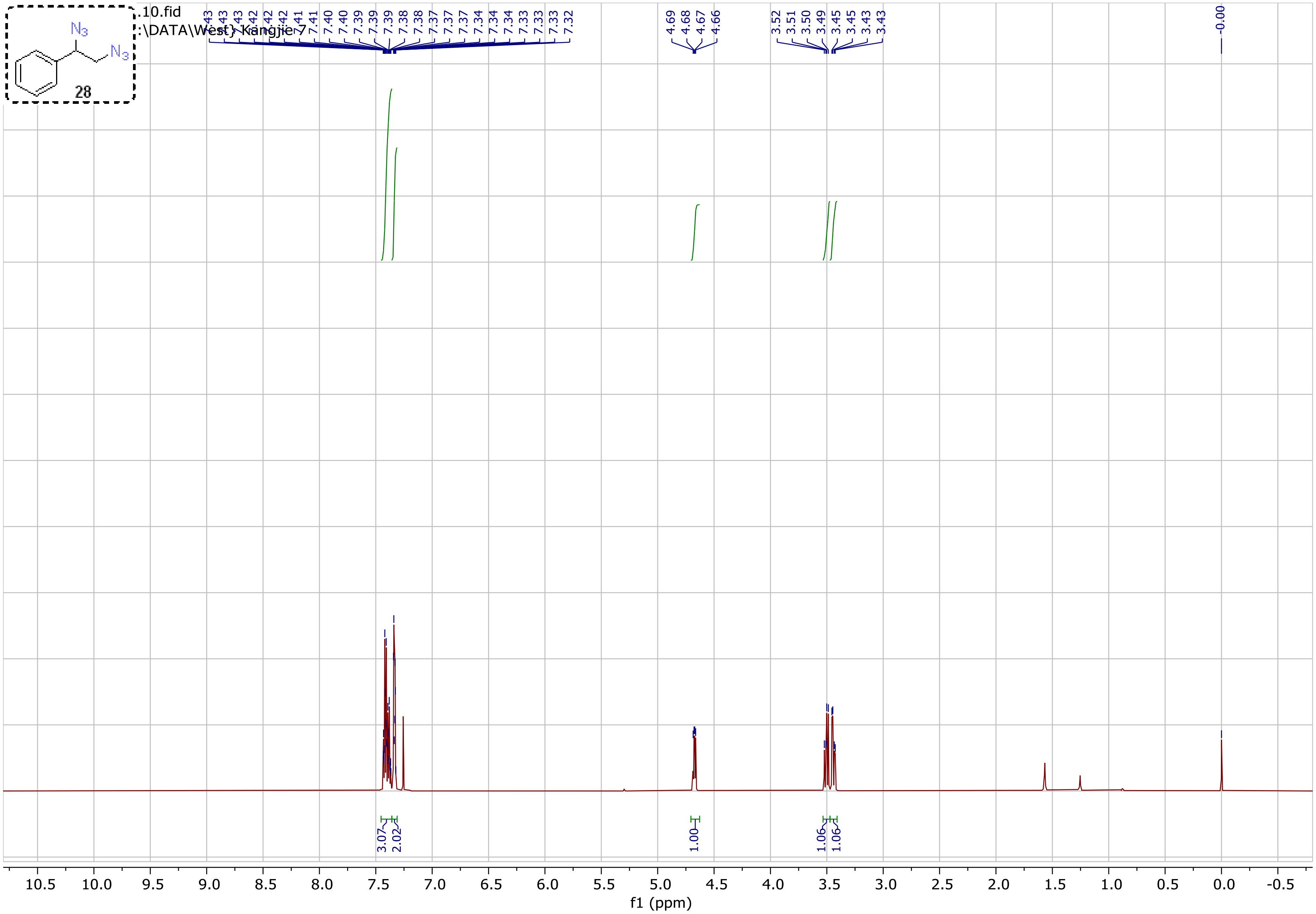

N3
N3
28
.10.fid
:\DATA\West} Kangjie 7
7.43 7.43 7.43 7.42 7.42 7.41 7.41 7.40 7.40 7.39 7.39 7.39 7.38 7.38 7.37 7.37 7.37 7.34 7.34 7.34 7.33 7.33 7.33 7.32
4.69 4.68 4.67 4.66
3.52 3.51 3.50 3.49 3.45 3.45 3.43 3.43
-0.00
3.07
2.02
1.00
1.06
1.06
10.5 10.0 9.5 9.0 8.5 8.0 7.5 7.0 6.5 6.0 5.5 5.0 4.5 4.0 3.5 3.0 2.5 2.0 1.5 1.0 0.5 0.0 -0.5
f1 (ppm)

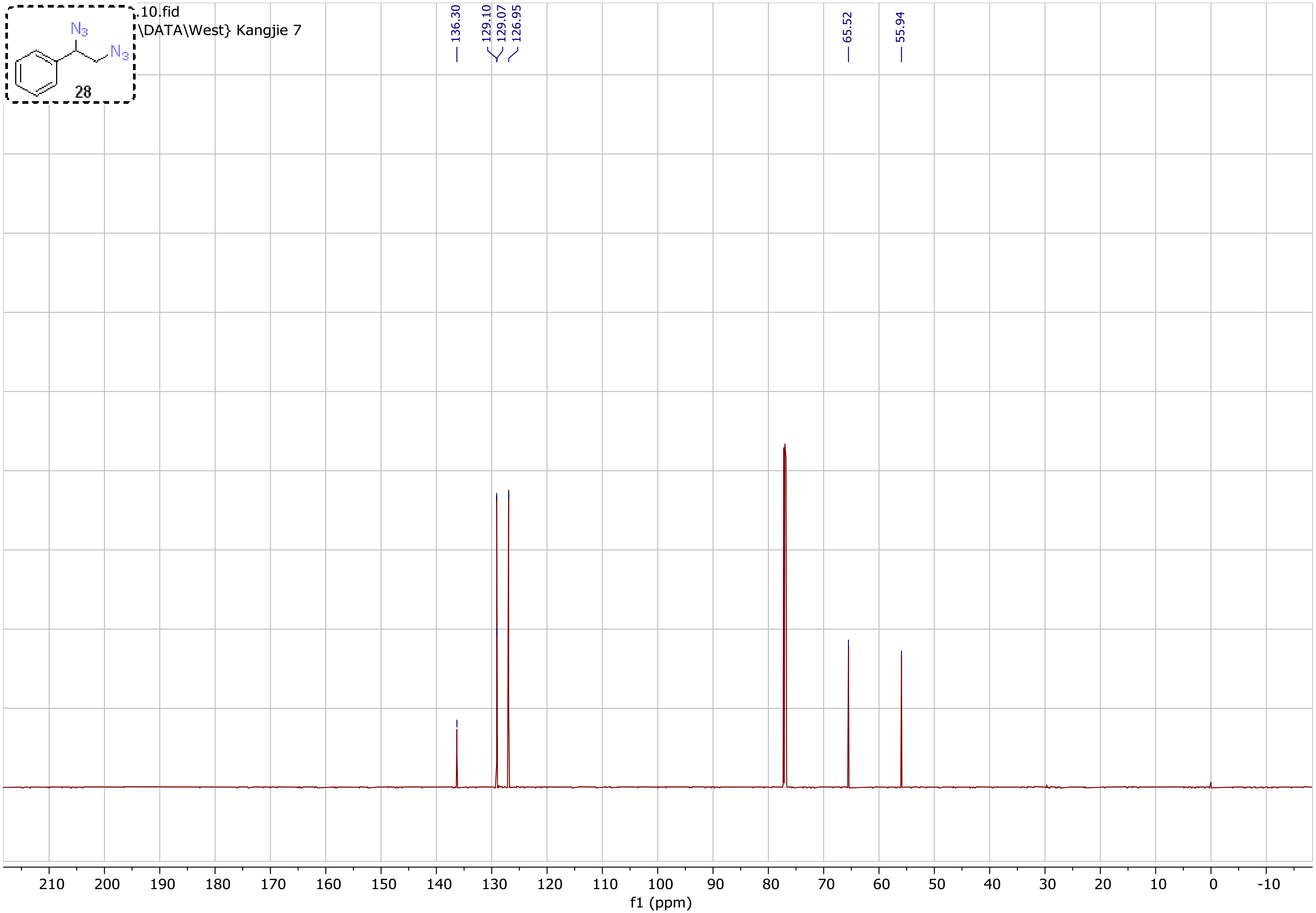

.10.fid
\DATA\West} Kangjie 7
N3
N3
28
136.30
129.10
129.07
126.95
65.52
55.94
210
200
190
180
170
160
150
140
130
120
110
100
90
80
70
60
50
40
30
20
10
0
-10
f1 (ppm)

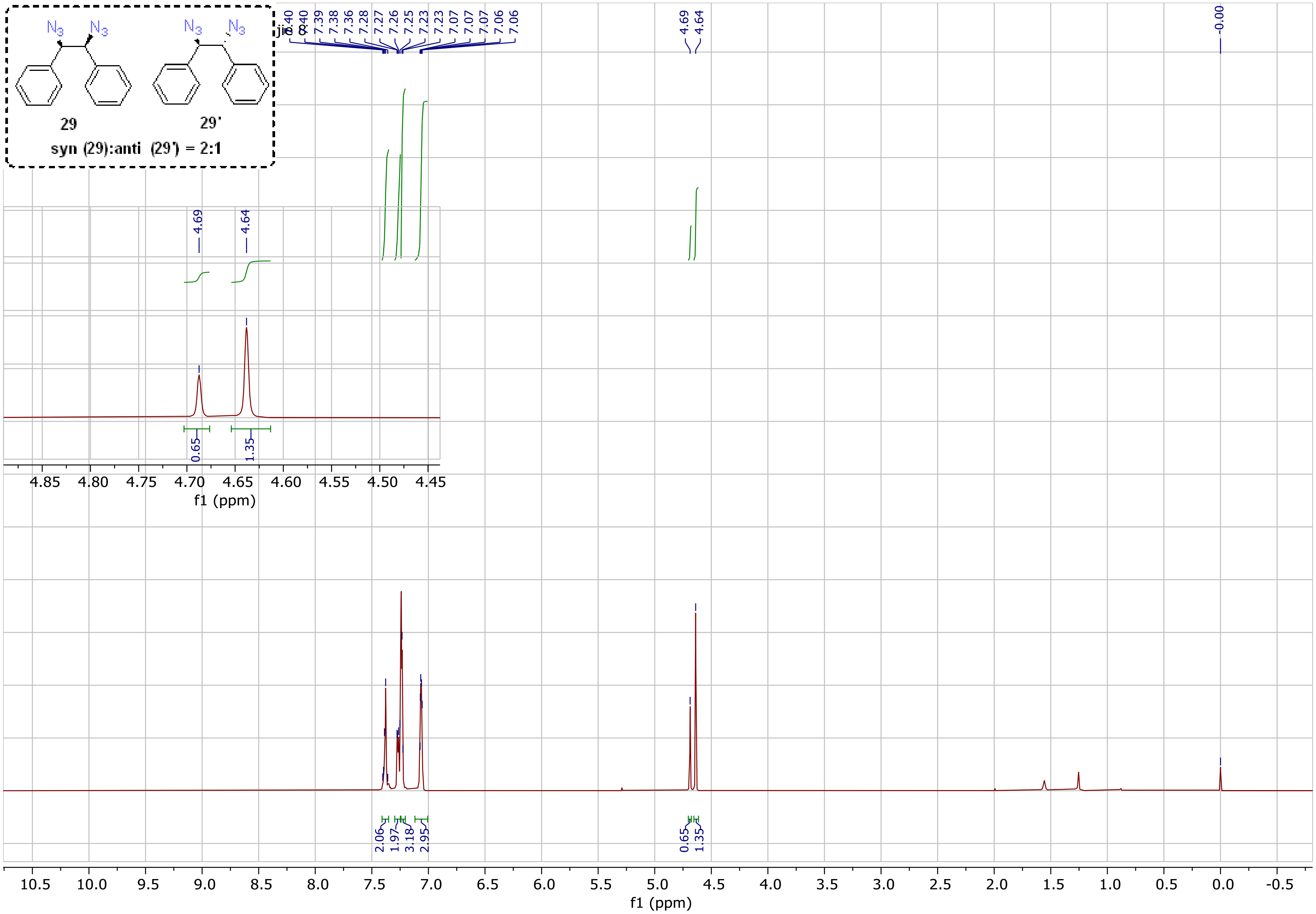

N3 N3
29
29'
syn (29):anti (29') = 2:1
f1 (ppm)

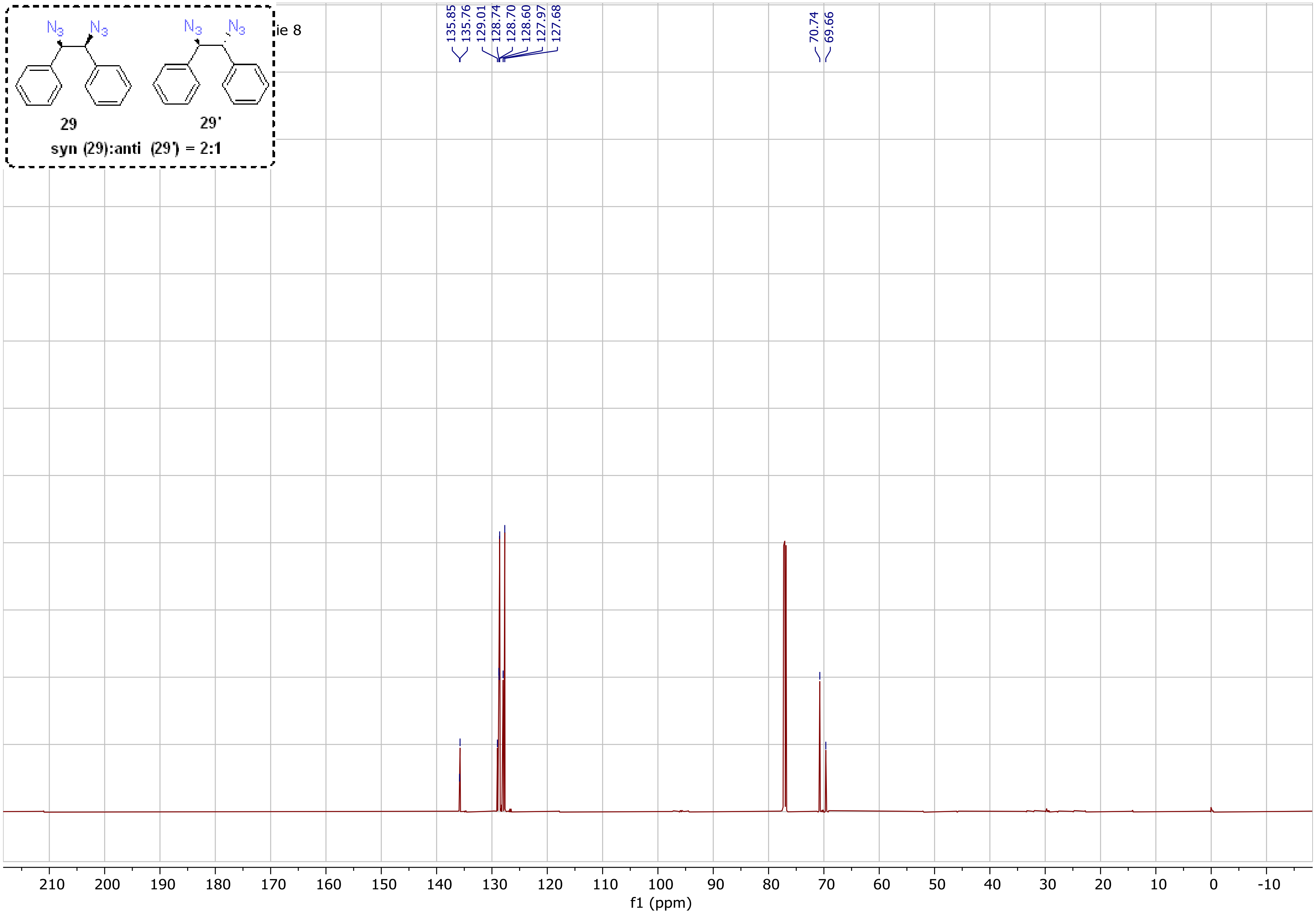
N3
29
29'
syn (29):anti (29') = 2:1
ie 8
135.85
135.76
129.01
128.74
128.70
128.60
127.97
127.68
70.74
69.66
f1 (ppm)

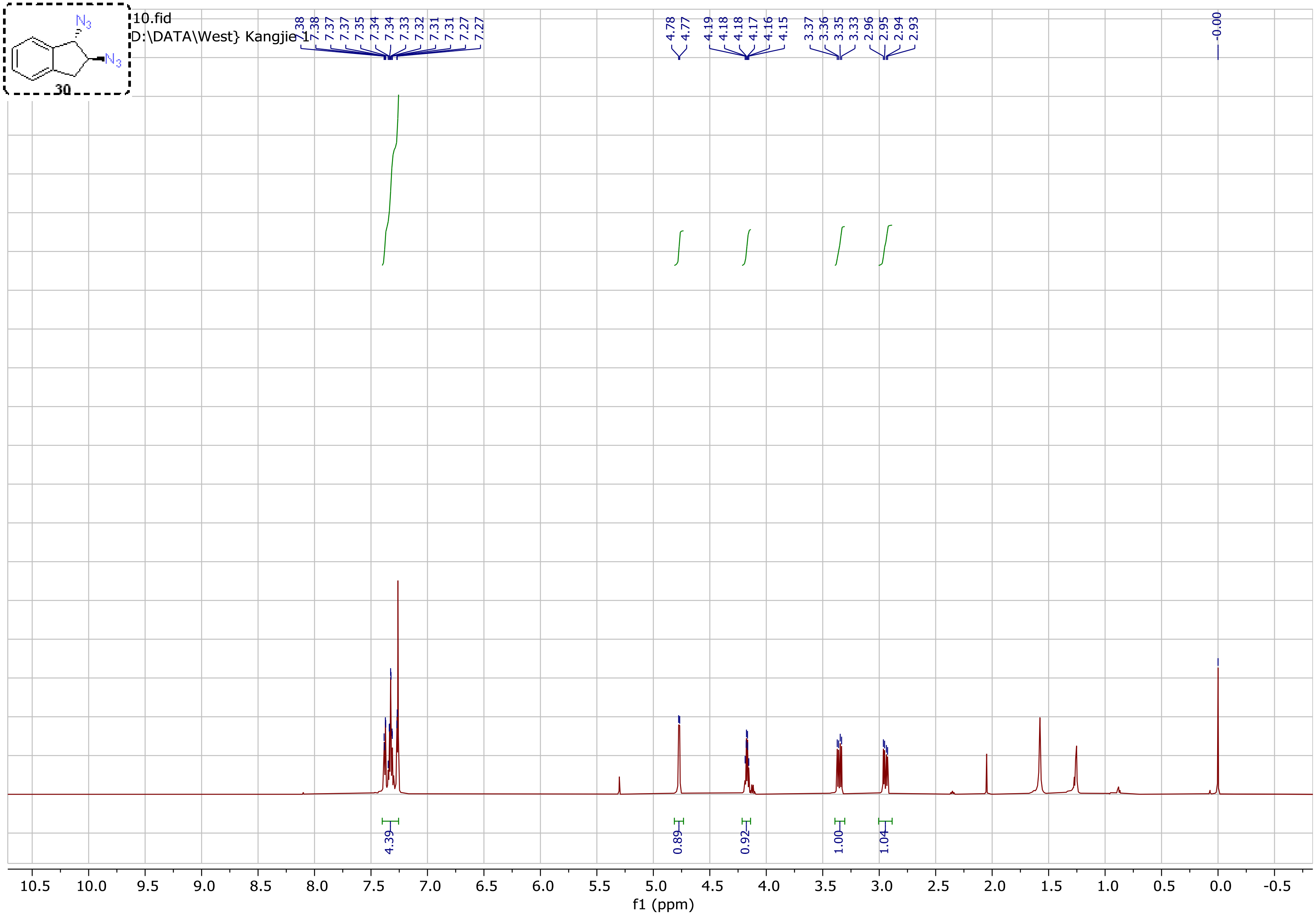

30
10.fid
D:\DATA\West} Kangjie 1
7.38
7.38
7.37
7.37
7.35
7.34
7.34
7.33
7.32
7.31
7.31
7.27
7.27
4.78
4.77
4.19
4.18
4.18
4.17
4.16
4.15
3.37
3.36
3.35
3.33
2.96
2.95
2.94
2.93
-0.00
4.39
0.89
0.92
1.00
1.04
f1 (ppm)

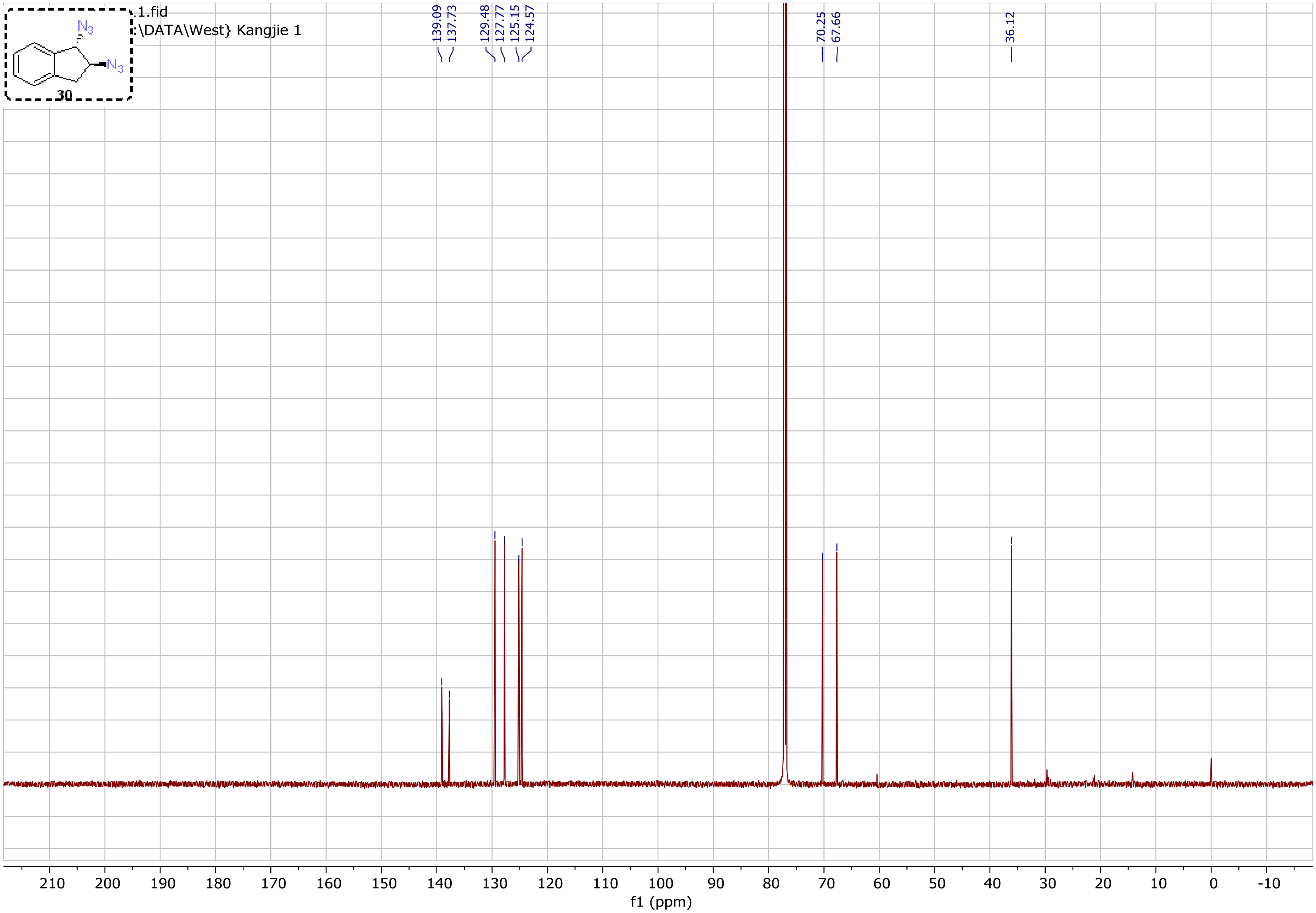
.1.fid
:\DATA\West} Kangjie 1
139.09
137.73
129.48
127.77
125.15
124.57
70.25
67.66
36.12
N3
N3
30
210 200 190 180 170 160 150 140 130 120 110 100 90 80 70 60 50 40 30 20 10 0 -10
f1 (ppm)

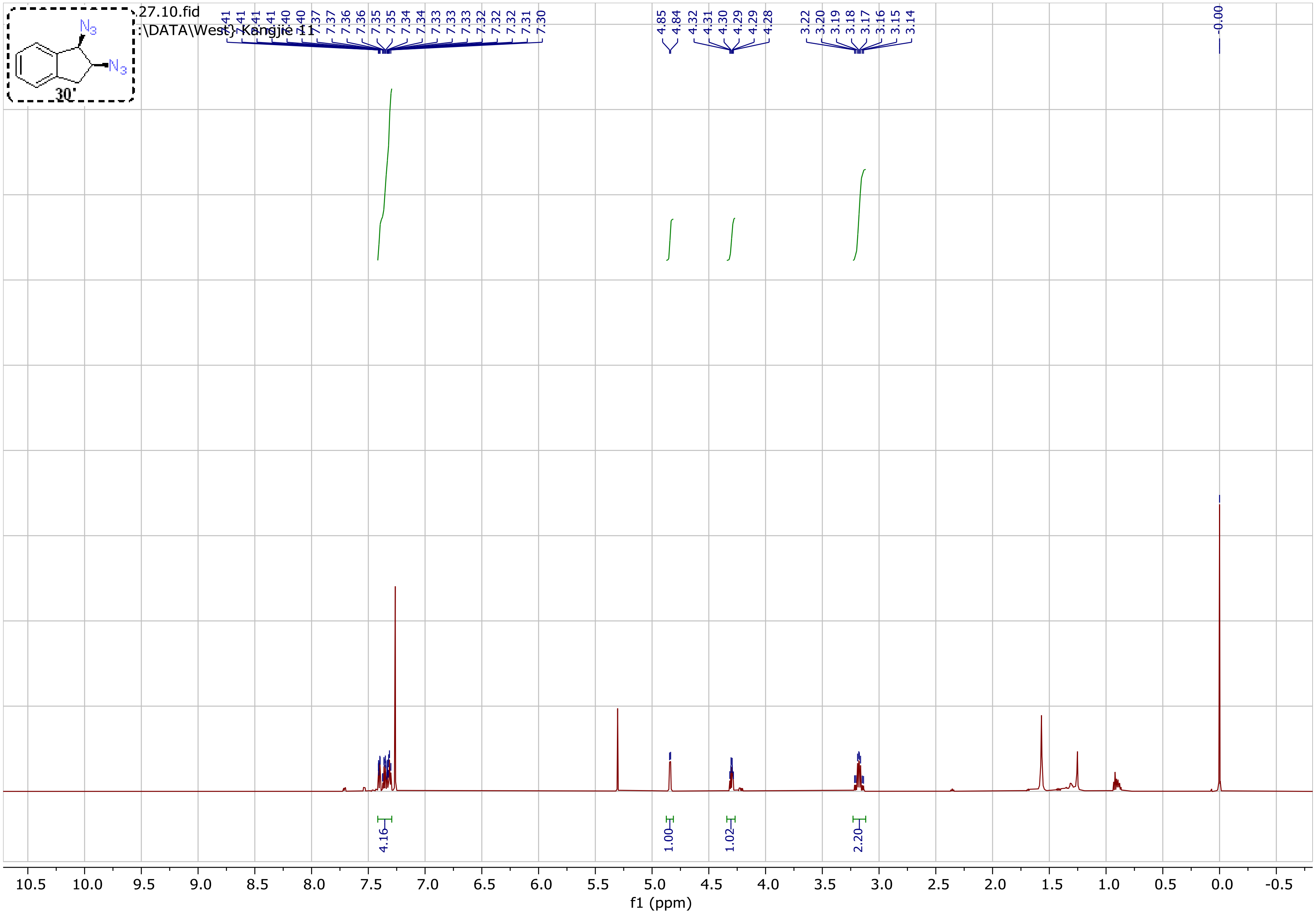
27.10.fid
:\DATA\West} Kangjie 11
N3
N3
30'
7.41 7.41 7.41 7.41 7.40 7.40 7.37 7.37 7.36 7.36 7.35 7.35 7.34 7.34 7.33 7.33 7.33 7.32 7.32 7.32 7.31 7.30
4.85 4.84
4.32 4.31 4.30 4.29 4.29 4.28
3.22 3.20 3.19 3.18 3.17 3.16 3.15 3.14
-0.00
4.16
1.00
1.02
2.20
10.5 10.0 9.5 9.0 8.5 8.0 7.5 7.0 6.5 6.0 5.5 5.0 4.5 4.0 3.5 3.0 2.5 2.0 1.5 1.0 0.5 0.0 -0.5
f1 (ppm)

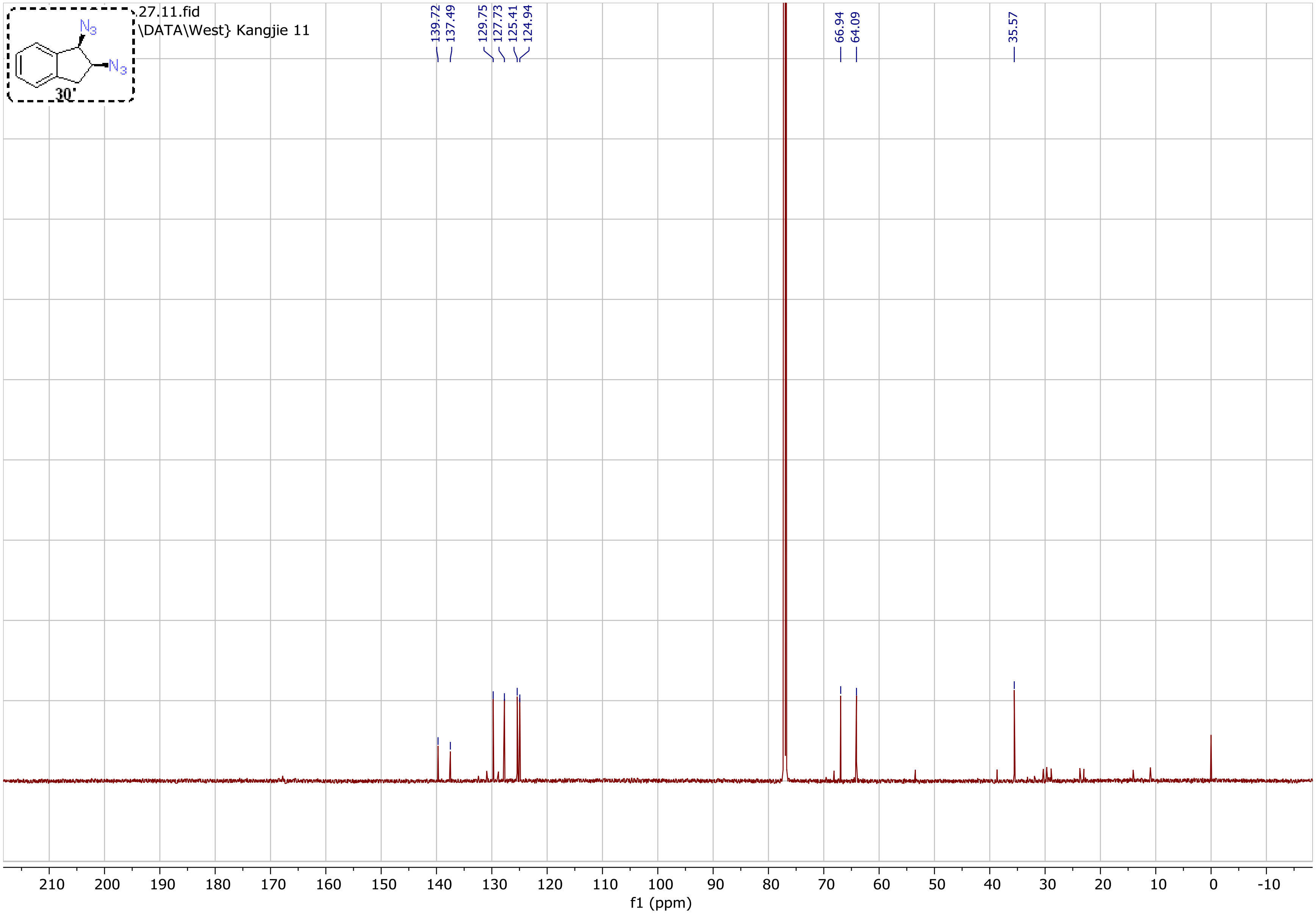
27.11.fid
\DATA\West} Kangjie 11
N3
N3
30'
139.72
137.49
129.75
127.73
125.41
124.94
66.94
64.09
35.57
210 200 190 180 170 160 150 140 130 120 110 100 90 80 70 60 50 40 30 20 10 0 -10
f1 (ppm)

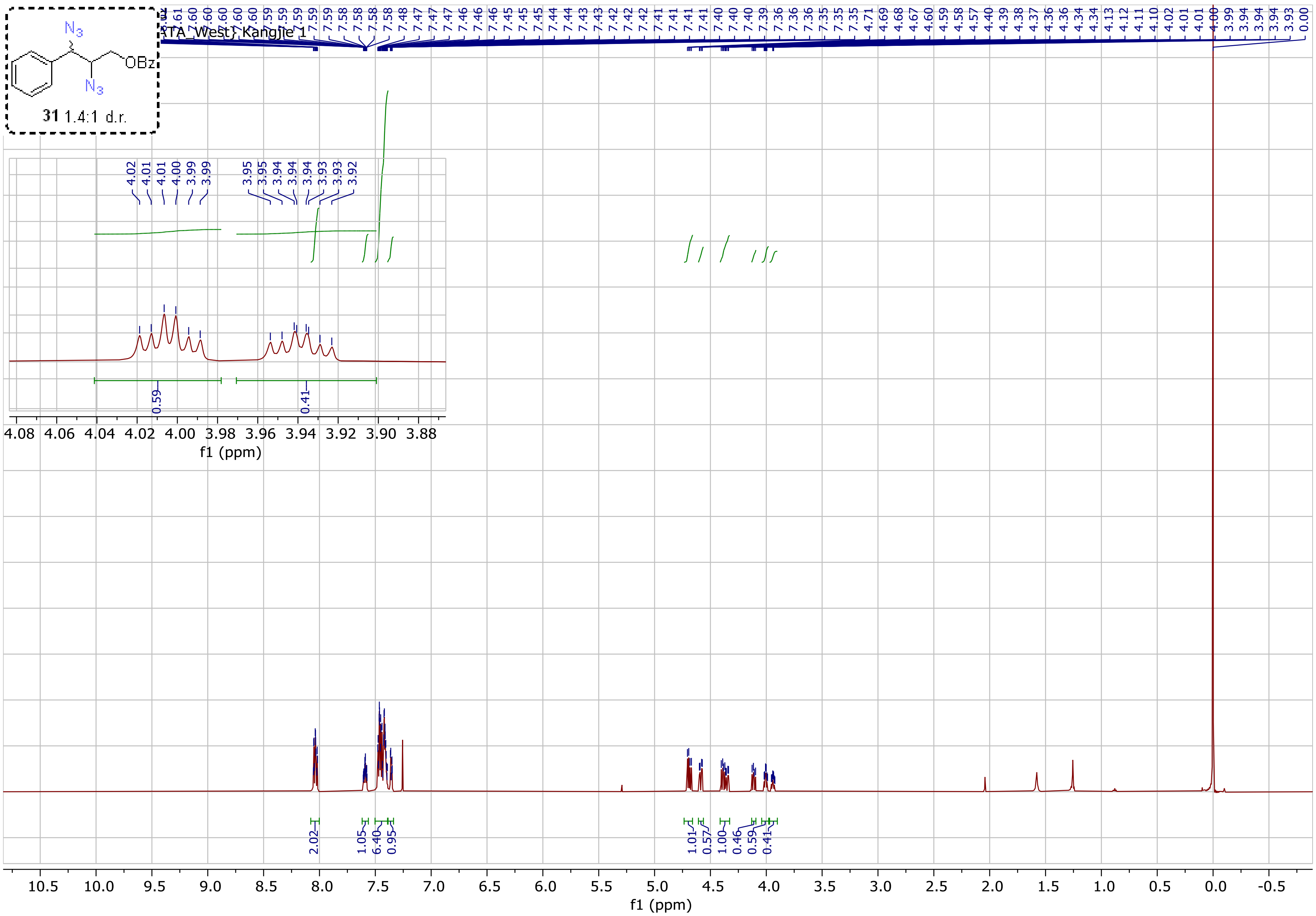

N3
N3
OBz
31 1.4:1 d.r.
f1 (ppm)

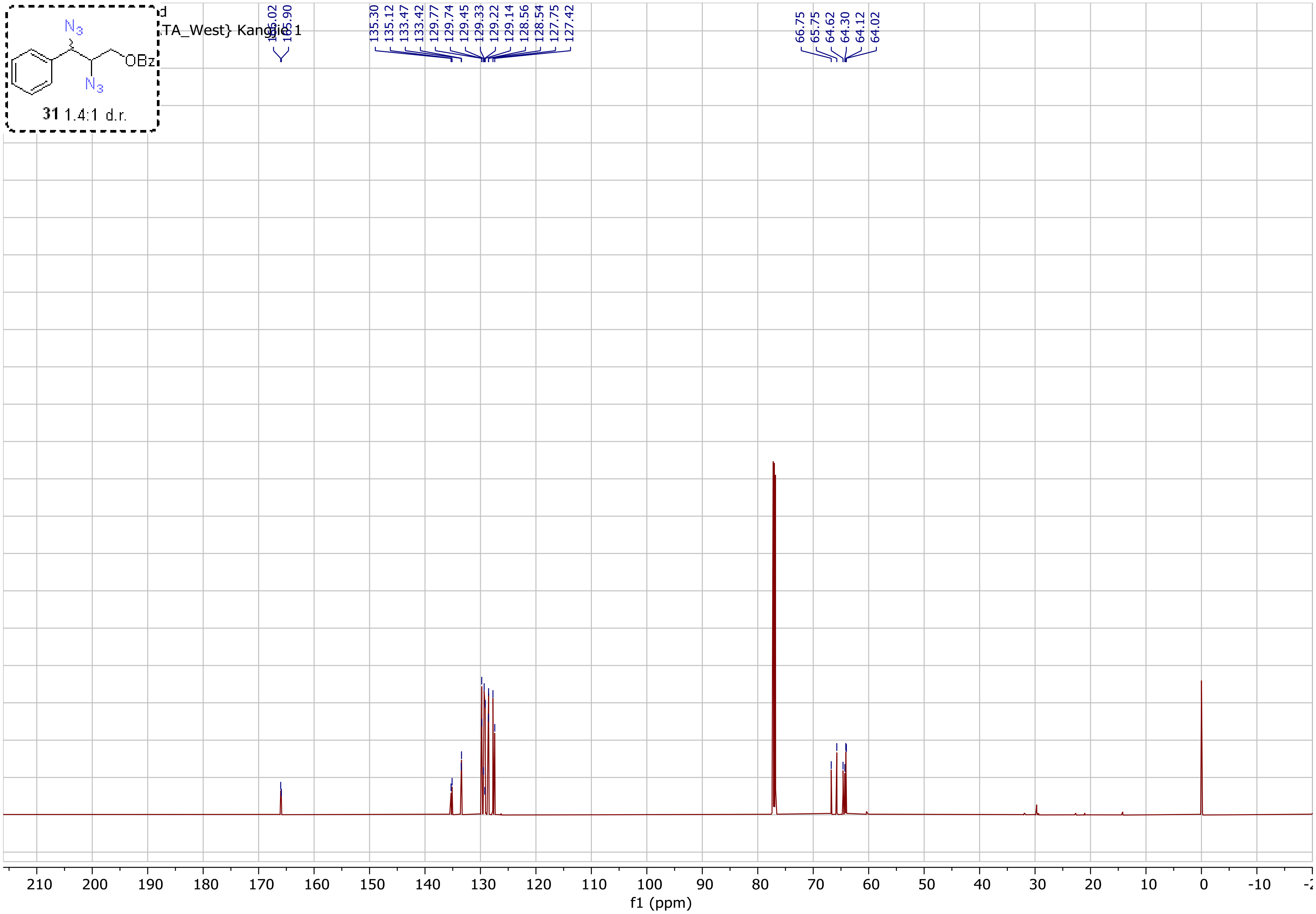
N3
N3
OBz
31 1.4:1 d.r.
165.90
135.30
135.12
133.47
133.42
129.77
129.74
129.45
129.33
129.22
129.14
128.56
128.54
127.75
127.42
66.75
65.75
64.62
64.30
64.12
64.02
f1 (ppm)

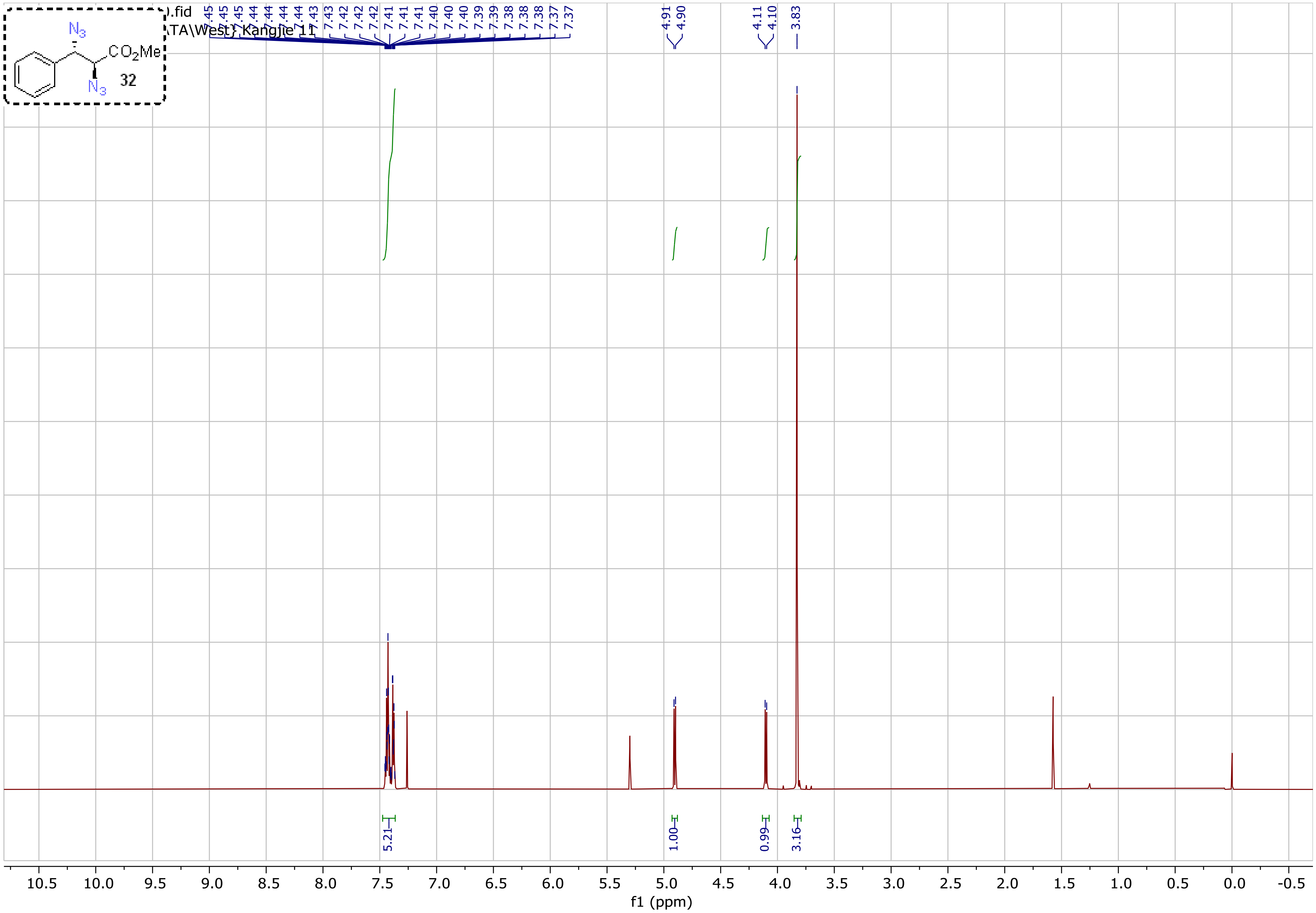

).fid
\TA\West} Kangjie 11
N3
CO2Me
N3
32
7.45 7.45 7.45 7.44 7.44 7.44 7.44 7.43 7.43 7.42 7.42 7.42 7.41 7.41 7.41 7.40 7.40 7.40 7.39 7.39 7.38 7.38 7.38 7.37 7.37
4.91 4.90
4.11 4.10
3.83
5.21
1.00
0.99
3.16
10.5 10.0 9.5 9.0 8.5 8.0 7.5 7.0 6.5 6.0 5.5 5.0 4.5 4.0 3.5 3.0 2.5 2.0 1.5 1.0 0.5 0.0 -0.5
f1 (ppm)

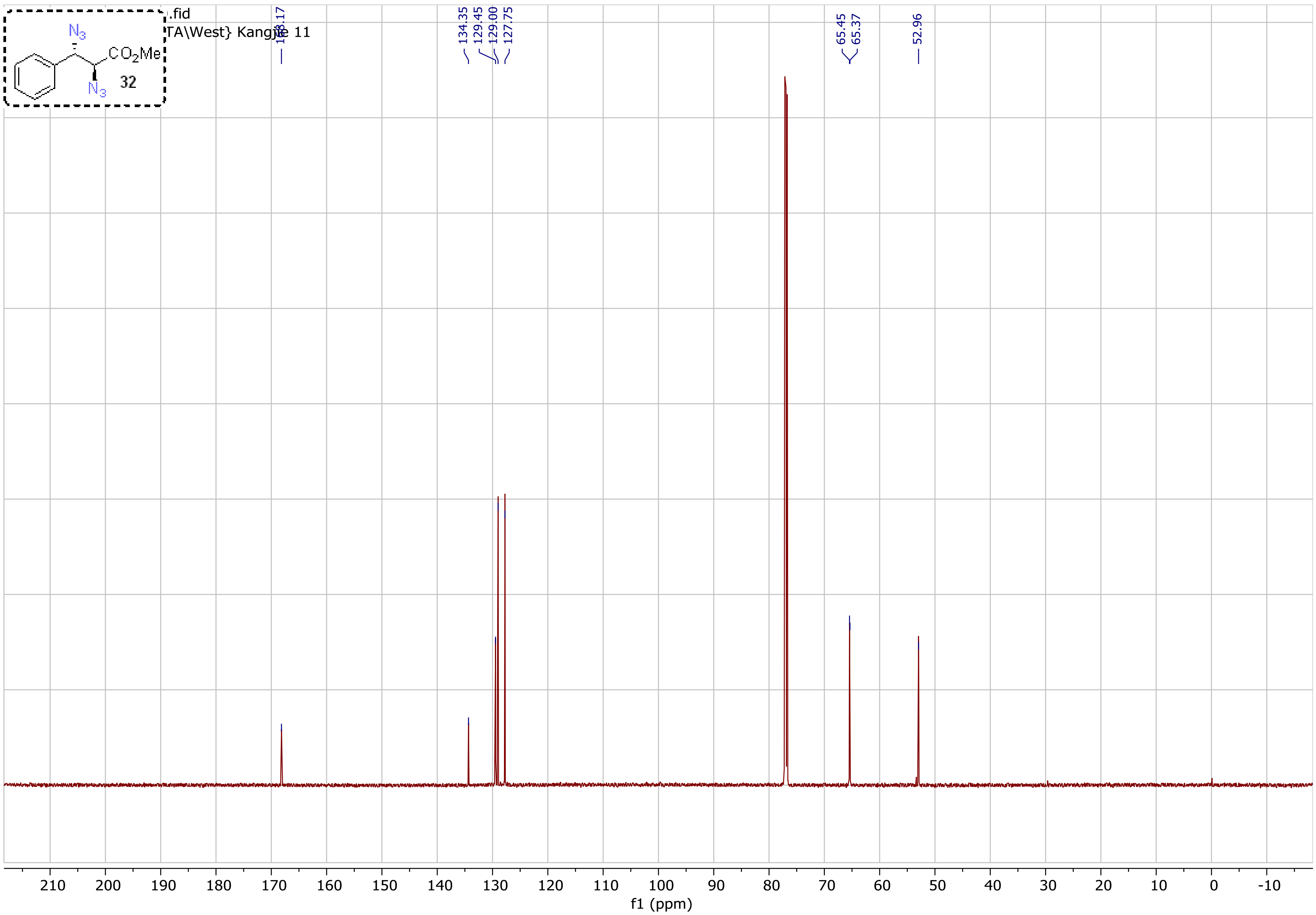
.fid
TA\West} Kangjie 11
N_3
CO_2Me
32
168.17
134.35
129.45
129.00
127.75
65.45
65.37
52.96
f1 (ppm)

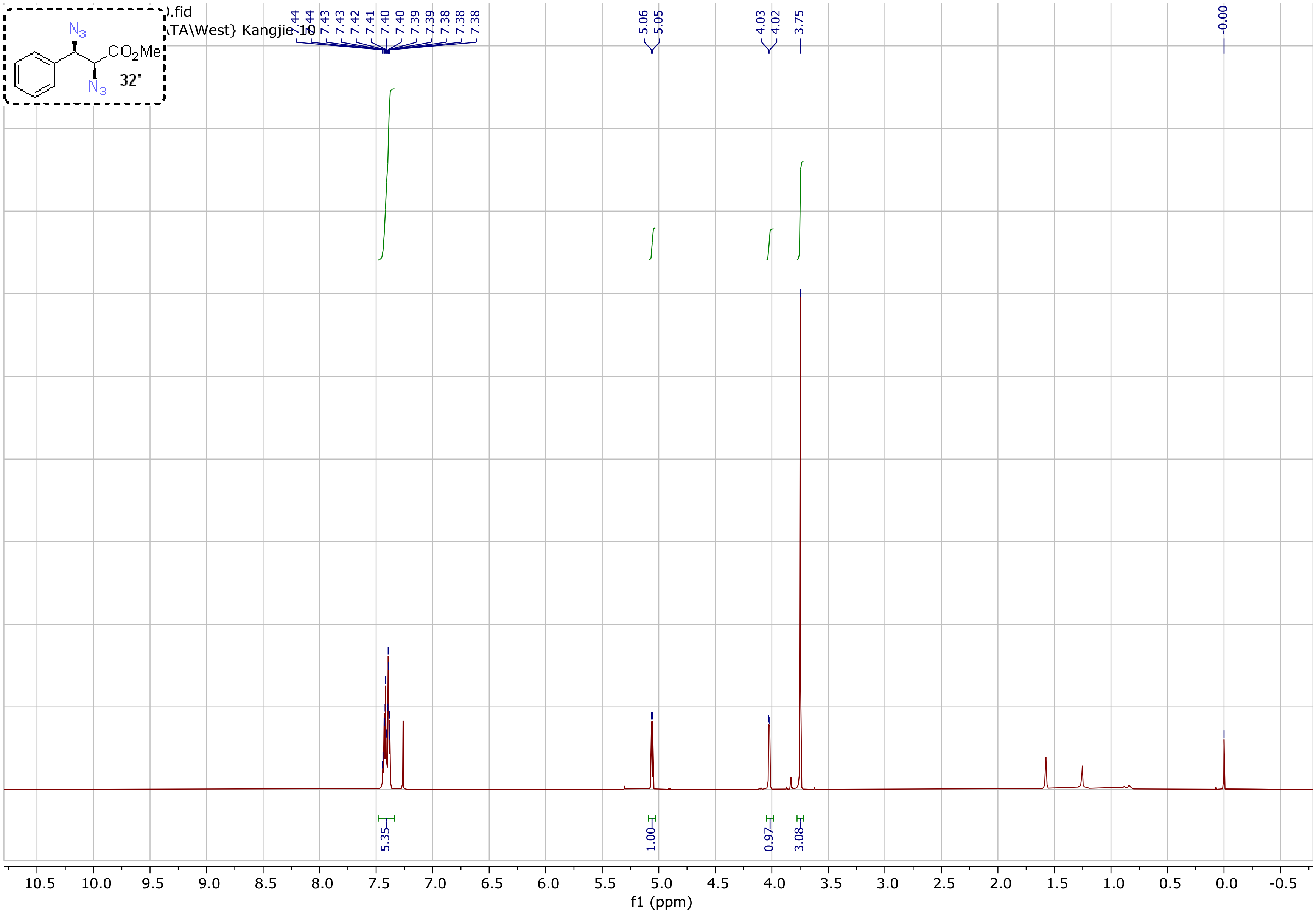
).fid
\TA\West} Kangjie 10
N3
CO2Me
N3
32'
7.44
7.44
7.43
7.43
7.42
7.41
7.40
7.40
7.39
7.39
7.38
7.38
7.38
5.06
5.05
4.03
4.02
3.75
-0.00
5.35
1.00
0.97
3.08
10.5
10.0
9.5
9.0
8.5
8.0
7.5
7.0
6.5
6.0
5.5
5.0
4.5
4.0
3.5
3.0
2.5
2.0
1.5
1.0
0.5
0.0
-0.5
f1 (ppm)

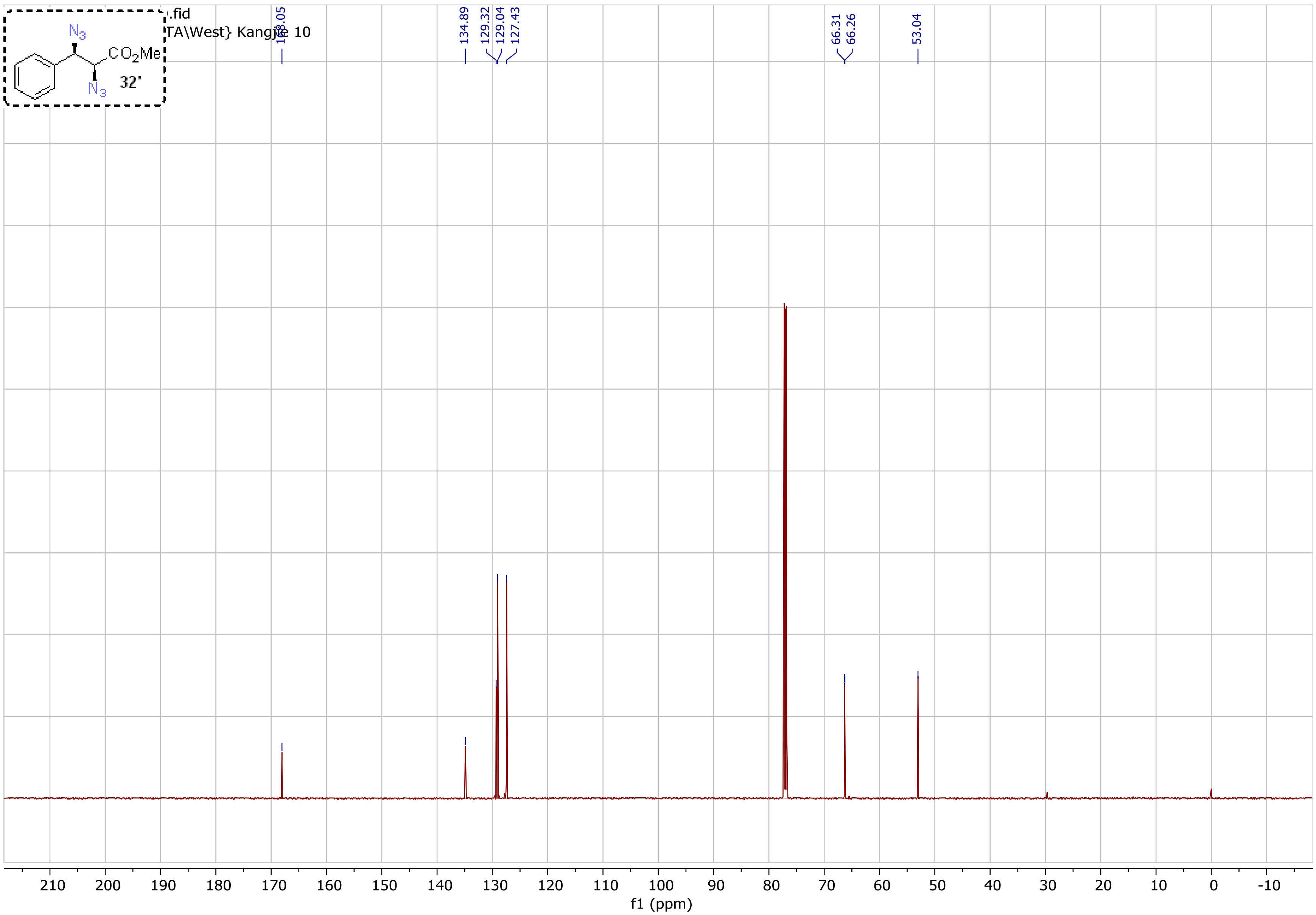
.fid
TA\West} Kangjie 10
N3
CO2Me
N3
32'
168.05
134.89
129.32
129.04
127.43
66.31
66.26
53.04
210 200 190 180 170 160 150 140 130 120 110 100 90 80 70 60 50 40 30 20 10 0 -10
f1 (ppm)

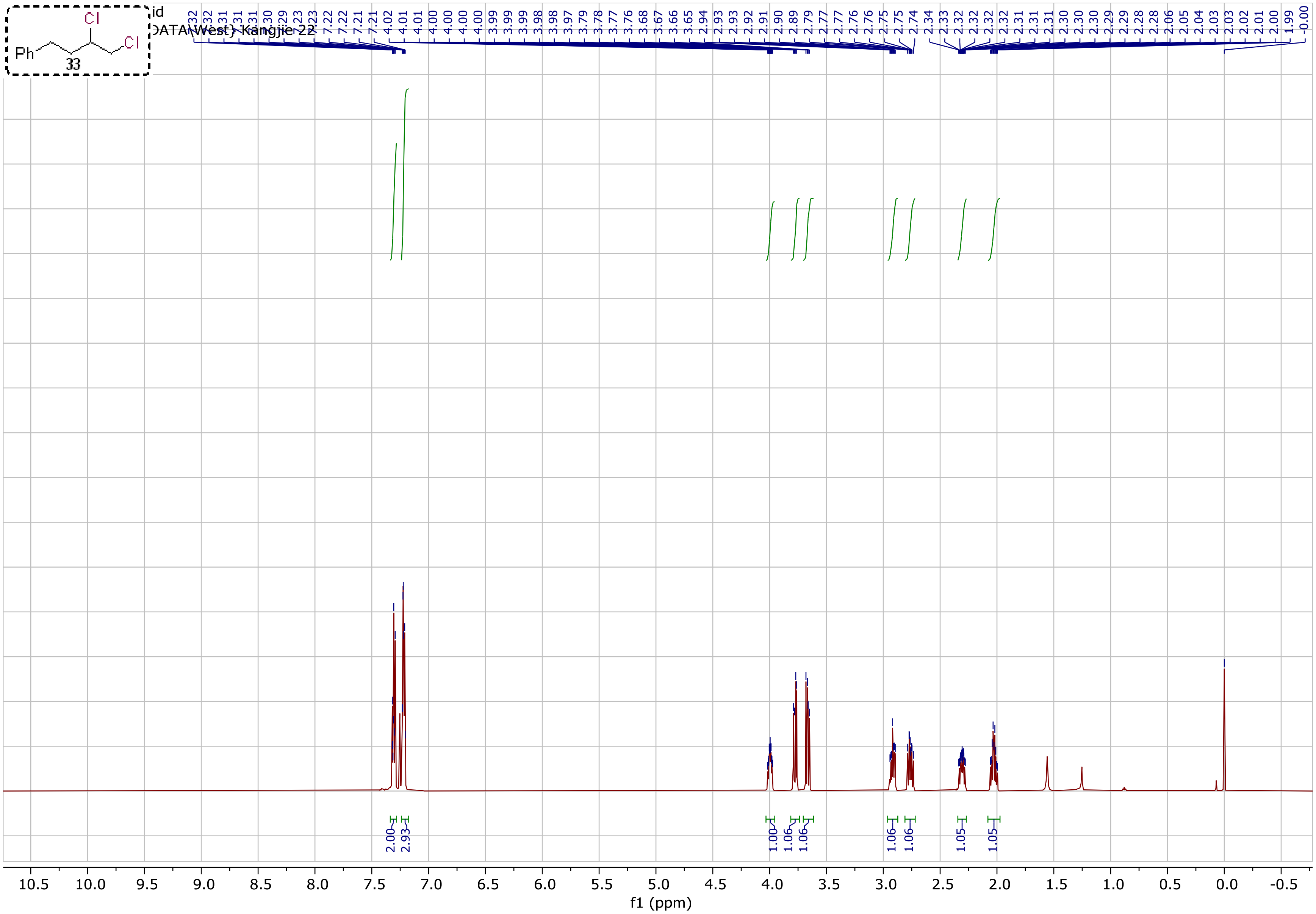
Cl
Ph
Cl
33
f1 (ppm)

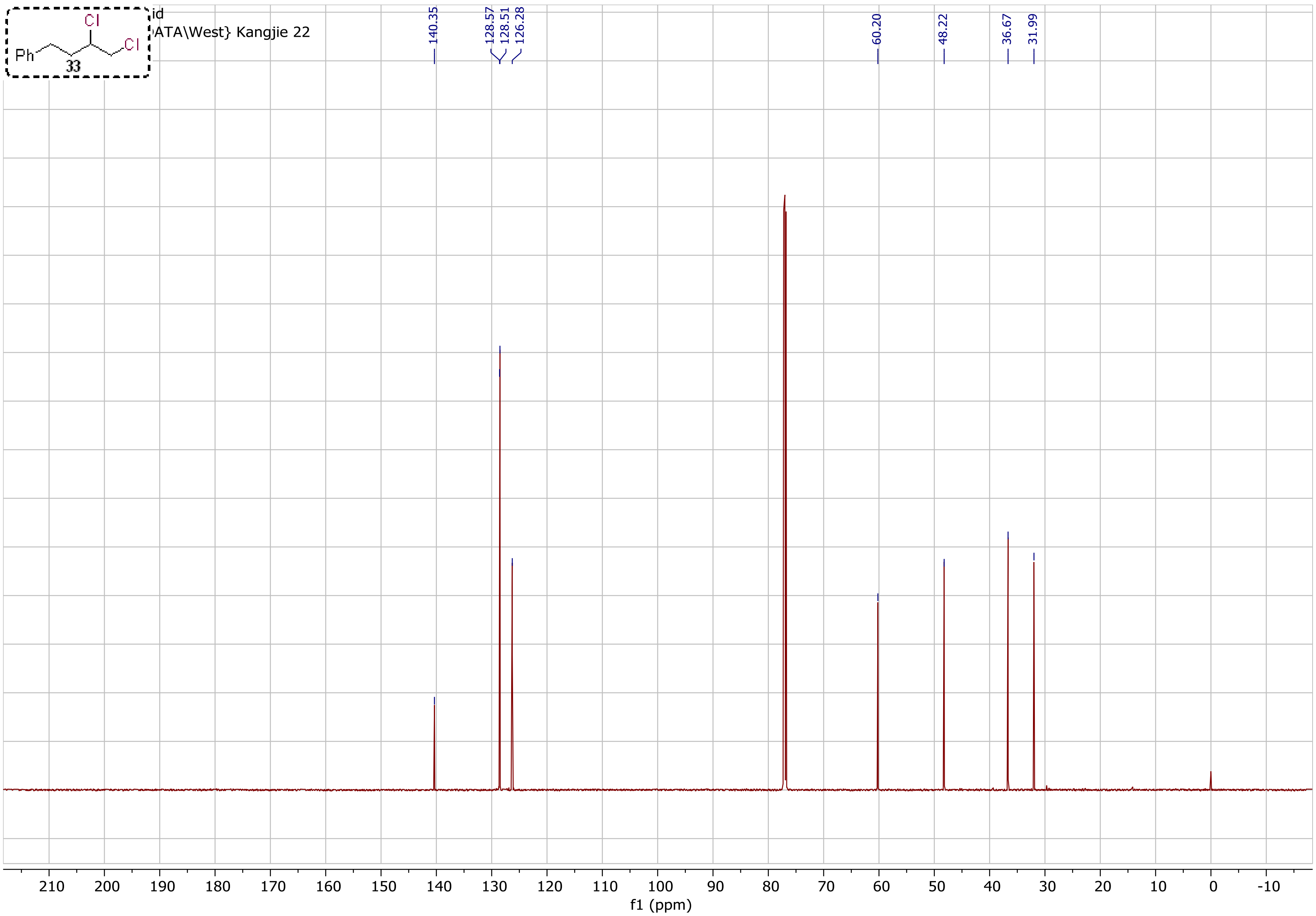

Cl
Ph
Cl
33
id
\ATA\West} Kangjie 22
140.35
128.57
128.51
126.28
60.20
48.22
36.67
31.99
210
200
190
180
170
160
150
140
130
120
110
100
90
80
70
60
50
40
30
20
10
0
-10
f1 (ppm)

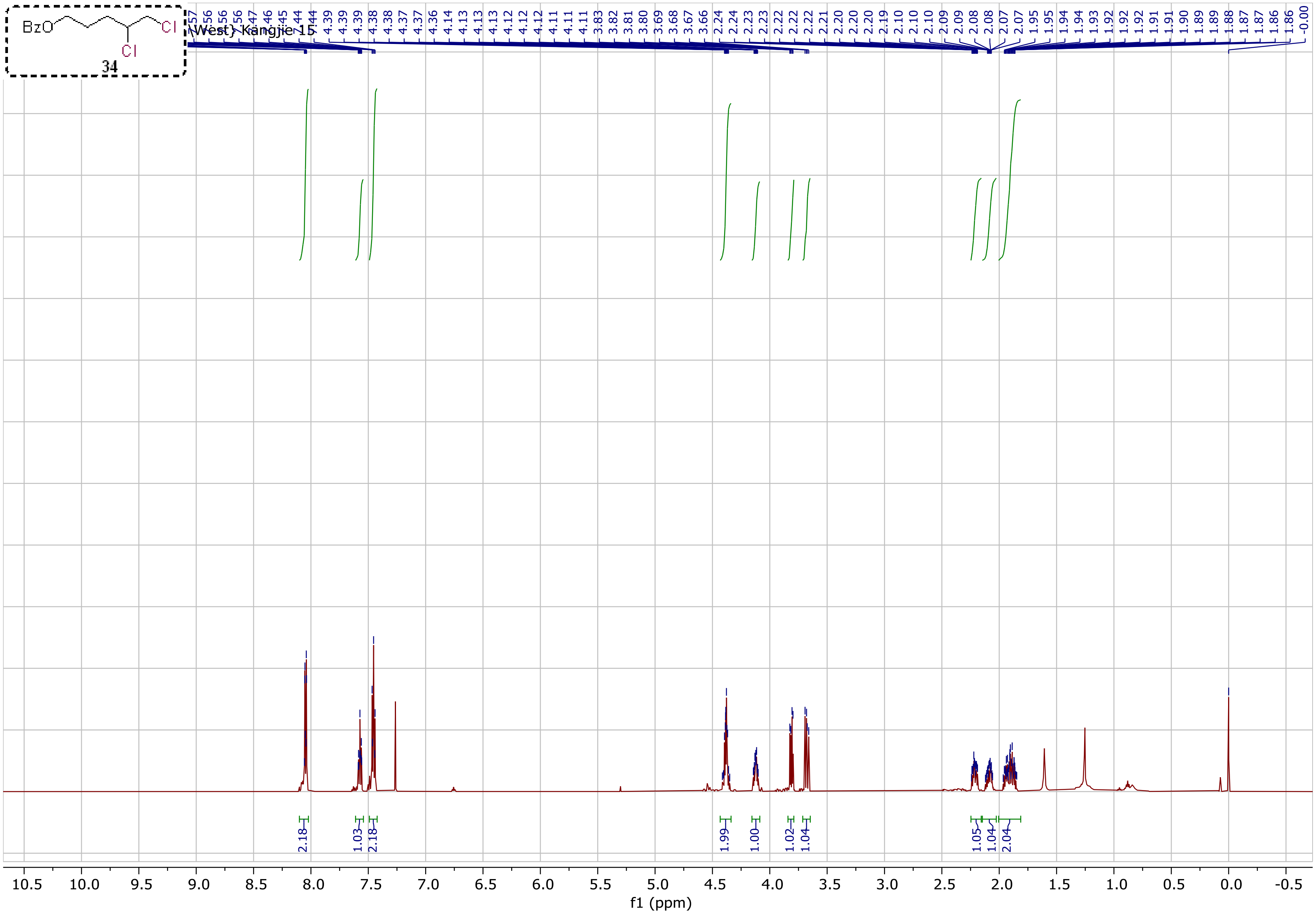
BzO
Cl
Cl
34
(West) Kangjie 15
f1 (ppm)

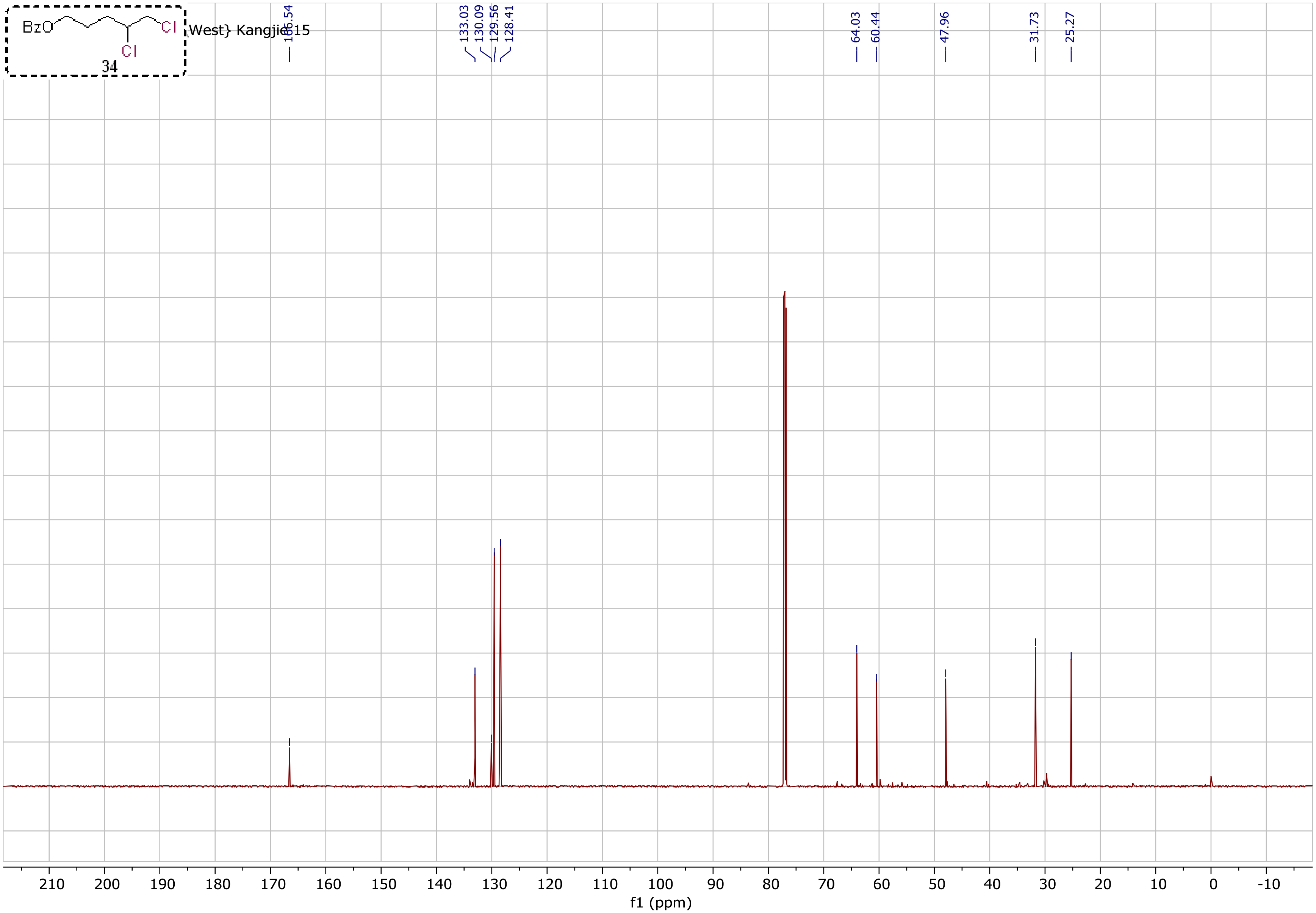

BzO
Cl
Cl
34
West} Kangjie15
166.54
133.03
130.09
129.56
128.41
64.03
60.44
47.96
31.73
25.27
210
200
190
180
170
160
150
140
130
120
110
100
90
80
70
60
50
40
30
20
10
0
-10
f1 (ppm)

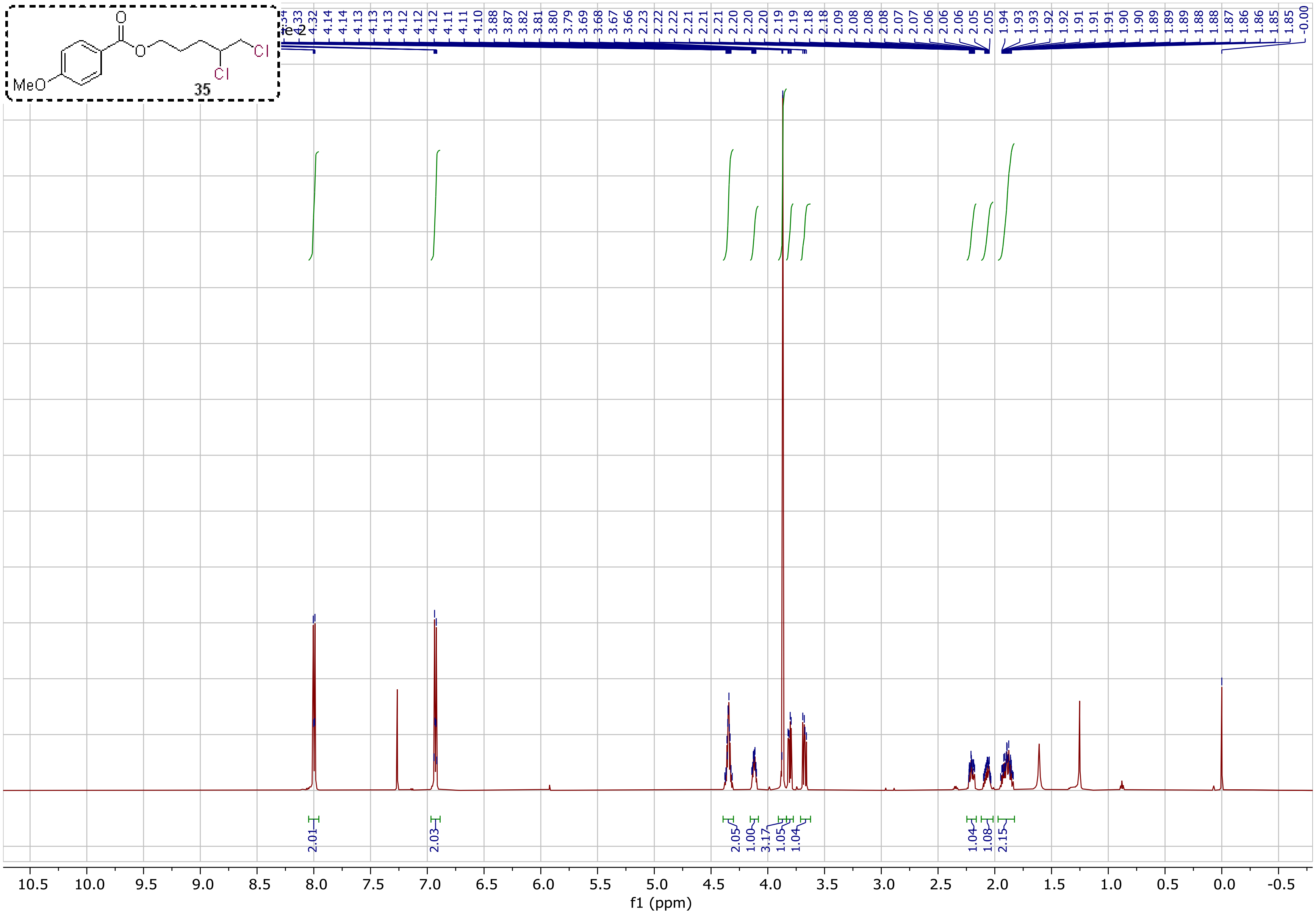

O
O
Cl
Cl
MeO
35
2.01
2.03
2.05
1.00
3.17
1.05
1.04
1.04
1.08
2.15
10.5 10.0 9.5 9.0 8.5 8.0 7.5 7.0 6.5 6.0 5.5 5.0 4.5 4.0 3.5 3.0 2.5 2.0 1.5 1.0 0.5 0.0 -0.5
f1 (ppm)

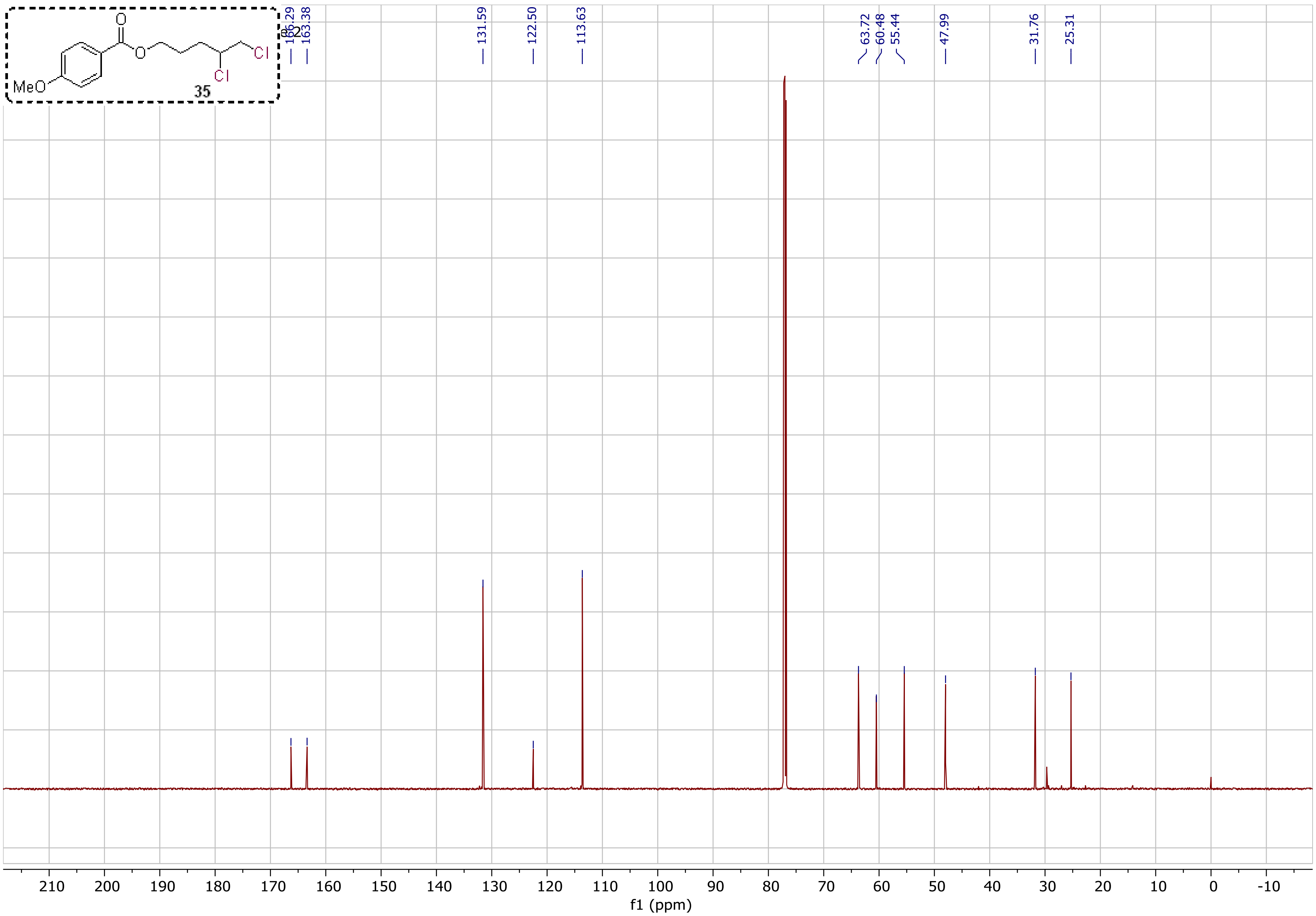
MeO
O
O
Cl
Cl
35
166.29
163.38
131.59
122.50
113.63
63.72
60.48
55.44
47.99
31.76
25.31
210
200
190
180
170
160
150
140
130
120
110
100
90
80
70
60
50
40
30
20
10
0
-10
f1 (ppm)

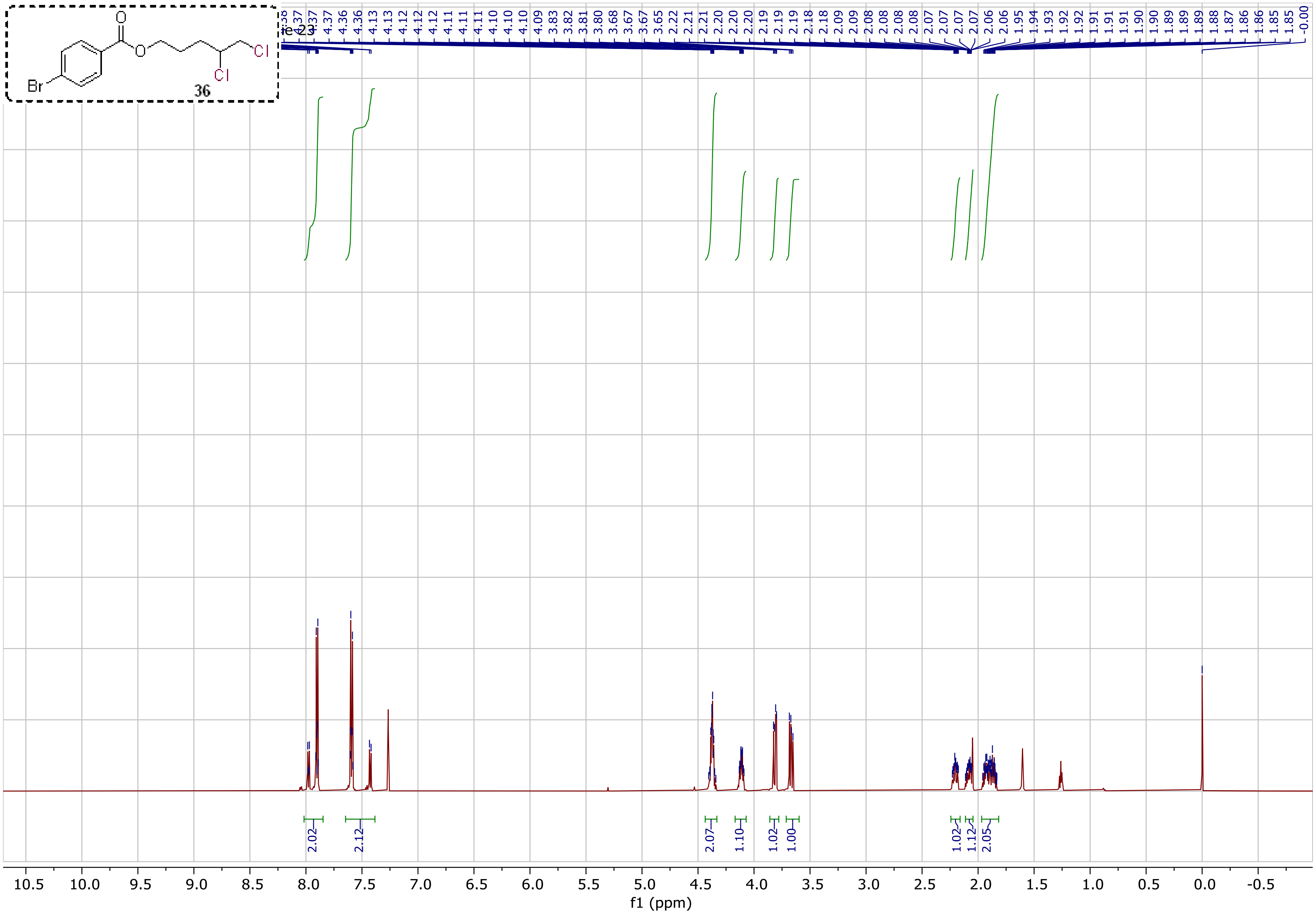
36
2.02
2.12
2.07
1.10
1.02
1.00
1.02
1.12
2.05
10.5 10.0 9.5 9.0 8.5 8.0 7.5 7.0 6.5 6.0 5.5 5.0 4.5 4.0 3.5 3.0 2.5 2.0 1.5 1.0 0.5 0.0 -0.5
f1 (ppm)

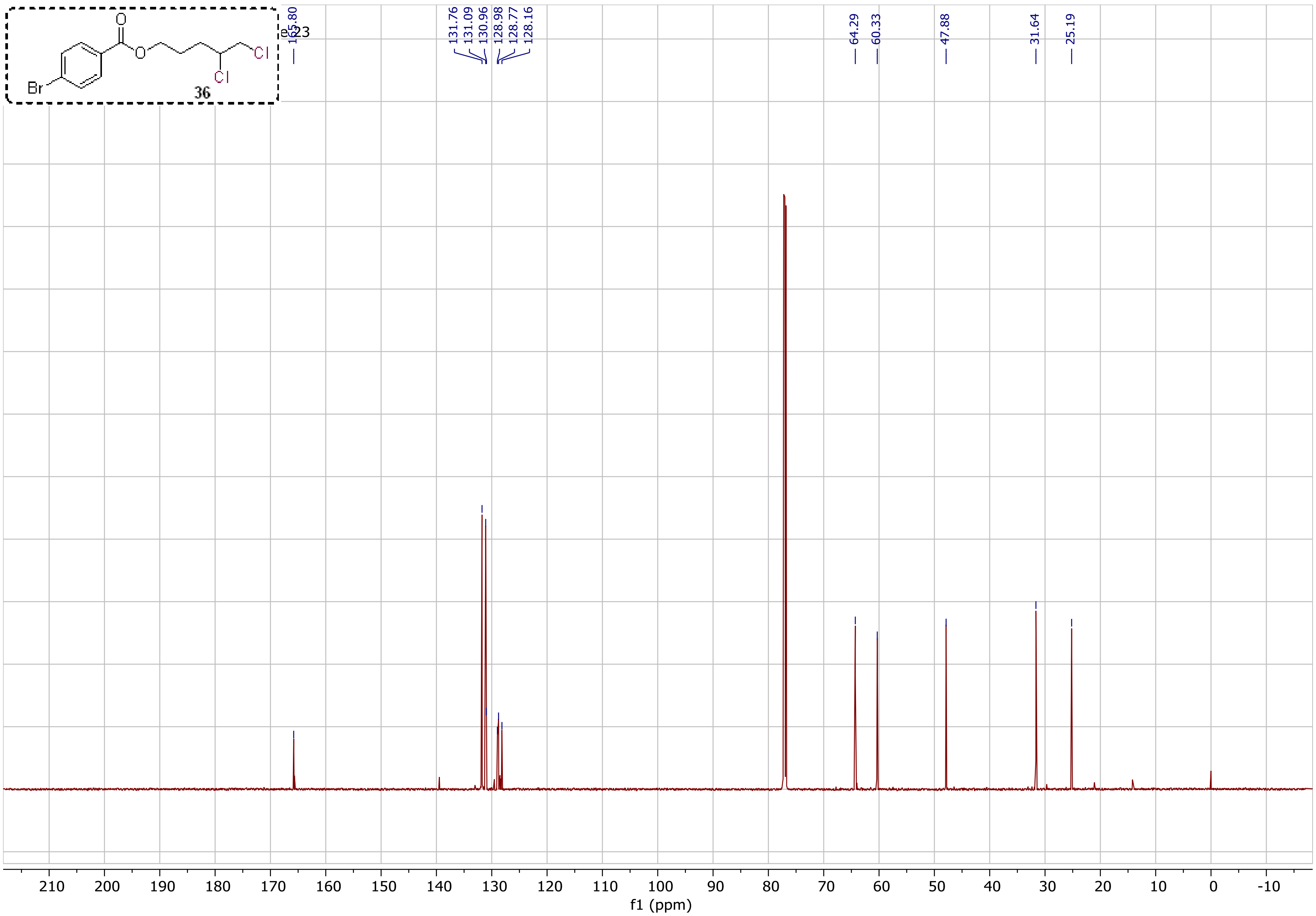
Br
O
O
Cl
Cl
36
e623
165.80
131.76
131.09
130.96
128.98
128.77
128.16
64.29
60.33
47.88
31.64
25.19
210
200
190
180
170
160
150
140
130
120
110
100
90
80
70
60
50
40
30
20
10
0
-10
f1 (ppm)

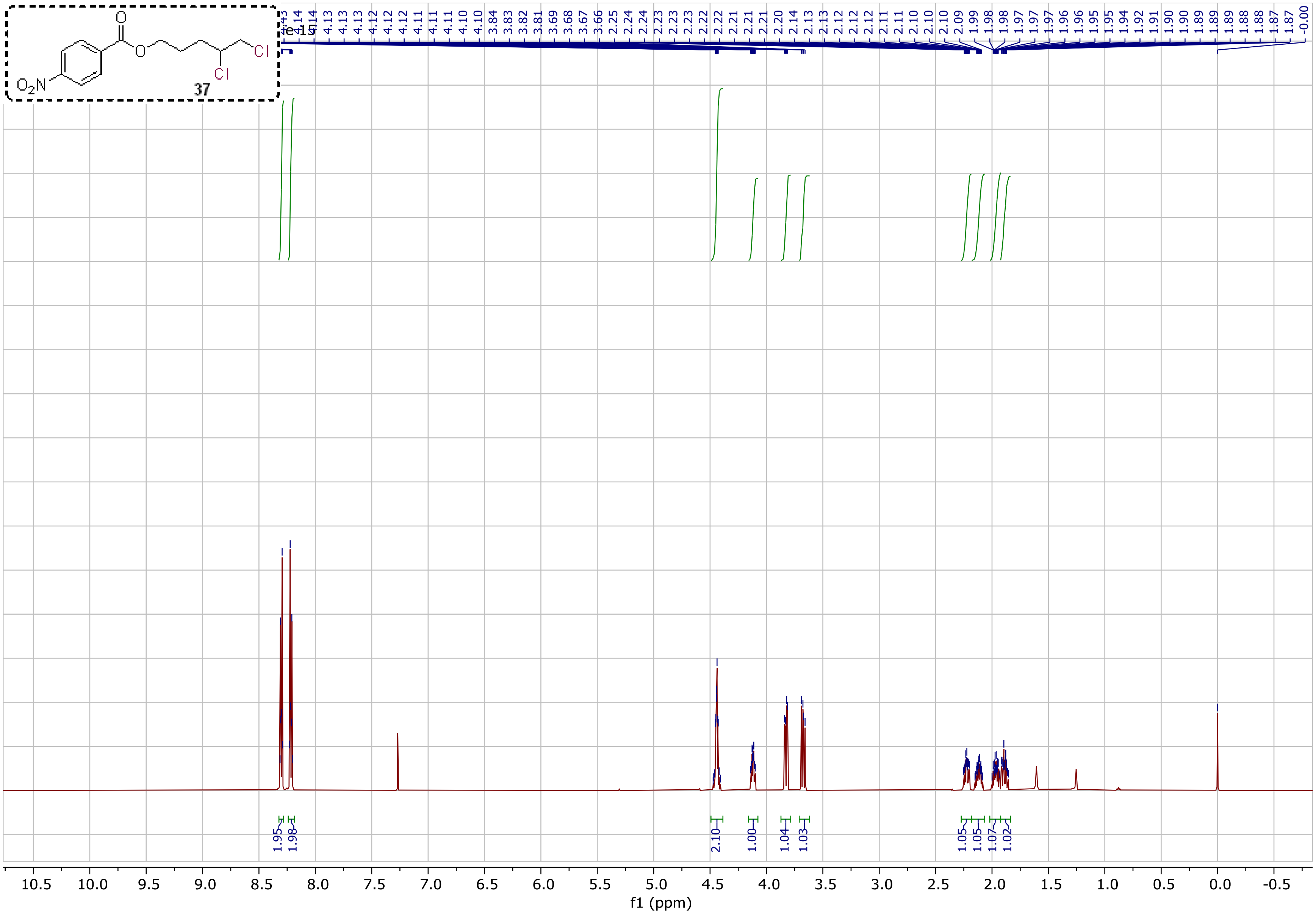
O
O
O_2N
Cl
Cl
37
1.95
1.98
2.10
1.00
1.04
1.03
1.05
1.05
1.07
1.02
10.5 10.0 9.5 9.0 8.5 8.0 7.5 7.0 6.5 6.0 5.5 5.0 4.5 4.0 3.5 3.0 2.5 2.0 1.5 1.0 0.5 0.0 -0.5
f1 (ppm)

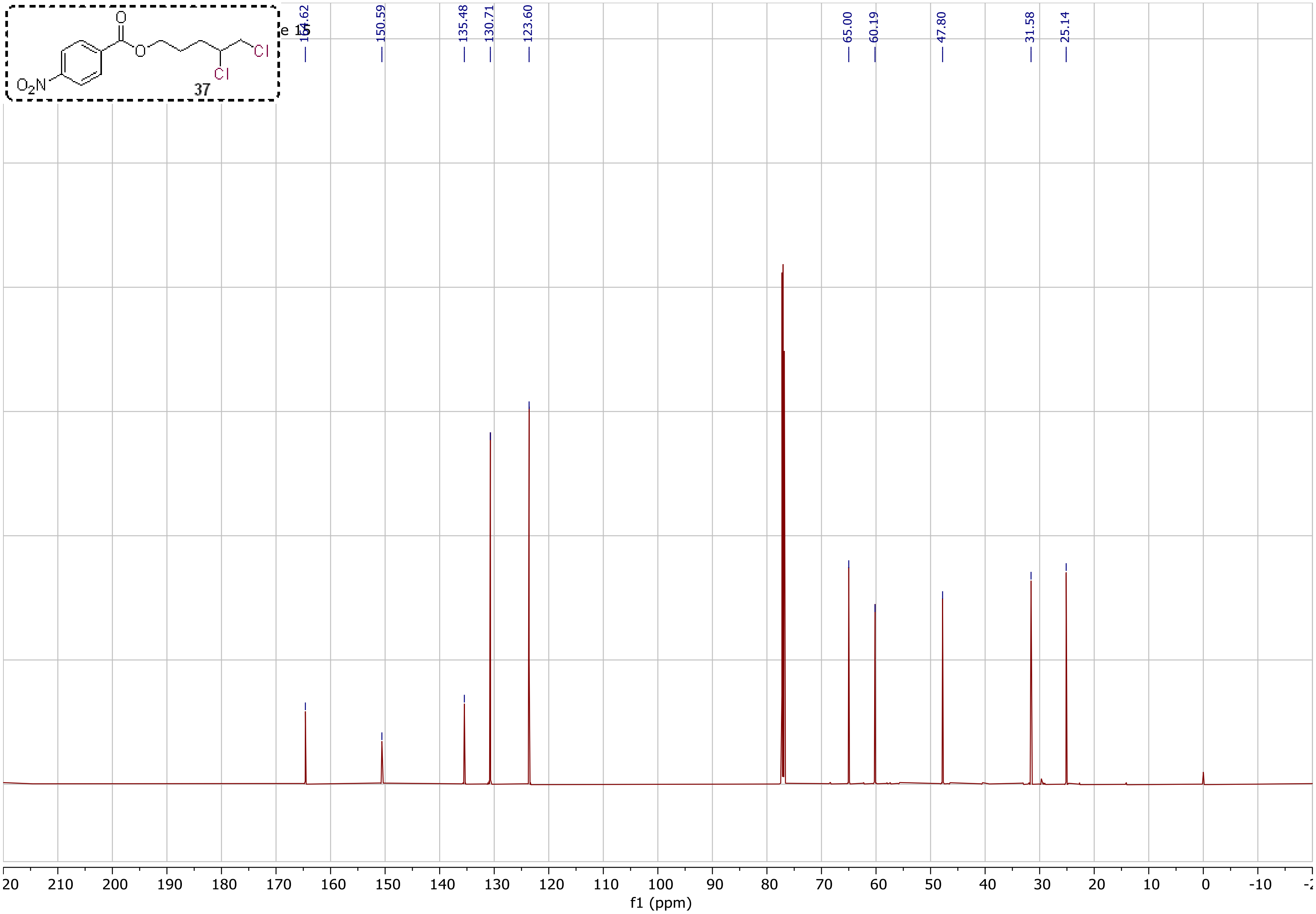
O
O
O2N
Cl
Cl
37
e 17
164.62
150.59
135.48
130.71
123.60
65.00
60.19
47.80
31.58
25.14
220
210
200
190
180
170
160
150
140
130
120
110
100
90
80
70
60
50
40
30
20
10
0
-10
-20
f1 (ppm)

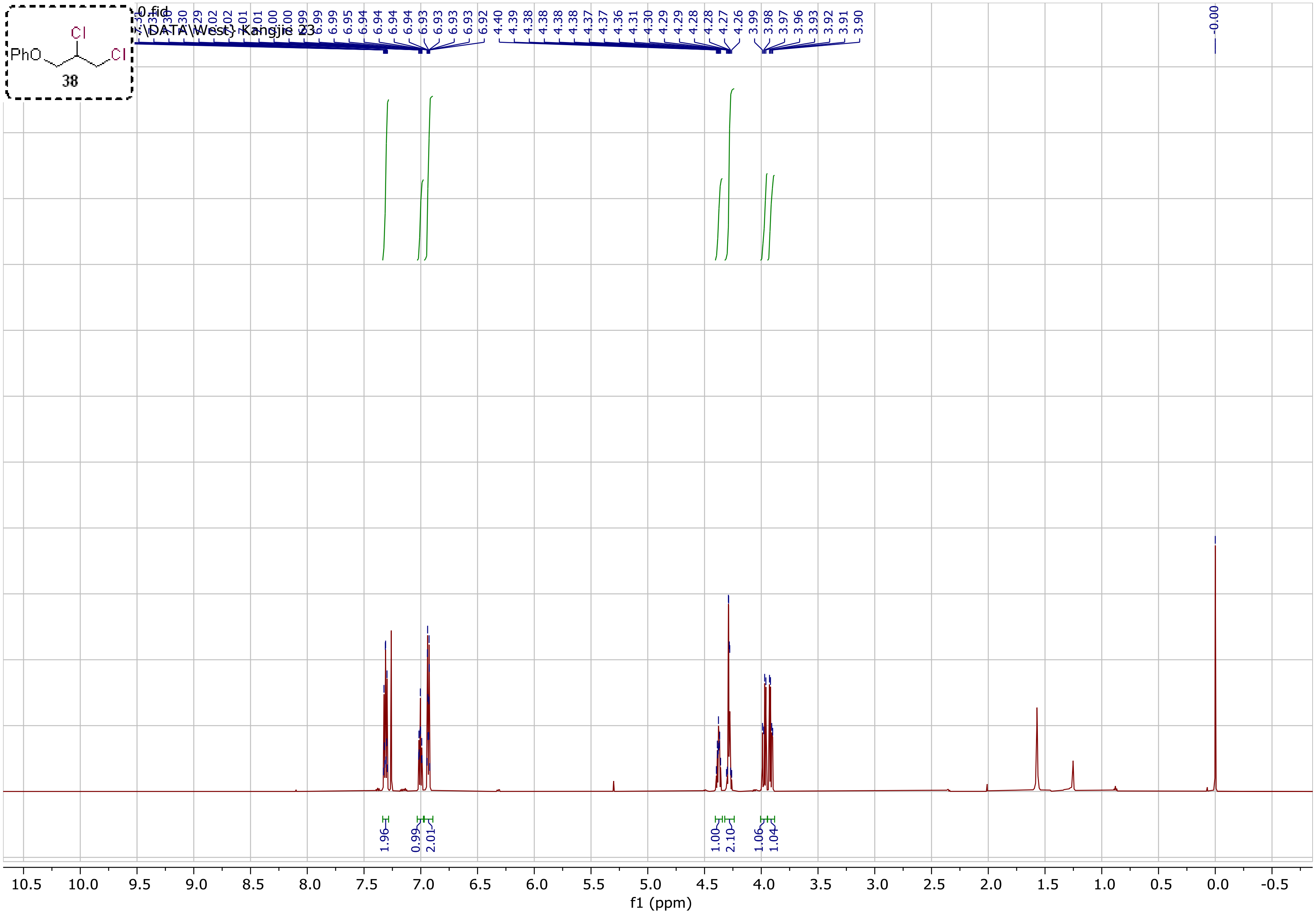

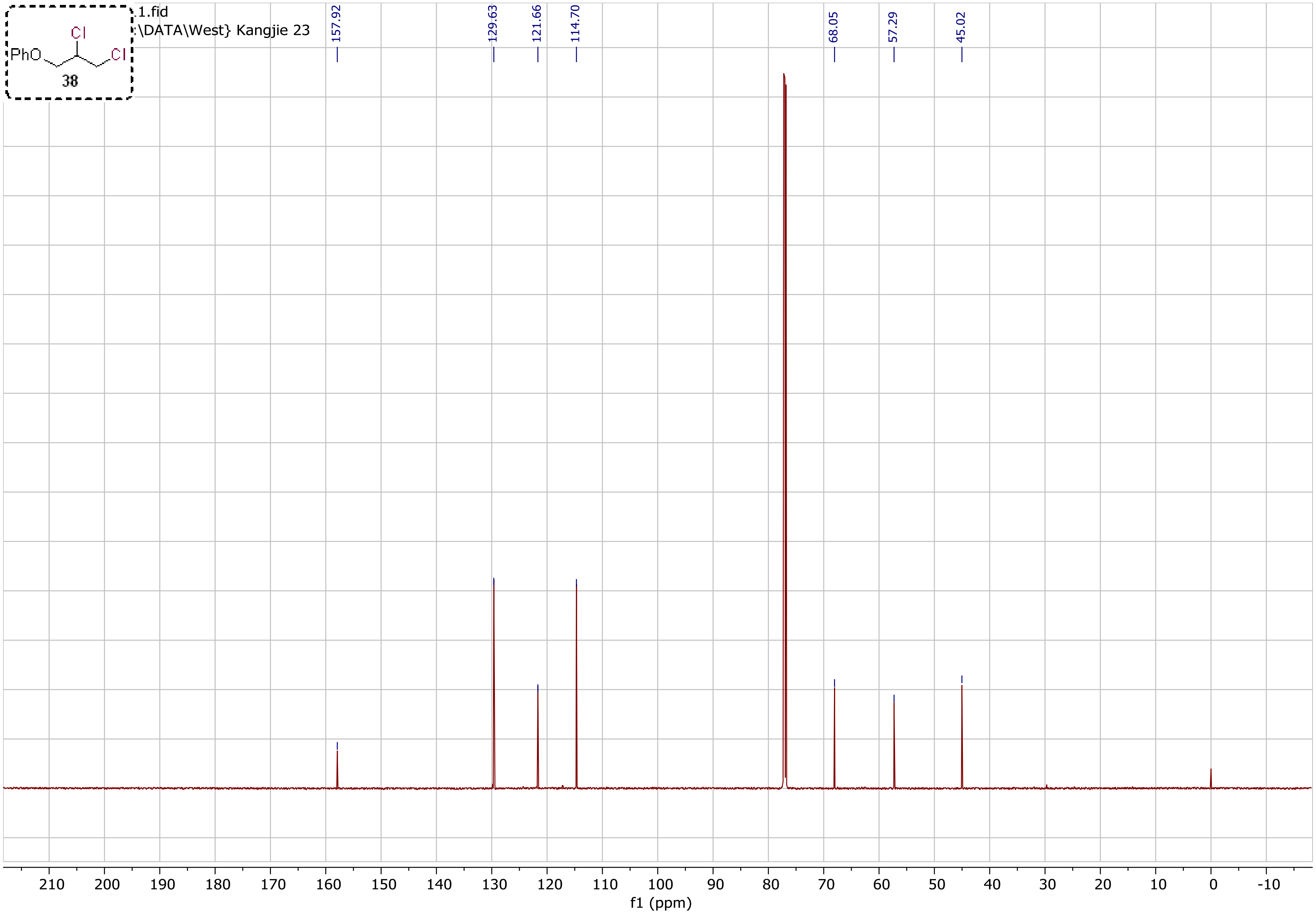
PhO
Cl
Cl
38
1.fid
:\DATA\West} Kangjie 23
157.92
129.63
121.66
114.70
68.05
57.29
45.02
210 200 190 180 170 160 150 140 130 120 110 100 90 80 70 60 50 40 30 20 10 0 -10
f1 (ppm)

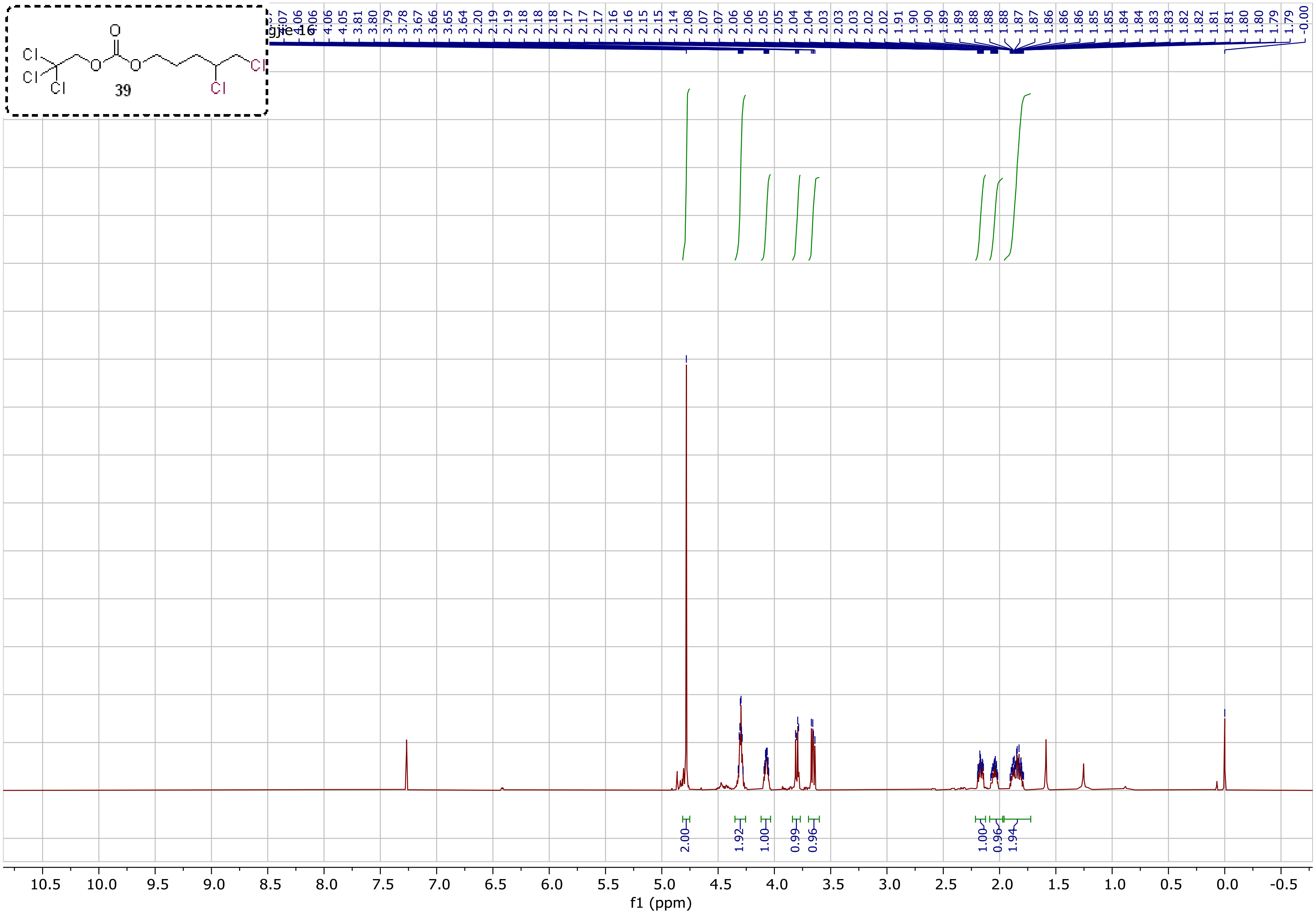

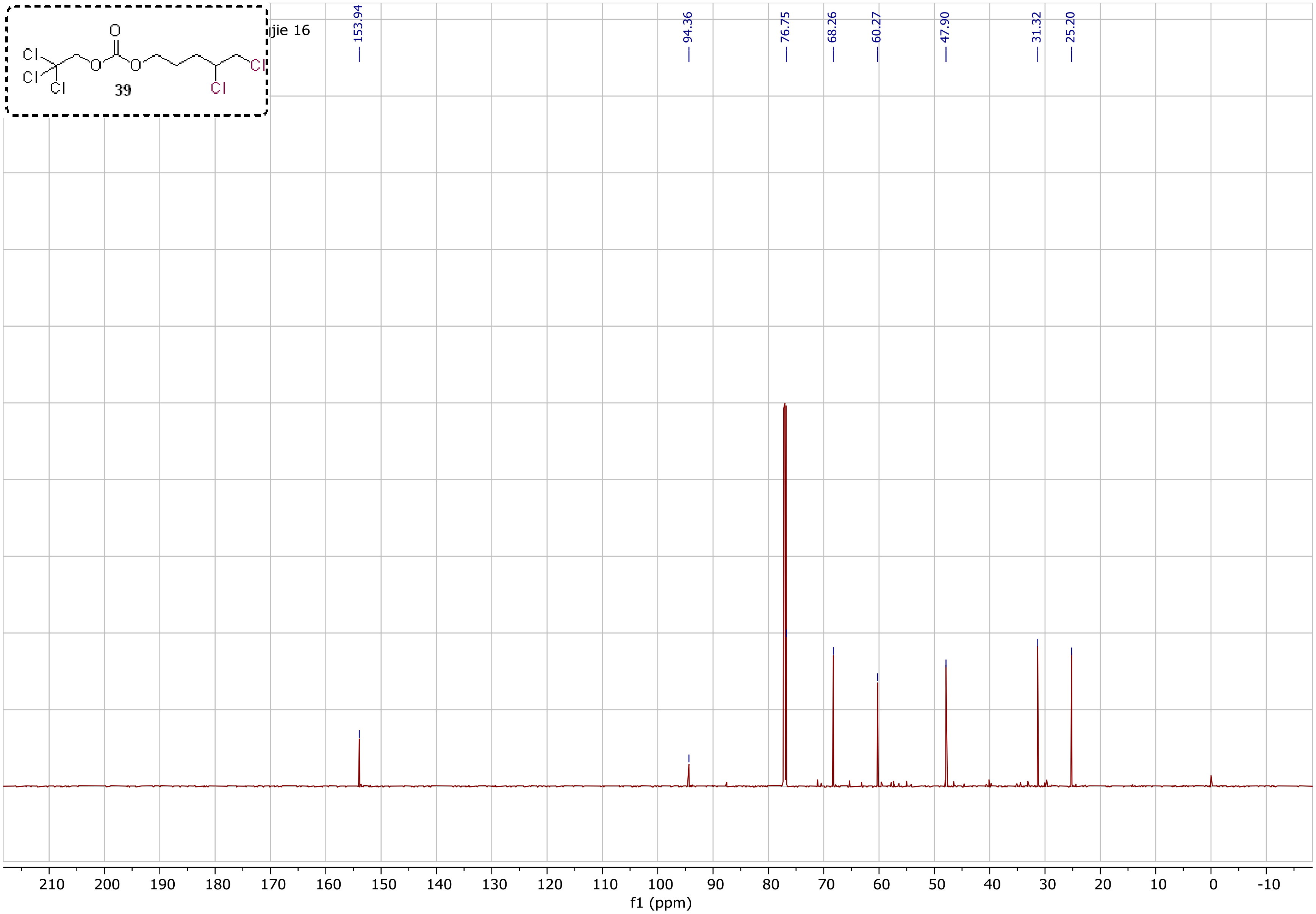

39
jie 16
153.94
94.36
76.75
68.26
60.27
47.90
31.32
25.20
210 200 190 180 170 160 150 140 130 120 110 100 90 80 70 60 50 40 30 20 10 0 -10
f1 (ppm)

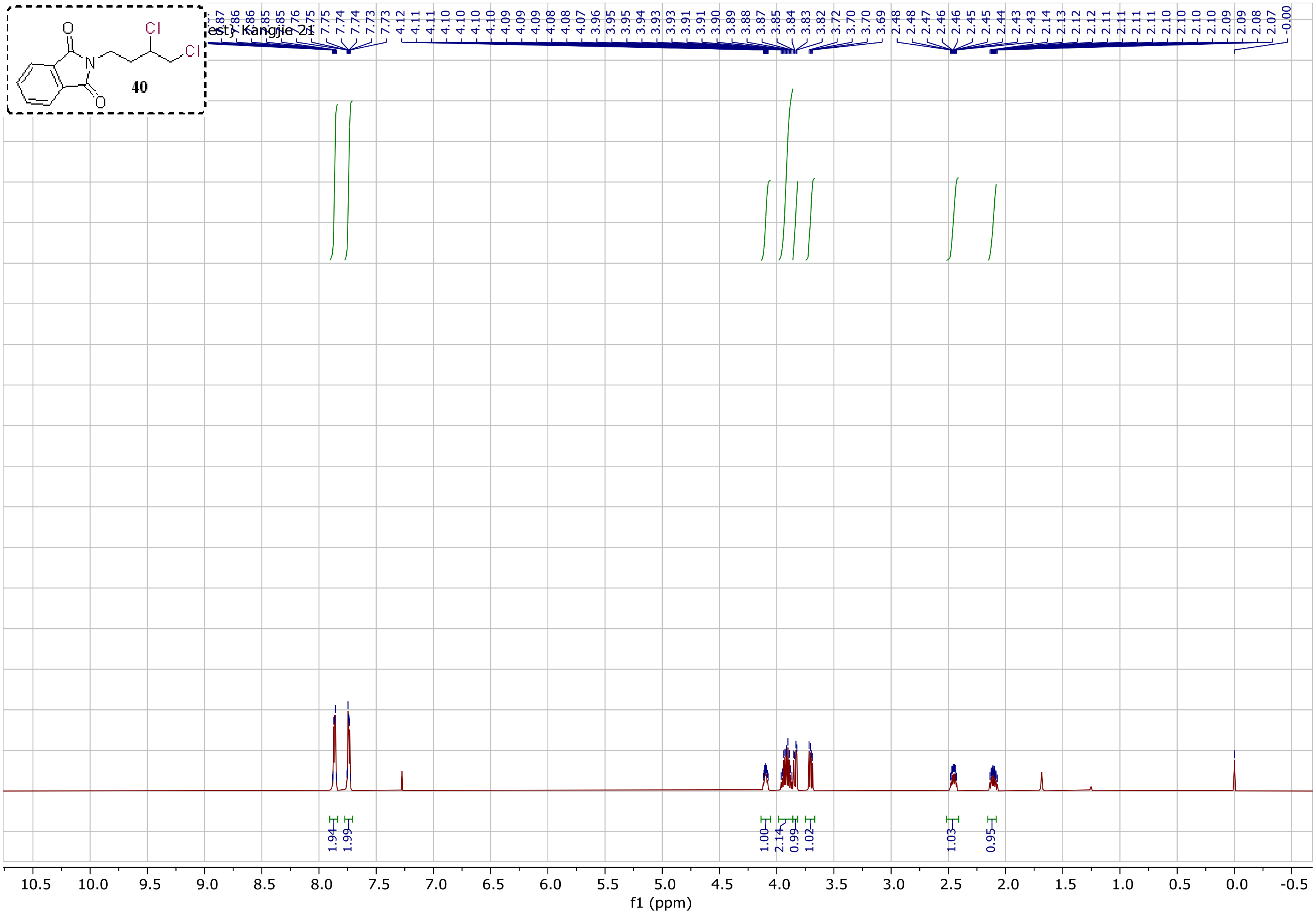

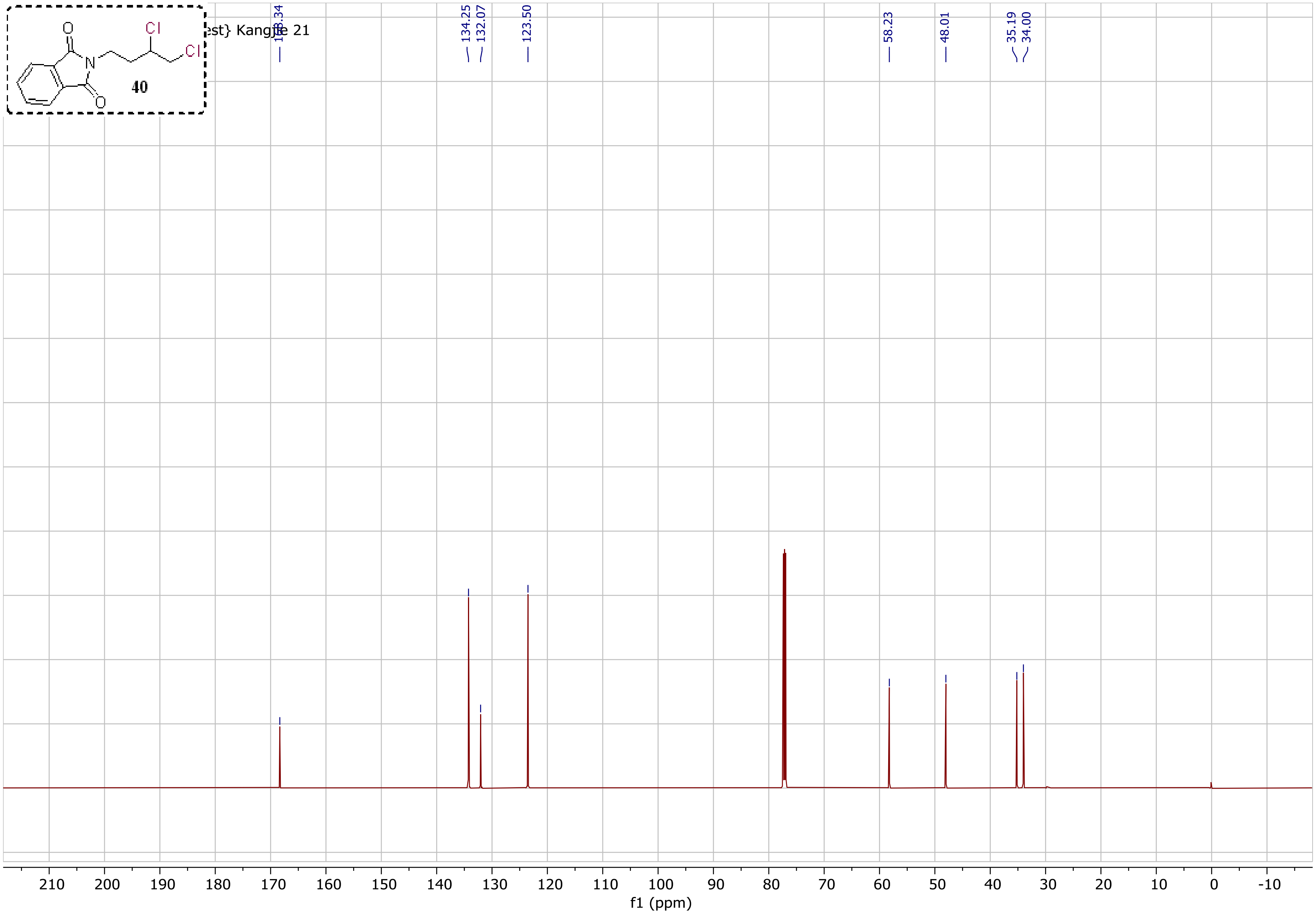
40
Kangjie 21
134.25
132.07
123.50
58.23
48.01
35.19
34.00
210 200 190 180 170 160 150 140 130 120 110 100 90 80 70 60 50 40 30 20 10 0 -10
f1 (ppm)

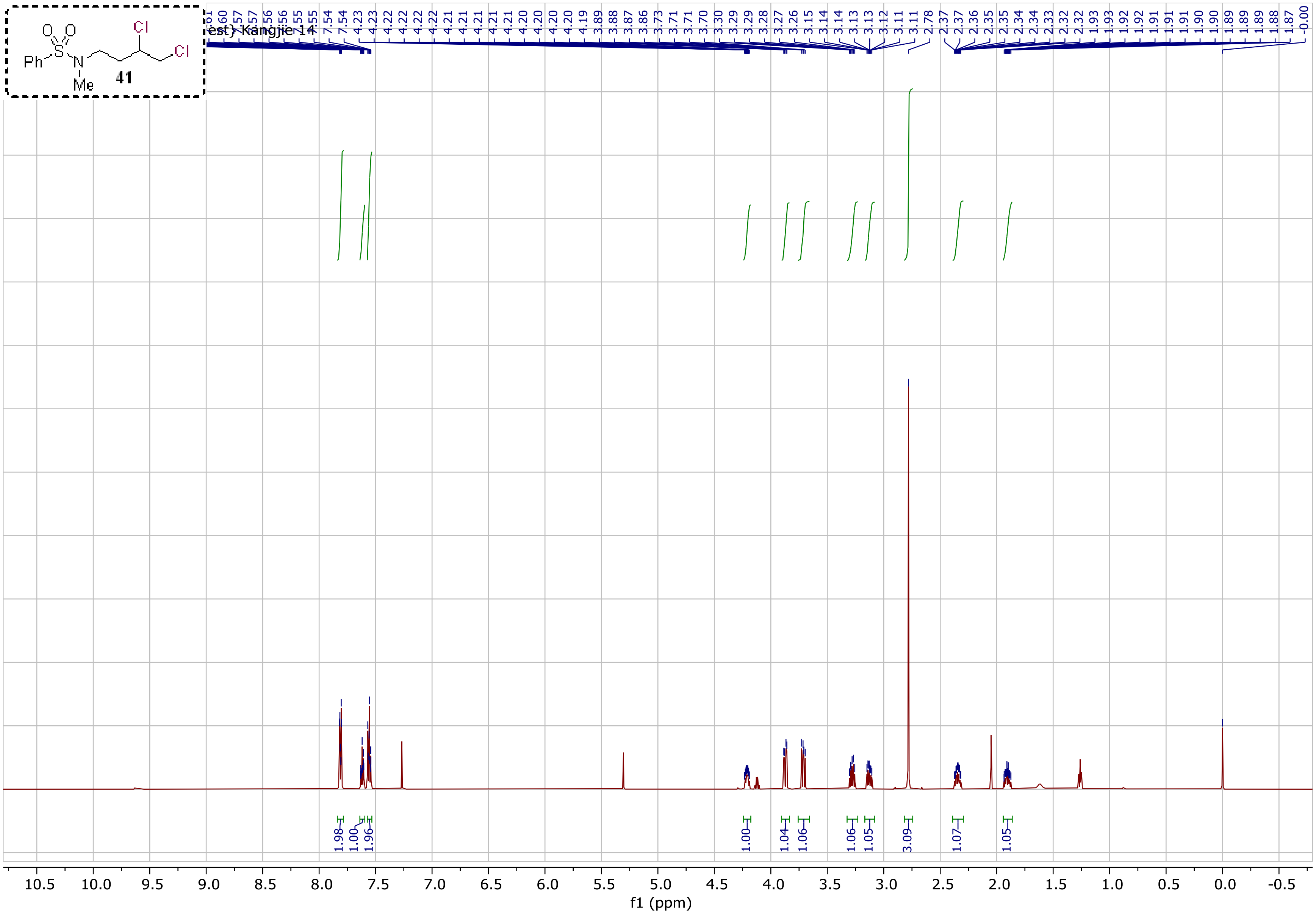
Ph
N
Me
Cl
41
f1 (ppm)

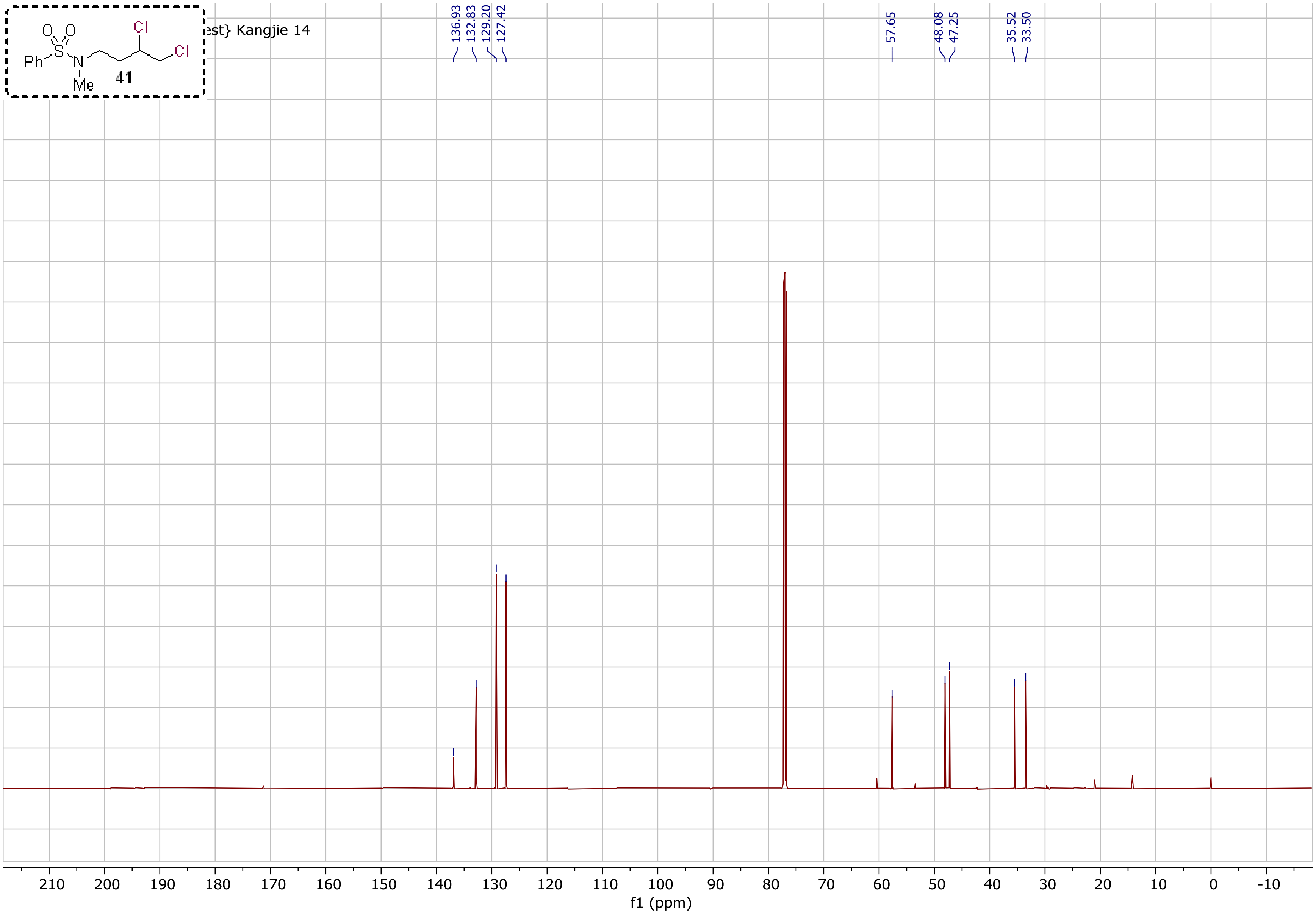

O O
S
Ph
N
Me
Cl
Cl
41
est} Kangjie 14
136.93
132.83
129.20
127.42
57.65
48.08
47.25
35.52
33.50
210 200 190 180 170 160 150 140 130 120 110 100 90 80 70 60 50 40 30 20 10 0 -10
f1 (ppm)

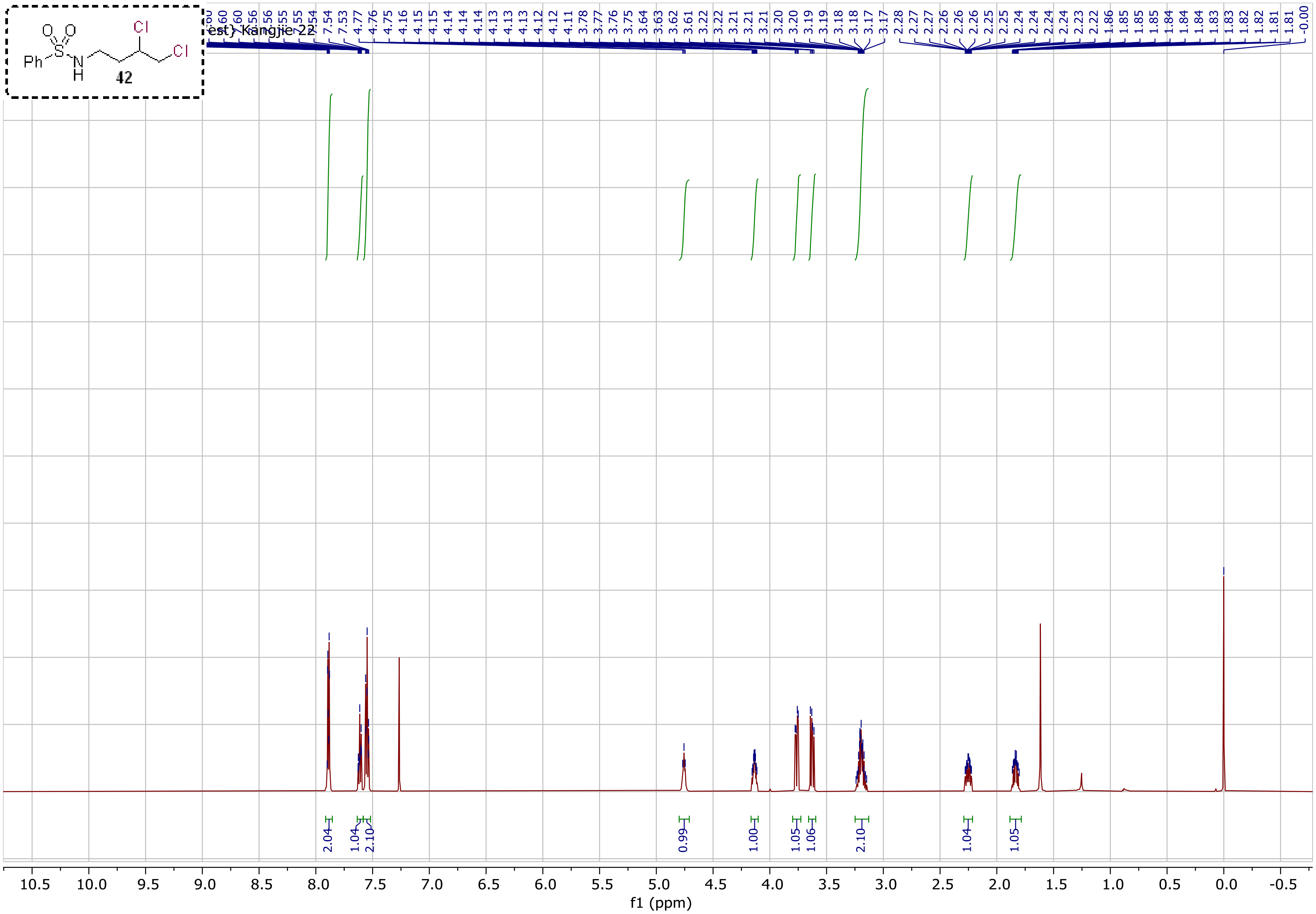

42
f1 (ppm)
10.5 10.0 9.5 9.0 8.5 8.0 7.5 7.0 6.5 6.0 5.5 5.0 4.5 4.0 3.5 3.0 2.5 2.0 1.5 1.0 0.5 0.0 -0.5
2.04 1.04 2.10 0.99 1.00 1.05 1.06 2.10 1.04 1.05

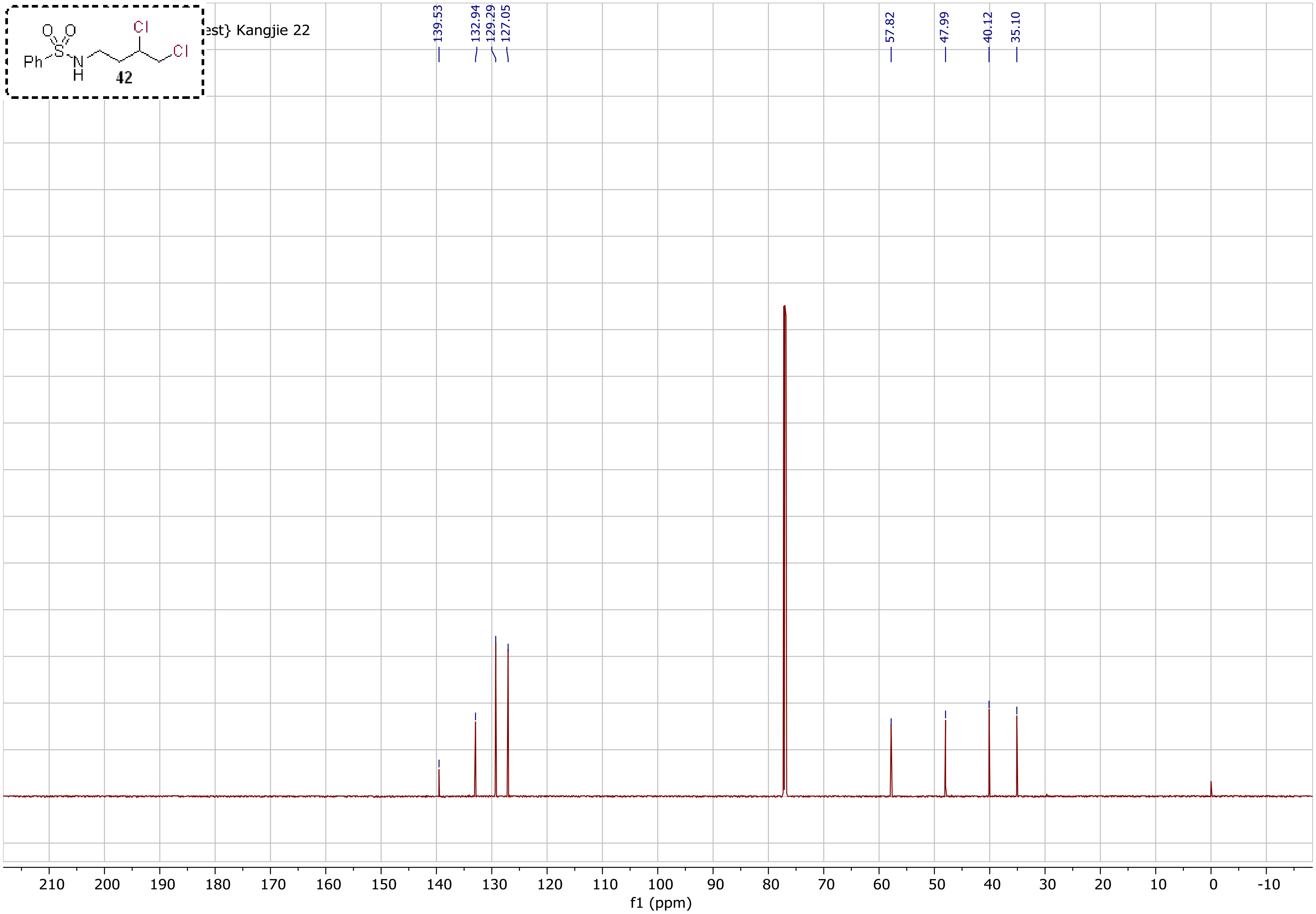

Ph
O
O
S
N
H
Cl
Cl
42
est} Kangjie 22
139.53
132.94
129.29
127.05
57.82
47.99
40.12
35.10
210
200
190
180
170
160
150
140
130
120
110
100
90
80
70
60
50
40
30
20
10
0
-10
f1 (ppm)

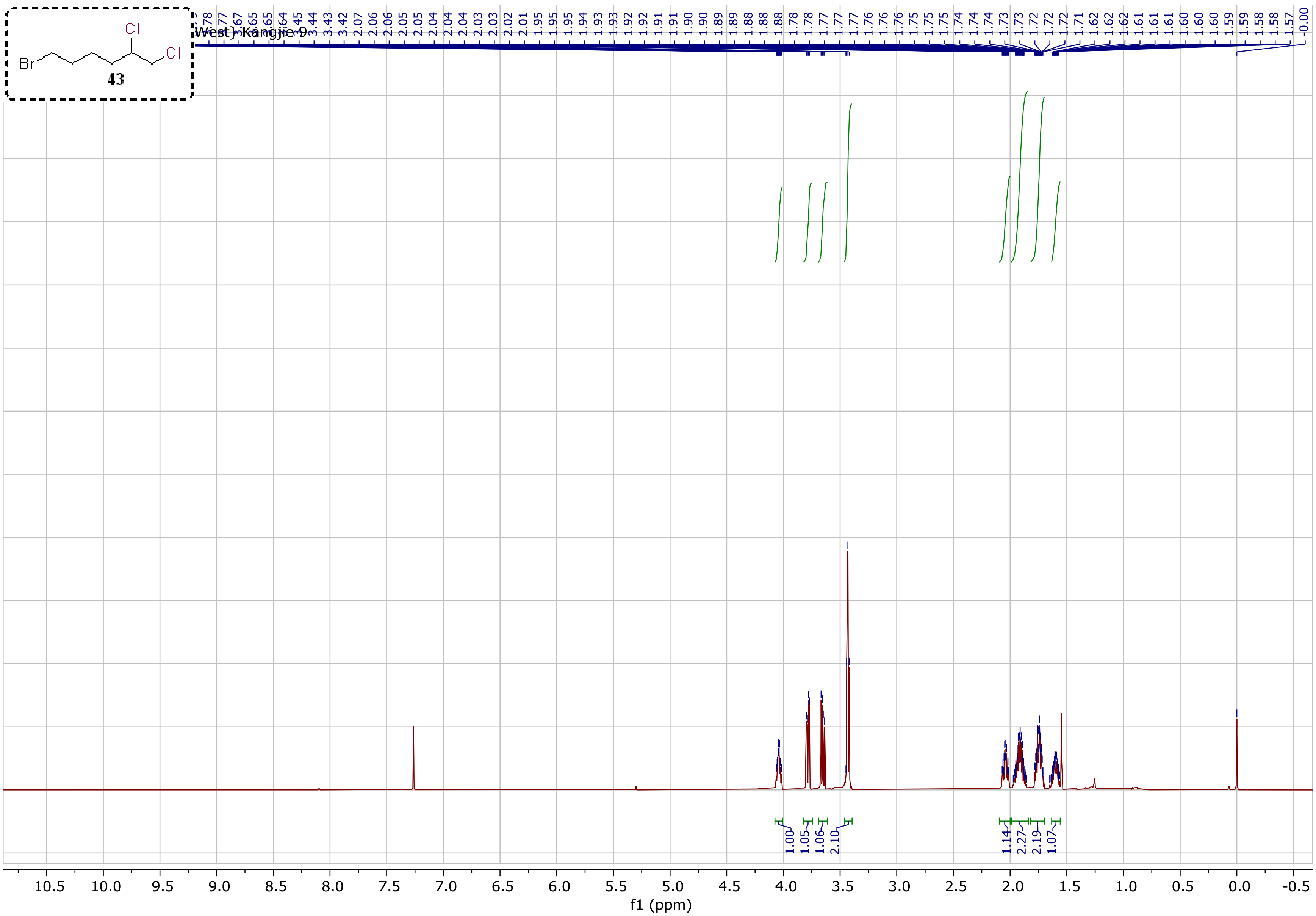
Br
Cl
Cl
43
West} Kangjie 9
f1 (ppm)

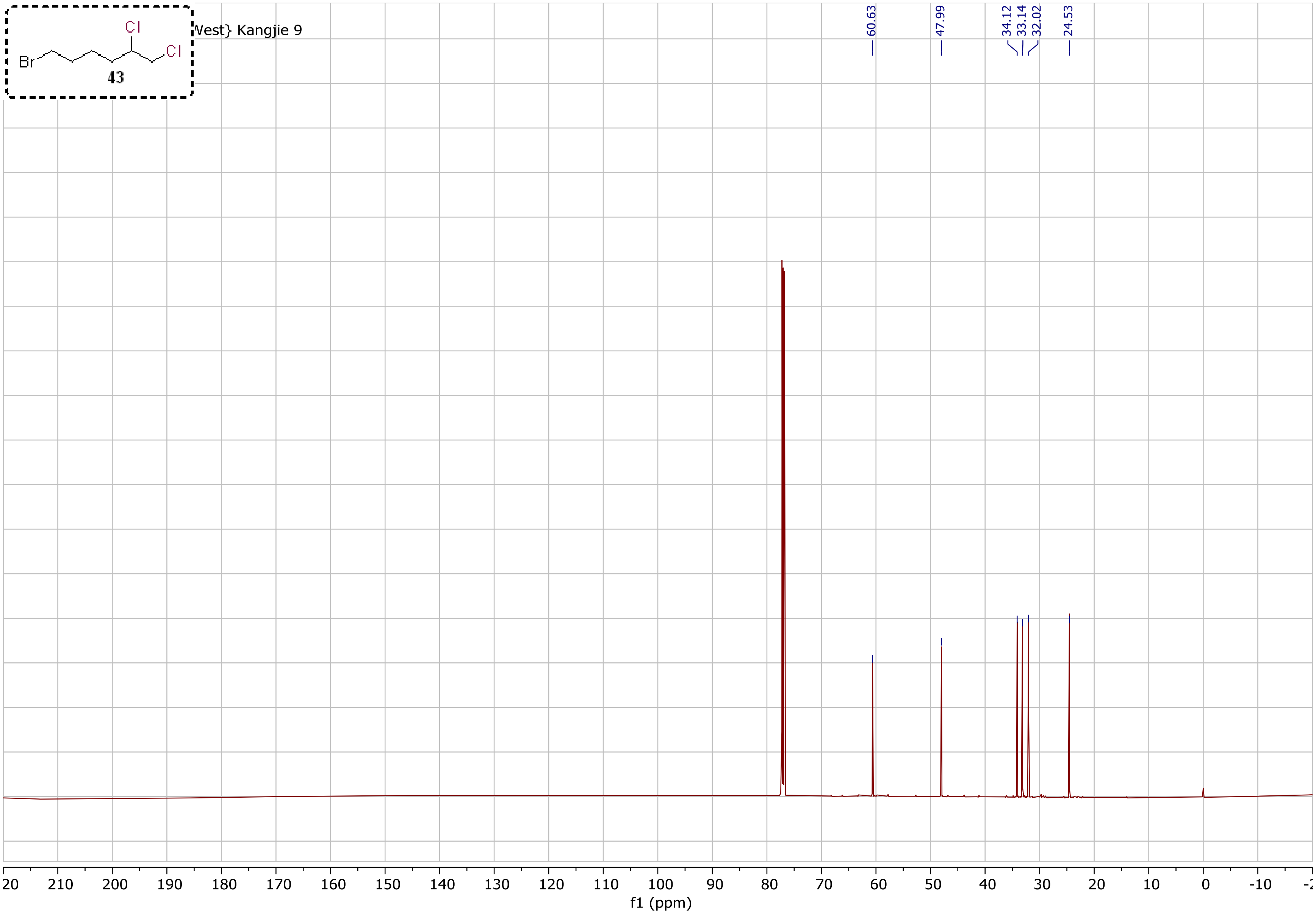
Br
Cl
Cl
43
West} Kangjie 9
60.63
47.99
34.12
33.14
32.02
24.53
f1 (ppm)

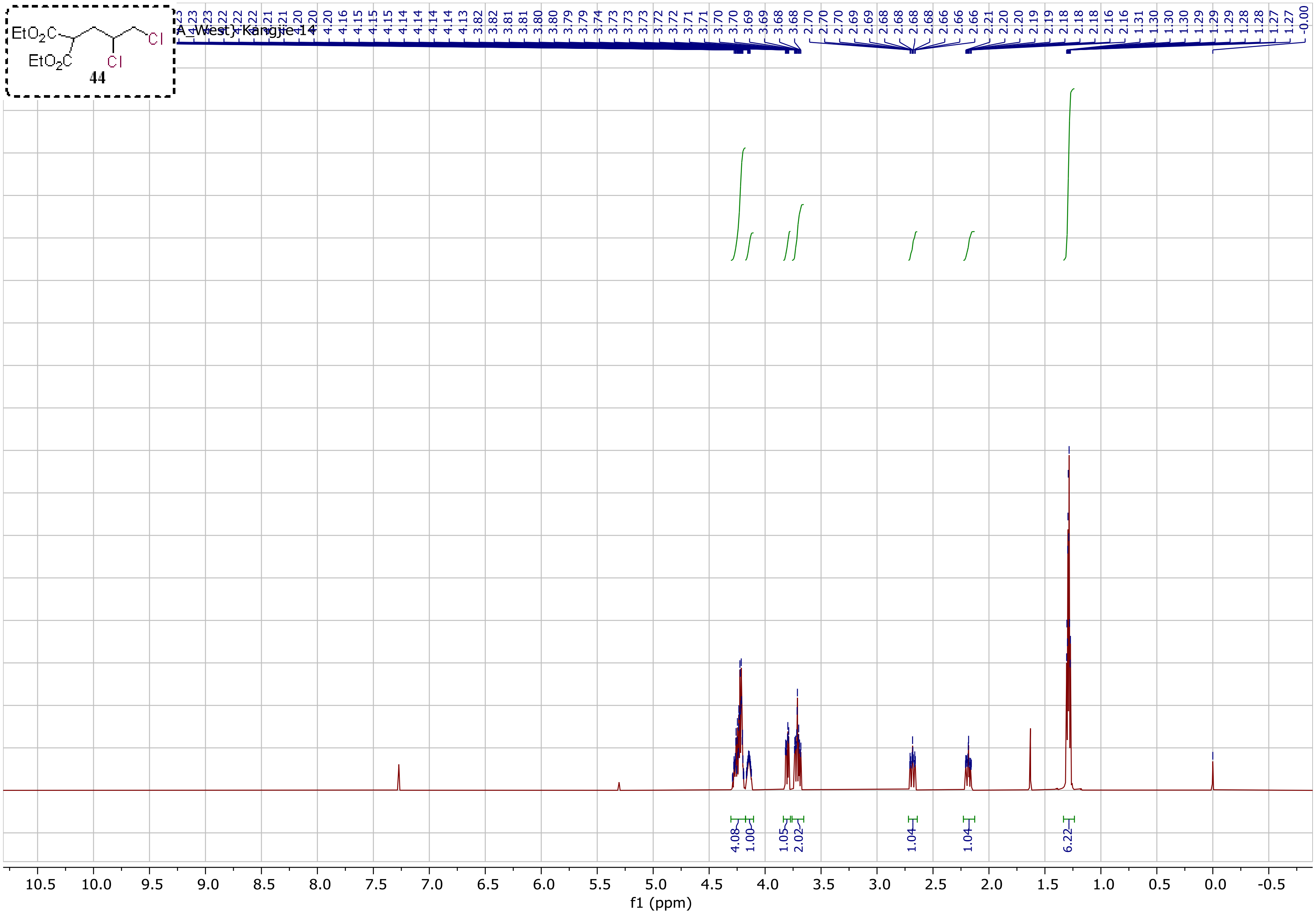
EtO2C
EtO2C
Cl
Cl
44
A_West}-Kangjie-14
f1 (ppm)

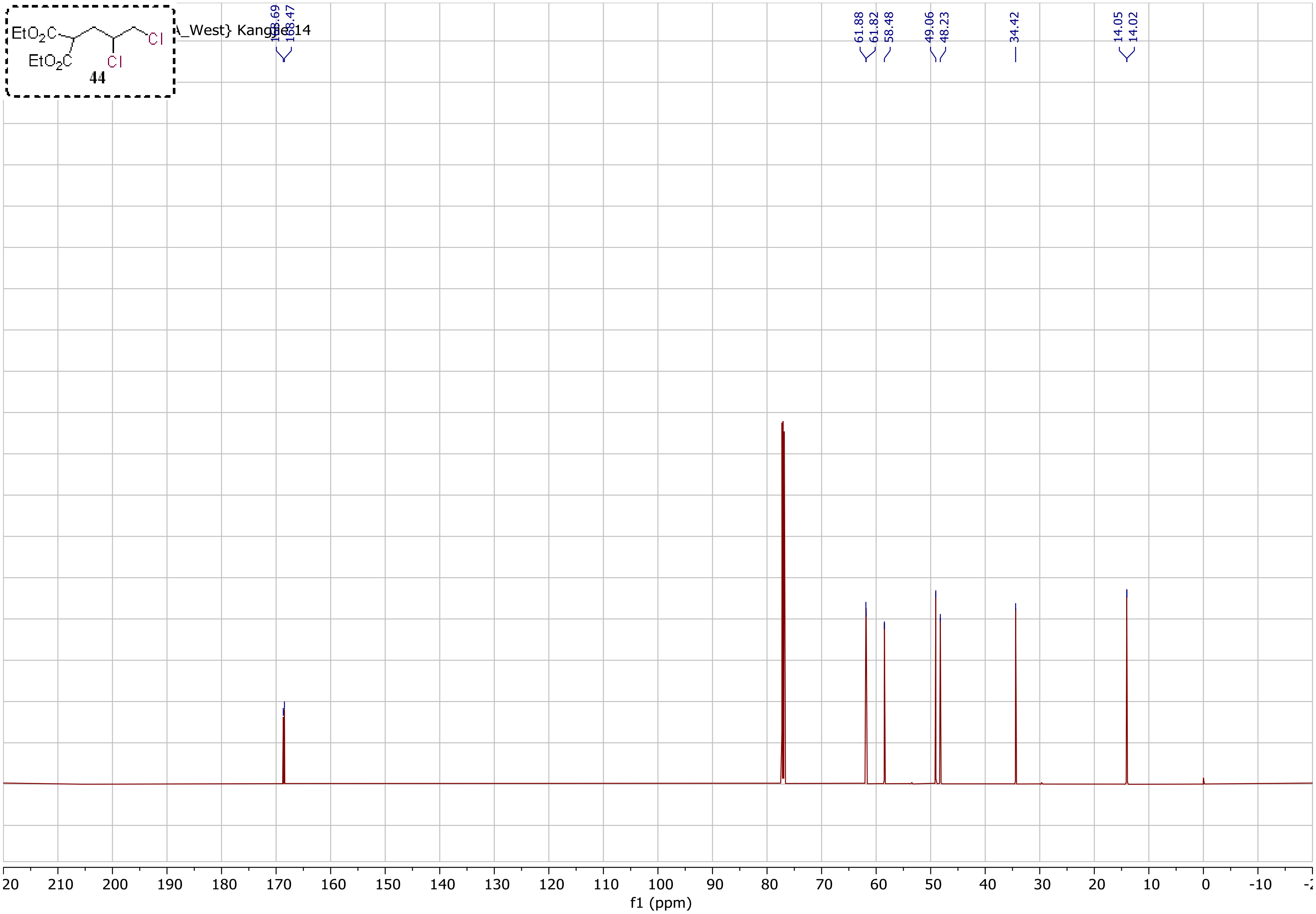

EtO2C
EtO2C
Cl
Cl
44
_West} Kangde14
168.69
168.47
61.88
61.82
58.48
49.06
48.23
34.42
14.05
14.02
f1 (ppm)

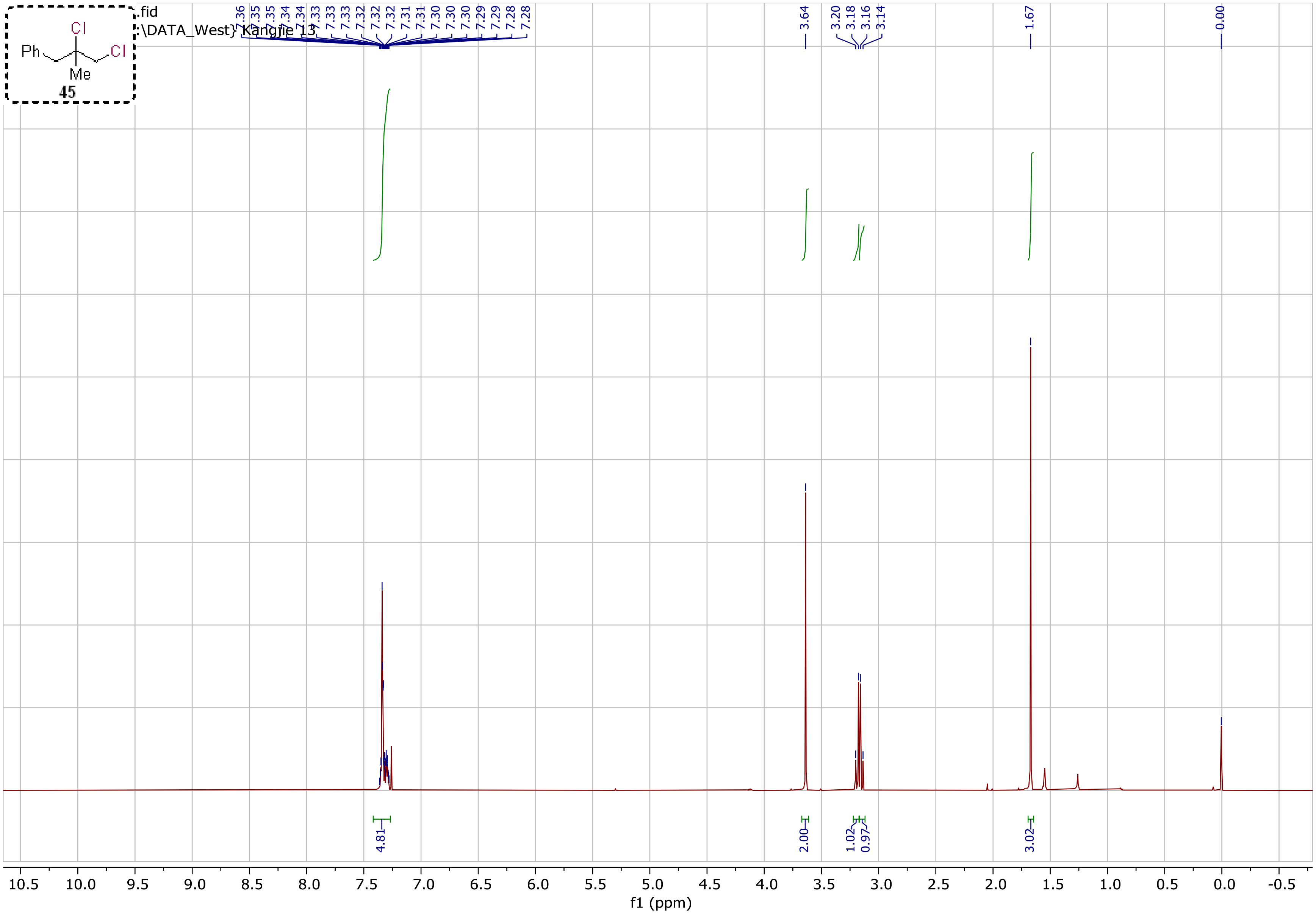
Cl
Ph
Cl
Me
45
.fid
:\DATA_West} Kangjie 13
7.36
7.35
7.35
7.34
7.34
7.33
7.33
7.33
7.32
7.32
7.32
7.31
7.31
7.30
7.30
7.30
7.29
7.29
7.28
7.28
3.64
3.20
3.18
3.16
3.14
1.67
0.00
4.81
2.00
1.02
0.97
3.02
f1 (ppm)

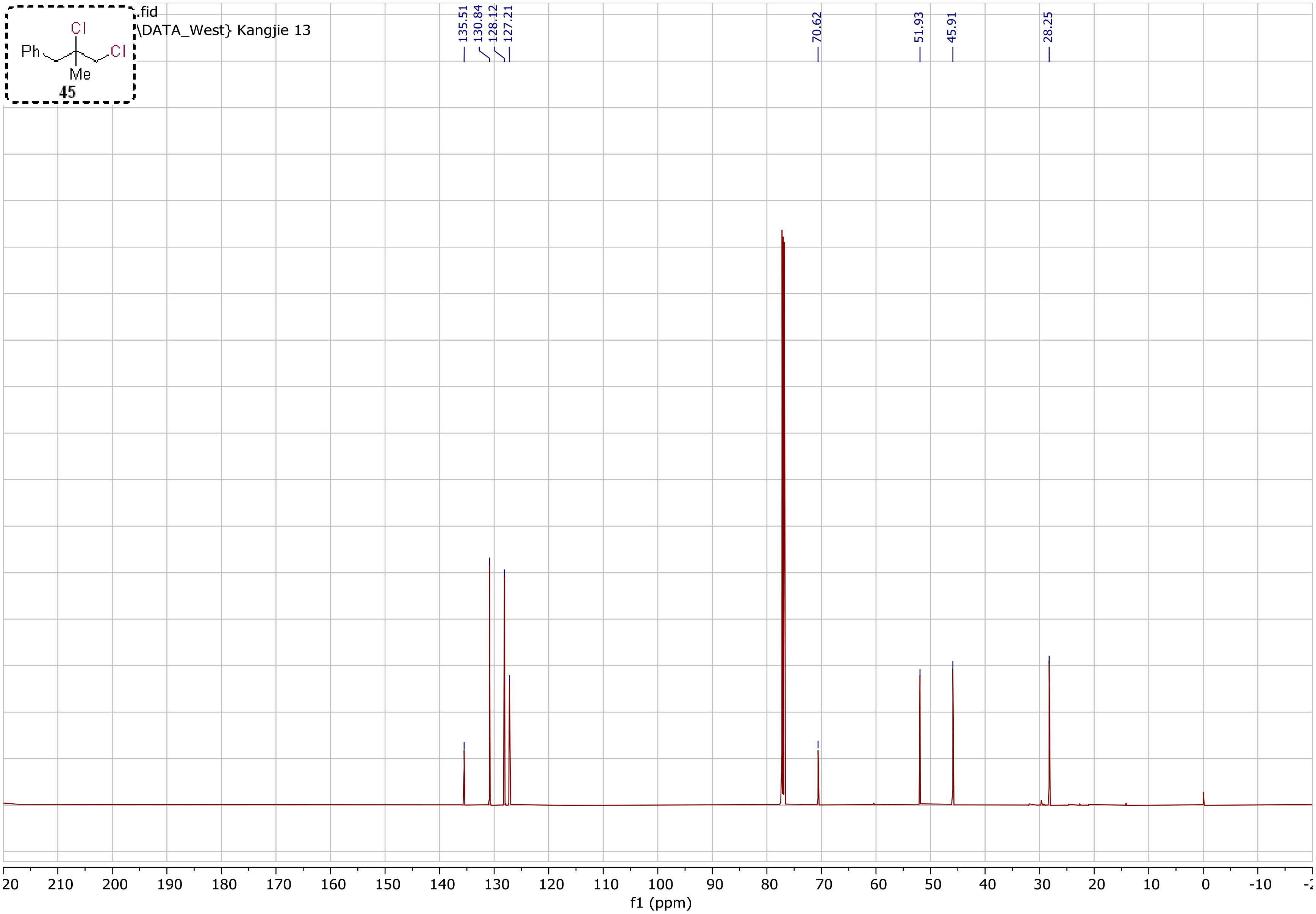
.fid
\DATA_West} Kangjie 13
Cl
Ph
Cl
Me
45
135.51
130.84
128.12
127.21
70.62
51.93
45.91
28.25
f1 (ppm)

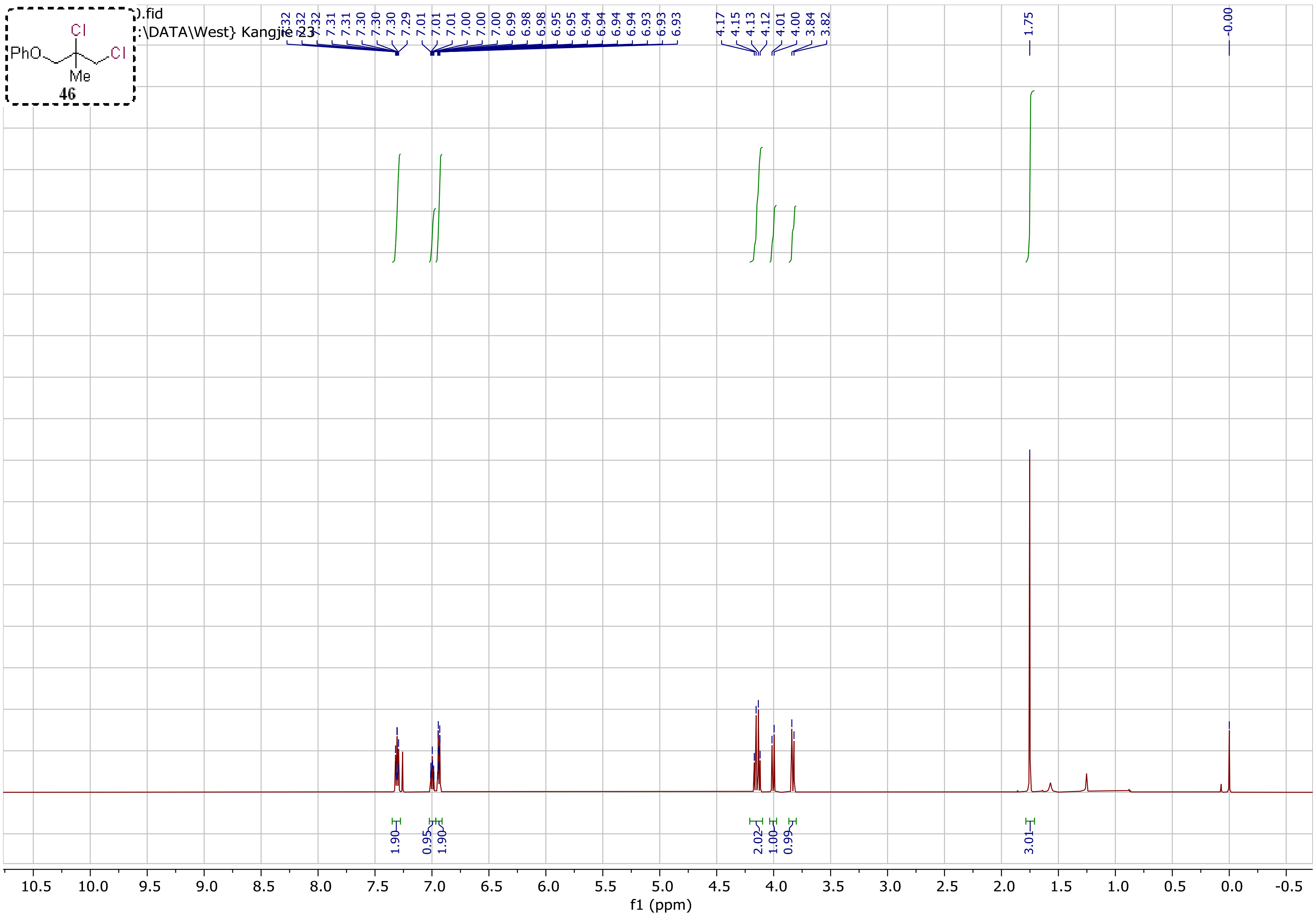
PhO
Cl
Cl
Me
46
).fid
:\DATA\West} Kangjie 23
f1 (ppm)
1.90
0.95
1.90
2.02
1.00
0.99
3.01
1.75
-0.00

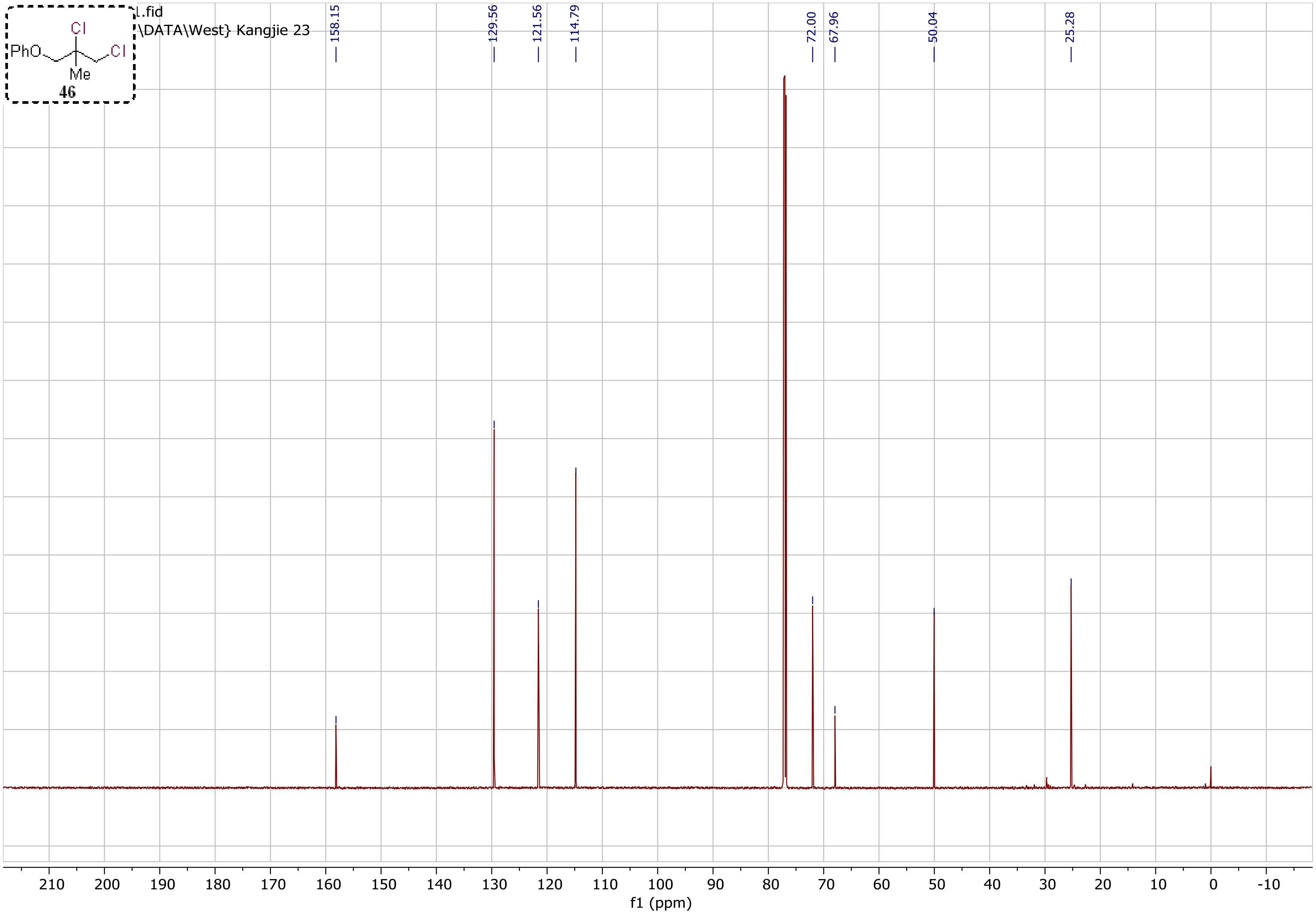

1.fid
\DATA\West} Kangjie 23
PhO
Cl
Cl
Me
46
158.15
129.56
121.56
114.79
72.00
67.96
50.04
25.28
210 200 190 180 170 160 150 140 130 120 110 100 90 80 70 60 50 40 30 20 10 0 -10
f1 (ppm)

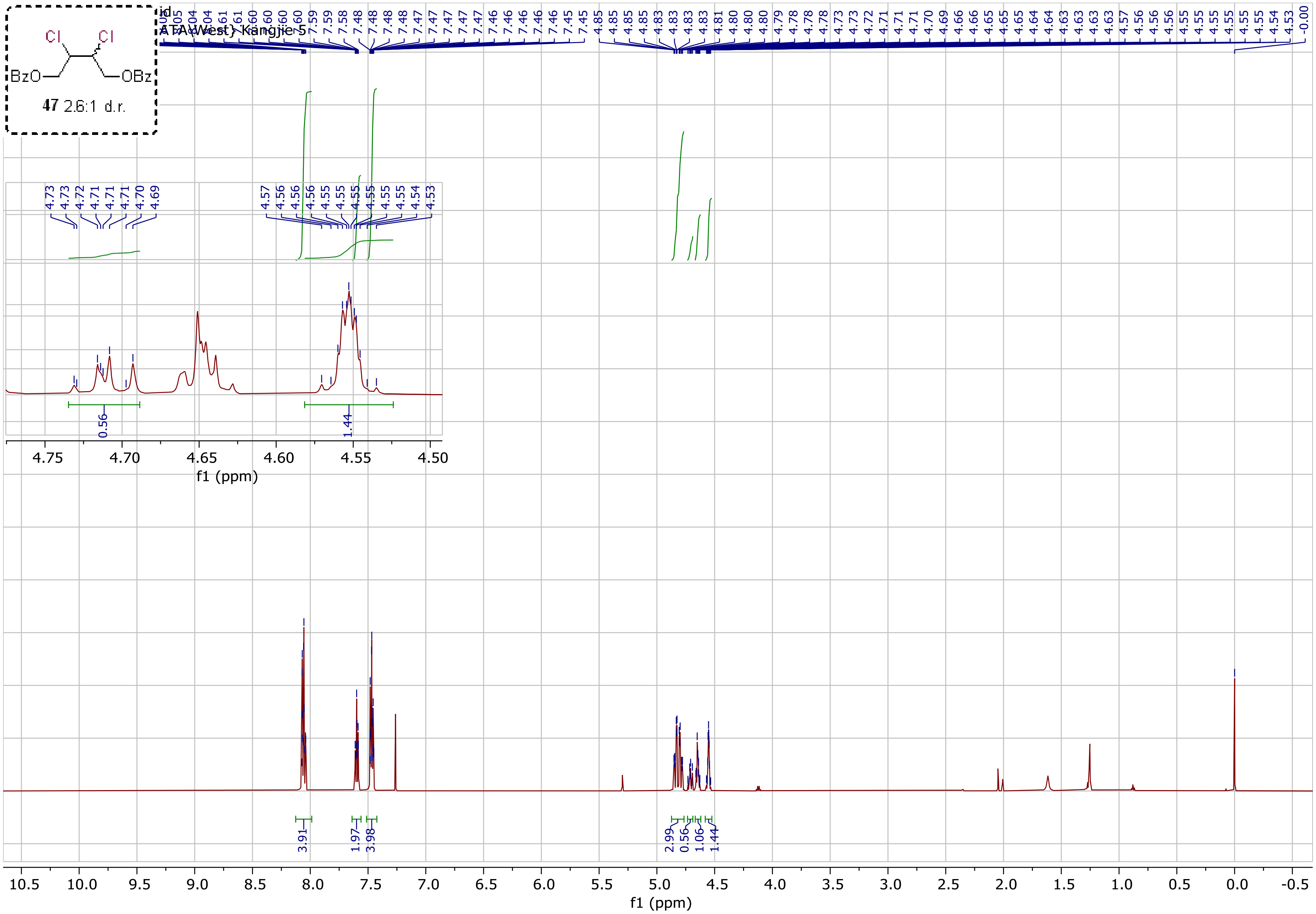

Cl
Cl
BzO
OBz
47 2.6:1 d.r.
f1 (ppm)

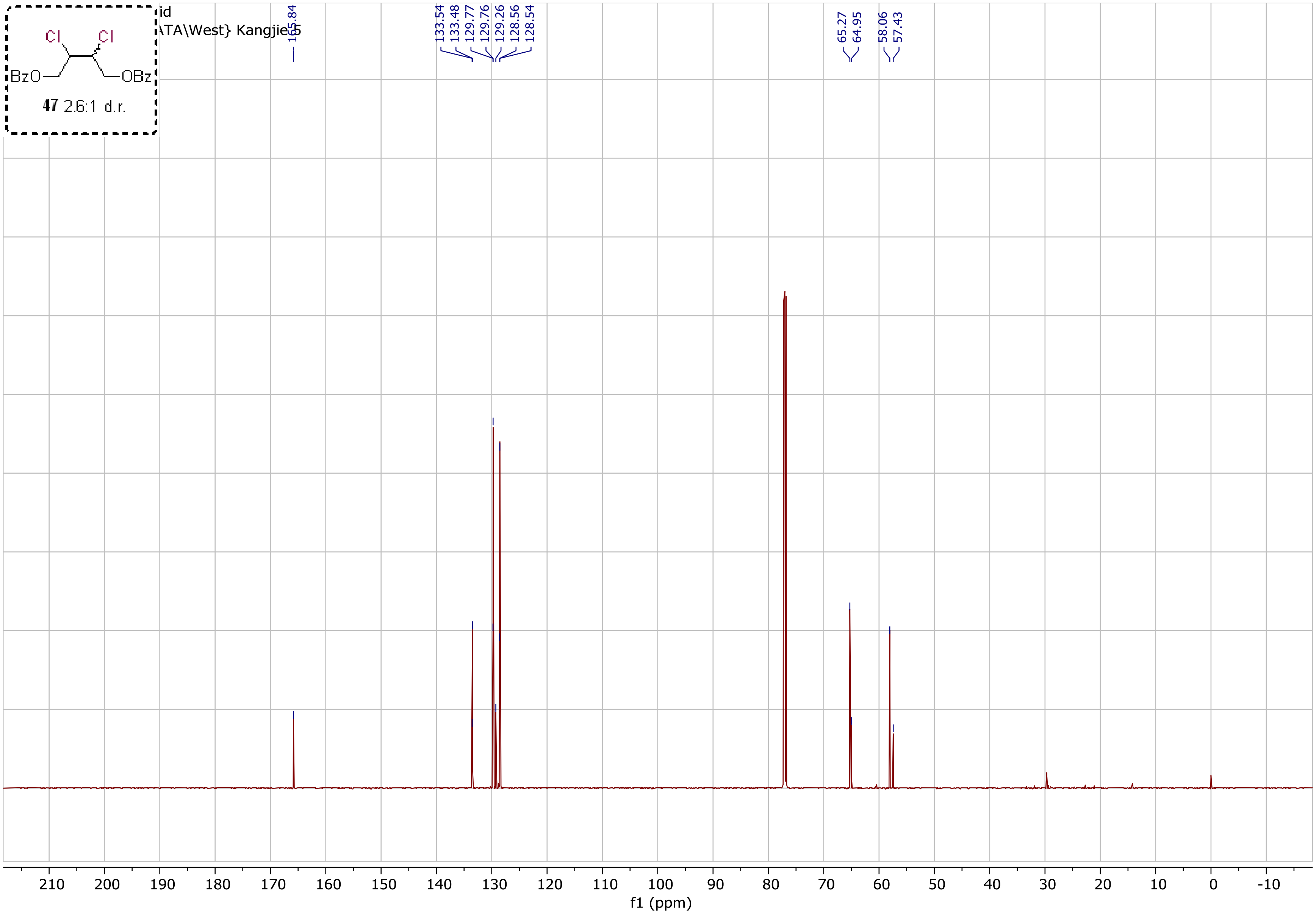

id
\TA\West} Kangjie65
165.84
133.54
133.48
129.77
129.76
129.26
128.56
128.54
65.27
64.95
58.06
57.43
Cl
Cl
BzO
OBz
47 2.6:1 d.r.
210
200
190
180
170
160
150
140
130
120
110
100
90
80
70
60
50
40
30
20
10
0
-10
f1 (ppm)

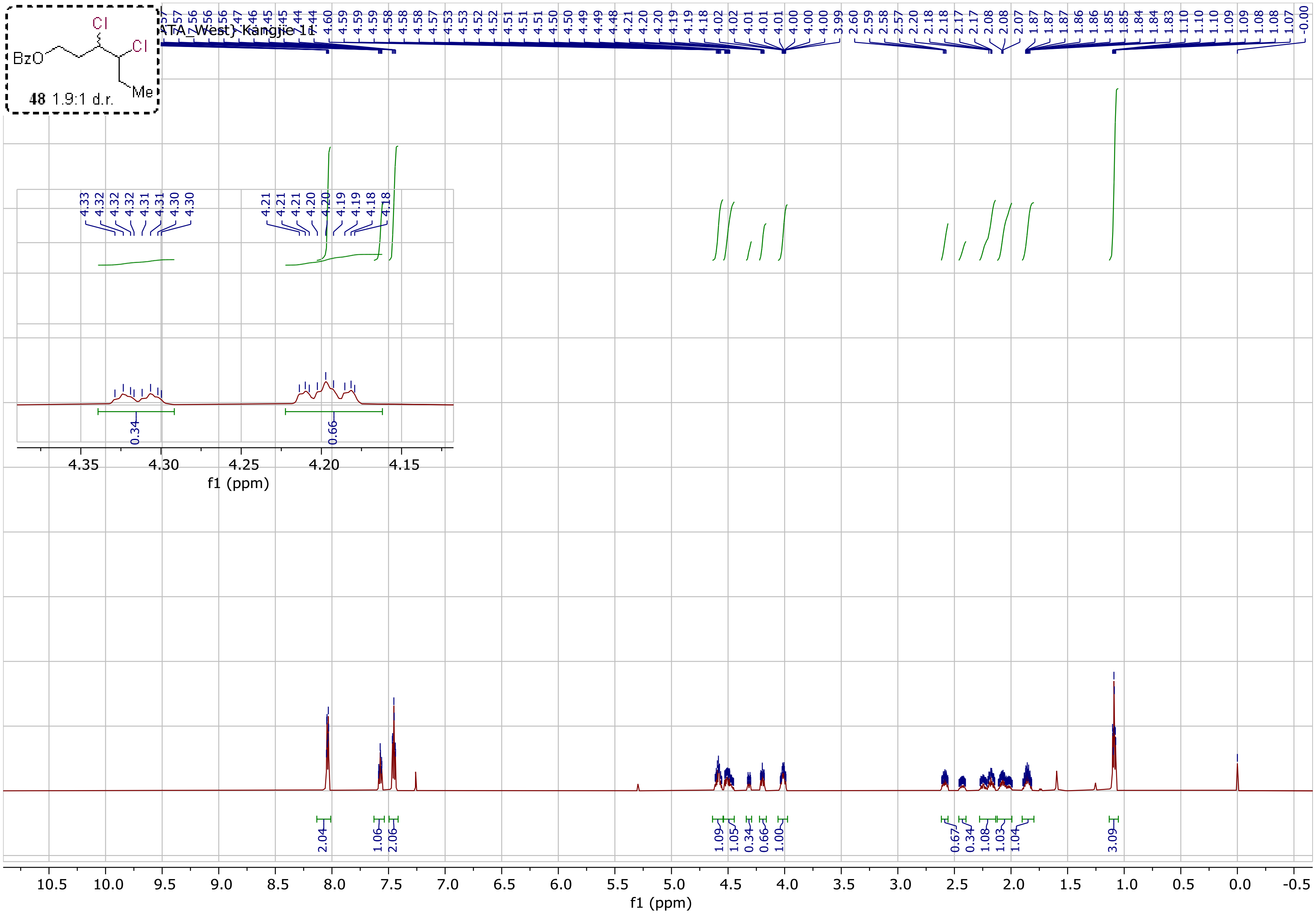

BzO
Cl
Cl
Me
48 1.9:1 d.r.
f1 (ppm)

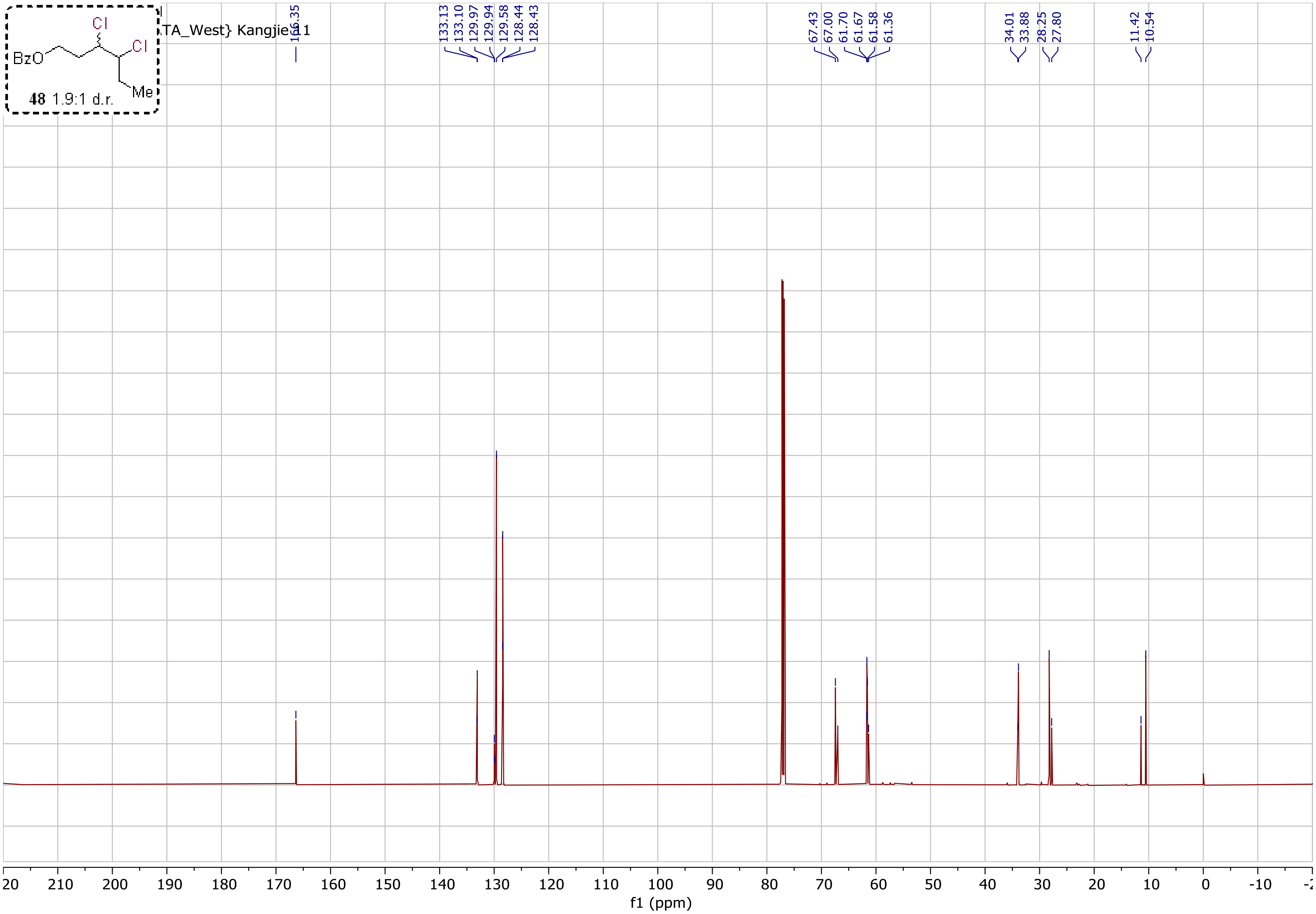
BzO
Cl
Cl
Me
48 1.9:1 d.r.
TA_West} Kangjie 11
166.35
133.13
133.10
129.97
129.94
129.58
128.44
128.43
67.43
67.00
61.70
61.67
61.58
61.36
34.01
33.88
28.25
27.80
11.42
10.54
f1 (ppm)

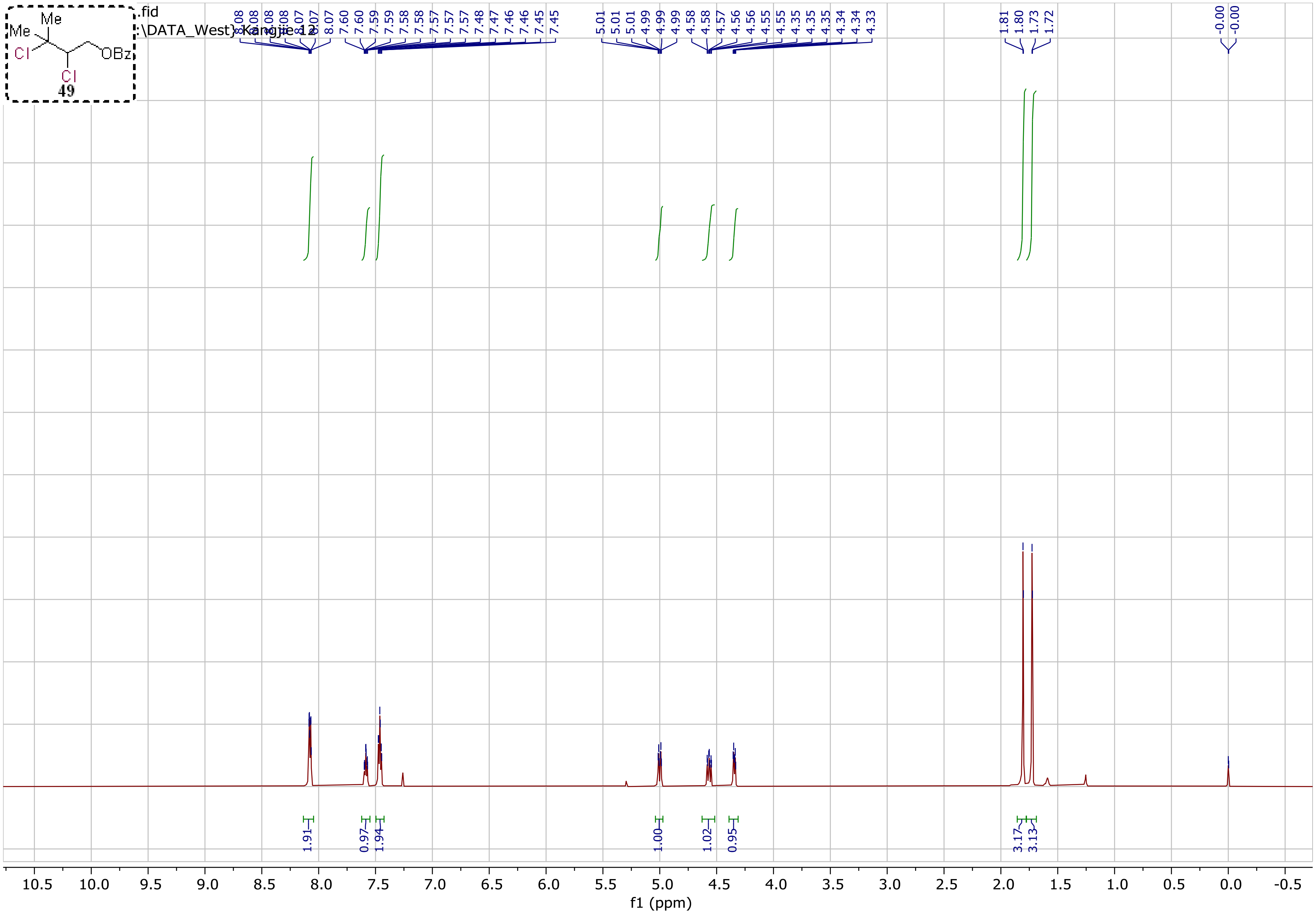
Me
Me
Cl
Cl
OBz
49
.fid
\DATA_West}-Kangjie-12
8.08
8.08
8.08
8.08
8.07
8.07
8.07
7.60
7.59
7.59
7.58
7.57
7.57
7.57
7.48
7.47
7.46
7.45
7.45
5.01
5.01
5.01
4.99
4.99
4.99
4.58
4.58
4.57
4.56
4.56
4.55
4.55
4.35
4.35
4.35
4.34
4.34
4.33
1.81
1.80
1.73
1.72
-0.00
-0.00
1.91
0.97
1.94
1.00
1.02
0.95
3.17
3.13
10.5
10.0
9.5
9.0
8.5
8.0
7.5
7.0
6.5
6.0
5.5
5.0
4.5
4.0
3.5
3.0
2.5
2.0
1.5
1.0
0.5
0.0
-0.5
f1 (ppm)

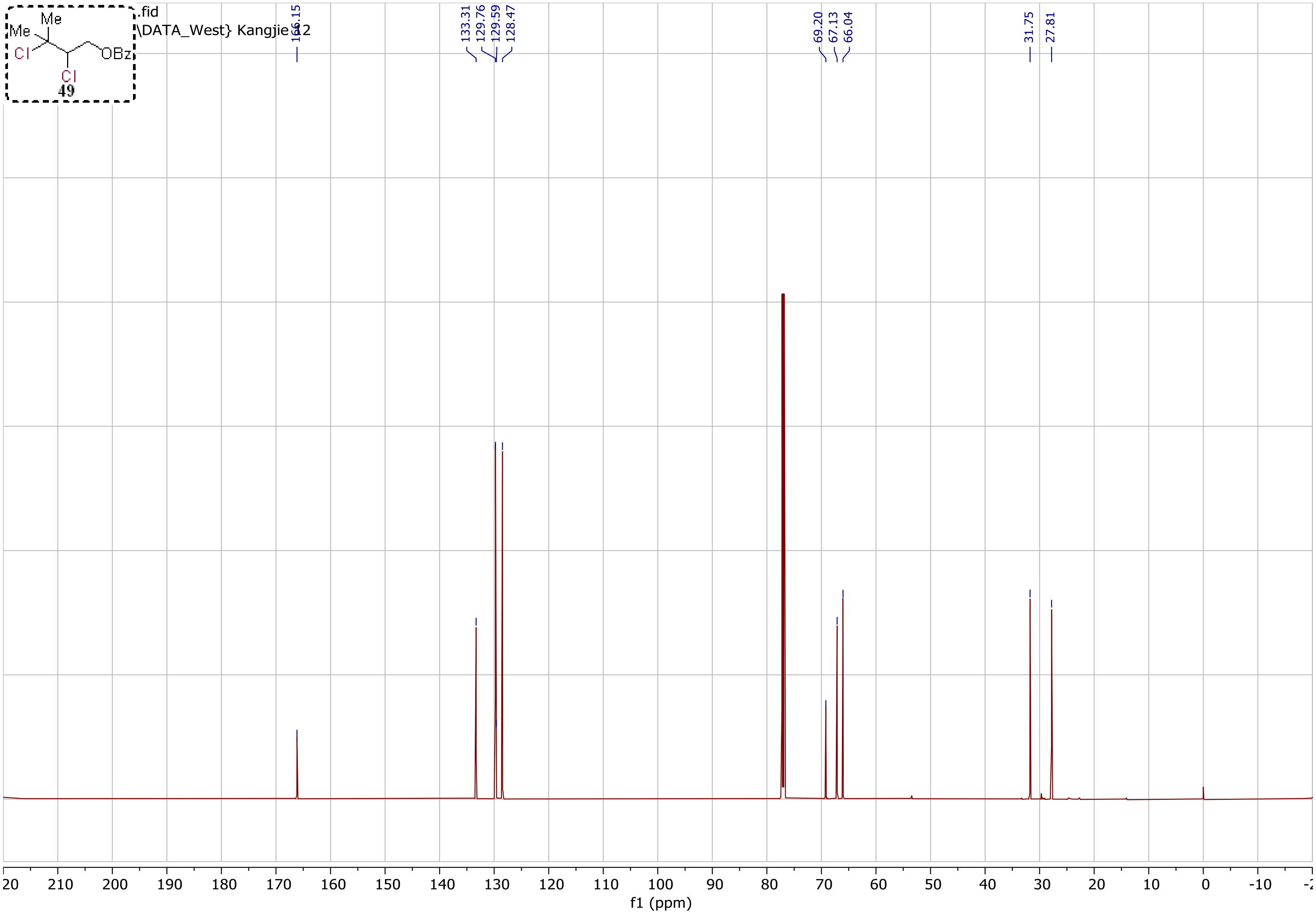

Me
Me
Cl
Cl
OBz
49
.fid
\DATA_West} Kangjie 12
166.15
133.31
129.76
129.59
128.47
69.20
67.13
66.04
31.75
27.81
f1 (ppm)

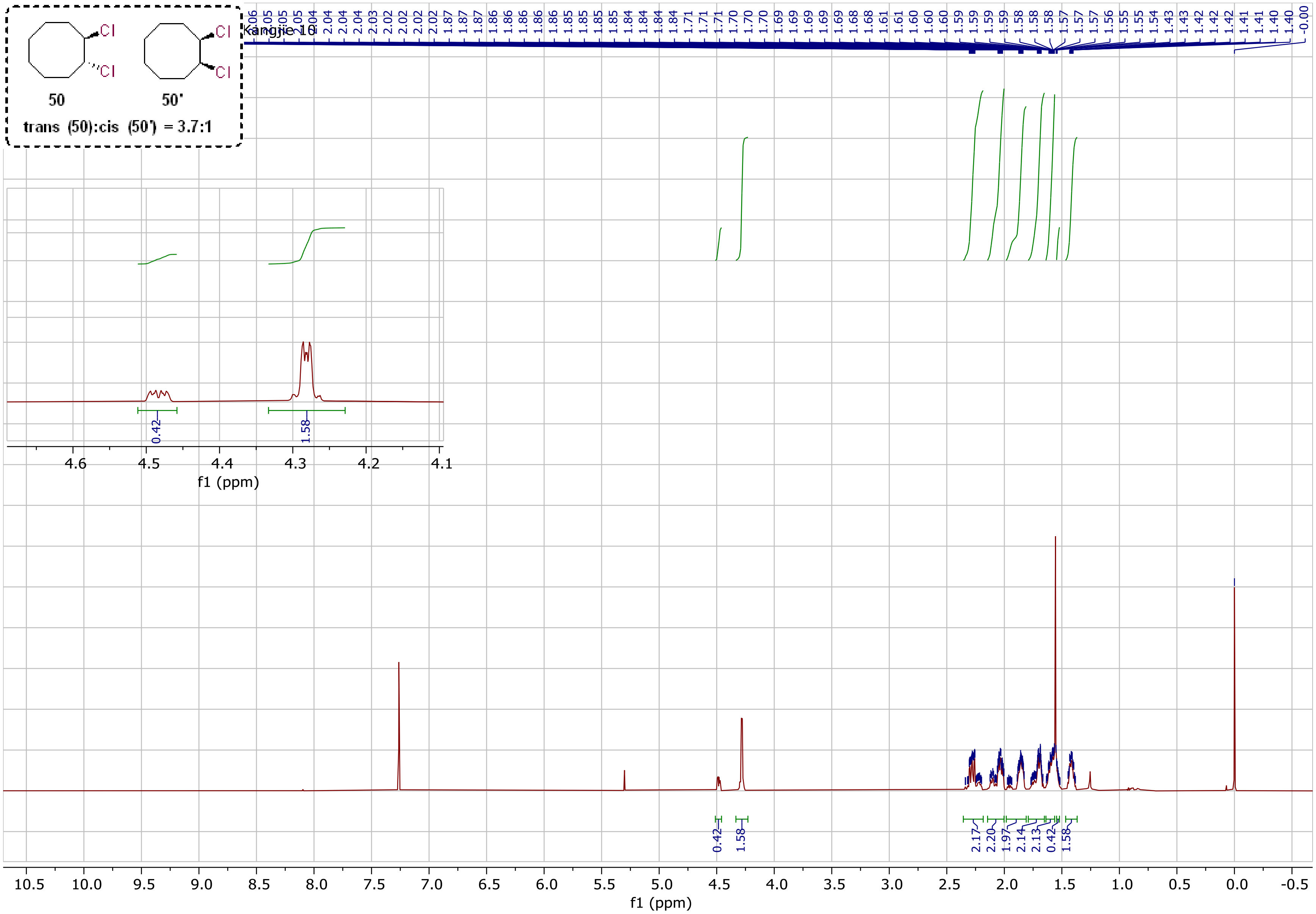

50
50'
trans (50):cis (50') = 3.7:1
Kangjie 10
f1 (ppm)

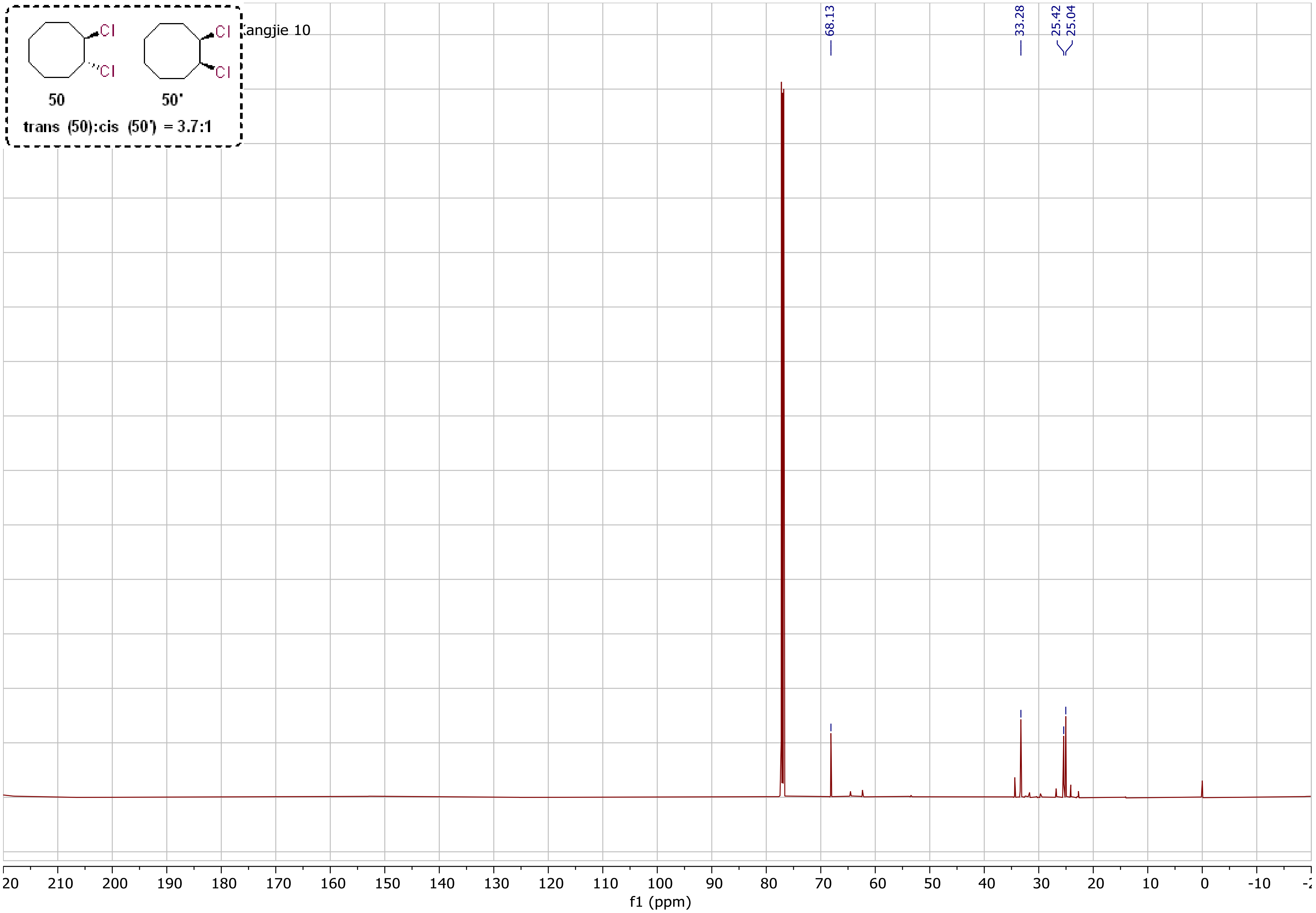

Cl
Cl
Cl
Cl
50
50'
trans (50):cis (50') = 3.7:1
68.13
33.28
25.42
25.04
220 210 200 190 180 170 160 150 140 130 120 110 100 90 80 70 60 50 40 30 20 10 0 -10 -20
f1 (ppm)

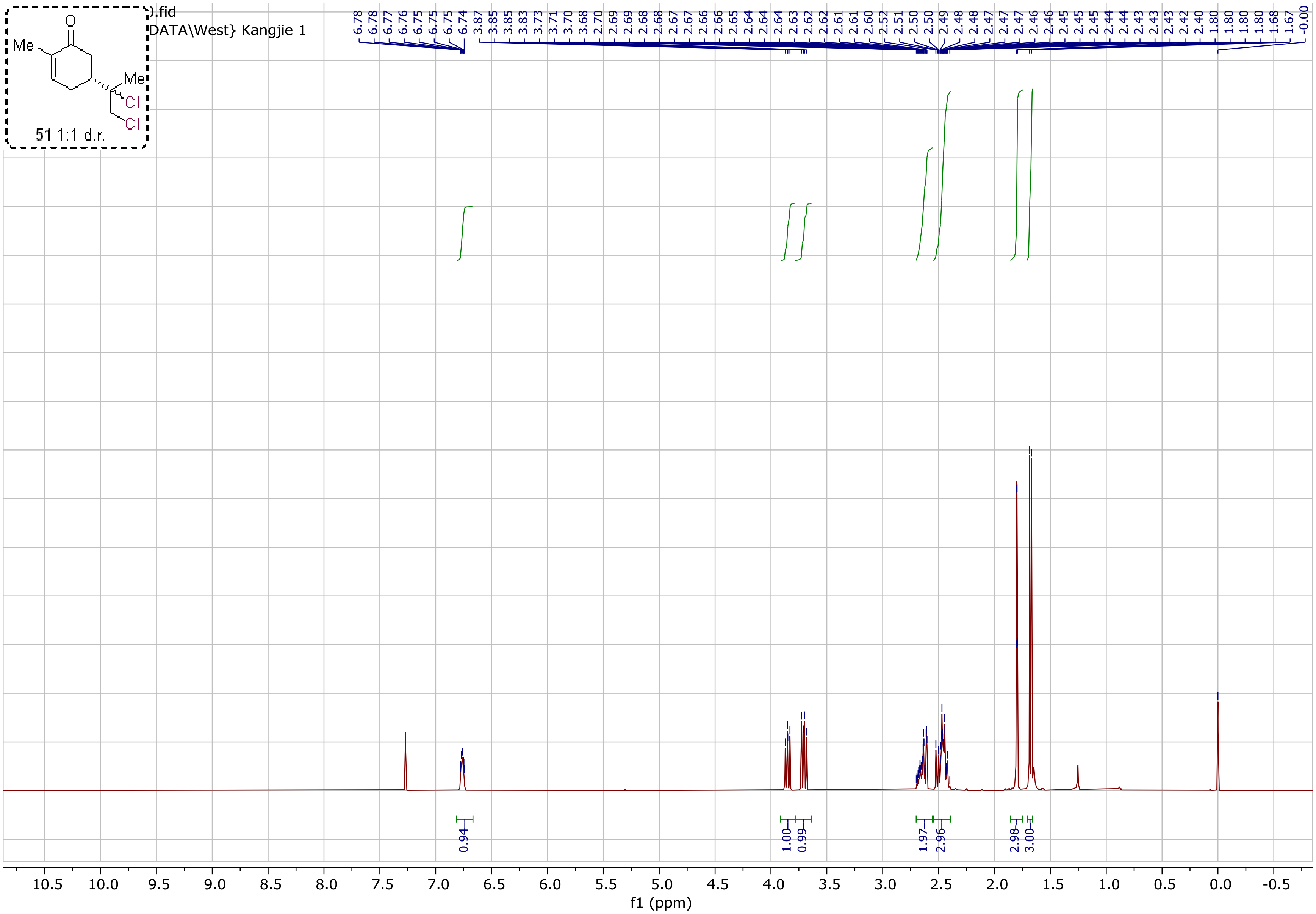

).fid
DATA\West} Kangjie 1
O
Me
Me
Cl
Cl
51 1:1 d.r.
6.78 6.78 6.77 6.76 6.75 6.75 6.75 6.74 3.87 3.85 3.85 3.83 3.73 3.71 3.70 3.68 2.70 2.69 2.69 2.68 2.68 2.67 2.67 2.66 2.66 2.65 2.64 2.64 2.64 2.63 2.62 2.62 2.61 2.61 2.60 2.52 2.51 2.50 2.50 2.49 2.48 2.47 2.47 2.47 2.46 2.46 2.45 2.45 2.44 2.44 2.43 2.43 2.43 2.42 2.40 1.80 1.80 1.80 1.80 1.68 1.67 -0.00
0.94
1.00
0.99
1.97
2.96
2.98
3.00
10.5 10.0 9.5 9.0 8.5 8.0 7.5 7.0 6.5 6.0 5.5 5.0 4.5 4.0 3.5 3.0 2.5 2.0 1.5 1.0 0.5 0.0 -0.5
f1 (ppm)

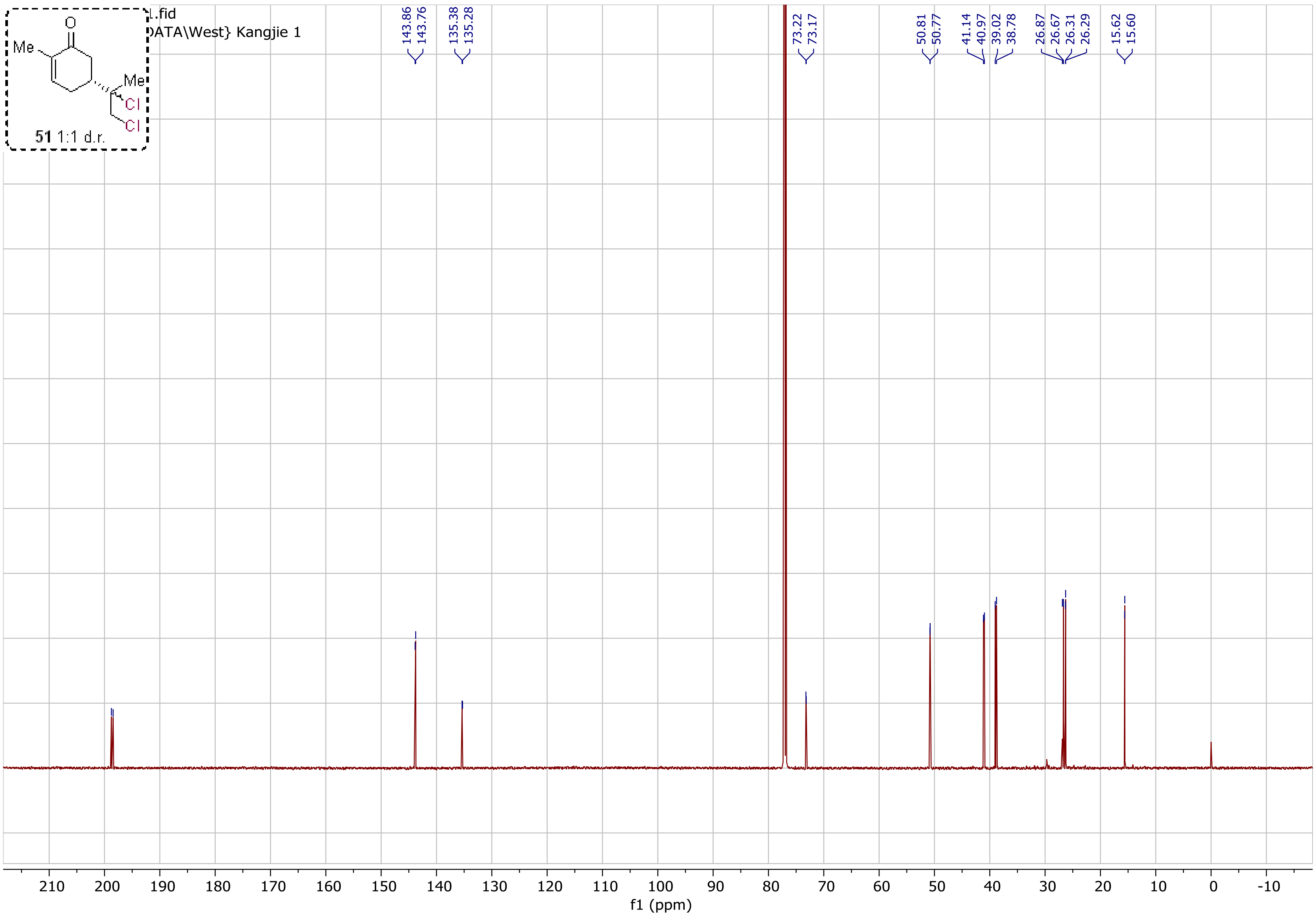

O
Me
Me
Cl
Cl
51 1:1 d.r.
1.fid
DATA\West} Kangjie 1
143.86
143.76
135.38
135.28
73.22
73.17
50.81
50.77
41.14
40.97
39.02
38.78
26.87
26.67
26.31
26.29
15.62
15.60
210
200
190
180
170
160
150
140
130
120
110
100
90
80
70
60
50
40
30
20
10
0
-10
f1 (ppm)

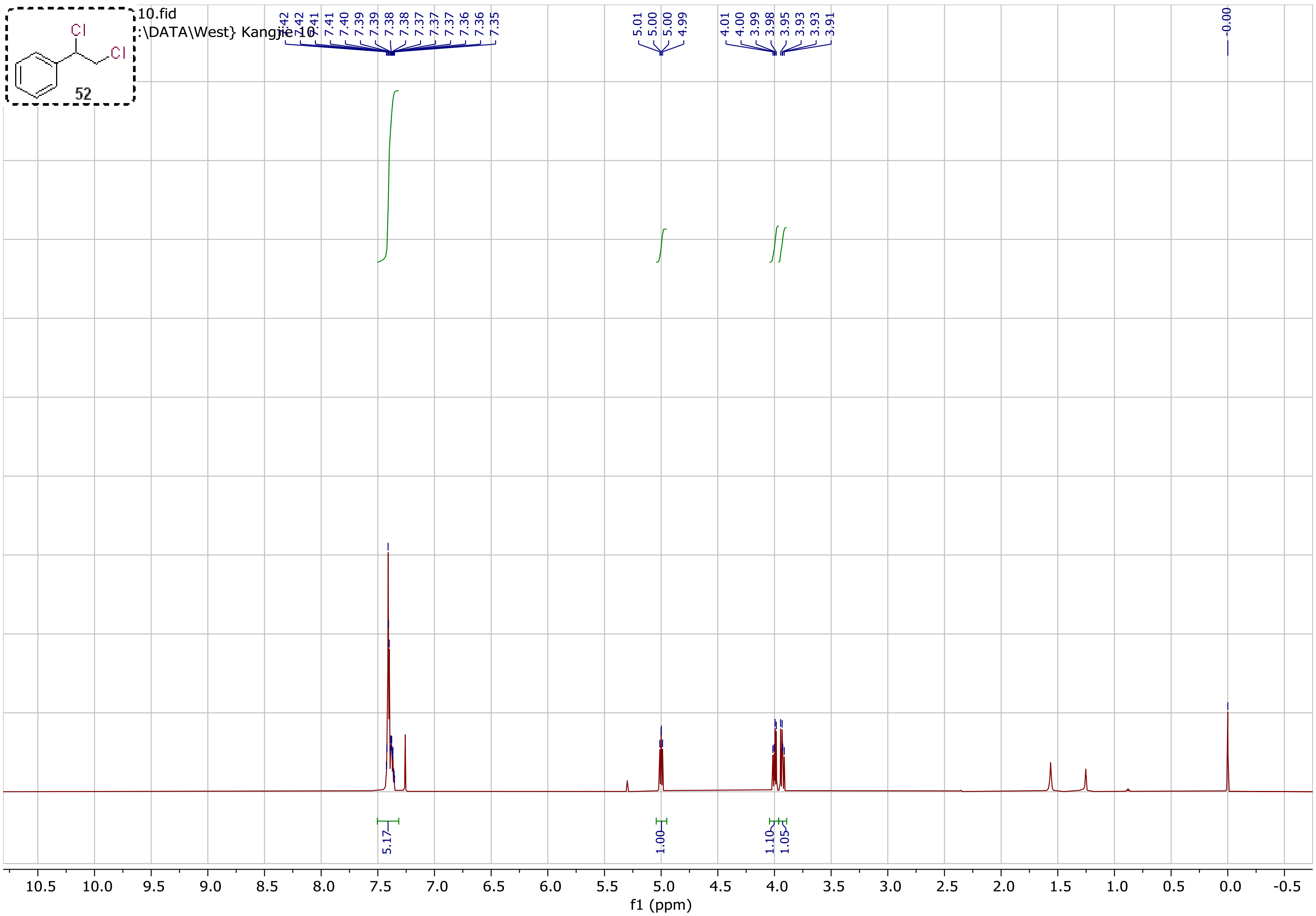

52
10.fid
:\DATA\West} Kangjie 10
7.42
7.42
7.41
7.41
7.40
7.39
7.39
7.38
7.38
7.37
7.37
7.37
7.36
7.36
7.35
5.01
5.00
5.00
4.99
4.01
4.00
3.99
3.98
3.95
3.93
3.93
3.91
-0.00
5.17
1.00
1.10
1.05
f1 (ppm)

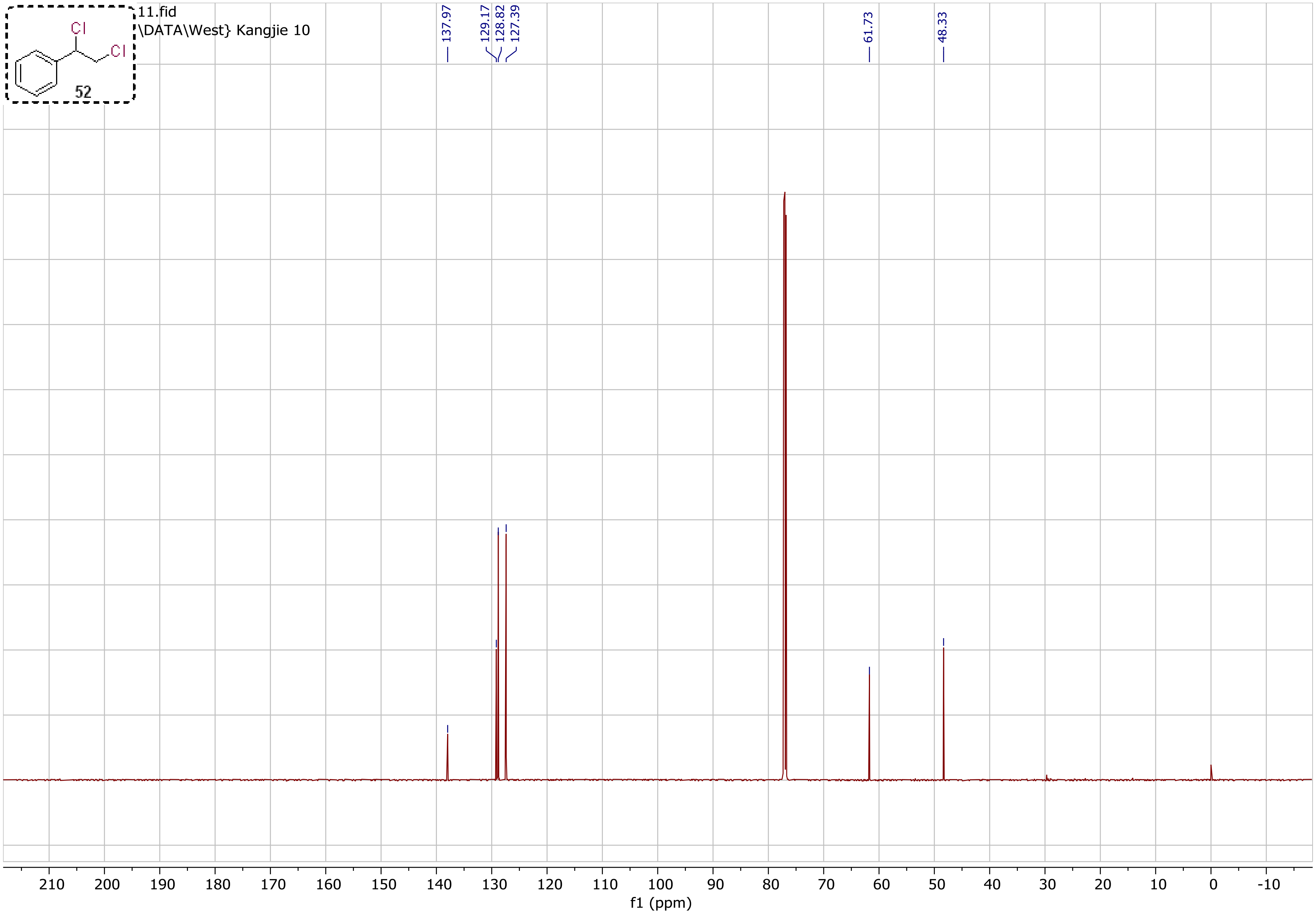

Cl
Cl
52
11.fid
\DATA\West} Kangjie 10
137.97
129.17
128.82
127.39
61.73
48.33
210
200
190
180
170
160
150
140
130
120
110
100
90
80
70
60
50
40
30
20
10
0
-10
f1 (ppm)

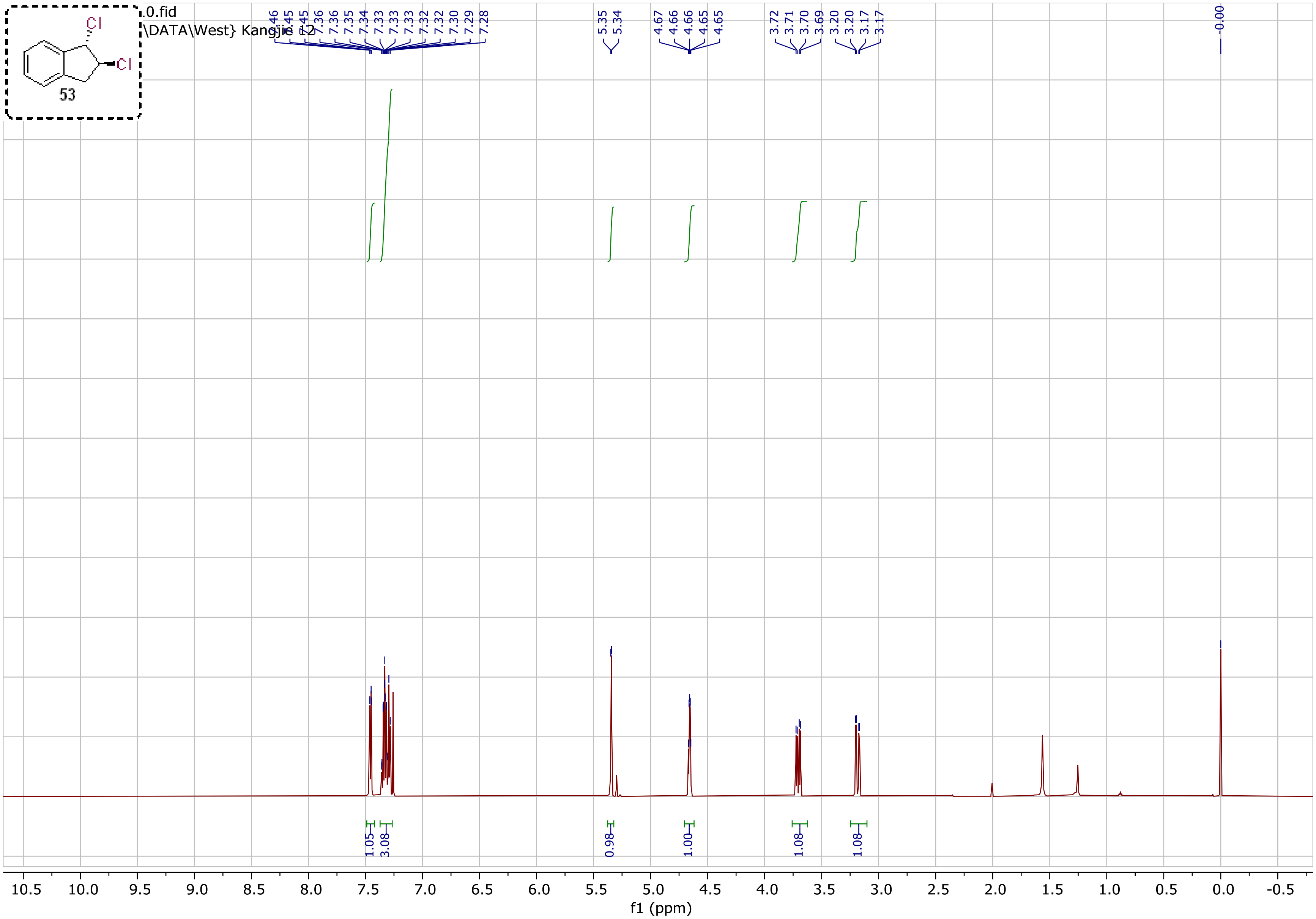
53
.0.fid
\DATA\West} Kangjie 12
7.46
7.45
7.45
7.36
7.36
7.35
7.34
7.33
7.33
7.33
7.32
7.32
7.30
7.29
7.28
5.35
5.34
4.67
4.66
4.66
4.65
4.65
3.72
3.71
3.70
3.69
3.20
3.20
3.17
3.17
-0.00
1.05
3.08
0.98
1.00
1.08
1.08
f1 (ppm)

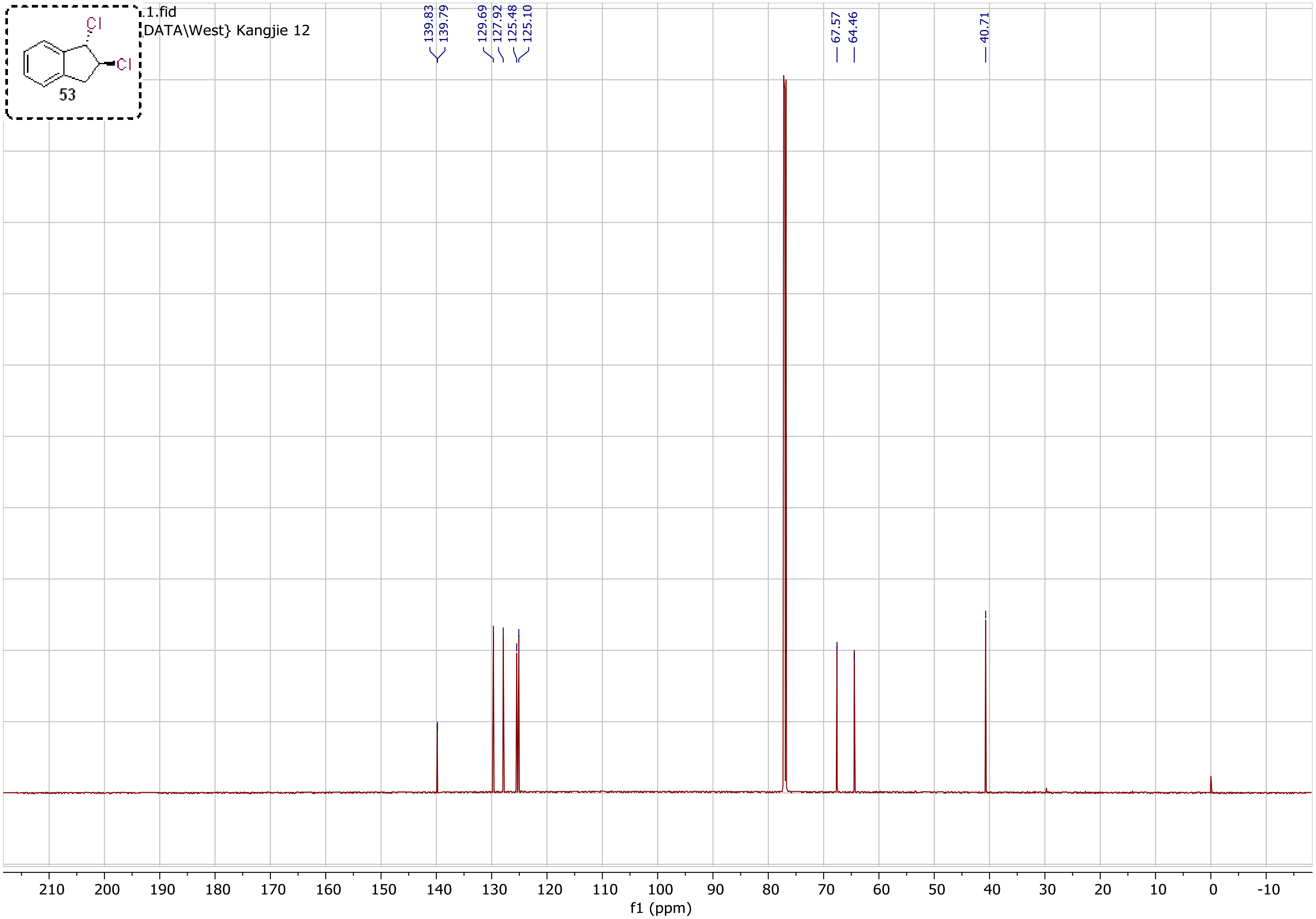
.1.fid
DATA\West} Kangjie 12
Cl
Cl
53
139.83
139.79
129.69
127.92
125.48
125.10
67.57
64.46
40.71
210 200 190 180 170 160 150 140 130 120 110 100 90 80 70 60 50 40 30 20 10 0 -10
f1 (ppm)

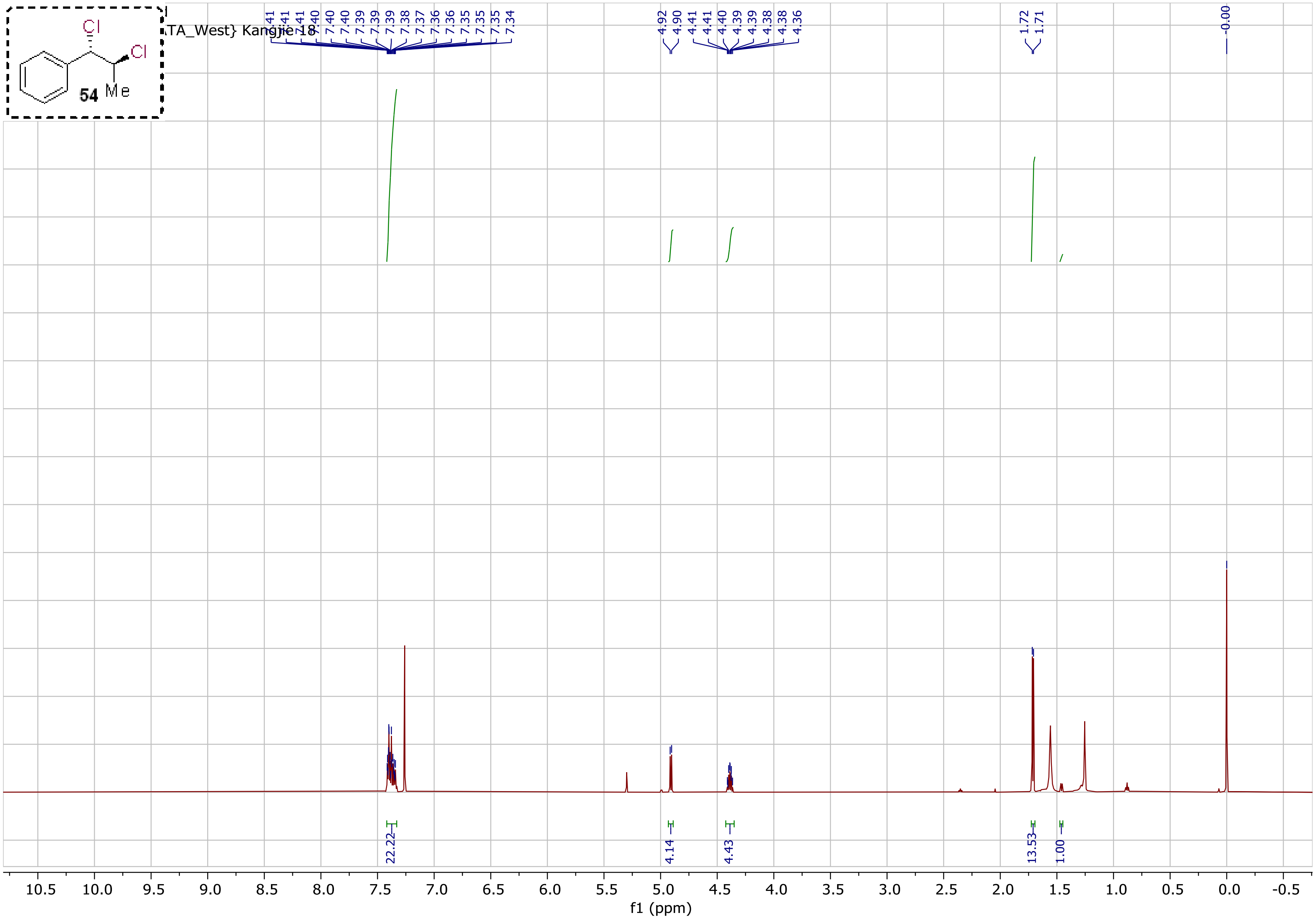

54
TA_West} Kangjie 18
f1 (ppm)

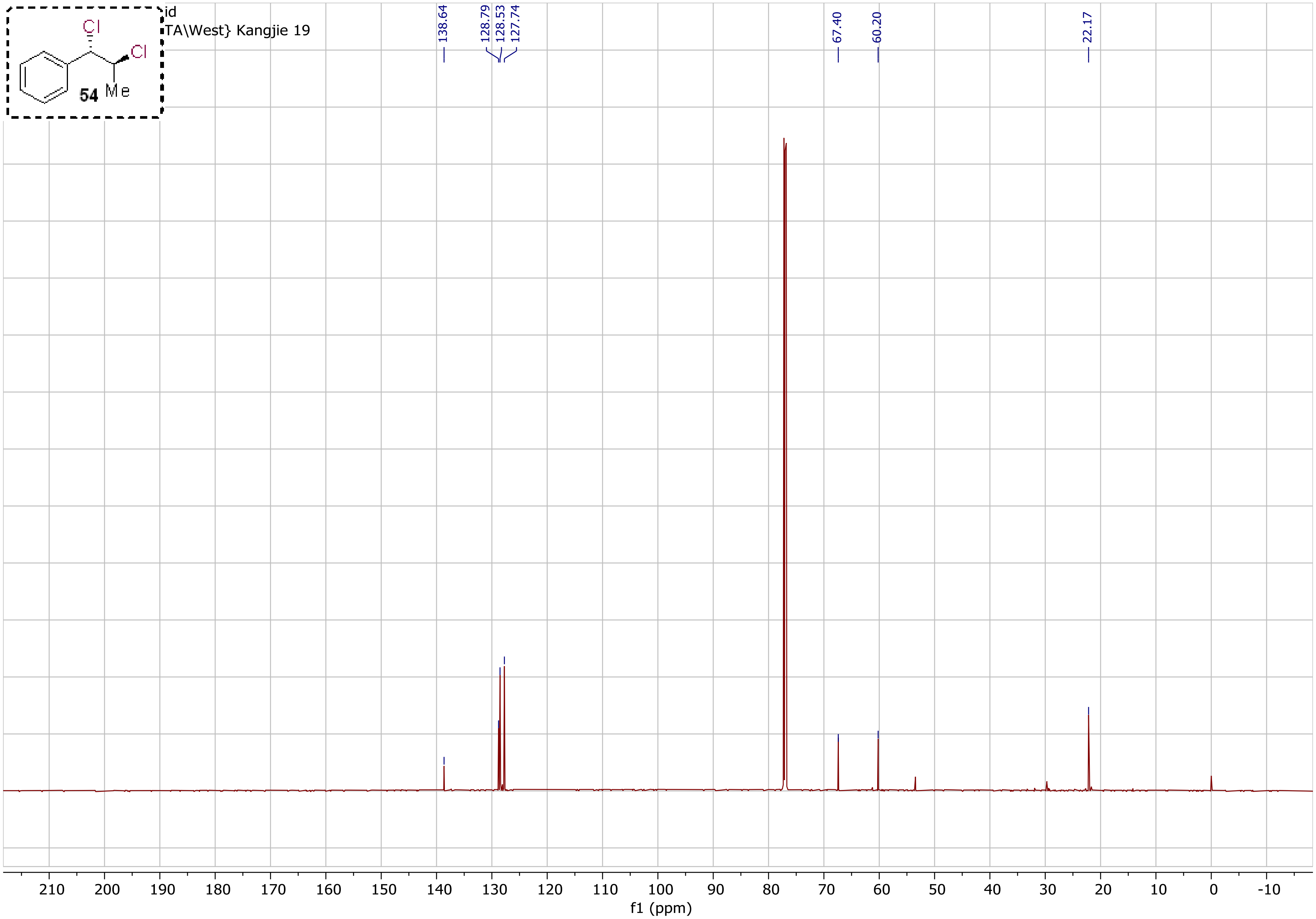
Cl
Cl
54
Me
id
TA\West} Kangjie 19
138.64
128.79
128.53
127.74
67.40
60.20
22.17
210
200
190
180
170
160
150
140
130
120
110
100
90
80
70
60
50
40
30
20
10
0
-10
f1 (ppm)

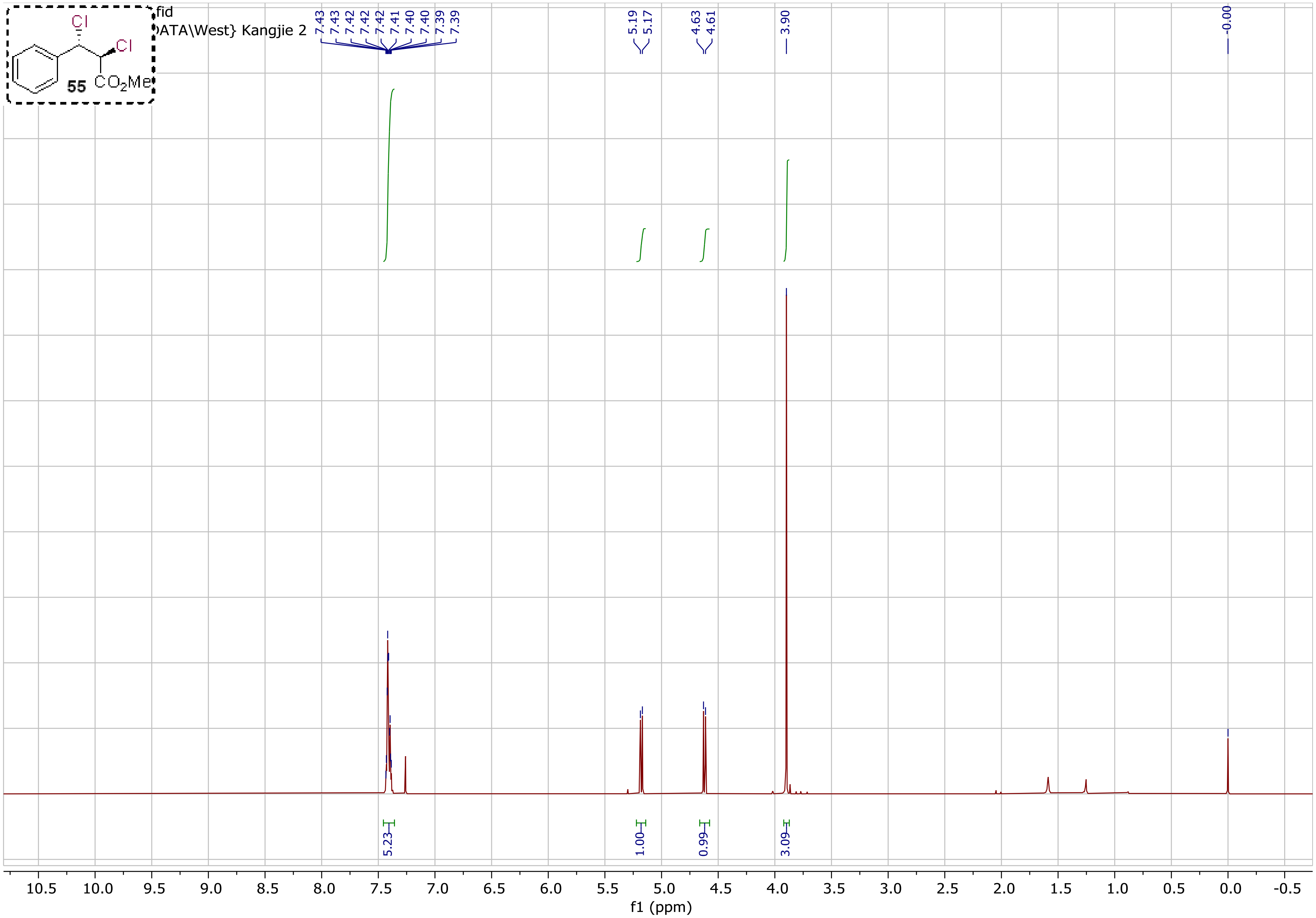
fid
)ATA\West} Kangjie 2
55
7.43 7.43 7.42 7.42 7.42 7.41 7.40 7.40 7.39 7.39
5.19 5.17
4.63 4.61
3.90
-0.00
5.23
1.00
0.99
3.09
f1 (ppm)

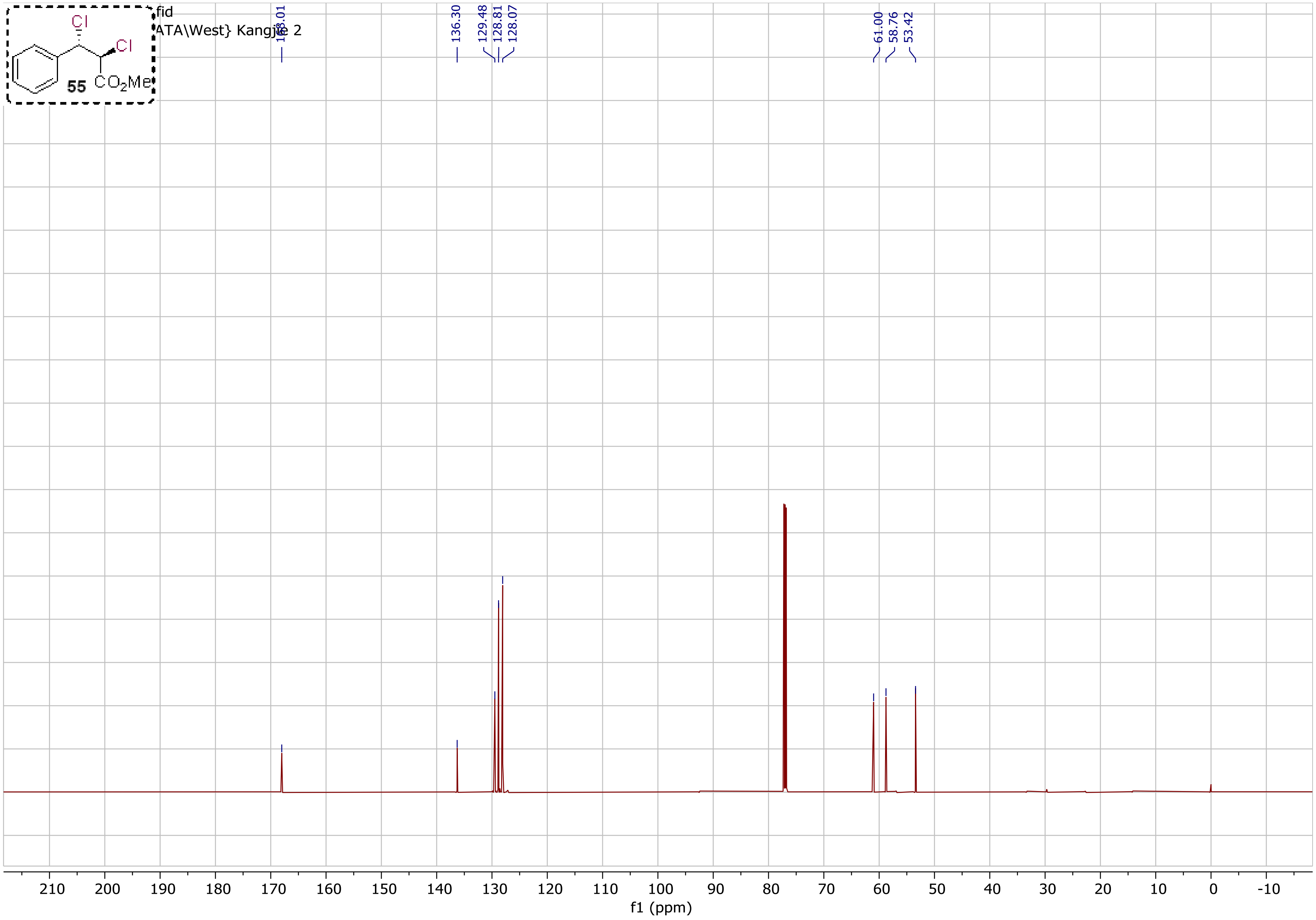

fid
ATA\West} Kangjie 2
Cl
Cl
55
CO2Me
168.01
136.30
129.48
128.81
128.07
61.00
58.76
53.42
210 200 190 180 170 160 150 140 130 120 110 100 90 80 70 60 50 40 30 20 10 0 -10
f1 (ppm)

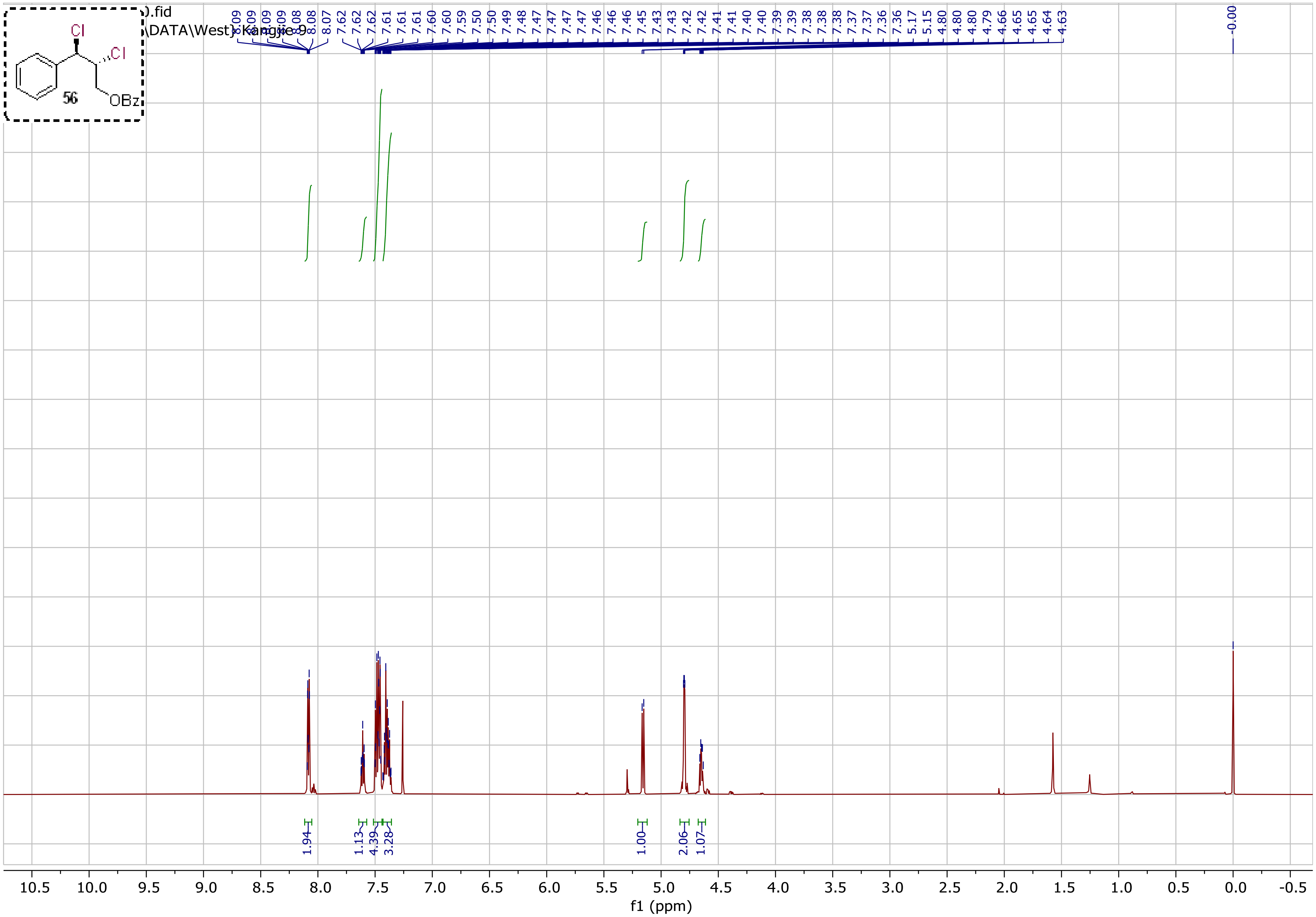
Cl
Cl
56
OBz
).fid
\DATA\West\Kangjie 9
8.09
8.09
8.09
8.09
8.08
8.08
8.07
7.62
7.62
7.62
7.61
7.61
7.61
7.60
7.59
7.50
7.50
7.49
7.48
7.47
7.47
7.47
7.46
7.46
7.46
7.45
7.43
7.43
7.42
7.42
7.41
7.41
7.40
7.40
7.39
7.39
7.38
7.38
7.38
7.37
7.37
7.36
7.36
5.17
5.15
4.80
4.80
4.79
4.66
4.65
4.65
4.64
4.63
-0.00
1.94
1.13
4.39
3.28
1.00
2.06
1.07
10.5
10.0
9.5
9.0
8.5
8.0
7.5
7.0
6.5
6.0
5.5
5.0
4.5
4.0
3.5
3.0
2.5
2.0
1.5
1.0
0.5
0.0
-0.5
f1 (ppm)

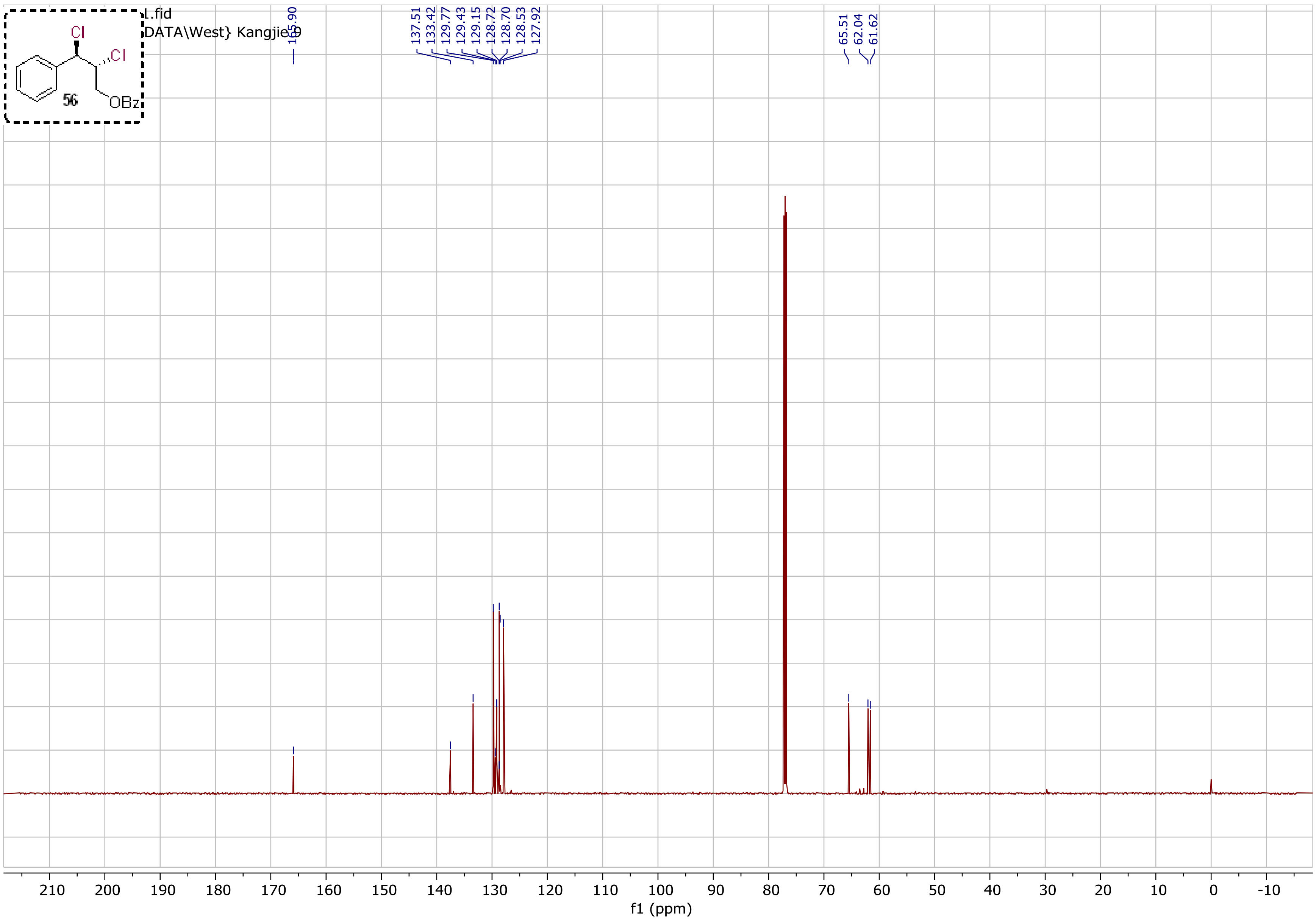

1.fid
DATA\West} Kangjie-9
165.90
137.51
133.42
129.77
129.43
129.15
128.72
128.70
128.53
127.92
65.51
62.04
61.62
Cl
Cl
56
OBz
f1 (ppm)

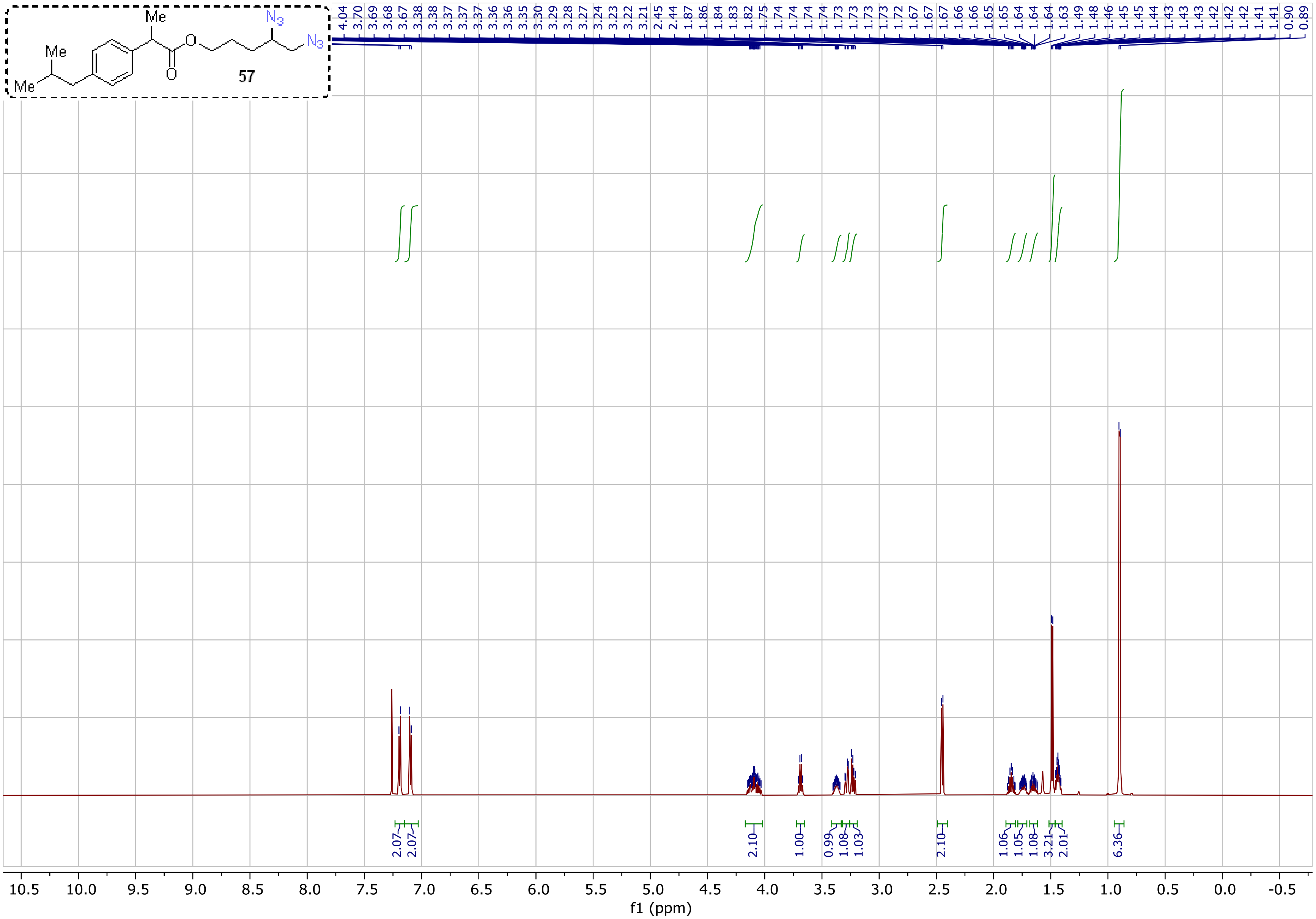

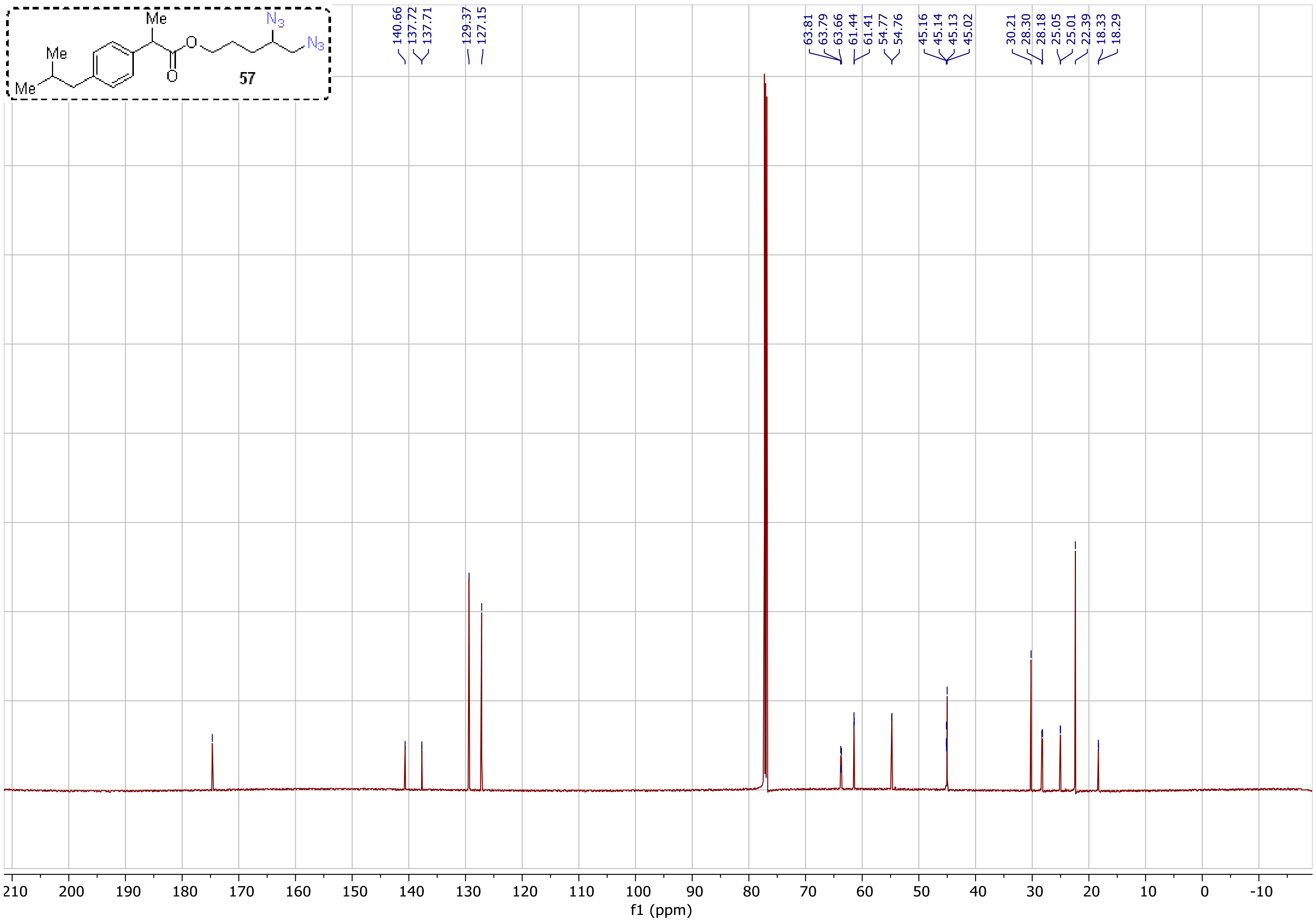

57
140.66
137.72
137.71
129.37
127.15
63.81
63.79
63.66
61.44
61.41
54.77
54.76
45.16
45.14
45.13
45.02
30.21
28.30
28.18
25.05
25.01
22.39
18.33
18.29
f1 (ppm)

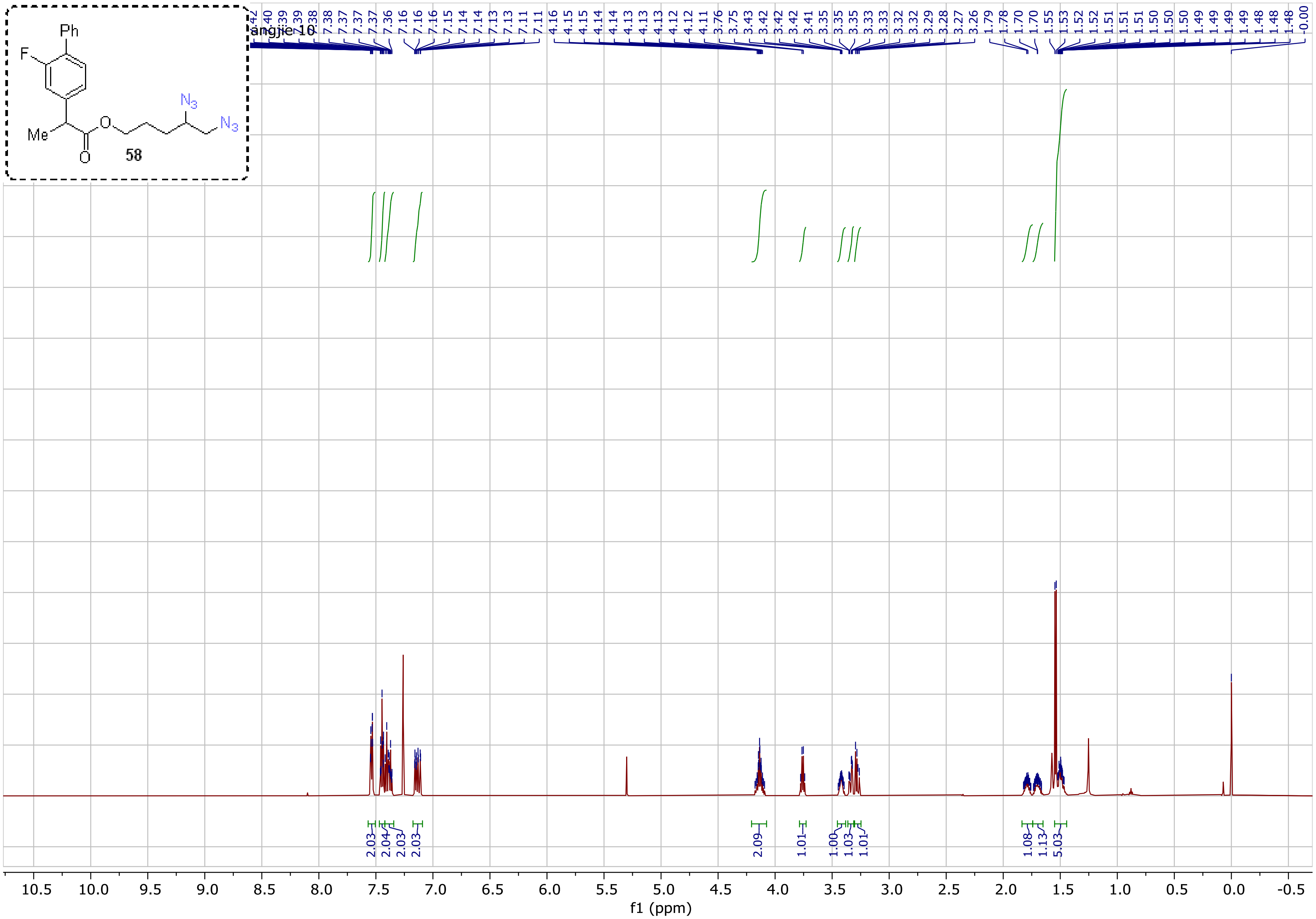

Ph
F
N3
N3
Me
O
O
58
f1 (ppm)

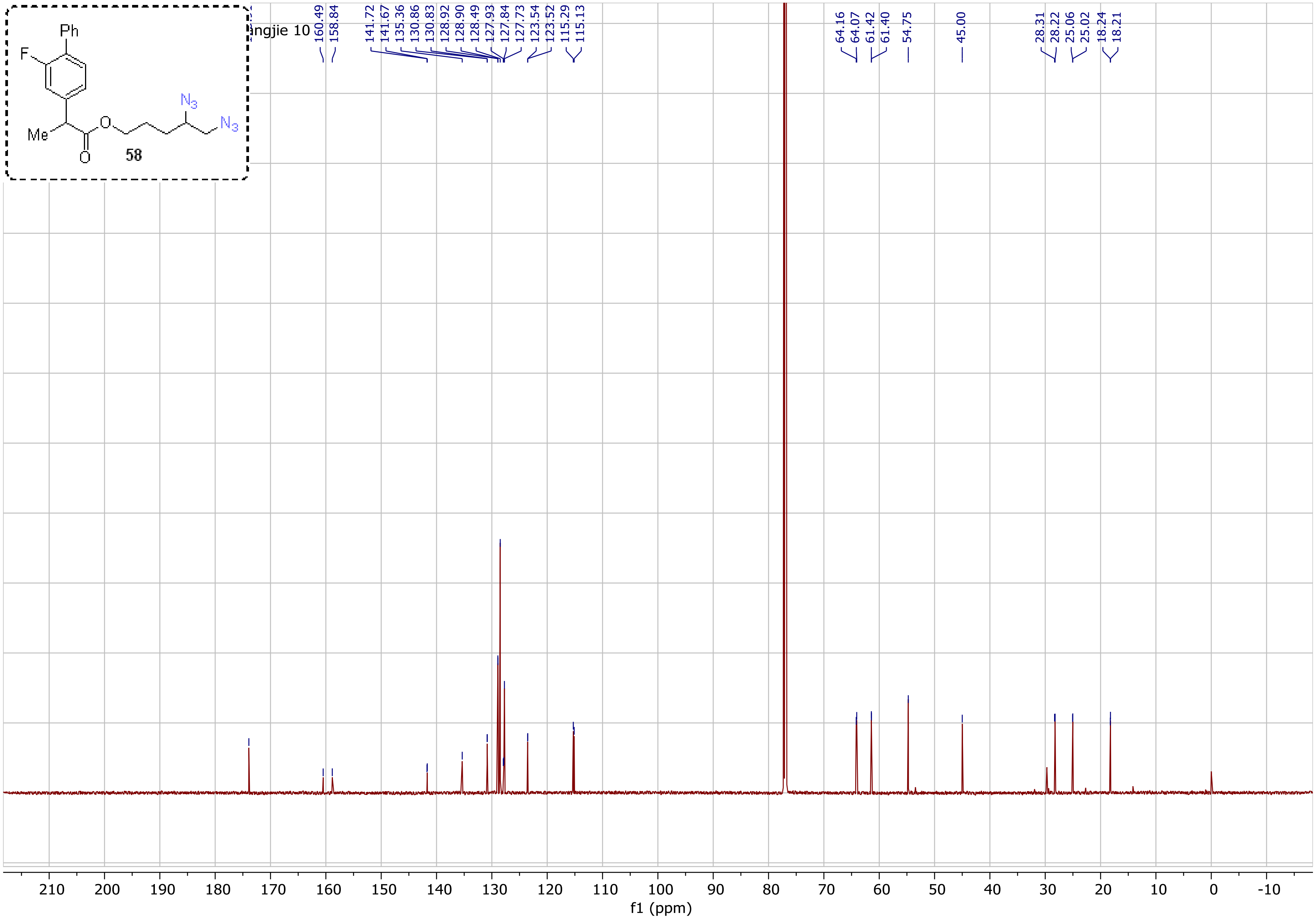

Ph
F
N3
N3
Me
O
O
58
ingjie 10
160.49
158.84
141.72
141.67
135.36
130.86
130.83
128.92
128.90
128.49
127.93
127.84
127.73
123.54
123.52
115.29
115.13
64.16
64.07
61.42
61.40
54.75
45.00
28.31
28.22
25.06
25.02
18.24
18.21
210
200
190
180
170
160
150
140
130
120
110
100
90
80
70
60
50
40
30
20
10
0
-10
f1 (ppm)

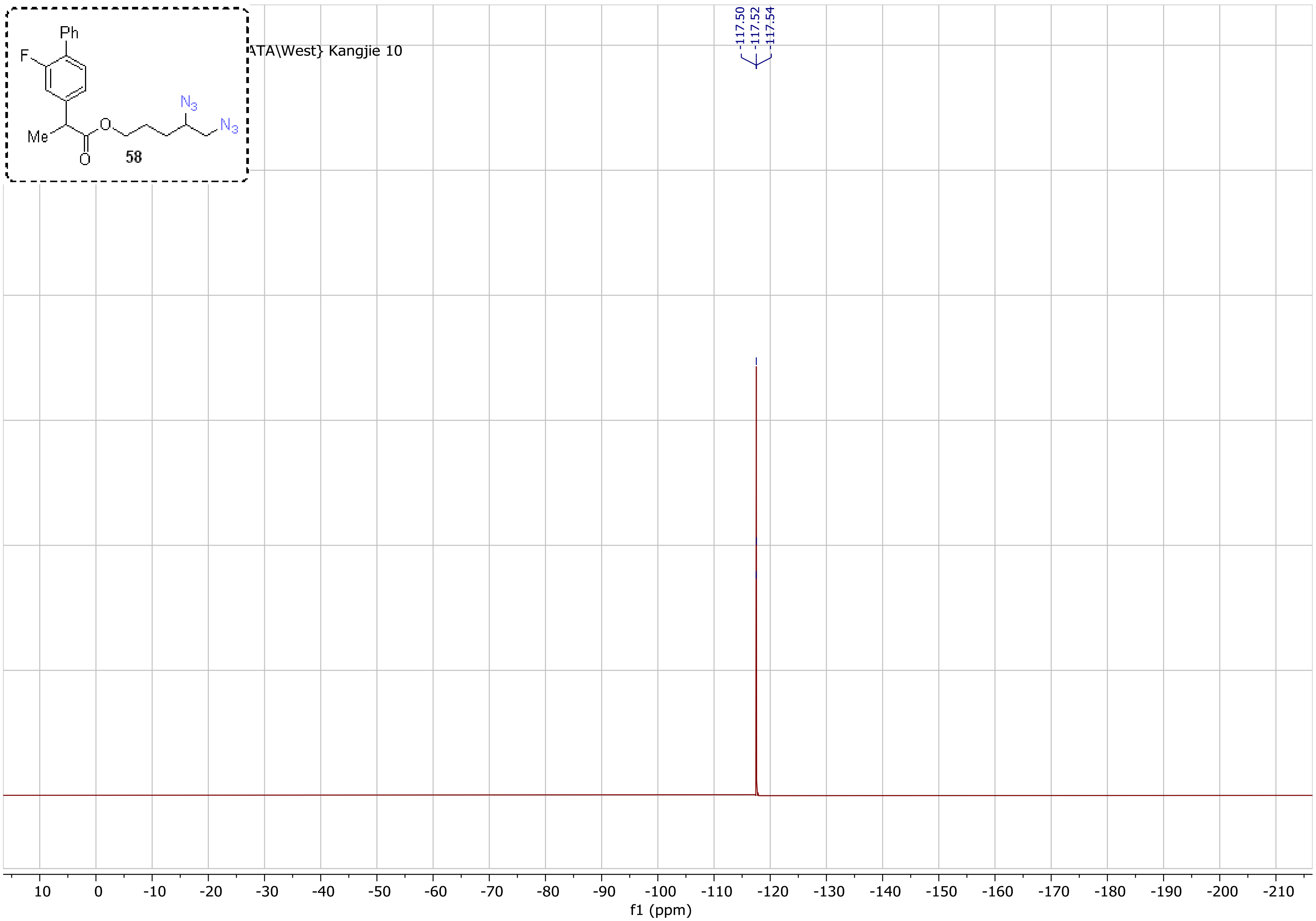
ATA\West} Kangjie 10
-117.50
-117.52
-117.54
Ph
F
Me
O
O
N3
N3
58
10
0
-10
-20
-30
-40
-50
-60
-70
-80
-90
-100
-110
-120
-130
-140
-150
-160
-170
-180
-190
-200
-210
f1 (ppm)

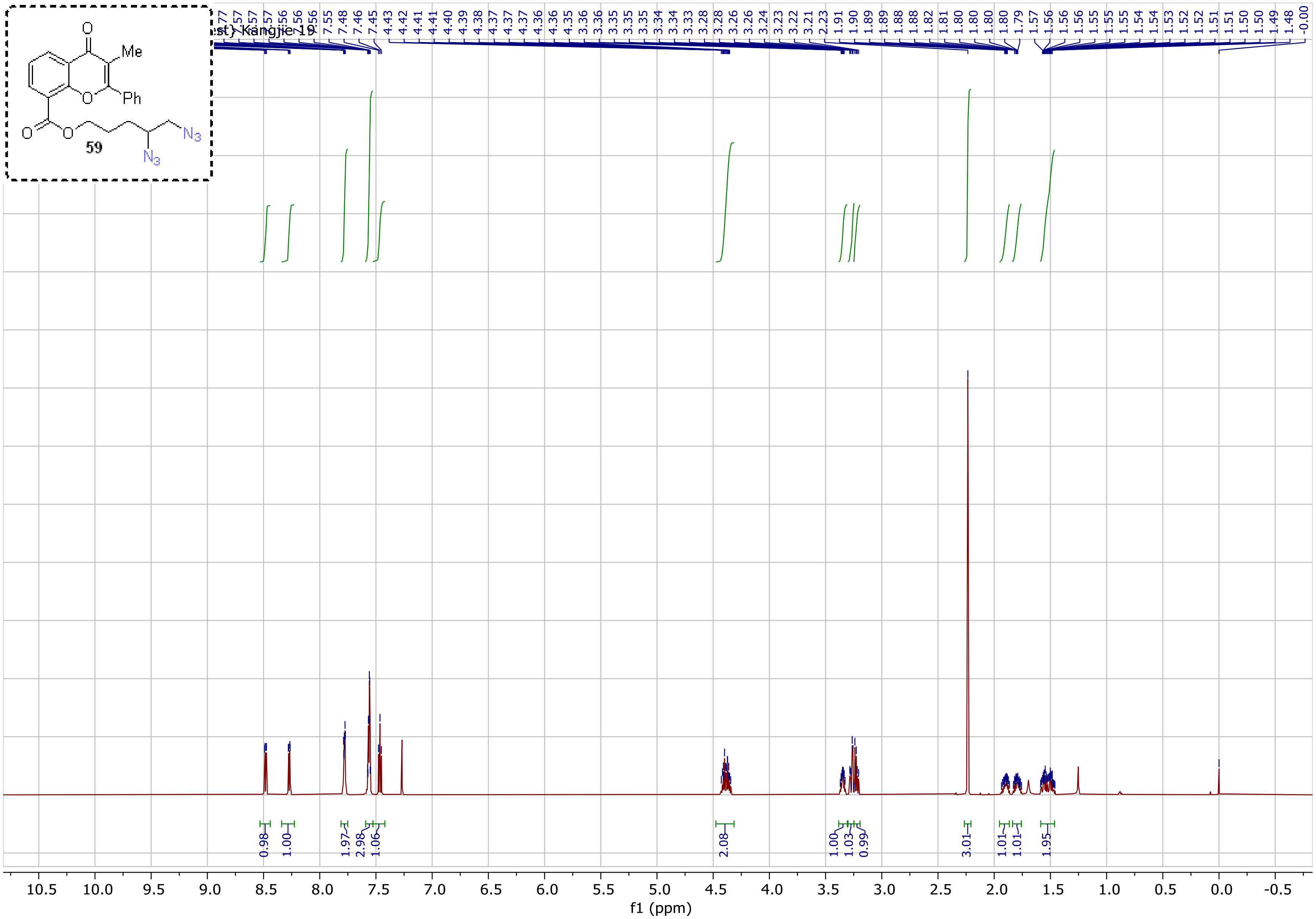
59
Me
Ph
O
N3
f1 (ppm)

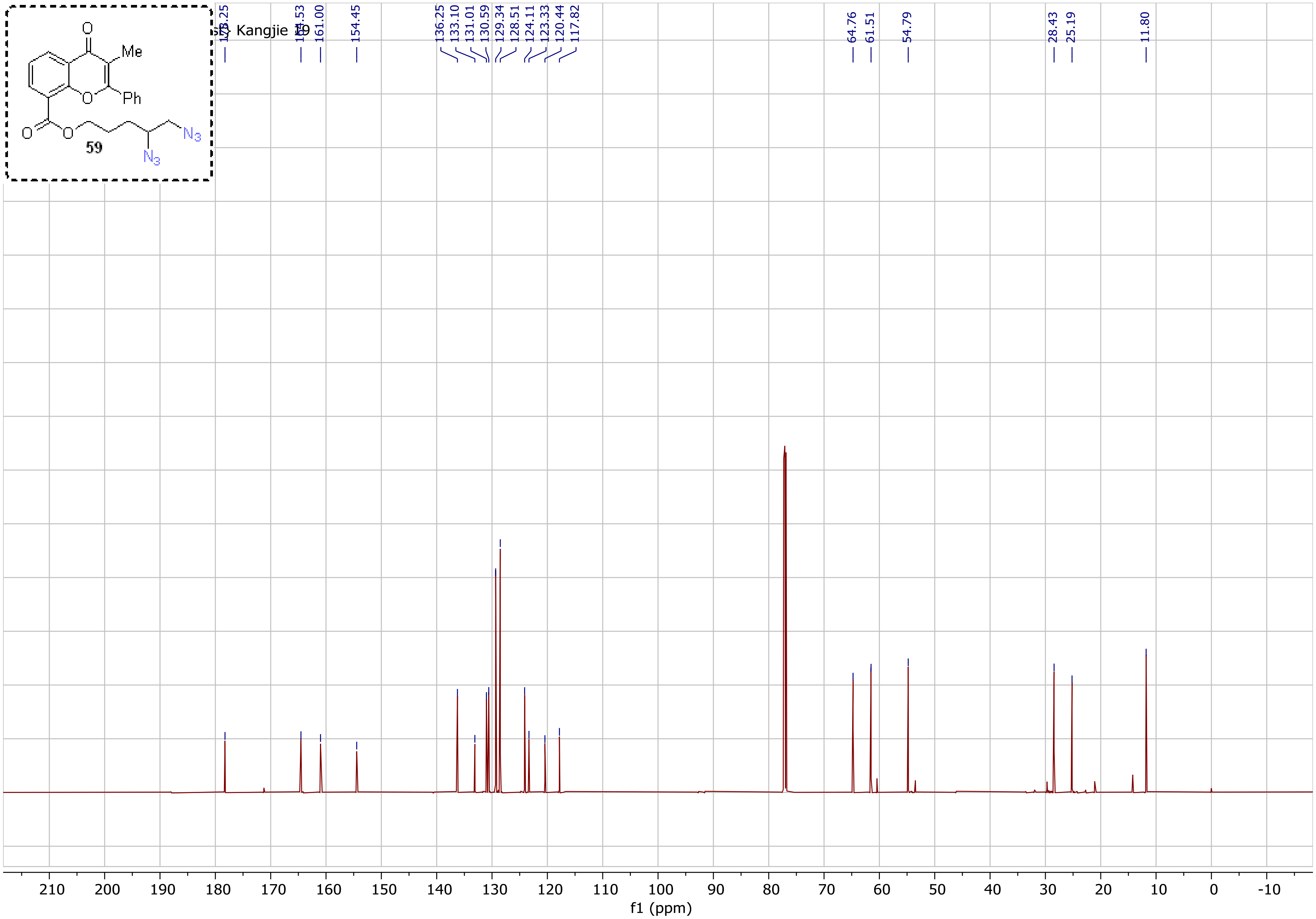

Me
Ph
N3
59
Kangjie
161.00
154.45
136.25
133.10
131.01
130.59
129.34
128.51
124.11
123.33
120.44
117.82
64.76
61.51
54.79
28.43
25.19
11.80
f1 (ppm)

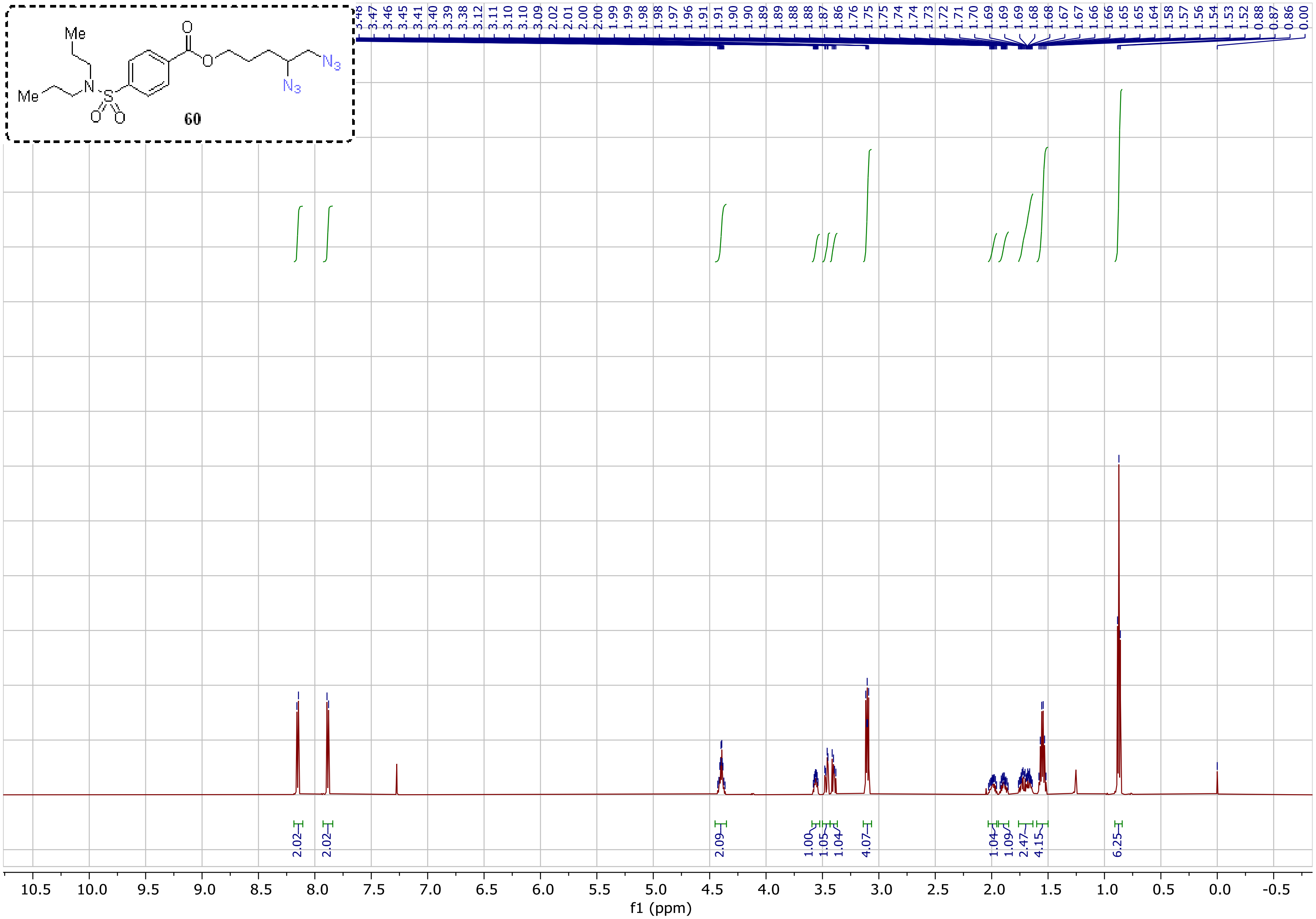
Me
Me
N
S
O
O
O
O
N3
N3
60
3.47
3.46
3.45
3.41
3.40
3.39
3.38
3.12
3.11
3.10
3.10
3.09
2.02
2.01
2.00
2.00
1.99
1.99
1.98
1.98
1.97
1.96
1.91
1.91
1.90
1.90
1.89
1.88
1.88
1.88
1.87
1.86
1.76
1.75
1.75
1.74
1.74
1.73
1.72
1.71
1.70
1.69
1.69
1.69
1.68
1.68
1.67
1.67
1.66
1.66
1.65
1.65
1.64
1.58
1.57
1.56
1.54
1.53
1.52
0.88
0.87
0.86
0.00
2.02
2.02
2.09
1.00
1.05
1.04
4.07
1.04
1.09
2.47
4.15
6.25
10.5
10.0
9.5
9.0
8.5
8.0
7.5
7.0
6.5
6.0
5.5
5.0
4.5
4.0
3.5
3.0
2.5
2.0
1.5
1.0
0.5
0.0
-0.5
f1 (ppm)

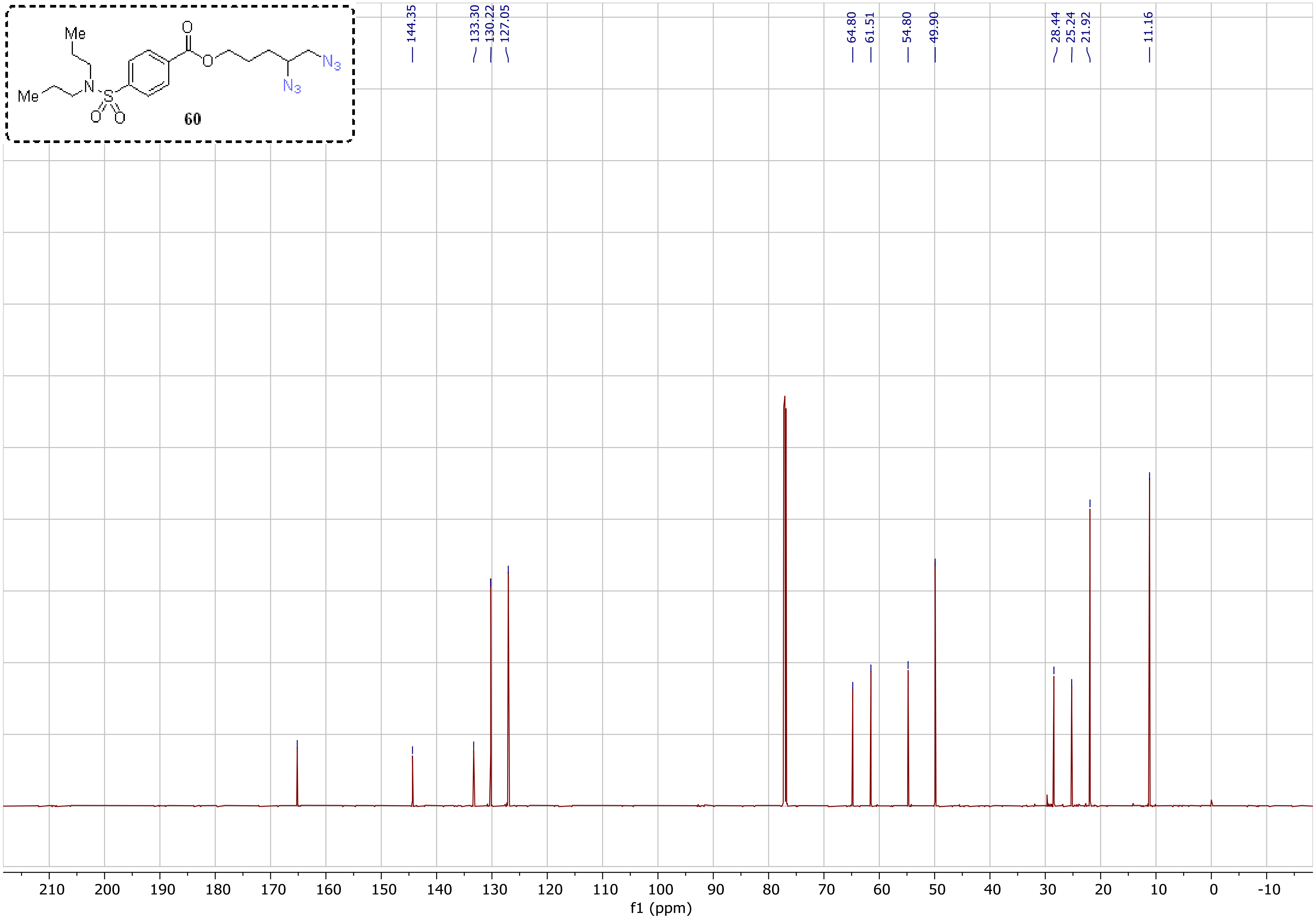

Me
O
O
N3
Me
N
S
O
O
N3
60
144.35
133.30
130.22
127.05
64.80
61.51
54.80
49.90
28.44
25.24
21.92
11.16
210
200
190
180
170
160
150
140
130
120
110
100
90
80
70
60
50
40
30
20
10
0
-10
f1 (ppm)

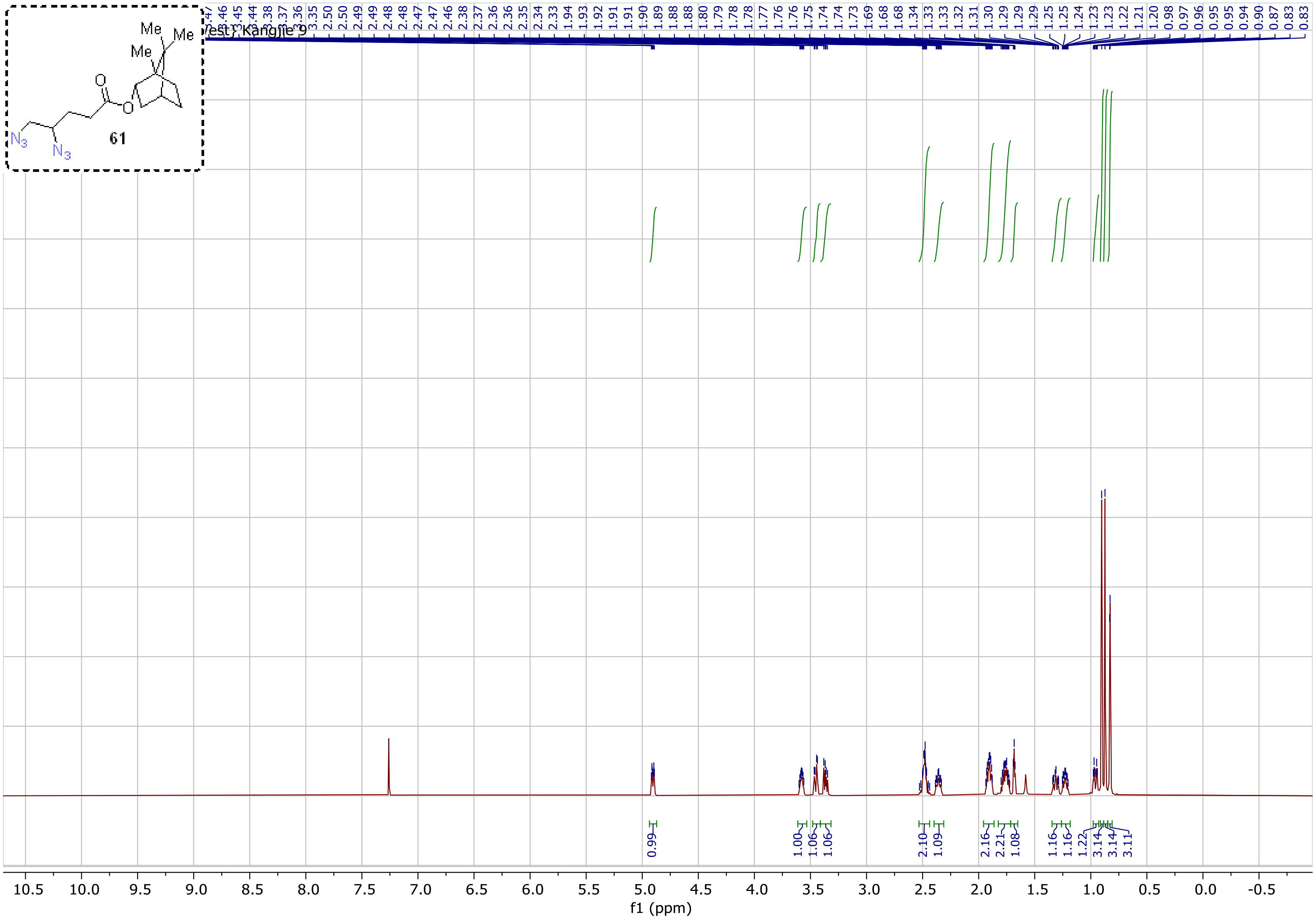

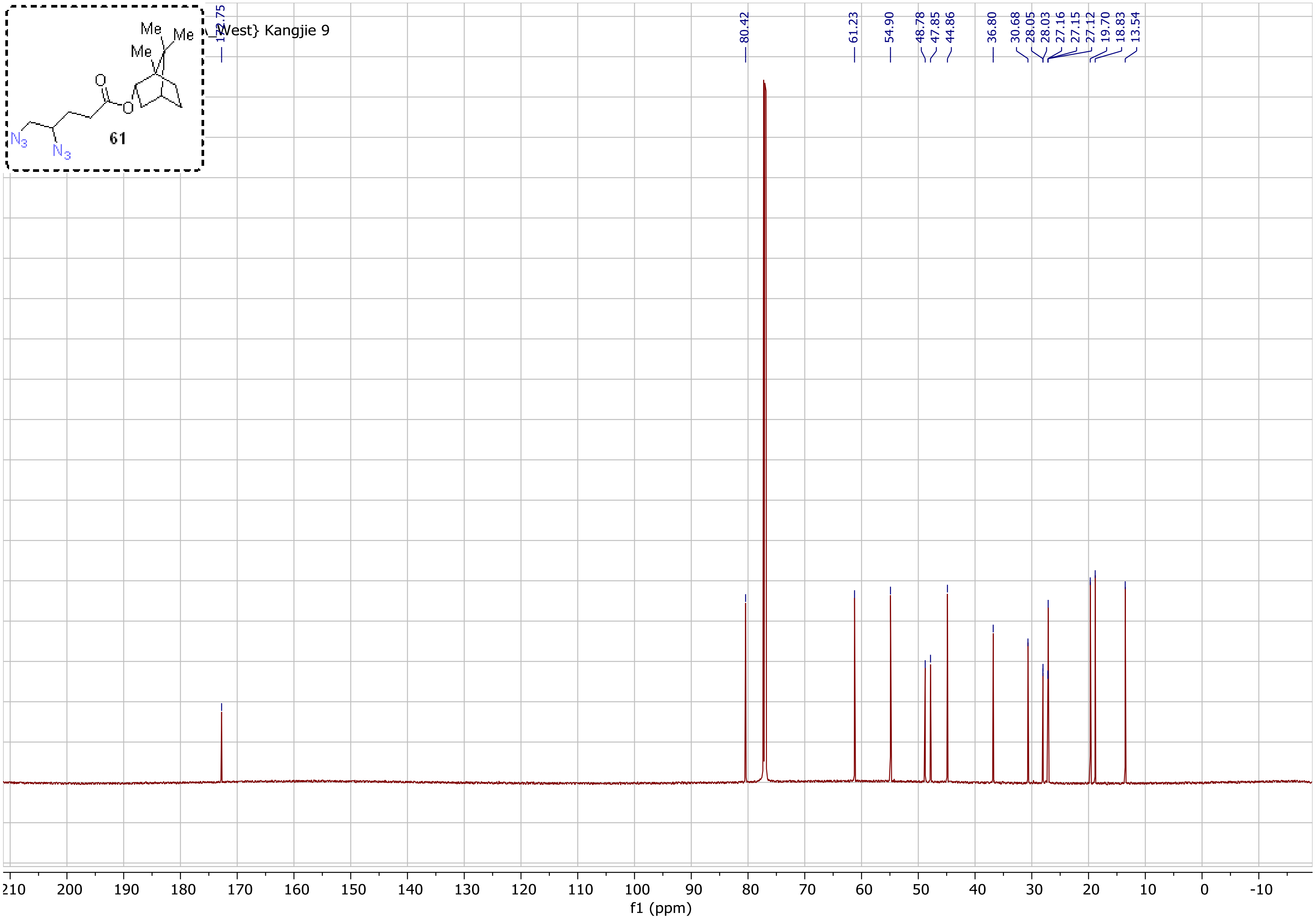
West} Kangjie 9
Me
Me
Me
O
O
N3
N3
61
80.42
61.23
54.90
48.78
47.85
44.86
36.80
30.68
28.05
28.03
27.16
27.15
27.12
19.70
18.83
13.54
210 200 190 180 170 160 150 140 130 120 110 100 90 80 70 60 50 40 30 20 10 0 -10
f1 (ppm)

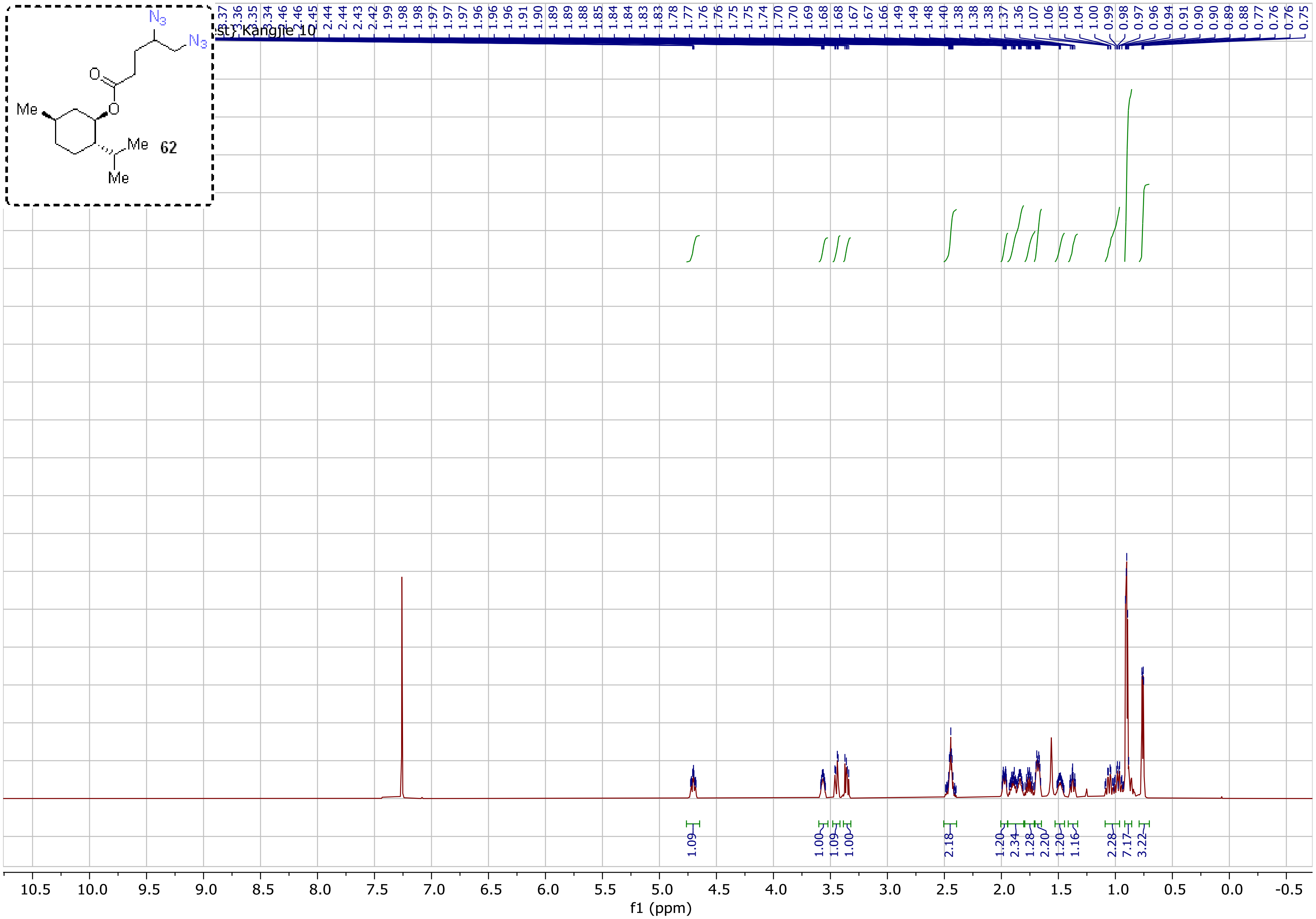
N3
N3
O
O
Me
Me
Me
62
st} Kangjie 10
3.37 3.36 3.35 3.34 2.46 2.45 2.44 2.44 2.43 2.42 1.99 1.98 1.98 1.97 1.97 1.97 1.96 1.96 1.91 1.90 1.89 1.89 1.88 1.85 1.84 1.84 1.83 1.83 1.78 1.77 1.76 1.76 1.75 1.75 1.74 1.70 1.70 1.69 1.68 1.68 1.67 1.67 1.66 1.49 1.49 1.48 1.40 1.38 1.38 1.38 1.37 1.36 1.07 1.06 1.05 1.04 1.00 0.99 0.98 0.97 0.96 0.94 0.91 0.90 0.90 0.89 0.88 0.77 0.76 0.75
1.09
1.00 1.09 1.00
2.18
1.20 2.34 1.28 2.20 1.20 1.16
2.28 7.17 3.22
10.5 10.0 9.5 9.0 8.5 8.0 7.5 7.0 6.5 6.0 5.5 5.0 4.5 4.0 3.5 3.0 2.5 2.0 1.5 1.0 0.5 0.0 -0.5
f1 (ppm)

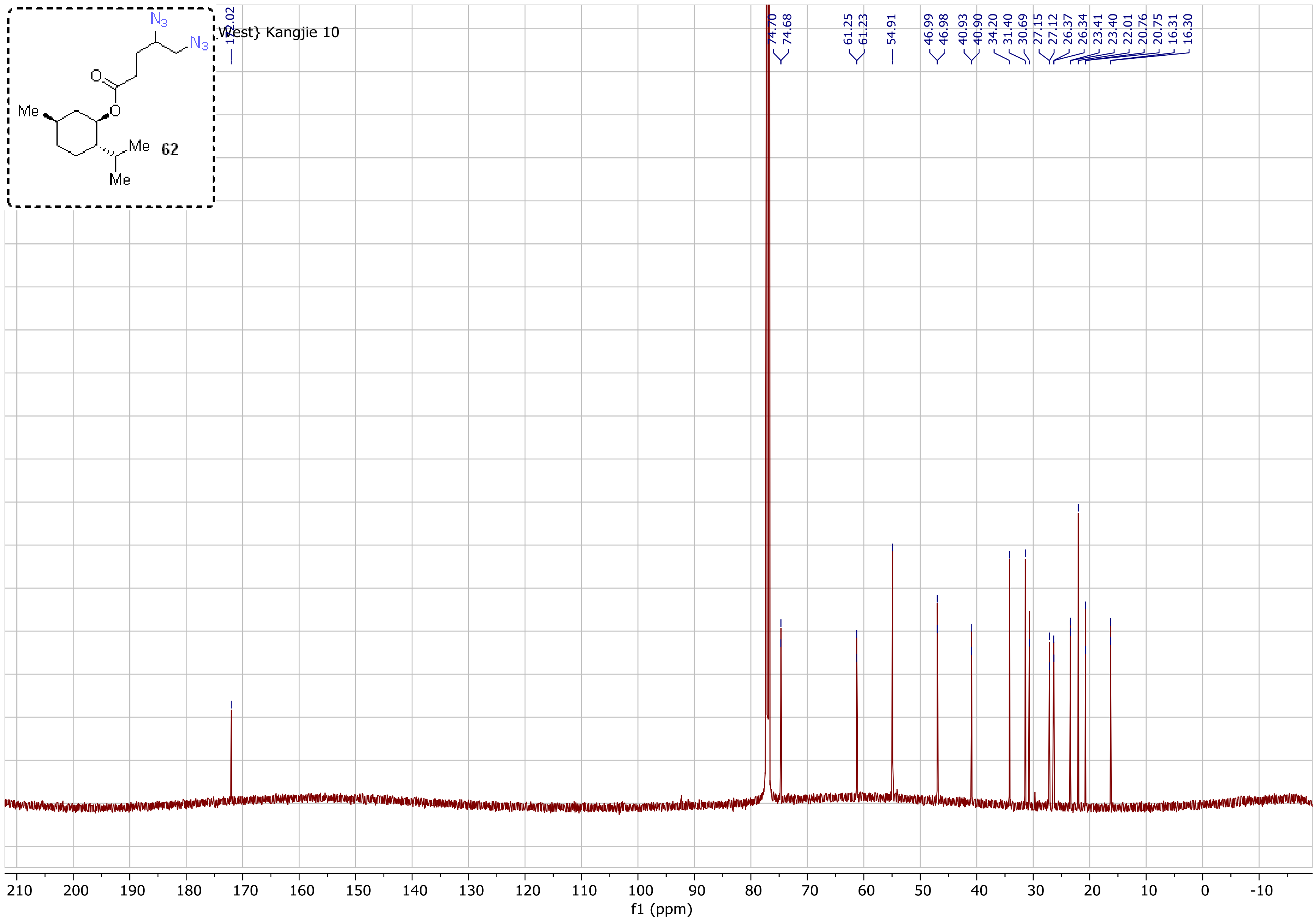
West} Kangjie 10
62
172.02
74.70
74.68
61.25
61.23
54.91
46.99
46.98
40.93
40.90
34.20
31.40
30.69
27.15
27.12
26.37
26.34
23.41
23.40
22.01
20.76
20.75
16.31
16.30
f1 (ppm)

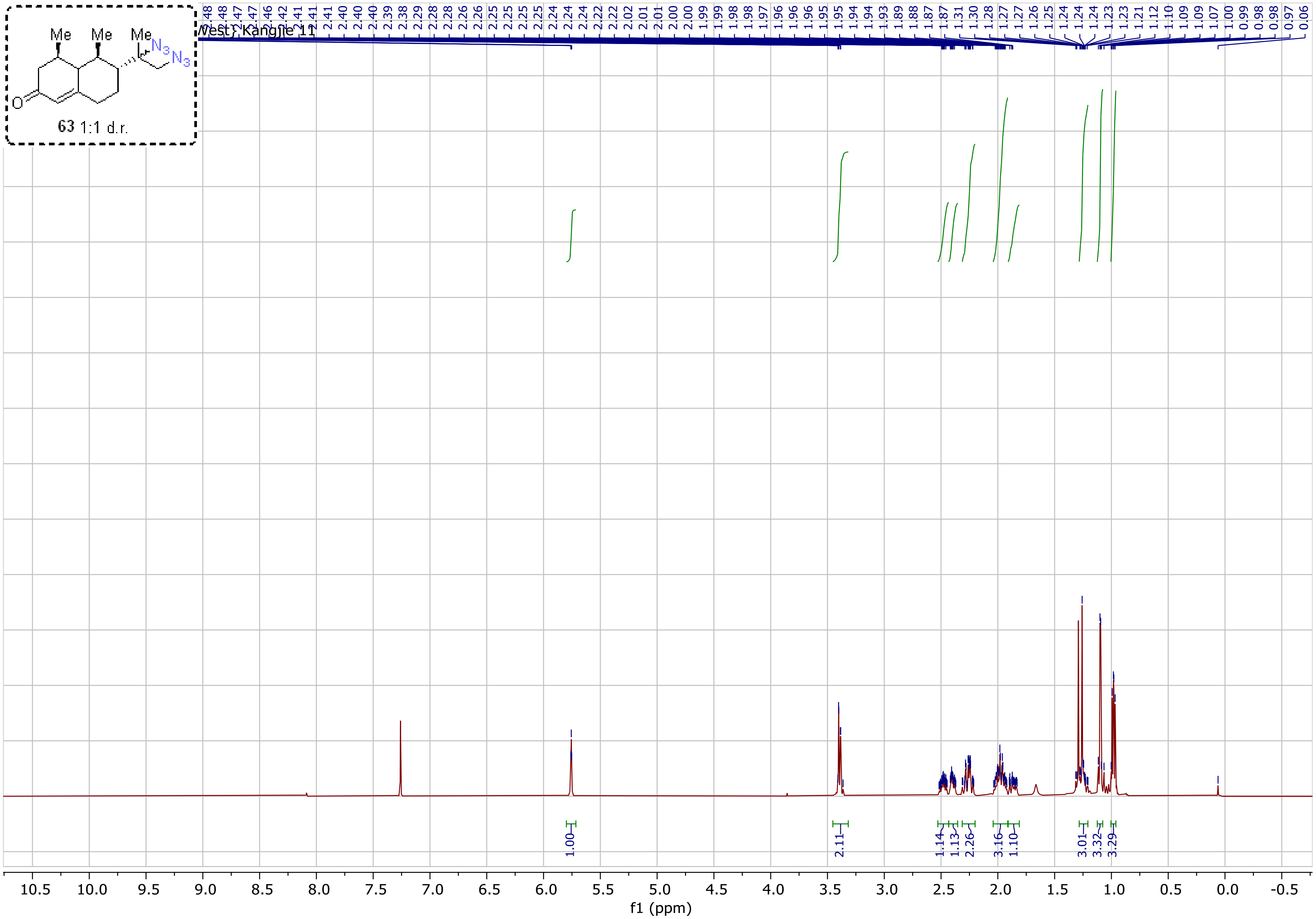

Me
Me
Me
N3
O
63 1:1 d.r.
1.00
2.11
1.14
1.13
2.26
3.16
1.10
3.01
3.32
3.29
10.5
10.0
9.5
9.0
8.5
8.0
7.5
7.0
6.5
6.0
5.5
5.0
4.5
4.0
3.5
3.0
2.5
2.0
1.5
1.0
0.5
0.0
-0.5
f1 (ppm)

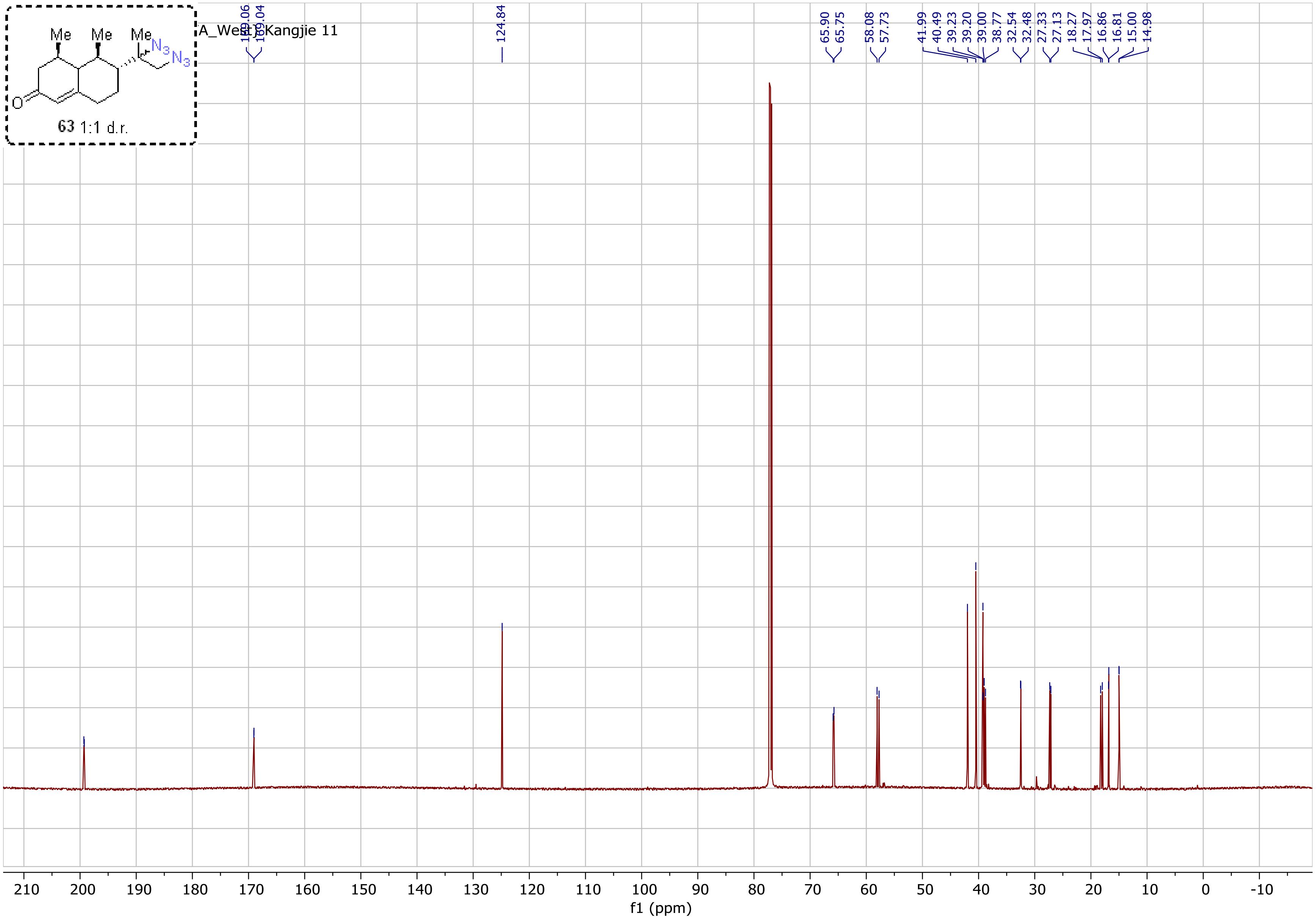
A_West_Kangjie 11
63 1:1 d.r.
169.06
169.04
124.84
65.90
65.75
58.08
57.73
41.99
40.49
39.23
39.20
39.00
38.77
32.54
32.48
27.33
27.13
18.27
17.97
16.86
16.81
15.00
14.98
f1 (ppm)

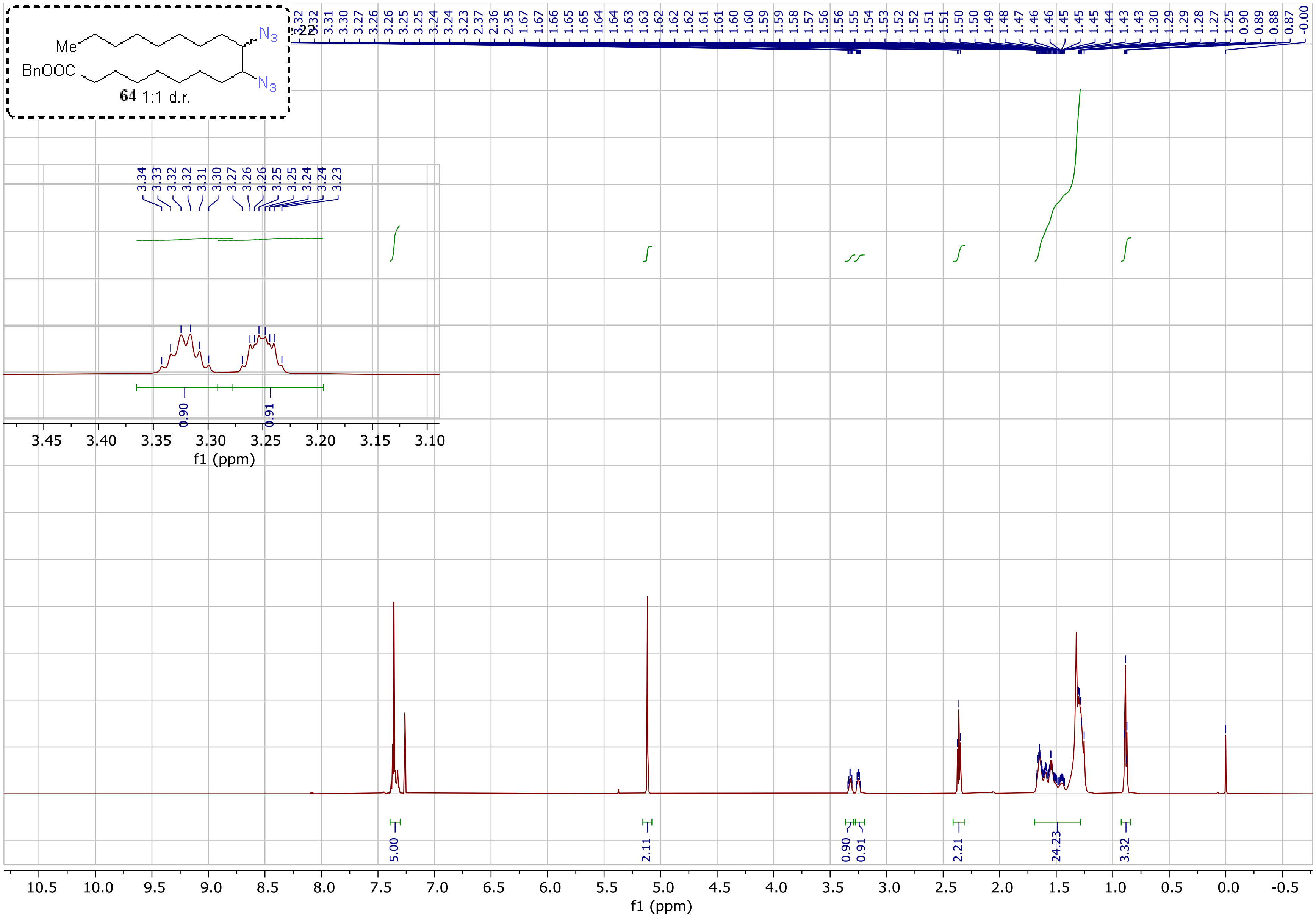
Me
BnOOC
N3
N3
64 1:1 d.r.
f1 (ppm)
f1 (ppm)

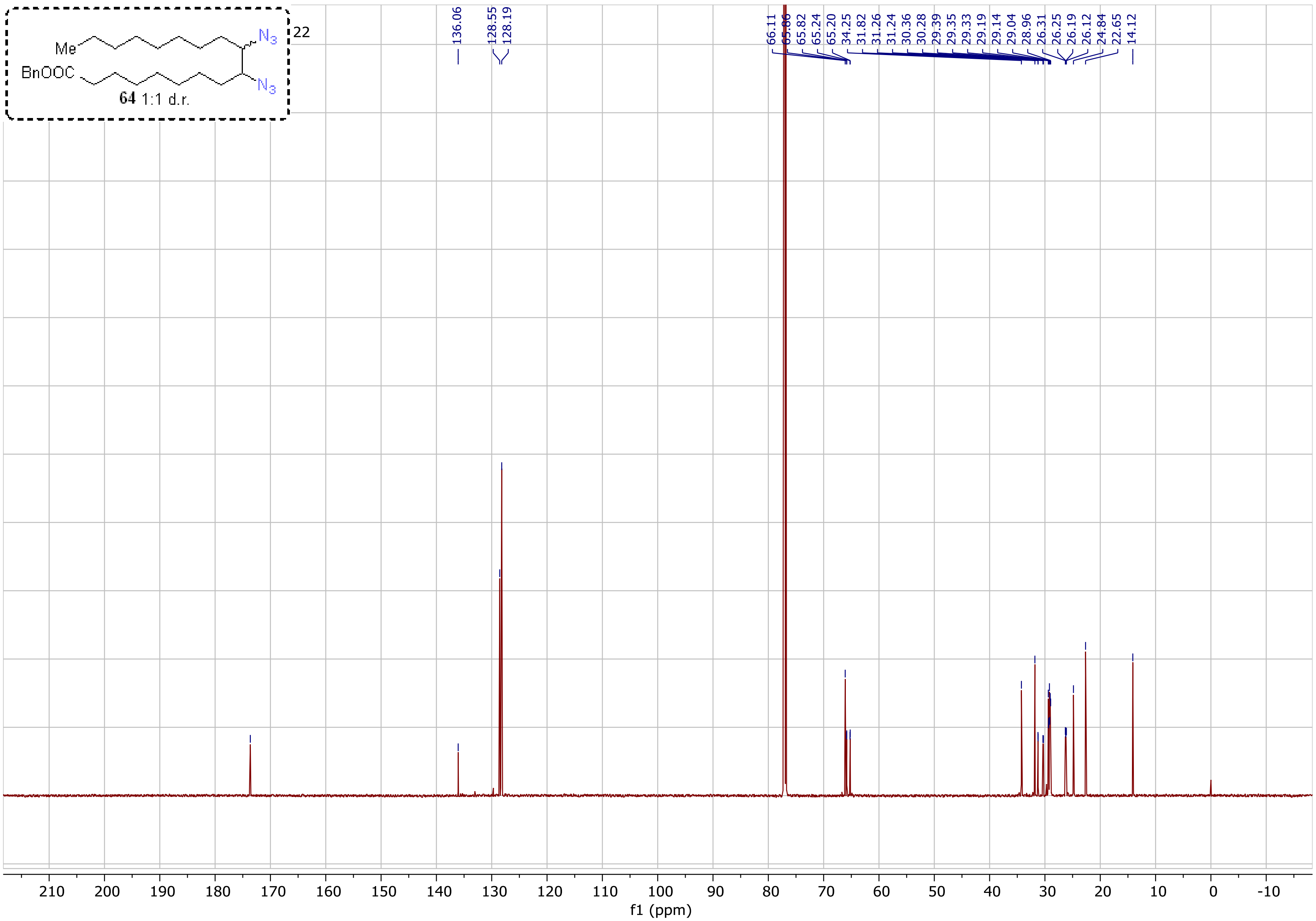

Me
BnOOC
N3
N3
64 1:1 d.r.
22
136.06
128.55
128.19
66.11
65.86
65.82
65.24
65.20
34.25
31.82
31.26
31.24
30.36
30.28
29.39
29.35
29.33
29.19
29.14
29.04
28.96
26.31
26.25
26.19
26.12
24.84
22.65
14.12
210
200
190
180
170
160
150
140
130
120
110
100
90
80
70
60
50
40
30
20
10
0
-10
f1 (ppm)

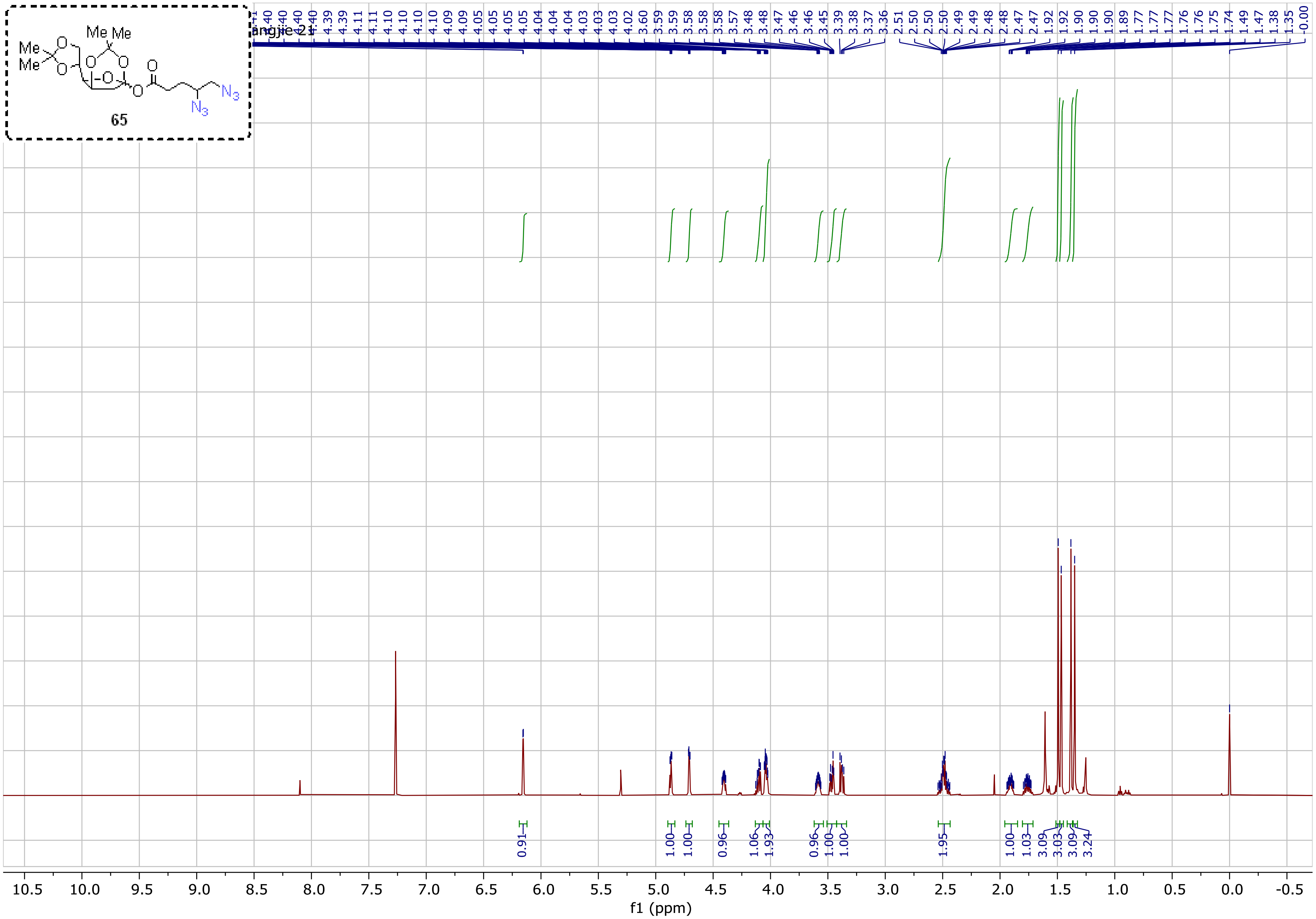

65
f1 (ppm)

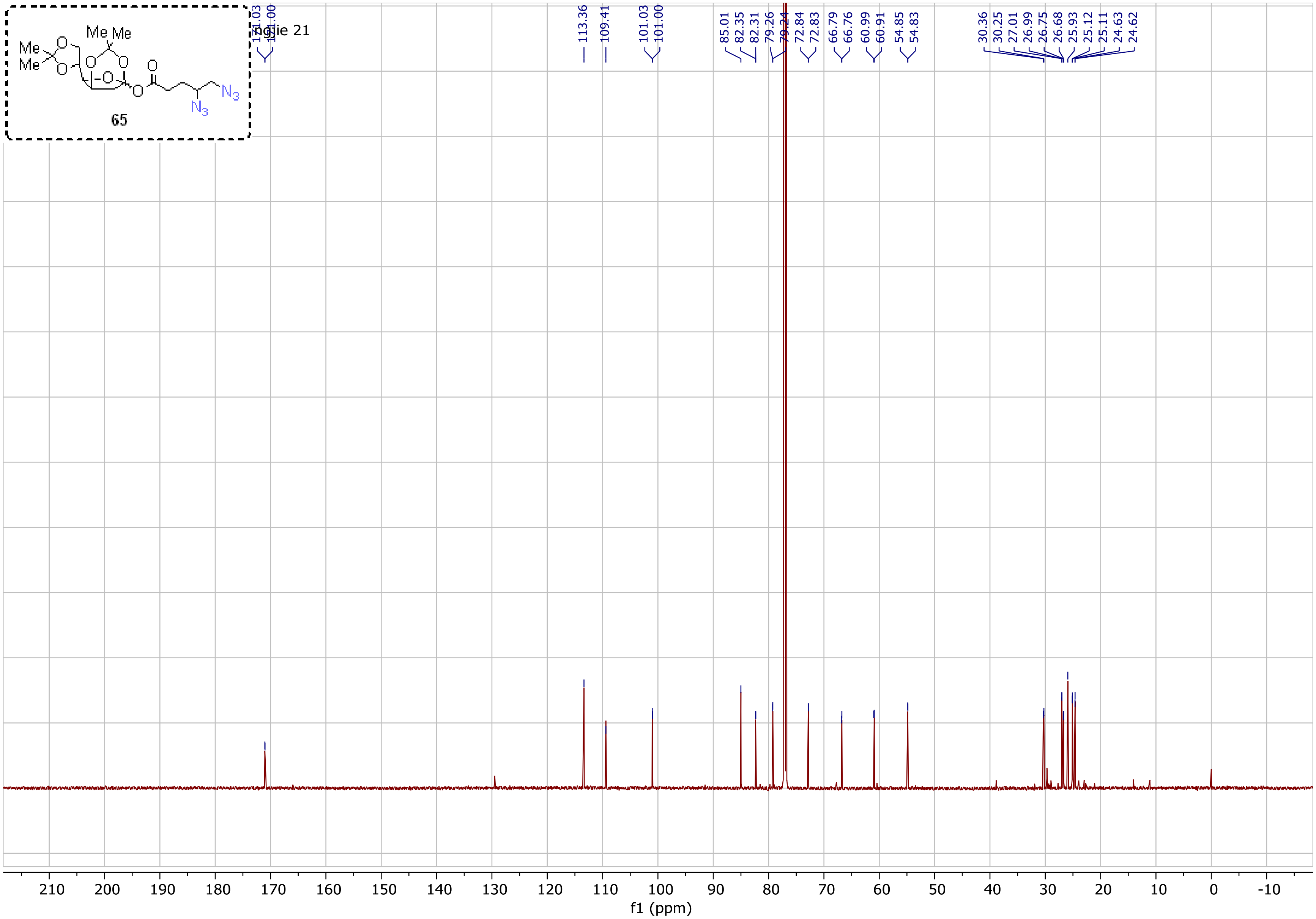
65
Me
Me
N3
ngjie 21
171.03
171.00
113.36
109.41
101.03
101.00
85.01
82.35
82.31
79.26
79.24
72.84
72.83
66.79
66.76
60.99
60.91
54.85
54.83
30.36
30.25
27.01
26.99
26.75
26.68
25.93
25.12
25.11
24.63
24.62
f1 (ppm)

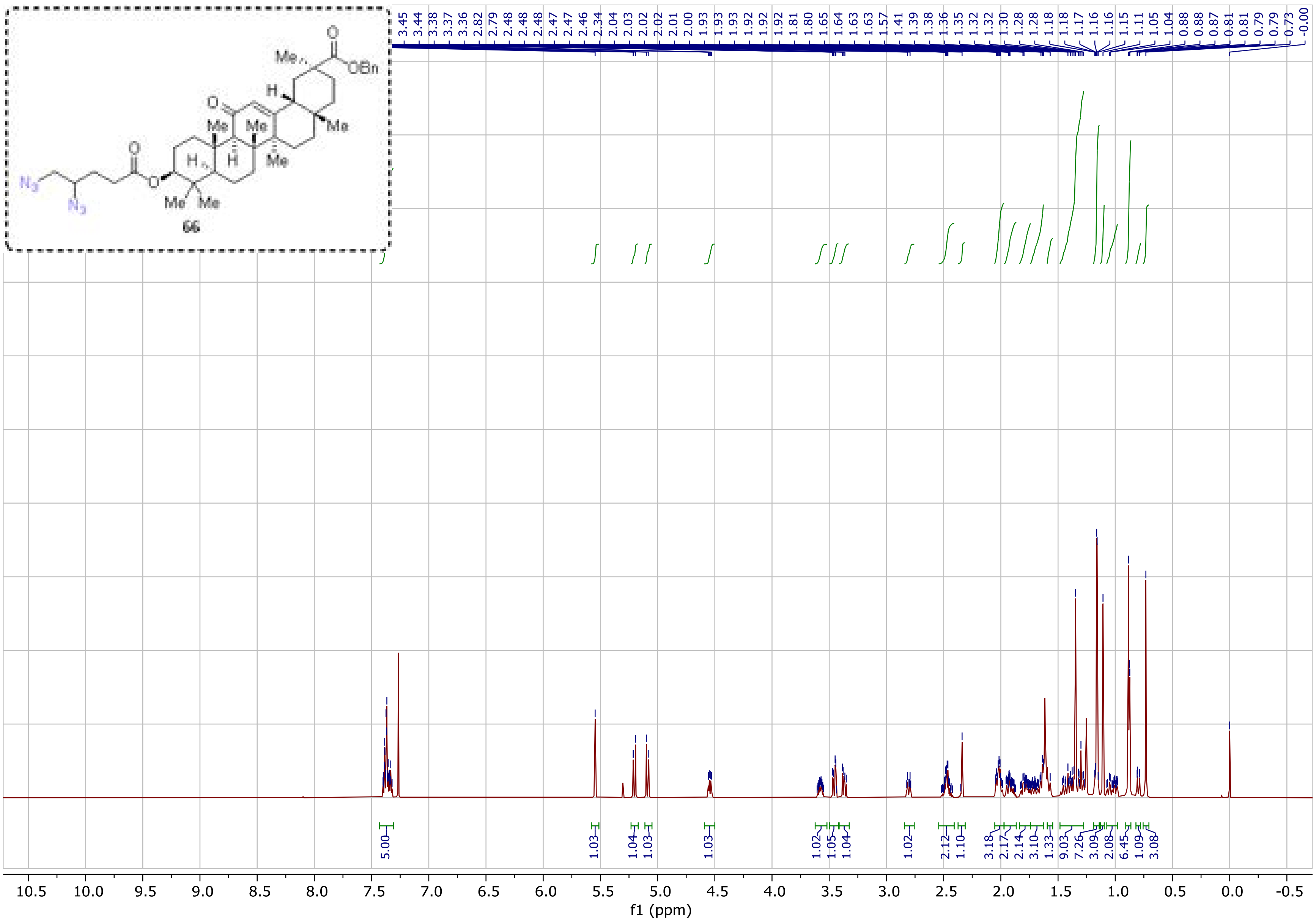

Me
OBn
H
O
Me
Me
Me
H
H
Me
N3
N3
O
Me
Me
66
f1 (ppm)

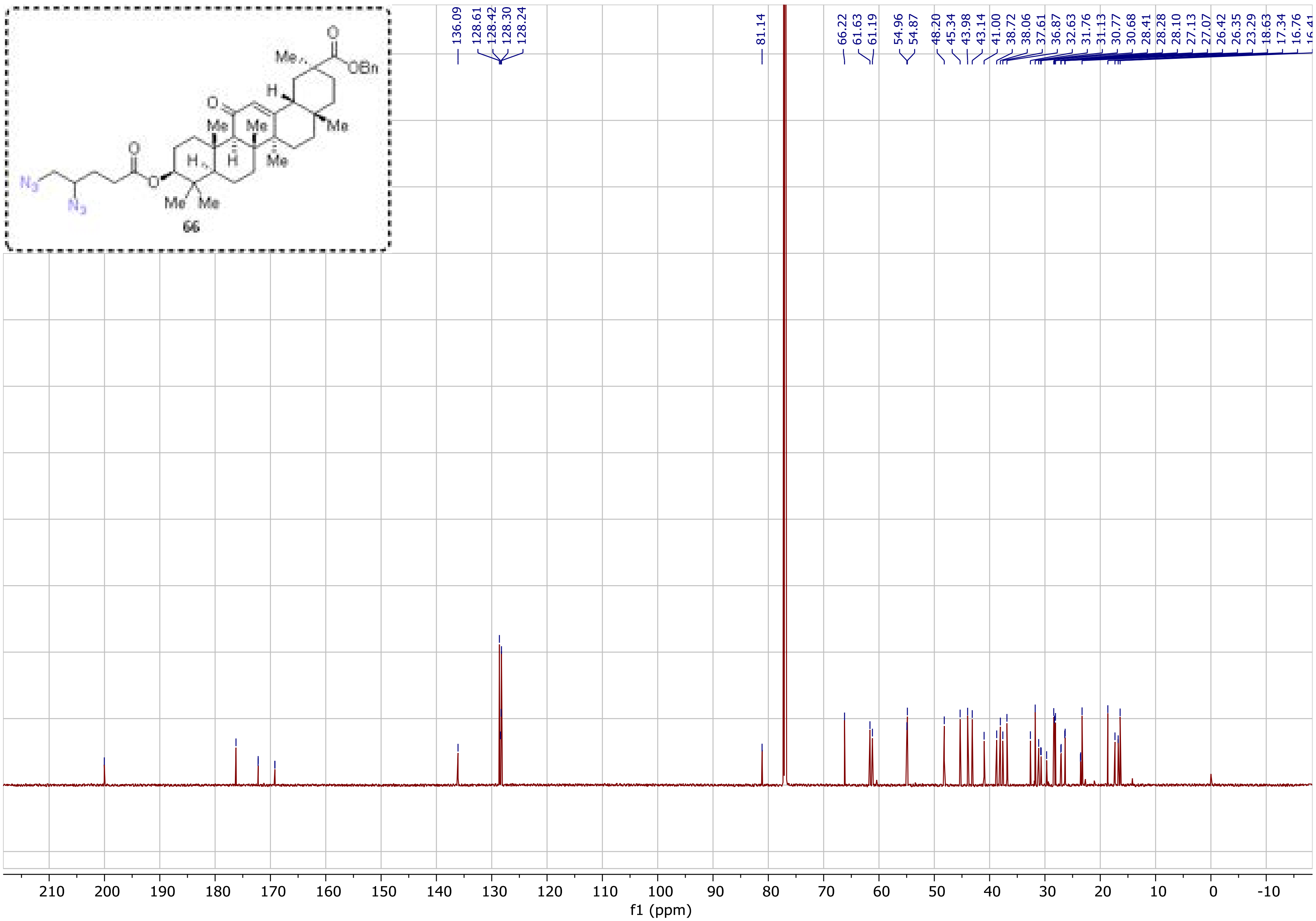

136.09
128.61
128.42
128.30
128.24
81.14
66.22
61.63
61.19
54.96
54.87
48.20
45.34
43.98
43.14
41.00
38.72
38.06
37.61
36.87
32.63
31.76
31.13
30.77
30.68
28.41
28.28
28.10
27.13
27.07
26.42
26.35
23.29
18.63
17.34
16.76
Me
O
OBn
H
O
Me
Me
Me
Me
O
H
H
N3
N3
O
Me
Me
66
210
200
190
180
170
160
150
140
130
120
110
100
90
80
70
60
50
40
30
20
10
0
-10
f1 (ppm)

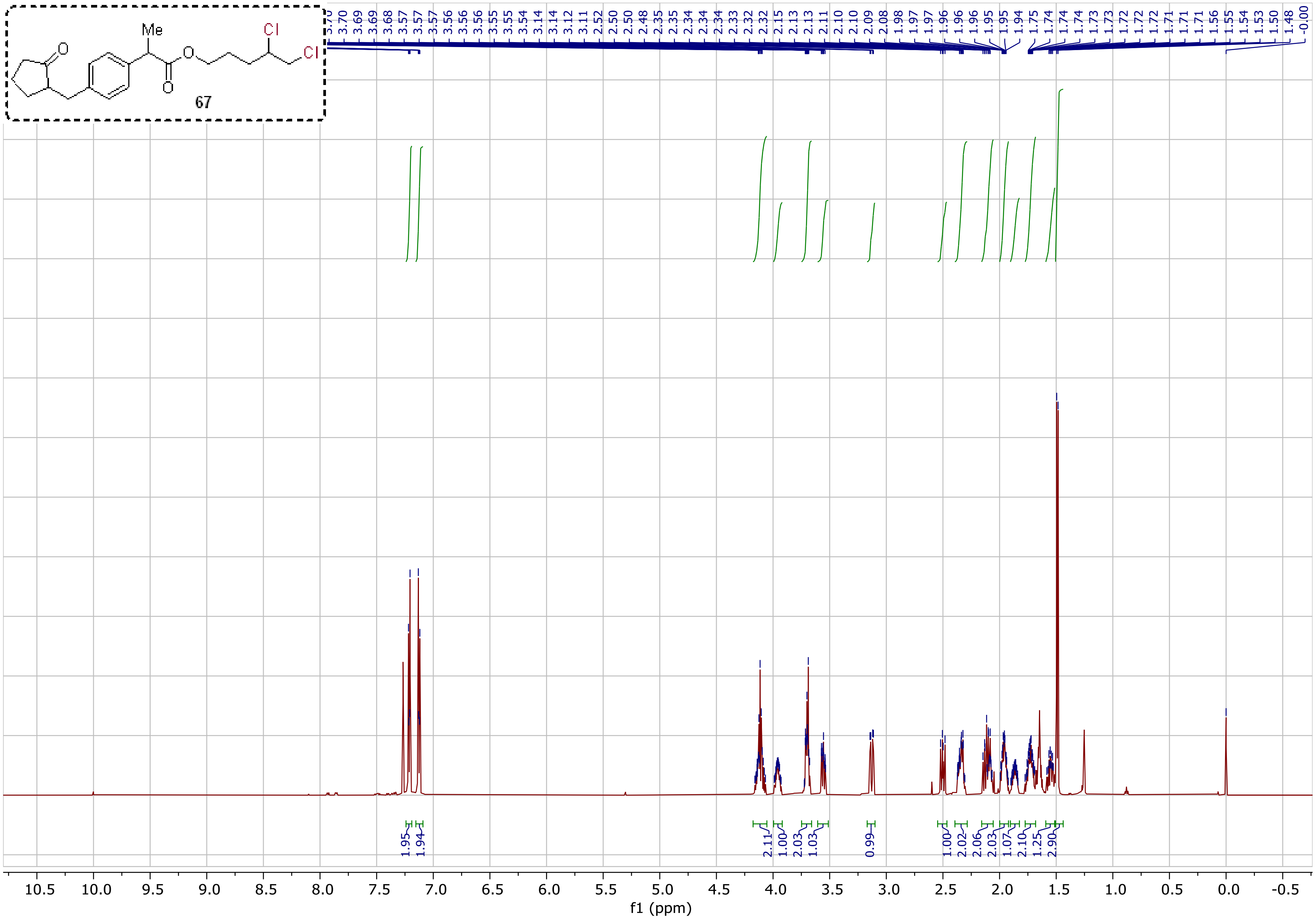

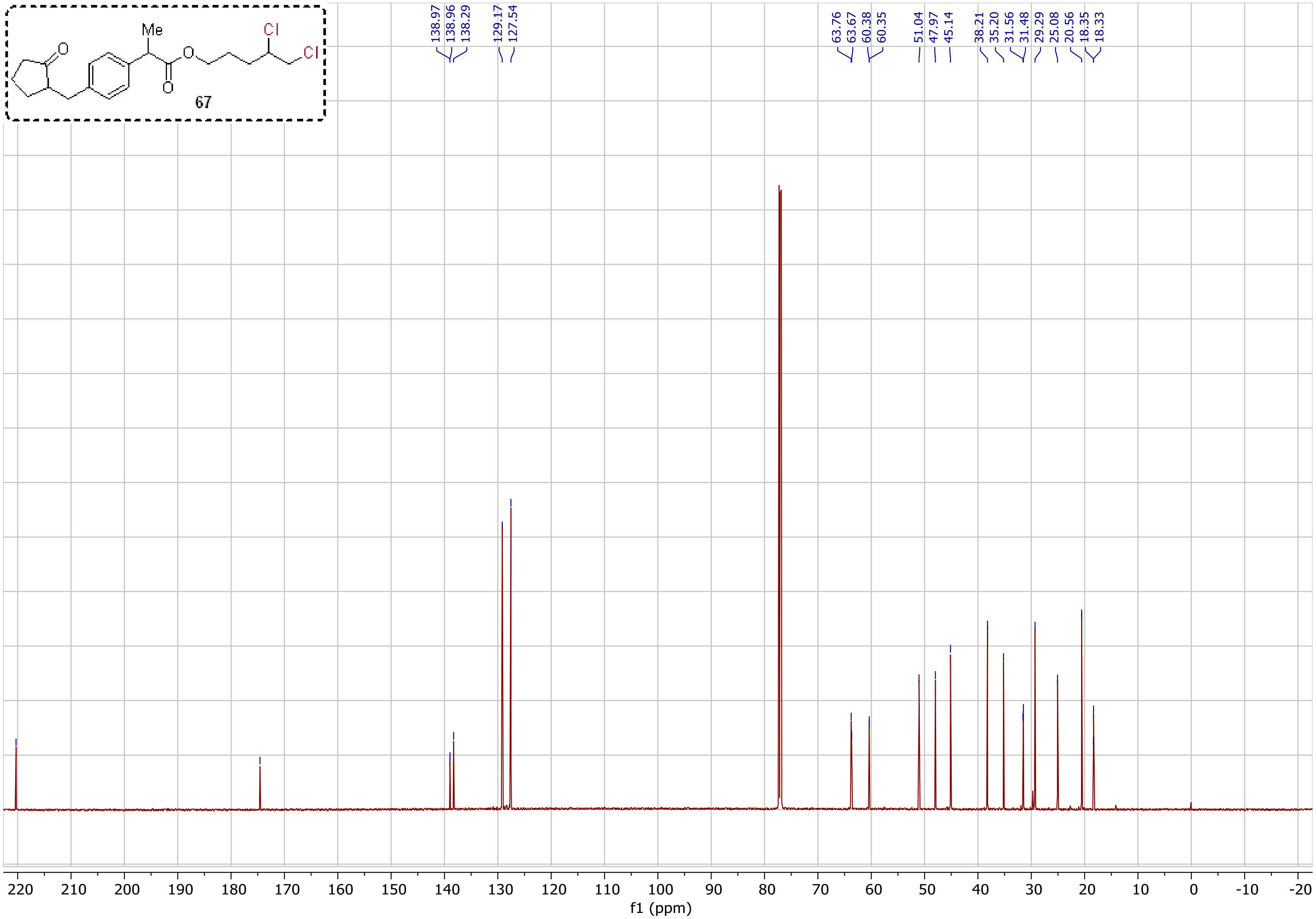

Me
O
Cl
Cl
67
138.97
138.96
138.29
129.17
127.54
63.76
63.67
60.38
60.35
51.04
47.97
45.14
38.21
35.20
31.56
31.48
29.29
25.08
20.56
18.35
18.33
220
210
200
190
180
170
160
150
140
130
120
110
100
90
80
70
60
50
40
30
20
10
0
-10
-20
f1 (ppm)

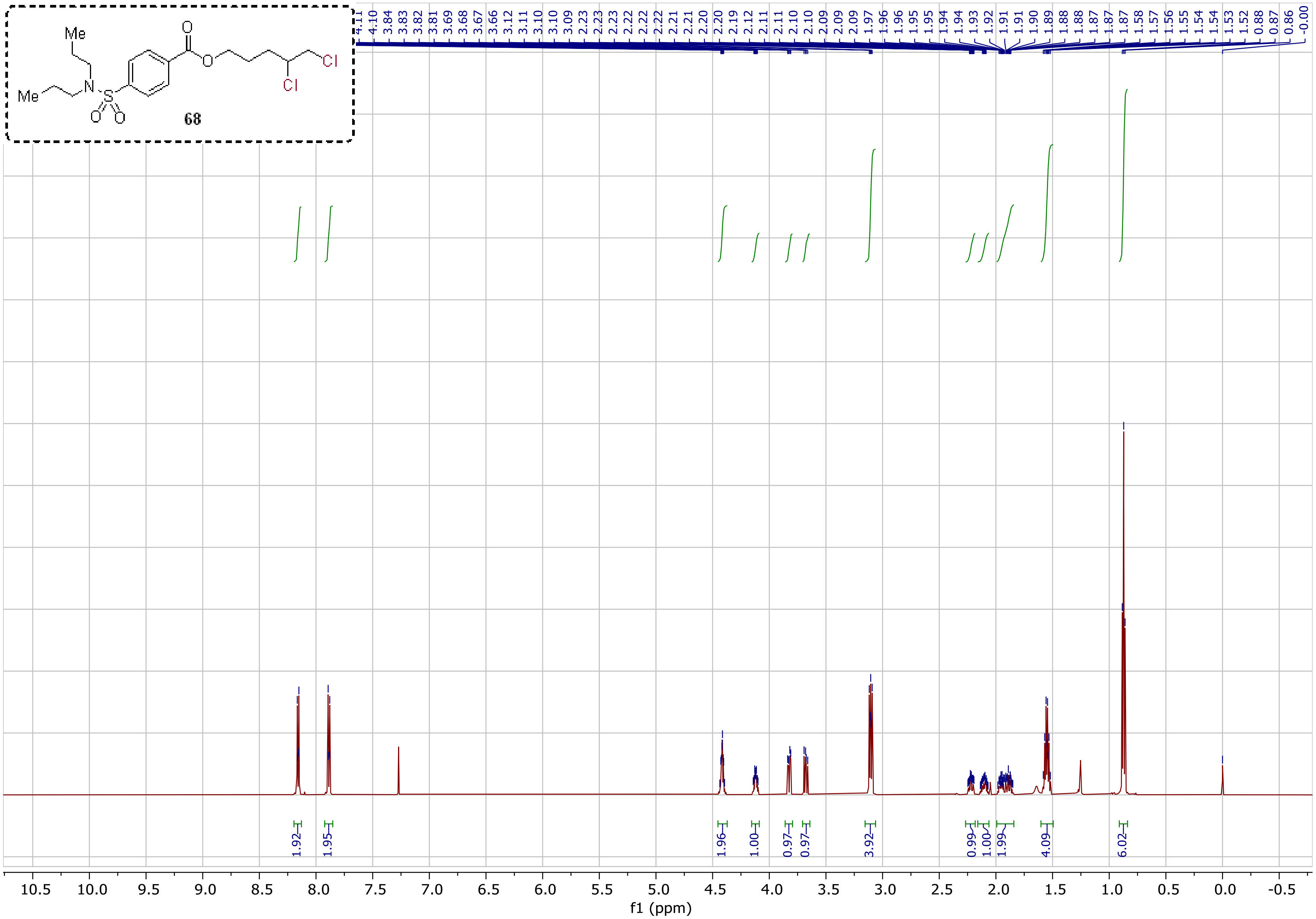

Me
Me
N
S
O
O
O
O
Cl
Cl
68
f1 (ppm)

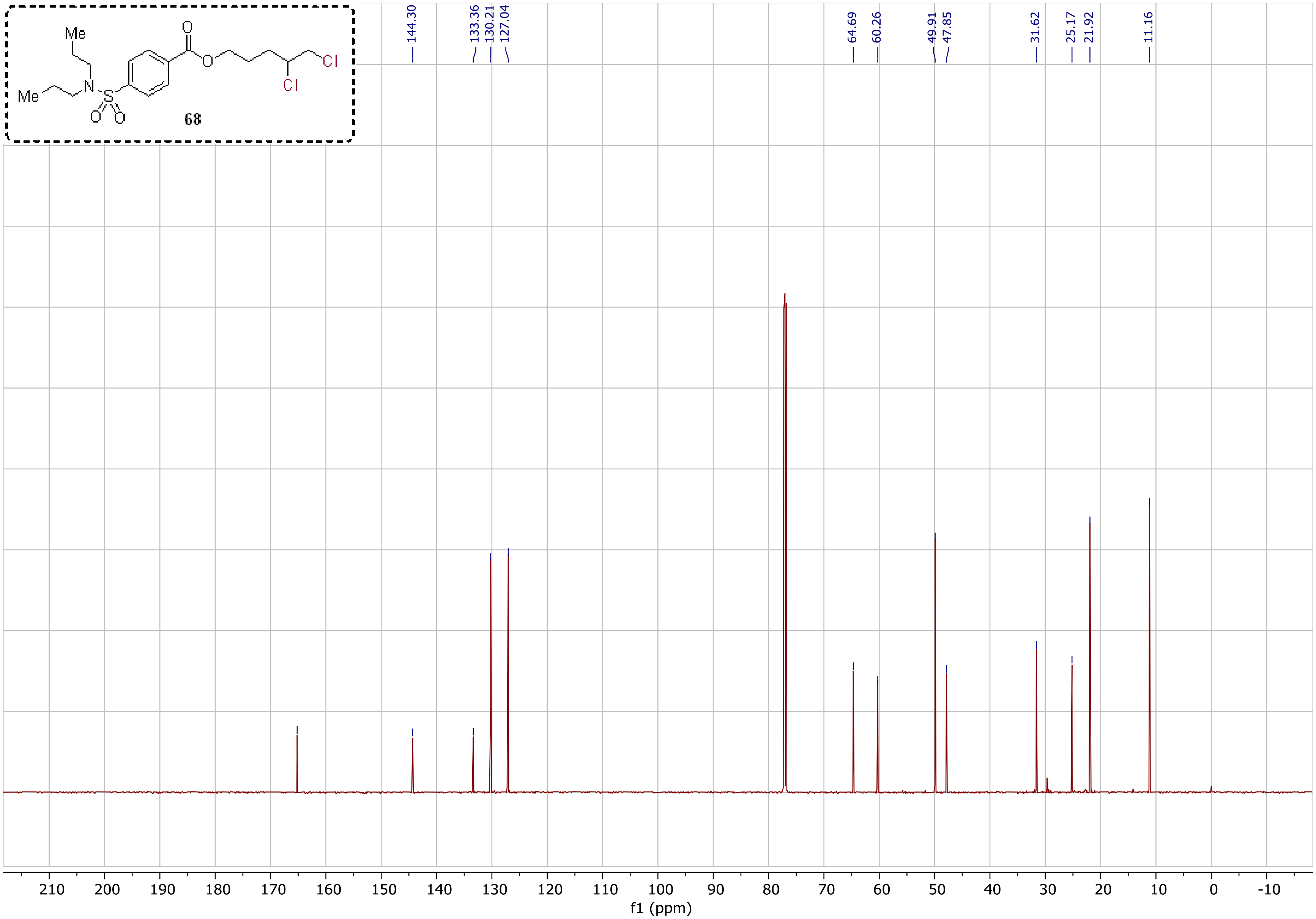

68
144.30
133.36
130.21
127.04
64.69
60.26
49.91
47.85
31.62
25.17
21.92
11.16
210 200 190 180 170 160 150 140 130 120 110 100 90 80 70 60 50 40 30 20 10 0 -10
f1 (ppm)

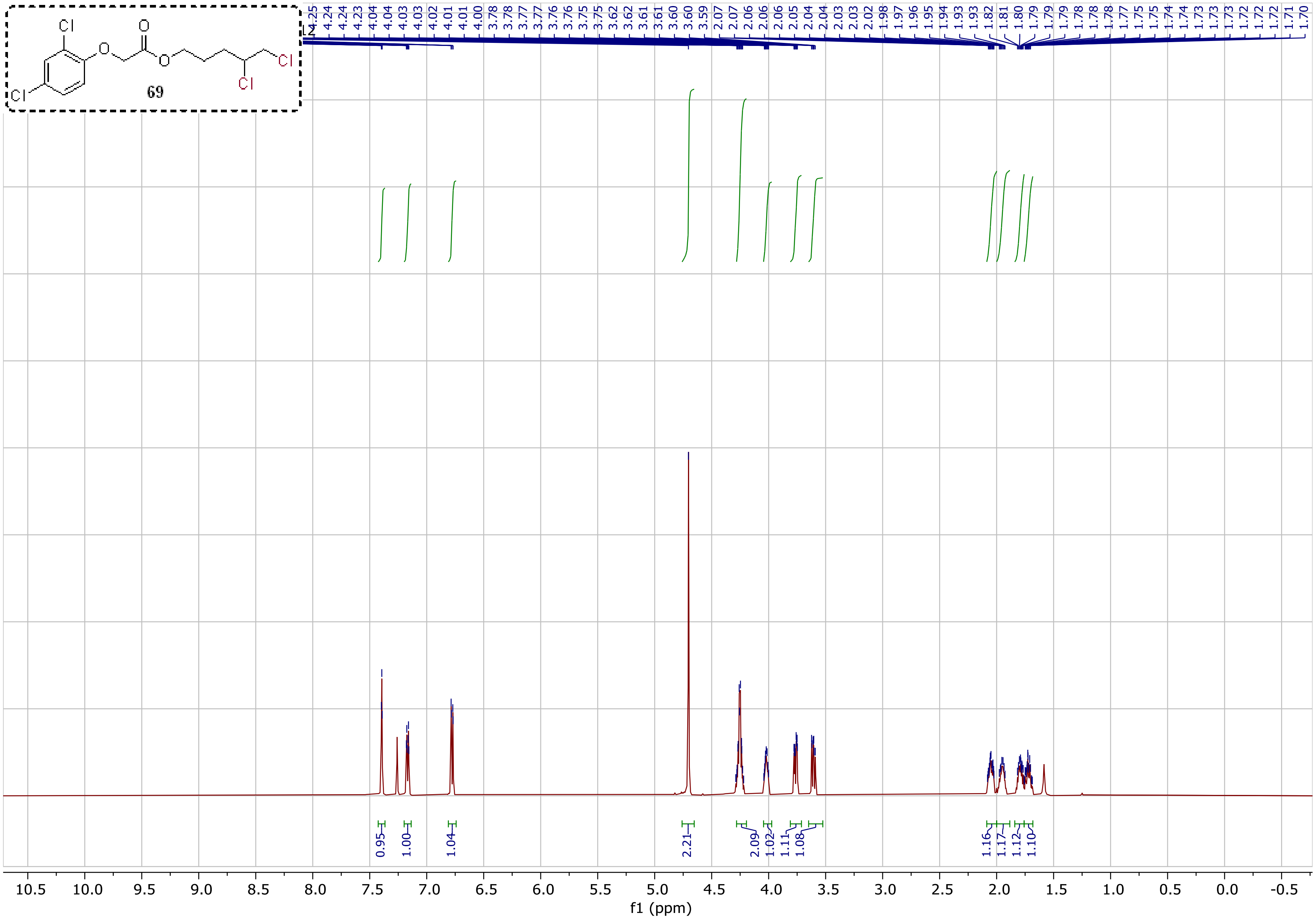
69
f1 (ppm)
10.5
10.0
9.5
9.0
8.5
8.0
7.5
7.0
6.5
6.0
5.5
5.0
4.5
4.0
3.5
3.0
2.5
2.0
1.5
1.0
0.5
0.0
-0.5
0.95
1.00
1.04
2.21
2.09
1.02
1.11
1.08
1.16
1.17
1.12
1.10

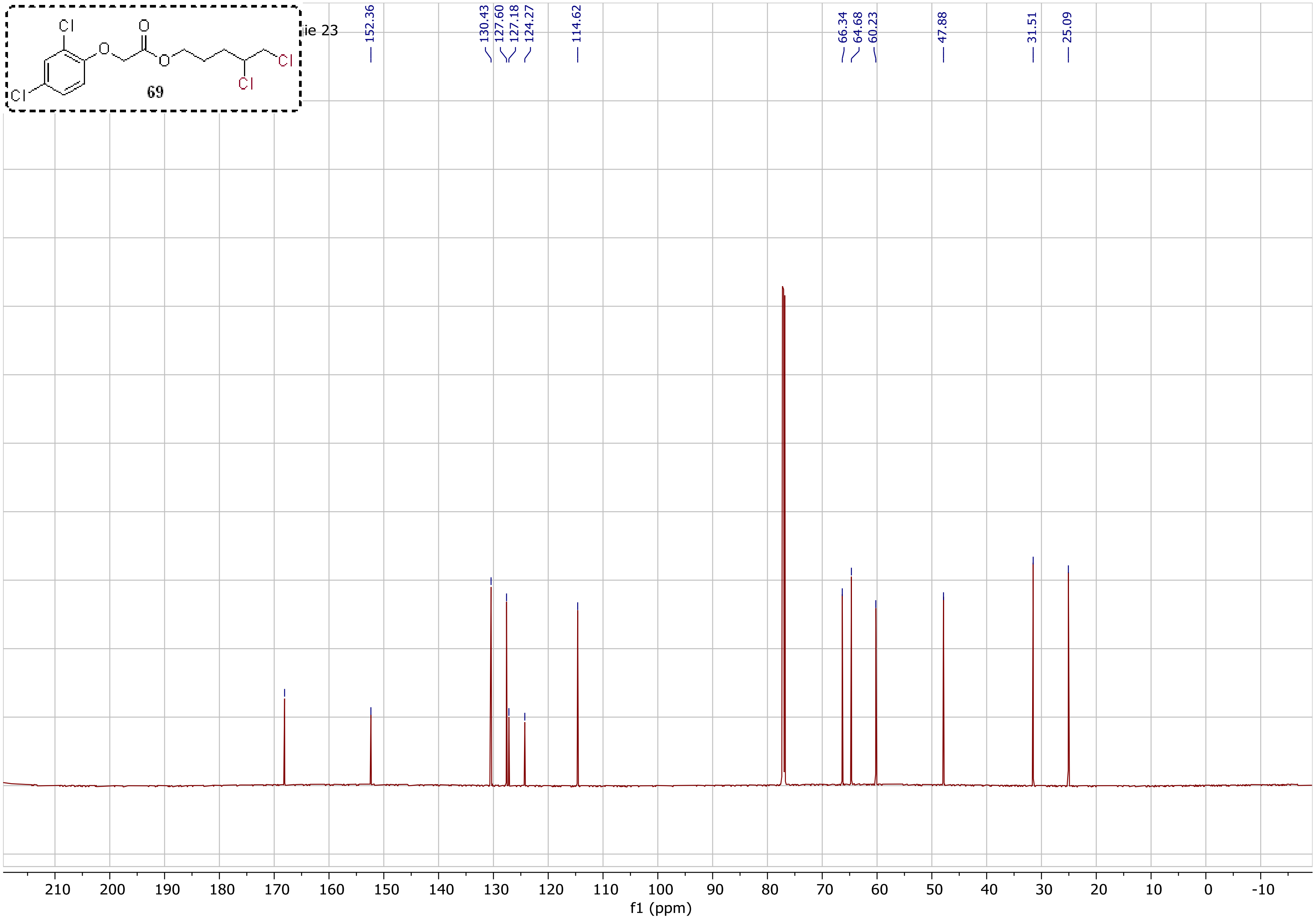

69
ie 23
152.36
130.43
127.60
127.18
124.27
114.62
66.34
64.68
60.23
47.88
31.51
25.09
210
200
190
180
170
160
150
140
130
120
110
100
90
80
70
60
50
40
30
20
10
0
-10
f1 (ppm)

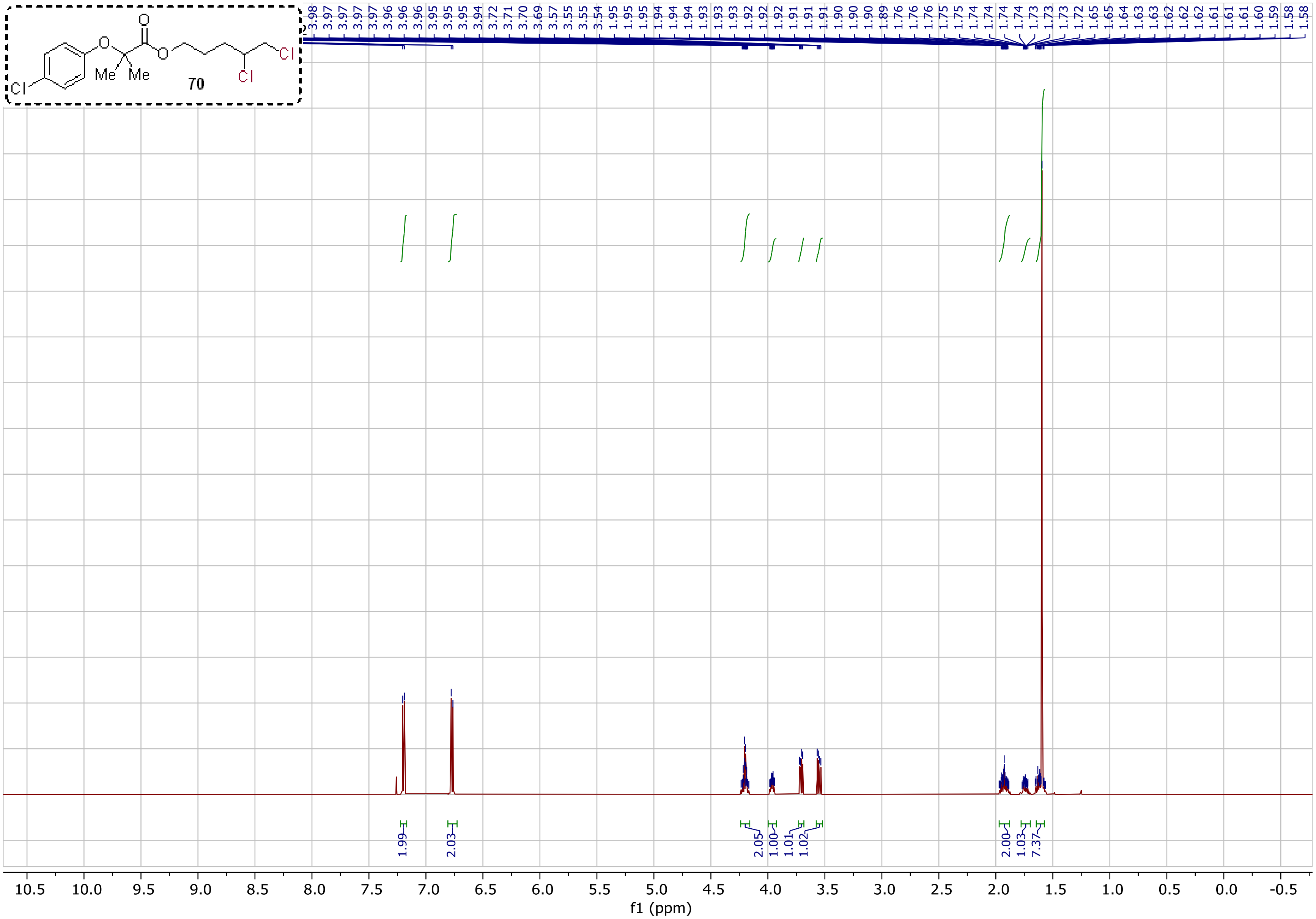
70
Me
Me
Cl
1.99
2.03
2.05
1.00
1.01
1.02
2.00
1.03
7.37
10.5 10.0 9.5 9.0 8.5 8.0 7.5 7.0 6.5 6.0 5.5 5.0 4.5 4.0 3.5 3.0 2.5 2.0 1.5 1.0 0.5 0.0 -0.5
f1 (ppm)

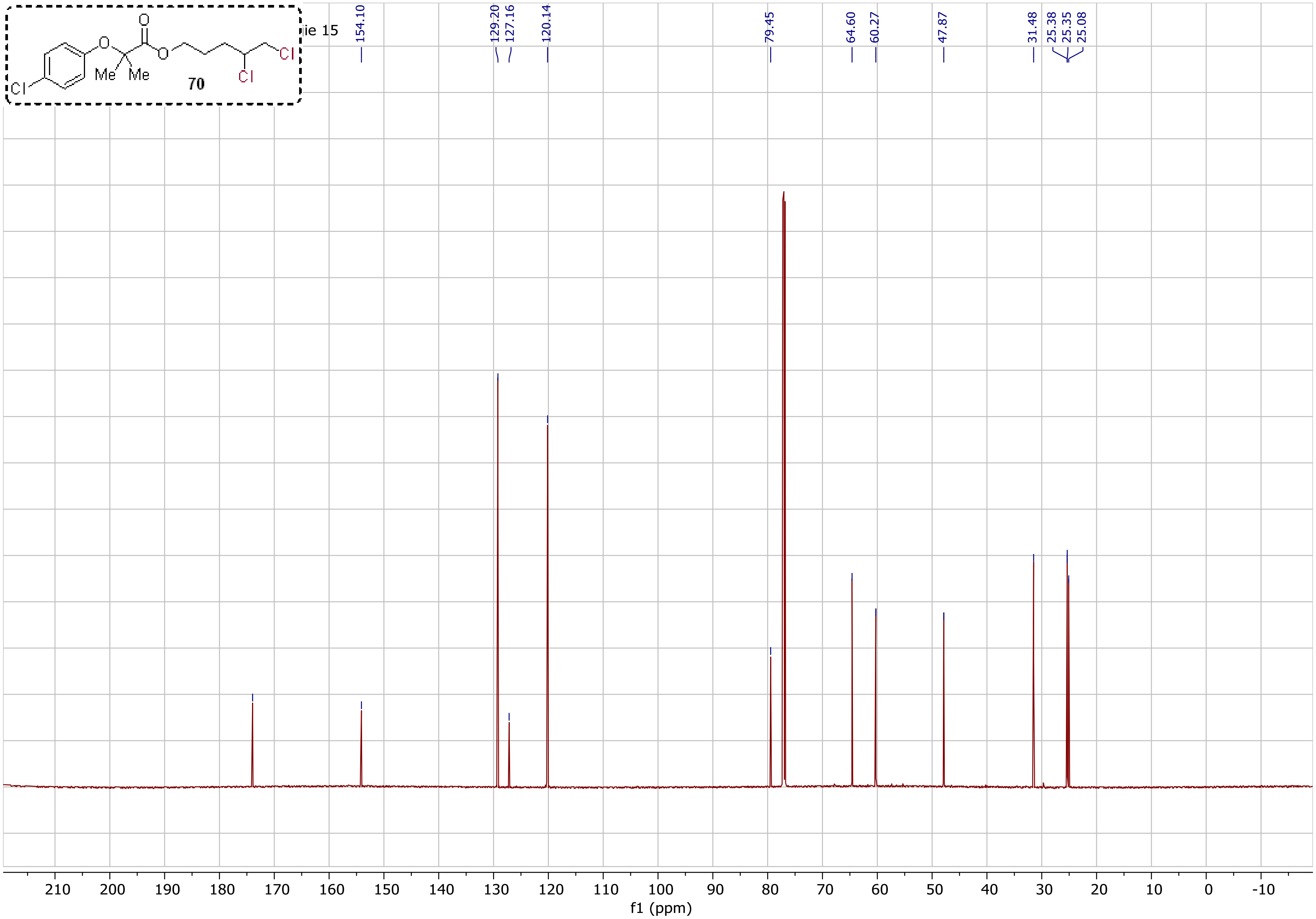

Cl
O
O
O
Me
Me
Cl
Cl
70
ie 15
154.10
129.20
127.16
120.14
79.45
64.60
60.27
47.87
31.48
25.38
25.35
25.08
210
200
190
180
170
160
150
140
130
120
110
100
90
80
70
60
50
40
30
20
10
0
-10
f1 (ppm)

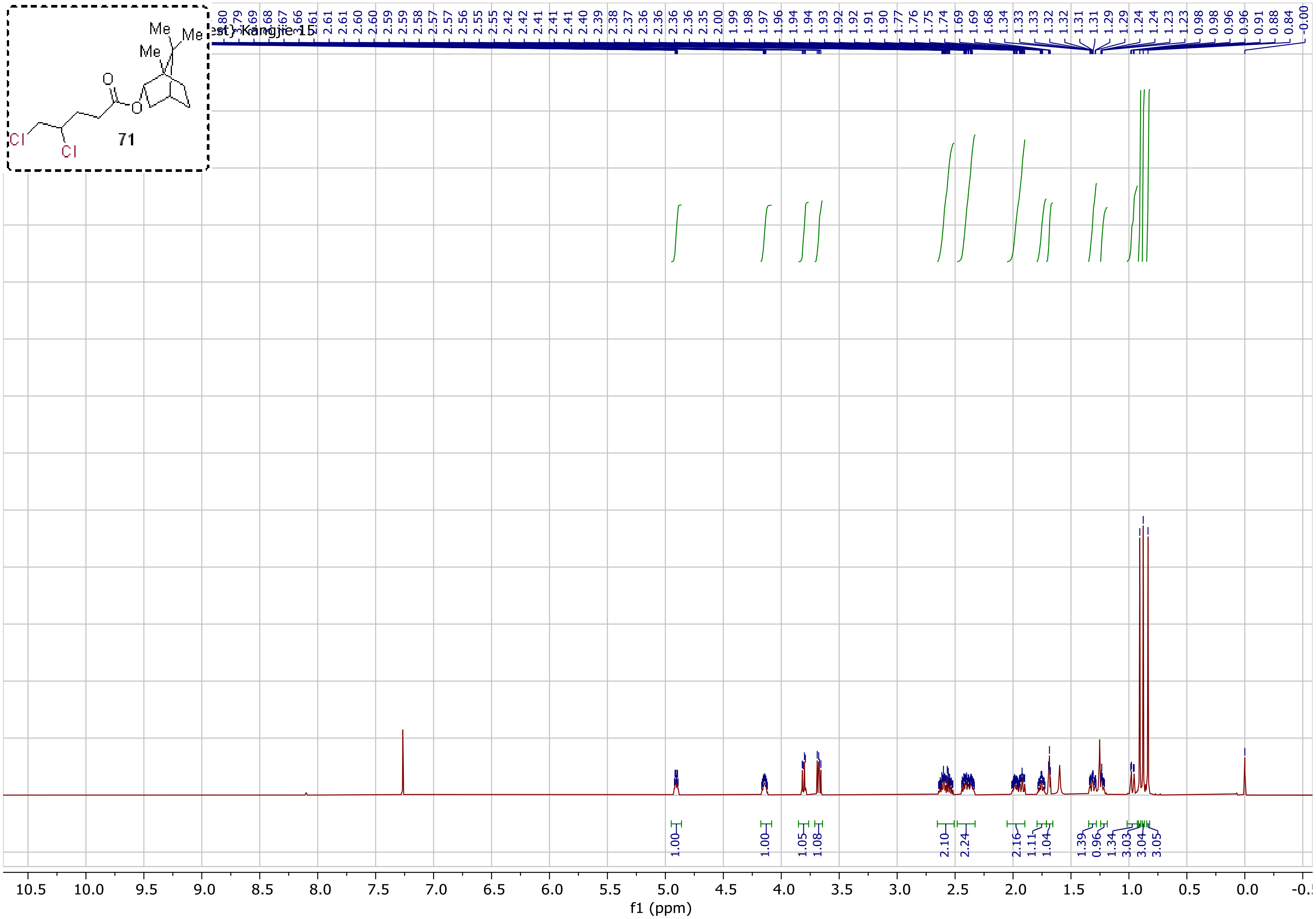
71
f1 (ppm)

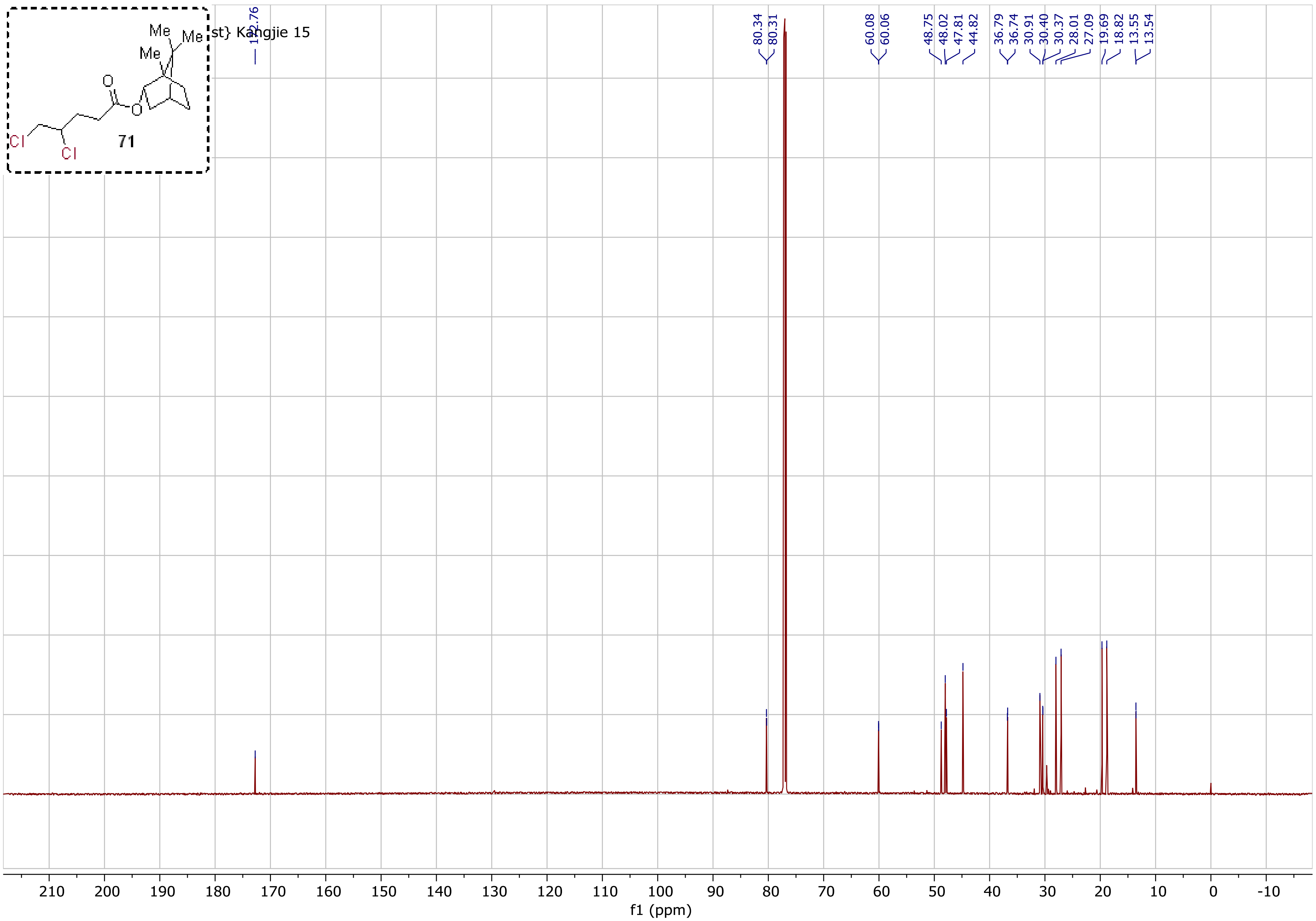

st} Kangjie 15
Me
Me
Me
O
O
Cl
Cl
71
80.34
80.31
60.08
60.06
48.75
48.02
47.81
44.82
36.79
36.74
30.91
30.40
30.37
28.01
27.09
19.69
18.82
13.55
13.54
210 200 190 180 170 160 150 140 130 120 110 100 90 80 70 60 50 40 30 20 10 0 -10
f1 (ppm)

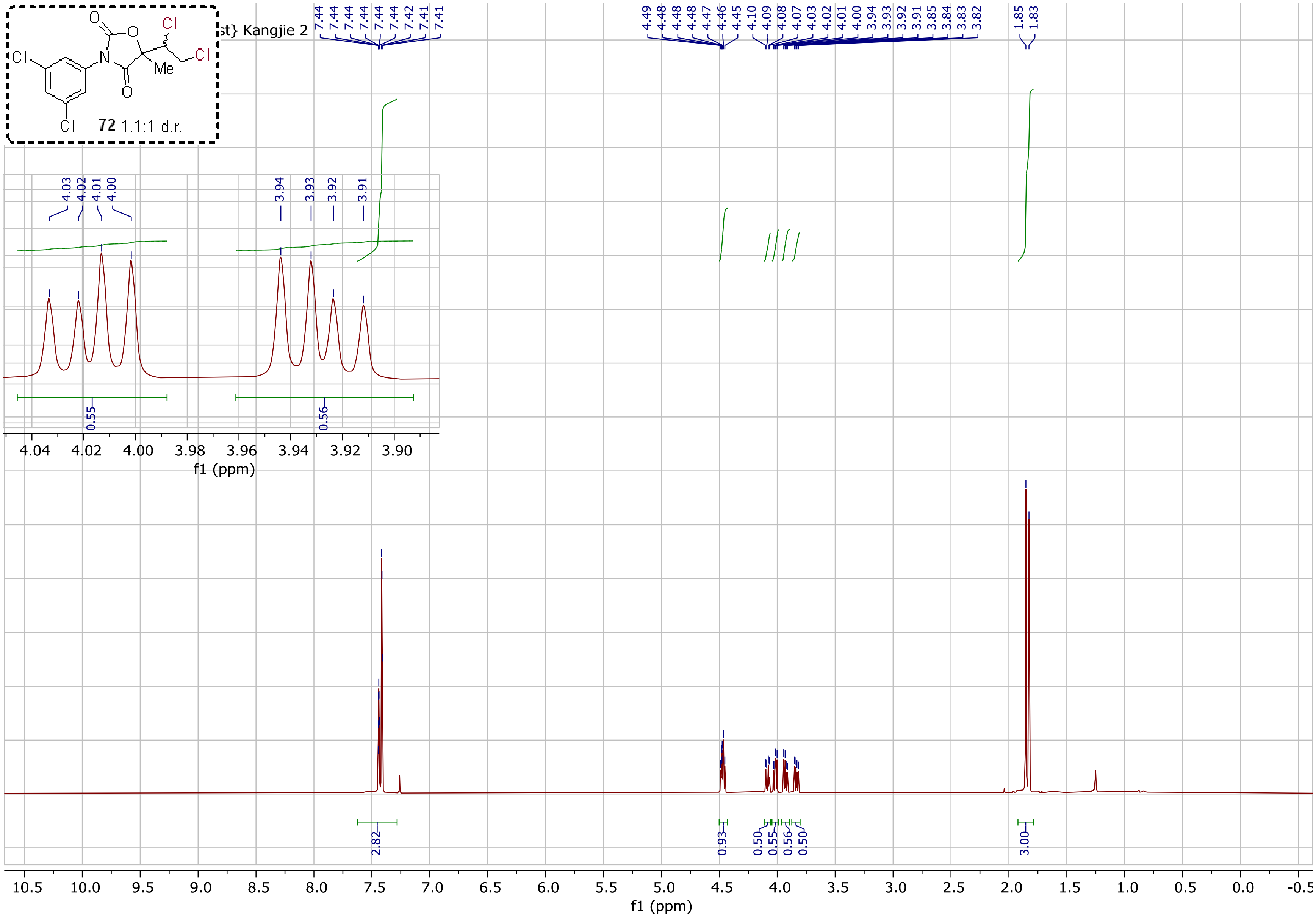
st} Kangjie 2
72 1.1:1 d.r.
f1 (ppm)
f1 (ppm)

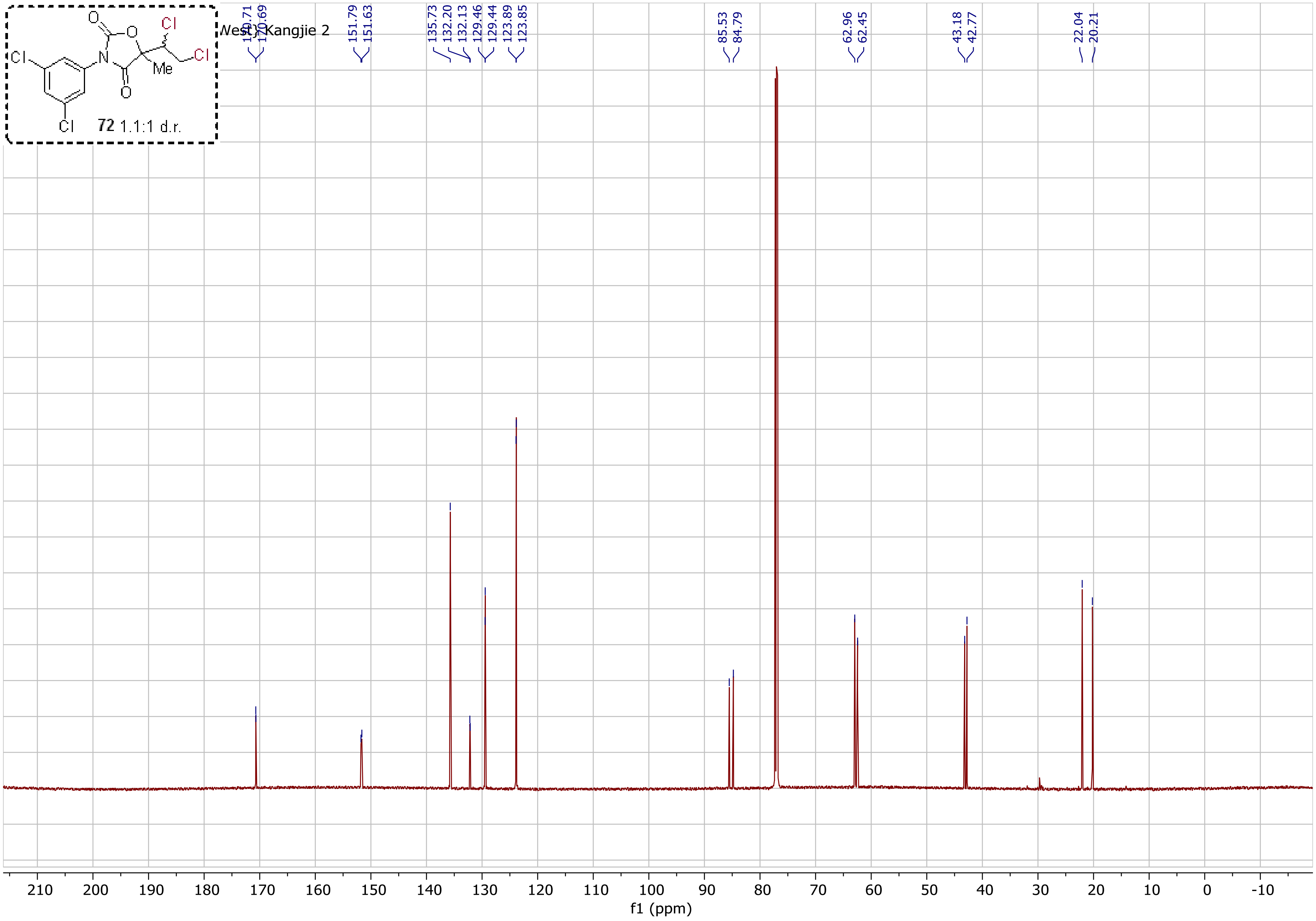

West) Kangjie 2
170.71
170.69
151.79
151.63
135.73
132.20
132.13
129.46
129.44
123.89
123.85
85.53
84.79
62.96
62.45
43.18
42.77
22.04
20.21
72 1.1:1 d.r.
f1 (ppm)

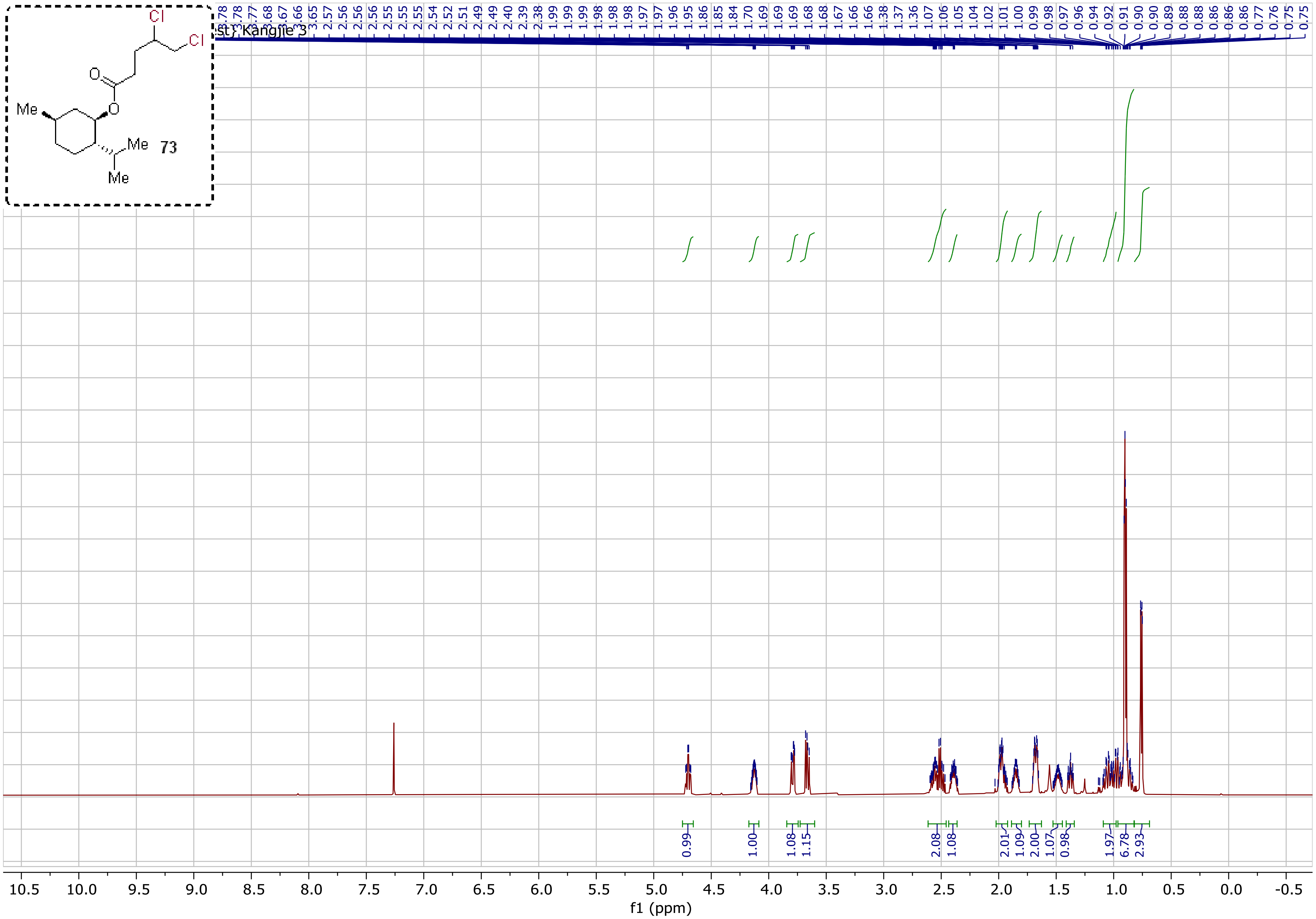

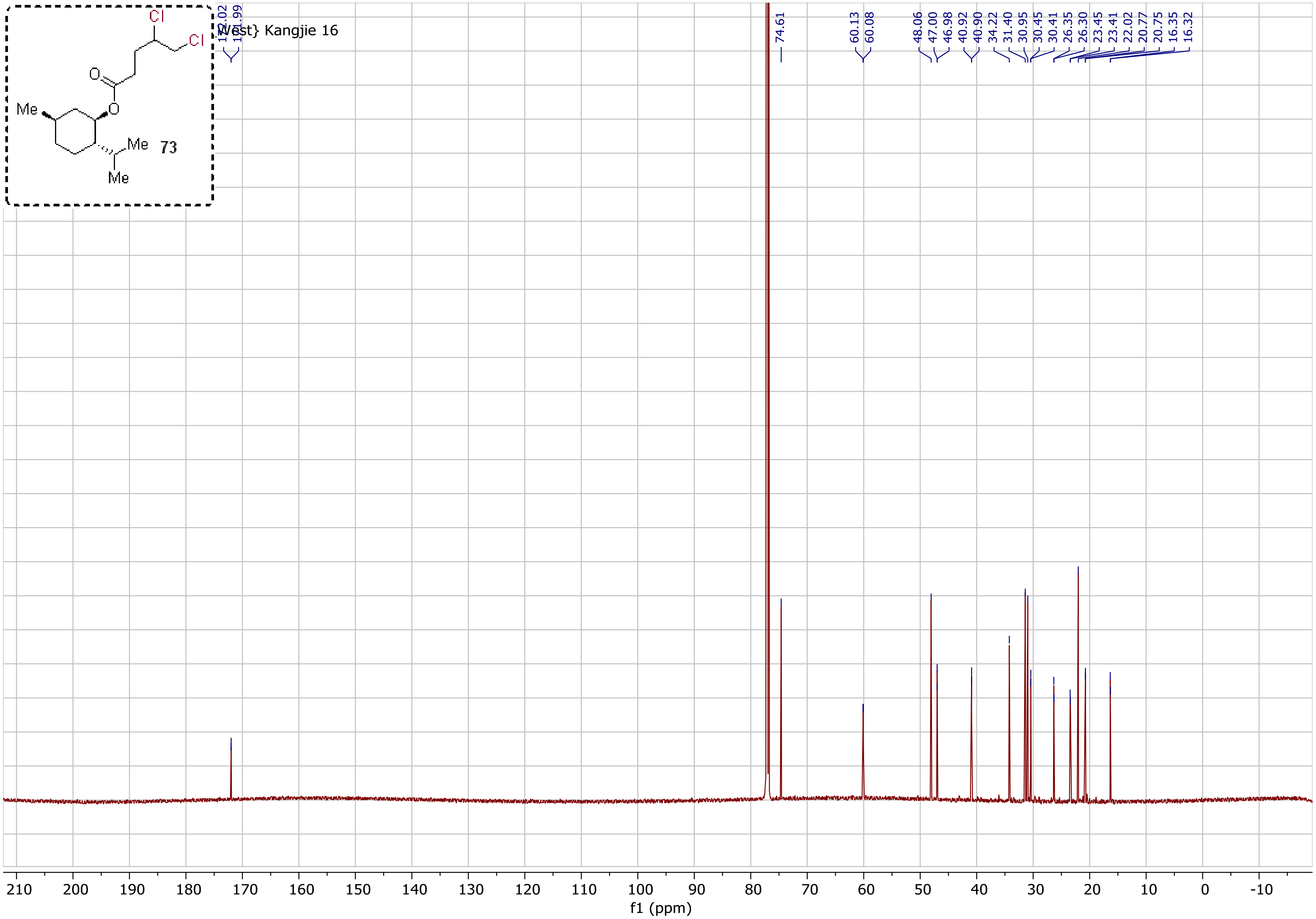

West} Kangjie 16
74.61
60.13
60.08
48.06
47.00
46.98
40.92
40.90
34.22
31.40
30.95
30.45
30.41
26.35
26.30
23.45
23.41
22.02
20.77
20.75
16.35
16.32
Cl
Cl
O
O
Me
Me
Me
73
210 200 190 180 170 160 150 140 130 120 110 100 90 80 70 60 50 40 30 20 10 0 -10
f1 (ppm)

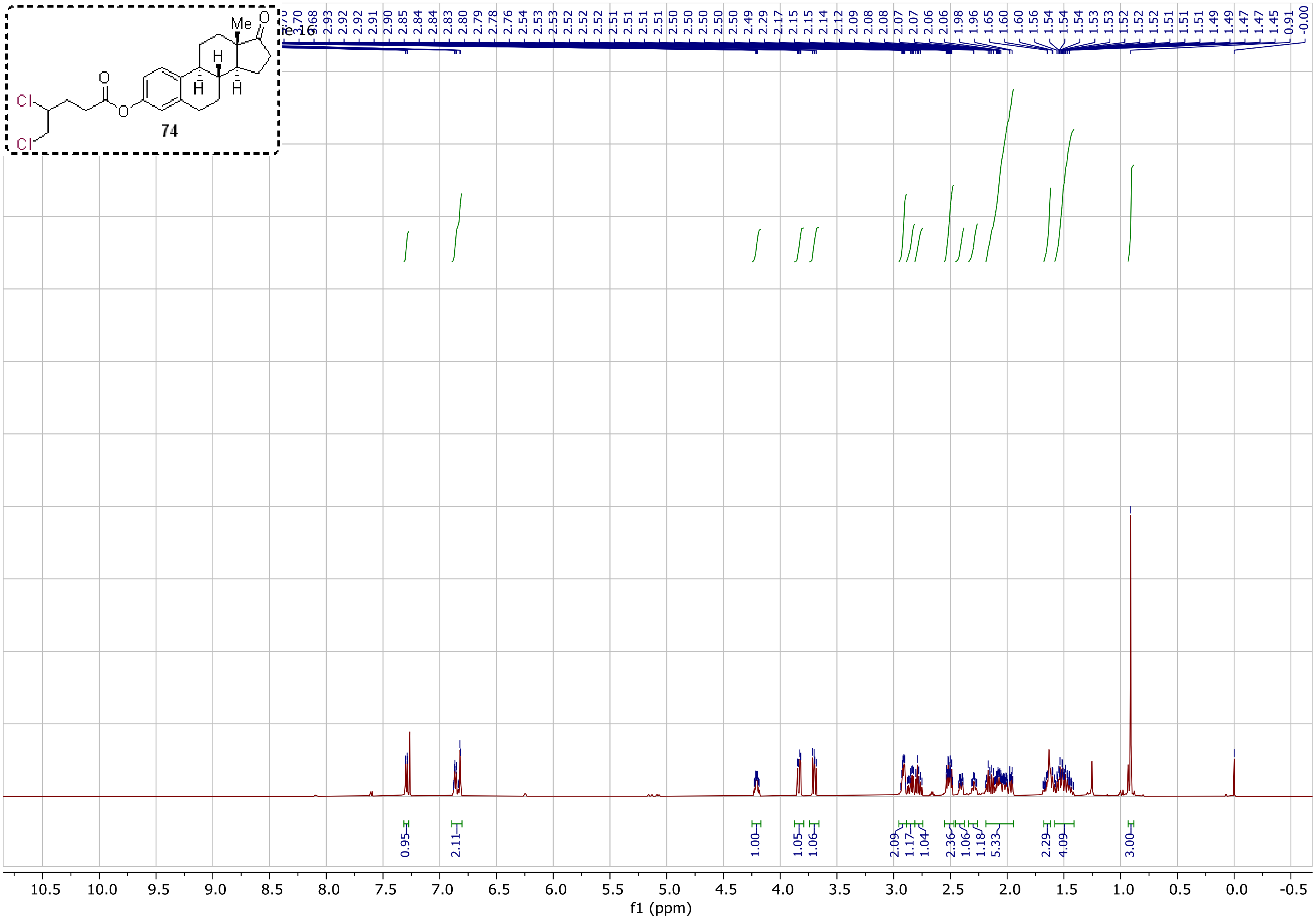

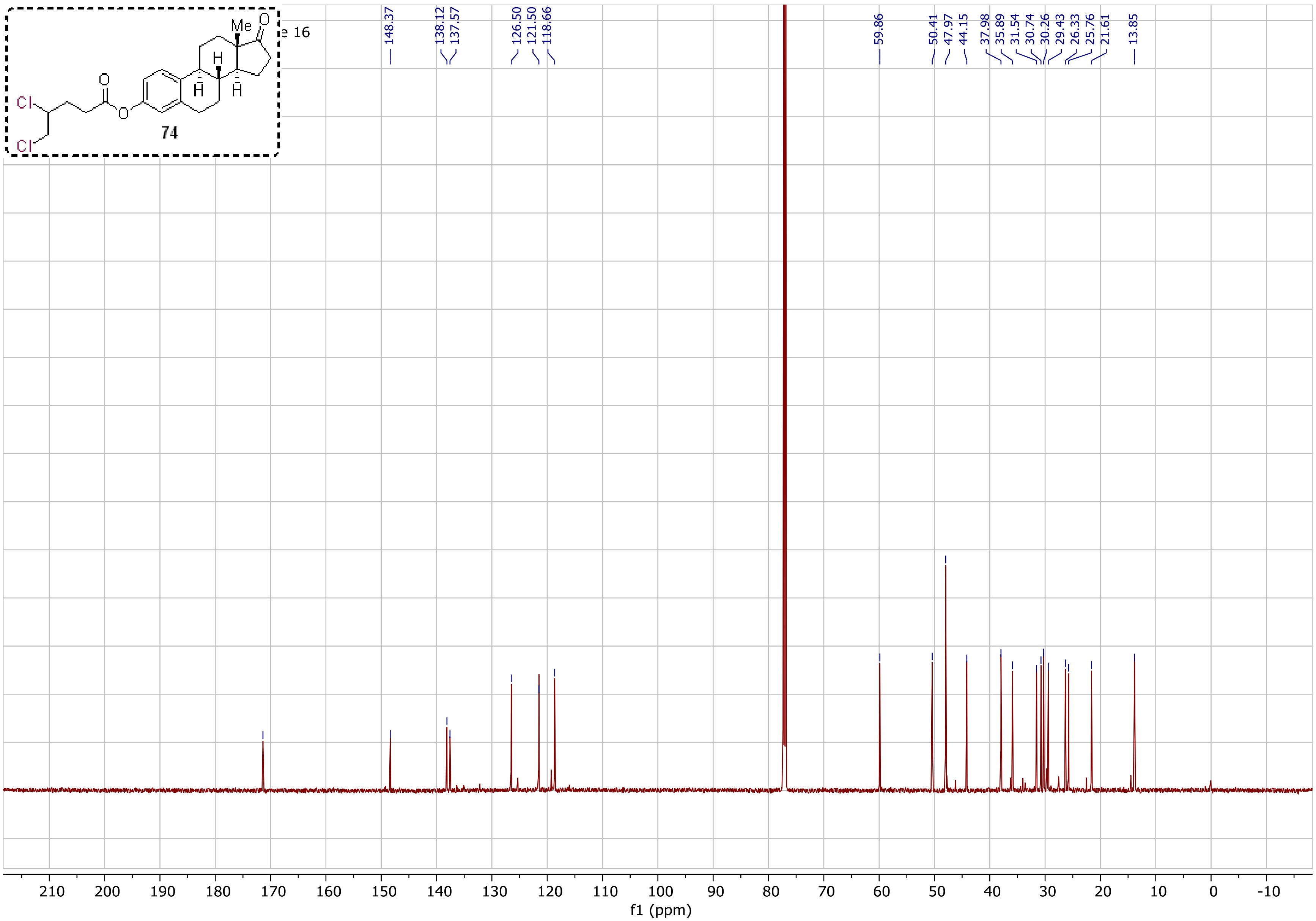
Me
O
H
H
H
Cl
Cl
O
O
74
e 16
148.37
138.12
137.57
126.50
121.50
118.66
59.86
50.41
47.97
44.15
37.98
35.89
31.54
30.74
30.26
29.43
26.33
25.76
21.61
13.85
210 200 190 180 170 160 150 140 130 120 110 100 90 80 70 60 50 40 30 20 10 0 -10
f1 (ppm)

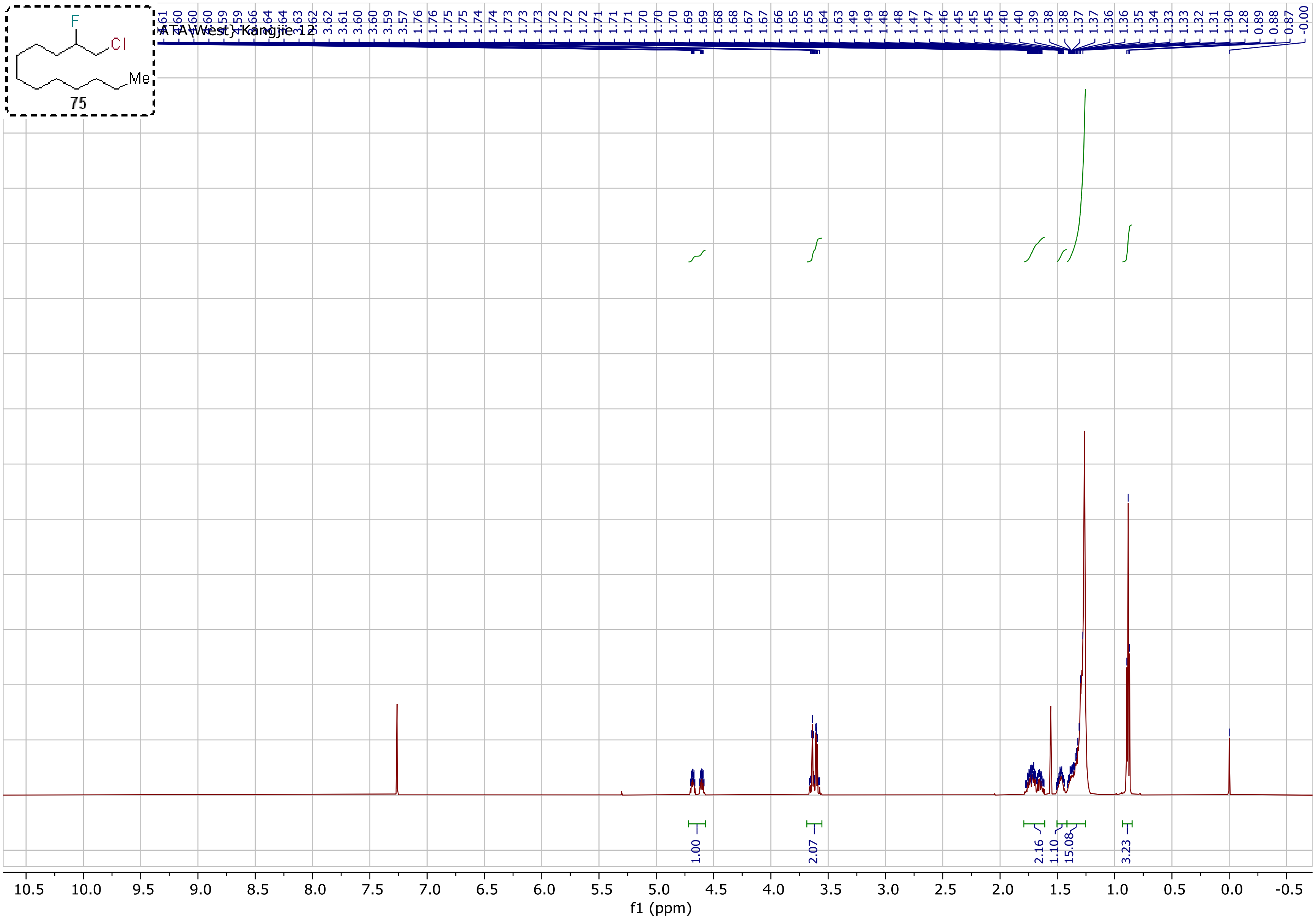

F
Cl
Me
75
ATA-West}-Kangjie 12
1.00
2.07
2.16
1.10
15.08
3.23
f1 (ppm)

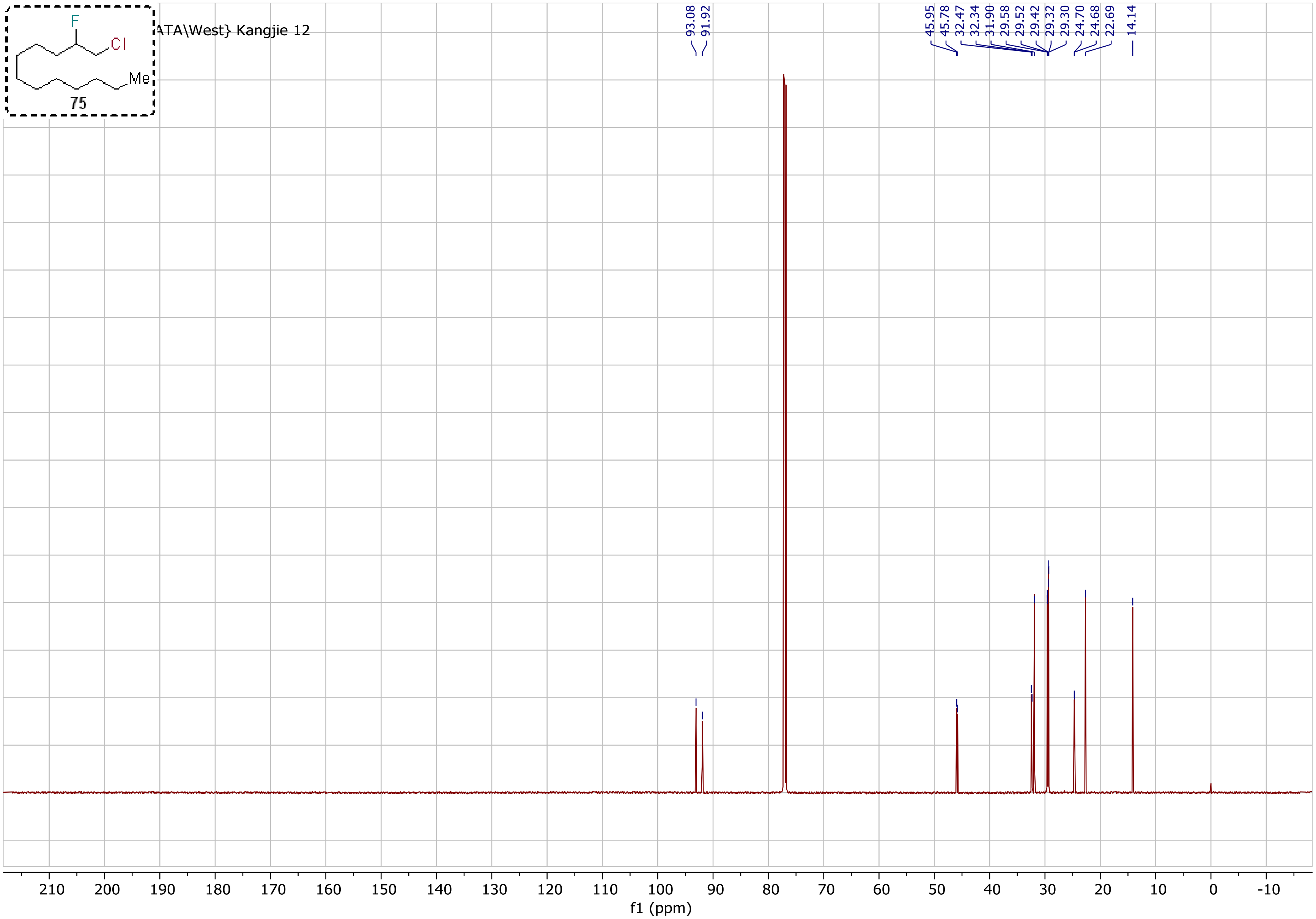
ATA\West} Kangjie 12
F
Cl
Me
75
93.08
91.92
45.95
45.78
32.47
32.34
31.90
29.58
29.52
29.42
29.32
29.30
24.70
24.68
22.69
14.14
f1 (ppm)

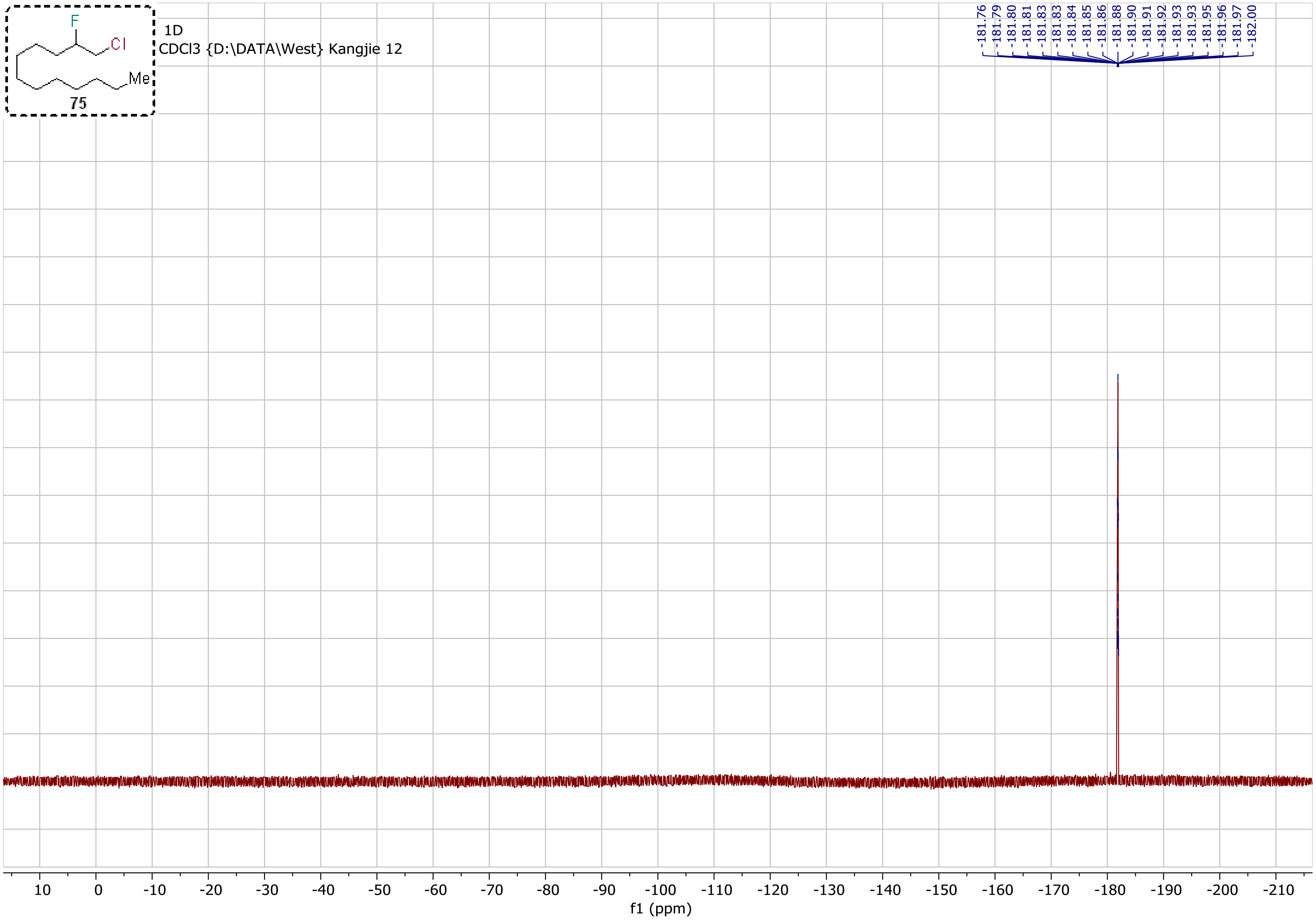
F
Cl
Me
75
1D
CDCl3 {D:\DATA\West} Kangjie 12
-181.76
-181.79
-181.80
-181.81
-181.83
-181.83
-181.84
-181.85
-181.86
-181.88
-181.90
-181.91
-181.92
-181.93
-181.93
-181.95
-181.96
-181.97
-182.00
10
0
-10
-20
-30
-40
-50
-60
-70
-80
-90
-100
-110
-120
-130
-140
-150
-160
-170
-180
-190
-200
-210
f1 (ppm)

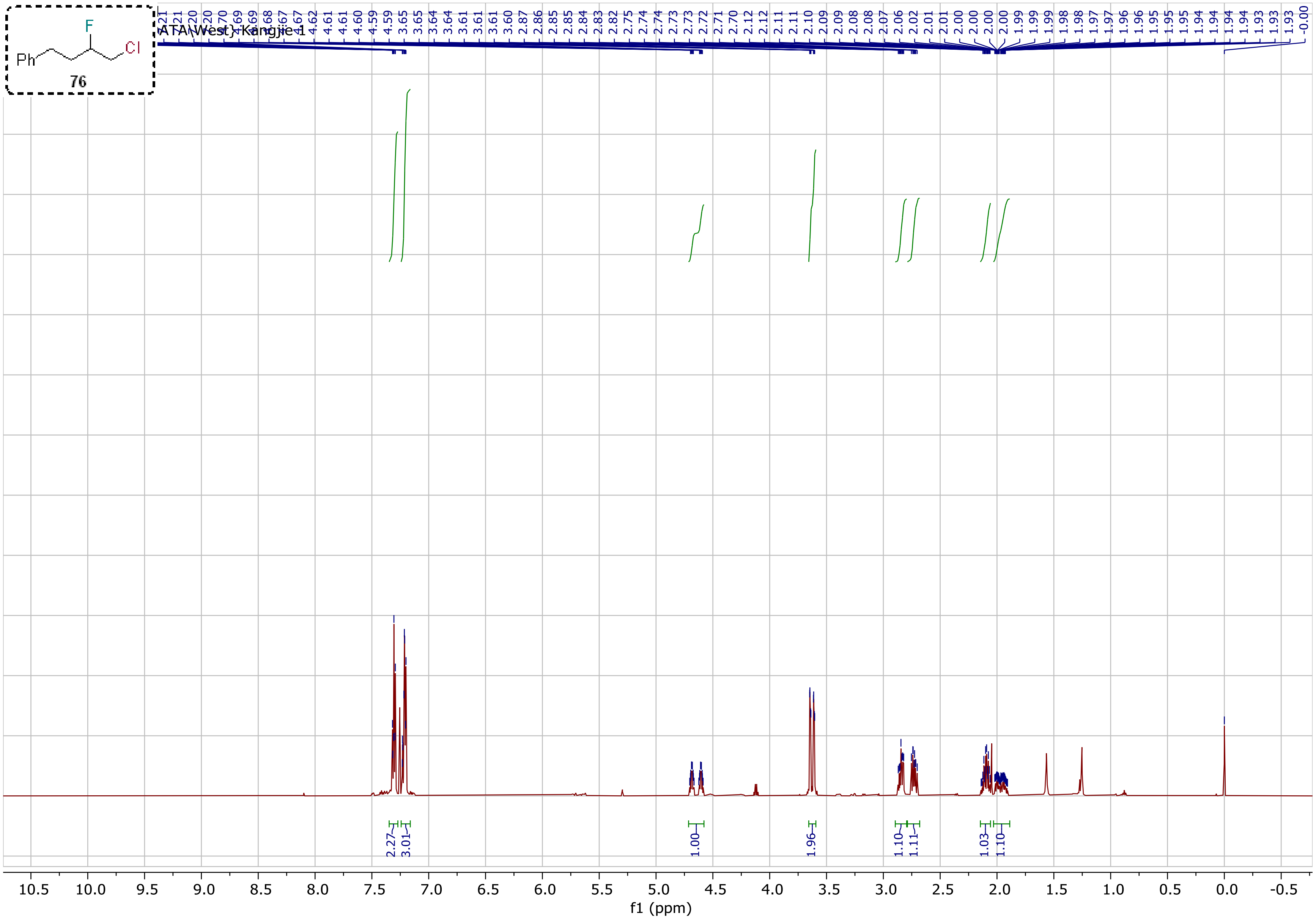
F
Ph
Cl
76
ATA(West)-Kangjie-1
4.62 4.61 4.61 4.60 4.59 4.59 3.65 3.65 3.64 3.64 3.61 3.61 3.61 3.60 2.87 2.86 2.85 2.85 2.84 2.83 2.82 2.75 2.74 2.74 2.73 2.73 2.72 2.71 2.70 2.12 2.12 2.11 2.11 2.10 2.09 2.09 2.08 2.08 2.07 2.06 2.02 2.01 2.01 2.00 2.00 2.00 2.00 1.99 1.99 1.99 1.98 1.98 1.97 1.97 1.96 1.96 1.95 1.95 1.95 1.94 1.94 1.94 1.94 1.93 1.93 -0.00
2.27
3.01
1.00
1.96
1.10
1.11
1.03
1.10
10.5 10.0 9.5 9.0 8.5 8.0 7.5 7.0 6.5 6.0 5.5 5.0 4.5 4.0 3.5 3.0 2.5 2.0 1.5 1.0 0.5 0.0 -0.5
f1 (ppm)

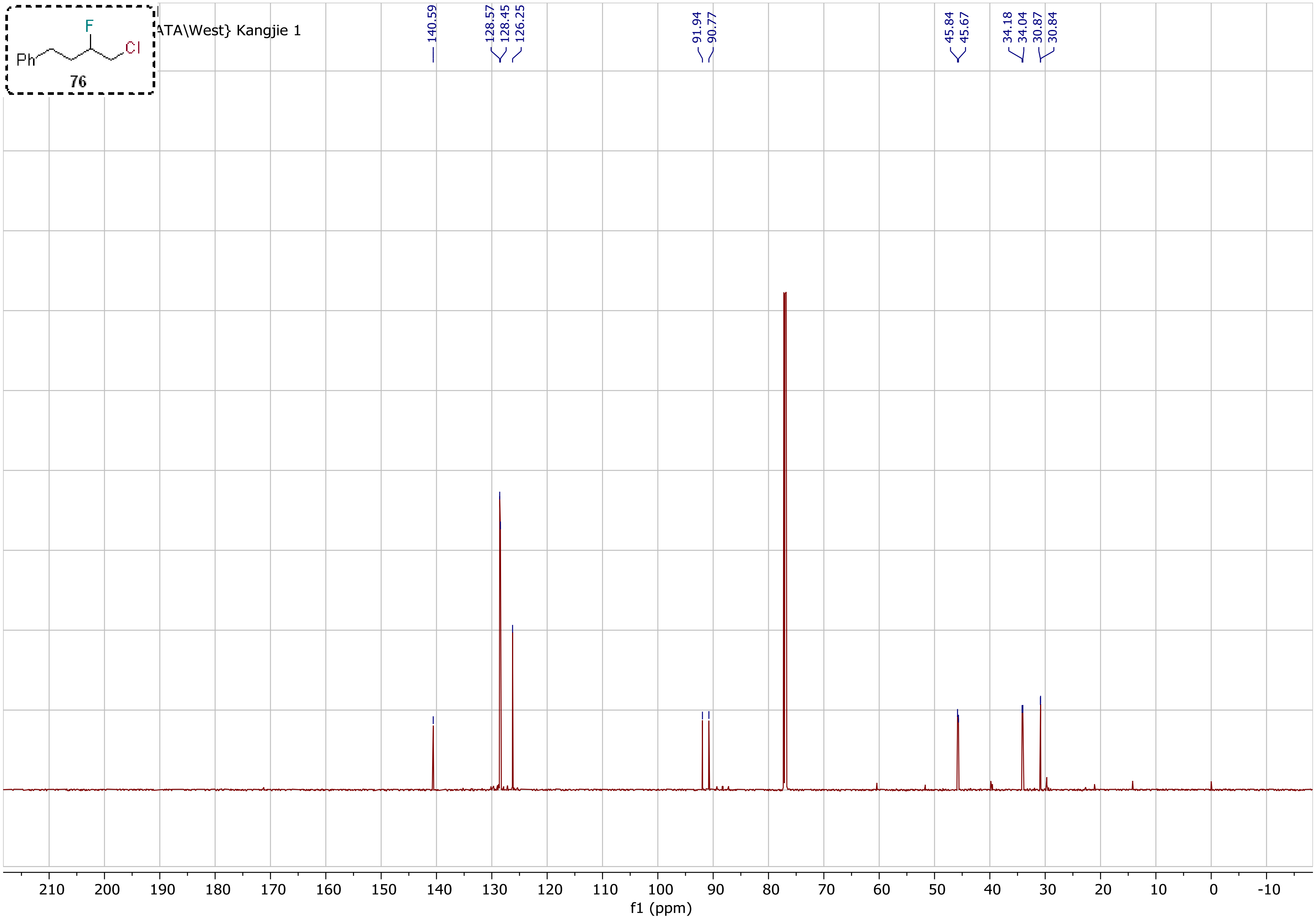
F
Cl
Ph
76
ATA\West} Kangjie 1
140.59
128.57
128.45
126.25
91.94
90.77
45.84
45.67
34.18
34.04
30.87
30.84
f1 (ppm)

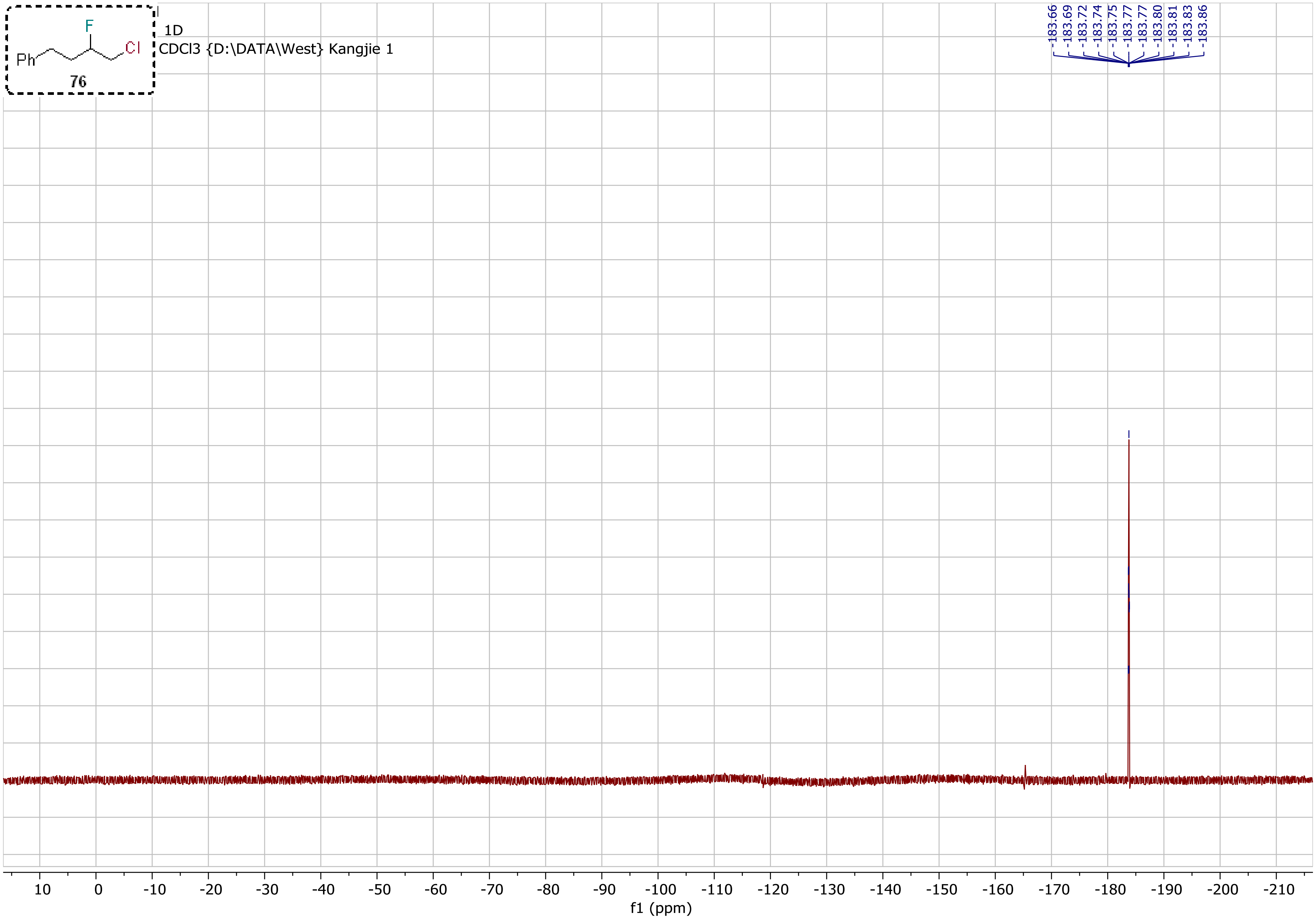
F
Ph
Cl
76
1D
CDCl3 {D:\DATA\West} Kangjie 1
-183.66
-183.69
-183.72
-183.74
-183.75
-183.77
-183.77
-183.80
-183.81
-183.83
-183.86
10
0
-10
-20
-30
-40
-50
-60
-70
-80
-90
-100
-110
-120
-130
-140
-150
-160
-170
-180
-190
-200
-210
f1 (ppm)

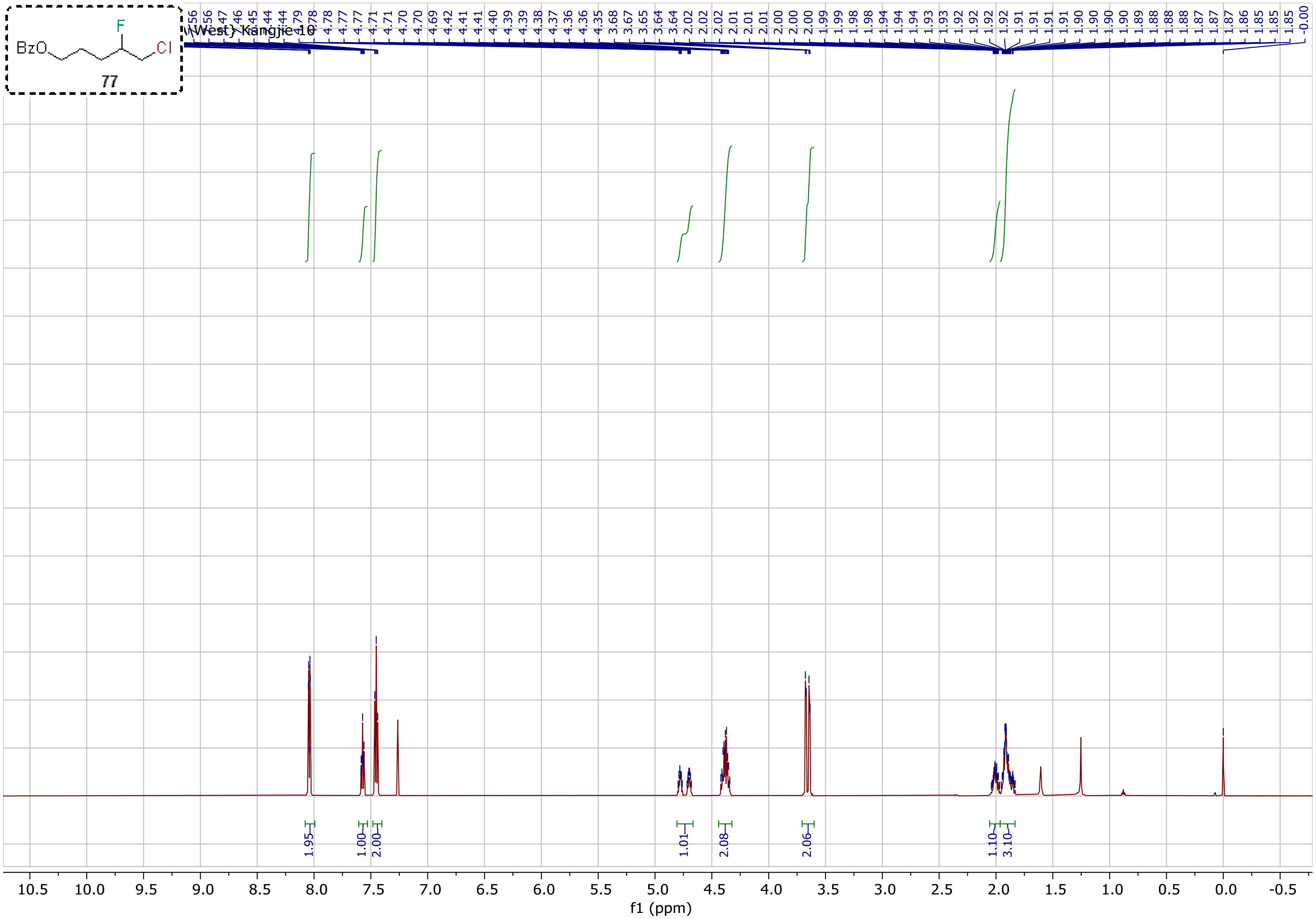

BzO
F
Cl
77
1.95
1.00
2.00
1.01
2.08
2.06
1.10
3.10
10.5 10.0 9.5 9.0 8.5 8.0 7.5 7.0 6.5 6.0 5.5 5.0 4.5 4.0 3.5 3.0 2.5 2.0 1.5 1.0 0.5 0.0 -0.5
f1 (ppm)

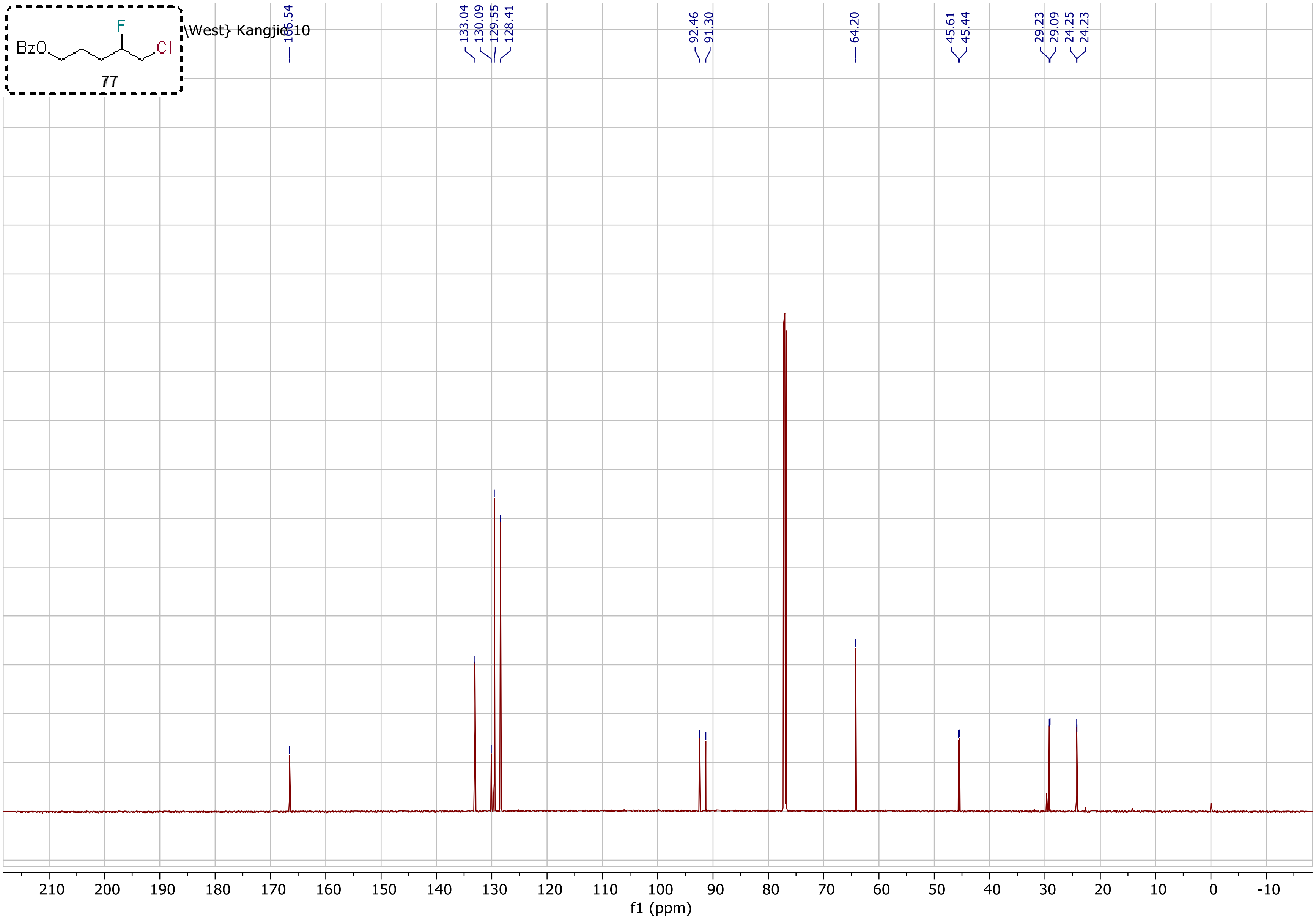
BzO
F
Cl
77
\West} Kangjie610
166.54
133.04
130.09
129.55
128.41
92.46
91.30
64.20
45.61
45.44
29.23
29.09
24.25
24.23
210 200 190 180 170 160 150 140 130 120 110 100 90 80 70 60 50 40 30 20 10 0 -10
f1 (ppm)

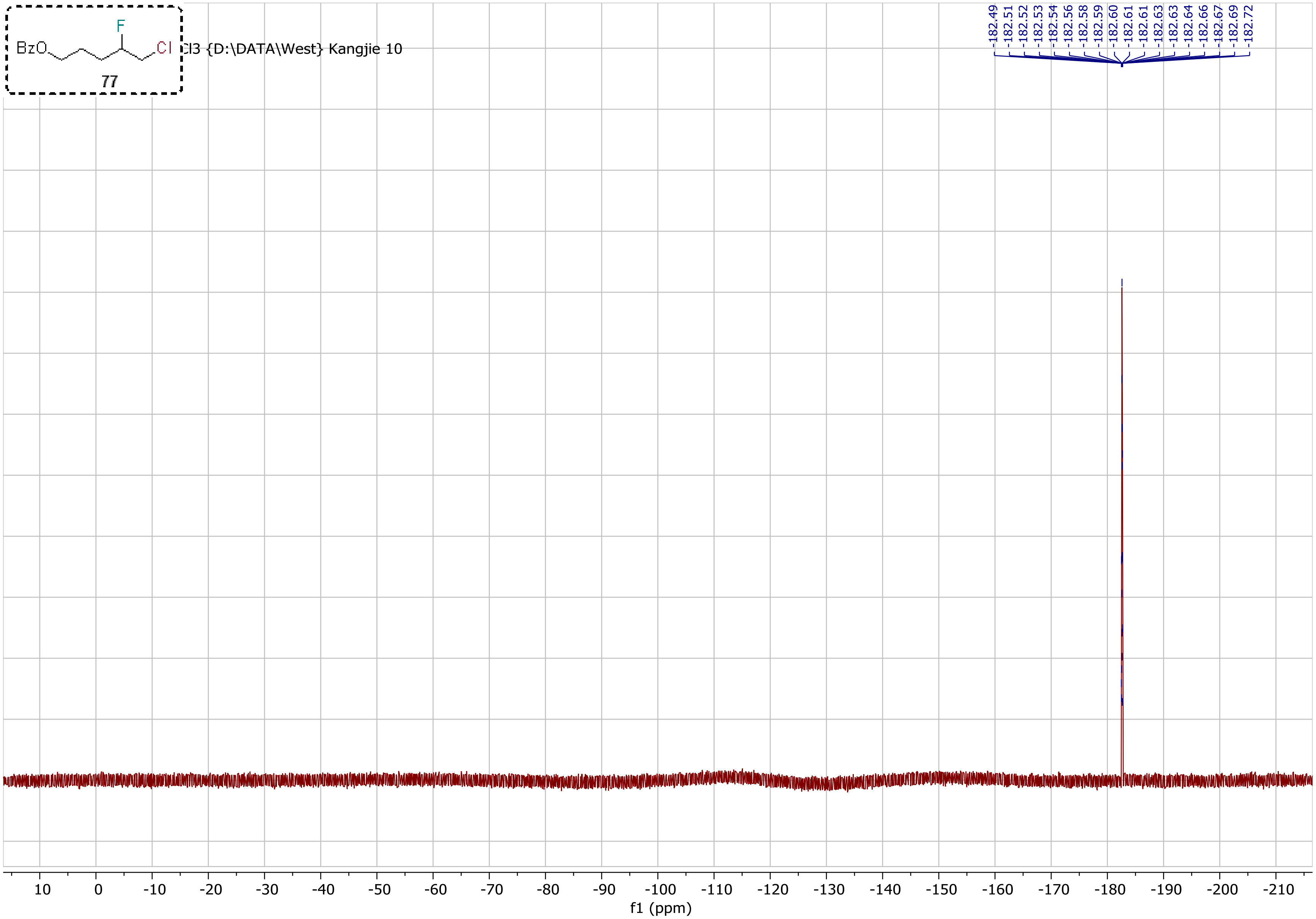
BzO
F
Cl
77
Cl3 {D:\DATA\West} Kangjie 10
-182.49
-182.51
-182.52
-182.53
-182.54
-182.56
-182.58
-182.59
-182.60
-182.61
-182.61
-182.63
-182.63
-182.64
-182.66
-182.67
-182.69
-182.72
10 0 -10 -20 -30 -40 -50 -60 -70 -80 -90 -100 -110 -120 -130 -140 -150 -160 -170 -180 -190 -200 -210
f1 (ppm)

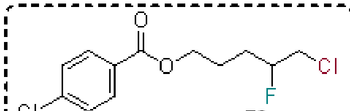

78

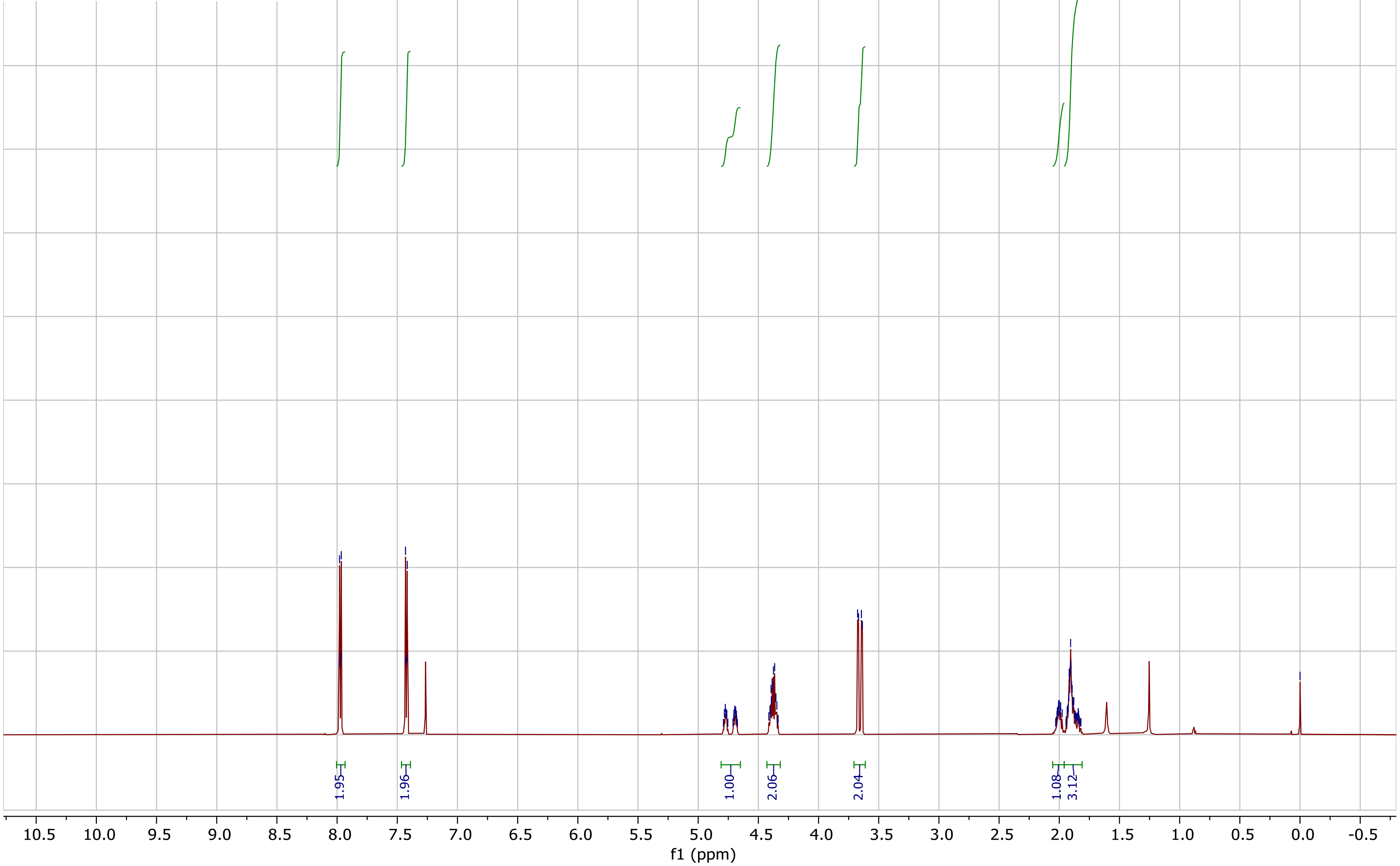

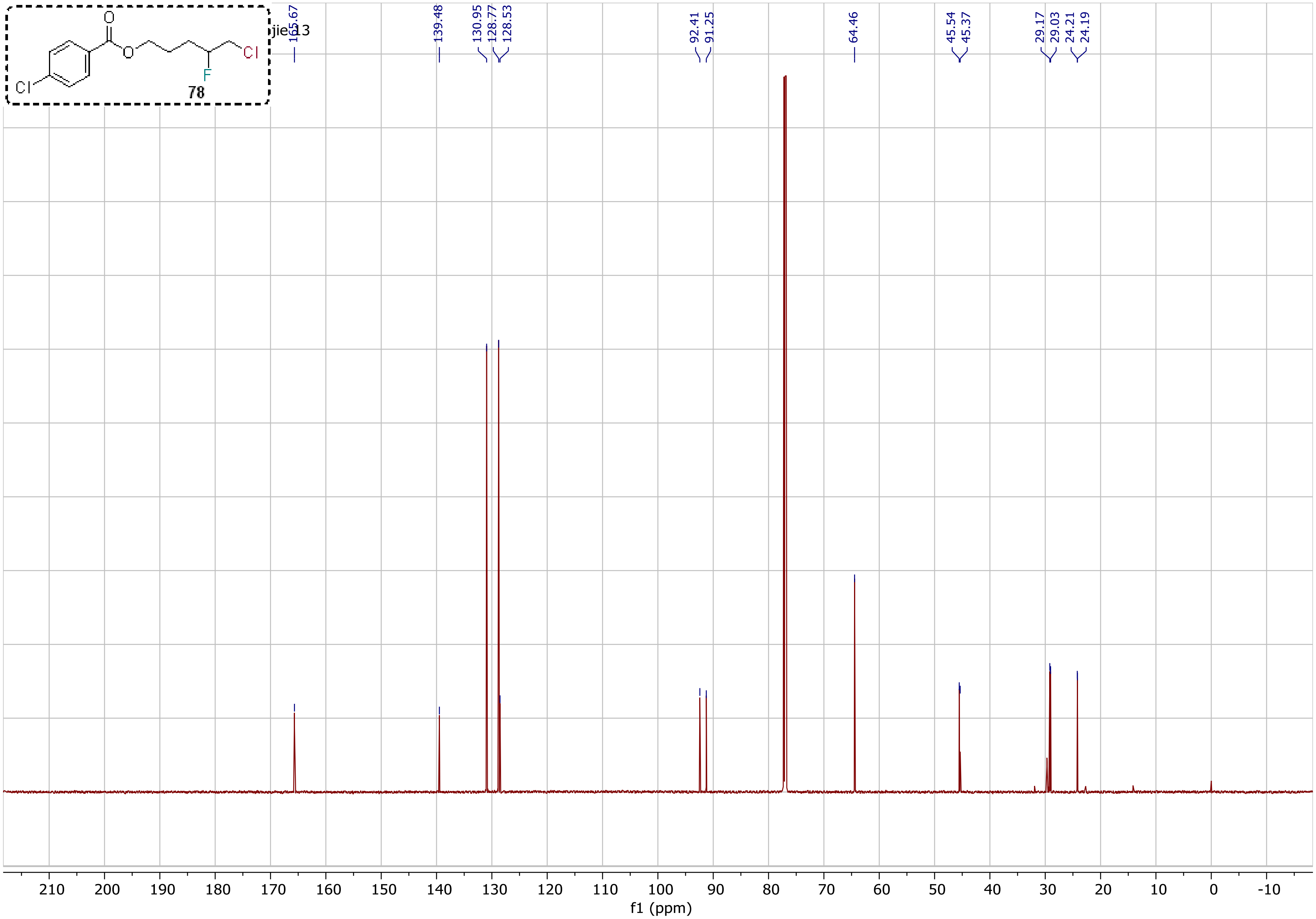

jie13
Cl
O
O
F
Cl
78
165.67
139.48
130.95
128.77
128.53
92.41
91.25
64.46
45.54
45.37
29.17
29.03
24.21
24.19
210
200
190
180
170
160
150
140
130
120
110
100
90
80
70
60
50
40
30
20
10
0
-10
f1 (ppm)

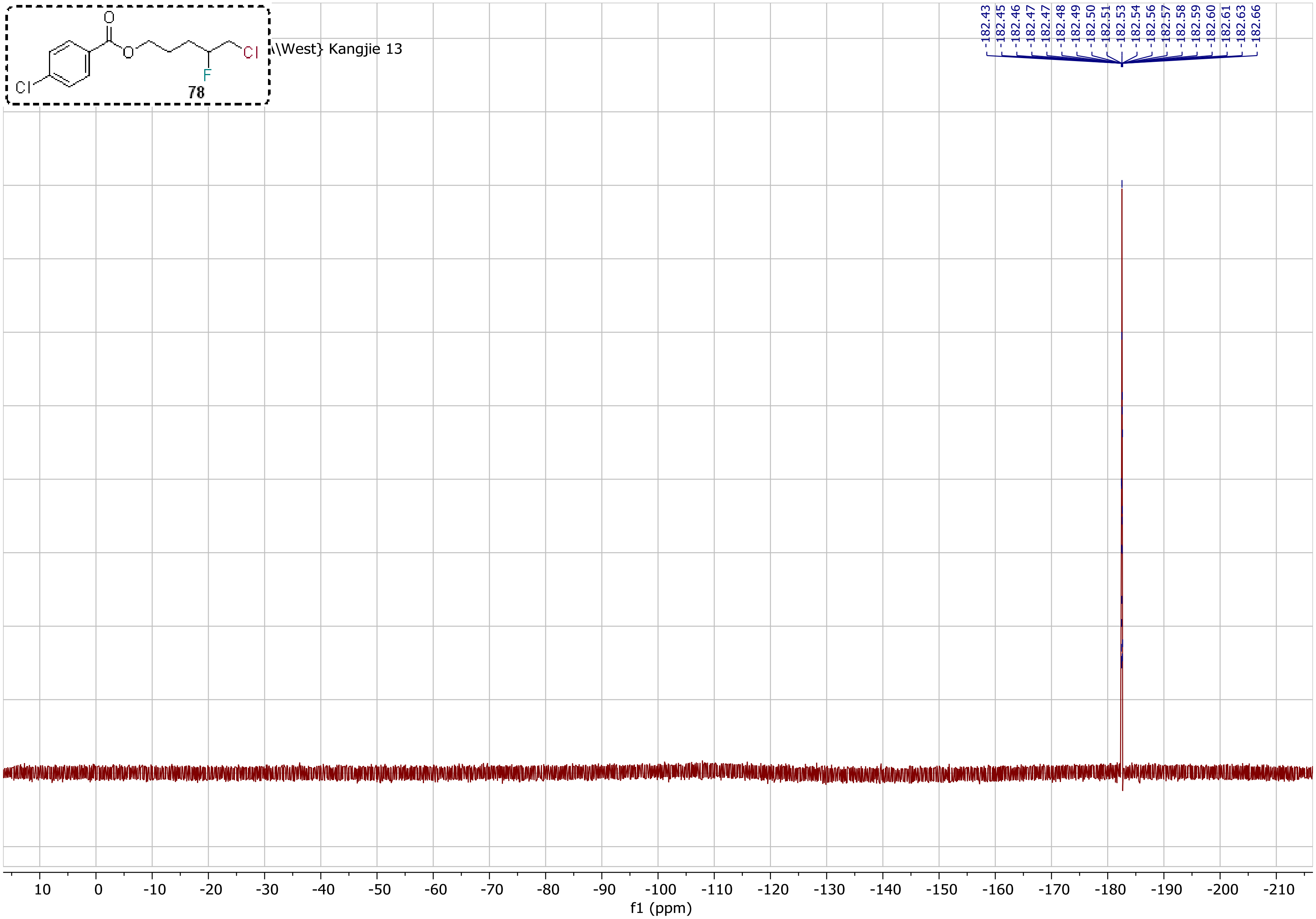

78
\\West} Kangjie 13
-182.43
-182.45
-182.46
-182.47
-182.47
-182.48
-182.49
-182.50
-182.51
-182.53
-182.54
-182.56
-182.57
-182.58
-182.59
-182.60
-182.61
-182.63
-182.66
f1 (ppm)

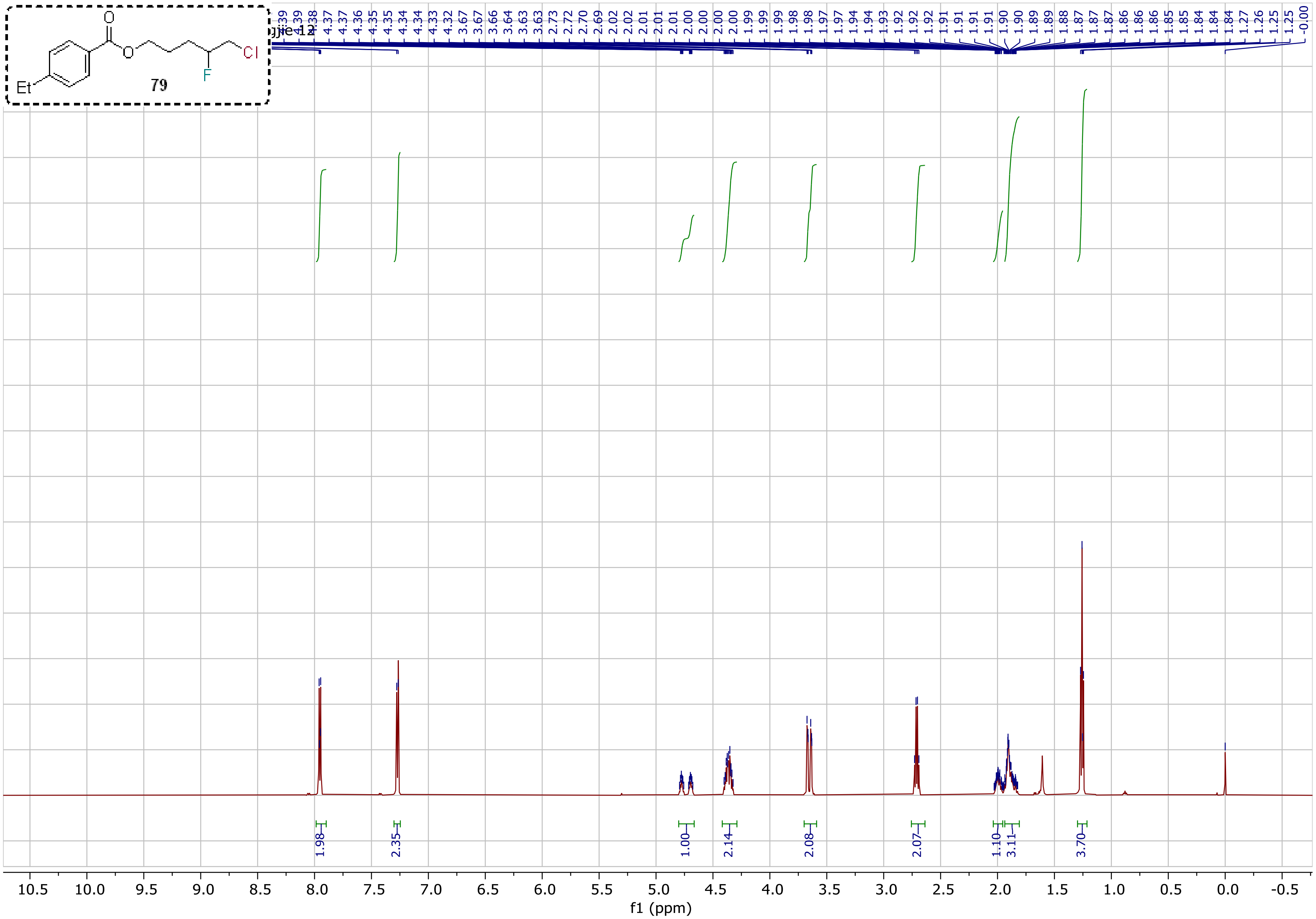
79
Et
O
F
Cl
f1 (ppm)
1.98
2.35
1.00
2.14
2.08
2.07
1.10
3.11
3.70

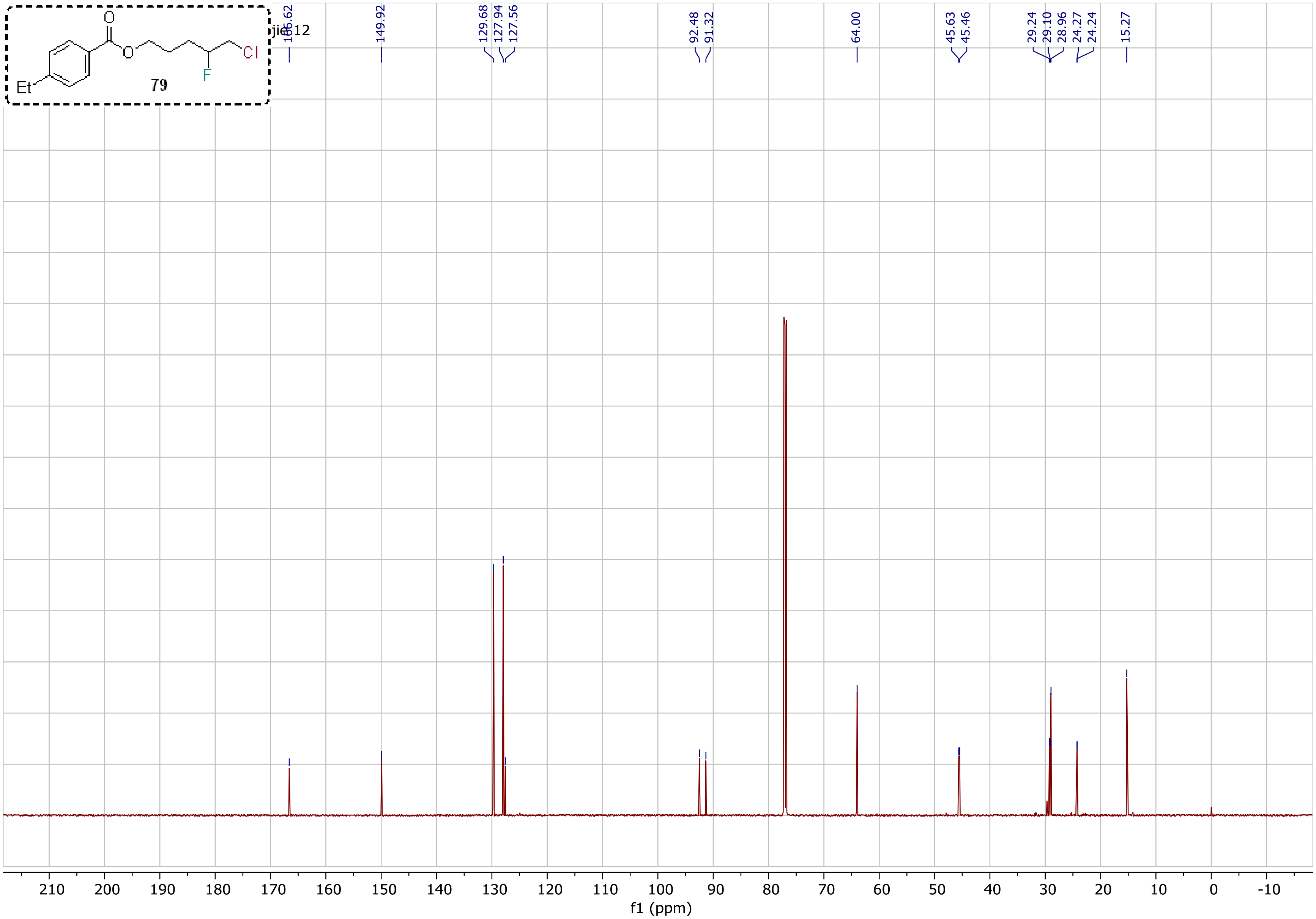

O
O
F
Cl
Et
79
jie12
166.62
149.92
129.68
127.94
127.56
92.48
91.32
64.00
45.63
45.46
29.24
29.10
28.96
24.27
24.24
15.27
210
200
190
180
170
160
150
140
130
120
110
100
90
80
70
60
50
40
30
20
10
0
-10
f1 (ppm)

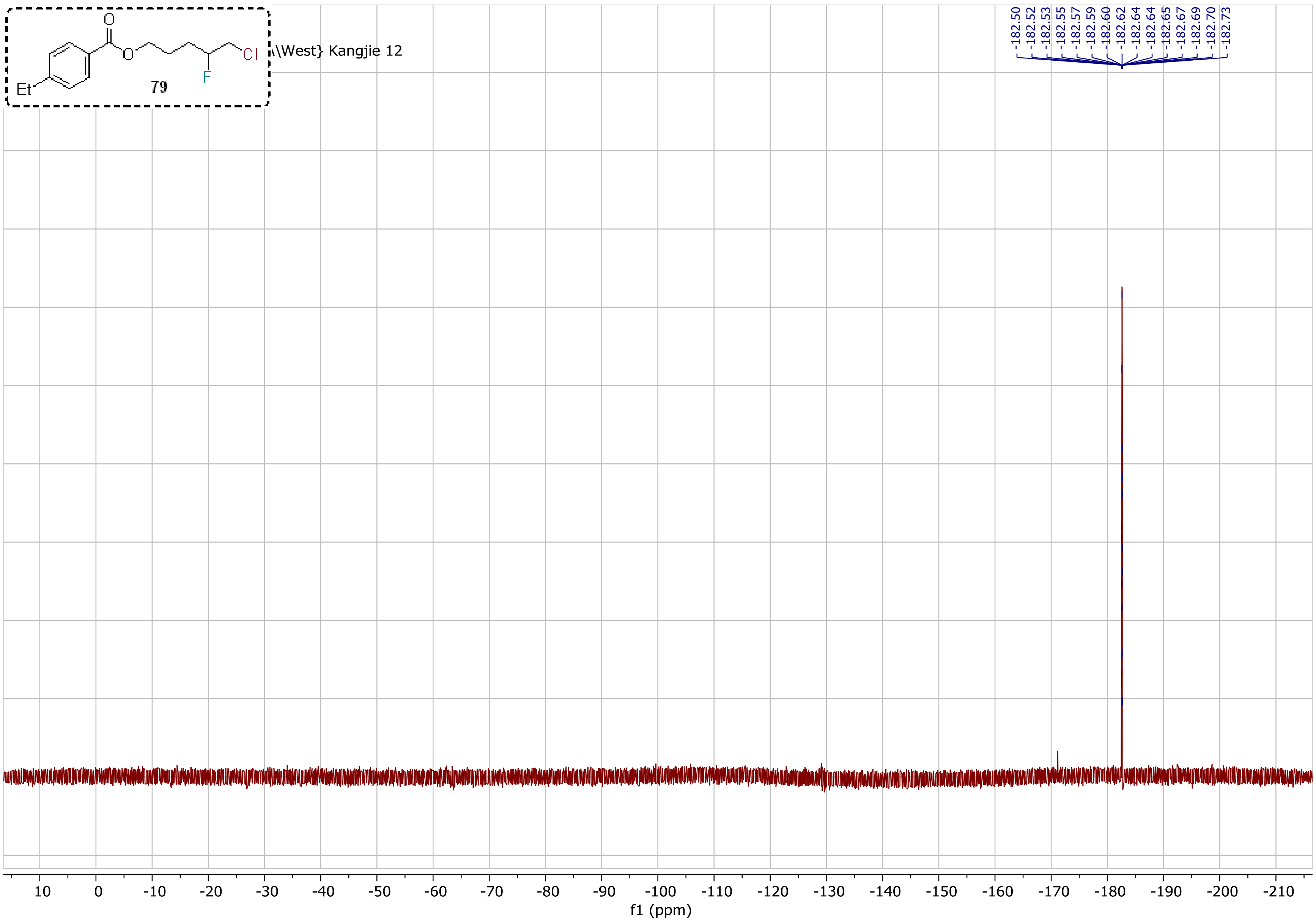
Et
O
O
F
Cl
79
\\West} Kangjie 12
-182.50
-182.52
-182.53
-182.55
-182.57
-182.59
-182.60
-182.62
-182.64
-182.64
-182.65
-182.67
-182.69
-182.70
-182.73
10 0 -10 -20 -30 -40 -50 -60 -70 -80 -90 -100 -110 -120 -130 -140 -150 -160 -170 -180 -190 -200 -210
f1 (ppm)

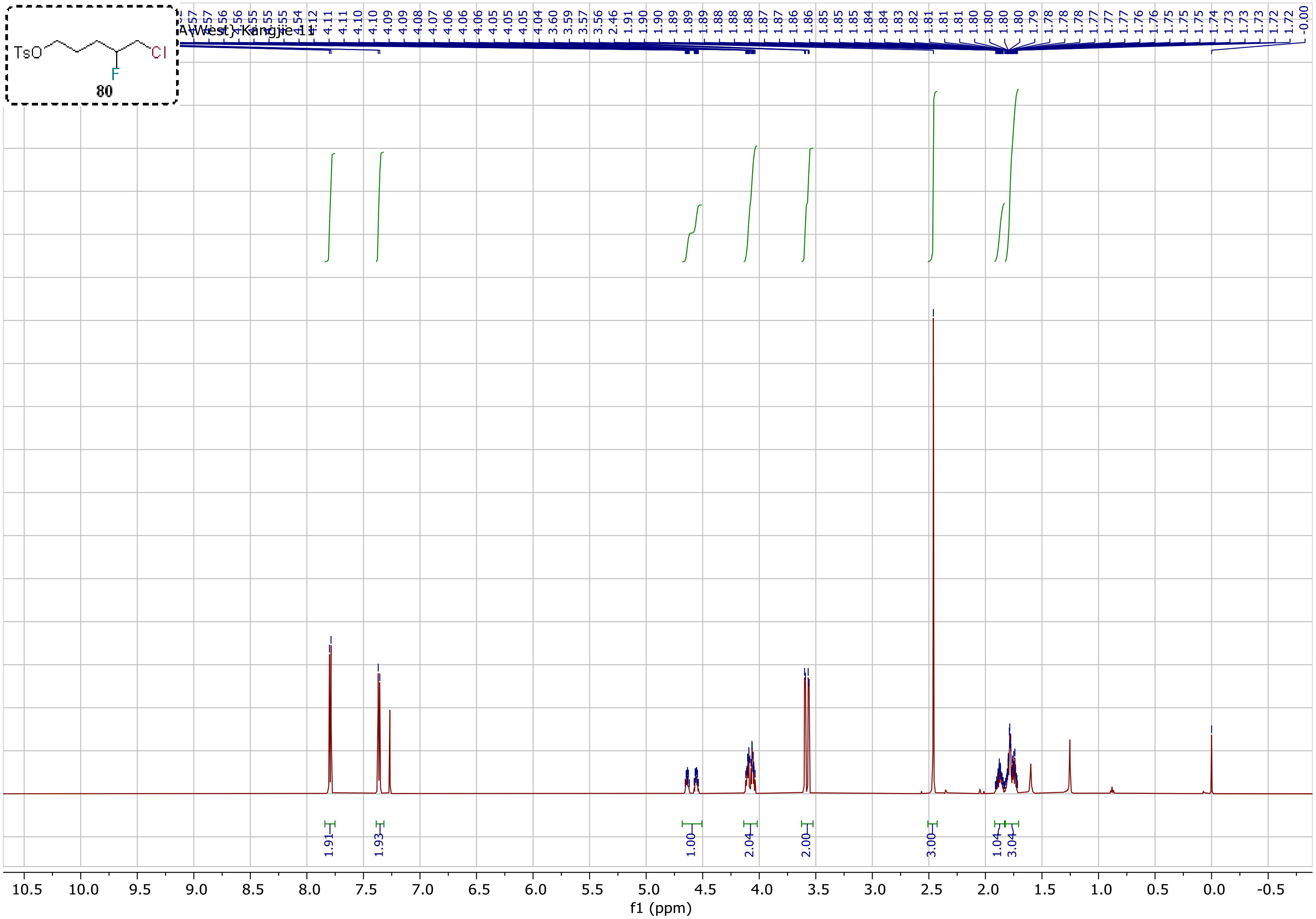
TsO
F
Cl
80
f1 (ppm)

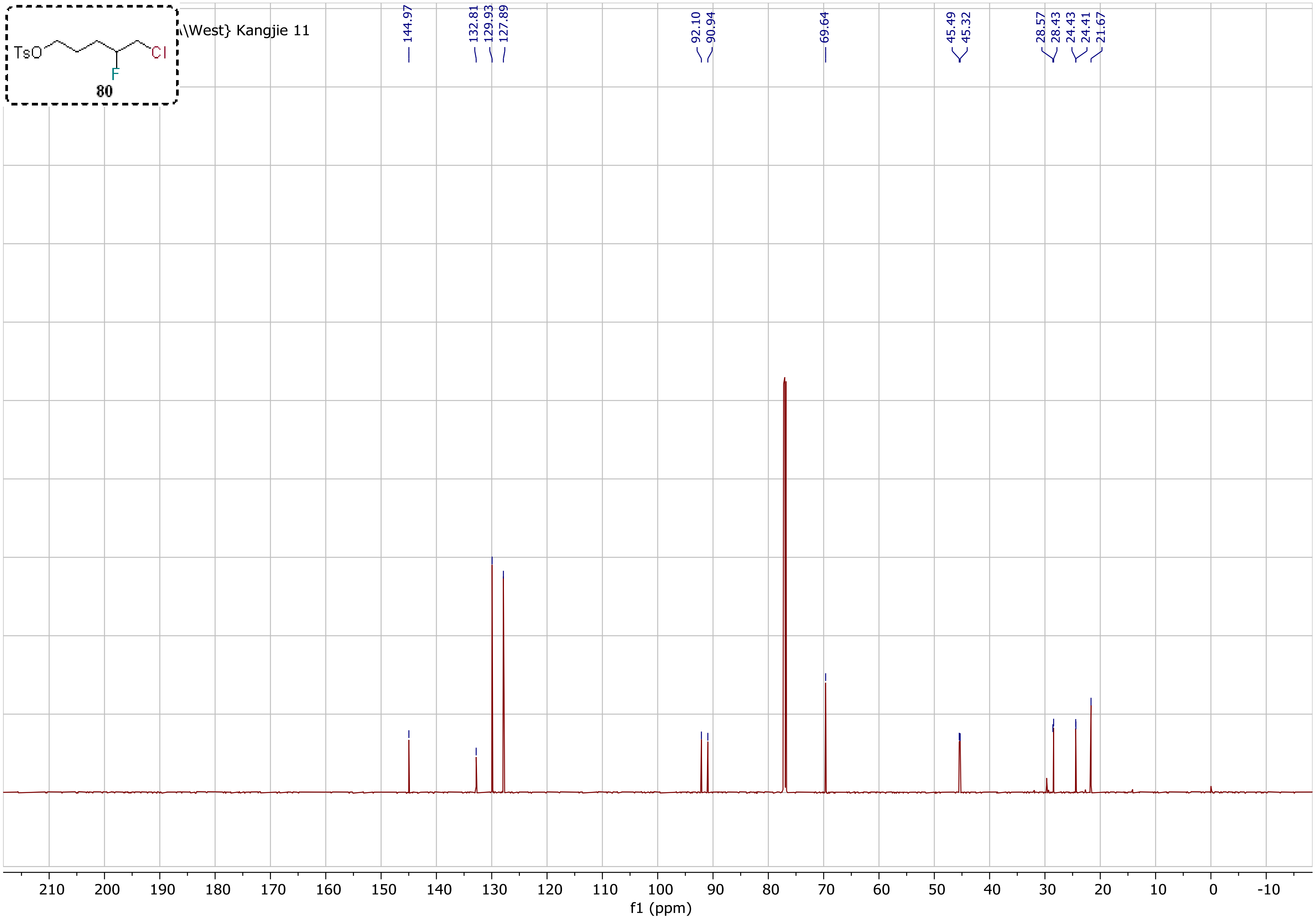
TsO
F
Cl
80
\West} Kangjie 11
144.97
132.81
129.93
127.89
92.10
90.94
69.64
45.49
45.32
28.57
28.43
24.43
24.41
21.67
210 200 190 180 170 160 150 140 130 120 110 100 90 80 70 60 50 40 30 20 10 0 -10
f1 (ppm)

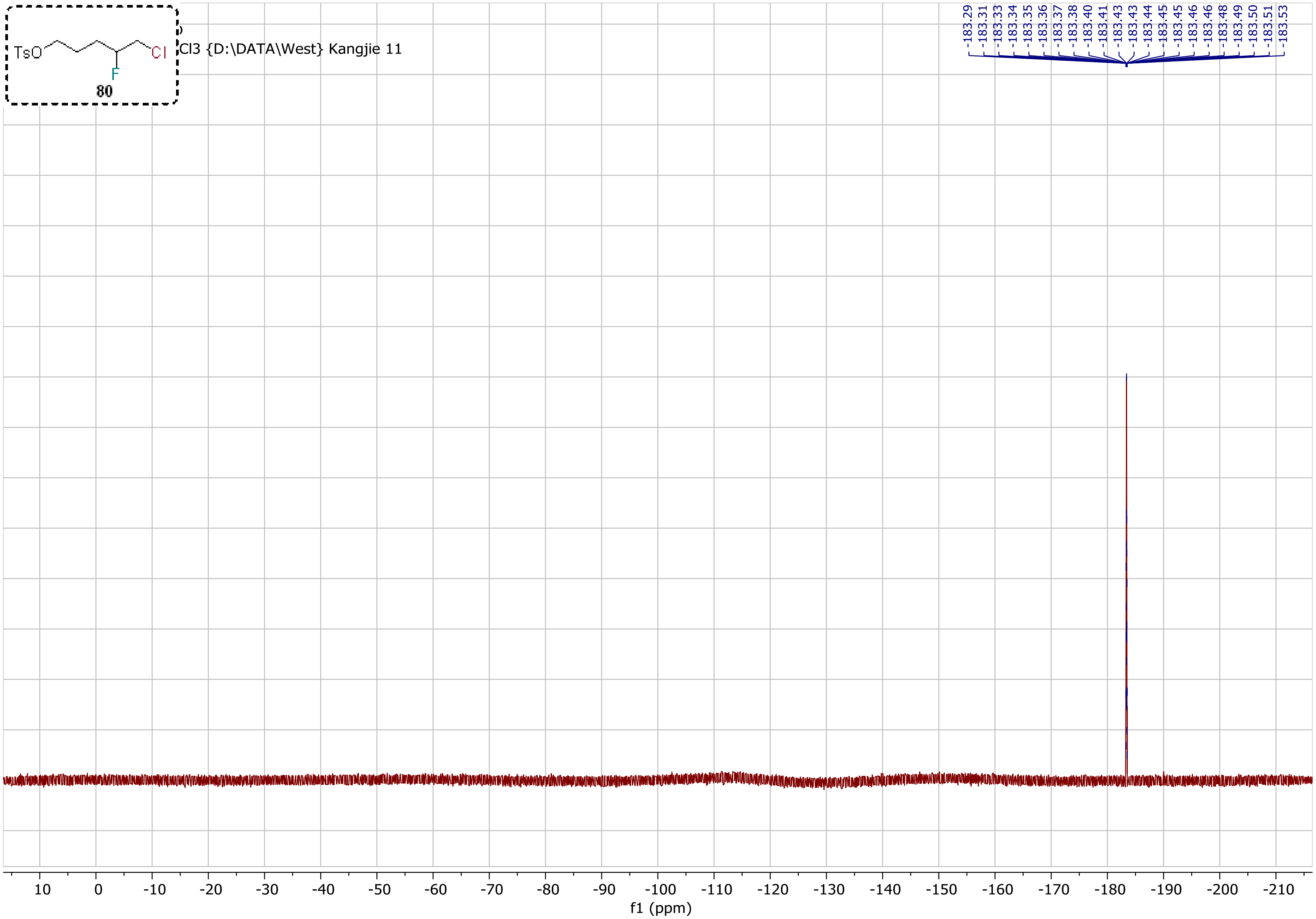
TsO
F
Cl
80
Cl3 {D:\DATA\West} Kangjie 11
-183.29
-183.31
-183.33
-183.34
-183.35
-183.36
-183.37
-183.38
-183.40
-183.41
-183.43
-183.43
-183.44
-183.45
-183.45
-183.46
-183.46
-183.48
-183.49
-183.50
-183.51
-183.53
10 0 -10 -20 -30 -40 -50 -60 -70 -80 -90 -100 -110 -120 -130 -140 -150 -160 -170 -180 -190 -200 -210
f1 (ppm)

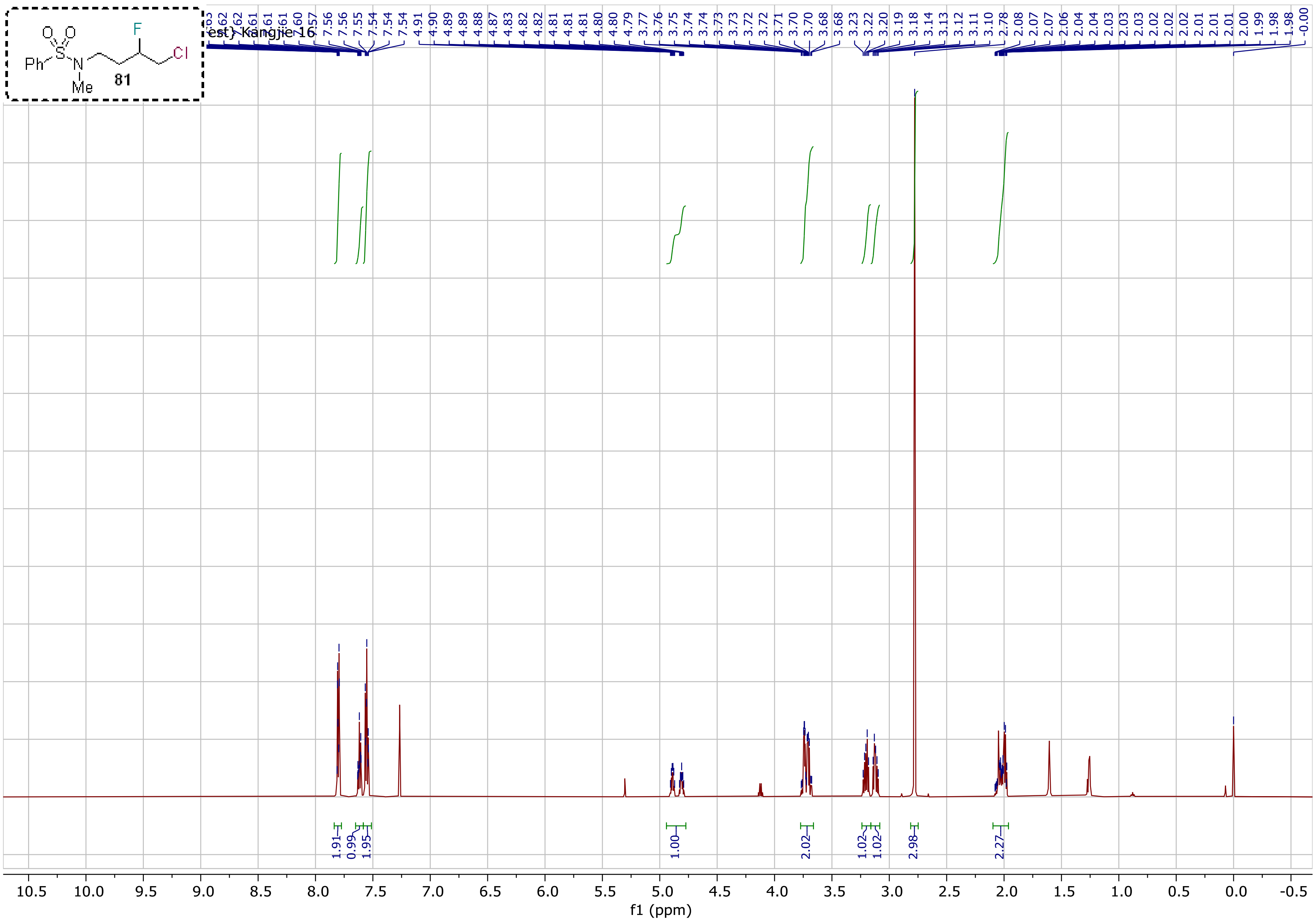

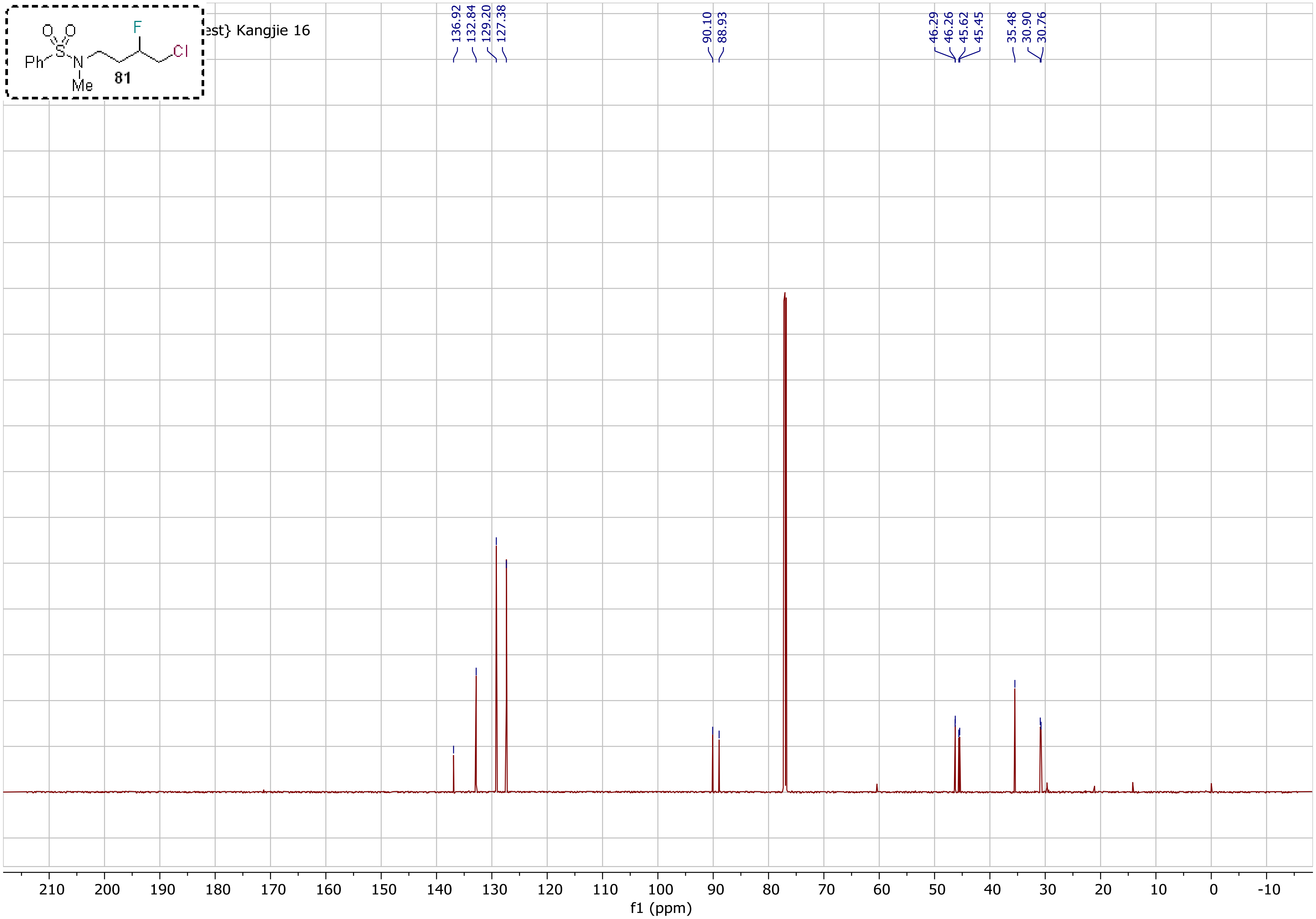
est} Kangjie 16
Ph
S
O O
N
Me
F
Cl
81
136.92
132.84
129.20
127.38
90.10
88.93
46.29
46.26
45.62
45.45
35.48
30.90
30.76
210 200 190 180 170 160 150 140 130 120 110 100 90 80 70 60 50 40 30 20 10 0 -10
f1 (ppm)

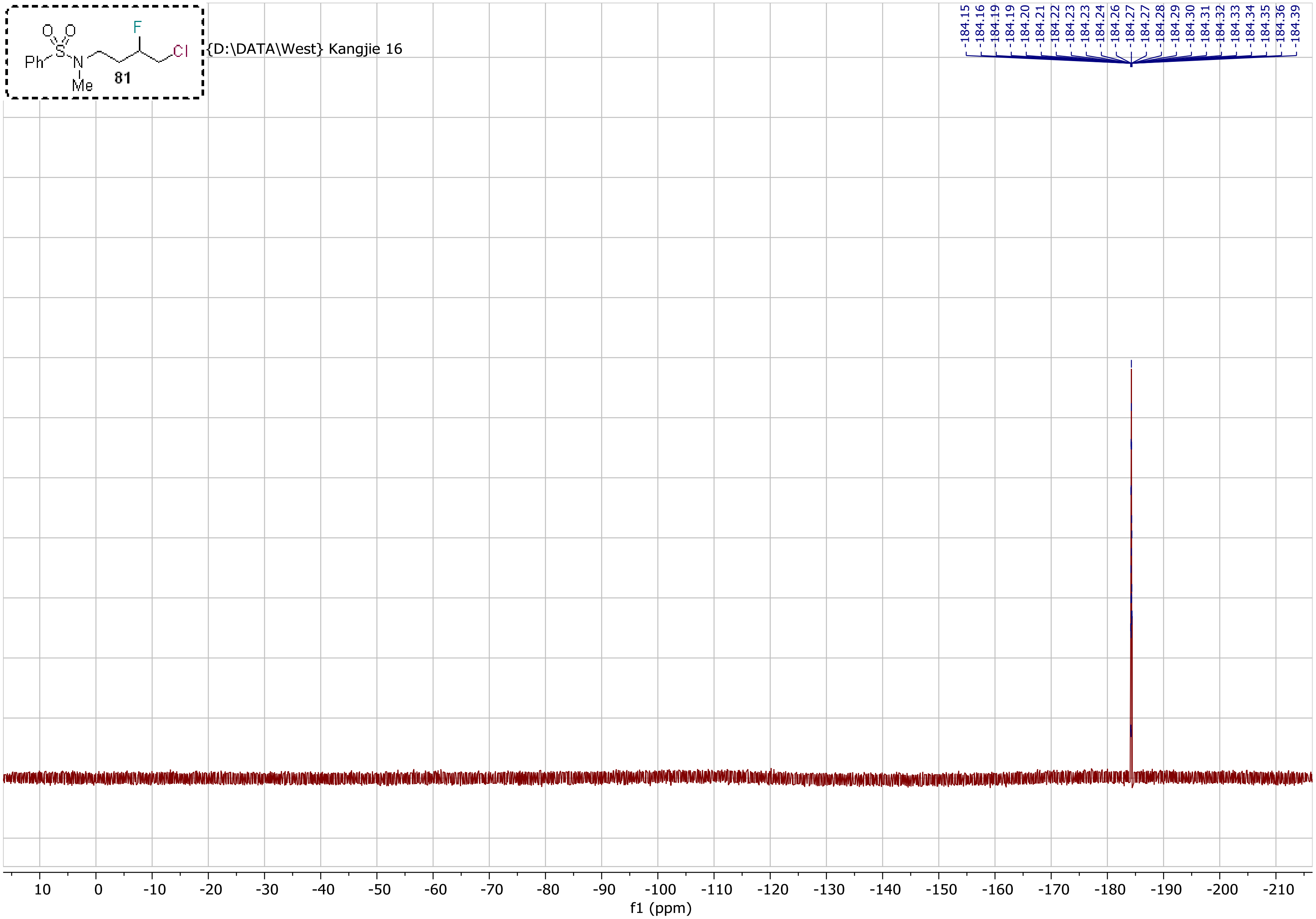

Ph
S
O O
N
Me
F
Cl
81
{D:\DATA\West} Kangjie 16
-184.15
-184.16
-184.19
-184.19
-184.20
-184.21
-184.22
-184.23
-184.23
-184.24
-184.26
-184.27
-184.27
-184.28
-184.29
-184.30
-184.31
-184.32
-184.33
-184.34
-184.35
-184.36
-184.39
10 0 -10 -20 -30 -40 -50 -60 -70 -80 -90 -100 -110 -120 -130 -140 -150 -160 -170 -180 -190 -200 -210
f1 (ppm)

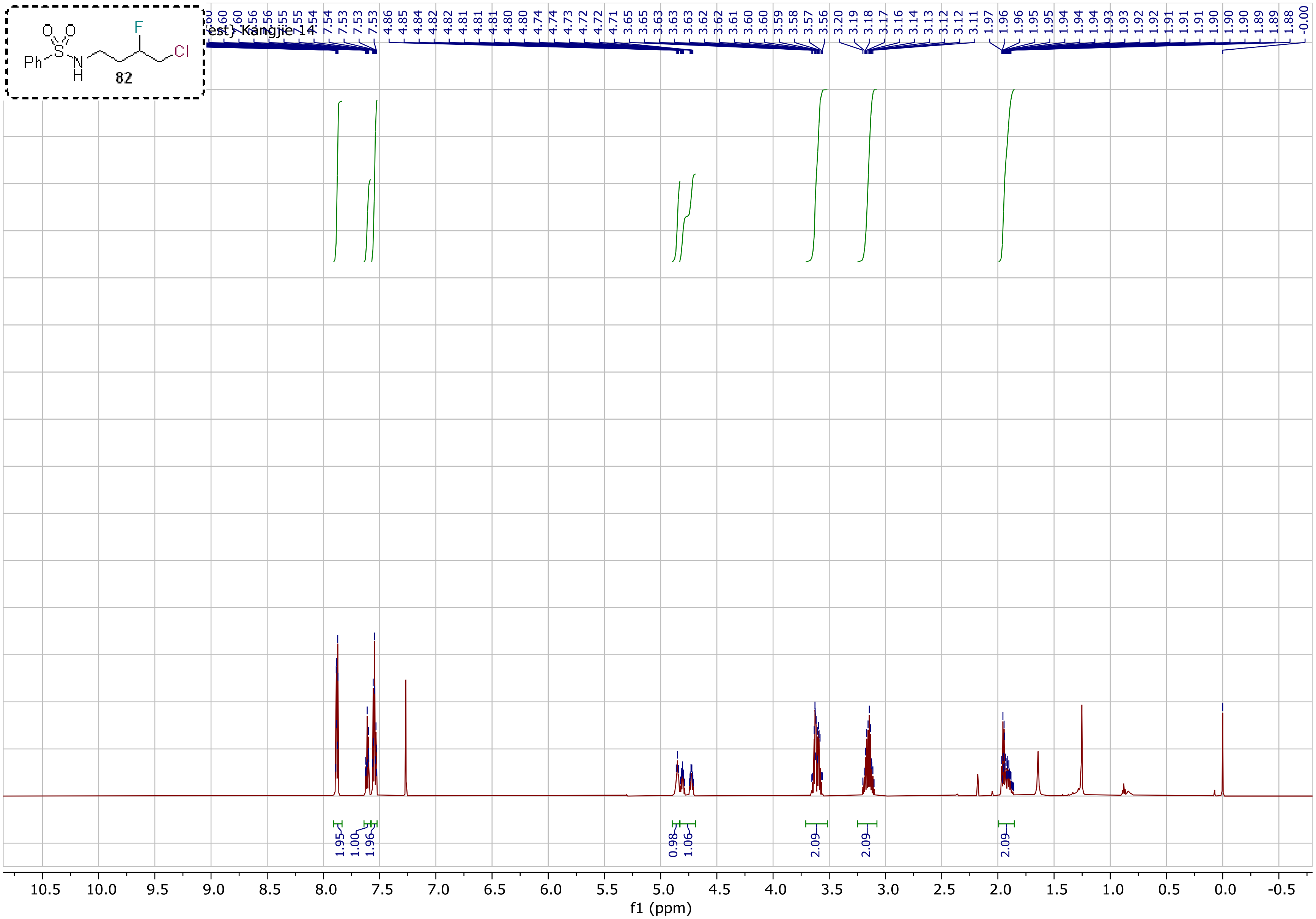

82
est} Kangjie 14
f1 (ppm)

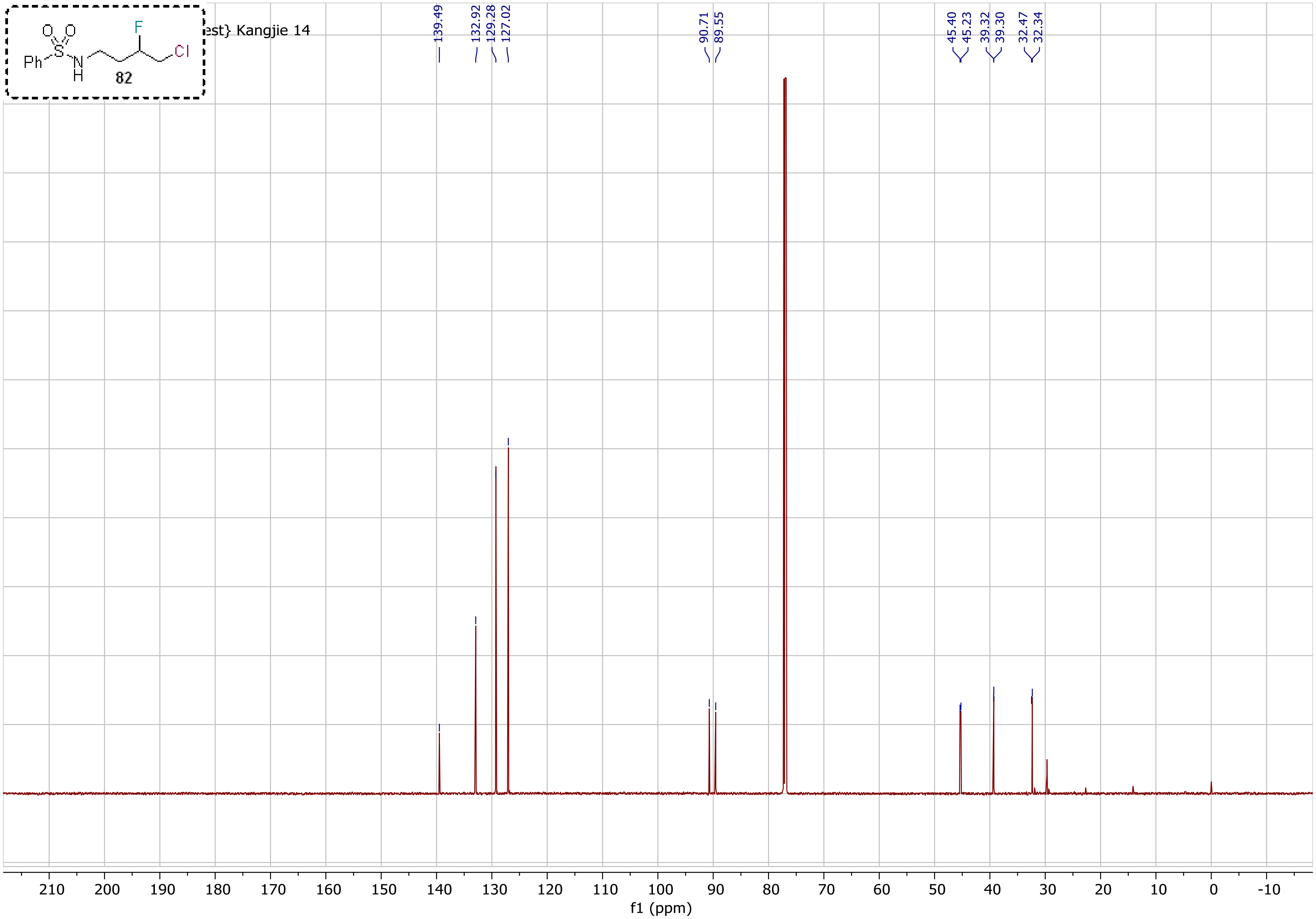
82
est} Kangjie 14
139.49
132.92
129.28
127.02
90.71
89.55
45.40
45.23
39.32
39.30
32.47
32.34
210
200
190
180
170
160
150
140
130
120
110
100
90
80
70
60
50
40
30
20
10
0
-10
f1 (ppm)

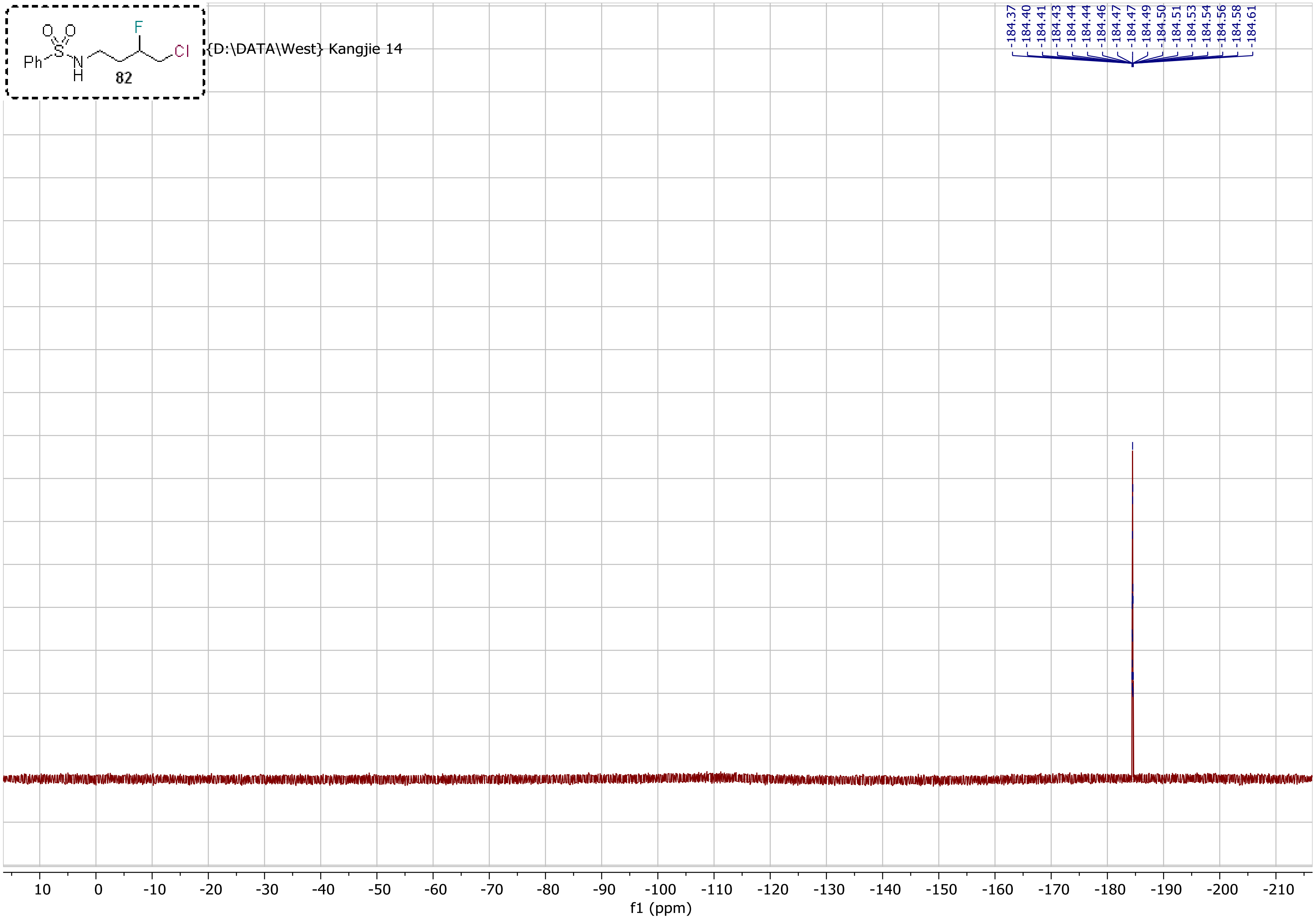

82
{D:\DATA\West} Kangjie 14
-184.37
-184.40
-184.41
-184.43
-184.44
-184.44
-184.46
-184.47
-184.47
-184.49
-184.50
-184.51
-184.53
-184.54
-184.56
-184.58
-184.61
f1 (ppm)

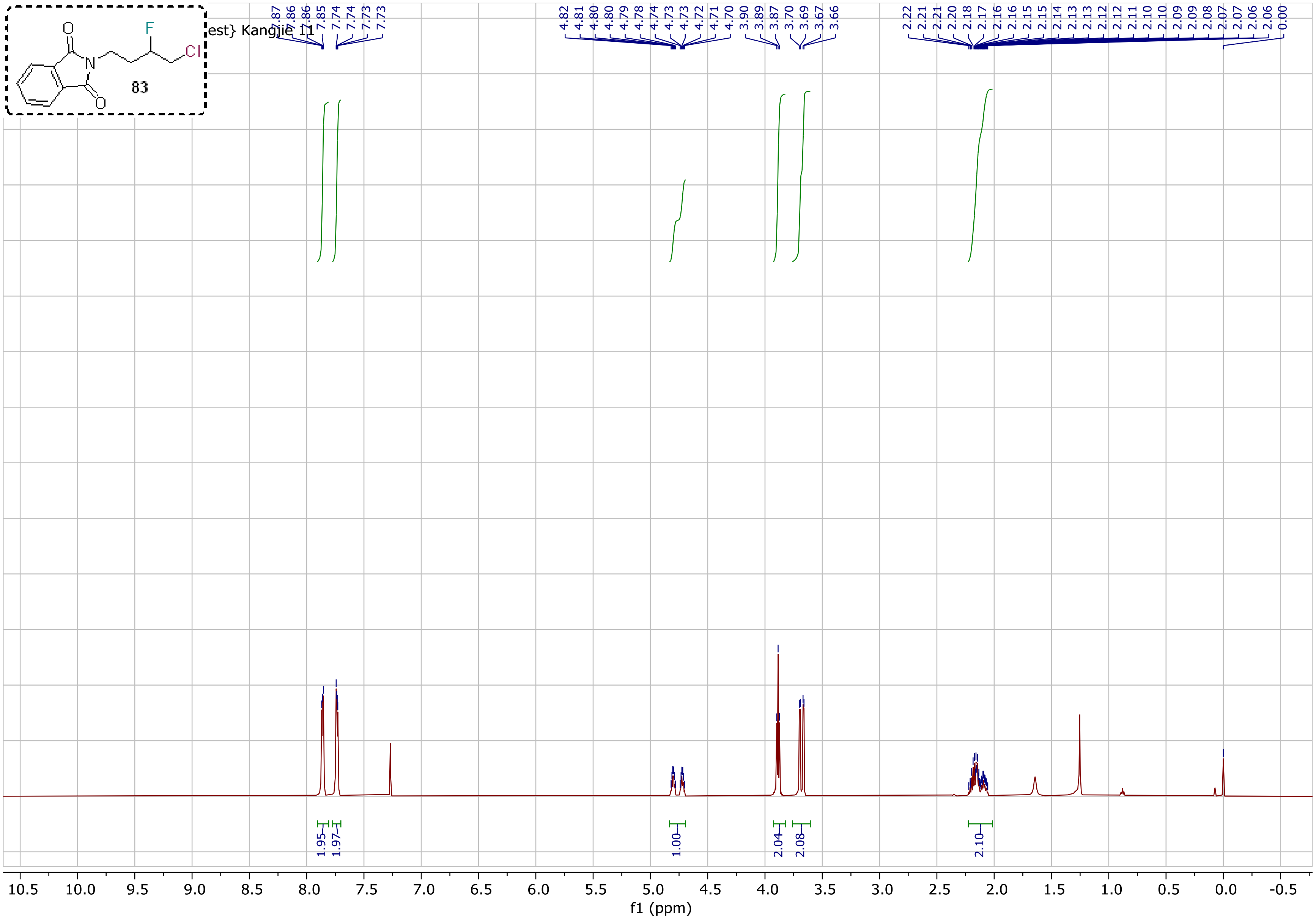
est} Kangjie 11
83
7.87
7.86
7.86
7.85
7.74
7.74
7.73
7.73
4.82
4.81
4.80
4.80
4.79
4.78
4.74
4.73
4.73
4.72
4.71
4.70
3.90
3.89
3.87
3.70
3.69
3.67
3.66
2.22
2.21
2.21
2.20
2.18
2.17
2.16
2.16
2.15
2.15
2.14
2.13
2.13
2.12
2.12
2.11
2.10
2.10
2.09
2.09
2.08
2.07
2.07
2.06
2.06
0.00
1.95
1.97
1.00
2.04
2.08
2.10
f1 (ppm)

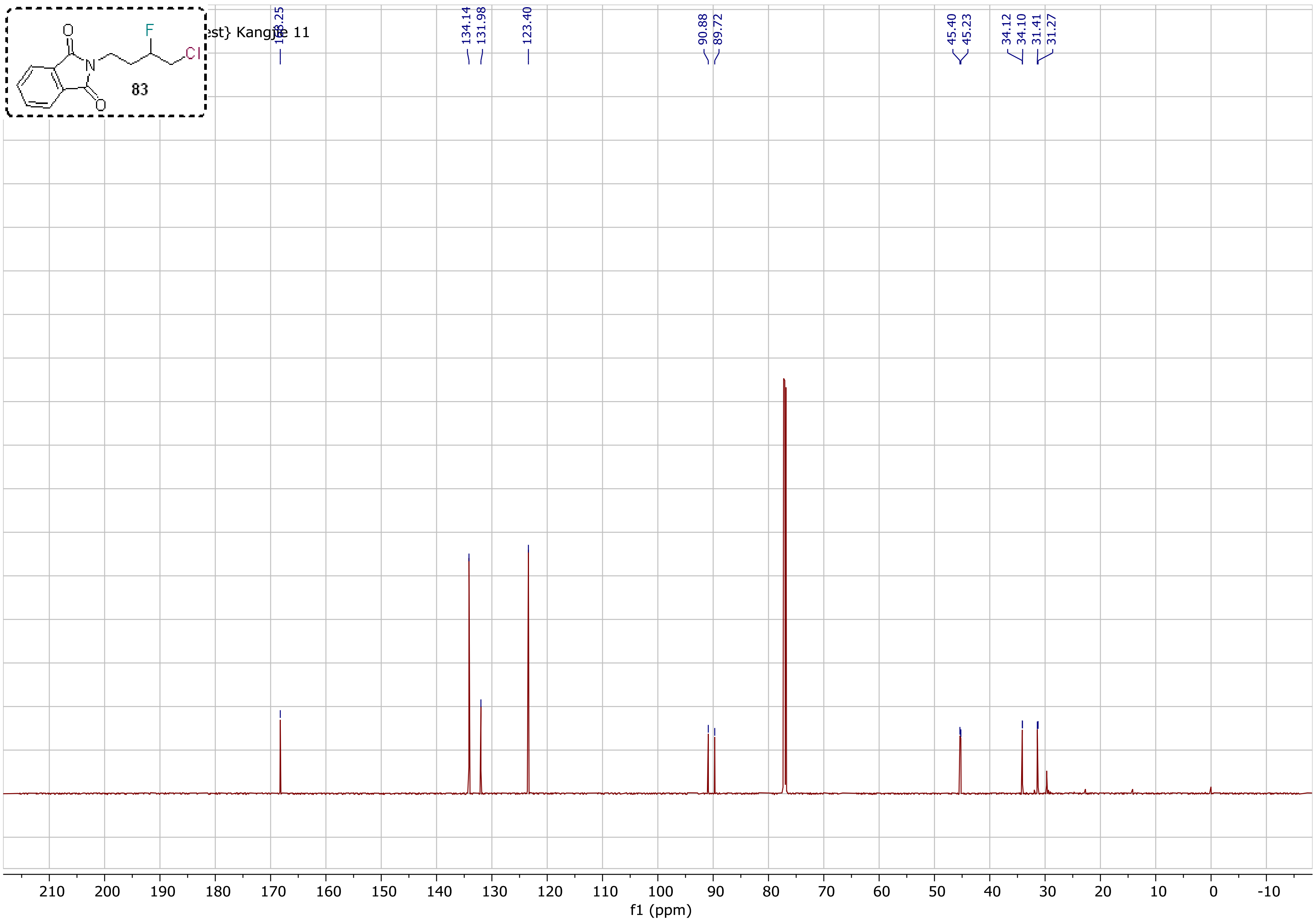

O
F
N
Cl
O
83
est} Kangjie 11
134.14
131.98
123.40
90.88
89.72
45.40
45.23
34.12
34.10
31.41
31.27
210 200 190 180 170 160 150 140 130 120 110 100 90 80 70 60 50 40 30 20 10 0 -10
f1 (ppm)

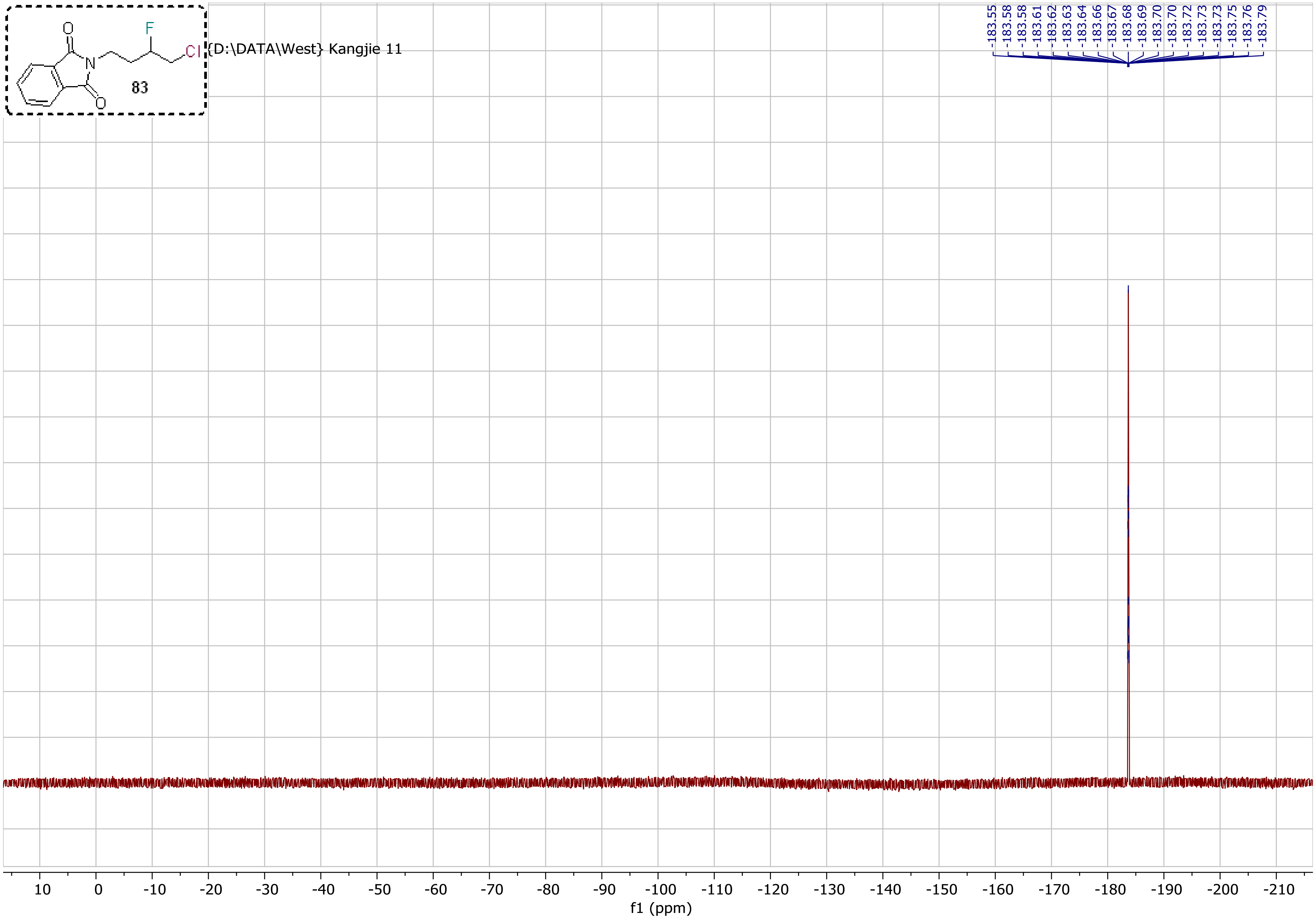

O
F
N
Cl
83
{D:\DATA\West} Kangjie 11
-183.55
-183.58
-183.58
-183.61
-183.62
-183.63
-183.64
-183.66
-183.67
-183.68
-183.69
-183.70
-183.72
-183.73
-183.73
-183.75
-183.76
-183.79
10 0 -10 -20 -30 -40 -50 -60 -70 -80 -90 -100 -110 -120 -130 -140 -150 -160 -170 -180 -190 -200 -210
f1 (ppm)

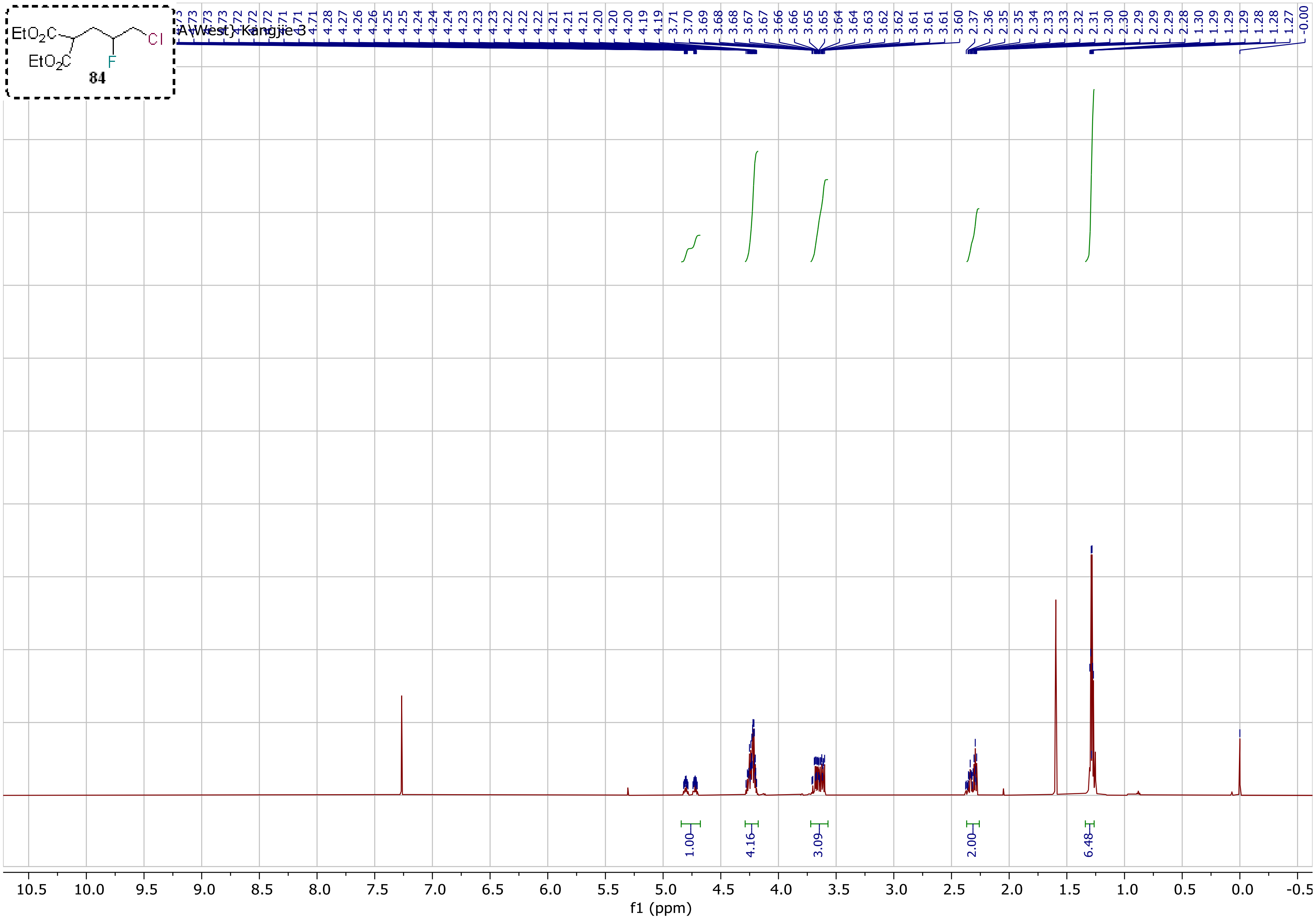

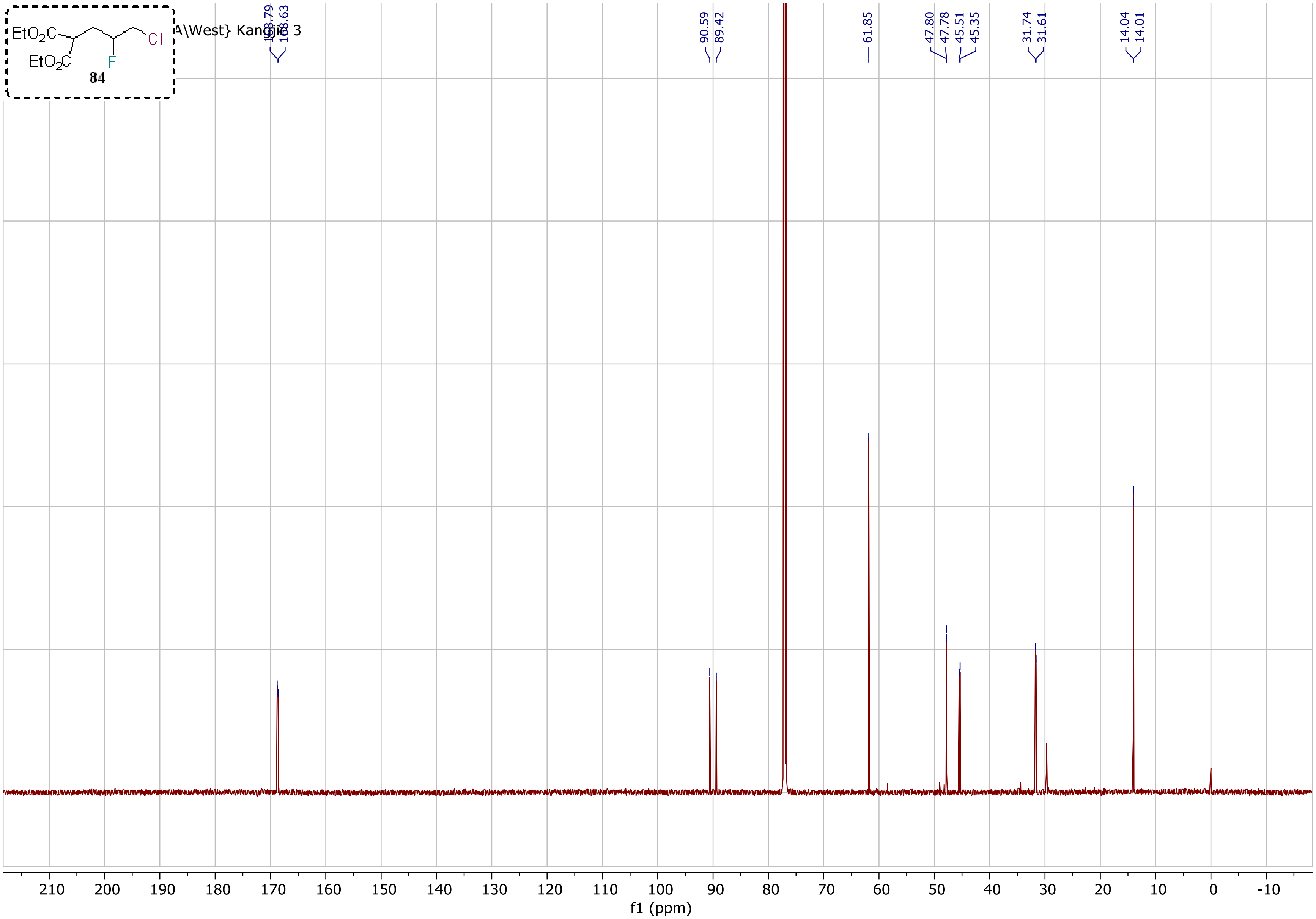
EtO2C
EtO2C
F
Cl
84
A\West} Kangjie 3
168.79
168.63
90.59
89.42
61.85
47.80
47.78
45.51
45.35
31.74
31.61
14.04
14.01
210 200 190 180 170 160 150 140 130 120 110 100 90 80 70 60 50 40 30 20 10 0 -10
f1 (ppm)

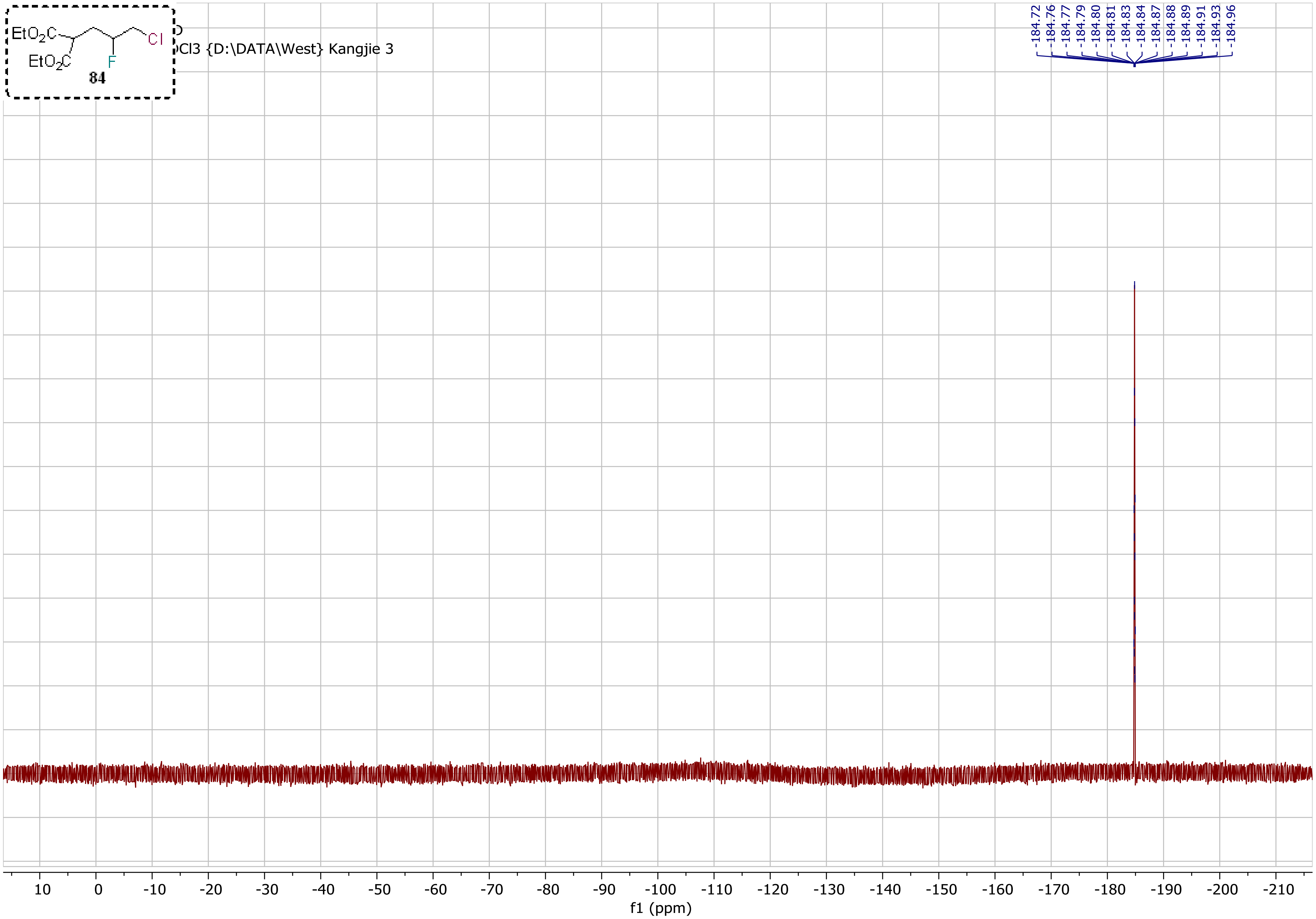
EtO2C
EtO2C
F
Cl
84
Cl3 {D:\DATA\West} Kangjie 3
-184.72
-184.76
-184.77
-184.79
-184.80
-184.81
-184.83
-184.84
-184.87
-184.88
-184.89
-184.91
-184.93
-184.96
10 0 -10 -20 -30 -40 -50 -60 -70 -80 -90 -100 -110 -120 -130 -140 -150 -160 -170 -180 -190 -200 -210
f1 (ppm)

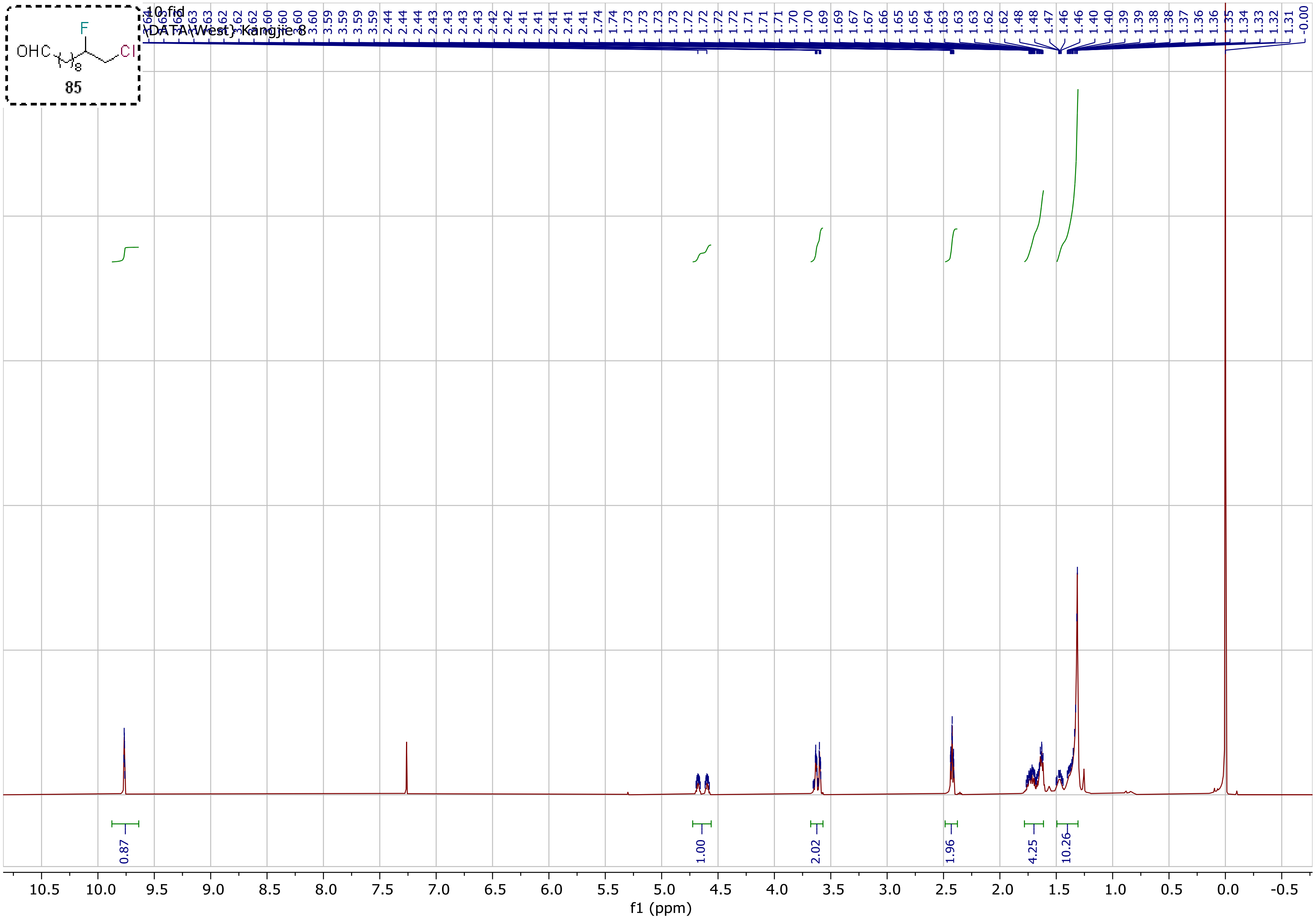

F
OHC
8
Cl
85
10.fid
f1 (ppm)

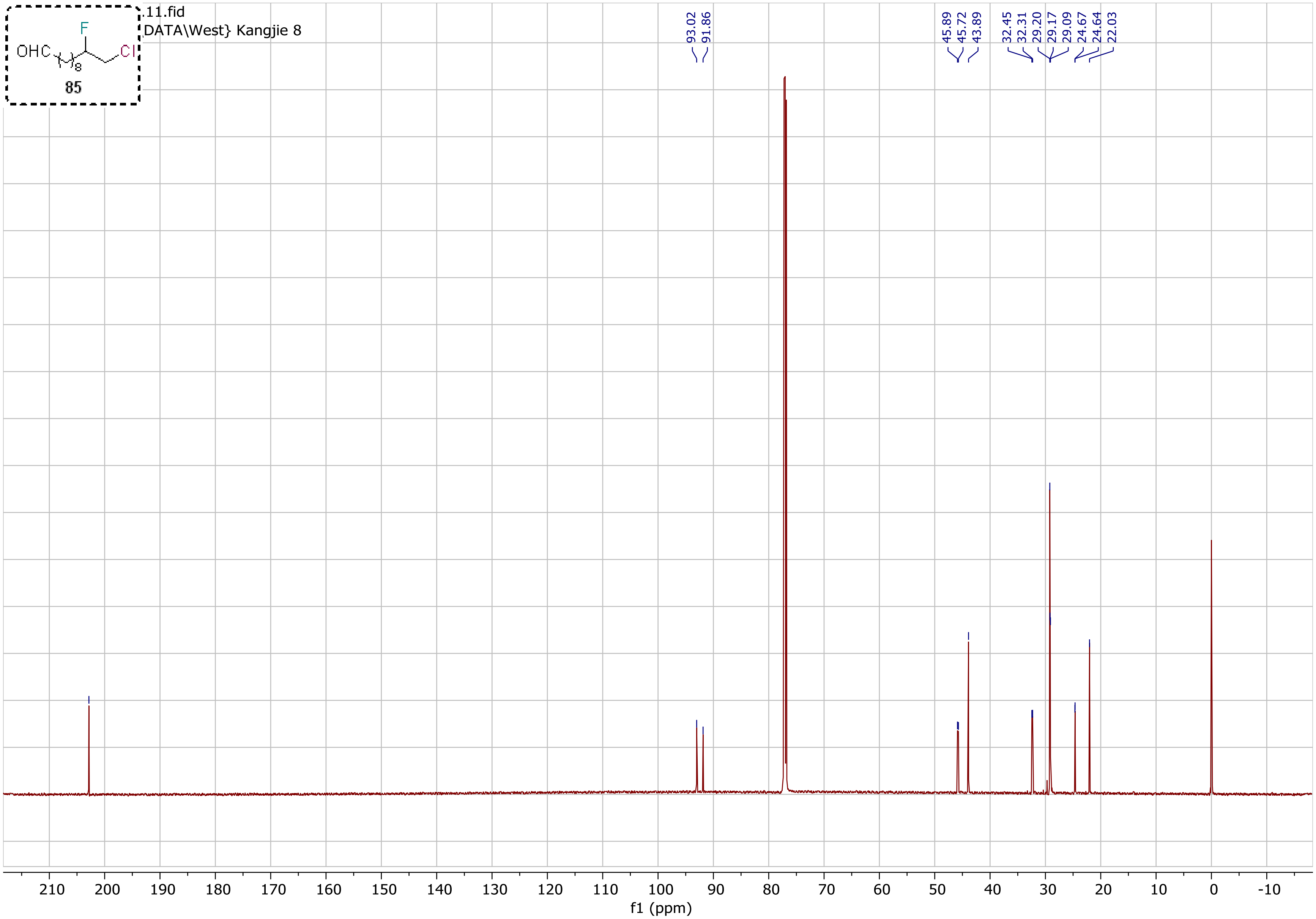

.11.fid
DATA\West} Kangjie 8
OHC
F
Cl
8
85
93.02
91.86
45.89
45.72
43.89
32.45
32.31
29.20
29.17
29.09
24.67
24.64
22.03
210
200
190
180
170
160
150
140
130
120
110
100
90
80
70
60
50
40
30
20
10
0
-10
f1 (ppm)

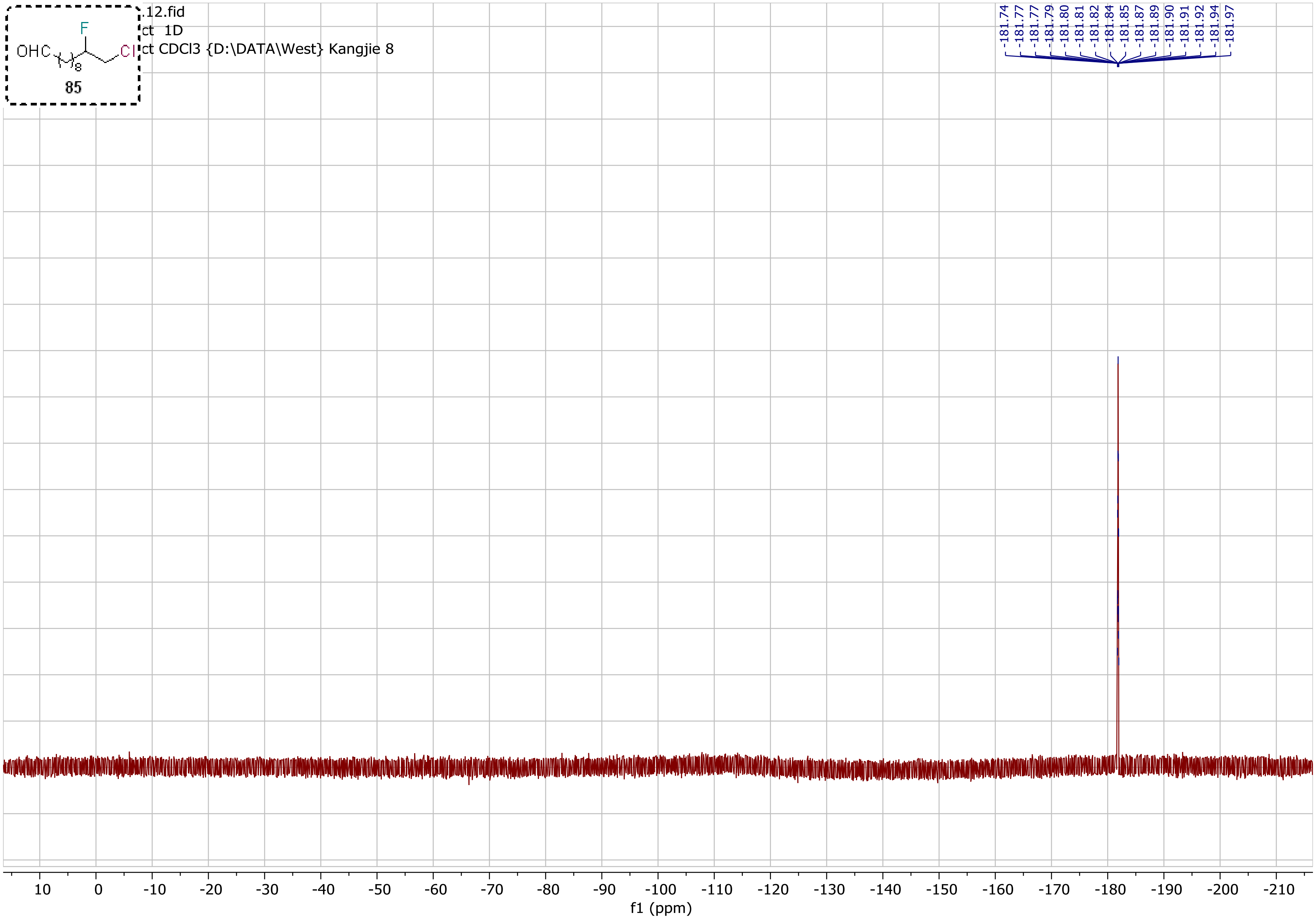

OHC
F
Cl
8
85
.12.fid
ct 1D
ct CDCl3 {D:\DATA\West} Kangjie 8
-181.74
-181.77
-181.77
-181.79
-181.80
-181.81
-181.82
-181.84
-181.85
-181.87
-181.89
-181.90
-181.91
-181.92
-181.94
-181.97
10
0
-10
-20
-30
-40
-50
-60
-70
-80
-90
-100
-110
-120
-130
-140
-150
-160
-170
-180
-190
-200
-210
f1 (ppm)

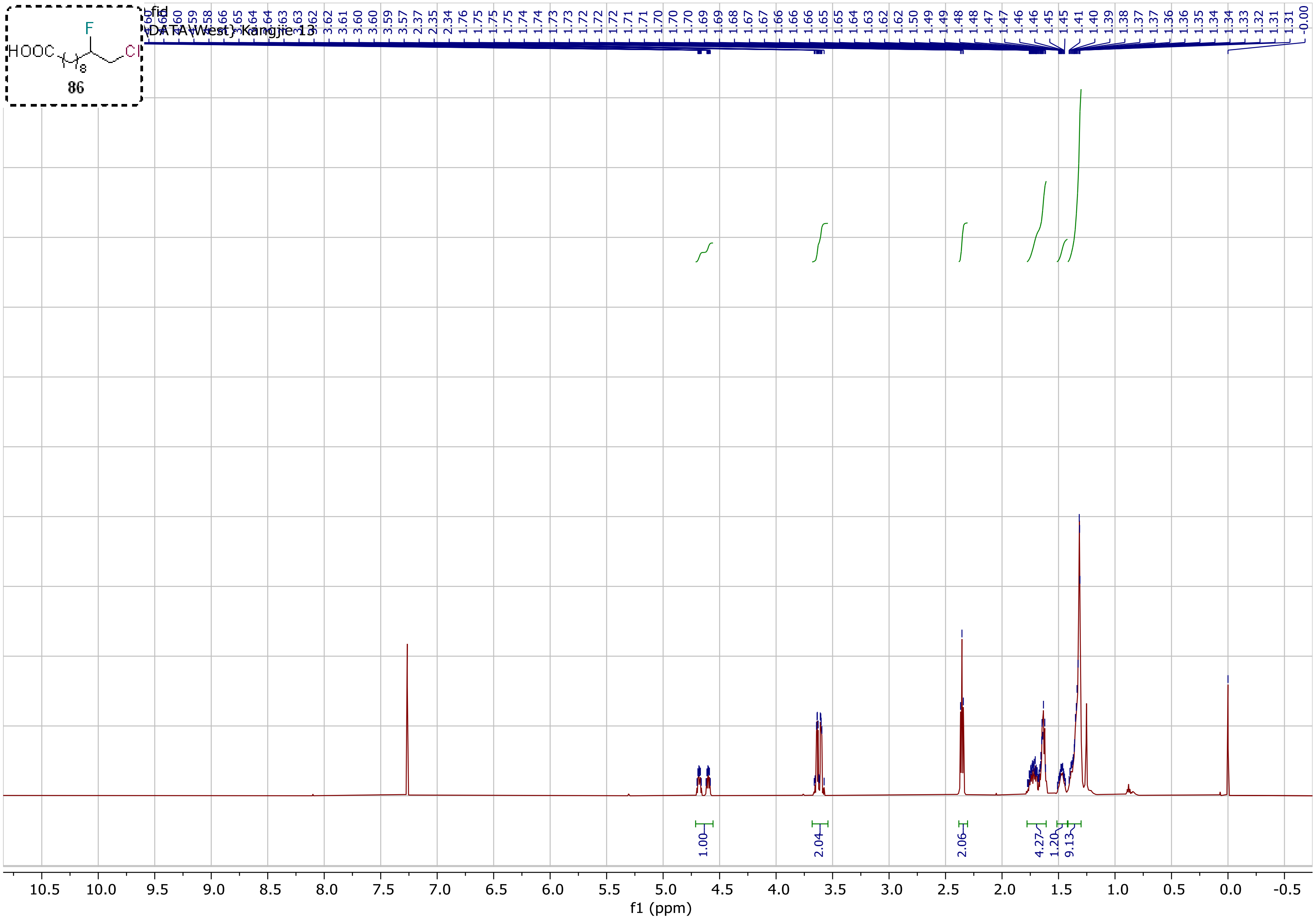

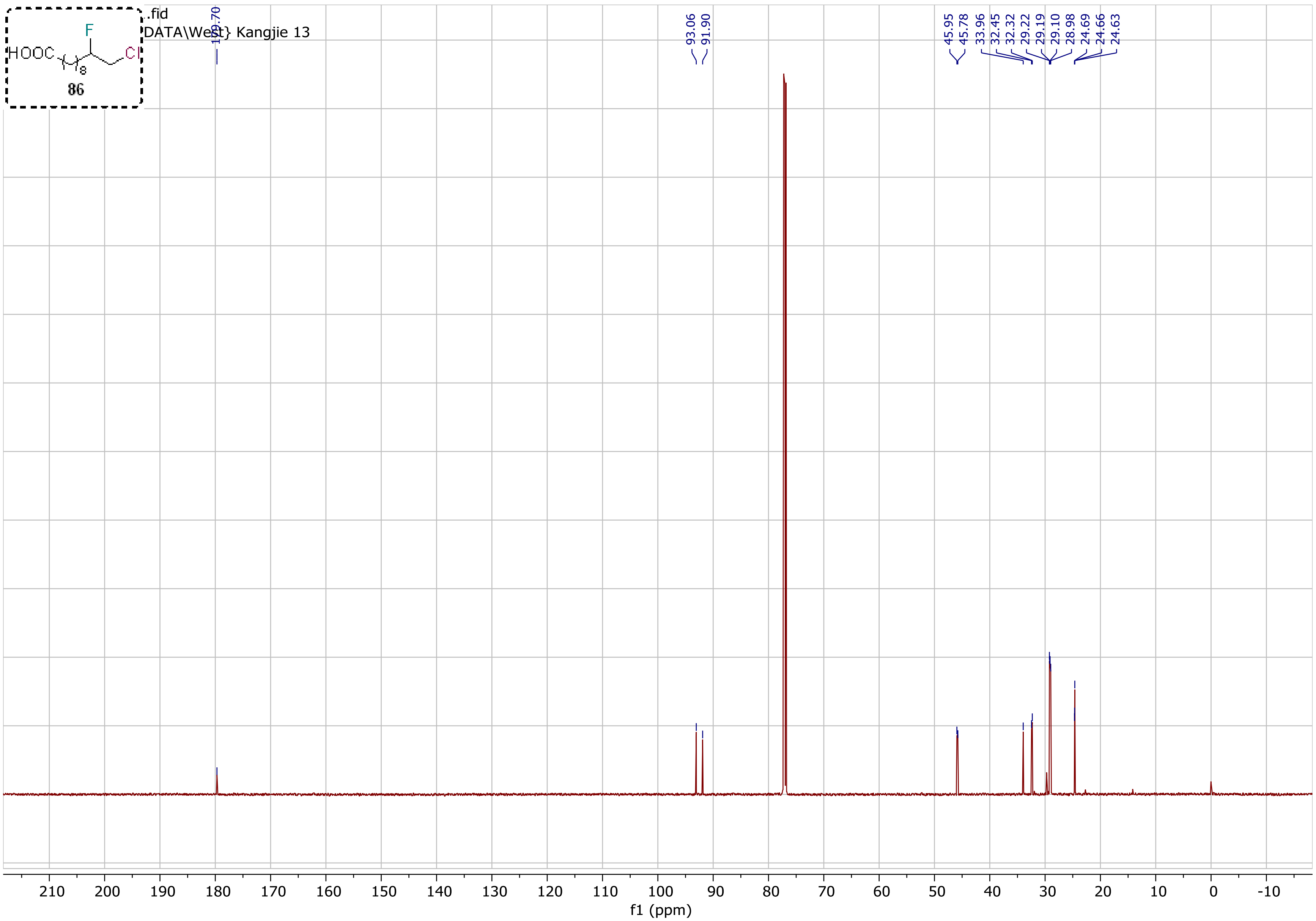

.fid
DATA\West} Kangjie 13
HOOC
F
Cl
8
86
179.70
93.06
91.90
45.95
45.78
33.96
32.45
32.32
29.22
29.19
29.10
28.98
24.69
24.66
24.63
210
200
190
180
170
160
150
140
130
120
110
100
90
80
70
60
50
40
30
20
10
0
-10
f1 (ppm)

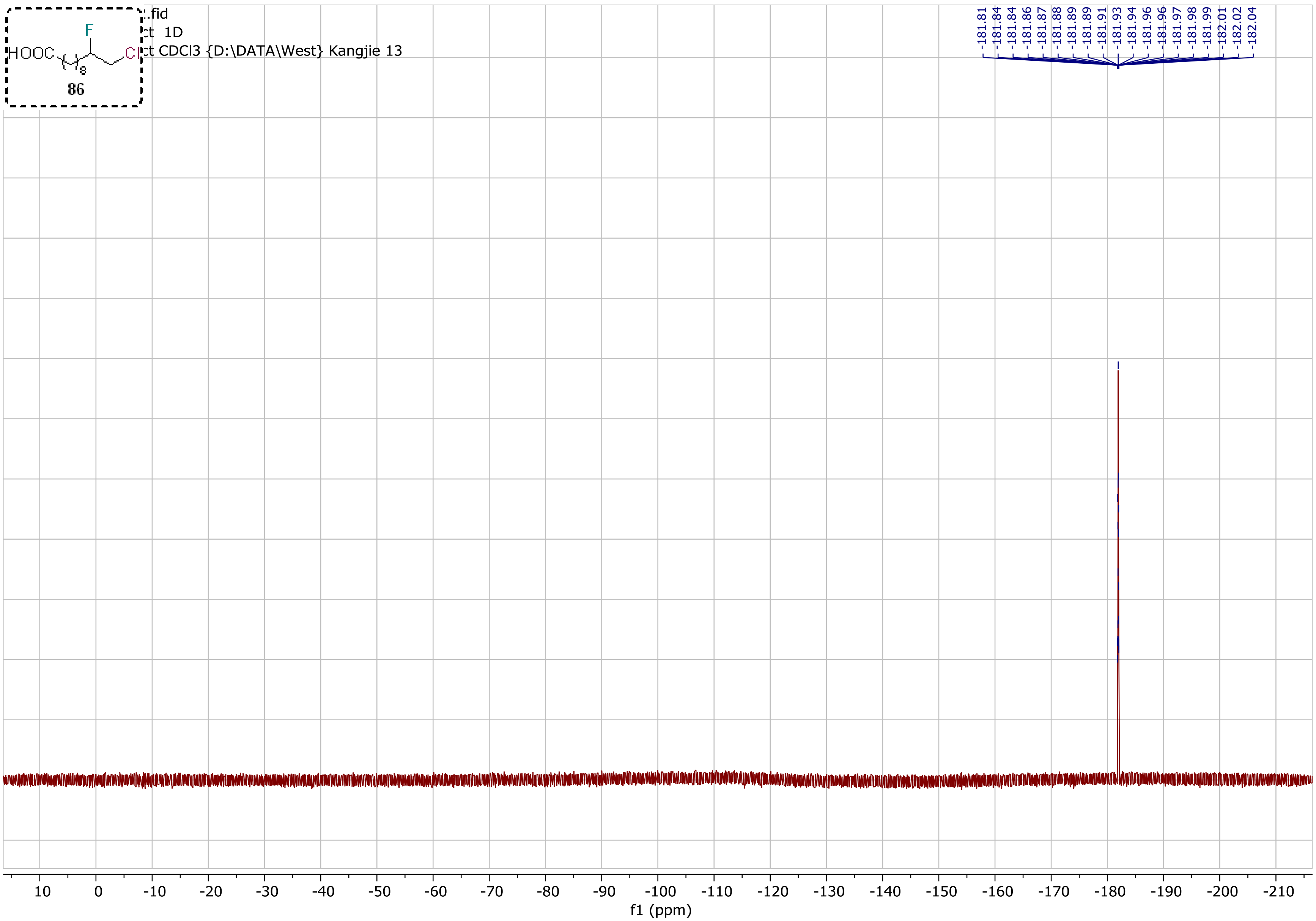

.fid
ct 1D
ct CDCl3 {D:\DATA\West} Kangjie 13
-181.81
-181.84
-181.84
-181.86
-181.87
-181.88
-181.89
-181.89
-181.91
-181.93
-181.94
-181.96
-181.96
-181.97
-181.98
-181.99
-182.01
-182.02
-182.04
10 0 -10 -20 -30 -40 -50 -60 -70 -80 -90 -100 -110 -120 -130 -140 -150 -160 -170 -180 -190 -200 -210
f1 (ppm)

86

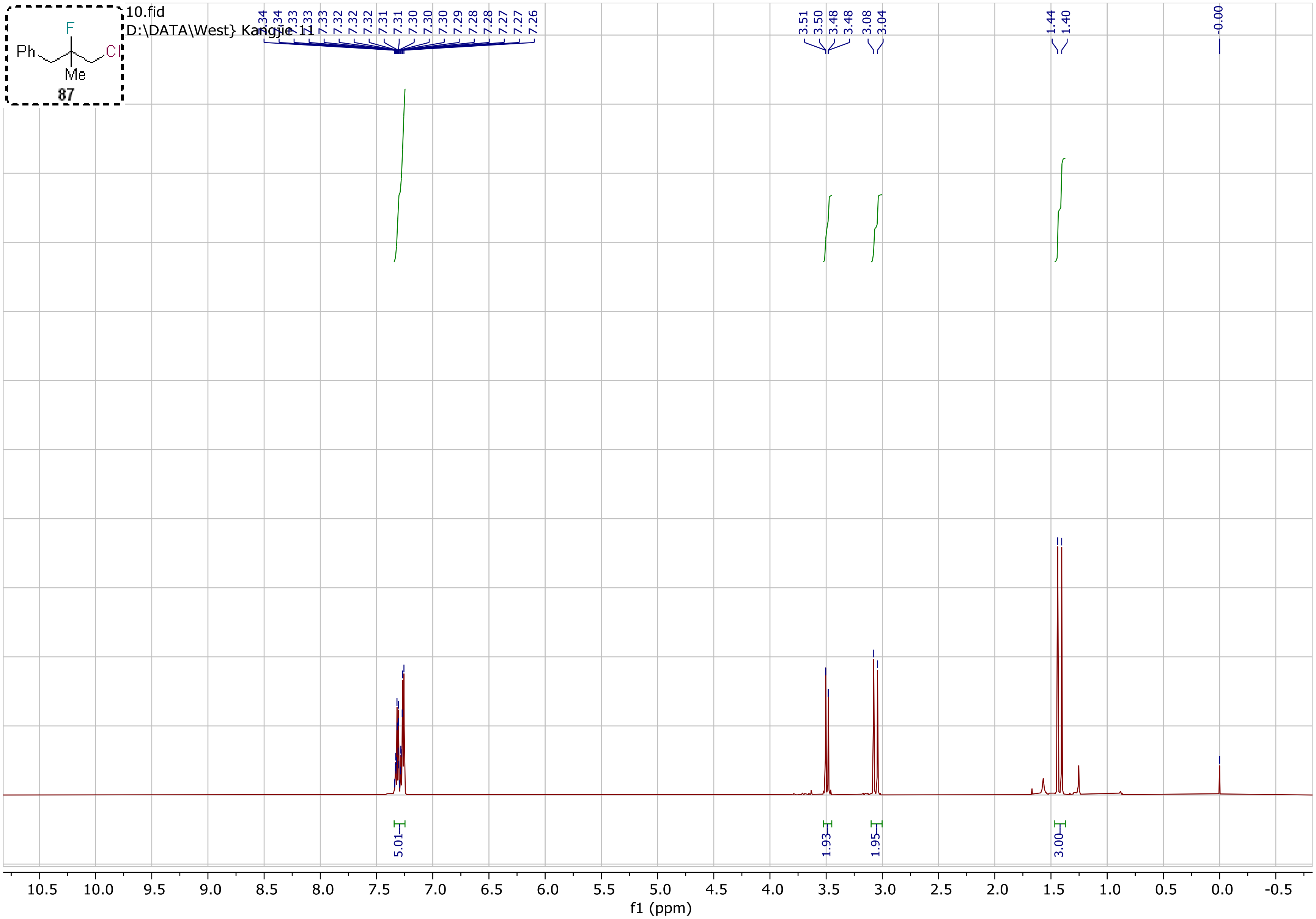

Ph
F
Cl
Me
87
10.fid
D:\DATA\West} Kangjie 11
7.34
7.34
7.33
7.33
7.33
7.32
7.32
7.32
7.31
7.31
7.30
7.30
7.30
7.29
7.28
7.27
7.27
7.26
3.51
3.50
3.48
3.48
3.08
3.04
1.44
1.40
-0.00
5.01
1.93
1.95
3.00
10.5
10.0
9.5
9.0
8.5
8.0
7.5
7.0
6.5
6.0
5.5
5.0
4.5
4.0
3.5
3.0
2.5
2.0
1.5
1.0
0.5
0.0
-0.5
f1 (ppm)

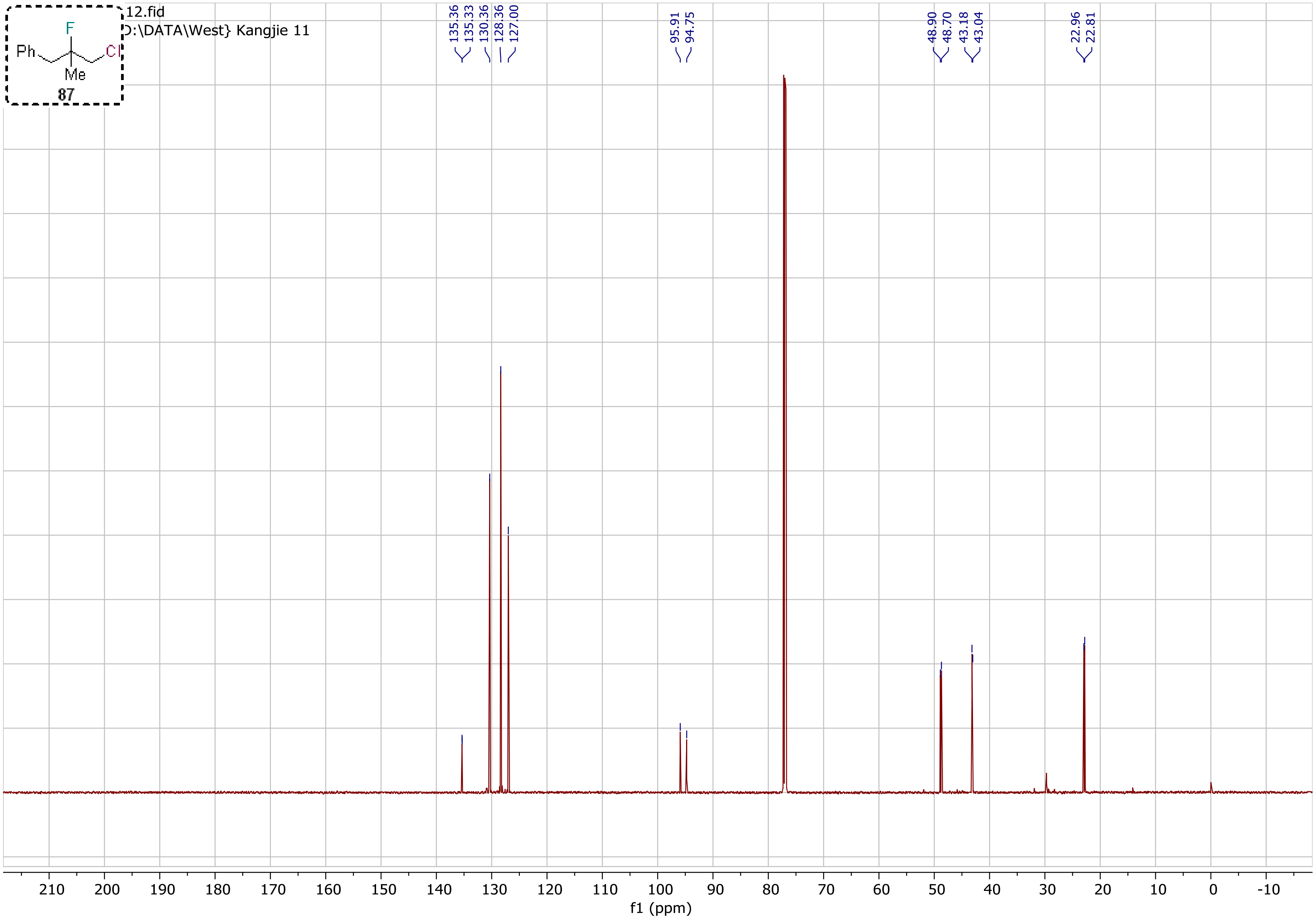

12.fid
D:\DATA\West} Kangjie 11
87
135.36
135.33
130.36
128.36
127.00
95.91
94.75
48.90
48.70
43.18
43.04
22.96
22.81
210 200 190 180 170 160 150 140 130 120 110 100 90 80 70 60 50 40 30 20 10 0 -10
f1 (ppm)

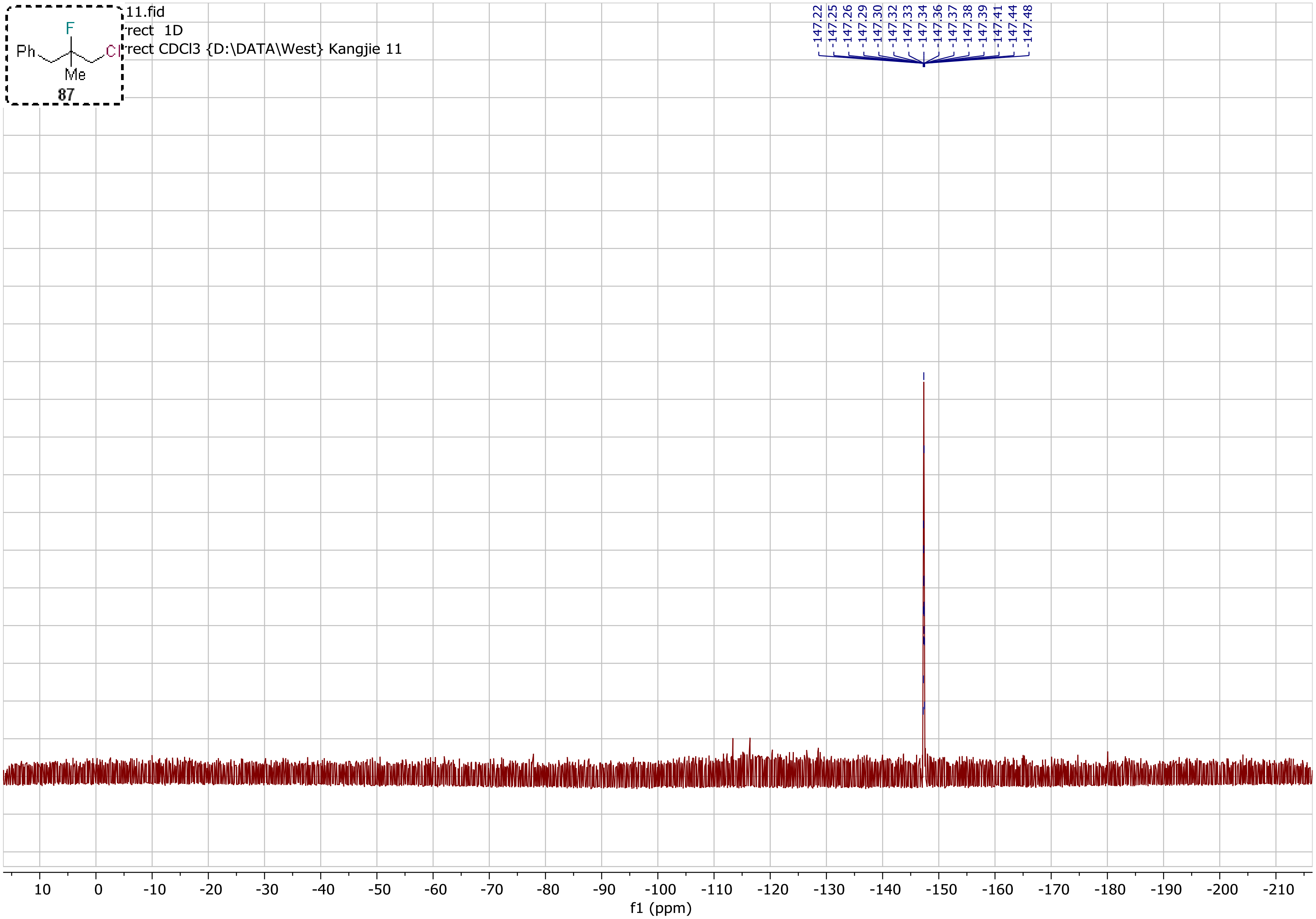
11.fid
rect 1D
rect CDCl3 {D:\DATA\West} Kangjie 11
-147.22
-147.25
-147.26
-147.29
-147.30
-147.32
-147.33
-147.34
-147.36
-147.37
-147.38
-147.39
-147.41
-147.44
-147.48
f1 (ppm)

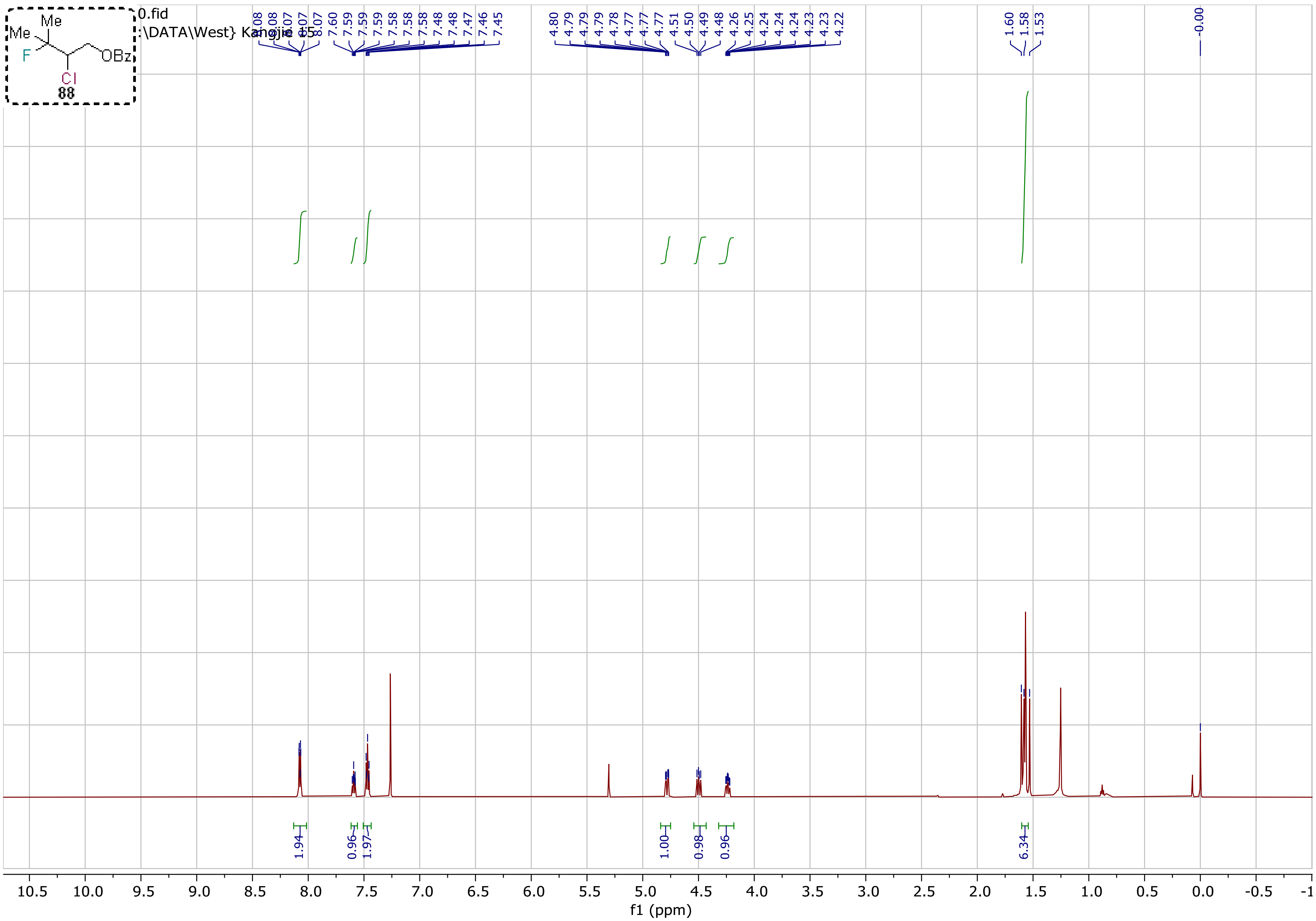
0.fid
:\DATA\West} Kangjie d5
Me
Me
F
Cl
OBz
88
8.08
8.08
8.07
8.07
7.60
7.59
7.59
7.58
7.58
7.48
7.48
7.47
7.46
7.45
4.80
4.79
4.79
4.78
4.77
4.77
4.51
4.50
4.49
4.48
4.26
4.25
4.24
4.24
4.24
4.23
4.23
4.22
1.60
1.58
1.53
-0.00
1.94
0.96
1.97
1.00
0.98
0.96
6.34
f1 (ppm)

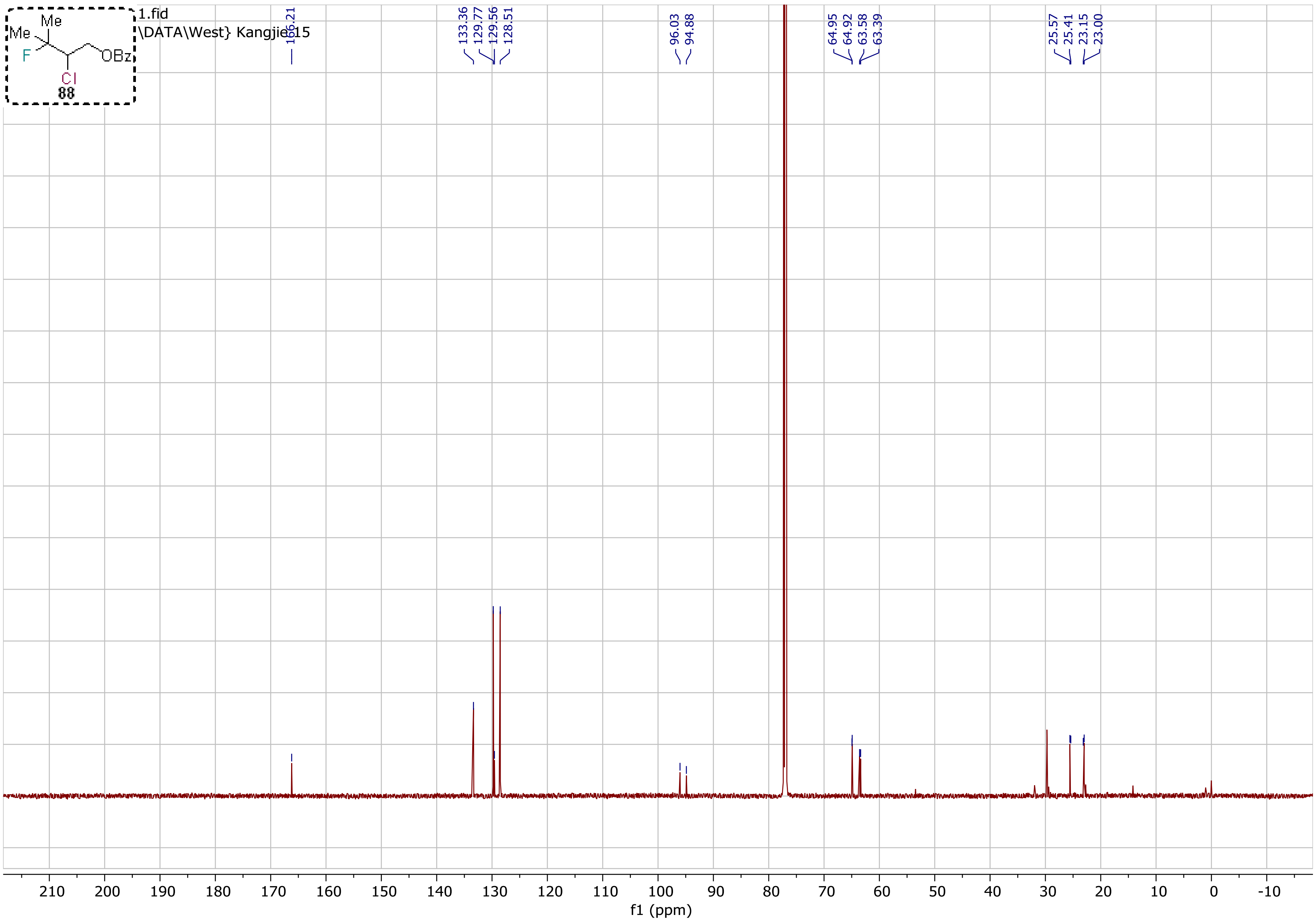
Me
Me
F
OBz
Cl
88
1.fid
\DATA\West} Kangjie 15
166.21
133.36
129.77
129.56
128.51
96.03
94.88
64.95
64.92
63.58
63.39
25.57
25.41
23.15
23.00
210 200 190 180 170 160 150 140 130 120 110 100 90 80 70 60 50 40 30 20 10 0 -10
f1 (ppm)

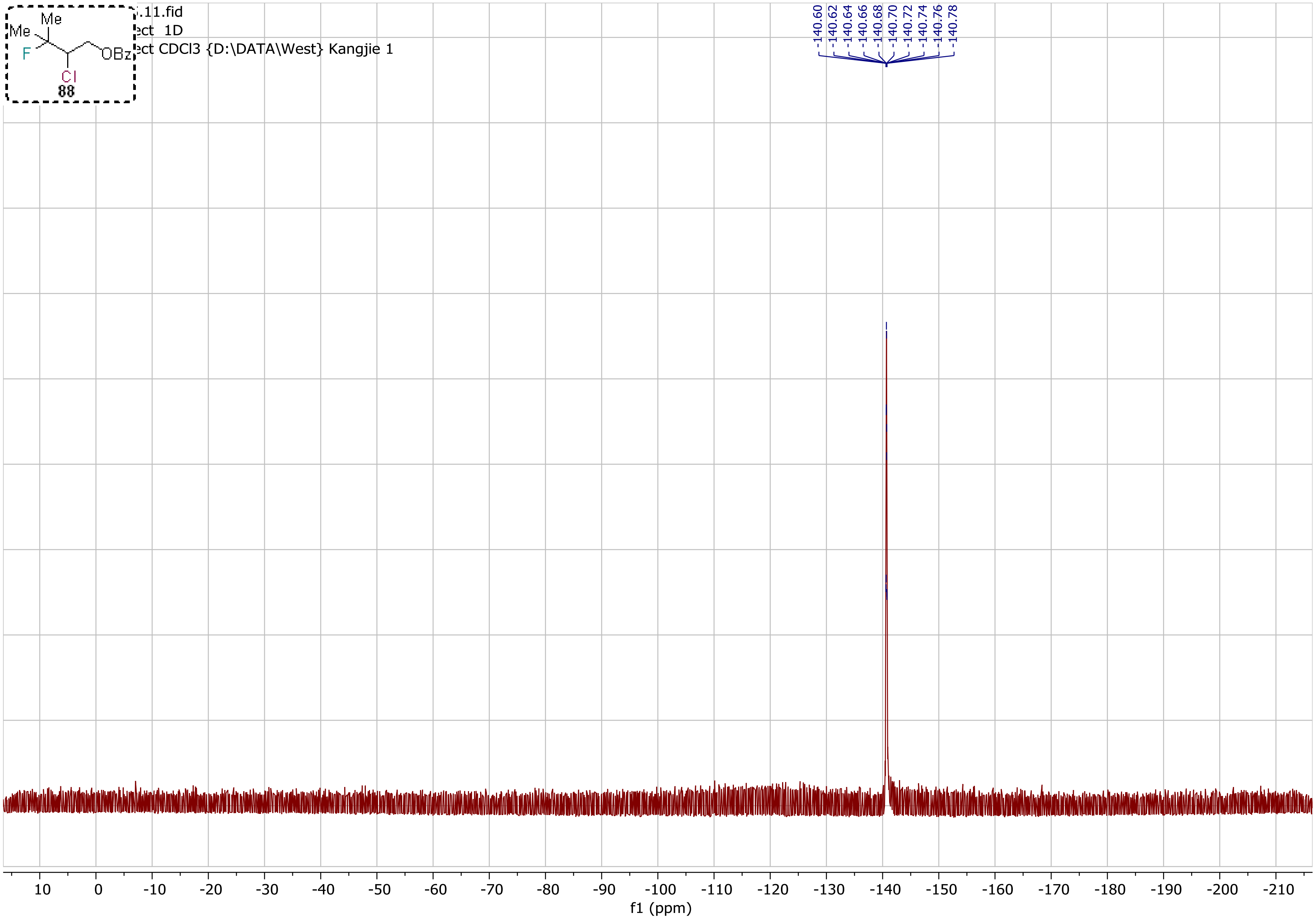

Me
Me
F
OBz
Cl
88
.11.fid
ect 1D
ect CDCl3 {D:\DATA\West} Kangjie 1
-140.60
-140.62
-140.64
-140.66
-140.68
-140.70
-140.72
-140.74
-140.76
-140.78
10 0 -10 -20 -30 -40 -50 -60 -70 -80 -90 -100 -110 -120 -130 -140 -150 -160 -170 -180 -190 -200 -210
f1 (ppm)

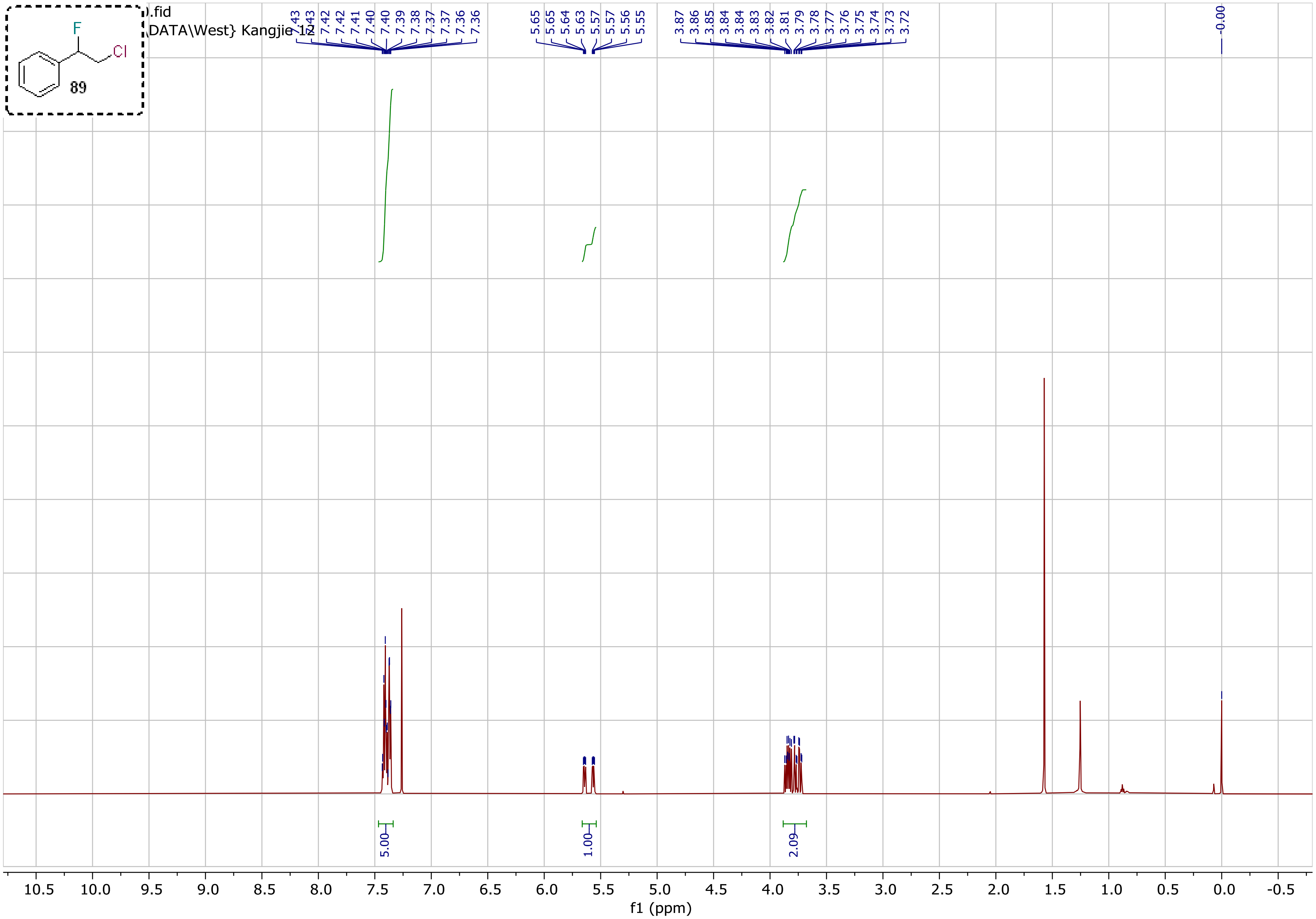

89
).fid
{DATA\West} Kangjie 12
7.43
7.43
7.42
7.42
7.41
7.40
7.40
7.39
7.38
7.37
7.37
7.36
7.36
5.65
5.65
5.64
5.63
5.57
5.57
5.56
5.55
3.87
3.86
3.85
3.84
3.84
3.83
3.82
3.81
3.79
3.78
3.77
3.76
3.75
3.74
3.73
3.72
-0.00
5.00
1.00
2.09
f1 (ppm)

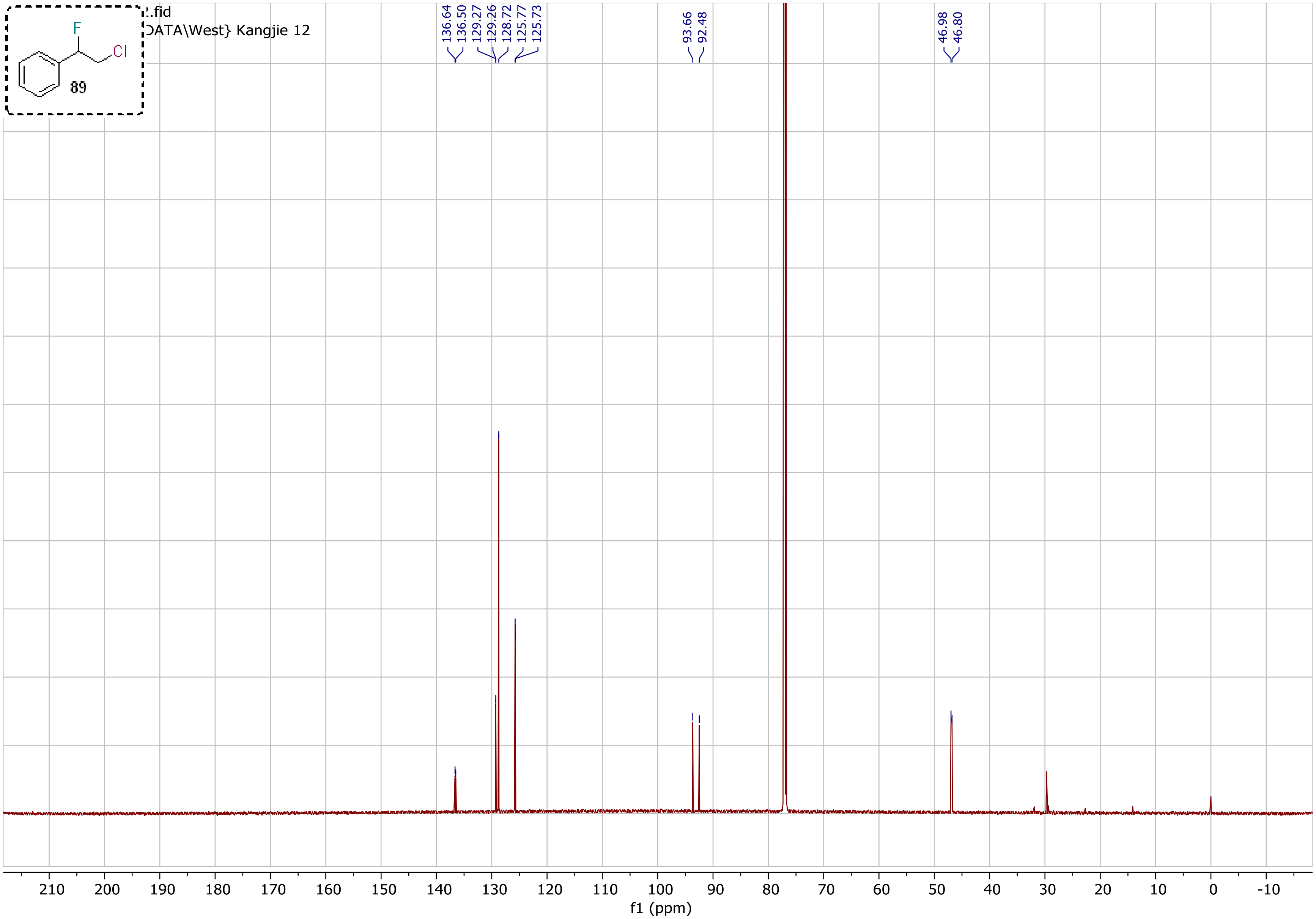

F
Cl
89
2.fid
DATA\West} Kangjie 12
136.64
136.50
129.27
129.26
128.72
125.77
125.73
93.66
92.48
46.98
46.80
210
200
190
180
170
160
150
140
130
120
110
100
90
80
70
60
50
40
30
20
10
0
-10
f1 (ppm)

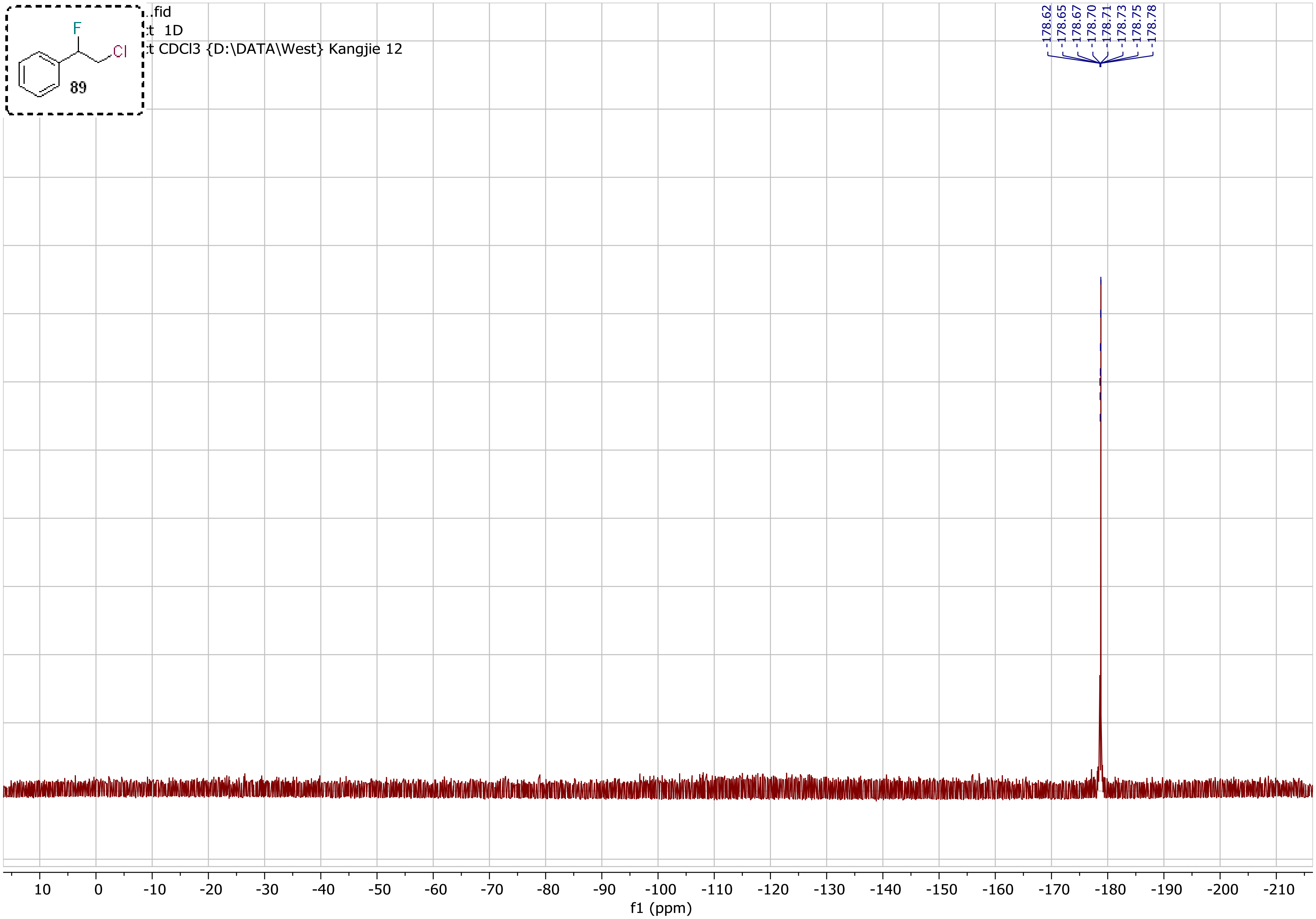

..fid
:t 1D
:t CDCl3 {D:\DATA\West} Kangjie 12
F
Cl
89
-178.62
-178.65
-178.67
-178.70
-178.71
-178.73
-178.75
-178.78
10 0 -10 -20 -30 -40 -50 -60 -70 -80 -90 -100 -110 -120 -130 -140 -150 -160 -170 -180 -190 -200 -210
f1 (ppm)

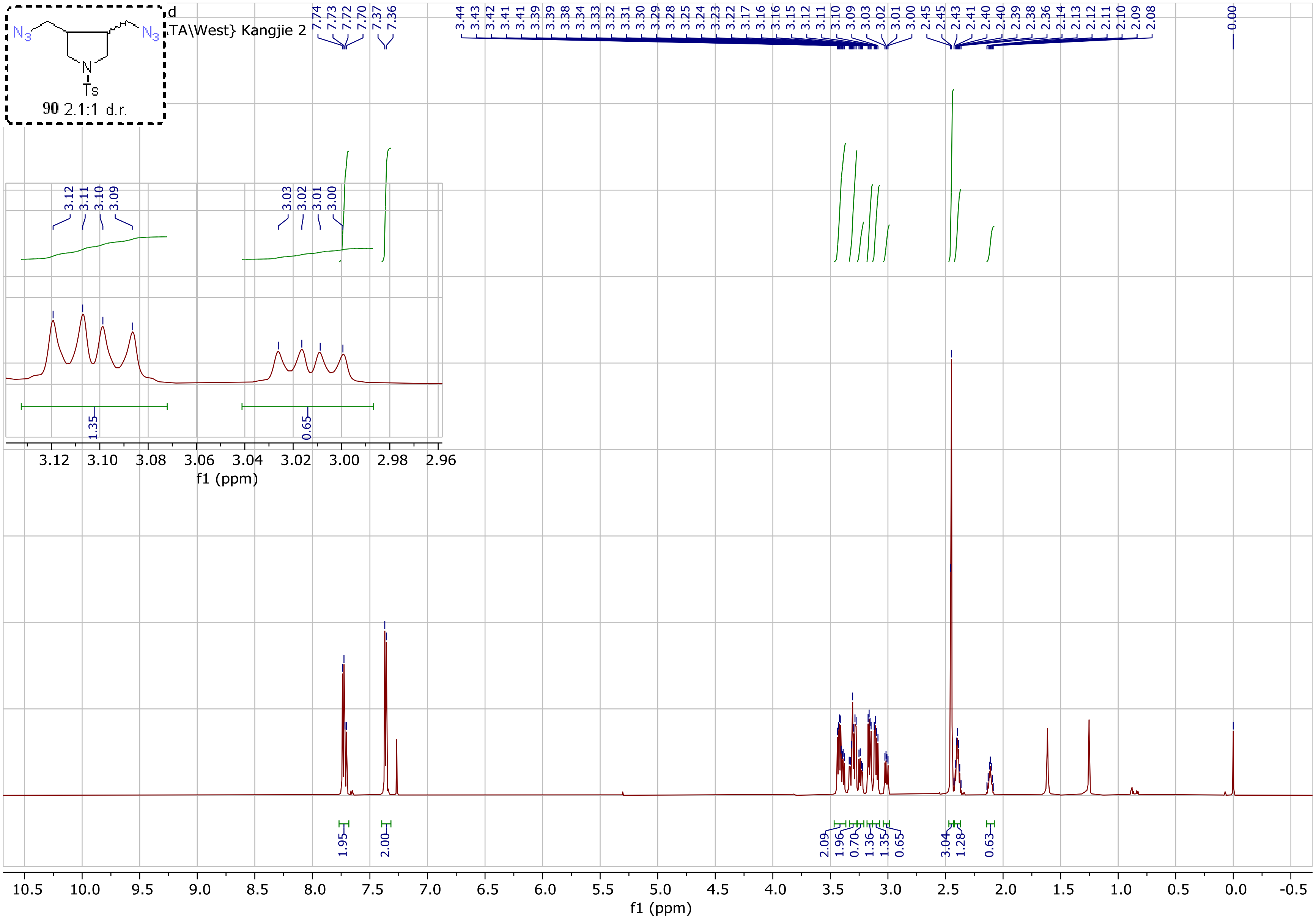

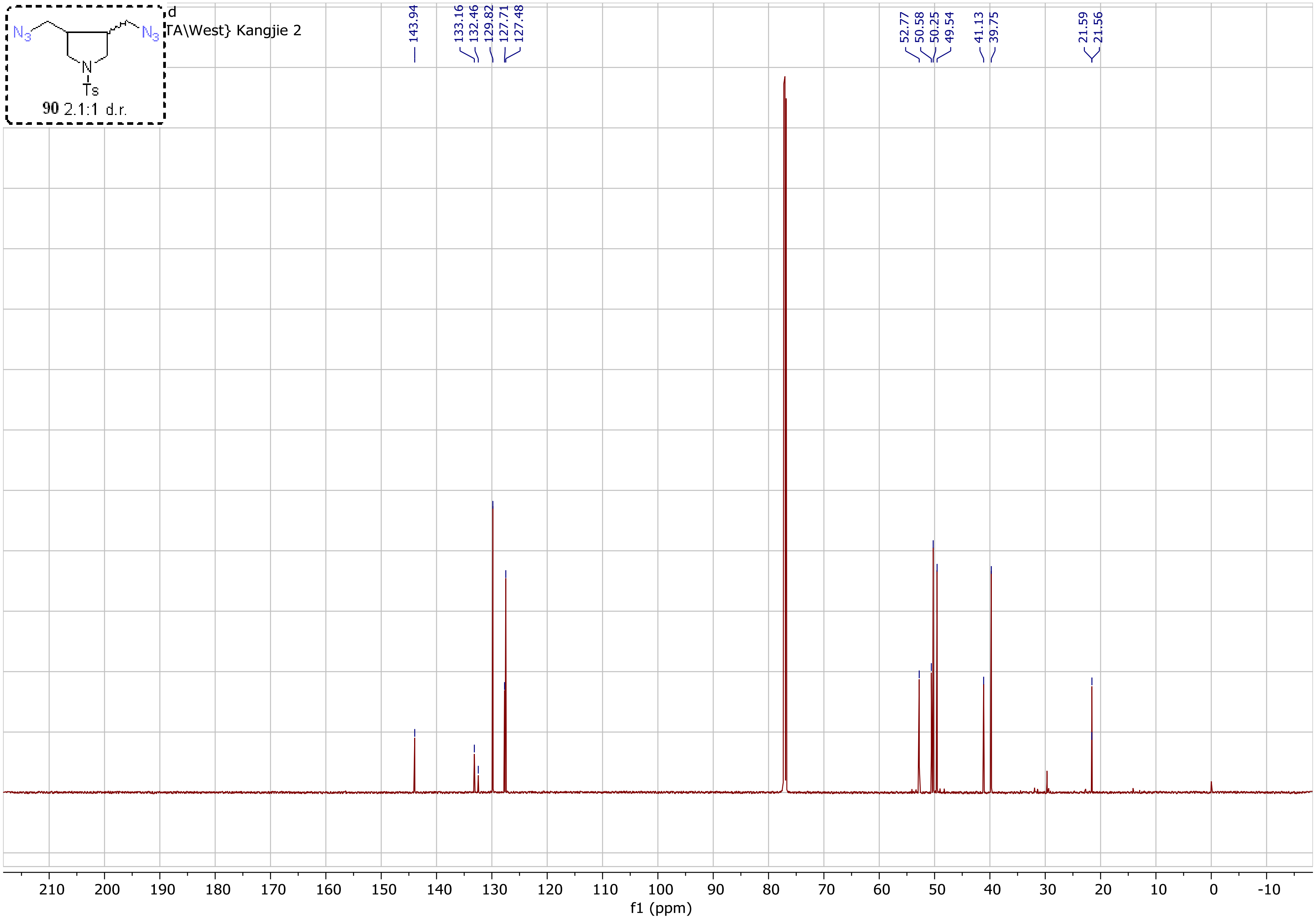

N3
N3
N
Ts
90 2.1:1 d.r.
d
TA\West} Kangjie 2
143.94
133.16
132.46
129.82
127.71
127.48
52.77
50.58
50.25
49.54
41.13
39.75
21.59
21.56
210 200 190 180 170 160 150 140 130 120 110 100 90 80 70 60 50 40 30 20 10 0 -10
f1 (ppm)

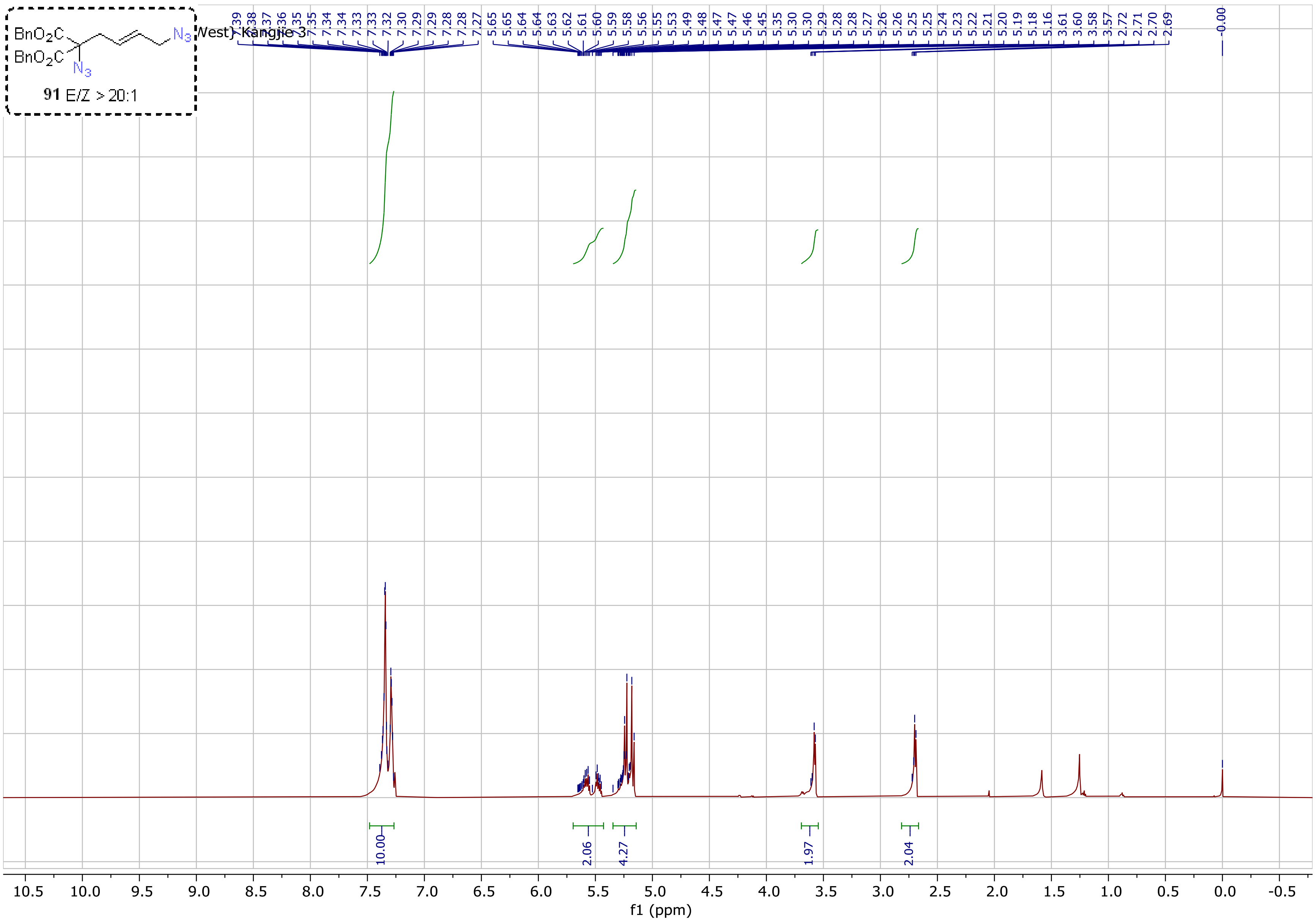
BnO2C
BnO2C
N3
N3
91 E/Z > 20:1
f1 (ppm)
10.00
2.06
4.27
1.97
2.04

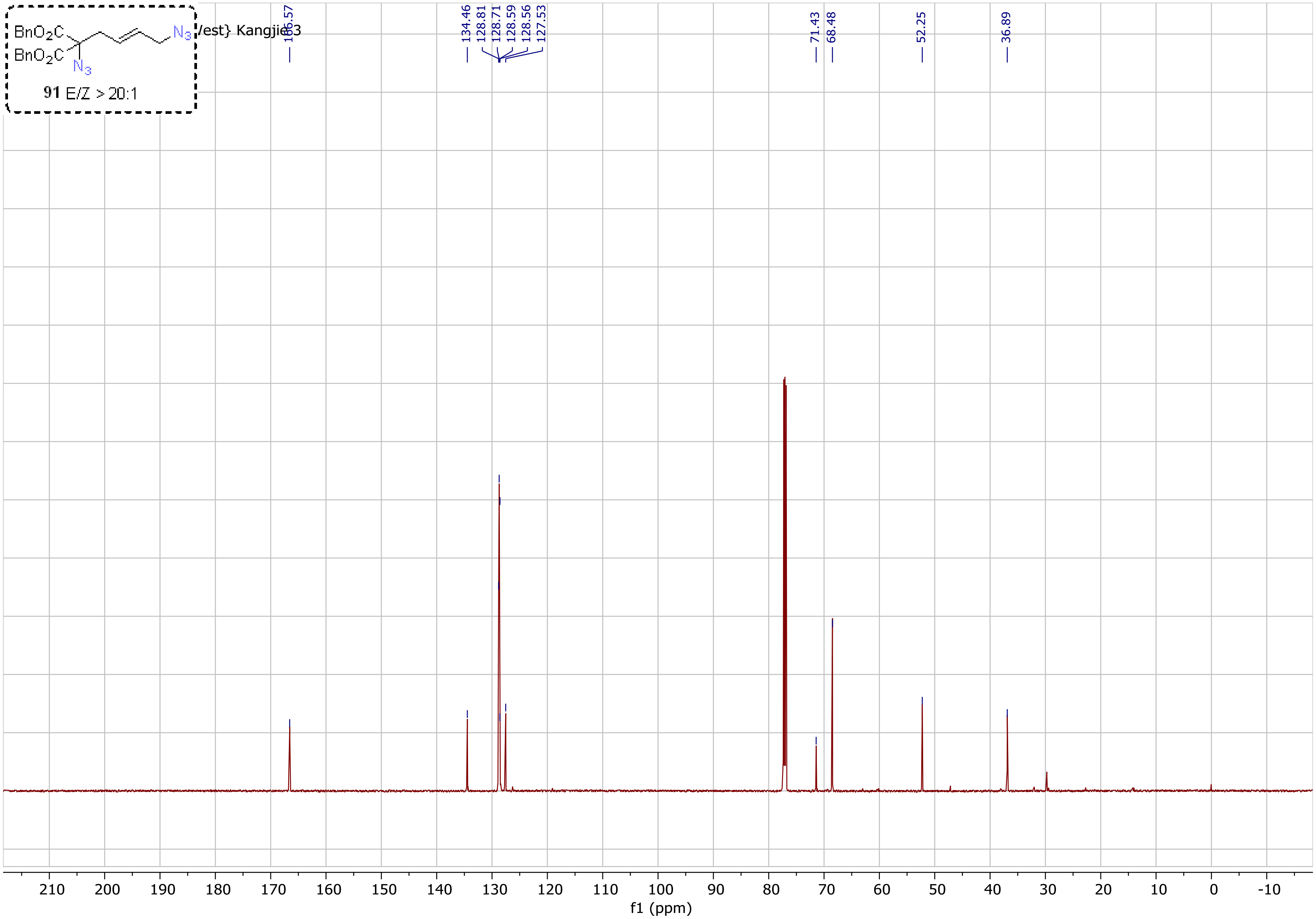

BnO2C
BnO2C
N3
N3
91 E/Z > 20:1
Kangjie63
166.57
134.46
128.81
128.71
128.59
128.56
127.53
71.43
68.48
52.25
36.89
210 200 190 180 170 160 150 140 130 120 110 100 90 80 70 60 50 40 30 20 10 0 -10
f1 (ppm)

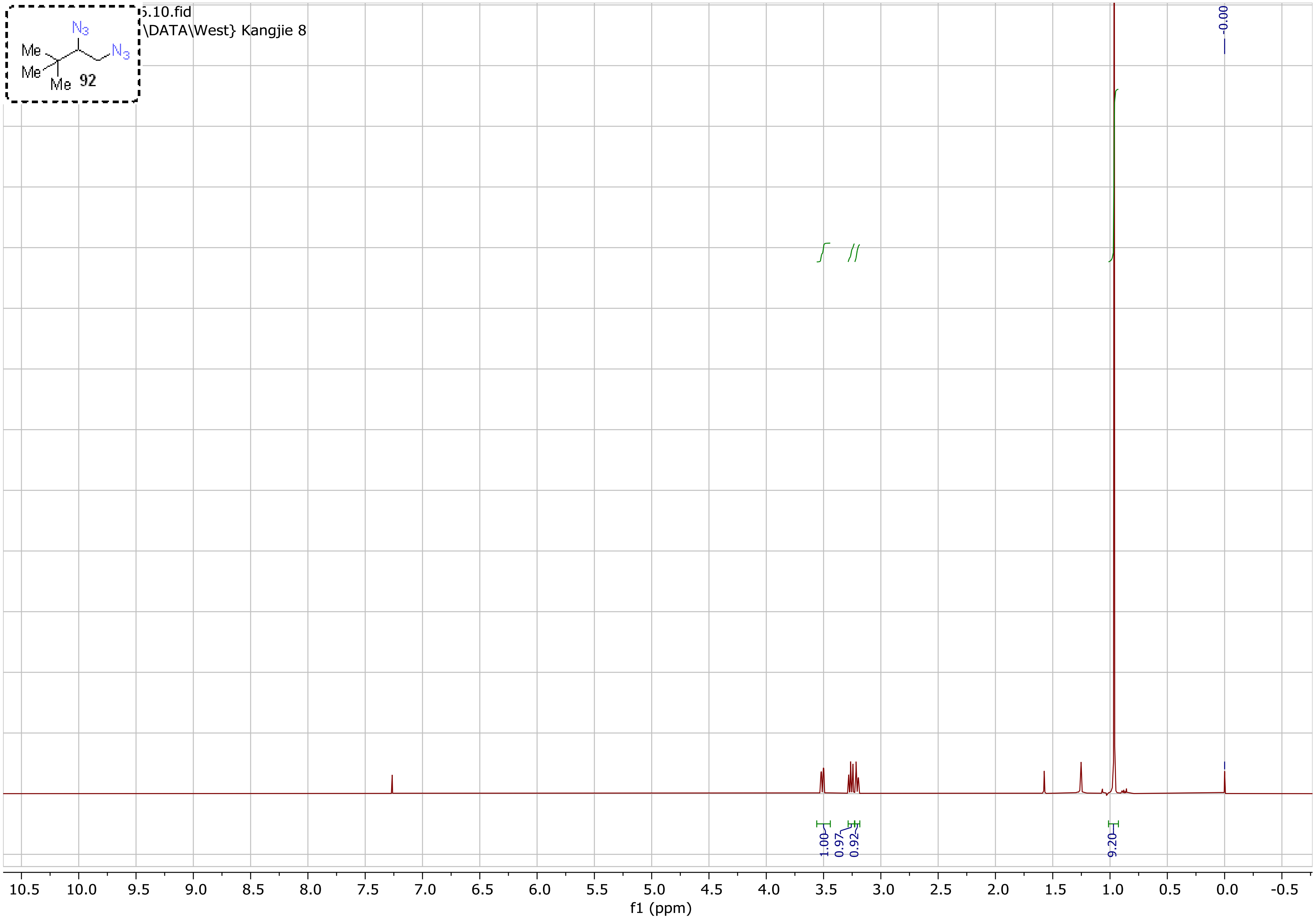

5.10.fid
\DATA\West} Kangjie 8
N3
N3
Me
Me
Me
92
-0.00
1.00
0.97
0.92
9.20
10.5 10.0 9.5 9.0 8.5 8.0 7.5 7.0 6.5 6.0 5.5 5.0 4.5 4.0 3.5 3.0 2.5 2.0 1.5 1.0 0.5 0.0 -0.5
f1 (ppm)

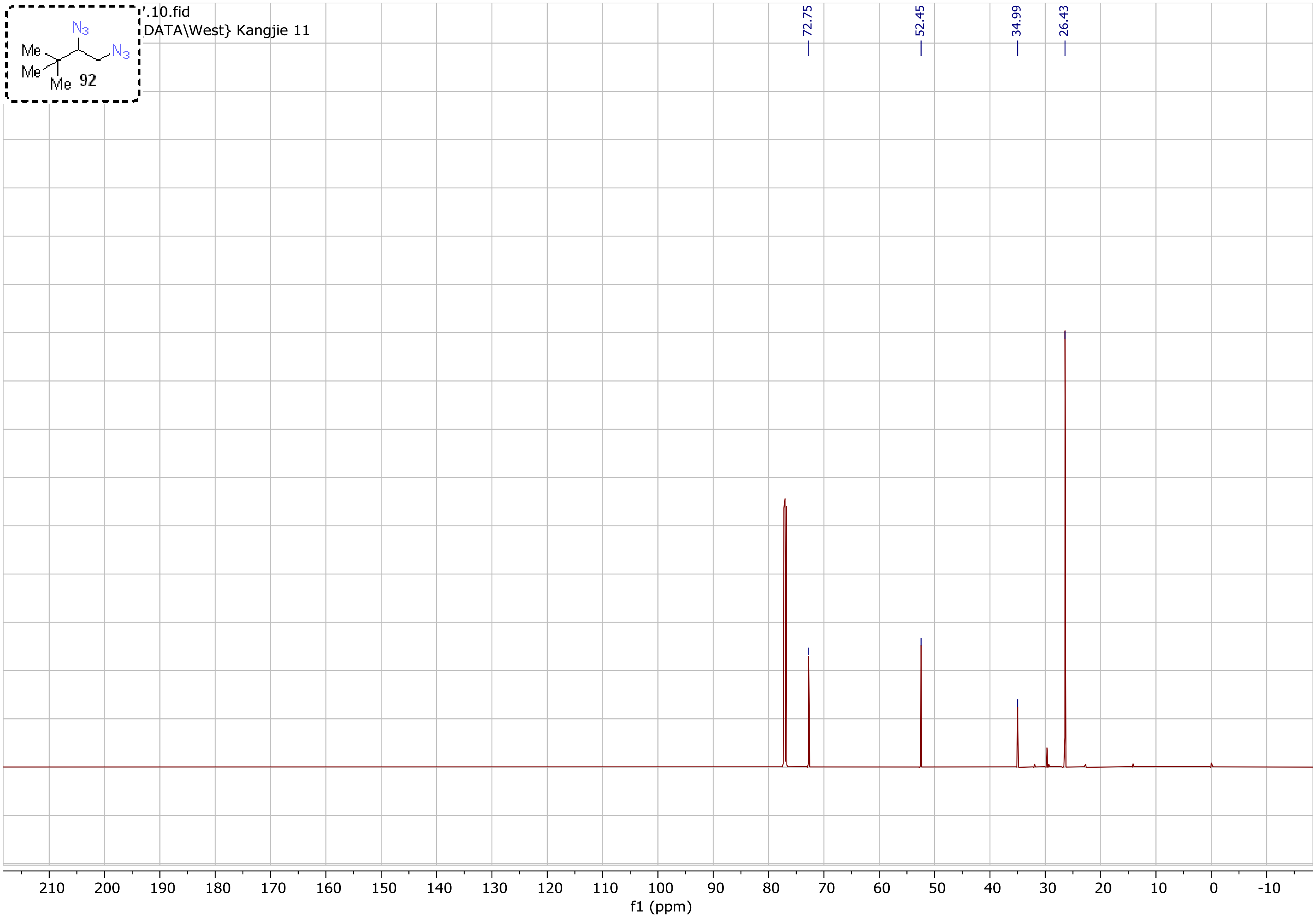

N3
Me
Me
Me
N3
92
'.10.fid
DATA\West} Kangjie 11
72.75
52.45
34.99
26.43
210
200
190
180
170
160
150
140
130
120
110
100
90
80
70
60
50
40
30
20
10
0
-10
f1 (ppm)

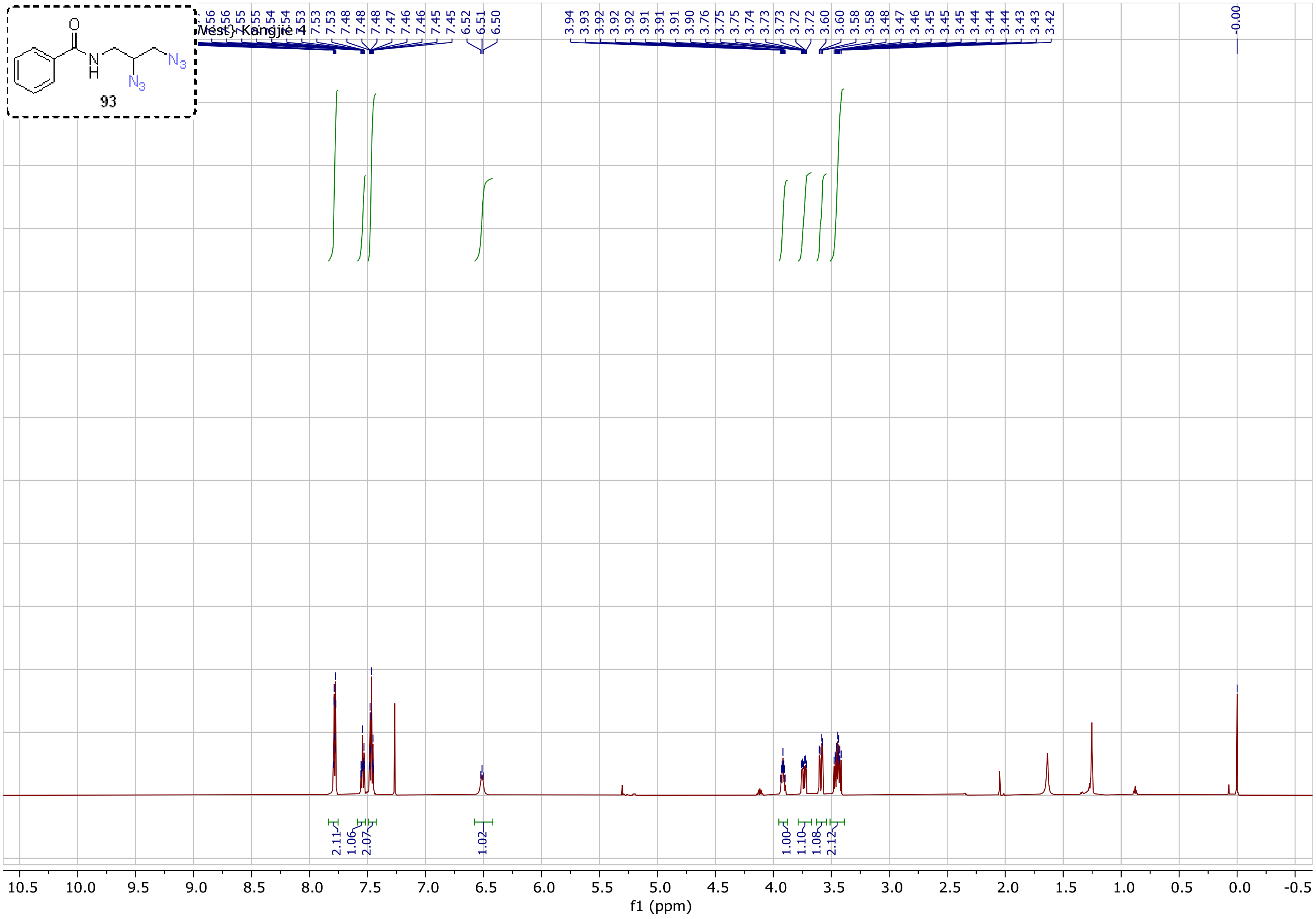
93
7.56 7.56 7.55 7.55 7.54 7.54 7.53 7.53 7.53 7.48 7.48 7.47 7.46 7.46 7.45 7.45 6.52 6.51 6.50
3.94 3.93 3.92 3.92 3.91 3.91 3.91 3.90 3.76 3.75 3.75 3.74 3.73 3.73 3.72 3.72 3.60 3.60 3.58 3.48 3.47 3.46 3.45 3.45 3.44 3.44 3.44 3.43 3.43 3.42
-0.00
2.11 1.06 2.07 1.02 1.00 1.10 1.08 2.12
10.5 10.0 9.5 9.0 8.5 8.0 7.5 7.0 6.5 6.0 5.5 5.0 4.5 4.0 3.5 3.0 2.5 2.0 1.5 1.0 0.5 0.0 -0.5
f1 (ppm)

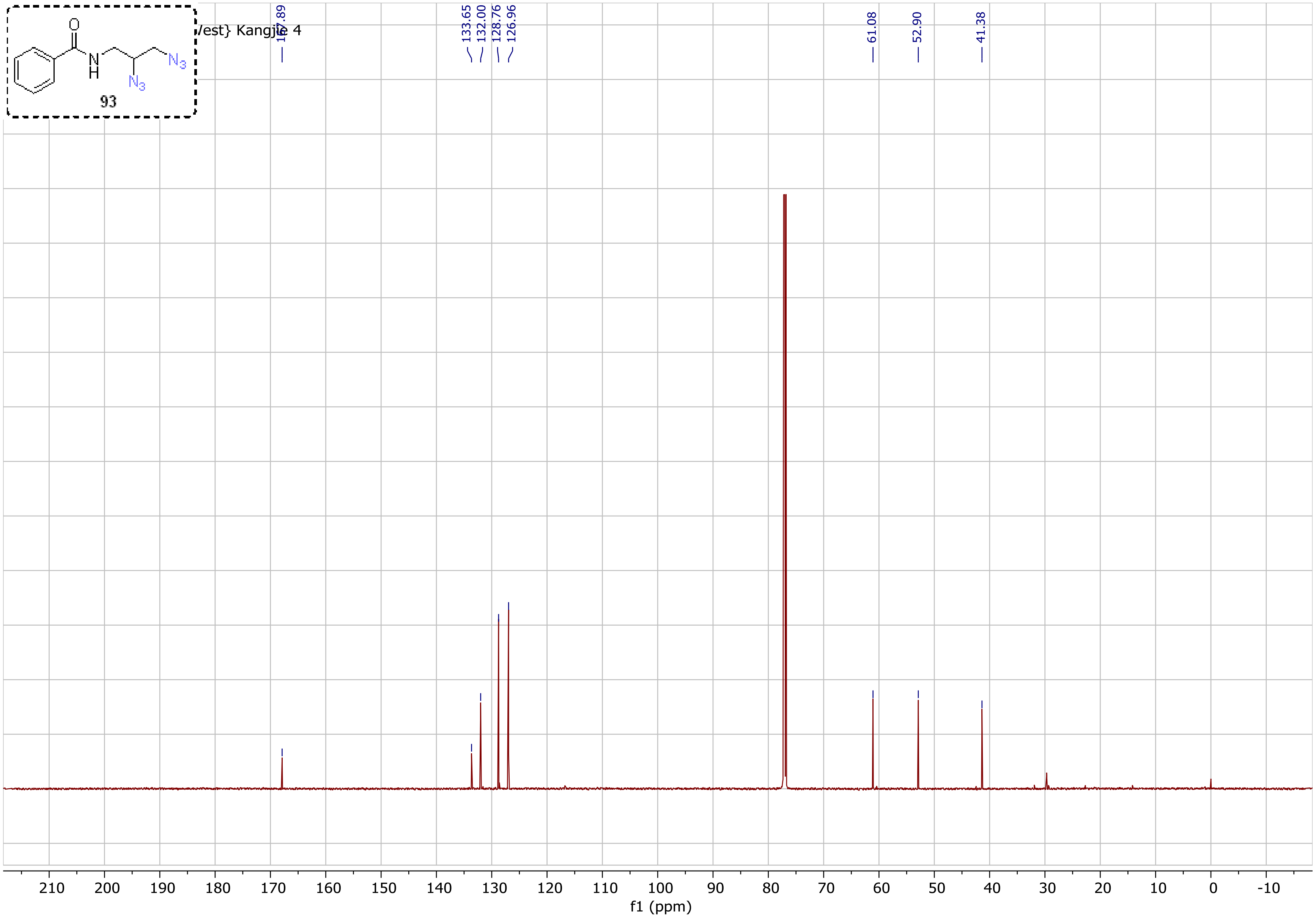

93
Kangjie 4
133.65
132.00
128.76
126.96
61.08
52.90
41.38
210 200 190 180 170 160 150 140 130 120 110 100 90 80 70 60 50 40 30 20 10 0 -10
f1 (ppm)

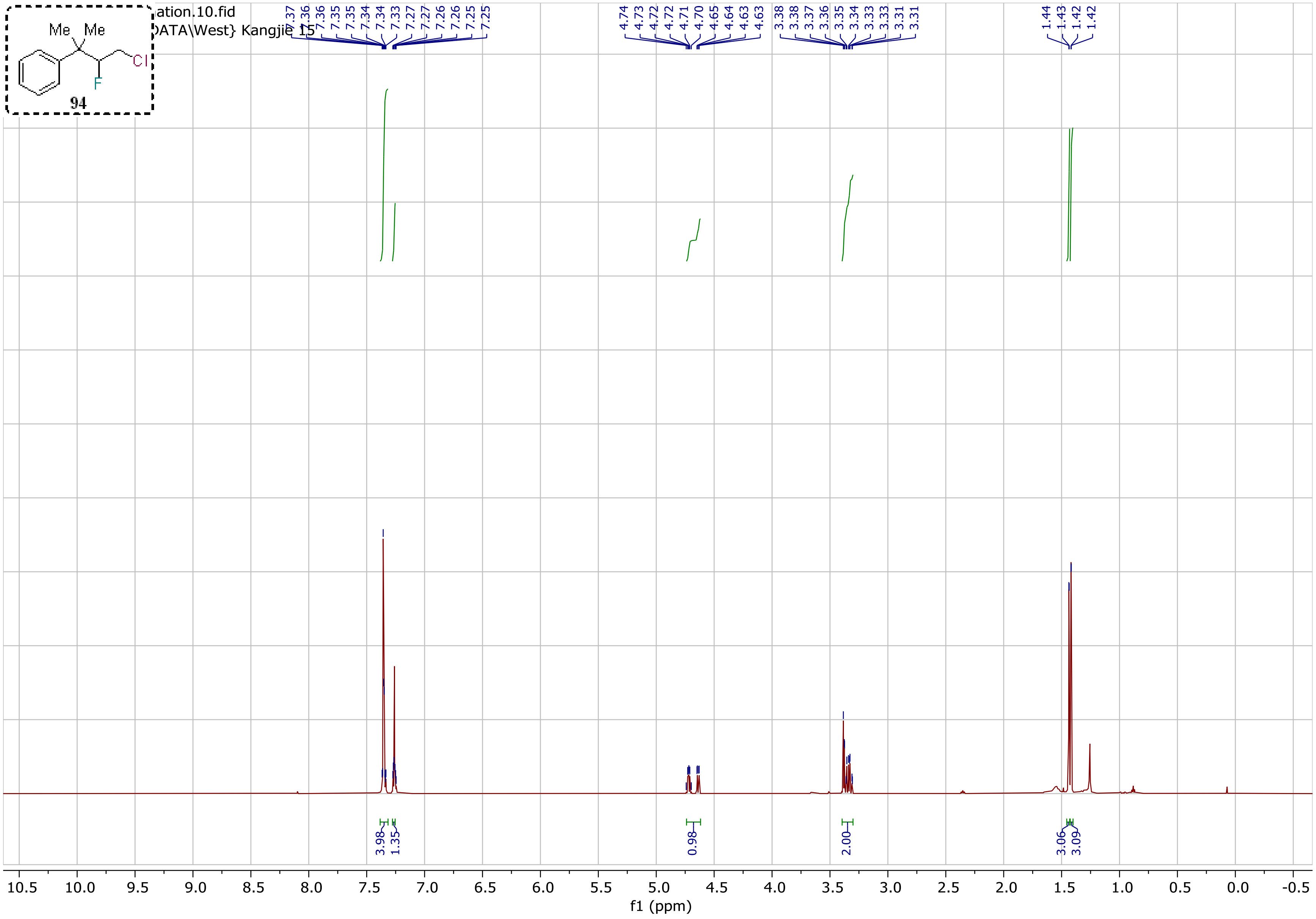

ation.10.fid
DATA\West} Kangjie 15
Me Me
F
Cl
94
7.37 7.36 7.36 7.35 7.35 7.34 7.34 7.33 7.27 7.27 7.26 7.26 7.25 7.25
4.74 4.73 4.72 4.72 4.71 4.70 4.65 4.64 4.63 4.63
3.38 3.38 3.37 3.36 3.35 3.34 3.33 3.33 3.31 3.31
1.44 1.43 1.42 1.42
3.98 1.35 0.98 2.00 3.06 3.09
f1 (ppm)

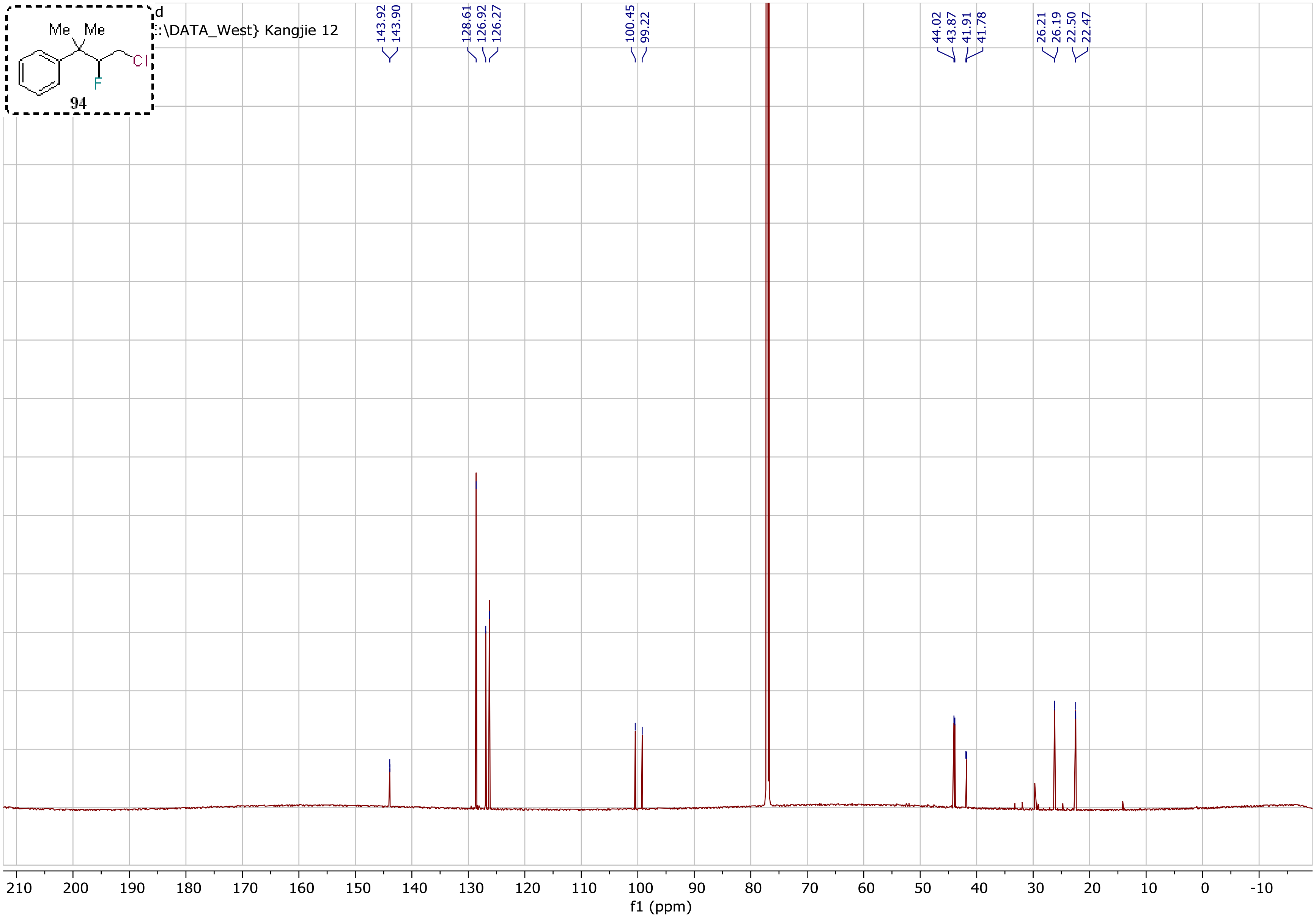
d
::\DATA_West} Kangjie 12
Me Me
Cl
F
94
143.92
143.90
128.61
126.92
126.27
100.45
99.22
44.02
43.87
41.91
41.78
26.21
26.19
22.50
22.47
210 200 190 180 170 160 150 140 130 120 110 100 90 80 70 60 50 40 30 20 10 0 -10
f1 (ppm)

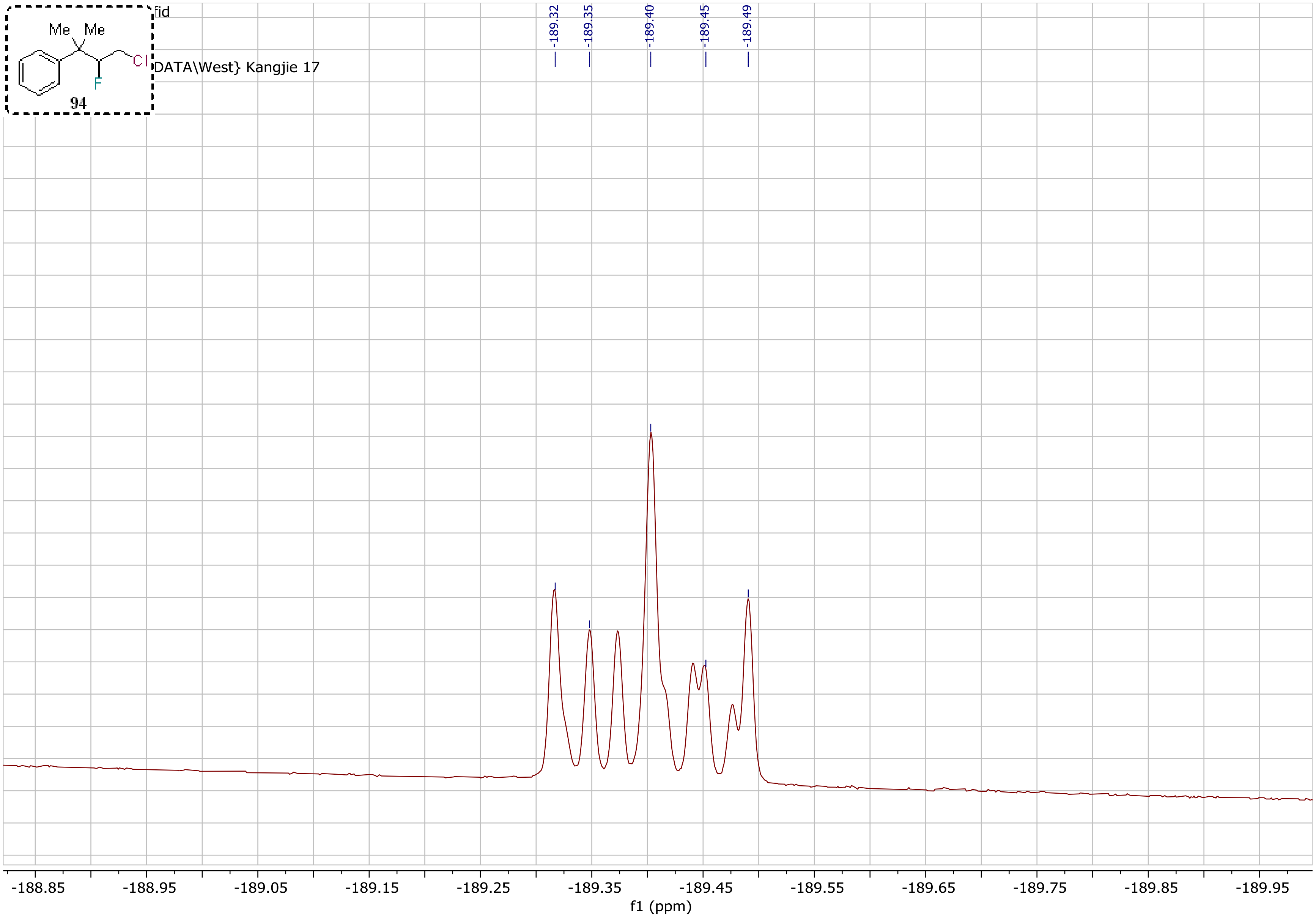
Me Me
Cl
F
94
fid
DATA\West} Kangjie 17
-189.32
-189.35
-189.40
-189.45
-189.49
-188.85
-188.95
-189.05
-189.15
-189.25
-189.35
-189.45
-189.55
-189.65
-189.75
-189.85
-189.95
f1 (ppm)

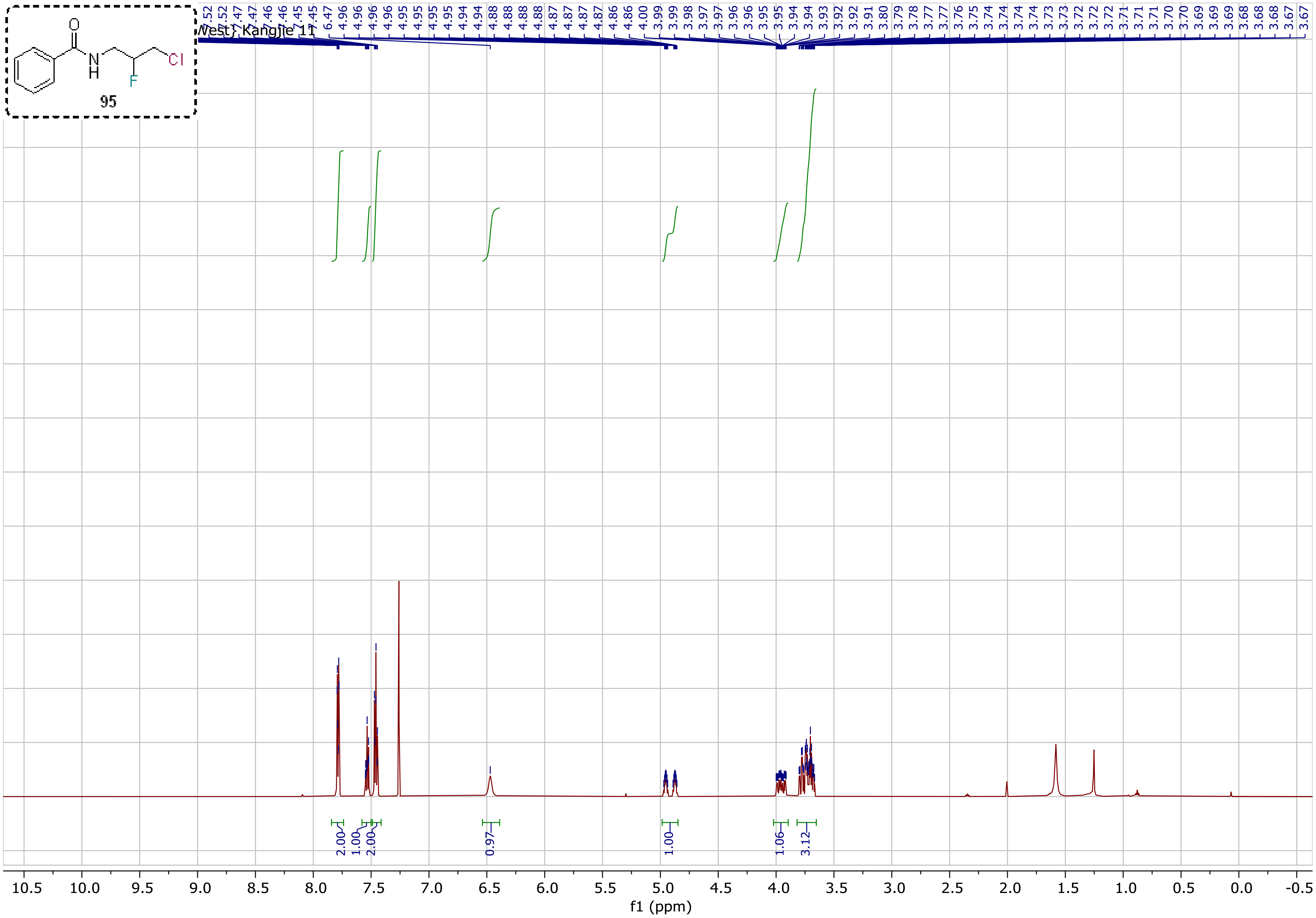

95
f1 (ppm)

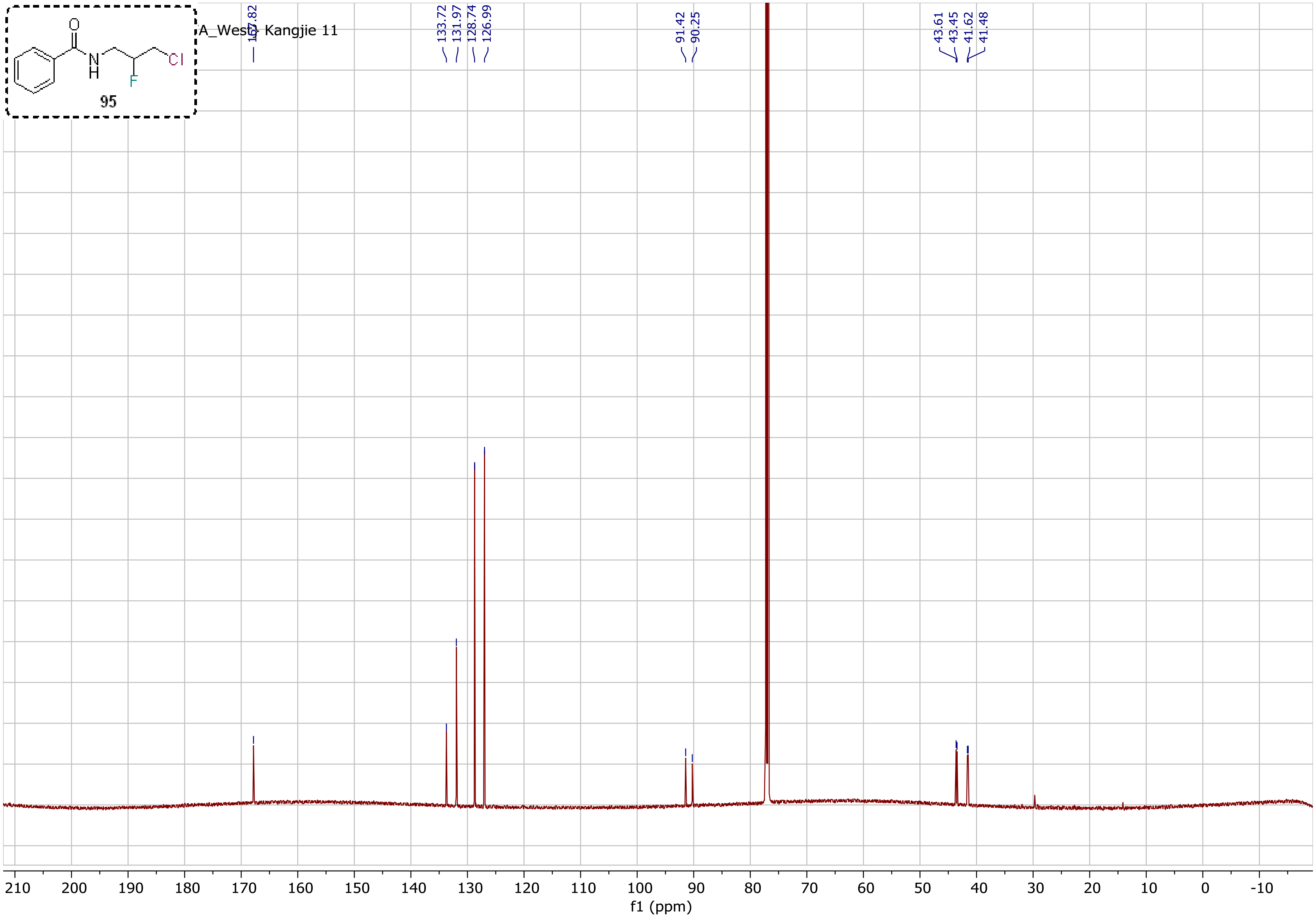

O
N
H
F
Cl
95
A_West- Kangjie 11
167.82
133.72
131.97
128.74
126.99
91.42
90.25
43.61
43.45
41.62
41.48
210 200 190 180 170 160 150 140 130 120 110 100 90 80 70 60 50 40 30 20 10 0 -10
f1 (ppm)

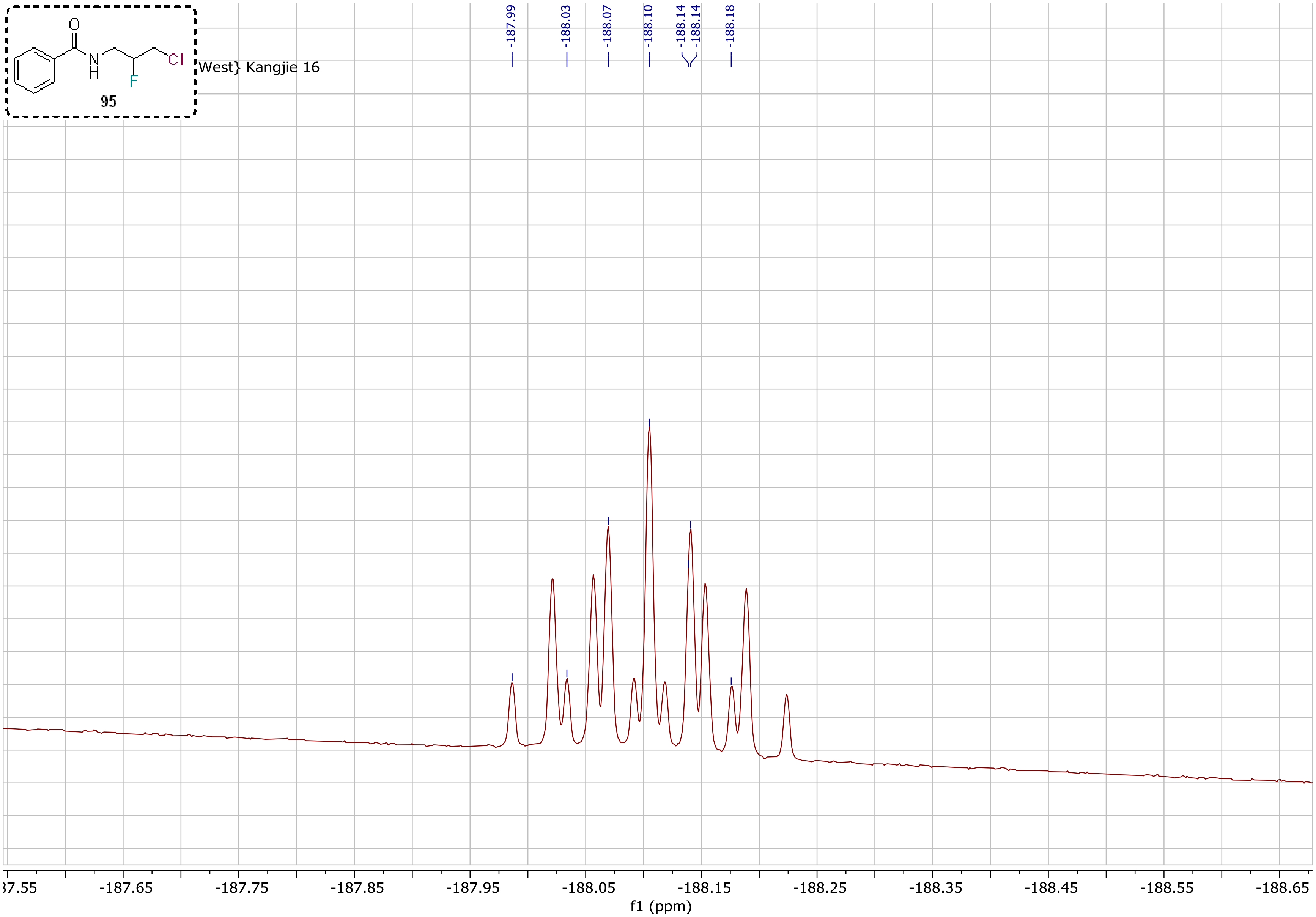
95
West} Kangjie 16
-187.99
-188.03
-188.07
-188.10
-188.14
-188.14
-188.18
-187.65
-187.75
-187.85
-187.95
-188.05
-188.15
-188.25
-188.35
-188.45
-188.55
-188.65
f1 (ppm)

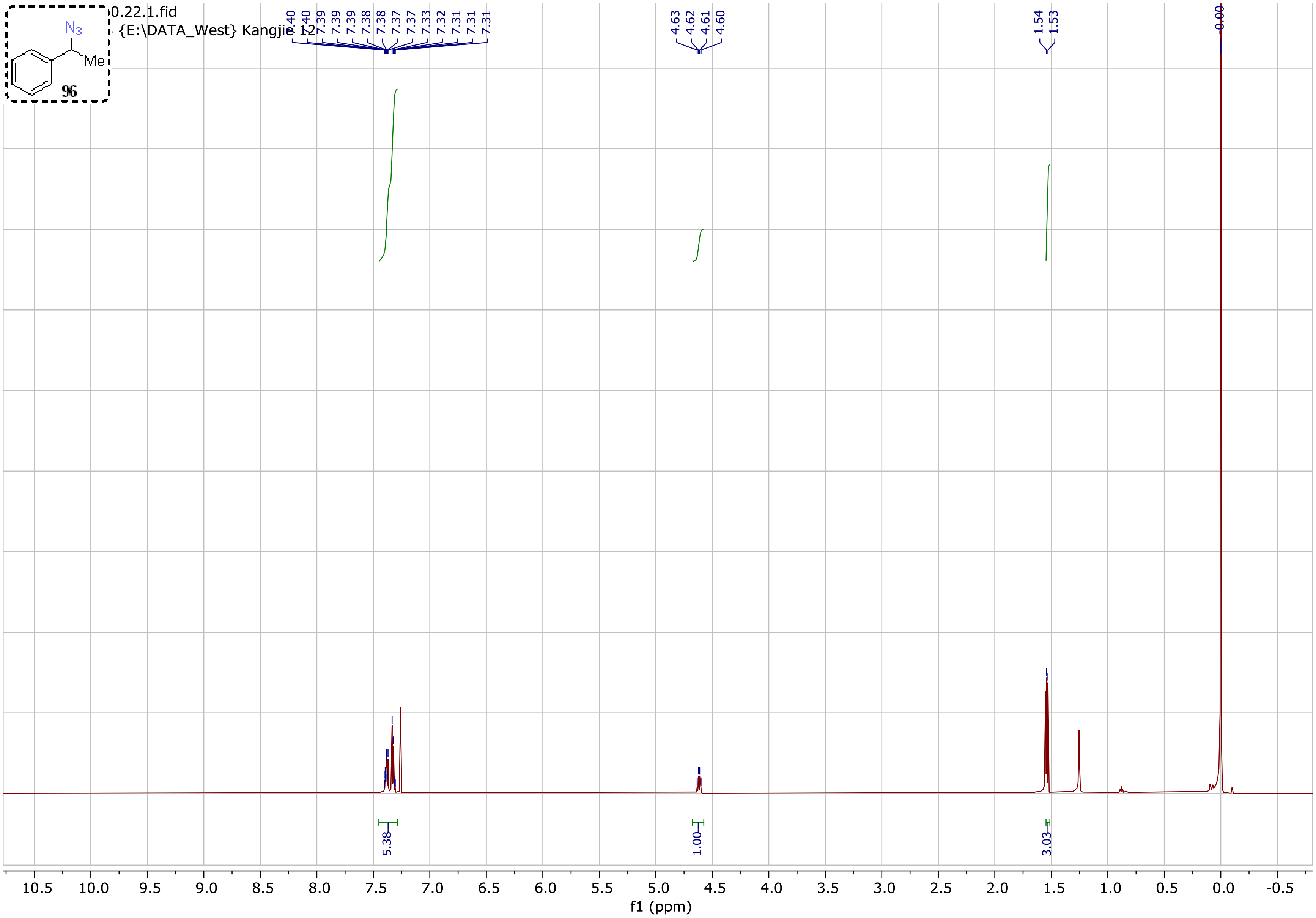

0.22.1.fid
{E:\DATA_West} Kangjie 12
N3
Me
96
7.40
7.40
7.39
7.39
7.38
7.38
7.37
7.37
7.33
7.32
7.31
7.31
7.31
4.63
4.62
4.61
4.60
1.54
1.53
0.00
5.38
1.00
3.03
f1 (ppm)

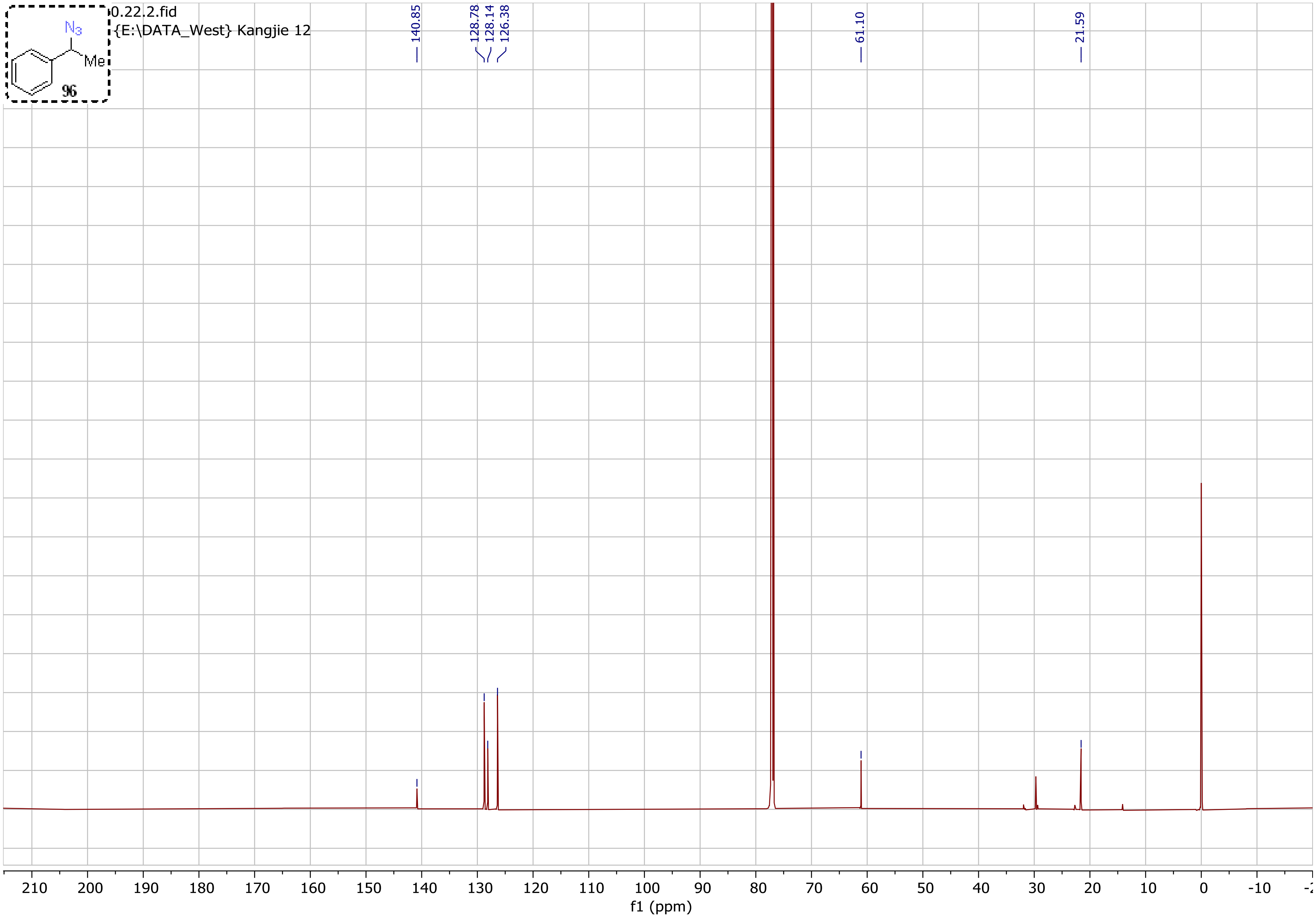

N3
Me
96
0.22.2.fid
{E:\DATA_West} Kangjie 12
140.85
128.78
128.14
126.38
61.10
21.59
f1 (ppm)

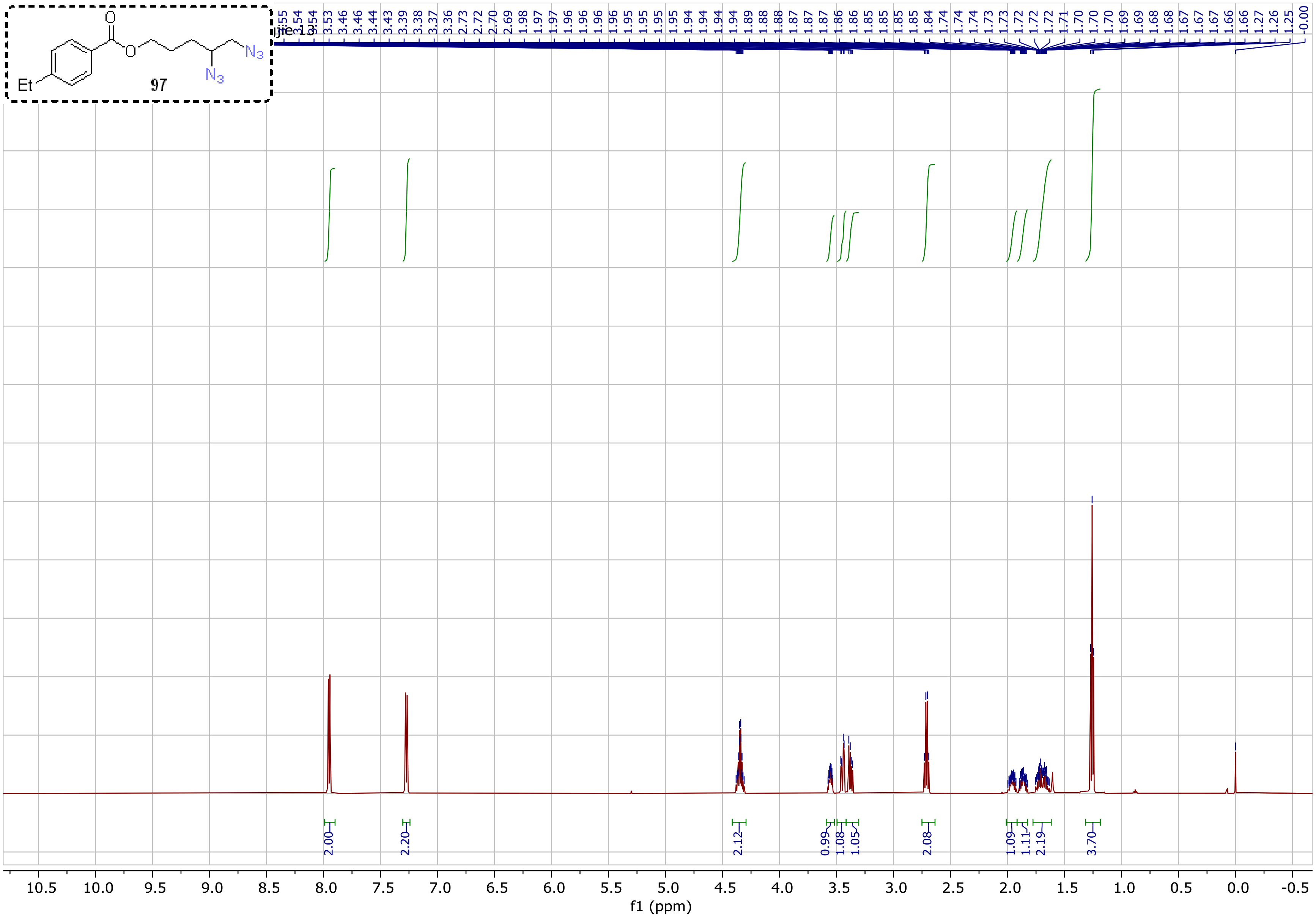
97
f1 (ppm)

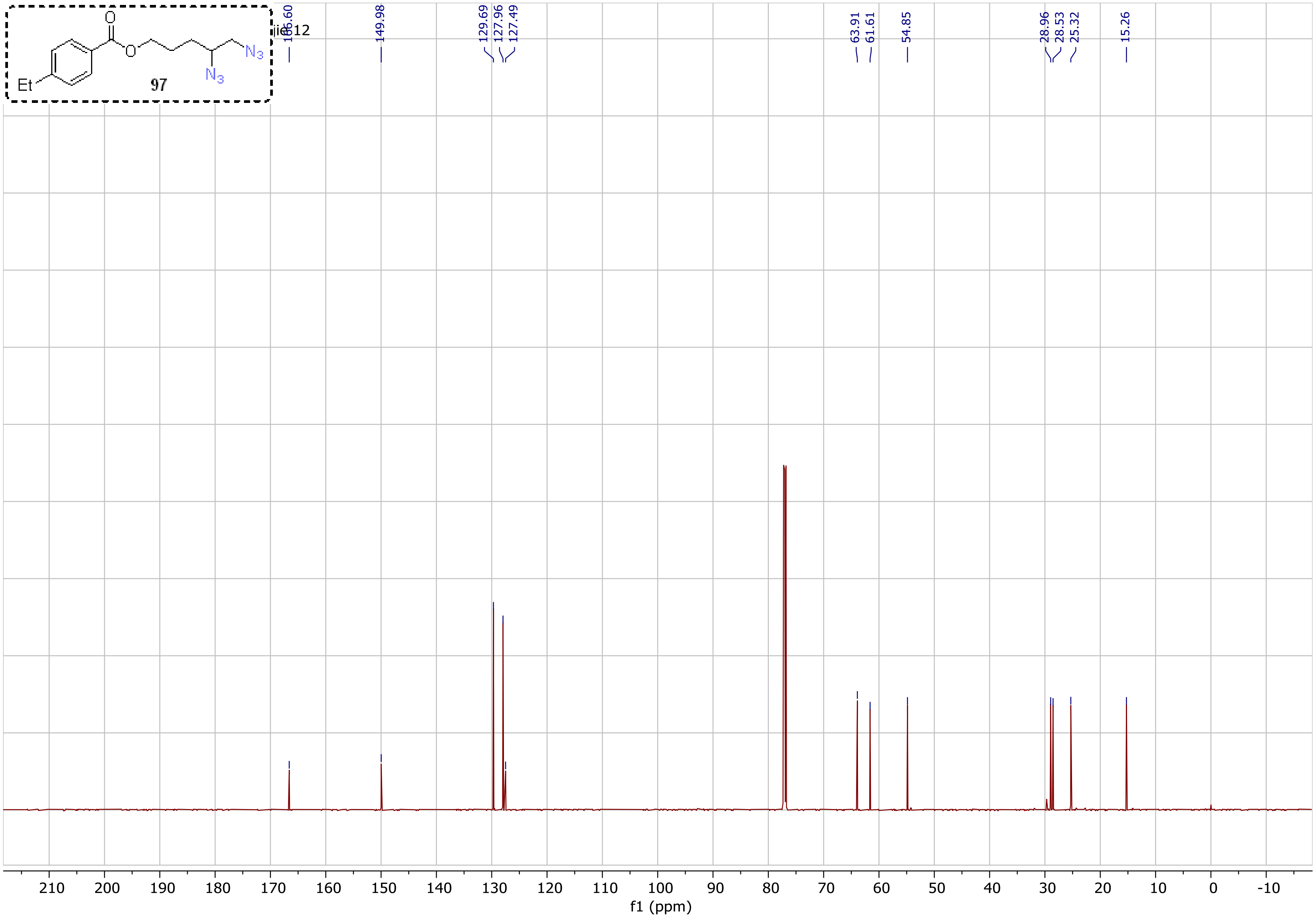
O
O
Et
N3
N3
97
166.60
149.98
129.69
127.96
127.49
63.91
61.61
54.85
28.96
28.53
25.32
15.26
210 200 190 180 170 160 150 140 130 120 110 100 90 80 70 60 50 40 30 20 10 0 -10
f1 (ppm)

IX. References

[1] Nikitas, N. F.; Voutyritsa, E.; Gkizis, P. L.; Kokotos, C. G. *Eur. J. Org. Chem.* **2021**, *2021*, 96.

[2] Liu, J.; Lu, C.; Wu, S.; Wu, X.; Wu, Z.; Xue, X.-S.; Yu, J.; Zhu, C. *Angew. Chem. Int. Ed.* **2020**, *59*, 8195.

[3] Jiang, H.-J.; Liu, K.; Yu, J.; Zhang, L.; Gong, L.-Z. *Angew. Chem. Int. Ed.* **2017**, *56*, 11931.

[4] C. Almansa, J. Alfón, A. F. de Arriba, F. L. Cavalcanti, I. Escamilla, L. A. Gómez, et al. *J. Med. Chem.* **2003**, *46*, 3463.

[5] Aldrich, C.; Brody, S.; Cushman, M.; Fan, B.-Z.; Hiasa, H.; Liang, J.-H.; Lv, W.; Yang, Z.-Y. *Eur. J. Med. Chem.* **2020**, *193*, 112222.

[6] Bian, K.-J.; Kao, S.-C.; Nemoto, D.; Chen, X.-W.; West, J. G. *Nature Communications* **2022**, *13* (1), 7881.

[7] Scheurer, A.; Mosset, P.; Saalfrank, R. W. *Tetrahedron: Asymmetry* **1997**, *8* (8), 1243-1251.

[8] Siu, J. C.; Parry, J. B.; Lin, S. *Journal of the American Chemical Society* **2019**, *141* (7), 2825-2831.

[9] Peng, H.; Yuan, Z.; Chen, P.; Liu, G. *Chinese Journal of Chemistry* **2017**, *35* (6), 876-880.

[10] Fumagalli, G.; Rabet, P. T. G.; Boyd, S.; Greaney, M. F. *Angewandte Chemie International Edition* **2015**, *54* (39), 11481-11484.

[11] Ho, M. L.; Flynn, A. B.; Ogilvie, W. W. *The Journal of Organic Chemistry* **2007**, *72* (3), 977.

[12] Kamada, Y.; Kitamura, Y.; Tanaka, T.; Yoshimitsu, T. *Organic & Biomolecular Chemistry* **2013**, *11* (10), 1598.

[13] Fu, N.; Sauer, G. S.; Lin, S. *Journal of the American Chemical Society* **2017**, *139* (43), 15548.

[14] Tan, E. W.; Chan, B.; Blackman, A. G. *Journal of the American Chemical Society* **2002**, *124* (10), 2078-2079.

[15] Xia, J.-B.; Zhu, C.; Chen, C. *Journal of the American Chemical Society* **2013**, *135* (46), 17494.
